# Glycoproteomic analysis of the changes in protein N-glycosylation during neuronal differentiation in human-induced pluripotent stem cells and derived neuronal cells

**DOI:** 10.1038/s41598-021-90102-z

**Published:** 2021-05-27

**Authors:** Kazumasa Kimura, Takumi Koizumi, Takaya Urasawa, Yuki Ohta, Daisuke Takakura, Nana Kawasaki

**Affiliations:** Biopharmaceutical and Regenerative Sciences, Graduate School of Medical Life Science, Yokohama City University, 1-7-29 Suehiro-cho, Tsurumi-ku, Yokohama, 230-0045 Japan

**Keywords:** Biochemistry, Neuroscience, Stem cells

## Abstract

N-glycosylation of glycoproteins, a major post-translational modification, plays a crucial role in various biological phenomena. In central nervous systems, N-glycosylation is thought to be associated with differentiation and regeneration; however, the state and role of N-glycosylation in neuronal differentiation remain unclear. Here, we conducted sequential LC/MS/MS analyses of tryptic digest, enriched glycopeptides, and deglycosylated peptides of proteins derived from human-induced pluripotent stem cells (iPSCs) and iPSC-derived neuronal cells, which were used as a model of neuronal differentiation. We demonstrate that the production profiles of many glycoproteins and their glycoforms were altered during neuronal differentiation. Particularly, the levels of glycoproteins modified with an N-glycan, consisting of five *N*-acetylhexosamines, three hexoses, and a fucose (HN5H3F), increased in dopaminergic neuron-rich cells (DAs). The N-glycan was deduced to be a fucosylated and bisected biantennary glycan based on product ion spectra. Interestingly, the HN5H3F-modified proteins were predicted to be functionally involved in neural cell adhesion, axon guidance, and the semaphorin-plexin signaling pathway, and protein modifications were site-selective and DA-selective regardless of protein production levels. Our integrated method for glycoproteome analysis and resultant profiles of glycoproteins and their glycoforms provide valuable information for further understanding the role of N-glycosylation in neuronal differentiation and neural regeneration.

## Introduction

N-glycosylation is one of the most important post-translational protein modifications^1^. N-glycans are involved in various biological phenomena such as development^1–3^, pluripotency^4–6^, differentiation^5–8^, regeneration^9,10^, and carcinogenesis^11,12^. In the neural system, N-glycans play crucial roles in neuronal system development^13,14^, neuroplasticity^14,15^, differentiation^7,14^, the regulation of signal transduction^13^, and are involved in the onset of neurodegenerative diseases^16^. However, the structure and functions of protein N-glycans in neuronal stem cells, progenitors, and neurons remain unclear due to the limited methods for the comprehensive analysis of cellular glycoproteins and the lack of appropriate models of human neuronal differentiation and regeneration. Delineating glycoprotein production and N-glycosylation during neuronal differentiation is crucial for understanding the molecular mechanism of neuronal differentiation, the onset mechanism of neurodegenerative diseases, and the development of therapeutic agents.

Generally, the cellularly expressed proteins can be identified using a comprehensive proteomic approach composed of LC/MS/MS of tryptic digests and data analysis^17–21^. The changes in a cell’s proteomic profile are associated with biological phenomena; some changes can be predicted with several tools such as the Gene Ontology (GO) enrichment analysis^22,23^ and pathway analysis^24,25^. In contrast, only a limited number of cellular glycoproteins can be identified because the measurement of glycopeptides by LC/MS/MS is challenging due to a high degree of heterogeneity and ion-suppression by coexisting unmodified peptides^26–28^. Furthermore, the mixture of glycan, peptide, and glycopeptide fragments makes product ion spectra very complex, thus hindering the processing of the LC/MS/MS data of glycopeptides^29,30^. Although automatic analysis software has become available for data processing^31,32^, failure to unequivocally identify glycoprotein remains a significant challenge.

Human-induced pluripotent stem cells (iPSCs) are useful for advancing regenerative medicine^33,34^, studying disease mechanisms^35,36^, and evaluating drug safety and efficacy in pharmaceutical development^36,37^. Human iPSC is known to differentiate into neural stem cells (NSC), neuronal progenitor cells (NPC), or various neurons^38–40^. These cells can be used as a model to elucidate proteins and the N-glycosylation associated with neuronal differentiation.

This study aims to characterize the production and N-glycosylation of glycoproteins in iPSCs, NSCs, NPCs, and neurons as a model of neuronal differentiation. Glycopeptides were enriched from the tryptic digests of cellular protein extraction with microcrystalline cellulose. Using LC/MS/MS, we conducted a sequential analysis of the tryptic digest, enriched glycopeptides, and deglycosylated peptides. The MS/MS data of glycoforms were processed with commercially available software and validated manually. The profiles of glycoproteins and their N-glycosylation obtained in this study would help further the understanding of the role of glycoproteins and N-glycosylation in neuronal differentiation, improve the quality of regenerative products, and enable the future discovery of molecular markers of neurodegenerative diseases.

## Results

### Experimental design for integrated glycoprotein analyses

**Figure 1:**
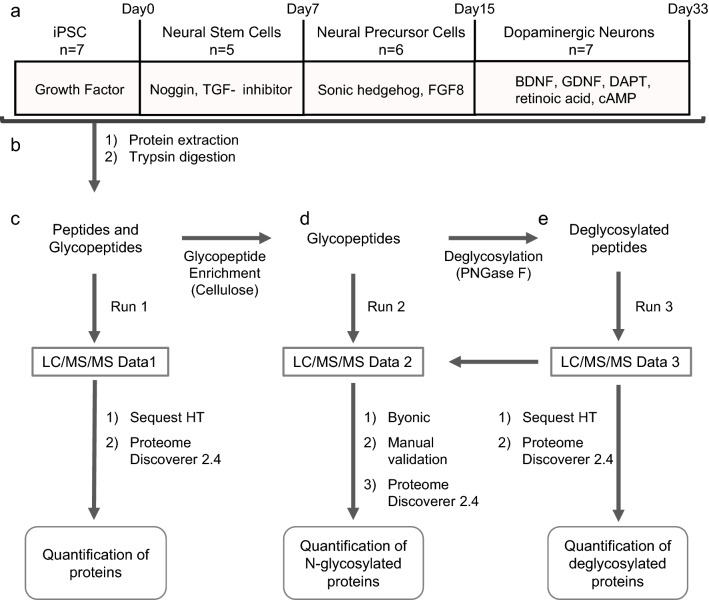
The workflow of the proteomic and glycoproteomic analyses. (**a**) Neuronal differentiation of iPSC. (**b**) Protein extraction and trypsin digestion. (**c**) Quantitative proteomic analysis. Label-free peptides and glycopeptides were analyzed by LC/MS/MS. (**d**) Quantitative glycoproteomic analysis. Cellulose-enriched glycopeptides were analyzed by LC/MS/MS. (**e**) Quantitative deglycosylated proteomic analysis. Peptide N-glycosidase F-treated glycopeptides were analyzed by LC/MS/MS.

According to the integrated glycoprotein analyses workflow (Fig. 1), iPSCs were differentiated into NSCs, NPCs, or dopaminergic neuron-rich cells (DA) (n = 5–7 in each stage) using a slightly modified method by Chambers et al*.* (2009)^38^. The differentiation stage of the cells was confirmed by immunostaining using stage-specific markers. Proteins were extracted from the cells in each stage and digested with trypsin. Each tryptic digest was divided into two tubes, one of which was used for a label-free relative quantification by LC/MS/MS (first run). The identification and relative quantification of proteins were performed using SequestHT and Proteome Discoverer 2.4, respectively. The remaining tryptic digest was enriched for glycopeptides with microcrystalline cellulose. Label-free quantification was performed with two-thirds of the enriched glycopeptides (second run). The glycopeptide composition was deduced by Byonic^41^, and quantitative analysis of glycoforms was performed by Proteome Discoverer 2.4. The remaining enriched glycopeptides were treated with peptide N-glycosidase F to remove the N-glycans and then subjected to proteomic analysis (third run). The glycoform data were manually evaluated by comparing the retention times and peptide-related product ion mass spectra from the second and third run.

### Immunofluorescence to estimate the differentiation stages of iPSC-derived cells

**Figure 2:**
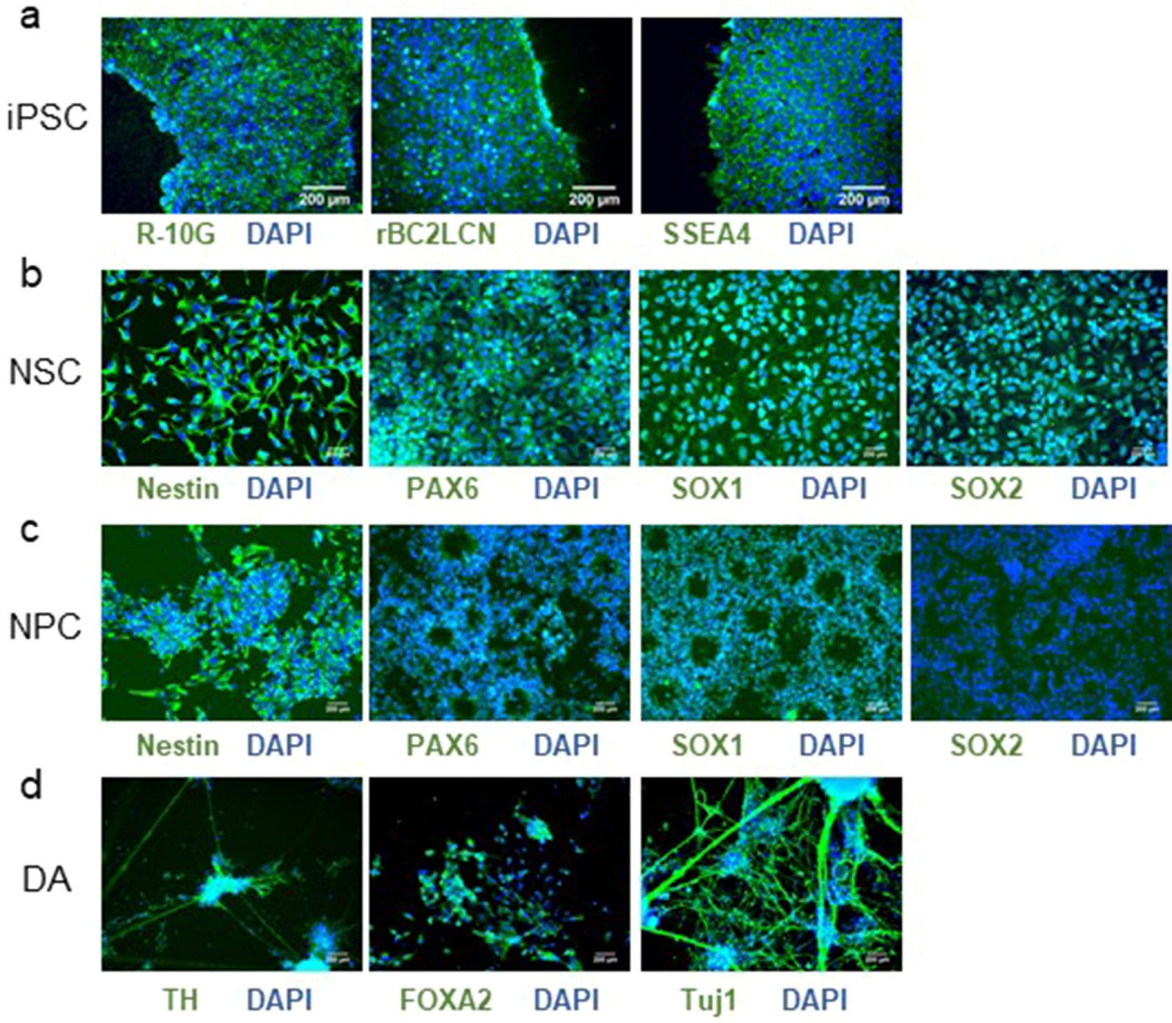
Immunofluorescence assays using antibodies against differentiation marker proteins. (**a**) R-10G (left panel), rBC2LCN (middle panel), and SSEA4 (right panel) in the membrane of the iPS cells. (**b**) Nestin (left) in the cytoplasm, PAX6 (second left) in the nucleus, SOX1 (second right) in the nucleus, and SOX2 (right) in the nucleus of the iPSC-derived neural stem cells. (**c**) Nestin (left) in the cytoplasm, PAX6 (second left) in the nucleus, SOX1 (second right) in the nucleus, SOX2 (right) in the nucleus in the iPSC-derived neural progenitor cells. (**d**) Tyrosine hydroxylase (left) in the cytoplasm, FOX2 (middle) in the nucleus, and Tuj1 (right) in the cytoplasm in the iPSC-derived dopamine neurons. The staining was green for different antibodies and blue for 4′,6-diamidino-2-phenylindole (DAPI). Scale bars: 200 μm.

The differentiation stages of the cells were estimated by immunostaining using commonly used stage-specific markers. The iPSCs were confirmed by the presence of R-10G, rBC2LCN, SSEA4 on the cell surface (Fig. 2a). Nestin in the cytoplasm and PAX6/SOX1in the nucleus were stained after culturing for 7 and 15 days (Fig. 2b,c). The presence of SOX2 was observed in the nucleus of cells after 7 days, but it disappeared after 15 days (Fig. 2b,c). These observations suggest that the cells at 7 days and 15 days may be classified as NSC and NPC, respectively. The presence of tyrosine hydroxylase in the cytoplasm, FOXA2 in the nucleus, and neuron-marker Tuj1in the cytoplasm suggested differentiation into neurons containing dopaminergic cells (DA) (Fig. 2d).

### Quantitative analysis of proteins in iPSC-derived cells

**Figure 3:**
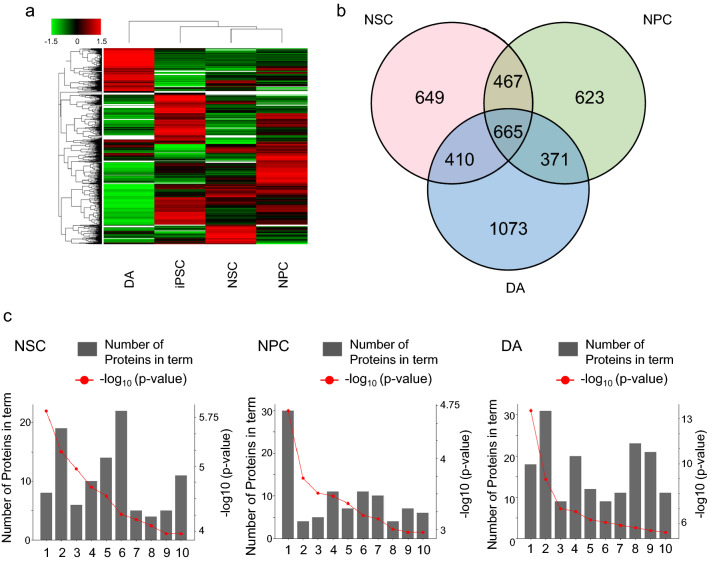
Proteomic datasets of all the proteins in the iPSCs, NSCs, NPCs, and DAs. (**a**) The heat map of all the identified proteins in the iPSCs, NSCs, NPCs, and DAs. (**b**) The Venn diagrams of proteins with increased levels in the NSCs, NPCs, and DAs compared to the iPSCs (*p*-value < 0.05). (**c**) GO enrichment analysis of the proteins with increased levels in the NSCs (left), NPCs (middle), and DAs (right) compared to the iPSCs (*p*-value < 0.05). Left: 1. glutathione derivative biosynthetic process, 2. brain development, 3. semaphorin-plexin signaling pathway involved in axon guidance, 4. glutathione metabolic process, 5. branchiomotor neuron axon guidance, 6. central nervous system development, 7. nitrobenzene metabolic process, 8. axon development, 9. regulation of cardiac muscle contraction by regulation of the release of sequestered calcium ion, 10. nervous system development. Middle: 1. cell adhesion, 2. mesenchyme migration, 3. protein localization to synapse, 4. dendrite morphogenesis, 5. muscle contraction, 6. fatty acid beta-oxidation, 7. integrin-mediated signaling pathway, 8. synapse assembly, 9. aging, 10. positive regulation of establishment of protein localization to plasma membrane. Right: 1. neurotransmitter secretion, 2. nervous system development, 3. synaptic vesicle exocytosis, 4. axon guidance, 5. chemical synaptic transmission, 6. glutamate secretion, 7. regulation of cardiac conduction, 8. learning, 9. ion transmembrane transport, 10. synapse assembly.

The proteins extracted from the iPSCs and iPSC-differentiated cells were precipitated by adding chloroform/methanol and digested with trypsin. Some of the tryptic digests were subjected to label-free relative quantitative analysis with a data-dependent acquisition measurement (first run). A total of 12,780 unique proteins were identified (FDR < 1%) from the iPSCs, NSCs, NPCs, and DAs. A heat map of the differentially expressed proteins in the cells from four different differentiation stages showed that the protein profile of the DAs had undergone the most significant changes compared to the other cells (Fig. 3a).

Compared to the iPSCs, a total of 1,821 unique proteins increased (relative peak area > 2.0, *p*-value < 0.05), and 2,069 proteins decreased (relative peak area < 0.5, *p*-value < 0.05) during neural differentiation (Fig. 3b, Supplementary Table S1). The GO enrichment analysis was conducted to annotate the proteins based on their molecular function to characterize the proteins with increased levels in each stage. The proteins increased in the cells after 7 days and were predicted to be linked with brain development and axon guidance. The proteins that increased in the cells after 15 days were likely to be associated with synapse and dendrite morphogenesis. Based on the proteins that increased in the cells after 33 days, such as neurexins, synaptotagmins, and calcium-dependent secretion activators, these cells were strongly predicted to be involved in neuronal function (Fig. 3c, Supplementary Table S1). The alteration of protein profiles suggests that the iPSC-derived cells after 7, 15, and 33 days have the characteristics of NSCs, NPCs, and neurons, respectively.

### N-glycosylation analysis of membrane-associated proteins in iPSC-derived cells

**Figure 4:**
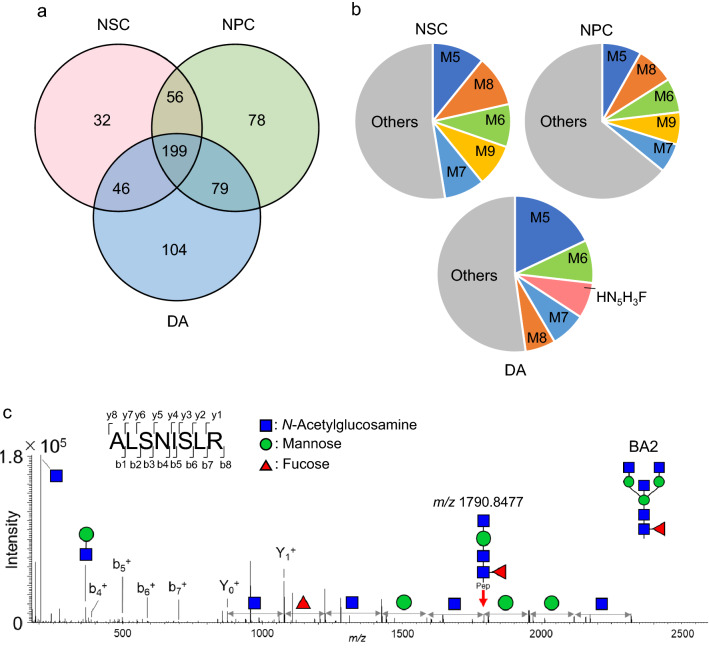
Glycoproteomic dataset of all N-glycosylated peptide glycoforms in the iPSCs, NSCs, NPCs, and DAs. (**a**) The Venn diagram of glycoproteins with increased levels (relative peak area > 2.0, p < 0.05) in NSCs, NPCs, and DAs compared to iPSCs. (**b**) Percentage of increased N-glycan forms (relative peak area > 2.0, p < 0.05) in the NSCs, NPCs, and DAs compared to the iPSCs. (**c**) Product ion spectrum of plexin B2 glycopeptide arising from the precursor ion at *m/z* 1,261.0690 (charge: 2). The product ion at *m/z* 1,790.8477 (charge: 1) suggests the presence of bisecting GlcNAc.

N-Glycopeptides were enriched by microcrystalline cellulose from the remaining tryptic digest of membrane proteins; two-thirds of the enriched sample was analyzed by LC/MS/MS (second run). The carbohydrate composition and the peptide sequence of the 7,192 glycoforms, derived from 2,192 glycopeptides and 1,149 glycoproteins, were deduced from the MS/MS data using the commercially available data processing software Byonic. We manually excluded suspicious glycoproteins based on the presence of unfamiliar glycans, such as fucosyl high-mannose type glycans, NeuGc-modified glycans, HexNAc_2_Hex_10-12_ glycans, and glycans below the trimannosyl core, narrowing the analysis to 6,590 glycoforms. The relative quantification of each glycoform was achieved by comparing the peak area of the iPSCs to that of the other cells (Supplementary Table S1). The number of glycoproteins that increased in the NSCs, NPCs, and DAs compared to those in the iPSCs (relative peak area > 2.0, *p*-value < 0.05) are shown in Fig. 4a. The comprehensive analysis of the enriched glycopeptides with LC/MS/MS succeeded in identifying 354 more glycoproteins that failed to be identified by the previous comprehensive analysis of the tryptic digests.

The percentage of N-glycan forms that increased in the NSCs, NPCs, and DAs compared to those in the iPSCs (relative peak area > 2.0, *p*-value < 0.05) is shown in Fig. 4b. It was suggested that the increase in glycoproteins after neuronal differentiation was mostly modified with high-mannose type glycans. Compared to the iPSCs, the NSCs and NPCs had more HexNAc_2_Hex_5_ (Man5) and HexNAc_2_Hex_8_ (Man8), whereas DA had more Man5 and HexNAc_2_Hex_6_ (Man6). Interestingly, the level of an N-glycan, composed of five HexNAcs, three Hexs, and a Fuc (HN_5_H_3_F), significantly increased only in the DAs. The HN_5_H_3_F-modified glycoforms were not detected in the iPSCs and were detected in low levels in the NPCs.

The structure of HN_5_H_3_F may be a triantennary glycan or a bisected biantennary glycan. We manually analyzed the product ion spectra of HN_5_H_3_F-modified proteins to determine their structures. In the representative product ion spectrum acquired from the plexin B2 glycopeptide (Fig. 4c), a product ion of *m/z* 1,790.8477 (charge: 1), which corresponds to a peptide bearing HN_5_H_3_F, suggests the presence of bisecting GlcNAc. The presence of the diagnostic ion related to bisected glycopeptides was observed in the product ion mass spectra acquired from 23 glycopeptides (Supplementary Figure S1). The diagnostic product ions were not detected in the product ion spectra acquired from 87 glycopeptides, which is likely due to the lower intensity of the precursor ions.

### Analysis of HN_5_H_3_F-modified glycoproteins

**Figure 5:**
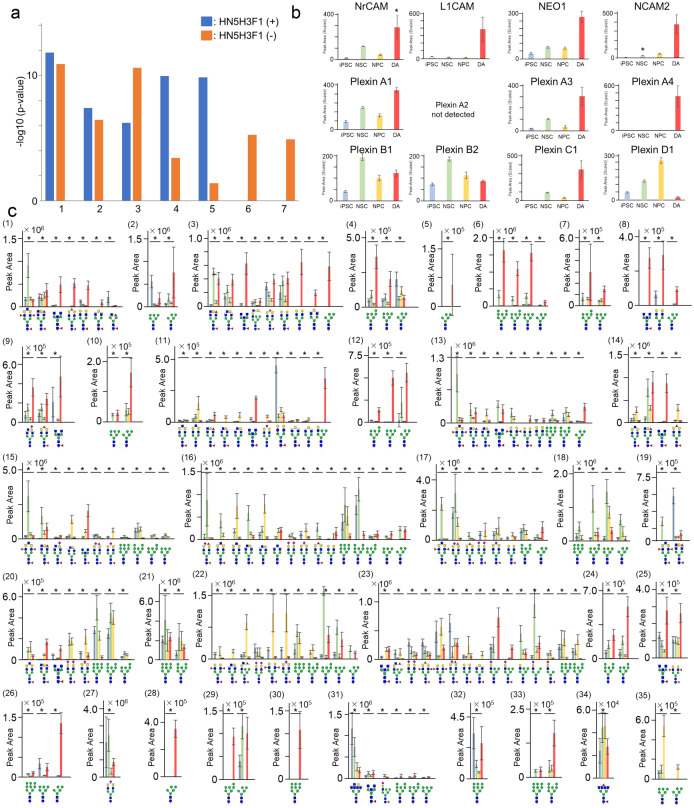
Analysis of the HN5H3F-modified glycoproteins. (**a**) The GO enrichment analysis of the HN_5_H_3_F-modified and unmodified proteins in the DAs. (1) Cell adhesion, (2) homophilic cell adhesion via membrane adhesion molecules, (3) axon guidance, (4) semaphorin-plexin signaling pathway, (5) negative chemotaxis, (6) ion transmembrane transport, (7) extracellular matrix organization. (**b**) The levels of NrCAM, L1CAM, NCAM2, NEO1, and plexins A1–A4, B1, B2, C1, and D1 proteins in the iPSCs, NSCs, NPCs, and DAs. *: Adj. *p* value < 0.05. (**c**) Glycoform peak area of plexins. Blue bar: iPSC, Green bar: NSC, Yellow bar: NPC, Red bar: DA. (1) PlexinA1 N74, (2) PlexinA1 N700, (3) PlexinA1 N1042, (4) PlexinA1 N1091, (5) PlexinA2 N73, (6) PlexinA2 N156, (7) PlexinA2 N653, (8) PlexinA3 N56, (9) PlexinA3 N1008, (10) PlexinA3 N1062, (11) PlexinA3 N1113, (12) PlexinA4 N442, (13) PlexinB1 N1180, (14) PlexinB1 N1248, (15) PlexinB2 N124, (16) PlexinB2 N517, (17) PlexinB2 N728, (18) PlexinB2 N753, (19) PlexinB2 N788, (20) PlexinB2 N844, (21) PlexinB2 N1000, (22) PlexinB2 N1031, (23) PlexinB2 N1084, (24) PlexinC1 N82, (25) PlexinC1 N132, (26) PlexinC1 N149, (27) PlexinC1 N550, (28) PlexinC1 N678, (29) PlexinC1 N690, (30) PlexinC1 N706, (31) PlexinC1 N820, (32) PlexinC1 N866, (33) PlexinC1 N914, (34) PlexinC1 N1078, (35) PlexinD1 N695. Blue bar: iPSC, Green bar: NSC, Yellow bar: NPC, Red bar: DA. *: *q*-value 2D < 0.01.

We conducted a GO functional annotation to characterize the function of HN_5_H_3_F-modified glycoproteins detected in the DAs. Both the HN_5_H_3_F-modified glycoproteins and unmodified glycoproteins were predicted to be involved in cell adhesion and axon guidance. The HN_5_H_3_F-modified glycoproteins were presumed to be involved in the semaphorin-plexin signaling pathway and negative chemotaxis, but unmodified glycoproteins were not. In contrast, glycoproteins not modified with HN_5_H_3_F in the DAs were suggested to be associated with the ion transmembrane transport and extracellular matrix organization, whereas no association was found with HN_5_H_3_F-modified proteins (Fig. 5a; Supplementary Table S1).

We performed the Kyoto Encyclopedia of Genes and Genomes (KEGG)^42^ pathway enrichment analysis to further explore the signaling pathway in which HN_5_H_3_F-modified glycoproteins were involved. The HN_5_H_3_F-modified proteins were suggested to be significantly associated with cell adhesion (*p*-value = 8.4E−15) and axon guidance (*p-*value = 9.8E−10) (Table 1). The representative glycoproteins associated with cell adhesion included contactin 1, neuronal cell adhesion molecules (NrCAM), L1 cell adhesion molecule (L1CAM), neural cell adhesion molecule 2 (NCAM2), and neogenin 1 (NEO1), which were classified into the immunoglobulin superfamily^43^.

**Table 1:** KEGG pathway analysis of the HN_5_H_3_F-modified proteins in the DAs.

Term	Count	*p*-value	Gene name
Cell adhesion molecules (CAMs)	15	8.40E−15	CD276*, L1CAM, ALCAM, CNTN1*, CNTN2*, ITGAV, NEO1, NCAM1, NCAM2, NRXN1, NRXN3*, NFASC*, NLGN4X, NRCAM, PTPRF
Axon guidance	11	9.80E−10	L1CAM, PLXNA1*, PLXNA3*, PLXNB1, PLXNB2*, SEMA4C*, SEMA4D, SEMA4G, SEMA6A, SEMA7A, SLIT1

*The presence of diagnostic ion for bisecting GlcNAc was confirmed in the product ion spectrum.

Then, we explored whether the increase in the levels of HN_5_H_3_F-modified glycoforms was dependent on protein expressions or differentiation stages. The peak areas of peptides derived from the above glycoproteins in various differentiation stages were extracted from the LC/MS/MS data of the PNGase F-treated glycopeptides (third run), which is appropriate for quantifying glycoproteins because of the elimination of microheterogeneity. The levels of NrCAM, L1CAM, NCAM2, NEO1, plexins A1, A3, A4, B1, 2, C1, and D1 changed with neuronal differentiation (Fig. 5b). The levels of the cell adhesion proteins and plexins A1, A3, A4, and C1 increased in DAs, whereas those of plexins B1, B2, and D1 decreased in DAs. Using the LC/MS/MS data of N-glycosylated peptides (LC/MS/MS Data 2), we sorted the glycoforms derived from the glycoprotein to elucidate the glycosylation profile of each glycosylation site in each glycoprotein and extracted some glycoforms derived from NrCAM, L1CAM, NCAM2, NEO1, and plexins A1, A3, A4, B1, B2, C1, D1 in the iPSCs, NSCs, NPCs, and DAs (Fig. 5c; Supplementary Figure S1). In the detected range, the HN5H3F modification was limited to the sites at N1006 in NrCAM; N288, N668, N723, and N977 in L1CAM; N217 and N445 in NCAM2; N461 and N628 in NEO1; and N74 and N1042 in plexin A1; N56, N1008, and N1113 in plexin A3; N1180 and N1248 in plexin B1; N124, N844, and N1084 in plexin B2; and N132 and N820 in plexin C1. Interestingly, HN_5_H_3_F-modified glycoforms of plexins B1 and B2 increased in DAs despite a reduction of protein levels. These findings suggest that the HN_5_H_3_F modification is neuron-specific and independent of protein levels.

## Discussion

In this study, first, we conducted the quantitative analysis of proteins in the iPSCs, and iPSC-derived neuronal cells to elucidate the changes in protein production during neuronal differentiation. Label-free quantitative proteomic analysis showed that the level of the proteins associated with brain development and axon guidance was already enhanced after 7 days of differentiation. An increase in the proteins related to synapse and dendrite morphogenesis was found in the cells after 15 days of culture, where axon extension and dendritic growth had not been observed. These findings suggest that the proteins with increased levels may be used as neuronal differentiation markers to define NSCs and NPCs, which cannot be distinguished using current markers^44,45^, such as Nestin, PAX6, SOX1, and SOX2. Many proteins involved in neurotransmitter secretion, nervous system development, and synaptic vesicle exocytosis were identified in the DAs. The presence of gamma-aminobutyric acid (GABA) receptors and glutamine receptors as well as tyrosine hydroxylase suggests the differentiated cells are a mixture of various neurons. The lack of glial fibrillary acidic protein implies the absence of astrocyte in DAs^46^.

For cell-based therapies, the World Health Organization requires information on the identity, purity, and activation state of the critical cell type. The identity of the primary cell populations needs to be described at the phenotypic level (i.e., describe the cell surface expression profile with at least two cell surface markers and the functional level^47^). Our approach demonstrating the expression profile at various differentiation stages could help to define the cell surface expression profile and identify appropriate cell surface markers. Consequently, it can be possible to identify the primary cells and evaluate their purity and maturation, predict their differentiation, or compare different manufacturing processes.

Many glycoproteins are involved in neuronal differentiation and function; thus, glycoproteomics analysis was performed to explore the changes in N-glycosylation and anticipate its role in neuronal differentiation by using two improved methods. One of the methods was N-glycopeptide enrichment by microcrystalline cellulose, which successfully eliminated the coexisting peptides that caused the ion-suppression of glycopeptides and enabled the acquisition of appropriate mass spectra for glycosylation analysis. The peptide sequences and carbohydrate composition of each glycoform were deduced from the LC/MS/MS data by using the software Byonic followed by manual validation. Some unreliable results, such as fucosylated high-mannose type, NeuGc-modified glycans, and glycans that were shorter than trimannosyl core or longer than Man10 were manually excluded. Furthermore, the glycopeptides bearing N-glycans of interest were manually analyzed to confirm the peptide sequence and carbohydrate composition by examining the product ion spectra.

The other improvement in methodology was the sequential LC/MS/MS analysis for determining more glycoprotein structures, which consisted of quantitative proteomic, glycoproteomic, and deglycosylated proteomic analyses. In addition to abundant glycoproteins, such as integrins and laminins, the method also elucidated structures of lower levels of proteins related to neural functions such as semaphorin subtypes and plexin subtypes.

We demonstrated that glycosylation changed at each glycosylation site and in each glycoprotein during differentiation. The N-glycan structures tended to be simplified with reduced fucosylation and sialylation, and the levels of glycoproteins modified with an N-glycan, consisting of five HexNAc, three Hex, and a Fuc (HN_5_H_3_F), increased in DAs. Shimizu *et al*. discovered a brain-specific glycan in the mouse brain and reported that it was a fucosylated and bisected biantennary glycan (BA2) by HPLC with fluorescence detection of released N-glycans^48^. The HN_5_H_3_F glycan is considered identical to BA2 based on the presence of a bisected glycan-related fragment in the product ion spectra of the glycoforms. An increase in bisected glycans during neural differentiation has been also reported in mouse iPSCs and embryonic stem cells^49^, suggesting that this phenomenon may be common to mouse and human neural differentiation. Previous research has used lectins^50,51^ and released N-glycans^48,49,52^ but could not conclusively identify the proteins modified with BA2 and other bisected glycans. In the biosynthesis of N-glycans, beta-1,4 *N*-acetylglucosaminyltransferase (GnT-III) catalyzes the formation of bisecting GlcNAc. It has been reported that GnT-III is associated with Alzheimer's disease^53–55^. Akasaka-Manya *et al.* demonstrated that the level of GnT-III mRNA had significantly increased in AD brains compared to controls^54^, and Kizuka *et al.* indicated that GnT-III-deficient AD model mice showed reduced amyloid-β (Aβ) accumulation in the brain by suppressing the function of a key Aβ-generating enzyme, β-site APP-cleaving enzyme-1 (BACE1), and greatly improved AD pathology^55^. They found the altered BACE1 subcellular localization in GnT-III-deficient cells, from early endosomes to lysosomes and speculated that bisecting GlcNAc serves as a trafficking tag for the movement of modified proteins to an endosomal compartment. It is of interest whether BA2 affects the localization of proteins during neuronal differentiation.

In this study, we identified BA2-modified proteins and explored the functions of the proteins using GO analysis and KEGG pathway analysis. The BA2 modified proteins were predicted to be involved in neural cell adhesion, axon guidance, and the semaphorin-plexin signaling pathway. Particularly, proteins in the immunoglobulin superfamily were included in cell adhesion molecules. Here, it is known that axon guidance proteins in the axon’s growth cone are associated with neurite outgrowth in neuronal cells^56–58^. The plexin proteins are transmembrane receptors for semaphorin ligands. The semaphorin-plexin signaling pathway is thought to be essential in neuronal development such as axon guidance and cell migration^59,60^; however, roles of individual plexin and semaphorin subtypes in neuronal differentiation are still unclear. The levels of the cell adhesion proteins and plexins A1, A3, A4, and C1 increased in DAs, and BA2 modifications occurred at only limited sites of the proteins in DAs. In contrast, the levels of plexins B1, B2, and D1 increased in NPC and decreased in DAs. Interestingly, BA2-modified glycoforms in plexins B1 and B2 increased in DAs. These results suggest that the BA2 modification is protein-, site-, and differentiation stage-selective regardless of protein production levels, implicating that BA2 may be associated with neuronal differentiation.

Our integrated method for glycoproteome analysis, and resultant glycoprotein and glycoform profiles provided valuable information for understanding the role of N-glycosylation in neuronal differentiation. As only one iPSC line was used in this study, further validation with different genotypes and multiple independent clones will be needed to confirm the phenomenon found in this study and apply it to quality control of cell therapy products.

## Methods

### iPCS culture and differentiation into DA

The human iPS cell line iPSC 201B7 (HPS0063; CELLBANK, IBARAKI, JAPAN) was cultured in 5% CO_2_ on vitronectin (rhVTN-N; Thermo Fisher Scientific Inc., Waltham, MA, USA)-coated 6-well plates (TPP Techno Plastic Products AG Int., Klettgau, Switzerland) in Essential 8 medium (Thermo Fisher Scientific) at 37 °C.

The neural differentiation of iPSCs was based on the method of Chambers et al.^38^. NSCs were generated by culturing subconfluent iPSC 201B7 in a differentiation medium composed of 48.5% DMEM/F12 (FUJIFILM Wako Pure Chemical Inc., Osaka, Japan), 48.5% Neurobasal medium (Thermo Fisher Scientific), 1% N_2_ supplement (Thermo Fisher Scientific), 2% B-27 supplement (Thermo Fisher Scientific), 1% nonessential amino acids (NEAA; FUJIFILM Wako Pure Chemical), 2 mM L-Alanyl-L-Glutamine (FUJIFILM Wako Pure Chemical), 100 μM 2-Mercaptoethanol (β-ME; FUJIFILM Wako Pure Chemical), 100 nM LDN193189 (FUJIFILM Wako Pure Chemical), and 10 μM SB431542 (FUJIFILM Wako Pure Chemical) at 37 °C in 5% CO_2_ for 7 days. After washing with Dulbecco’s phosphate buffered saline without calcium and magnesium (DPBS (–); Nacalai tesque Inc., Kyoto, Japan) twice, the cells were incubated in EDTA (FUJIFILM Wako Pure Chemical)/DPBS (–) at 37 °C in 5% CO_2_ for 15 min; the unattached cells were removed. Fresh EDTA/DPBS (–) was added to the wells, and the attached cells were collected by pipetting. The supernatant was removed by centrifuging at 300 g for 5 min. The NSC was maintained in the culture medium consisting of 97% DMEM/F12, 1% N2 supplement, 2% B-27 supplement, 2 μg/L fibroblast growth factor 2 (FGF2; FUJIFILM Wako Pure Chemical), 2 μg/L epidermal growth factor (EGF; FUJIFILM Wako Pure Chemical), 2 mM l-Alanyl-l-Glutamine, and 1% penicillin–streptomycin (P/S: FUJIFILM Wako Pure Chemical) on the CELLstart (Thermo Fisher Scientific)-coated 6-well plates at 37 °C in 5% CO_2_.

The NSCs were differentiated into NPCs by culturing in a differentiation medium containing 99% DMEM/F12, 1% N2 supplement, 200 μg/L recombinant human sonic hedgehog protein (rhSHH: FUJIFILM Wako Pure Chemical), and 100 μg/L fibroblast growth factor 8 (FGF8: FUJIFILM Wako Pure Chemical) at 37 °C in 5% CO_2_ for 8 days. The confluent NPCs were cultured in a dopaminergic neuron (DA)-differentiation medium composed of 95% DMEM/F12, 1% N2 supplement, 2% B-27 supplement, 1% NEAA, 2 mM l-Alanyl-l-Glutamine, 1% P/S, 20 μg/L rh- brain-derived neurotrophic factor (BDNF; FUJIFILM Wako Pure Chemical), 20 μg/L rh glia-derived neurotrophic factor (GDNF; FUJIFILM Wako Pure Chemical), 1 μM *N*-[*N*-(3,5-Difluorophenacetyl-l-alanyl)]-S-phenylglycine t-butyl ester (DAPT, (FUJIFILM Wako Pure Chemical), 1 μM all-trans-retinoic acid (FUJIFILM Wako Pure Chemical), and 0.5 mM dibutyryl-cAMP (Santa Cruz Biotechnology Int., Dallas, TX, USA) at 37 °C in 5% CO_2_ for 18 days.

### Immunostaining

The cells in a 24-well plate (TPP) were washed with DPBS (–) twice; the supernatant was removed. Methanol (FUJIFILM Wako Pure Chemical) was added to the cells to incubate for 30 min. For staining with SSEA4 and R-10G, the wells were incubated with 4%paraformaldehyde for 15 min, and then 0.1% saponin for 15 min. The solution was replaced by DPBS (–) containing 3% BSA (Nacalai tesque) to incubate for 1 h. The supernatant was removed, and the cells were incubated with primary antibodies at 4 °C for 16 h. The antibodies against R-10G (1/500 diluted; 017–25813; FUJIFILM Wako Pure Chemical), rBC2LCN (1/500; 180–02991; FUJIFILM Wako Pure Chemical), and SSEA4 (1/500; MA1‐021; Thermo Fisher Scientific) for iPSCs were used. Also, antibodies against Nestin (1/100; MA1‐110; Thermo Fisher Scientific), PAX6 (1/150; MA1‐109; Thermo Fisher Scientific), SOX1 (1/100; MA5‐32447; Thermo Fisher Scientific), and SOX2 (1/100; MA1‐014; Thermo Fisher Scientific) for NSCs and NPCs were used.

The differentiation into DA was confirmed with antibodies against tyrosine hydroxylase (TH; 1/100; PA5-17800; Thermo Fisher Scientific), FOXA2 (PA5-35033; Thermo Fisher Scientific), and tubulin β3 (1/100, Tuj1; MMS-435P; Thermo Fisher Scientific). The supernatant was removed. The cells were rinsed with DPBS (–) containing 0.05% Tween 20 (Sigma-Aldrich Int., St. Louis, MO, USA, PBST) and incubated with goat anti-mouse IgG Alexa Fluor 488 (1/2000, A-11001; Thermo Fisher Scientific) or goat anti-rabbit IgG (1/1000, ab150077; Abcam Int., Cambridge, UK) at 4 °C for 16 h. After rinsing with 0.05% Tween20 containing PBS (PBST) three times, the cells were stained with 4',6-Diamidino-2-phenylindole (DAPI) by incubating in DAPI solution (0.5 μg/mL) at 25 °C for 10 min and washed. The cells in the 24-well plates were examined under a microscope Eclipse Ts2 (Nikon, Tokyo, Japan) at 488 nm for differentiation-specific markers and 385 nm for the nucleus.

### Extraction and tryptic digestion of membrane proteins

The cells were washed with DPBS (–) twice and mixed with 400 μL of M-PER (Thermo Fisher Scientific) containing protease inhibitor cocktail (Nacalai tesque) and *O*-GlcNAcase inhibitor (Merck-Millipore Int., Burlington, MA) for 5 min. The lysate was centrifuged at 12,000 × g for 5 min to collect the supernatant. The protein concentration was determined with the Bicinchoninic acid (BCA) protein assay. Then, 100 μg of protein was precipitated by adding chloroform/methanol. The precipitate was dissolved in 50 μL of 50 mM Tris–HCl pH 8.0 containing 8 M Urea (FUJIFILM Wako Pure Chemical). The denatured proteins were reduced by incubating with 1 μL of 500 mM dithiothreitol (DTT; FUJIFILM Wako Pure Chemical) at 37 ℃ for 30 min. Alkylation was performed by incubation with 2.8 μL of 500 mM iodoacetamide (IAA; FUJIFILM Wako Pure Chemical) at 25 ℃ in the dark for 30 min. The reaction was terminated by adding 0.5 μL of 500 mM DTT. The protein solution was diluted with 950 μL of 50 mM Tris–HCl pH 8.0 and incubated with 2 μg Trypsin/Lys-C (Promega Int., Madison, WI, USA) at 37 ℃ for 16 h. Desalting was performed with an Oasis PRiME HLB 1 cc Extraction Cartridge (Waters Corporation Int., Milford, CA, USA) according to the manufacturer’s instructions. The protein fraction was dried using a Speed Vac concentrator (Sakuma Int., Tokyo, Japan).

### Glycopeptide enrichment

One mL of 90% [v/v] acetonitrile (Kanto Chemical Int., Tokyo, Japan)/ 0.1% [v/v] trifluoroacetic acid (TFA: FUJIFILM Wako Pure Chemical) was added to a 20–40 μL of suspension of cellulose microcrystalline (Merck-Millipore) in 80% acetonitrile/0.1% TFA, and then mixed with 20–40 μg of tryptic digest in 5% [v/v] acetonitrile/0.1% [v/v] formic acid by inverting the tube once, and the tubes were mixed with a Mini Disk Rotor (BIO CRAFT Int., Tokyo, Japan) at 1 rpm for 15 min. The suspension was then centrifuged at 1,000 g for 1 min to remove the supernatant. The microcrystalline cellulose was washed with 1 mL of 80% [v/v] acetonitrile/0.1% [v/v] formic acid three times, suspended in 1 mL of 80% [v/v] acetonitrile /0.1% [v/v] formic acid, and centrifuged at 1,000 rpm for 1 min to remove the supernatant. Next, the cellulose was mixed with 200 μL of 30% [v/v] acetonitrile using a Mini Disc Rotor at 1 rpm for 15 min. The suspension was centrifuged at 1,000 rpm for 1 min, and 180 μL of the supernatant was moved to tube A. The remaining cellulose was mixed with 200 μL of 30% [v/v] acetonitrile using a Mini Disc Rotor at 1 rpm for 15 min. Afterward, the mixture was centrifuged at 1,000 g for 1 min before removing 200 μL of the supernatant to tube A. After tube A was centrifuged at 14,000 g for 10 min, the supernatant was moved to tube B and dried using the Speed Vac concentrator (Sakuma). Lastly, the dried glycopeptides were resuspended in 3% acetonitrile/0.1% formic acid.

### Deglycosylation

The dried 20 μg glycopeptides were redissolved in 50 mM Tris–HCl pH 8.0, and 2 units of peptide-*N*-glycosidase F (PNGaseF; Roche Inc., Basel, Switzerland) were added and incubated at 37 ℃ for 16 h. Desalting was performed with a C-tip SDB (Nikkyo Technos Int., Tokyo, Japan). The deglycosylated peptides were dried with the Speed Vac concentrator (Sakuma). Lastly, the dried glycopeptides were resuspended in 3% acetonitrile/0.1% formic acid.

### LC/MS/MS

One μg of the tryptic digest or 10 μg deglycosylated peptides were analyzed using an LC/MS/MS instrument composed of Q-Exactive (Thermo Fisher Scientific) and EASY-nLC1000 (Thermo Fisher Scientific) equipped with an Acclaim PepMapC18 (3 μm, 0.075 mm × 10 mm; Thermo Fisher Science) column and an NTCC-360/75–3-125 (C18, 3 μm, 0.075 × 125 mm; Nikkyo Technos) column. Solvent A was 0.1% formic acid, and solvent B was acetonitrile with 0.1% formic acid. The separation was performed using a gradient eluent (0–35% linear gradient of B in min 0–150, 35–100% of B in min 150–151, and 100% of B in min 151–155) at a flow rate of 300 nL/min. The full mass scan from 350 to 2000 *m/z* was acquired in a positive ion mode with the automatic gain control (AGC) target value of 3 × 10^6^, resolution of 70,000, and maximum injection time (IT) of 120 ms. An HCD MS/MS of the 20 most intense ions in a data-dependent acquisition measurement was performed with a normalized collision energy (NCE) of 32, AGC target of 1 × 10^6^, resolution of 17,500, and maximum IT of 80 ms. Glycoproteomics was performed by injecting 10 μg of enriched glycopeptides into the LC/MS/MS system. A full MS scan was performed at a scan range of 350–2000 *m/z* with a resolution of 70,000, AGC target value of 3 × 10^6^, and maximum IT of 200 ms. The data-dependent acquisition of the top 20 ions was conducted with the NCE of 35, resolution of 17,500, AGC target value of 1 × 10^6^, and maximum IT of 350 ms.

### Data analysis

A database search was performed using the Proteome Discoverer 2.4 with Sequest HT (Thermo Fisher Scientific) against the UniProt human database (July 2020). Peptide sequencing was performed on the fully trypsin-digested proteins with a maximum of two missed cleavages using a 5-ppm mass tolerance for precursor ions and 0.02 Da of fragment ion tolerance. The carbamidomethylation of Cys was used for static modification. For the deglycosylated peptide analysis, asparagine deamidation was added. Dynamic modifications of proteins included the oxidation of methionine, the acetylation, methionine-loss, and methionine-loss + acetylation of N-Terminus. Label-free relative quantitation comparing iPSC proteins was performed using the Proteome Discoverer 2.4.

The qualitative and quantitative analysis of enriched glycopeptides was conducted using the Byonic software version 2.10 (Protein Metrics Inc., Cupertino, CA, USA) and Proteome Discoverer 2.4. The databases used were the UniProt human database (July 2017) and the glycan database containing 309 mammalian N-glycans. Glycopeptide sequencing was performed with a configuration involving trypsin digestion with a maximum of two missed cleavages, with a 5-ppm mass tolerance for precursor ions, and 0.01 Da of product ion mass tolerance. Static modifications included the carbamidomethylation of Cys and modification of GlcNAc with Asn. The results were evaluated manually, and peptides lacking N-glycosylation consensus sequence were excluded. Finally, the GO enrichment analysis and KEGG PATHWAY analysis were performed with DAVID^61^ ver.6.8.

### Statistical analysis

All calculations were performed using Proteome Discoverer (ver.2.4). Data are expressed as mean ± standard error. Gene ontology and KEGG pathway analysis were performed using DAVID (ver.6.8, https://david.ncifcrf.gov/). Heatmap (Fig. 3a) was created using Proteome Discoverer (ver.2.4). Gene ontology enrichment graphs (Fig. 3c) were created using numpy (ver.1.19.2), pandas (ver.1.1.2) and matplotlib (ver.3.3.2). Graphs (Figs. 4b and 5) were made using Microsoft Excel (ver.16.013127.21210). Proteomic and glycoproteomic data (Supplementary Tables S1) were analyzed by a two-tailed Student’s t test, and the resulting *p*-values were adjusted using the Benjamini–Hochberg method for controlling the discovery rate (FDR) using Proteome Discoverer (ver.2.4). Normalization was performed using the total peptide amount to account for random errors. Normalization mode was set to total peptide amount (Figs. 3b,c, and 5b, and Supplementary Figure S1). All spots with *p*-value smaller than 0.05 were considered as differentially expressed (two-tailed).

## Supplementary Information

Supplementary Information.
