# Supplementary material for: Glycoproteomic analysis of the changes in protein N-glycosylation during neuronal differentiation in human-induced pluripotent stem cells and derived neuronal cells
Source: Sci Rep. 2021 May 27;11:11169. doi: 10.1038/s41598-021-90102-z (PMC8160270; doi:10.1038/s41598-021-90102-z)
Supplement: Supplementary file 1 — Supplementary Information. [file 41598_2021_90102_MOESM1_ESM.pdf]

Glycoproteomic analysis of the changes in protein N-glycosylation during neuronal differentiation in human-induced pluripotent stem cells and derived neuronal cells

Kazumasa Kimura, Takumi Koizumi, Takaya Urasawa, Yuki Ohta, Daisuke Takakura, Nana Kawasaki*

Biopharmaceutical and Regenerative Sciences, Graduate School of Medical Life Science, Yokohama City University

*To whom correspondence should be addressed.

Supplementary information

- Supplementary Table S1
- Supplementary Table S2
- Supplementary Table S3
- Supplementary Table S4
- Supplementary Figure S1
- Supplementary Figure S2

Supplementary Table S1. (a) Proteins with increased levels in the NSCs (>2 fold)

Accession	Protein Name	Abundance Ratio: (NSC) / (iPSC)	Abundance Ratio p-Value: (NSC) / (iPSC)
Q7Z6L0	Proline-rich transmembrane protein 2	100	1.00.E-17
Q7Z6L0-2	Isoform 2 of Proline-rich transmembrane protein 2	100	1.00.E-17
Q7Z6L0-3	Isoform 3 of Proline-rich transmembrane protein 2	100	1.00.E-17
P26378	ELAV-like protein 4	100	1.00.E-17
P26378-1	Isoform 1 of ELAV-like protein 4	100	1.00.E-17
P26378-3	Isoform 3 of ELAV-like protein 4	100	1.00.E-17
P26378-5	Isoform 5 of ELAV-like protein 4	100	1.00.E-17
P26378-4	Isoform 4 of ELAV-like protein 4	100	1.00.E-17
P26378-2	Isoform 2 of ELAV-like protein 4	100	1.00.E-17
Q8IUH4	Palmitoyltransferase ZDHHC13	100	1.00.E-17
Q9H902-3	Isoform 3 of Receptor expression-enhancing protein 1	100	1.00.E-17
Q9H902	Receptor expression-enhancing protein 1	100	1.00.E-17
Q9H902-2	Isoform 2 of Receptor expression-enhancing protein 1	100	1.00.E-17
Q9GZT4	Serine racemase	100	1.00.E-17
O15217-2	Isoform 2 of Glutathione S-transferase A4	100	1.00.E-17
O94772-2	Isoform 2 of Lymphocyte antigen 6H	100	1.00.E-17
O94772	Lymphocyte antigen 6H	100	1.00.E-17
O75051-2	Isoform 2 of Plexin-A2	100	1.00.E-17
Q9HAR2-2	Isoform 2 of Adhesion G protein-coupled receptor L3	100	1.00.E-17
Q9HAR2-4	Isoform 4 of Adhesion G protein-coupled receptor L3	100	1.00.E-17
Q9HAR2	Adhesion G protein-coupled receptor L3	100	1.00.E-17
O60641-2	Isoform 2 of Clathrin coat assembly protein AP180	100	1.00.E-17
O43602	Neuronal migration protein doublecortin	100	1.00.E-17
Q9HAP6-2	Isoform 2 of Protein lin-7 homolog B	100	1.00.E-17
Q9ULB1	Neurexin-1	100	1.00.E-17
Q07866-8	Isoform S of Kinesin light chain 1	100	1.00.E-17
Q8N4Q0	Prostaglandin reductase 3	100	1.00.E-17
Q96SB3	Neurabin-2	100	1.00.E-17
Q9BRK4	Leucine zipper putative tumor suppressor 2	100	1.00.E-17
Q9HAR2-3	Isoform 3 of Adhesion G protein-coupled receptor L3	100	1.00.E-17
P32418-2	Isoform 3 of Sodium/calcium exchanger 1	100	1.00.E-17
P32418-3	Isoform 7 of Sodium/calcium exchanger 1	100	1.00.E-17
P32418-4	Isoform 10 of Sodium/calcium exchanger 1	100	1.00.E-17
P32418	Sodium/calcium exchanger 1	100	1.00.E-17
P32418-5	Isoform 5 of Sodium/calcium exchanger 1	100	1.00.E-17
P52895	Aldo-keto reductase family 1 member C2	100	1.00.E-17
Q9ULB1-4	Isoform 4 of Neurexin-1	100	1.00.E-17
P58400-1	Isoform 3b of Neurexin-1-beta	100	1.00.E-17
Q5GH72	XK-related protein 7	100	1.00.E-17
P29320	Ephrin type-A receptor 3	100	1.00.E-17
P56159	GDNF family receptor alpha-1	100	1.00.E-17
P56159-2	Isoform 2 of GDNF family receptor alpha-1	100	1.00.E-17
Q5T1Q4-2	Isoform 2 of Solute carrier family 35 member F1	100	1.00.E-17
Q5T1Q4	Solute carrier family 35 member F1	100	1.00.E-17
Q9UM19	Hippocalcin-like protein 4	100	1.00.E-17
Q9Y6I9	Testis-expressed protein 264	100	1.00.E-17
Q7Z6K5	Arpin	100	1.00.E-17
Q15173	Serine/threonine-protein phosphatase 2A 56 kDa regulatory subunit beta isoform	100	1.00.E-17
Q15173-2	Isoform Beta-2 of Serine/threonine-protein phosphatase 2A 56 kDa regulatory subunit beta isoform	100	1.00.E-17
O95670-3	Isoform 3 of V-type proton ATPase subunit G 2	100	1.00.E-17
Q9Y653-5	Isoform 5 of Adhesion G-protein coupled receptor G1	100	1.00.E-17
Q9Y653-2	Isoform 2 of Adhesion G-protein coupled receptor G1	100	1.00.E-17
Q9Y653	Adhesion G-protein coupled receptor G1	100	1.00.E-17
Q9Y653-3	Isoform 3 of Adhesion G-protein coupled receptor G1	100	1.00.E-17
A1A5C7	Solute carrier family 22 member 23	100	1.00.E-17
Q8WXA3-4	Isoform 3 of RUN and FYVE domain-containing protein 2	100	1.00.E-17
P54803	Galactocerebrosidase	100	1.00.E-17
Q8WXA3-5	Isoform 4 of RUN and FYVE domain-containing protein 2	100	1.00.E-17
O43426-5	Isoform 4 of Synaptojanin-1	100	1.00.E-17
P29320-2	Isoform 2 of Ephrin type-A receptor 3	100	1.00.E-17
P49441	Inositol polyphosphate 1-phosphatase	100	1.00.E-17

O95297-3	Isoform 3 of Myelin protein zero-like protein 1	100	1.00.E-17
Q8NFZ4	Neuroligin-2	100	1.00.E-17
O75093	Slit homolog 1 protein	100	1.00.E-17
Q9NWQ8	Phosphoprotein associated with glycosphingolipid-enriched microdomains 1	100	1.00.E-17
Q9UPN3	Microtubule-actin cross-linking factor 1, isoforms 1/2/3/5	100	1.00.E-17
Q9UPN3-4	Isoform 5 of Microtubule-actin cross-linking factor 1, isoforms 1/2/3/5	100	1.00.E-17
Q96B86-3	Isoform 2 of Repulsive guidance molecule A	100	1.00.E-17
Q96B86	Repulsive guidance molecule A	100	1.00.E-17
Q96B86-4	Isoform 3 of Repulsive guidance molecule A	100	1.00.E-17
P55273	Cyclin-dependent kinase 4 inhibitor D	100	1.00.E-17
Q8NFT8	Delta and Notch-like epidermal growth factor-related receptor	100	1.00.E-17
Q13114	TNF receptor-associated factor 3	100	1.00.E-17
C9JRZ8	Aldo-keto reductase family 1 member B15	100	1.00.E-17
C9JRZ8-2	Isoform 2 of Aldo-keto reductase family 1 member B15	100	1.00.E-17
P05067-3	Isoform L-APP677 of Amyloid-beta precursor protein	100	1.00.E-17
P05067-10	Isoform APP639 of Amyloid-beta precursor protein	100	1.00.E-17
P05067-4	Isoform APP695 of Amyloid-beta precursor protein	100	1.00.E-17
P43007	Neutral amino acid transporter A	100	1.00.E-17
P43007-2	Isoform 2 of Neutral amino acid transporter A	100	1.00.E-17
Q9H4G0-2	Isoform 2 of Band 4.1-like protein 1	100	1.00.E-17
Q99767-2	Isoform 2 of Amyloid-beta A4 precursor protein-binding family A member 2	100	1.00.E-17
Q99767	Amyloid-beta A4 precursor protein-binding family A member 2	100	1.00.E-17
A1A5C7-4	Isoform 4 of Solute carrier family 22 member 23	100	1.00.E-17
P23435	Cerebellin-1	100	1.00.E-17
O95479	GDH/6PGL endoplasmic bifunctional protein	100	1.00.E-17
O95479-2	Isoform 2 of GDH/6PGL endoplasmic bifunctional protein	100	1.00.E-17
Q9Y653-4	Isoform 4 of Adhesion G-protein coupled receptor G1	100	1.00.E-17
Q15111	Inactive phospholipase C-like protein 1	100	1.00.E-17
P81274	G-protein-signaling modulator 2	100	1.00.E-17
Q14982-4	Isoform 4 of Opioid-binding protein/cell adhesion molecule	100	1.00.E-17
Q14982-2	Isoform 2 of Opioid-binding protein/cell adhesion molecule	100	1.00.E-17
Q14982-3	Isoform 3 of Opioid-binding protein/cell adhesion molecule	100	1.00.E-17
Q14982	Opioid-binding protein/cell adhesion molecule	100	1.00.E-17
Q99946	Proline-rich transmembrane protein 1	100	1.00.E-17
Q02153	Guanylate cyclase soluble subunit beta-1	100	1.00.E-17
Q02153-3	Isoform 3 of Guanylate cyclase soluble subunit beta-1	100	1.00.E-17
Q02153-2	Isoform HSGC-2 of Guanylate cyclase soluble subunit beta-1	100	1.00.E-17
P61601	Neurocalcin-delta	100	1.00.E-17
Q96EQ0	Small glutamine-rich tetratricopeptide repeat-containing protein beta	100	1.00.E-17
Q9HCD6-3	Isoform 3 of Protein TANC2	100	1.00.E-17
Q9HCD6-4	Isoform 4 of Protein TANC2	100	1.00.E-17
Q9Y3E7-2	Isoform 2 of Charged multivesicular body protein 3	100	1.00.E-17
Q9Y3E7	Charged multivesicular body protein 3	100	1.00.E-17
Q9Y3E7-4	Isoform 4 of Charged multivesicular body protein 3	100	1.00.E-17
Q9Y3E7-3	Isoform 3 of Charged multivesicular body protein 3	100	1.00.E-17
O14770	Homeobox protein Meis2	100	1.00.E-17
O14770-8	Isoform 8 of Homeobox protein Meis2	100	1.00.E-17
O00470	Homeobox protein Meis1	100	1.00.E-17
O00470-2	Isoform 2 of Homeobox protein Meis1	100	1.00.E-17
O14770-7	Isoform 7 of Homeobox protein Meis2	100	1.00.E-17
O14770-5	Isoform 5 of Homeobox protein Meis2	100	1.00.E-17
O14770-3	Isoform 3 of Homeobox protein Meis2	100	1.00.E-17
O14770-6	Isoform 6 of Homeobox protein Meis2	100	1.00.E-17
O14770-4	Isoform 4 of Homeobox protein Meis2	100	1.00.E-17
O14770-2	Isoform 2 of Homeobox protein Meis2	100	1.00.E-17
Q13114-2	Isoform 2 of TNF receptor-associated factor 3	100	1.00.E-17
Q9P0B6	Coiled-coil domain-containing protein 167	100	1.00.E-17
Q9UKF7	Cytoplasmic phosphatidylinositol transfer protein 1	100	1.00.E-17
Q9UKF7-2	Isoform 2 of Cytoplasmic phosphatidylinositol transfer protein 1	100	1.00.E-17
P04062-2	Isoform Short of Lysosomal acid glucosylceramidase	100	1.00.E-17
P04062	Lysosomal acid glucosylceramidase	100	1.00.E-17
P04062-5	Isoform 5 of Lysosomal acid glucosylceramidase	100	1.00.E-17
P04062-4	Isoform 4 of Lysosomal acid glucosylceramidase	100	1.00.E-17
P47869	Gamma-aminobutyric acid receptor subunit alpha-2	100	1.00.E-17

P14867	Gamma-aminobutyric acid receptor subunit alpha-1	100	1.00.E-17
P47869-2	Isoform 2 of Gamma-aminobutyric acid receptor subunit alpha-2	100	1.00.E-17
P31644	Gamma-aminobutyric acid receptor subunit alpha-5	100	1.00.E-17
A1A5C7-2	Isoform 2 of Solute carrier family 22 member 23	100	1.00.E-17
A1A5C7-3	Isoform 3 of Solute carrier family 22 member 23	100	1.00.E-17
Q9UPW8	Protein unc-13 homolog A	100	1.00.E-17
O14795-2	Isoform 2 of Protein unc-13 homolog B	100	1.00.E-17
Q8NB66	Protein unc-13 homolog C	100	1.00.E-17
O14795	Protein unc-13 homolog B	100	1.00.E-17
Q8N350-4	Isoform 2 of Voltage-dependent calcium channel beta subunit-associated regulatory protein	100	1.00.E-17
Q8N350	Voltage-dependent calcium channel beta subunit-associated regulatory protein	100	1.00.E-17
Q53HC9	EARP and GARP complex-interacting protein 1	100	1.00.E-17
Q9Y4K3	TNF receptor-associated factor 6	100	1.00.E-17
Q8IYK8	GTP-binding protein REM 2	100	1.00.E-17
P13929-2	Isoform 2 of Beta-enolase	100	1.00.E-17
Q96DE0-4	Isoform 4 of U8 snoRNA-decapping enzyme	100	1.00.E-17
Q8N2G4-3	Isoform 3 of Ly6/PLAUR domain-containing protein 1	100	1.00.E-17
Q8N2G4	Ly6/PLAUR domain-containing protein 1	100	1.00.E-17
Q8N2G4-4	Isoform 4 of Ly6/PLAUR domain-containing protein 1	100	1.00.E-17
Q8N2G4-2	Isoform 2 of Ly6/PLAUR domain-containing protein 1	100	1.00.E-17
Q9H1C7	Cysteine-rich and transmembrane domain-containing protein 1	100	1.00.E-17
Q15111-2	Isoform 2 of Inactive phospholipase C-like protein 1	100	1.00.E-17
Q8IZR5-2	Isoform 2 of CKLF-like MARVEL transmembrane domain-containing protein 4	100	1.00.E-17
Q8IZR5-3	Isoform 3 of CKLF-like MARVEL transmembrane domain-containing protein 4	100	1.00.E-17
Q8IZR5	CKLF-like MARVEL transmembrane domain-containing protein 4	100	1.00.E-17
Q8N5H7-2	Isoform 2 of SH2 domain-containing protein 3C	100	1.00.E-17
Q8N5H7-3	Isoform 3 of SH2 domain-containing protein 3C	100	1.00.E-17
Q8N5H7	SH2 domain-containing protein 3C	100	1.00.E-17
Q8N5H7-5	Isoform 5 of SH2 domain-containing protein 3C	100	1.00.E-17
Q8N5H7-6	Isoform 6 of SH2 domain-containing protein 3C	100	1.00.E-17
Q8N5H7-4	Isoform 4 of SH2 domain-containing protein 3C	100	1.00.E-17
Q96Q04	Serine/threonine-protein kinase LMTK3	100	1.00.E-17
O95630	STAM-binding protein	100	1.00.E-17
Q9BYT3	Serine/threonine-protein kinase 33	100	1.00.E-17
Q96S06	Lipase maturation factor 1	100	1.00.E-17
P07101-4	Isoform 4 of Tyrosine 3-monooxygenase	100	1.00.E-17
P07101	Tyrosine 3-monooxygenase	100	1.00.E-17
P07101-5	Isoform 5 of Tyrosine 3-monooxygenase	100	1.00.E-17
P07101-6	Isoform 6 of Tyrosine 3-monooxygenase	100	1.00.E-17
P07101-3	Isoform 2 of Tyrosine 3-monooxygenase	100	1.00.E-17
P07101-2	Isoform 1 of Tyrosine 3-monooxygenase	100	1.00.E-17
Q04206-4	Isoform 4 of Transcription factor p65	100	1.00.E-17
Q8IWE4	DCN1-like protein 3	100	1.00.E-17
Q969R2-5	Isoform 5 of Oxysterol-binding protein 2	100	1.00.E-17
Q969R2-4	Isoform 4 of Oxysterol-binding protein 2	100	1.00.E-17
O00255-2	Isoform 2 of Menin	100	1.00.E-17
O00255-3	Isoform 3 of Menin	100	1.00.E-17
O00255	Menin	100	1.00.E-17
Q9UPU3	VPS10 domain-containing receptor SorCS3	100	1.00.E-17
Q9ULP0-4	Isoform 4 of Protein NDRG4	100	1.00.E-17
Q9ULP0	Protein NDRG4	100	1.00.E-17
Q9ULP0-2	Isoform 2 of Protein NDRG4	100	1.00.E-17
Q9ULP0-5	Isoform 5 of Protein NDRG4	100	1.00.E-17
Q96HH4	Transmembrane protein 169	100	1.00.E-17
P33897	ATP-binding cassette sub-family D member 1	100	1.00.E-17
Q8N2Q7	Neuroligin-1	100	1.00.E-17
Q8N2Q7-2	Isoform 2 of Neuroligin-1	100	1.00.E-17
Q86TC9-2	Isoform 2 of Myopalladin	100	1.00.E-17
Q86TC9	Myopalladin	100	1.00.E-17
Q9HCE9-2	Isoform 2 of Anoctamin-8	100	1.00.E-17
Q9HCE9	Anoctamin-8	100	1.00.E-17
Q8NHH9-4	Isoform 4 of Atlastin-2	100	1.00.E-17
Q5T4F4	Protrudin	100	1.00.E-17
Q5T4F4-8	Isoform 8 of Protrudin	100	1.00.E-17

Q5T4F4-6	Isoform 6 of Protrudin	100	1.00.E-17
Q5T4F4-2	Isoform 2 of Protrudin	100	1.00.E-17
Q5T4F4-5	Isoform 5 of Protrudin	100	1.00.E-17
Q5T4F4-7	Isoform 7 of Protrudin	100	1.00.E-17
Q5T4F4-3	Isoform 3 of Protrudin	100	1.00.E-17
Q8IVP5	FUN14 domain-containing protein 1	100	1.00.E-17
Q8WXA3-1	Isoform 5 of RUN and FYVE domain-containing protein 2	100	1.00.E-17
Q8WXA3	RUN and FYVE domain-containing protein 2	100	1.00.E-17
Q8WXA3-3	Isoform 2 of RUN and FYVE domain-containing protein 2	100	1.00.E-17
P39210	Protein Mpv17	100	1.00.E-17
O94813-3	Isoform 3 of Slit homolog 2 protein	100	1.00.E-17
O94813	Slit homolog 2 protein	100	1.00.E-17
O94813-2	Isoform 2 of Slit homolog 2 protein	100	1.00.E-17
P58511	Small integral membrane protein 11A	100	1.00.E-17
Q14511-3	Isoform 3 of Enhancer of filamentation 1	100	1.00.E-17
Q14511	Enhancer of filamentation 1	100	1.00.E-17
Q9GZY8	Mitochondrial fission factor	100	1.00.E-17
Q9GZY8-5	Isoform 5 of Mitochondrial fission factor	100	1.00.E-17
Q9GZY8-2	Isoform 2 of Mitochondrial fission factor	100	1.00.E-17
Q9Y6Q1	Calpain-6	100	1.00.E-17
Q5JTW2-3	Isoform 3 of Centrosomal protein of 78 kDa	100	1.00.E-17
Q8WX77	Insulin-like growth factor-binding protein-like 1	100	1.00.E-17
Q8N8E3	Centrosomal protein of 112 kDa	100	1.00.E-17
Q14693-4	Isoform 4 of Phosphatidate phosphatase LPIN1	100	1.00.E-17
P0CG40	Transcription factor Sp9	100	1.00.E-17
Q9H1J7	Protein Wnt-5b	100	1.00.E-17
Q9H313-5	Isoform 5 of Protein tweety homolog 1	100	1.00.E-17
Q9H313-2	Isoform 2 of Protein tweety homolog 1	100	1.00.E-17
Q9Y6N8	Cadherin-10	100	1.00.E-17
Q12981-1	Isoform 3 of Vesicle transport protein SEC20	100	1.00.E-17
Q9H840	Gem-associated protein 7	100	1.00.E-17
P34896-2	Isoform 2 of Serine hydroxymethyltransferase, cytosolic	100	1.00.E-17
O76038	Secretagogin	100	1.00.E-17
Q86W92	Liprin-beta-1	100	1.00.E-17
Q96GX1	Tectonic-2	100	1.00.E-17
Q9UMR5-3	Isoform 3 of Lysosomal thioesterase PPT2	100	1.00.E-17
Q9H7X7-2	Isoform 2 of Intraflagellar transport protein 22 homolog	100	1.00.E-17
Q14693-7	Isoform 7 of Phosphatidate phosphatase LPIN1	100	1.00.E-17
Q12981	Vesicle transport protein SEC20	100	1.00.E-17
Q86W92-4	Isoform 4 of Liprin-beta-1	100	1.00.E-17
P34896-3	Isoform 3 of Serine hydroxymethyltransferase, cytosolic	100	1.00.E-17
Q68CQ7-2	Isoform 2 of Glycosyltransferase 8 domain-containing protein 1	100	1.00.E-17
Q5JTW2-5	Isoform 4 of Centrosomal protein of 78 kDa	100	1.00.E-17
Q12981-2	Isoform 2 of Vesicle transport protein SEC20	100	1.00.E-17
P34896-4	Isoform 4 of Serine hydroxymethyltransferase, cytosolic	100	1.00.E-17
Q12981-3	Isoform 4 of Vesicle transport protein SEC20	100	1.00.E-17
Q5JTW2-2	Isoform 2 of Centrosomal protein of 78 kDa	100	1.00.E-17
P10071	Transcriptional activator GLI3	100	1.00.E-17
Q5JTW2	Centrosomal protein of 78 kDa	100	1.00.E-17
Q68CQ7	Glycosyltransferase 8 domain-containing protein 1	100	1.00.E-17
Q13474	Dystrophin-related protein 2	100	1.00.E-17
P47928	DNA-binding protein inhibitor ID-4	100	1.00.E-17
Q9H313-3	Isoform 3 of Protein tweety homolog 1	100	1.00.E-17
Q9NWB7	Intraflagellar transport protein 57 homolog	100	1.00.E-17
P21980-3	Isoform 3 of Protein-glutamine gamma-glutamyltransferase 2	100	1.00.E-17
Q13474-2	Isoform 2 of Dystrophin-related protein 2	100	1.00.E-17
Q8IXZ3-1	Isoform 1 of Transcription factor Sp8	100	1.00.E-17
Q8IXZ3	Transcription factor Sp8	100	1.00.E-17
P50135	Histamine N-methyltransferase	100	1.00.E-17
Q96GX1-2	Isoform 2 of Tectonic-2	100	1.00.E-17
Q14693-5	Isoform 5 of Phosphatidate phosphatase LPIN1	100	1.00.E-17
P41221-2	Isoform 2 of Protein Wnt-5a	100	1.00.E-17
Q86W92-2	Isoform 2 of Liprin-beta-1	100	1.00.E-17
Q9UMR5	Lysosomal thioesterase PPT2	100	1.00.E-17

Q9HCM2	Plexin-A4	100	1.00.E-17
Q7RTP0	Magnesium transporter NIPA1	100	1.00.E-17
Q86W92-3	Isoform 3 of Liprin-beta-1	100	1.00.E-17
Q14693-3	Isoform 3 of Phosphatidate phosphatase LPIN1	100	1.00.E-17
Q9ULB4	Cadherin-9	100	1.00.E-17
Q8IXZ3-4	Isoform 4 of Transcription factor Sp8	100	1.00.E-17
Q8IWY4	Signal peptide, CUB and EGF-like domain-containing protein 1	100	1.00.E-17
Q14693	Phosphatidate phosphatase LPIN1	100	1.00.E-17
Q9H313	Protein tweety homolog 1	100	1.00.E-17
Q8WYQ5-3	Isoform 3 of Microprocessor complex subunit DGCR8	100	1.00.E-17
P34896	Serine hydroxymethyltransferase, cytosolic	100	1.00.E-17
Q9H7X7	Intraflagellar transport protein 22 homolog	100	1.00.E-17
Q8WYQ5	Microprocessor complex subunit DGCR8	100	1.00.E-17
P41221	Protein Wnt-5a	100	1.00.E-17
Q8IXZ3-2	Isoform 2 of Transcription factor Sp8	100	1.00.E-17
Q9UMR5-2	Isoform 2 of Lysosomal thioesterase PPT2	100	1.00.E-17
Q14693-2	Isoform 2 of Phosphatidate phosphatase LPIN1	100	1.00.E-17
P23763	Vesicle-associated membrane protein 1	48.451	2.37.E-10
P23763-2	Isoform 3 of Vesicle-associated membrane protein 1	48.451	2.37.E-10
P23763-3	Isoform 2 of Vesicle-associated membrane protein 1	48.451	2.37.E-10
Q9Y2B0-2	Isoform 2 of Protein canopy homolog 2	46.375	2.71.E-08
P98172	Ephrin-B1	31.181	4.44.E-15
O15540	Fatty acid-binding protein, brain	29.204	4.24.E-14
O15540-2	Isoform 2 of Fatty acid-binding protein, brain	29.204	4.24.E-14
Q06187-2	Isoform BTK-C of Tyrosine-protein kinase BTK	28.762	1.74.E-08
Q06187	Tyrosine-protein kinase BTK	28.762	1.74.E-08
Q8TBF2-3	Isoform 3 of Prostamide/prostaglandin F synthase	28.425	3.96.E-07
Q8TBF2-7	Isoform 6 of Prostamide/prostaglandin F synthase	28.425	3.96.E-07
Q8TBF2-6	Isoform 5 of Prostamide/prostaglandin F synthase	28.425	3.96.E-07
Q8TBF2	Prostamide/prostaglandin F synthase	28.425	3.96.E-07
Q8TBF2-4	Isoform 4 of Prostamide/prostaglandin F synthase	28.425	3.96.E-07
Q8TBF2-2	Isoform 2 of Prostamide/prostaglandin F synthase	28.425	3.96.E-07
P35080	Profilin-2	24.022	4.74.E-08
P05937	Calbindin	23.489	1.47.E-12
Q96SJ8	Tetraspanin-18	22.658	1.60.E-07
P05937-2	Isoform 2 of Calbindin	22.456	2.98.E-12
P27701-2	Isoform 2 of CD82 antigen	22.014	1.33.E-10
P27701	CD82 antigen	22.014	1.33.E-10
P40939-2	Isoform 2 of Trifunctional enzyme subunit alpha, mitochondrial	20.72	4.08.E-08
O15217	Glutathione S-transferase A4	18.475	2.65.E-06
P22676	Calretinin	16.849	6.39.E-08
O75592	E3 ubiquitin-protein ligase MYCBP2	16.562	1.25.E-06
O75592-2	Isoform 2 of E3 ubiquitin-protein ligase MYCBP2	16.562	1.25.E-06
Q9Y625	Glypican-6	15.635	4.42.E-07
P21266	Glutathione S-transferase Mu 3	15.437	3.46.E-10
O43602-2	Isoform 2 of Neuronal migration protein doublecortin	14.122	4.53.E-09
P14618-2	Isoform M1 of Pyruvate kinase PKM	13.977	2.98.E-08
Q7L775	EPM2A-interacting protein 1	13.058	3.53.E-07
Q16352	Alpha-internexin	11.591	2.34.E-07
Q6P4I2	WD repeat-containing protein 73	11.462	1.38.E-04
P17677-2	Isoform 2 of Neuromodulin	11.407	6.00.E-08
P17677	Neuromodulin	11.407	6.00.E-08
P80370-2	Isoform Short of Protein delta homolog 1	11.015	2.73.E-05
Q9UBI1	COMM domain-containing protein 3	10.94	5.10.E-05
A6NKG5	Retrotransposon-like protein 1	10.865	1.02.E-03
P80370	Protein delta homolog 1	10.775	3.43.E-06
P09455	Retinol-binding protein 1	10.159	2.34.E-06
Q9NTI5-5	Isoform 5 of Sister chromatid cohesion protein PDS5 homolog B	9.959	2.83.E-04
Q8NFJ9-3	Isoform 2 of Bardet-Biedl syndrome 1 protein	9.475	4.65.E-03
Q8NFJ9	Bardet-Biedl syndrome 1 protein	9.475	4.65.E-03
Q8NFJ9-2	Isoform 3 of Bardet-Biedl syndrome 1 protein	9.475	4.65.E-03
Q8NFM7-3	Isoform 3 of Interleukin-17 receptor D	9.356	2.11.E-03
Q8NFM7-4	Isoform 4 of Interleukin-17 receptor D	9.356	2.11.E-03
Q8NFM7	Interleukin-17 receptor D	9.356	2.11.E-03

Q8NFM7-2	Isoform 2 of Interleukin-17 receptor D	9.356	2.11.E-03
P52943	Cysteine-rich protein 2	9.353	1.76.E-05
P52943-2	Isoform 2 of Cysteine-rich protein 2	9.353	4.99.E-05
Q9NU23	LYR motif-containing protein 2	9.316	7.96.E-04
Q9BXC9	Bardet-Biedl syndrome 2 protein	9.302	2.27.E-03
P22612	cAMP-dependent protein kinase catalytic subunit gamma	9.24	1.92.E-04
Q8IXV7	Kelch domain-containing protein 8B	9.22	2.37.E-03
O14786-3	Isoform 3 of Neuropilin-1	9.079	1.58.E-04
O14786	Neuropilin-1	9.079	1.58.E-04
O14786-2	Isoform 2 of Neuropilin-1	9.079	1.58.E-04
Q0VDG4-2	Isoform 2 of Secernin-3	9.021	2.59.E-03
Q0VDG4	Secernin-3	9.021	2.59.E-03
P11137-2	Isoform 2 of Microtubule-associated protein 2	9.008	7.40.E-07
P12036	Neurofilament heavy polypeptide	8.785	8.33.E-07
P12036-2	Isoform 2 of Neurofilament heavy polypeptide	8.785	8.33.E-07
Q5SQI0-5	Isoform 5 of Alpha-tubulin N-acetyltransferase 1	8.672	5.03.E-04
Q5SQI0-4	Isoform 4 of Alpha-tubulin N-acetyltransferase 1	8.672	5.03.E-04
Q5SQI0	Alpha-tubulin N-acetyltransferase 1	8.672	5.03.E-04
Q5SQI0-2	Isoform 2 of Alpha-tubulin N-acetyltransferase 1	8.672	5.03.E-04
Q5SQI0-3	Isoform 3 of Alpha-tubulin N-acetyltransferase 1	8.672	5.27.E-04
Q5SQI0-7	Isoform 7 of Alpha-tubulin N-acetyltransferase 1	8.672	5.27.E-04
Q5SQI0-6	Isoform 6 of Alpha-tubulin N-acetyltransferase 1	8.672	5.27.E-04
Q96KR6	Protein FAM210B, mitochondrial	8.607	9.69.E-04
Q9UL42	Paraneoplastic antigen Ma2	8.579	3.56.E-03
P0C7U0	Protein ELFN1	8.532	9.23.E-04
Q5R3F8	Protein phosphatase 1 regulatory subunit 29	8.532	9.23.E-04
Q9BPU6	Dihydropyrimidinase-related protein 5	8.43	1.32.E-06
Q96K83	Zinc finger protein 521	8.399	1.97.E-04
Q9UI15	Transgelin-3	8.281	4.26.E-05
Q9NZ53	Podocalyxin-like protein 2	8.251	1.50.E-03
P06396-4	Isoform 4 of Gelsolin	8.214	2.54.E-06
P06396	Gelsolin	8.214	2.54.E-06
P06396-2	Isoform 2 of Gelsolin	8.214	3.02.E-06
P06396-3	Isoform 3 of Gelsolin	8.214	2.54.E-06
Q01081-2	Isoform 2 of Splicing factor U2AF 35 kDa subunit	8.21	3.57.E-03
Q8WXH0-8	Isoform 8 of Nesprin-2	8.132	3.25.E-05
Q8WXH0-9	Isoform 9 of Nesprin-2	8.132	3.25.E-05
Q9NRX4-2	Isoform 2 of 14 kDa phosphohistidine phosphatase	8.098	2.46.E-04
Q9NRX4	14 kDa phosphohistidine phosphatase	8.098	2.46.E-04
Q9NSA3	Beta-catenin-interacting protein 1	8.052	3.45.E-04
P09455-2	Isoform 2 of Retinol-binding protein 1	8.027	2.58.E-05
P09455-3	Isoform 3 of Retinol-binding protein 1	8.027	2.58.E-05
Q8N6N7	Acyl-CoA-binding domain-containing protein 7	7.916	7.43.E-04
P84074	Neuron-specific calcium-binding protein hippocalcin	7.902	5.37.E-04
Q9Y281	Cofilin-2	7.855	8.23.E-05
Q9Y281-3	Isoform 3 of Cofilin-2	7.855	8.23.E-05
O60826	Coiled-coil domain-containing protein 22	7.838	1.84.E-04
Q9UQ03-2	Isoform 2 of Coronin-2B	7.809	3.91.E-04
Q9UQ03	Coronin-2B	7.809	3.91.E-04
P29317	Ephrin type-A receptor 2	7.705	2.42.E-05
Q8TC26-2	Isoform 2 of Transmembrane protein 163	7.676	8.59.E-03
P08670	Vimentin	7.585	4.10.E-06
Q8TCZ2-2	Isoform 2 of CD99 antigen-like protein 2	7.527	9.37.E-04
Q8TCZ2	CD99 antigen-like protein 2	7.527	9.37.E-04
Q8TCZ2-5	Isoform 5 of CD99 antigen-like protein 2	7.527	9.37.E-04
Q8TCZ2-3	Isoform 3 of CD99 antigen-like protein 2	7.527	9.37.E-04
O60831	PRA1 family protein 2	7.524	1.09.E-04
Q5VVQ6	Ubiquitin thioesterase OTU1	7.505	1.90.E-03
Q5VVQ6-2	Isoform 2 of Ubiquitin thioesterase OTU1	7.505	1.90.E-03
O14531	Dihydropyrimidinase-related protein 4	7.488	5.35.E-06
Q9Y548	Protein YIPF1	7.465	2.16.E-03
Q5T4D3-4	Isoform 4 of Protein O-mannosyl-transferase TMTC4	7.387	2.75.E-03
Q5T4D3	Protein O-mannosyl-transferase TMTC4	7.387	2.75.E-03
Q5T4D3-2	Isoform 2 of Protein O-mannosyl-transferase TMTC4	7.387	2.75.E-03

Q5T4D3-3	Isoform 3 of Protein O-mannosyl-transferase TMTC4	7.387	2.75.E-03
P42575-3	Isoform 3 of Caspase-2	7.18	8.18.E-03
Q5SSJ5-5	Isoform 4 of Heterochromatin protein 1-binding protein 3	7.101	6.94.E-04
Q9UG01-2	Isoform 2 of Intraflagellar transport protein 172 homolog	7.016	6.69.E-03
Q9UG01	Intraflagellar transport protein 172 homolog	7.016	6.69.E-03
Q8TC26	Transmembrane protein 163	6.961	7.57.E-03
Q5IJ48-2	Isoform 2 of Protein crumbs homolog 2	6.942	1.24.E-04
P80404	4-aminobutyrate aminotransferase, mitochondrial	6.896	8.14.E-05
Q6SZW1	NAD(+) hydrolase SARM1	6.854	2.19.E-04
Q96FC7-2	Isoform 2 of Phytanoyl-CoA hydroxylase-interacting protein-like	6.808	5.49.E-03
P53801	Pituitary tumor-transforming gene 1 protein-interacting protein	6.787	8.72.E-03
P19022	Cadherin-2	6.723	1.41.E-05
P19022-2	Isoform 2 of Cadherin-2	6.723	1.41.E-05
P56557	Transmembrane protein 50B	6.698	1.05.E-02
P35080-2	Isoform IIb of Profilin-2	6.643	1.15.E-05
P59768	Guanine nucleotide-binding protein G(I)/G(S)/G(O) subunit gamma-2	6.604	6.62.E-04
Q14699	Raftlin	6.591	4.55.E-03
P40123-3	Isoform 3 of Adenylyl cyclase-associated protein 2	6.471	1.23.E-04
Q9BV23	Monoacylglycerol lipase ABHD6	6.449	6.87.E-03
P13591-1	Isoform 2 of Neural cell adhesion molecule 1	6.438	4.53.E-05
P13591	Neural cell adhesion molecule 1	6.42	5.13.E-05
P07197-2	Isoform 2 of Neurofilament medium polypeptide	6.384	2.34.E-05
P14415	Sodium/potassium-transporting ATPase subunit beta-2	6.36	3.21.E-03
Q9UPT6	C-Jun-amino-terminal kinase-interacting protein 3	6.315	3.63.E-04
Q8NEU8-3	Isoform 3 of DCC-interacting protein 13-beta	6.311	6.63.E-04
Q16566	Calcium/calmodulin-dependent protein kinase type IV	6.278	1.71.E-03
O43347	RNA-binding protein Musashi homolog 1	6.249	2.88.E-05
Q8NEU8-2	Isoform 2 of DCC-interacting protein 13-beta	6.234	8.15.E-04
P17655	Calpain-2 catalytic subunit	6.206	5.23.E-05
P40123-2	Isoform 2 of Adenylyl cyclase-associated protein 2	6.193	1.25.E-04
Q8NEU8	DCC-interacting protein 13-beta	6.176	8.81.E-04
Q86X83	COMM domain-containing protein 2	6.174	2.40.E-03
Q86X83-2	Isoform 2 of COMM domain-containing protein 2	6.174	2.40.E-03
Q92604	Acyl-CoA:lysophosphatidylglycerol acyltransferase 1	6.172	1.25.E-02
P0CG35	Thymosin beta-15B	6.135	2.38.E-04
P34913-2	Isoform 2 of Bifunctional epoxide hydrolase 2	6.119	1.11.E-03
P34913-3	Isoform 3 of Bifunctional epoxide hydrolase 2	6.119	1.11.E-03
P34913	Bifunctional epoxide hydrolase 2	6.119	1.11.E-03
Q99766	ATP synthase subunit s, mitochondrial	6.112	1.34.E-02
P17655-2	Isoform 2 of Calpain-2 catalytic subunit	6.099	1.14.E-04
P51649-2	Isoform 2 of Succinate-semialdehyde dehydrogenase, mitochondrial	6.013	4.47.E-04
P51649	Succinate-semialdehyde dehydrogenase, mitochondrial	6.013	4.47.E-04
P40123	Adenylyl cyclase-associated protein 2	5.975	6.75.E-05
O94830	Phospholipase DDHD2	5.969	4.73.E-03
O94830-2	Isoform 2 of Phospholipase DDHD2	5.969	4.73.E-03
P00918	Carbonic anhydrase 2	5.966	7.21.E-05
Q13449	Limbic system-associated membrane protein	5.939	2.04.E-03
Q5IJ48	Protein crumbs homolog 2	5.936	2.21.E-04
Q7L099	Protein RUFY3	5.894	7.22.E-05
Q92997-2	Isoform 2 of Segment polarity protein dishevelled homolog DVL-3	5.836	1.51.E-02
Q92997	Segment polarity protein dishevelled homolog DVL-3	5.836	1.51.E-02
O15439-4	Isoform 4 of Multidrug resistance-associated protein 4	5.828	4.13.E-03
O15439-3	Isoform 3 of Multidrug resistance-associated protein 4	5.828	4.13.E-03
Q8N392-2	Isoform 2 of Rho GTPase-activating protein 18	5.824	1.80.E-03
Q8N392	Rho GTPase-activating protein 18	5.824	2.57.E-03
Q04727-4	Isoform 4 of Transducin-like enhancer protein 4	5.82	4.89.E-03
Q04727-3	Isoform 3 of Transducin-like enhancer protein 4	5.82	4.89.E-03
Q04727-2	Isoform 2 of Transducin-like enhancer protein 4	5.82	4.89.E-03
Q04727	Transducin-like enhancer protein 4	5.82	4.89.E-03
Q96AY4	Tetratricopeptide repeat protein 28	5.812	9.95.E-04
Q86SK9-2	Isoform 2 of Stearoyl-CoA desaturase 5	5.807	2.57.E-03
Q86SK9	Stearoyl-CoA desaturase 5	5.807	2.57.E-03
O60229-4	Isoform 4 of Kalirin	5.807	9.23.E-03
O60229-6	Isoform 6 of Kalirin	5.807	9.23.E-03

O60229	Kalirin	5.807	9.23.E-03
A6NFI3	Zinc finger protein 316	5.747	2.43.E-02
Q15742	NGFI-A-binding protein 2	5.747	1.68.E-02
Q15742-3	Isoform 3 of NGFI-A-binding protein 2	5.747	1.68.E-02
Q15742-2	Isoform 2 of NGFI-A-binding protein 2	5.747	1.68.E-02
Q8WXH0-2	Isoform 2 of Nesprin-2	5.699	6.84.E-05
Q8WXH0	Nesprin-2	5.699	6.84.E-05
P13591-6	Isoform 6 of Neural cell adhesion molecule 1	5.683	4.14.E-04
Q9C0H2-3	Isoform 3 of Protein tweety homolog 3	5.627	2.65.E-03
P13591-4	Isoform 4 of Neural cell adhesion molecule 1	5.609	9.88.E-05
P13591-3	Isoform 3 of Neural cell adhesion molecule 1	5.609	9.85.E-05
Q7L7V1	Putative pre-mRNA-splicing factor ATP-dependent RNA helicase DHX32	5.594	1.04.E-02
Q7L7V1-2	Isoform 2 of Putative pre-mRNA-splicing factor ATP-dependent RNA helicase DHX32	5.594	1.04.E-02
Q9UIJ7-3	Isoform 3 of GTP:AMP phosphotransferase AK3, mitochondrial	5.48	6.95.E-03
Q9UIJ7	GTP:AMP phosphotransferase AK3, mitochondrial	5.48	6.95.E-03
Q9Y4F1-3	Isoform 3 of FERM, ARHGEF and pleckstrin domain-containing protein 1	5.48	5.89.E-03
Q8WXH0-4	Isoform 4 of Nesprin-2	5.456	7.57.E-04
Q8WXH0-10	Isoform 10 of Nesprin-2	5.447	1.76.E-03
P46439	Glutathione S-transferase Mu 5	5.443	5.97.E-04
O15439-2	Isoform 2 of Multidrug resistance-associated protein 4	5.441	6.48.E-03
O15439	Multidrug resistance-associated protein 4	5.441	6.93.E-03
Q7L099-3	Isoform 3 of Protein RUFY3	5.415	1.64.E-04
Q7L099-4	Isoform 4 of Protein RUFY3	5.415	1.53.E-04
Q6NTF9	Rhomboid domain-containing protein 2	5.37	8.33.E-03
Q6NTF9-2	Isoform 2 of Rhomboid domain-containing protein 2	5.37	8.33.E-03
Q9Y2B9	cAMP-dependent protein kinase inhibitor gamma	5.349	1.11.E-02
P07197	Neurofilament medium polypeptide	5.34	1.23.E-04
Q9NVH1-2	Isoform 2 of DnaJ homolog subfamily C member 11	5.336	1.12.E-02
Q9NVH1-3	Isoform 3 of DnaJ homolog subfamily C member 11	5.336	1.12.E-02
Q9NVH1	DnaJ homolog subfamily C member 11	5.336	1.12.E-02
Q8WXH0-7	Isoform 7 of Nesprin-2	5.316	4.76.E-04
Q2Y0W8-6	Isoform 6 of Electroneutral sodium bicarbonate exchanger 1	5.3	7.11.E-03
Q2Y0W8-8	Isoform 8 of Electroneutral sodium bicarbonate exchanger 1	5.3	7.11.E-03
Q2Y0W8-7	Isoform 7 of Electroneutral sodium bicarbonate exchanger 1	5.3	7.11.E-03
P23471-3	Isoform 3 of Receptor-type tyrosine-protein phosphatase zeta	5.268	7.90.E-03
P23471	Receptor-type tyrosine-protein phosphatase zeta	5.268	7.90.E-03
P23471-2	Isoform 2 of Receptor-type tyrosine-protein phosphatase zeta	5.268	7.90.E-03
Q13683	Integrin alpha-7	5.217	1.05.E-02
Q13683-9	Isoform Alpha-7X2DB of Integrin alpha-7	5.217	1.05.E-02
Q13683-10	Isoform Alpha-7X1X2A of Integrin alpha-7	5.217	1.05.E-02
Q13683-7	Isoform Alpha-7X2B of Integrin alpha-7	5.217	1.05.E-02
Q13683-3	Isoform Alpha-7X1B of Integrin alpha-7	5.217	1.05.E-02
P02768	Albumin	5.215	8.57.E-03
P02768-2	Isoform 2 of Albumin	5.215	8.57.E-03
P02768-3	Isoform 3 of Albumin	5.215	4.71.E-03
P12081-3	Isoform 3 of Histidine--tRNA ligase, cytoplasmic	5.186	9.27.E-03
P12081-2	Isoform 2 of Histidine--tRNA ligase, cytoplasmic	5.186	9.27.E-03
P08138	Tumor necrosis factor receptor superfamily member 16	5.182	8.88.E-03
P08138-2	Isoform 2 of Tumor necrosis factor receptor superfamily member 16	5.182	7.96.E-03
Q01814	Plasma membrane calcium-transporting ATPase 2	5.165	8.21.E-03
Q01814-6	Isoform ZB of Plasma membrane calcium-transporting ATPase 2	5.165	8.21.E-03
Q01814-3	Isoform YA of Plasma membrane calcium-transporting ATPase 2	5.165	8.21.E-03
Q01814-2	Isoform WA of Plasma membrane calcium-transporting ATPase 2	5.165	8.21.E-03
Q01814-8	Isoform XB of Plasma membrane calcium-transporting ATPase 2	5.165	8.21.E-03
Q01814-4	Isoform ZA of Plasma membrane calcium-transporting ATPase 2	5.165	8.21.E-03
Q01814-5	Isoform YB of Plasma membrane calcium-transporting ATPase 2	5.165	8.21.E-03
Q01814-7	Isoform XA of Plasma membrane calcium-transporting ATPase 2	5.165	8.21.E-03
Q96RT1-8	Isoform 8 of Erbin	5.12	1.51.E-02
Q96RT1-5	Isoform 5 of Erbin	5.12	1.51.E-02
Q96RT1-6	Isoform 6 of Erbin	5.12	1.51.E-02
Q96RT1-4	Isoform 4 of Erbin	5.12	1.51.E-02
Q96RT1	Erbin	5.12	1.51.E-02
Q96RT1-9	Isoform 9 of Erbin	5.12	1.51.E-02
Q96RT1-3	Isoform 3 of Erbin	5.12	1.51.E-02

Q96RT1-2	Isoform 2 of Erbin	5.12	1.51.E-02
Q96RT1-7	Isoform 7 of Erbin	5.12	1.51.E-02
Q8N9N7	Leucine-rich repeat-containing protein 57	5.095	8.71.E-03
Q9Y4J8-7	Isoform 7 of Dystrobrevin alpha	5.094	6.51.E-03
Q9Y4J8-9	Isoform 9 of Dystrobrevin alpha	5.094	6.51.E-03
Q9Y2H6	Fibronectin type-III domain-containing protein 3A	5.084	1.61.E-03
Q9Y2H6-2	Isoform 2 of Fibronectin type-III domain-containing protein 3A	5.084	1.61.E-03
Q96DH6-3	Isoform 3 of RNA-binding protein Musashi homolog 2	5.083	1.25.E-03
Q8WXH0-12	Isoform 12 of Nesprin-2	5.073	9.86.E-03
Q8WXH0-5	Isoform 5 of Nesprin-2	5.073	9.86.E-03
Q8WXH0-11	Isoform 11 of Nesprin-2	5.073	9.86.E-03
Q8WXH0-6	Isoform 6 of Nesprin-2	5.073	9.86.E-03
Q6SZW1-2	Isoform 2 of NAD(+) hydrolase SARM1	5.065	1.60.E-03
Q8NBX0	Saccharopine dehydrogenase-like oxidoreductase	5.065	1.28.E-03
Q8WX93-7	Isoform 7 of Palladin	5.063	1.55.E-03
Q7L099-2	Isoform 2 of Protein RUFY3	5.044	2.79.E-02
Q8N573-3	Isoform 3 of Oxidation resistance protein 1	5.04	4.15.E-02
Q8N573-5	Isoform 5 of Oxidation resistance protein 1	5.04	4.15.E-02
Q8N573	Oxidation resistance protein 1	5.04	4.15.E-02
Q8N573-2	Isoform 2 of Oxidation resistance protein 1	5.04	4.15.E-02
Q8N573-8	Isoform 8 of Oxidation resistance protein 1	5.04	4.15.E-02
P57723-2	Isoform 2 of Poly(rC)-binding protein 4	5.021	1.04.E-02
P57723	Poly(rC)-binding protein 4	5.021	1.04.E-02
P17661	Desmin	4.988	2.24.E-04
Q8N370-4	Isoform 4 of Large neutral amino acids transporter small subunit 4	4.988	2.99.E-02
Q8N370-2	Isoform 2 of Large neutral amino acids transporter small subunit 4	4.988	2.99.E-02
Q8N370	Large neutral amino acids transporter small subunit 4	4.988	2.99.E-02
Q8N370-3	Isoform 3 of Large neutral amino acids transporter small subunit 4	4.988	2.99.E-02
Q9UHQ4-2	Isoform 2 of B-cell receptor-associated protein 29	4.908	1.49.E-02
Q9UHQ4	B-cell receptor-associated protein 29	4.908	1.49.E-02
Q8WXH0-3	Isoform 3 of Nesprin-2	4.867	1.43.E-03
Q13683-13	Isoform 2 of Integrin alpha-7	4.852	7.60.E-03
Q969Q5	Ras-related protein Rab-24	4.848	2.00.E-02
Q6ZMK1-4	Isoform 4 of Cysteine and histidine-rich protein 1	4.837	3.11.E-02
Q9Y4F1	FERM, ARHGEF and pleckstrin domain-containing protein 1	4.835	1.26.E-03
Q2Y0W8	Electroneutral sodium bicarbonate exchanger 1	4.833	6.95.E-03
Q2Y0W8-3	Isoform 3 of Electroneutral sodium bicarbonate exchanger 1	4.833	6.95.E-03
Q2Y0W8-5	Isoform 5 of Electroneutral sodium bicarbonate exchanger 1	4.833	6.95.E-03
Q2Y0W8-2	Isoform 2 of Electroneutral sodium bicarbonate exchanger 1	4.833	6.95.E-03
Q2Y0W8-4	Isoform 4 of Electroneutral sodium bicarbonate exchanger 1	4.833	6.95.E-03
P29317-2	Isoform 2 of Ephrin type-A receptor 2	4.826	1.23.E-02
P28161	Glutathione S-transferase Mu 2	4.824	5.04.E-04
P28161-2	Isoform 2 of Glutathione S-transferase Mu 2	4.824	5.07.E-04
Q8IV38	Ankyrin repeat and MYND domain-containing protein 2	4.788	4.03.E-04
P50993	Sodium/potassium-transporting ATPase subunit alpha-2	4.761	1.57.E-03
Q99574	Neuroserpin	4.759	2.59.E-02
Q9Y2Y0-2	Isoform 2 of ADP-ribosylation factor-like protein 2-binding protein	4.749	5.32.E-03
Q9Y2Y0	ADP-ribosylation factor-like protein 2-binding protein	4.749	5.32.E-03
Q13325	Interferon-induced protein with tetratricopeptide repeats 5	4.737	5.44.E-03
Q13325-2	Isoform 2 of Interferon-induced protein with tetratricopeptide repeats 5	4.737	5.44.E-03
P00395	Cytochrome c oxidase subunit 1	4.717	2.27.E-02
Q8N4T8	Carbonyl reductase family member 4	4.697	3.55.E-02
P07858	Cathepsin B	4.689	3.74.E-03
Q15126	Phosphomevalonate kinase	4.688	2.48.E-03
O43295-2	Isoform 2 of SLIT-ROBO Rho GTPase-activating protein 3	4.683	1.74.E-03
O43295	SLIT-ROBO Rho GTPase-activating protein 3	4.683	1.74.E-03
Q9C0H2-2	Isoform 2 of Protein tweety homolog 3	4.661	9.60.E-03
Q9C0H2-4	Isoform 4 of Protein tweety homolog 3	4.661	9.60.E-03
Q9C0H2	Protein tweety homolog 3	4.661	9.60.E-03
Q96P47-6	Isoform 6 of Arf-GAP with GTPase, ANK repeat and PH domain-containing protein 3	4.66	2.26.E-02
Q96P47-3	Isoform 3 of Arf-GAP with GTPase, ANK repeat and PH domain-containing protein 3	4.66	2.26.E-02
Q9P0M6	Core histone macro-H2A.2	4.647	3.11.E-02
Q15139	Serine/threonine-protein kinase D1	4.628	3.50.E-02
O75884	Serine hydrolase RBBP9	4.614	2.11.E-03

Q9BX67	Junctional adhesion molecule C	4.613	3.67.E-03
Q9BX67-2	Isoform 2 of Junctional adhesion molecule C	4.613	3.67.E-03
P48735-2	Isoform 2 of Isocitrate dehydrogenase [NADP], mitochondrial	4.586	4.54.E-04
P48735	Isocitrate dehydrogenase [NADP], mitochondrial	4.586	4.54.E-04
P26992	Ciliary neurotrophic factor receptor subunit alpha	4.583	2.21.E-02
O43295-3	Isoform 3 of SLIT-ROBO Rho GTPase-activating protein 3	4.574	9.32.E-03
Q96ER9-2	Isoform 2 of Mitochondrial potassium channel	4.573	2.56.E-02
Q96ER9	Mitochondrial potassium channel	4.573	2.56.E-02
Q9BVC6	Transmembrane protein 109	4.564	3.73.E-03
P48681	Nestin	4.553	4.82.E-04
P78559-2	Isoform 2 of Microtubule-associated protein 1A	4.539	5.46.E-04
Q9Y4F1-2	Isoform 2 of FERM, ARHGEF and pleckstrin domain-containing protein 1	4.537	2.67.E-03
P78559	Microtubule-associated protein 1A	4.505	7.09.E-04
P13591-5	Isoform 5 of Neural cell adhesion molecule 1	4.493	1.18.E-03
Q13423	NAD(P) transhydrogenase, mitochondrial	4.49	2.33.E-03
P49418	Amphiphysin	4.387	4.07.E-03
P49418-2	Isoform 2 of Amphiphysin	4.387	4.04.E-03
Q9UKG9-3	Isoform 3 of Peroxisomal carnitine O-octanoyltransferase	4.387	4.51.E-02
Q9UKG9	Peroxisomal carnitine O-octanoyltransferase	4.387	4.51.E-02
Q9UPT6-2	Isoform 2 of C-Jun-amino-terminal kinase-interacting protein 3	4.382	1.86.E-02
Q12792	Twinfilin-1	4.38	3.79.E-03
Q8NBI6-3	Isoform 3 of Xyloside xylosyltransferase 1	4.343	4.61.E-02
Q96KB5	Lymphokine-activated killer T-cell-originated protein kinase	4.342	2.92.E-03
Q96KB5-2	Isoform 2 of Lymphokine-activated killer T-cell-originated protein kinase	4.342	3.95.E-03
P30626	Sorcin	4.337	1.35.E-03
P30626-2	Isoform 2 of Sorcin	4.337	1.35.E-03
P30626-3	Isoform 3 of Sorcin	4.337	1.35.E-03
Q9BY67-2	Isoform 2 of Cell adhesion molecule 1	4.325	1.40.E-02
P35052-2	Isoform 2 of Glypican-1	4.324	2.28.E-02
Q9C0E8-4	Isoform 4 of Endoplasmic reticulum junction formation protein lunapark	4.317	4.27.E-03
Q9C0E8	Endoplasmic reticulum junction formation protein lunapark	4.317	4.27.E-03
Q9C0E8-2	Isoform 2 of Endoplasmic reticulum junction formation protein lunapark	4.317	4.27.E-03
Q9C0E8-3	Isoform 3 of Endoplasmic reticulum junction formation protein lunapark	4.317	4.60.E-03
P08473	Neprilysin	4.31	3.25.E-03
P35237	Serpin B6	4.299	8.64.E-04
O15068	Guanine nucleotide exchange factor DBS	4.273	5.00.E-02
O15068-8	Isoform 8 of Guanine nucleotide exchange factor DBS	4.273	5.00.E-02
O15068-9	Isoform 7 of Guanine nucleotide exchange factor DBS	4.273	5.00.E-02
O15068-4	Isoform 4 of Guanine nucleotide exchange factor DBS	4.273	5.00.E-02
O15068-5	Isoform 5 of Guanine nucleotide exchange factor DBS	4.273	5.00.E-02
O15068-6	Isoform 6 of Guanine nucleotide exchange factor DBS	4.273	5.00.E-02
O15068-2	Isoform 2 of Guanine nucleotide exchange factor DBS	4.273	5.00.E-02
O15068-3	Isoform 3 of Guanine nucleotide exchange factor DBS	4.273	5.00.E-02
O15068-10	Isoform 9 of Guanine nucleotide exchange factor DBS	4.273	5.00.E-02
P11137	Microtubule-associated protein 2	4.272	8.63.E-04
P11137-3	Isoform 3 of Microtubule-associated protein 2	4.272	8.63.E-04
Q8WX93-8	Isoform 8 of Palladin	4.246	6.74.E-03
Q8WX93-3	Isoform 3 of Palladin	4.246	6.74.E-03
Q8WX93-2	Isoform 2 of Palladin	4.246	4.41.E-03
Q8WX93-4	Isoform 4 of Palladin	4.246	6.74.E-03
Q8WX93-5	Isoform 5 of Palladin	4.246	6.74.E-03
Q8WX93	Palladin	4.246	6.74.E-03
Q8WXH0-13	Isoform 13 of Nesprin-2	4.232	3.09.E-03
Q9P232	Contactin-3	4.229	1.74.E-02
Q9NT99	Leucine-rich repeat-containing protein 4B	4.221	1.04.E-02
A8MVJ9	Putative histone PARylation factor 1-like	4.213	2.74.E-02
P14406	Cytochrome c oxidase subunit 7A2, mitochondrial	4.212	3.77.E-03
O14967	Calmegin	4.192	7.28.E-03
P51812	Ribosomal protein S6 kinase alpha-3	4.183	2.01.E-03
O95757	Heat shock 70 kDa protein 4L	4.182	1.35.E-03
P08247	Synaptophysin	4.173	3.21.E-02
O75884-2	Isoform 2 of Serine hydrolase RBBP9	4.167	5.14.E-03
Q16698	2,4-dienoyl-CoA reductase, mitochondrial	4.146	1.43.E-03
Q16698-2	Isoform 2 of 2,4-dienoyl-CoA reductase, mitochondrial	4.146	1.43.E-03

Q9Y243	RAC-gamma serine/threonine-protein kinase	4.144	3.23.E-02
Q9Y243-2	Isoform 2 of RAC-gamma serine/threonine-protein kinase	4.144	3.23.E-02
O60486	Plexin-C1	4.139	1.34.E-02
Q9Y4D7	Plexin-D1	4.139	1.34.E-02
P37235	Hippocalcin-like protein 1	4.126	2.58.E-02
Q6ZVM7-2	Isoform 2 of TOM1-like protein 2	4.104	3.26.E-02
Q6ZVM7-4	Isoform 4 of TOM1-like protein 2	4.104	3.89.E-02
P20020-2	Isoform A of Plasma membrane calcium-transporting ATPase 1	4.088	1.13.E-03
P20020-5	Isoform E of Plasma membrane calcium-transporting ATPase 1	4.088	1.13.E-03
Q15165-3	Isoform 3 of Serum paraoxonase/arylesterase 2	4.082	5.26.E-03
Q15165	Serum paraoxonase/arylesterase 2	4.082	5.26.E-03
Q15165-1	Isoform 1 of Serum paraoxonase/arylesterase 2	4.082	5.26.E-03
Q8NBF2	NHL repeat-containing protein 2	4.079	1.32.E-03
O00562	Membrane-associated phosphatidylinositol transfer protein 1	4.077	2.22.E-02
O00562-2	Isoform 2 of Membrane-associated phosphatidylinositol transfer protein 1	4.077	2.22.E-02
Q14332	Frizzled-2	4.075	3.59.E-02
P20020-1	Isoform D of Plasma membrane calcium-transporting ATPase 1	4.073	1.17.E-03
P20020-4	Isoform C of Plasma membrane calcium-transporting ATPase 1	4.073	1.17.E-03
P20020	Plasma membrane calcium-transporting ATPase 1	4.073	1.17.E-03
P20020-6	Isoform K of Plasma membrane calcium-transporting ATPase 1	4.073	1.17.E-03
P35052	Glypican-1	4.068	5.99.E-03
Q8NBF2-2	Isoform 2 of NHL repeat-containing protein 2	4.037	4.16.E-03
Q8NBI6	Xyloside xylosyltransferase 1	3.988	4.06.E-02
Q4G0J3-3	Isoform 3 of La-related protein 7	3.974	7.52.E-03
Q4G0J3	La-related protein 7	3.974	7.52.E-03
P09488-2	Isoform 2 of Glutathione S-transferase Mu 1	3.971	9.09.E-03
P09488	Glutathione S-transferase Mu 1	3.971	9.09.E-03
Q03013-2	Isoform 2 of Glutathione S-transferase Mu 4	3.971	7.88.E-03
Q03013	Glutathione S-transferase Mu 4	3.971	7.88.E-03
Q03013-3	Isoform 3 of Glutathione S-transferase Mu 4	3.971	7.88.E-03
Q08345-4	Isoform 3 of Epithelial discoidin domain-containing receptor 1	3.971	2.38.E-02
Q8WX93-9	Isoform 9 of Palladin	3.97	4.69.E-03
P12931-2	Isoform 2 of Proto-oncogene tyrosine-protein kinase Src	3.953	1.14.E-02
P12931	Proto-oncogene tyrosine-protein kinase Src	3.953	1.14.E-02
P03891	NADH-ubiquinone oxidoreductase chain 2	3.953	2.55.E-02
Q9H6U8-2	Isoform 2 of Alpha-1,2-mannosyltransferase ALG9	3.927	3.77.E-02
Q9H6U8-4	Isoform 4 of Alpha-1,2-mannosyltransferase ALG9	3.927	3.77.E-02
Q9NUJ1-3	Isoform 3 of Mycophenolic acid acyl-glucuronide esterase, mitochondrial	3.896	2.93.E-02
Q6ZMK1-2	Isoform 2 of Cysteine and histidine-rich protein 1	3.887	4.30.E-02
Q6ZMK1	Cysteine and histidine-rich protein 1	3.887	4.30.E-02
P42575	Caspase-2	3.848	4.33.E-02
P42575-2	Isoform 2 of Caspase-2	3.848	4.33.E-02
O43790	Keratin, type II cuticular Hb6	3.842	1.81.E-03
P78386	Keratin, type II cuticular Hb5	3.842	1.81.E-03
Q8N1N4-2	Isoform 2 of Keratin, type II cytoskeletal 78	3.842	1.81.E-03
Q14533	Keratin, type II cuticular Hb1	3.842	1.81.E-03
Q8N1N4	Keratin, type II cytoskeletal 78	3.842	1.81.E-03
P78385	Keratin, type II cuticular Hb3	3.842	1.81.E-03
Q9BY67-3	Isoform 3 of Cell adhesion molecule 1	3.827	1.73.E-02
Q9BY67-4	Isoform 4 of Cell adhesion molecule 1	3.827	1.73.E-02
Q9BY67	Cell adhesion molecule 1	3.827	1.73.E-02
Q9BY67-5	Isoform 5 of Cell adhesion molecule 1	3.827	1.73.E-02
Q08623-2	Isoform 2 of Pseudouridine-5'-phosphatase	3.815	1.09.E-02
Q9H0A8-2	Isoform 2 of COMM domain-containing protein 4	3.812	4.26.E-02
Q9H0A8-3	Isoform 3 of COMM domain-containing protein 4	3.812	4.26.E-02
Q9H0A8	COMM domain-containing protein 4	3.812	4.26.E-02
P04818	Thymidylate synthase	3.801	2.58.E-03
Q9BUR5	MICOS complex subunit MIC26	3.795	2.56.E-02
Q9BUR5-2	Isoform 2 of MICOS complex subunit MIC26	3.795	2.56.E-02
Q5SSJ5	Heterochromatin protein 1-binding protein 3	3.792	8.82.E-03
Q6ZVM7	TOM1-like protein 2	3.786	4.72.E-02
Q6ZVM7-5	Isoform 5 of TOM1-like protein 2	3.786	4.72.E-02
Q92522	Histone H1.10	3.781	1.74.E-03
P11532-16	Isoform 10 of Dystrophin	3.773	7.65.E-03

P11532-13	Isoform 7 of Dystrophin	3.773	7.65.E-03
Q12792-4	Isoform 4 of Twinfilin-1	3.761	8.97.E-03
Q6U841-4	Isoform 4 of Sodium-driven chloride bicarbonate exchanger	3.754	4.76.E-02
P11532-14	Isoform 8 of Dystrophin	3.745	4.34.E-03
P11532-11	Isoform 3 of Dystrophin	3.745	4.34.E-03
P11532-12	Isoform 6 of Dystrophin	3.745	4.34.E-03
P11532-3	Isoform 5 of Dystrophin	3.745	4.34.E-03
P11532-15	Isoform 9 of Dystrophin	3.745	4.46.E-03
P11532-2	Isoform 4 of Dystrophin	3.745	4.34.E-03
P11532	Dystrophin	3.745	4.34.E-03
P11532-4	Isoform 2 of Dystrophin	3.745	4.34.E-03
Q9BW30	Tubulin polymerization-promoting protein family member 3	3.744	1.98.E-02
P11532-17	Isoform 11 of Dystrophin	3.738	6.32.E-03
Q96DH6	RNA-binding protein Musashi homolog 2	3.737	1.23.E-02
P00403	Cytochrome c oxidase subunit 2	3.712	4.32.E-03
Q9H1E5	Thioredoxin-related transmembrane protein 4	3.706	1.53.E-02
P04818-2	Isoform 2 of Thymidylate synthase	3.703	4.62.E-03
Q7L1T6	Cytochrome b5 reductase 4	3.7	4.08.E-02
O15031	Plexin-B2	3.699	4.85.E-03
Q92558	Wiskott-Aldrich syndrome protein family member 1	3.696	1.10.E-02
P41217-3	Isoform 3 of OX-2 membrane glycoprotein	3.689	9.06.E-03
P41217	OX-2 membrane glycoprotein	3.689	9.06.E-03
P41217-2	Isoform 2 of OX-2 membrane glycoprotein	3.689	9.06.E-03
Q12979-3	Isoform 3 of Active breakpoint cluster region-related protein	3.654	4.94.E-02
Q96DH6-2	Isoform 2 of RNA-binding protein Musashi homolog 2	3.64	1.75.E-02
Q15417-2	Isoform 2 of Calponin-3	3.637	1.14.E-02
P04818-3	Isoform 3 of Thymidylate synthase	3.637	9.69.E-03
P24941	Cyclin-dependent kinase 2	3.627	1.51.E-02
P24941-2	Isoform 2 of Cyclin-dependent kinase 2	3.627	1.98.E-02
Q9UPQ3-3	Isoform 3 of Arf-GAP with GTPase, ANK repeat and PH domain-containing protein 1	3.621	2.73.E-02
Q9UBH6	Xenotropic and polytropic retrovirus receptor 1	3.619	4.05.E-02
Q8TCZ2-6	Isoform 6 of CD99 antigen-like protein 2	3.603	2.66.E-02
P21796	Voltage-dependent anion-selective channel protein 1	3.593	2.95.E-03
Q9Y512	Sorting and assembly machinery component 50 homolog	3.583	3.40.E-02
O95865	N(G),N(G)-dimethylarginine dimethylaminohydrolase 2	3.582	4.28.E-03
P30085-2	Isoform 2 of UMP-CMP kinase	3.567	2.87.E-02
Q9UBH6-2	Isoform 2 of Xenotropic and polytropic retrovirus receptor 1	3.549	4.77.E-02
Q8N9F7-3	Isoform 3 of Lysophospholipase D GDPD1	3.541	4.99.E-02
Q8N9F7-2	Isoform 2 of Lysophospholipase D GDPD1	3.541	4.99.E-02
Q9H0Q3-2	Isoform 2 of FXYD domain-containing ion transport regulator 6	3.533	4.70.E-02
P04083	Annexin A1	3.532	3.33.E-03
Q13641	Trophoblast glycoprotein	3.527	1.38.E-02
Q12979-4	Isoform 4 of Active breakpoint cluster region-related protein	3.527	4.50.E-02
Q12979-2	Isoform Short of Active breakpoint cluster region-related protein	3.527	4.50.E-02
Q12979	Active breakpoint cluster region-related protein	3.527	4.50.E-02
Q15121-2	Isoform 2 of Astrocytic phosphoprotein PEA-15	3.498	4.79.E-03
Q15121	Astrocytic phosphoprotein PEA-15	3.498	4.79.E-03
Q14108	Lysosome membrane protein 2	3.481	5.07.E-03
Q9HC38-3	Isoform 3 of Glyoxalase domain-containing protein 4	3.473	5.80.E-03
Q9HCM2-4	Isoform 4 of Plexin-A4	3.472	3.57.E-02
P28676	Grancalcin	3.454	4.93.E-02
Q7LGA3	Heparan sulfate 2-O-sulfotransferase 1	3.447	4.16.E-02
Q14011	Cold-inducible RNA-binding protein	3.435	4.04.E-03
Q13557-10	Isoform Delta 10 of Calcium/calmodulin-dependent protein kinase type II subunit delta	3.433	8.30.E-03
Q13557-6	Isoform Delta 9 of Calcium/calmodulin-dependent protein kinase type II subunit delta	3.433	8.30.E-03
Q13557-11	Isoform Delta 11 of Calcium/calmodulin-dependent protein kinase type II subunit delta	3.433	7.74.E-03
Q13557	Calcium/calmodulin-dependent protein kinase type II subunit delta	3.433	7.74.E-03
Q13557-12	Isoform Delta 12 of Calcium/calmodulin-dependent protein kinase type II subunit delta	3.433	7.74.E-03
Q13557-8	Isoform Delta 6 of Calcium/calmodulin-dependent protein kinase type II subunit delta	3.433	7.74.E-03
Q13557-4	Isoform Delta 4 of Calcium/calmodulin-dependent protein kinase type II subunit delta	3.404	9.18.E-03
Q13557-3	Isoform Delta 3 of Calcium/calmodulin-dependent protein kinase type II subunit delta	3.404	9.18.E-03
Q13557-9	Isoform Delta 7 of Calcium/calmodulin-dependent protein kinase type II subunit delta	3.404	9.18.E-03
Q13557-5	Isoform Delta 8 of Calcium/calmodulin-dependent protein kinase type II subunit delta	3.404	9.18.E-03
P22694-10	Isoform 10 of cAMP-dependent protein kinase catalytic subunit beta	3.399	8.09.E-03

Q96K76	Ubiquitin carboxyl-terminal hydrolase 47	3.397	4.36.E-03
Q96K76-2	Isoform 2 of Ubiquitin carboxyl-terminal hydrolase 47	3.397	4.36.E-03
Q96K76-4	Isoform 4 of Ubiquitin carboxyl-terminal hydrolase 47	3.397	4.36.E-03
Q86YR5-3	Isoform 3 of G-protein-signaling modulator 1	3.392	3.73.E-02
Q9H3Z4-2	Isoform 2 of DnaJ homolog subfamily C member 5	3.378	2.48.E-02
Q9UPY3-3	Isoform 3 of Endoribonuclease Dicer	3.378	1.90.E-02
Q9H0V9-3	Isoform 3 of VIP36-like protein	3.366	2.46.E-02
Q14108-2	Isoform 2 of Lysosome membrane protein 2	3.359	7.11.E-03
Q5EB52	Mesoderm-specific transcript homolog protein	3.357	1.59.E-02
Q5EB52-2	Isoform 2 of Mesoderm-specific transcript homolog protein	3.357	1.59.E-02
Q9ULK5	Vang-like protein 2	3.354	2.82.E-02
Q5EB52-3	Isoform 3 of Mesoderm-specific transcript homolog protein	3.35	1.96.E-02
P31321	cAMP-dependent protein kinase type I-beta regulatory subunit	3.349	2.56.E-02
Q8NEY1-5	Isoform 5 of Neuron navigator 1	3.346	1.97.E-02
Q9UIJ7-2	Isoform 2 of GTP:AMP phosphotransferase AK3, mitochondrial	3.339	4.06.E-02
Q8N9F7	Lysophospholipase D GDPD1	3.312	4.29.E-02
Q5SSJ5-2	Isoform 2 of Heterochromatin protein 1-binding protein 3	3.308	1.55.E-02
Q5SSJ5-3	Isoform 3 of Heterochromatin protein 1-binding protein 3	3.308	2.10.E-02
Q9H9B4	Sideroflexin-1	3.293	6.90.E-03
P30085-3	Isoform 3 of UMP-CMP kinase	3.293	1.83.E-02
P30085	UMP-CMP kinase	3.293	1.83.E-02
O00192	Armadillo repeat protein deleted in velo-cardio-facial syndrome	3.291	2.61.E-02
O00192-2	Isoform Short of Armadillo repeat protein deleted in velo-cardio-facial syndrome	3.291	2.61.E-02
O00560-3	Isoform 3 of Syntenin-1	3.281	2.85.E-02
O00560-2	Isoform 2 of Syntenin-1	3.281	2.85.E-02
O00560	Syntenin-1	3.281	2.85.E-02
Q8TB36	Ganglioside-induced differentiation-associated protein 1	3.247	4.85.E-02
Q8TB36-2	Isoform 2 of Ganglioside-induced differentiation-associated protein 1	3.247	4.71.E-02
Q9HC38	Glyoxalase domain-containing protein 4	3.245	5.95.E-03
O60518	Ran-binding protein 6	3.237	2.23.E-02
Q9Y666	Solute carrier family 12 member 7	3.237	3.14.E-02
Q9HC38-2	Isoform 2 of Glyoxalase domain-containing protein 4	3.235	6.07.E-03
P10644-2	Isoform 2 of cAMP-dependent protein kinase type I-alpha regulatory subunit	3.222	7.30.E-03
Q14011-2	Isoform 2 of Cold-inducible RNA-binding protein	3.217	6.38.E-03
Q9UBG0	C-type mannose receptor 2	3.209	2.67.E-02
O75914-4	Isoform 4 of Serine/threonine-protein kinase PAK 3	3.204	4.49.E-02
O75914-2	Isoform 2 of Serine/threonine-protein kinase PAK 3	3.204	4.49.E-02
O75914	Serine/threonine-protein kinase PAK 3	3.204	4.49.E-02
O75914-3	Isoform 3 of Serine/threonine-protein kinase PAK 3	3.204	4.49.E-02
Q9NUJ1	Mycophenolic acid acyl-glucuronide esterase, mitochondrial	3.201	1.27.E-02
P10644	cAMP-dependent protein kinase type I-alpha regulatory subunit	3.193	1.13.E-02
Q08623-4	Isoform 4 of Pseudouridine-5'-phosphatase	3.172	2.09.E-02
Q08722-3	Isoform OA3-305 of Leukocyte surface antigen CD47	3.142	2.66.E-02
Q08722-4	Isoform OA3-312 of Leukocyte surface antigen CD47	3.142	2.66.E-02
Q08722-2	Isoform OA3-293 of Leukocyte surface antigen CD47	3.142	2.66.E-02
P17612-2	Isoform 2 of cAMP-dependent protein kinase catalytic subunit alpha	3.13	4.05.E-02
P17612	cAMP-dependent protein kinase catalytic subunit alpha	3.13	4.05.E-02
P24752-2	Isoform 2 of Acetyl-CoA acetyltransferase, mitochondrial	3.128	4.05.E-02
Q08722	Leukocyte surface antigen CD47	3.113	2.88.E-02
Q9NUJ1-2	Isoform 2 of Mycophenolic acid acyl-glucuronide esterase, mitochondrial	3.112	1.38.E-02
P07196	Neurofilament light polypeptide	3.109	3.47.E-02
Q86UW7-3	Isoform 3 of Calcium-dependent secretion activator 2	3.101	4.85.E-02
Q86UW7	Calcium-dependent secretion activator 2	3.101	4.85.E-02
Q86UW7-2	Isoform 2 of Calcium-dependent secretion activator 2	3.101	4.85.E-02
Q86YR5-4	Isoform 4 of G-protein-signaling modulator 1	3.091	3.60.E-02
Q86YR5	G-protein-signaling modulator 1	3.091	3.60.E-02
P09211	Glutathione S-transferase P	3.088	8.24.E-03
O95336	6-phosphogluconolactonase	3.069	8.46.E-03
Q9NRZ5-2	Isoform 2 of 1-acyl-sn-glycerol-3-phosphate acyltransferase delta	3.062	4.47.E-02
Q5IJ48-3	Isoform 3 of Protein crumbs homolog 2	3.045	2.42.E-02
O00483	Cytochrome c oxidase subunit NDUFA4	3.042	4.10.E-02
P04179-4	Isoform 4 of Superoxide dismutase [Mn], mitochondrial	3.041	1.35.E-02
P04179	Superoxide dismutase [Mn], mitochondrial	3.041	1.35.E-02
Q8NEY1-3	Isoform 3 of Neuron navigator 1	3.039	3.53.E-02

Q8NEY1	Neuron navigator 1	3.039	3.53.E-02
Q8NEY1-7	Isoform 7 of Neuron navigator 1	3.039	3.53.E-02
Q8NEY1-2	Isoform 2 of Neuron navigator 1	3.039	3.53.E-02
Q8NEY1-4	Isoform 4 of Neuron navigator 1	3.039	3.53.E-02
Q16513	Serine/threonine-protein kinase N2	3.037	4.71.E-02
Q16513-3	Isoform 3 of Serine/threonine-protein kinase N2	3.037	4.71.E-02
Q16513-2	Isoform 2 of Serine/threonine-protein kinase N2	3.037	4.71.E-02
Q16513-4	Isoform 4 of Serine/threonine-protein kinase N2	3.037	3.91.E-02
Q08623	Pseudouridine-5'-phosphatase	3.034	2.70.E-02
P31146	Coronin-1A	3.023	1.33.E-02
P24752	Acetyl-CoA acetyltransferase, mitochondrial	3.014	2.85.E-02
Q8N158	Glypican-2	2.986	3.78.E-02
P04179-2	Isoform 2 of Superoxide dismutase [Mn], mitochondrial	2.976	2.53.E-02
P22694-7	Isoform 7 of cAMP-dependent protein kinase catalytic subunit beta	2.969	1.46.E-02
P22694-5	Isoform 5 of cAMP-dependent protein kinase catalytic subunit beta	2.969	1.46.E-02
P22694-3	Isoform 3 of cAMP-dependent protein kinase catalytic subunit beta	2.969	1.46.E-02
P22694-2	Isoform 2 of cAMP-dependent protein kinase catalytic subunit beta	2.969	1.46.E-02
P22694	cAMP-dependent protein kinase catalytic subunit beta	2.969	1.46.E-02
P22694-4	Isoform 4 of cAMP-dependent protein kinase catalytic subunit beta	2.969	1.46.E-02
Q8WXF7	Atlastin-1	2.956	4.25.E-02
Q8WXF7-2	Isoform 2 of Atlastin-1	2.956	4.25.E-02
Q9ULH0-4	Isoform 4 of Kinase D-interacting substrate of 220 kDa	2.946	3.67.E-02
Q9ULH0	Kinase D-interacting substrate of 220 kDa	2.946	3.67.E-02
P52306-2	Isoform 2 of Rap1 GTPase-GDP dissociation stimulator 1	2.941	3.21.E-02
P52306-6	Isoform 6 of Rap1 GTPase-GDP dissociation stimulator 1	2.941	3.21.E-02
P52306-3	Isoform 3 of Rap1 GTPase-GDP dissociation stimulator 1	2.941	3.21.E-02
Q9NRZ5	1-acyl-sn-glycerol-3-phosphate acyltransferase delta	2.941	3.50.E-02
P46821	Microtubule-associated protein 1B	2.94	1.13.E-02
Q08623-3	Isoform 3 of Pseudouridine-5'-phosphatase	2.938	3.08.E-02
Q9H0V9-2	Isoform 2 of VIP36-like protein	2.911	3.47.E-02
Q9H0V9	VIP36-like protein	2.911	3.47.E-02
P27707	Deoxycytidine kinase	2.904	3.93.E-02
Q15631	Translin	2.902	1.13.E-02
Q8NCW5-2	Isoform 2 of NAD(P)H-hydrate epimerase	2.897	4.42.E-02
Q9Y394-2	Isoform 2 of Dehydrogenase/reductase SDR family member 7	2.897	4.85.E-02
Q9Y394	Dehydrogenase/reductase SDR family member 7	2.897	4.85.E-02
Q9HBK9	Arsenite methyltransferase	2.887	4.54.E-02
Q9UKA9-5	Isoform 5 of Polypyrimidine tract-binding protein 2	2.878	3.43.E-02
Q8NCW5	NAD(P)H-hydrate epimerase	2.87	4.35.E-02
Q9ULH0-2	Isoform 2 of Kinase D-interacting substrate of 220 kDa	2.864	4.71.E-02
P31327-2	Isoform 2 of Carbamoyl-phosphate synthase [ammonia], mitochondrial	2.859	4.70.E-02
Q15349-2	Isoform 2 of Ribosomal protein S6 kinase alpha-2	2.852	3.77.E-02
Q15349	Ribosomal protein S6 kinase alpha-2	2.852	3.77.E-02
Q15349-3	Isoform 3 of Ribosomal protein S6 kinase alpha-2	2.852	3.77.E-02
Q9UPY3	Endoribonuclease Dicer	2.828	2.22.E-02
P33176	Kinesin-1 heavy chain	2.827	1.43.E-02
Q8NBJ5	Procollagen galactosyltransferase 1	2.824	2.58.E-02
Q8IZ81	ELMO domain-containing protein 2	2.81	3.82.E-02
Q9H0Q3	FXYD domain-containing ion transport regulator 6	2.804	4.34.E-02
Q16891-2	Isoform 2 of MICOS complex subunit MIC60	2.794	3.17.E-02
Q16891	MICOS complex subunit MIC60	2.794	3.17.E-02
Q86Y13	E3 ubiquitin-protein ligase DZIP3	2.789	4.93.E-02
P23368-2	Isoform 2 of NAD-dependent malic enzyme, mitochondrial	2.788	2.15.E-02
Q9UPY3-2	Isoform 2 of Endoribonuclease Dicer	2.788	2.78.E-02
P04179-3	Isoform 3 of Superoxide dismutase [Mn], mitochondrial	2.783	4.37.E-02
Q16658	Fascin	2.768	1.62.E-02
P52306-4	Isoform 4 of Rap1 GTPase-GDP dissociation stimulator 1	2.764	3.88.E-02
Q4G0F5	Vacuolar protein sorting-associated protein 26B	2.75	4.36.E-02
O75915	PRA1 family protein 3	2.737	4.55.E-02
P23368	NAD-dependent malic enzyme, mitochondrial	2.722	1.66.E-02
Q9NRN7	L-aminoadipate-semialdehyde dehydrogenase-phosphopantetheinyl transferase	2.711	3.75.E-02
O43657	Tetraspanin-6	2.71	2.86.E-02
Q16891-3	Isoform 3 of MICOS complex subunit MIC60	2.702	4.57.E-02
Q16891-4	Isoform 4 of MICOS complex subunit MIC60	2.702	4.54.E-02

P26232-3	Isoform 3 of Catenin alpha-2	2.698	2.75.E-02
Q3SXM5	Inactive hydroxysteroid dehydrogenase-like protein 1	2.698	3.49.E-02
Q9UKA9-2	Isoform 2 of Polypyrimidine tract-binding protein 2	2.697	3.01.E-02
Q9UKA9	Polypyrimidine tract-binding protein 2	2.697	3.01.E-02
Q16836-3	Isoform 3 of Hydroxyacyl-coenzyme A dehydrogenase, mitochondrial	2.695	1.87.E-02
Q16836-2	Isoform 2 of Hydroxyacyl-coenzyme A dehydrogenase, mitochondrial	2.695	1.87.E-02
Q16836	Hydroxyacyl-coenzyme A dehydrogenase, mitochondrial	2.695	1.87.E-02
P52306-5	Isoform 5 of Rap1 GTPase-GDP dissociation stimulator 1	2.693	3.40.E-02
P52306	Rap1 GTPase-GDP dissociation stimulator 1	2.693	3.40.E-02
P26232-6	Isoform 6 of Catenin alpha-2	2.69	4.67.E-02
Q8WZA0	Protein LZIC	2.672	4.81.E-02
Q8WZA0-2	Isoform 2 of Protein LZIC	2.672	4.81.E-02
Q15631-2	Isoform 2 of Translin	2.64	3.73.E-02
P26232-2	Isoform 2 of Catenin alpha-2	2.623	3.11.E-02
P26232-5	Isoform 5 of Catenin alpha-2	2.623	3.11.E-02
Q07866-4	Isoform J of Kinesin light chain 1	2.586	2.41.E-02
Q07866-5	Isoform K of Kinesin light chain 1	2.586	2.41.E-02
Q07866-10	Isoform D of Kinesin light chain 1	2.586	2.41.E-02
Q07866-9	Isoform I of Kinesin light chain 1	2.586	2.41.E-02
Q07866-2	Isoform C of Kinesin light chain 1	2.586	2.41.E-02
Q07866	Kinesin light chain 1	2.586	2.41.E-02
Q07866-7	Isoform P of Kinesin light chain 1	2.586	2.41.E-02
Q07866-6	Isoform N of Kinesin light chain 1	2.586	2.41.E-02
Q13177	Serine/threonine-protein kinase PAK 2	2.584	2.43.E-02
Q13642	Four and a half LIM domains protein 1	2.584	2.42.E-02
P07954	Fumarate hydratase, mitochondrial	2.558	3.11.E-02
P07954-2	Isoform Cytoplasmic of Fumarate hydratase, mitochondrial	2.558	3.11.E-02
O00291-4	Isoform 4 of Huntingtin-interacting protein 1	2.546	3.46.E-02
O00291	Huntingtin-interacting protein 1	2.546	3.46.E-02
Q07866-3	Isoform G of Kinesin light chain 1	2.545	2.64.E-02
O00291-3	Isoform 3 of Huntingtin-interacting protein 1	2.522	3.64.E-02
Q15334	Lethal(2) giant larvae protein homolog 1	2.506	2.94.E-02
A6NHL2-2	Isoform 2 of Tubulin alpha chain-like 3	2.504	2.90.E-02
A6NHL2	Tubulin alpha chain-like 3	2.504	2.90.E-02
Q96N67-5	Isoform 5 of Dedicator of cytokinesis protein 7	2.489	4.63.E-02
Q96N67-4	Isoform 4 of Dedicator of cytokinesis protein 7	2.489	4.63.E-02
Q96N67-3	Isoform 3 of Dedicator of cytokinesis protein 7	2.489	4.63.E-02
P11166	Solute carrier family 2, facilitated glucose transporter member 1	2.459	3.20.E-02
Q8WZA9	Immunity-related GTPase family Q protein	2.451	4.34.E-02
Q9NUQ9	CYFIP-related Rac1 interactor B	2.446	4.01.E-02
P55084	Trifunctional enzyme subunit beta, mitochondrial	2.439	3.34.E-02
P55084-2	Isoform 2 of Trifunctional enzyme subunit beta, mitochondrial	2.439	3.34.E-02
P11171-3	Isoform 3 of Protein 4.1	2.393	4.98.E-02
P61970	Nuclear transport factor 2	2.382	3.80.E-02
P14324-2	Isoform 2 of Farnesyl pyrophosphate synthase	2.364	3.95.E-02
Q9NQC3-4	Isoform 6 of Reticulon-4	2.36	3.99.E-02
P51648-2	Isoform 2 of Aldehyde dehydrogenase family 3 member A2	2.329	4.55.E-02
P51648	Aldehyde dehydrogenase family 3 member A2	2.329	4.55.E-02
P40939	Trifunctional enzyme subunit alpha, mitochondrial	2.312	4.45.E-02
P14324	Farnesyl pyrophosphate synthase	2.306	4.51.E-02
Q9NQC3-6	Isoform D of Reticulon-4	2.291	4.66.E-02
Q13642-3	Isoform 3 of Four and a half LIM domains protein 1	2.277	4.81.E-02
P10155	60 kDa SS-A/Ro ribonucleoprotein	2.273	4.86.E-02
P11171	Protein 4.1	2.271	4.81.E-02
O43795	Unconventional myosin-Ib	2.271	4.83.E-02
O43795-2	Isoform 2 of Unconventional myosin-Ib	2.271	4.83.E-02
Q14194-2	Isoform LCRMP-1 of Dihydropyrimidinase-related protein 1	2.268	4.92.E-02
P10768	S-formylglutathione hydrolase	2.262	4.97.E-02
P61019	Ras-related protein Rab-2A	2.251	4.87.E-02
P11171-2	Isoform 2 of Protein 4.1	2.243	4.94.E-02

Supplementary Table S1. (b) Proteins with increased levels in the NPCs (>2 fold)

Accession	Protein Name	Abundance Ratio: (NPC) / (iPSC)	Abundance Ratio p-Value: (NPC) / (iPSC)
Q7Z6L0	Proline-rich transmembrane protein 2	100	1.00.E-17
Q7Z6L0-2	Isoform 2 of Proline-rich transmembrane protein 2	100	1.00.E-17
Q7Z6L0-3	Isoform 3 of Proline-rich transmembrane protein 2	100	1.00.E-17
P26378	ELAV-like protein 4	100	1.00.E-17
P26378-1	Isoform 1 of ELAV-like protein 4	100	1.00.E-17
P26378-3	Isoform 3 of ELAV-like protein 4	100	1.00.E-17
P26378-5	Isoform 5 of ELAV-like protein 4	100	1.00.E-17
P26378-4	Isoform 4 of ELAV-like protein 4	100	1.00.E-17
P26378-2	Isoform 2 of ELAV-like protein 4	100	1.00.E-17
Q8IUH4	Palmitoyltransferase ZDHHC13	100	1.00.E-17
Q9H902-3	Isoform 3 of Receptor expression-enhancing protein 1	100	1.00.E-17
Q9H902	Receptor expression-enhancing protein 1	100	1.00.E-17
Q9H902-2	Isoform 2 of Receptor expression-enhancing protein 1	100	1.00.E-17
Q9GZT4	Serine racemase	100	1.00.E-17
O15217-2	Isoform 2 of Glutathione S-transferase A4	100	1.00.E-17
O94772-2	Isoform 2 of Lymphocyte antigen 6H	100	1.00.E-17
O94772	Lymphocyte antigen 6H	100	1.00.E-17
Q9HAR2-2	Isoform 2 of Adhesion G protein-coupled receptor L3	100	1.00.E-17
Q9HAR2-4	Isoform 4 of Adhesion G protein-coupled receptor L3	100	1.00.E-17
Q9HAR2	Adhesion G protein-coupled receptor L3	100	1.00.E-17
O60641-2	Isoform 2 of Clathrin coat assembly protein AP180	100	1.00.E-17
Q9HAP6-2	Isoform 2 of Protein lin-7 homolog B	100	1.00.E-17
Q9ULB1	Neurexin-1	100	1.00.E-17
Q07866-8	Isoform S of Kinesin light chain 1	100	1.00.E-17
Q8N4Q0	Prostaglandin reductase 3	100	1.00.E-17
Q96SB3	Neurabin-2	100	1.00.E-17
Q9BRK4	Leucine zipper putative tumor suppressor 2	100	1.00.E-17
Q9HAR2-3	Isoform 3 of Adhesion G protein-coupled receptor L3	100	1.00.E-17
P32418-2	Isoform 3 of Sodium/calcium exchanger 1	100	1.00.E-17
P32418-3	Isoform 7 of Sodium/calcium exchanger 1	100	1.00.E-17
P32418-4	Isoform 10 of Sodium/calcium exchanger 1	100	1.00.E-17
P32418	Sodium/calcium exchanger 1	100	1.00.E-17
P32418-5	Isoform 5 of Sodium/calcium exchanger 1	100	1.00.E-17
Q9ULB1-4	Isoform 4 of Neurexin-1	100	1.00.E-17
P58400-1	Isoform 3b of Neurexin-1-beta	100	1.00.E-17
P56159	GDNF family receptor alpha-1	100	1.00.E-17
P56159-2	Isoform 2 of GDNF family receptor alpha-1	100	1.00.E-17
Q6ZVF9	G protein-regulated inducer of neurite outgrowth 3	100	1.00.E-17
Q5T1Q4-2	Isoform 2 of Solute carrier family 35 member F1	100	1.00.E-17
Q5T1Q4	Solute carrier family 35 member F1	100	1.00.E-17
Q16526	Cryptochrome-1	100	1.00.E-17
Q9Y6I9	Testis-expressed protein 264	100	1.00.E-17
Q7Z6K5	Arpin	100	1.00.E-17
Q15173	Serine/threonine-protein phosphatase 2A 56 kDa regulatory subunit beta isoform	100	1.00.E-17
Q15173-2	Isoform Beta-2 of Serine/threonine-protein phosphatase 2A 56 kDa regulatory subunit beta isoform	100	1.00.E-17
O95670-3	Isoform 3 of V-type proton ATPase subunit G 2	100	1.00.E-17
Q9Y653-5	Isoform 5 of Adhesion G-protein coupled receptor G1	100	1.00.E-17
Q9Y653-2	Isoform 2 of Adhesion G-protein coupled receptor G1	100	1.00.E-17
Q9Y653	Adhesion G-protein coupled receptor G1	100	1.00.E-17
Q9Y653-3	Isoform 3 of Adhesion G-protein coupled receptor G1	100	1.00.E-17
O75334	Liprin-alpha-2	100	1.00.E-17
O75334-5	Isoform 5 of Liprin-alpha-2	100	1.00.E-17
O75334-2	Isoform 2 of Liprin-alpha-2	100	1.00.E-17
O75334-3	Isoform 3 of Liprin-alpha-2	100	1.00.E-17
O75334-4	Isoform 4 of Liprin-alpha-2	100	1.00.E-17
A1A5C7	Solute carrier family 22 member 23	100	1.00.E-17
Q8WXA3-4	Isoform 3 of RUN and FYVE domain-containing protein 2	100	1.00.E-17
O75334-6	Isoform 6 of Liprin-alpha-2	100	1.00.E-17
P54803	Galactocerebrosidase	100	1.00.E-17
Q8WXA3-5	Isoform 4 of RUN and FYVE domain-containing protein 2	100	1.00.E-17
O43426-5	Isoform 4 of Synaptojanin-1	100	1.00.E-17
P49441	Inositol polyphosphate 1-phosphatase	100	1.00.E-17
O95297-3	Isoform 3 of Myelin protein zero-like protein 1	100	1.00.E-17
Q8NFZ4	Neuroligin-2	100	1.00.E-17
Q9NWQ8	Phosphoprotein associated with glycosphingolipid-enriched microdomains 1	100	1.00.E-17
Q9NZR2	Low-density lipoprotein receptor-related protein 1B	100	1.00.E-17

P55273	Cyclin-dependent kinase 4 inhibitor D	100	1.00.E-17
Q13114	TNF receptor-associated factor 3	100	1.00.E-17
C9JRZ8	Aldo-keto reductase family 1 member B15	100	1.00.E-17
C9JRZ8-2	Isoform 2 of Aldo-keto reductase family 1 member B15	100	1.00.E-17
P05067-3	Isoform L-APP677 of Amyloid-beta precursor protein	100	1.00.E-17
P05067-10	Isoform APP639 of Amyloid-beta precursor protein	100	1.00.E-17
P05067-4	Isoform APP695 of Amyloid-beta precursor protein	100	1.00.E-17
P43007	Neutral amino acid transporter A	100	1.00.E-17
P43007-2	Isoform 2 of Neutral amino acid transporter A	100	1.00.E-17
Q9H4G0-2	Isoform 2 of Band 4.1-like protein 1	100	1.00.E-17
Q99767-2	Isoform 2 of Amyloid-beta A4 precursor protein-binding family A member 2	100	1.00.E-17
Q99767	Amyloid-beta A4 precursor protein-binding family A member 2	100	1.00.E-17
A1A5C7-4	Isoform 4 of Solute carrier family 22 member 23	100	1.00.E-17
O95479	GDH/6PGL endoplasmic bifunctional protein	100	1.00.E-17
O95479-2	Isoform 2 of GDH/6PGL endoplasmic bifunctional protein	100	1.00.E-17
Q9Y653-4	Isoform 4 of Adhesion G-protein coupled receptor G1	100	1.00.E-17
Q15111	Inactive phospholipase C-like protein 1	100	1.00.E-17
P81274	G-protein-signaling modulator 2	100	1.00.E-17
Q99946	Proline-rich transmembrane protein 1	100	1.00.E-17
Q02153	Guanylate cyclase soluble subunit beta-1	100	1.00.E-17
Q02153-3	Isoform 3 of Guanylate cyclase soluble subunit beta-1	100	1.00.E-17
Q02153-2	Isoform HSGC-2 of Guanylate cyclase soluble subunit beta-1	100	1.00.E-17
P61601	Neurocalcin-delta	100	1.00.E-17
Q96EQ0	Small glutamine-rich tetratricopeptide repeat-containing protein beta	100	1.00.E-17
Q9HCD6-3	Isoform 3 of Protein TANC2	100	1.00.E-17
Q9HCD6-4	Isoform 4 of Protein TANC2	100	1.00.E-17
Q9Y3E7-2	Isoform 2 of Charged multivesicular body protein 3	100	1.00.E-17
Q9Y3E7	Charged multivesicular body protein 3	100	1.00.E-17
Q9Y3E7-4	Isoform 4 of Charged multivesicular body protein 3	100	1.00.E-17
Q9Y3E7-3	Isoform 3 of Charged multivesicular body protein 3	100	1.00.E-17
Q13114-2	Isoform 2 of TNF receptor-associated factor 3	100	1.00.E-17
Q9UKF7	Cytoplasmic phosphatidylinositol transfer protein 1	100	1.00.E-17
Q9UKF7-2	Isoform 2 of Cytoplasmic phosphatidylinositol transfer protein 1	100	1.00.E-17
P46108-2	Isoform Crk-I of Adapter molecule crk	100	1.00.E-17
P04062-2	Isoform Short of Lysosomal acid glucosylceramidase	100	1.00.E-17
P04062	Lysosomal acid glucosylceramidase	100	1.00.E-17
P04062-5	Isoform 5 of Lysosomal acid glucosylceramidase	100	1.00.E-17
P04062-4	Isoform 4 of Lysosomal acid glucosylceramidase	100	1.00.E-17
A1A5C7-2	Isoform 2 of Solute carrier family 22 member 23	100	1.00.E-17
A1A5C7-3	Isoform 3 of Solute carrier family 22 member 23	100	1.00.E-17
P04271	Protein S100-B	100	1.00.E-17
Q9UPW8	Protein unc-13 homolog A	100	1.00.E-17
O14795-2	Isoform 2 of Protein unc-13 homolog B	100	1.00.E-17
Q8NB66	Protein unc-13 homolog C	100	1.00.E-17
O14795	Protein unc-13 homolog B	100	1.00.E-17
Q8N350-4	Isoform 2 of Voltage-dependent calcium channel beta subunit-associated regulatory protein	100	1.00.E-17
Q8N350	Voltage-dependent calcium channel beta subunit-associated regulatory protein	100	1.00.E-17
Q53HC9	EARP and GARP complex-interacting protein 1	100	1.00.E-17
Q8NBJ7-5	Isoform 5 of Inactive C-alpha-formylglycine-generating enzyme 2	100	1.00.E-17
Q9Y4K3	TNF receptor-associated factor 6	100	1.00.E-17
Q9P2G3	Kelch-like protein 14	100	1.00.E-17
Q9P2G3-2	Isoform 2 of Kelch-like protein 14	100	1.00.E-17
P13929-2	Isoform 2 of Beta-enolase	100	1.00.E-17
O14523	Phospholipid transfer protein C2CD2L	100	1.00.E-17
O14523-2	Isoform 2 of Phospholipid transfer protein C2CD2L	100	1.00.E-17
Q9NYQ7-2	Isoform 2 of Cadherin EGF LAG seven-pass G-type receptor 3	100	1.00.E-17
Q9NYQ7	Cadherin EGF LAG seven-pass G-type receptor 3	100	1.00.E-17
Q96DE0-4	Isoform 4 of U8 snoRNA-decapping enzyme	100	1.00.E-17
Q8N2G4-3	Isoform 3 of Ly6/PLAUR domain-containing protein 1	100	1.00.E-17
Q8N2G4	Ly6/PLAUR domain-containing protein 1	100	1.00.E-17
Q8N2G4-4	Isoform 4 of Ly6/PLAUR domain-containing protein 1	100	1.00.E-17
Q8N2G4-2	Isoform 2 of Ly6/PLAUR domain-containing protein 1	100	1.00.E-17
Q9H1C7	Cysteine-rich and transmembrane domain-containing protein 1	100	1.00.E-17
Q8IZR5-2	Isoform 2 of CKLF-like MARVEL transmembrane domain-containing protein 4	100	1.00.E-17
Q8IZR5-3	Isoform 3 of CKLF-like MARVEL transmembrane domain-containing protein 4	100	1.00.E-17
Q8IZR5	CKLF-like MARVEL transmembrane domain-containing protein 4	100	1.00.E-17
Q9NYI0-3	Isoform 3 of PH and SEC7 domain-containing protein 3	100	1.00.E-17
Q9NYI0-2	Isoform 2 of PH and SEC7 domain-containing protein 3	100	1.00.E-17
Q9NYI0	PH and SEC7 domain-containing protein 3	100	1.00.E-17

P0DMP2	SLIT-ROBO Rho GTPase-activating protein 2B	100	1.00.E-17
P0DJJ0	SLIT-ROBO Rho GTPase-activating protein 2C	100	1.00.E-17
Q9UPV7	PHD finger protein 24	100	1.00.E-17
O95630	STAM-binding protein	100	1.00.E-17
Q9BYT3	Serine/threonine-protein kinase 33	100	1.00.E-17
Q8TDW7-2	Isoform 2 of Protocadherin Fat 3	100	1.00.E-17
Q96S06	Lipase maturation factor 1	100	1.00.E-17
Q9Y4C0-3	Isoform 3a of Neurexin-3	100	1.00.E-17
Q9Y4C0-4	Isoform 4a of Neurexin-3	100	1.00.E-17
Q9Y4C0	Neurexin-3	100	1.00.E-17
Q04206-4	Isoform 4 of Transcription factor p65	100	1.00.E-17
Q6P6B1	Glutamate-rich protein 5	100	1.00.E-17
Q8IWE4	DCN1-like protein 3	100	1.00.E-17
O00255-2	Isoform 2 of Menin	100	1.00.E-17
O00255-3	Isoform 3 of Menin	100	1.00.E-17
O00255	Menin	100	1.00.E-17
Q9UPU3	VPS10 domain-containing receptor SorCS3	100	1.00.E-17
Q96HH4	Transmembrane protein 169	100	1.00.E-17
P33897	ATP-binding cassette sub-family D member 1	100	1.00.E-17
Q8N2Q7	Neuroligin-1	100	1.00.E-17
Q8N2Q7-2	Isoform 2 of Neuroligin-1	100	1.00.E-17
Q86TC9-2	Isoform 2 of Myopalladin	100	1.00.E-17
Q86TC9	Myopalladin	100	1.00.E-17
Q5T4F4	Protrudin	100	1.00.E-17
Q5T4F4-8	Isoform 8 of Protrudin	100	1.00.E-17
Q5T4F4-6	Isoform 6 of Protrudin	100	1.00.E-17
Q5T4F4-2	Isoform 2 of Protrudin	100	1.00.E-17
Q5T4F4-5	Isoform 5 of Protrudin	100	1.00.E-17
Q5T4F4-7	Isoform 7 of Protrudin	100	1.00.E-17
Q5T4F4-3	Isoform 3 of Protrudin	100	1.00.E-17
P37059	Estradiol 17-beta-dehydrogenase 2	100	1.00.E-17
Q8WXA3-1	Isoform 5 of RUN and FYVE domain-containing protein 2	100	1.00.E-17
Q8WXA3	RUN and FYVE domain-containing protein 2	100	1.00.E-17
Q8WXA3-3	Isoform 2 of RUN and FYVE domain-containing protein 2	100	1.00.E-17
P39210	Protein Mpv17	100	1.00.E-17
O94813-3	Isoform 3 of Slit homolog 2 protein	100	1.00.E-17
O94813	Slit homolog 2 protein	100	1.00.E-17
O94813-2	Isoform 2 of Slit homolog 2 protein	100	1.00.E-17
P58511	Small integral membrane protein 11A	100	1.00.E-17
Q14511-3	Isoform 3 of Enhancer of filamentation 1	100	1.00.E-17
Q14511	Enhancer of filamentation 1	100	1.00.E-17
Q9GZY8	Mitochondrial fission factor	100	1.00.E-17
Q9GZY8-5	Isoform 5 of Mitochondrial fission factor	100	1.00.E-17
Q9GZY8-2	Isoform 2 of Mitochondrial fission factor	100	1.00.E-17
Q8TCX1-2	Isoform 2 of Cytoplasmic dynein 2 light intermediate chain 1	100	1.00.E-17
Q14693-4	Isoform 4 of Phosphatidate phosphatase LPIN1	100	1.00.E-17
P0CG40	Transcription factor Sp9	100	1.00.E-17
P06239-3	Isoform 3 of Tyrosine-protein kinase Lck	100	1.00.E-17
Q8TCX1	Cytoplasmic dynein 2 light intermediate chain 1	100	1.00.E-17
Q9H1J7	Protein Wnt-5b	100	1.00.E-17
Q9H313-5	Isoform 5 of Protein tweety homolog 1	100	1.00.E-17
Q9H313-2	Isoform 2 of Protein tweety homolog 1	100	1.00.E-17
Q9Y6N8	Cadherin-10	100	1.00.E-17
Q12981-1	Isoform 3 of Vesicle transport protein SEC20	100	1.00.E-17
Q9H840	Gem-associated protein 7	100	1.00.E-17
P34896-2	Isoform 2 of Serine hydroxymethyltransferase, cytosolic	100	1.00.E-17
O76038	Secretagogin	100	1.00.E-17
P14384	Carboxypeptidase M	100	1.00.E-17
Q86W92	Liprin-beta-1	100	1.00.E-17
Q96GX1	Tectonic-2	100	1.00.E-17
Q9UMR5-3	Isoform 3 of Lysosomal thioesterase PPT2	100	1.00.E-17
P12107-2	Isoform B of Collagen alpha-1(XI) chain	100	1.00.E-17
Q9H7X7-2	Isoform 2 of Intraflagellar transport protein 22 homolog	100	1.00.E-17
Q14693-7	Isoform 7 of Phosphatidate phosphatase LPIN1	100	1.00.E-17
Q12981	Vesicle transport protein SEC20	100	1.00.E-17
Q86W92-4	Isoform 4 of Liprin-beta-1	100	1.00.E-17
P06239	Tyrosine-protein kinase Lck	100	1.00.E-17
P21291	Cysteine and glycine-rich protein 1	100	1.00.E-17
P34896-3	Isoform 3 of Serine hydroxymethyltransferase, cytosolic	100	1.00.E-17

Q68CQ7-2	Isoform 2 of Glycosyltransferase 8 domain-containing protein 1	100	1.00.E-17
Q12981-2	Isoform 2 of Vesicle transport protein SEC20	100	1.00.E-17
O15231-6	Isoform 6 of Zinc finger protein 185	100	1.00.E-17
O15231-9	Isoform 9 of Zinc finger protein 185	100	1.00.E-17
P34896-4	Isoform 4 of Serine hydroxymethyltransferase, cytosolic	100	1.00.E-17
Q12981-3	Isoform 4 of Vesicle transport protein SEC20	100	1.00.E-17
P10071	Transcriptional activator GLI3	100	1.00.E-17
O15231-2	Isoform 2 of Zinc finger protein 185	100	1.00.E-17
P82663-2	Isoform 2 of 28S ribosomal protein S25, mitochondrial	100	1.00.E-17
Q8TCX1-3	Isoform 3 of Cytoplasmic dynein 2 light intermediate chain 1	100	1.00.E-17
Q68CQ7	Glycosyltransferase 8 domain-containing protein 1	100	1.00.E-17
Q13474	Dystrophin-related protein 2	100	1.00.E-17
P38936	Cyclin-dependent kinase inhibitor 1	100	1.00.E-17
Q9H313-3	Isoform 3 of Protein tweety homolog 1	100	1.00.E-17
P21980-3	Isoform 3 of Protein-glutamine gamma-glutamyltransferase 2	100	1.00.E-17
Q13474-2	Isoform 2 of Dystrophin-related protein 2	100	1.00.E-17
Q8IXZ3-1	Isoform 1 of Transcription factor Sp8	100	1.00.E-17
Q8IXZ3	Transcription factor Sp8	100	1.00.E-17
P50135	Histamine N-methyltransferase	100	1.00.E-17
Q9UBS9-2	Isoform 2 of SUN domain-containing ossification factor	100	1.00.E-17
Q96GX1-2	Isoform 2 of Tectonic-2	100	1.00.E-17
Q14693-5	Isoform 5 of Phosphatidate phosphatase LPIN1	100	1.00.E-17
P41221-2	Isoform 2 of Protein Wnt-5a	100	1.00.E-17
O15231-8	Isoform 8 of Zinc finger protein 185	100	1.00.E-17
Q86W92-2	Isoform 2 of Liprin-beta-1	100	1.00.E-17
Q9UMR5	Lysosomal thioesterase PPT2	100	1.00.E-17
Q9HCM2	Plexin-A4	100	1.00.E-17
Q86W92-3	Isoform 3 of Liprin-beta-1	100	1.00.E-17
P12107-3	Isoform C of Collagen alpha-1(XI) chain	100	1.00.E-17
Q14693-3	Isoform 3 of Phosphatidate phosphatase LPIN1	100	1.00.E-17
Q9ULB4	Cadherin-9	100	1.00.E-17
Q8IXZ3-4	Isoform 4 of Transcription factor Sp8	100	1.00.E-17
Q14693	Phosphatidate phosphatase LPIN1	100	1.00.E-17
Q9H313	Protein tweety homolog 1	100	1.00.E-17
P49221	Protein-glutamine gamma-glutamyltransferase 4	100	1.00.E-17
P34896	Serine hydroxymethyltransferase, cytosolic	100	1.00.E-17
Q9H7X7	Intraflagellar transport protein 22 homolog	100	1.00.E-17
P41221	Protein Wnt-5a	100	1.00.E-17
Q8IXZ3-2	Isoform 2 of Transcription factor Sp8	100	1.00.E-17
P82663-3	Isoform 3 of 28S ribosomal protein S25, mitochondrial	100	1.00.E-17
Q9UMR5-2	Isoform 2 of Lysosomal thioesterase PPT2	100	1.00.E-17
P12107-4	Isoform 4 of Collagen alpha-1(XI) chain	100	1.00.E-17
Q9UBS9	SUN domain-containing ossification factor	100	1.00.E-17
P12107	Collagen alpha-1(XI) chain	100	1.00.E-17
Q14693-2	Isoform 2 of Phosphatidate phosphatase LPIN1	100	1.00.E-17
O75592	E3 ubiquitin-protein ligase MYCBP2	98.016	1.00.E-17
O75592-2	Isoform 2 of E3 ubiquitin-protein ligase MYCBP2	98.016	1.00.E-17
O75891-3	Isoform 3 of Cytosolic 10-formyltetrahydrofolate dehydrogenase	90.559	1.00.E-17
O75891	Cytosolic 10-formyltetrahydrofolate dehydrogenase	90.559	1.00.E-17
O75891-2	Isoform 2 of Cytosolic 10-formyltetrahydrofolate dehydrogenase	90.559	1.00.E-17
P23763	Vesicle-associated membrane protein 1	65.699	1.09.E-10
P23763-2	Isoform 3 of Vesicle-associated membrane protein 1	65.699	1.09.E-10
P23763-3	Isoform 2 of Vesicle-associated membrane protein 1	65.699	1.09.E-10
Q59EK9-2	Isoform 2 of RUN domain-containing protein 3A	52.515	4.58.E-10
Q59EK9-3	Isoform 3 of RUN domain-containing protein 3A	52.515	4.58.E-10
Q59EK9	RUN domain-containing protein 3A	52.515	4.58.E-10
Q59EK9-4	Isoform 4 of RUN domain-containing protein 3A	52.515	4.58.E-10
P51797-4	Isoform 4 of Chloride transport protein 6	44.057	2.00.E-08
P51797-2	Isoform 2 of Chloride transport protein 6	44.057	2.00.E-08
P51797-3	Isoform 3 of Chloride transport protein 6	44.057	2.00.E-08
Q9Y2B0-2	Isoform 2 of Protein canopy homolog 2	39.72	7.21.E-06
Q06187-2	Isoform BTK-C of Tyrosine-protein kinase BTK	31.615	7.37.E-08
Q06187	Tyrosine-protein kinase BTK	31.615	7.37.E-08
Q9NTI5-5	Isoform 5 of Sister chromatid cohesion protein PDS5 homolog B	30.843	4.76.E-10
P40939-2	Isoform 2 of Trifunctional enzyme subunit alpha, mitochondrial	29.283	3.48.E-06
P04083	Annexin A1	26.311	1.00.E-17
Q9Y625	Glypican-6	25.004	9.65.E-09
Q9NQC3-2	Isoform B of Reticulon-4	21.377	1.08.E-06
P00918	Carbonic anhydrase 2	20.562	2.23.E-13

Q9UIG0	Tyrosine-protein kinase BAZ1B	18.478	1.61.E-03
Q9UIG0-2	Isoform 2 of Tyrosine-protein kinase BAZ1B	18.478	1.61.E-03
Q96HF1	Secreted frizzled-related protein 2	18.385	2.36.E-09
P12036	Neurofilament heavy polypeptide	18.225	1.73.E-14
P12036-2	Isoform 2 of Neurofilament heavy polypeptide	18.225	1.73.E-14
P09455	Retinol-binding protein 1	16.527	1.77.E-07
Q5T4D3-4	Isoform 4 of Protein O-mannosyl-transferase TMTC4	16.512	2.55.E-04
Q5T4D3	Protein O-mannosyl-transferase TMTC4	16.512	2.55.E-04
Q5T4D3-2	Isoform 2 of Protein O-mannosyl-transferase TMTC4	16.512	2.55.E-04
Q5T4D3-3	Isoform 3 of Protein O-mannosyl-transferase TMTC4	16.512	2.55.E-04
P09455-2	Isoform 2 of Retinol-binding protein 1	16.025	1.29.E-07
P09455-3	Isoform 3 of Retinol-binding protein 1	16.025	1.29.E-07
O15540	Fatty acid-binding protein, brain	15.392	2.30.E-08
O15540-2	Isoform 2 of Fatty acid-binding protein, brain	15.392	1.92.E-08
P02511	Alpha-crystallin B chain	14.513	3.06.E-08
P08670	Vimentin	13.49	3.75.E-12
P00352	Retinal dehydrogenase 1	13.322	8.05.E-11
Q8TDW7	Protocadherin Fat 3	13.076	5.21.E-04
Q8TDW7-1	Isoform 3 of Protocadherin Fat 3	13.076	5.21.E-04
Q9BRT3	Migration and invasion enhancer 1	12.441	6.86.E-05
P35080	Profilin-2	12.275	8.08.E-05
P29317	Ephrin type-A receptor 2	12.059	4.89.E-06
Q9NR96-4	Isoform 4 of Toll-like receptor 9	11.912	4.38.E-06
Q9NRX4-2	Isoform 2 of 14 kDa phosphohistidine phosphatase	11.877	5.07.E-06
Q9NRX4	14 kDa phosphohistidine phosphatase	11.877	5.07.E-06
O94875-3	Isoform 3 of Sorbin and SH3 domain-containing protein 2	11.653	6.61.E-04
O94875-4	Isoform 4 of Sorbin and SH3 domain-containing protein 2	11.653	6.61.E-04
O94875	Sorbin and SH3 domain-containing protein 2	11.653	6.61.E-04
O94875-2	Isoform 2 of Sorbin and SH3 domain-containing protein 2	11.653	6.61.E-04
O94875-7	Isoform 7 of Sorbin and SH3 domain-containing protein 2	11.653	6.61.E-04
O94875-10	Isoform 10 of Sorbin and SH3 domain-containing protein 2	11.653	6.61.E-04
O94875-12	Isoform 12 of Sorbin and SH3 domain-containing protein 2	11.653	6.61.E-04
O94875-5	Isoform 5 of Sorbin and SH3 domain-containing protein 2	11.653	6.61.E-04
O94875-9	Isoform 9 of Sorbin and SH3 domain-containing protein 2	11.653	6.61.E-04
O94875-8	Isoform 8 of Sorbin and SH3 domain-containing protein 2	11.653	6.61.E-04
O94875-11	Isoform 11 of Sorbin and SH3 domain-containing protein 2	11.653	6.61.E-04
P27701-2	Isoform 2 of CD82 antigen	11.396	1.28.E-04
P27701	CD82 antigen	11.396	1.28.E-04
Q8WX93-7	Isoform 7 of Palladin	11.348	2.15.E-06
P80370	Protein delta homolog 1	11.333	3.78.E-04
Q8TBF2-3	Isoform 3 of Prostamide/prostaglandin F synthase	11.181	7.47.E-03
Q8TBF2-7	Isoform 6 of Prostamide/prostaglandin F synthase	11.181	7.47.E-03
Q8TBF2-6	Isoform 5 of Prostamide/prostaglandin F synthase	11.181	7.47.E-03
Q8TBF2	Prostamide/prostaglandin F synthase	11.181	7.47.E-03
Q8TBF2-4	Isoform 4 of Prostamide/prostaglandin F synthase	11.181	7.47.E-03
Q8TBF2-2	Isoform 2 of Prostamide/prostaglandin F synthase	11.181	7.47.E-03
P56557	Transmembrane protein 50B	11.14	6.48.E-03
P05937	Calbindin	10.883	4.53.E-08
P21266	Glutathione S-transferase Mu 3	10.545	4.45.E-06
P17661	Desmin	10.454	2.40.E-10
Q5IJ48-2	Isoform 2 of Protein crumbs homolog 2	10.378	4.53.E-06
P56199	Integrin alpha-1	10.323	1.71.E-03
P05937-2	Isoform 2 of Calbindin	10.269	1.32.E-07
P14618-2	Isoform M1 of Pyruvate kinase PKM	10.087	2.25.E-05
P07203-2	Isoform 2 of Glutathione peroxidase 1	9.994	2.85.E-04
P11137-2	Isoform 2 of Microtubule-associated protein 2	9.94	8.43.E-06
Q9Y2Y0-2	Isoform 2 of ADP-ribosylation factor-like protein 2-binding protein	9.927	3.32.E-04
Q9Y2Y0	ADP-ribosylation factor-like protein 2-binding protein	9.927	3.32.E-04
Q9P0M6	Core histone macro-H2A.2	9.399	3.95.E-03
P98172	Ephrin-B1	9.336	3.00.E-05
P80370-2	Isoform Short of Protein delta homolog 1	9.18	1.59.E-04
Q96SJ8	Tetraspanin-18	9.003	4.58.E-03
P62745	Rho-related GTP-binding protein RhoB	8.951	2.04.E-03
Q5IJ48	Protein crumbs homolog 2	8.939	5.26.E-06
Q9UKG9-3	Isoform 3 of Peroxisomal carnitine O-octanoyltransferase	8.776	8.59.E-03
Q9UKG9	Peroxisomal carnitine O-octanoyltransferase	8.776	8.59.E-03
P53801	Pituitary tumor-transforming gene 1 protein-interacting protein	8.749	6.50.E-03
Q8WX93-8	Isoform 8 of Palladin	8.711	2.95.E-05
Q8WX93-3	Isoform 3 of Palladin	8.711	2.95.E-05

Q8WX93-2	Isoform 2 of Palladin	8.711	6.17.E-05
Q8WX93-4	Isoform 4 of Palladin	8.711	2.95.E-05
Q8WX93-5	Isoform 5 of Palladin	8.711	2.95.E-05
Q8WX93	Palladin	8.711	2.95.E-05
P35556-2	Isoform 2 of Fibrillin-2	8.623	1.57.E-02
O95834	Echinoderm microtubule-associated protein-like 2	8.443	4.19.E-03
O95834-3	Isoform 3 of Echinoderm microtubule-associated protein-like 2	8.443	4.19.E-03
O95834-2	Isoform 2 of Echinoderm microtubule-associated protein-like 2	8.443	4.19.E-03
O43790	Keratin, type II cuticular Hb6	8.432	6.02.E-09
P78386	Keratin, type II cuticular Hb5	8.432	6.02.E-09
Q8N1N4-2	Isoform 2 of Keratin, type II cytoskeletal 78	8.432	6.02.E-09
Q14533	Keratin, type II cuticular Hb1	8.432	6.02.E-09
Q8N1N4	Keratin, type II cytoskeletal 78	8.432	6.02.E-09
P78385	Keratin, type II cuticular Hb3	8.432	6.02.E-09
P35556	Fibrillin-2	8.25	6.48.E-03
Q8IYB5-2	Isoform 2 of Stromal membrane-associated protein 1	8.246	3.06.E-03
Q8IYB5	Stromal membrane-associated protein 1	8.246	3.06.E-03
Q8IYB5-3	Isoform 3 of Stromal membrane-associated protein 1	8.246	3.06.E-03
Q8WX93-9	Isoform 9 of Palladin	8.156	5.58.E-05
P17655	Calpain-2 catalytic subunit	8.06	2.70.E-07
P29317-2	Isoform 2 of Ephrin type-A receptor 2	8.038	5.00.E-04
P22676	Calretinin	7.995	1.02.E-04
P55285	Cadherin-6	7.989	1.99.E-03
P17655-2	Isoform 2 of Calpain-2 catalytic subunit	7.973	9.44.E-07
Q6DN90-3	Isoform 3 of IQ motif and SEC7 domain-containing protein 1	7.963	1.47.E-03
Q6DN90-2	Isoform 2 of IQ motif and SEC7 domain-containing protein 1	7.963	1.47.E-03
Q6DN90	IQ motif and SEC7 domain-containing protein 1	7.963	1.47.E-03
P17677-2	Isoform 2 of Neuromodulin	7.823	7.65.E-05
P17677	Neuromodulin	7.823	7.65.E-05
P13591	Neural cell adhesion molecule 1	7.747	1.66.E-06
P13591-1	Isoform 2 of Neural cell adhesion molecule 1	7.747	1.88.E-06
Q0VDG4-2	Isoform 2 of Secernin-3	7.741	2.29.E-02
Q0VDG4	Secernin-3	7.741	2.29.E-02
Q9NU23	LYR motif-containing protein 2	7.568	1.49.E-02
Q9P232	Contactin-3	7.512	1.26.E-03
P84157-2	Isoform 2 of Matrix-remodeling-associated protein 7	7.499	1.19.E-03
P84157	Matrix-remodeling-associated protein 7	7.499	1.19.E-03
P84157-3	Isoform 3 of Matrix-remodeling-associated protein 7	7.499	1.19.E-03
Q9Y2S2-2	Isoform 2 of Lambda-crystallin homolog	7.383	2.09.E-02
Q9Y2S2	Lambda-crystallin homolog	7.383	2.09.E-02
P55285-2	Isoform 2 of Cadherin-6	7.345	1.41.E-03
Q6PIU2-3	Isoform 3 of Neutral cholesterol ester hydrolase 1	7.273	5.26.E-03
Q9UIJ7-3	Isoform 3 of GTP:AMP phosphotransferase AK3, mitochondrial	7.228	1.66.E-04
Q9UIJ7	GTP:AMP phosphotransferase AK3, mitochondrial	7.228	1.35.E-04
P13591-4	Isoform 4 of Neural cell adhesion molecule 1	7.208	3.97.E-06
P13591-3	Isoform 3 of Neural cell adhesion molecule 1	7.208	3.89.E-06
Q96NY7	Chloride intracellular channel protein 6	7.198	1.10.E-04
Q96NY7-2	Isoform A of Chloride intracellular channel protein 6	7.198	1.10.E-04
P63267-2	Isoform 2 of Actin, gamma-enteric smooth muscle	7.125	2.71.E-06
Q96FC7-2	Isoform 2 of Phytanoyl-CoA hydroxylase-interacting protein-like	7.113	2.93.E-02
P06396-4	Isoform 4 of Gelsolin	7.103	7.01.E-06
P06396	Gelsolin	7.103	7.01.E-06
P06396-2	Isoform 2 of Gelsolin	7.103	7.01.E-06
P06396-3	Isoform 3 of Gelsolin	7.103	7.01.E-06
Q96KR6	Protein FAM210B, mitochondrial	6.954	9.29.E-03
Q75LS8	Putative FK506-binding protein 9-like protein	6.907	1.28.E-02
O60826	Coiled-coil domain-containing protein 22	6.889	8.89.E-04
Q9NZM1-3	Isoform 3 of Myoferlin	6.855	5.19.E-05
O14763-3	Isoform 3 of Tumor necrosis factor receptor superfamily member 10B	6.788	1.25.E-02
Q9UBI1	COMM domain-containing protein 3	6.73	1.29.E-02
Q68D91	Metallo-beta-lactamase domain-containing protein 2	6.68	2.72.E-06
Q68D91-2	Isoform 2 of Metallo-beta-lactamase domain-containing protein 2	6.68	2.72.E-06
P07996	Thrombospondin-1	6.567	1.45.E-03
P07996-2	Isoform 2 of Thrombospondin-1	6.567	1.45.E-03
Q15417-2	Isoform 2 of Calponin-3	6.49	7.74.E-05
Q6PIU2	Neutral cholesterol ester hydrolase 1	6.485	5.86.E-03
Q6PIU2-2	Isoform 2 of Neutral cholesterol ester hydrolase 1	6.485	5.86.E-03
O95302-2	Isoform 2 of Peptidyl-prolyl cis-trans isomerase FKBP9	6.464	1.04.E-02
O75367	Core histone macro-H2A.1	6.442	2.64.E-03

O75367-3	Isoform 3 of Core histone macro-H2A.1	6.442	2.64.E-03
O75367-2	Isoform 1 of Core histone macro-H2A.1	6.442	2.64.E-03
Q9Y281	Cofilin-2	6.424	1.73.E-04
Q9Y281-3	Isoform 3 of Cofilin-2	6.424	1.73.E-04
P51649-2	Isoform 2 of Succinate-semialdehyde dehydrogenase, mitochondrial	6.422	2.43.E-04
P51649	Succinate-semialdehyde dehydrogenase, mitochondrial	6.422	2.43.E-04
Q13683	Integrin alpha-7	6.341	3.35.E-03
Q13683-9	Isoform Alpha-7X2DB of Integrin alpha-7	6.341	3.35.E-03
Q13683-10	Isoform Alpha-7X1X2A of Integrin alpha-7	6.341	3.35.E-03
Q13683-7	Isoform Alpha-7X2B of Integrin alpha-7	6.341	3.35.E-03
Q13683-3	Isoform Alpha-7X1B of Integrin alpha-7	6.341	3.35.E-03
P98164	Low-density lipoprotein receptor-related protein 2	6.336	3.17.E-03
Q8WWN8-2	Isoform 2 of Arf-GAP with Rho-GAP domain, ANK repeat and PH domain-containing protein 3	6.328	2.22.E-03
Q8WWN8	Arf-GAP with Rho-GAP domain, ANK repeat and PH domain-containing protein 3	6.328	2.22.E-03
Q13443	Disintegrin and metalloproteinase domain-containing protein 9	6.315	1.13.E-02
Q13443-2	Isoform 2 of Disintegrin and metalloproteinase domain-containing protein 9	6.315	1.13.E-02
Q9UBG0	C-type mannose receptor 2	6.289	1.15.E-04
Q13557-10	Isoform Delta 10 of Calcium/calmodulin-dependent protein kinase type II subunit delta	6.27	1.62.E-05
Q13557-6	Isoform Delta 9 of Calcium/calmodulin-dependent protein kinase type II subunit delta	6.27	1.62.E-05
Q13557-11	Isoform Delta 11 of Calcium/calmodulin-dependent protein kinase type II subunit delta	6.27	1.61.E-05
Q13557	Calcium/calmodulin-dependent protein kinase type II subunit delta	6.27	1.61.E-05
Q13557-12	Isoform Delta 12 of Calcium/calmodulin-dependent protein kinase type II subunit delta	6.27	1.61.E-05
Q13557-8	Isoform Delta 6 of Calcium/calmodulin-dependent protein kinase type II subunit delta	6.27	1.61.E-05
Q9UI15	Transgelin-3	6.243	2.77.E-04
P13929-3	Isoform 3 of Beta-enolase	6.243	9.40.E-03
P13929	Beta-enolase	6.243	9.40.E-03
Q8TAT2	Fibroblast growth factor-binding protein 3	6.205	1.21.E-02
Q96GA7	Serine dehydratase-like	6.194	6.47.E-03
P68133	Actin, alpha skeletal muscle	6.157	4.20.E-07
Q7Z7K6-2	Isoform 2 of Centromere protein V	6.146	7.77.E-03
Q9UPT6	C-Jun-amino-terminal kinase-interacting protein 3	6.125	2.38.E-03
P06756-2	Isoform 2 of Integrin alpha-V	6.12	1.32.E-05
Q9Y4F1-3	Isoform 3 of FERM, ARHGEF and pleckstrin domain-containing protein 1	6.106	3.84.E-03
P84074	Neuron-specific calcium-binding protein hippocalcin	6.076	8.16.E-03
O95757	Heat shock 70 kDa protein 4L	6.01	3.93.E-05
P06756-3	Isoform 3 of Integrin alpha-V	5.995	1.84.E-05
P06756	Integrin alpha-V	5.995	1.84.E-05
P13591-6	Isoform 6 of Neural cell adhesion molecule 1	5.99	2.90.E-04
O60831	PRA1 family protein 2	5.954	7.98.E-04
P26006-1	Isoform 2 of Integrin alpha-3	5.948	2.08.E-02
P26006	Integrin alpha-3	5.948	2.08.E-02
P02787	Serotransferrin	5.938	9.13.E-04
P09211	Glutathione S-transferase P	5.861	7.75.E-07
P19022	Cadherin-2	5.859	5.82.E-07
P19022-2	Isoform 2 of Cadherin-2	5.859	5.82.E-07
Q5SSJ5-2	Isoform 2 of Heterochromatin protein 1-binding protein 3	5.852	2.94.E-04
Q5SSJ5-3	Isoform 3 of Heterochromatin protein 1-binding protein 3	5.852	3.34.E-04
O14763-2	Isoform Short of Tumor necrosis factor receptor superfamily member 10B	5.837	4.77.E-04
O14763	Tumor necrosis factor receptor superfamily member 10B	5.837	4.77.E-04
Q13557-4	Isoform Delta 4 of Calcium/calmodulin-dependent protein kinase type II subunit delta	5.815	6.58.E-05
Q13557-3	Isoform Delta 3 of Calcium/calmodulin-dependent protein kinase type II subunit delta	5.815	6.58.E-05
Q13557-9	Isoform Delta 7 of Calcium/calmodulin-dependent protein kinase type II subunit delta	5.815	6.58.E-05
Q13557-5	Isoform Delta 8 of Calcium/calmodulin-dependent protein kinase type II subunit delta	5.815	6.58.E-05
P62736	Actin, aortic smooth muscle	5.807	8.69.E-07
P68032	Actin, alpha cardiac muscle 1	5.807	8.69.E-07
Q9Y4J8-7	Isoform 7 of Dystrobrevin alpha	5.796	8.62.E-03
Q9Y4J8-9	Isoform 9 of Dystrobrevin alpha	5.796	8.62.E-03
P63267	Actin, gamma-enteric smooth muscle	5.786	9.09.E-07
Q8NI22-3	Isoform 3 of Multiple coagulation factor deficiency protein 2	5.786	3.90.E-04
Q8NI22	Multiple coagulation factor deficiency protein 2	5.786	3.90.E-04
Q8NI22-2	Isoform 2 of Multiple coagulation factor deficiency protein 2	5.786	3.90.E-04
P07858	Cathepsin B	5.77	1.64.E-03
P46459-2	Isoform 2 of Vesicle-fusing ATPase	5.767	3.68.E-02
O15439-4	Isoform 4 of Multidrug resistance-associated protein 4	5.707	9.69.E-03
O15439-3	Isoform 3 of Multidrug resistance-associated protein 4	5.707	9.69.E-03
Q9BPU6	Dihydropyrimidinase-related protein 5	5.681	4.80.E-06
P13591-5	Isoform 5 of Neural cell adhesion molecule 1	5.67	1.58.E-04
Q9UHQ4-2	Isoform 2 of B-cell receptor-associated protein 29	5.622	2.30.E-02
Q9UHQ4	B-cell receptor-associated protein 29	5.622	2.30.E-02

Q9NZM1-5	Isoform 5 of Myoferlin	5.616	2.72.E-04
Q86SK9-2	Isoform 2 of Stearoyl-CoA desaturase 5	5.605	9.23.E-03
Q86SK9	Stearoyl-CoA desaturase 5	5.605	9.23.E-03
Q9Y548	Protein YIPF1	5.576	2.23.E-02
P54819-4	Isoform 4 of Adenylate kinase 2, mitochondrial	5.454	1.20.E-02
P54819-6	Isoform 6 of Adenylate kinase 2, mitochondrial	5.454	1.20.E-02
P35080-2	Isoform IIb of Profilin-2	5.449	7.27.E-05
Q14332	Frizzled-2	5.437	1.68.E-02
Q13683-13	Isoform 2 of Integrin alpha-7	5.431	8.42.E-03
O94830	Phospholipase DDHD2	5.415	1.57.E-02
O94830-2	Isoform 2 of Phospholipase DDHD2	5.415	1.57.E-02
O00469-3	Isoform 3 of Procollagen-lysine,2-oxoglutarate 5-dioxygenase 2	5.371	2.81.E-02
Q5SSJ5	Heterochromatin protein 1-binding protein 3	5.365	5.25.E-04
P37235	Hippocalcin-like protein 1	5.357	7.21.E-03
O76041-2	Isoform 2 of Nebulette	5.328	6.79.E-04
Q9H1I8-3	Isoform 3 of Activating signal cointegrator 1 complex subunit 2	5.326	6.42.E-03
O95302-3	Isoform 3 of Peptidyl-prolyl cis-trans isomerase FKBP9	5.279	1.58.E-02
O95302	Peptidyl-prolyl cis-trans isomerase FKBP9	5.279	1.58.E-02
P07311	Acylphosphatase-1	5.274	6.96.E-03
Q9C0B1-4	Isoform 4 of Alpha-ketoglutarate-dependent dioxygenase FTO	5.268	2.97.E-02
Q7L775	EPM2A-interacting protein 1	5.241	6.96.E-03
P80404	4-aminobutyrate aminotransferase, mitochondrial	5.228	2.51.E-03
Q16352	Alpha-internexin	5.208	8.30.E-04
P40123-3	Isoform 3 of Adenylyl cyclase-associated protein 2	5.048	1.25.E-03
Q9UQB8-6	Isoform 6 of Brain-specific angiogenesis inhibitor 1-associated protein 2	5.022	1.45.E-02
Q9UQB8-4	Isoform 4 of Brain-specific angiogenesis inhibitor 1-associated protein 2	5.022	1.45.E-02
Q9UQB8-2	Isoform 2 of Brain-specific angiogenesis inhibitor 1-associated protein 2	5.022	1.45.E-02
Q9UQB8	Brain-specific angiogenesis inhibitor 1-associated protein 2	5.022	1.45.E-02
Q9UQB8-3	Isoform 3 of Brain-specific angiogenesis inhibitor 1-associated protein 2	5.022	1.45.E-02
Q9UQB8-5	Isoform 5 of Brain-specific angiogenesis inhibitor 1-associated protein 2	5.022	1.45.E-02
P14415	Sodium/potassium-transporting ATPase subunit beta-2	5.007	3.60.E-02
Q12860-3	Isoform 3 of Contactin-1	4.996	1.62.E-02
P07197-2	Isoform 2 of Neurofilament medium polypeptide	4.975	5.43.E-06
P00813	Adenosine deaminase	4.94	1.59.E-02
Q8NEU8-2	Isoform 2 of DCC-interacting protein 13-beta	4.928	6.71.E-03
Q01995	Transgelin	4.91	6.31.E-06
O43347	RNA-binding protein Musashi homolog 1	4.902	4.40.E-05
P40123-2	Isoform 2 of Adenylyl cyclase-associated protein 2	4.895	2.64.E-03
O14967	Calmegin	4.881	7.61.E-04
P52943	Cysteine-rich protein 2	4.87	1.68.E-02
P52943-2	Isoform 2 of Cysteine-rich protein 2	4.87	1.02.E-02
Q96KN1	Protein LRATD2	4.865	3.73.E-03
Q8NEU8	DCC-interacting protein 13-beta	4.792	1.43.E-02
Q8IVS2	Malonyl-CoA-acyl carrier protein transacylase, mitochondrial	4.76	1.50.E-02
O15439-2	Isoform 2 of Multidrug resistance-associated protein 4	4.759	1.51.E-02
O15439	Multidrug resistance-associated protein 4	4.759	1.37.E-02
Q9Y2H6	Fibronectin type-III domain-containing protein 3A	4.699	1.61.E-03
Q9Y2H6-2	Isoform 2 of Fibronectin type-III domain-containing protein 3A	4.699	1.61.E-03
P34903	Gamma-aminobutyric acid receptor subunit alpha-3	4.692	4.37.E-02
Q8IV38	Ankyrin repeat and MYND domain-containing protein 2	4.661	2.28.E-04
Q8NEU8-3	Isoform 3 of DCC-interacting protein 13-beta	4.632	7.90.E-03
O76041	Nebulette	4.601	1.09.E-02
Q5IJ48-3	Isoform 3 of Protein crumbs homolog 2	4.592	1.04.E-03
O43852-5	Isoform 5 of Calumenin	4.539	1.51.E-05
P40123	Adenylyl cyclase-associated protein 2	4.523	3.74.E-03
Q7Z7K6-3	Isoform 3 of Centromere protein V	4.522	1.56.E-02
Q5VVQ6	Ubiquitin thioesterase OTU1	4.507	4.96.E-02
Q5VVQ6-2	Isoform 2 of Ubiquitin thioesterase OTU1	4.507	4.96.E-02
O43852-11	Isoform 11 of Calumenin	4.503	1.65.E-05
O43852-13	Isoform 13 of Calumenin	4.503	1.65.E-05
O43852-14	Isoform 14 of Calumenin	4.503	1.65.E-05
Q969Q5	Ras-related protein Rab-24	4.503	4.12.E-02
Q14108-2	Isoform 2 of Lysosome membrane protein 2	4.493	4.01.E-04
Q6NTF9	Rhomboid domain-containing protein 2	4.482	9.95.E-03
Q6NTF9-2	Isoform 2 of Rhomboid domain-containing protein 2	4.482	9.95.E-03
Q9NZM1-7	Isoform 7 of Myoferlin	4.462	2.31.E-03
Q9Y223-3	Isoform 3 of Bifunctional UDP-N-acetylglucosamine 2-epimerase/N-acetylmannosamine kinase	4.453	3.18.E-02
Q9Y223-5	Isoform 5 of Bifunctional UDP-N-acetylglucosamine 2-epimerase/N-acetylmannosamine kinase	4.453	3.18.E-02
Q9Y243	RAC-gamma serine/threonine-protein kinase	4.446	2.49.E-02

Q9Y243-2	Isoform 2 of RAC-gamma serine/threonine-protein kinase	4.446	2.49.E-02
Q9BVC6	Transmembrane protein 109	4.443	4.89.E-03
Q9BV23	Monoacylglycerol lipase ABHD6	4.433	4.35.E-02
Q5SQI0-5	Isoform 5 of Alpha-tubulin N-acetyltransferase 1	4.413	2.30.E-02
Q5SQI0-4	Isoform 4 of Alpha-tubulin N-acetyltransferase 1	4.413	2.30.E-02
Q5SQI0	Alpha-tubulin N-acetyltransferase 1	4.413	2.30.E-02
Q5SQI0-2	Isoform 2 of Alpha-tubulin N-acetyltransferase 1	4.413	2.30.E-02
Q5SQI0-3	Isoform 3 of Alpha-tubulin N-acetyltransferase 1	4.413	2.62.E-02
Q5SQI0-7	Isoform 7 of Alpha-tubulin N-acetyltransferase 1	4.413	2.62.E-02
Q5SQI0-6	Isoform 6 of Alpha-tubulin N-acetyltransferase 1	4.413	2.62.E-02
Q9UIJ7-2	Isoform 2 of GTP:AMP phosphotransferase AK3, mitochondrial	4.404	1.25.E-02
Q16799-3	Isoform RTN1-C of Reticulon-1	4.374	2.07.E-02
Q6ZVM7	TOM1-like protein 2	4.34	2.17.E-02
Q6ZVM7-5	Isoform 5 of TOM1-like protein 2	4.34	3.61.E-02
Q8N9N7	Leucine-rich repeat-containing protein 57	4.304	1.60.E-02
P48681	Nestin	4.264	2.96.E-05
Q08499-6	Isoform 5 of cAMP-specific 3',5'-cyclic phosphodiesterase 4D	4.246	4.88.E-02
P12081	Histidine--tRNA ligase, cytoplasmic	4.235	1.86.E-02
P12081-4	Isoform 4 of Histidine--tRNA ligase, cytoplasmic	4.235	1.86.E-02
O43852-9	Isoform 9 of Calumenin	4.233	3.19.E-05
Q96KB5	Lymphokine-activated killer T-cell-originated protein kinase	4.232	3.06.E-03
Q96KB5-2	Isoform 2 of Lymphokine-activated killer T-cell-originated protein kinase	4.232	3.73.E-03
O75051	Plexin-A2	4.23	3.10.E-02
O43852-6	Isoform 6 of Calumenin	4.214	3.35.E-05
Q9UBH6-2	Isoform 2 of Xenotropic and polytropic retrovirus receptor 1	4.132	3.20.E-02
P55283	Cadherin-4	4.122	1.63.E-02
P55283-2	Isoform 2 of Cadherin-4	4.122	1.63.E-02
Q8WUX2	Glutathione-specific gamma-glutamylcyclotransferase 2	4.085	4.41.E-02
O60462-2	Isoform A0 of Neuropilin-2	4.081	2.06.E-02
O60462	Neuropilin-2	4.081	2.06.E-02
O60462-3	Isoform A17 of Neuropilin-2	4.081	2.06.E-02
O60462-5	Isoform B5 of Neuropilin-2	4.081	2.13.E-02
O60462-4	Isoform B0 of Neuropilin-2	4.081	2.13.E-02
Q9NRN7-2	Isoform 2 of L-aminoadipate-semialdehyde dehydrogenase-phosphopantetheinyl transferase	4.076	1.89.E-02
Q04727-4	Isoform 4 of Transducin-like enhancer protein 4	4.06	3.48.E-02
Q04727-3	Isoform 3 of Transducin-like enhancer protein 4	4.06	3.48.E-02
Q04727-2	Isoform 2 of Transducin-like enhancer protein 4	4.06	3.48.E-02
Q04727	Transducin-like enhancer protein 4	4.06	3.48.E-02
Q8NEY8-5	Isoform 5 of Periphilin-1	4.059	2.73.E-02
Q08722-3	Isoform OA3-305 of Leukocyte surface antigen CD47	4.028	3.96.E-03
Q08722-4	Isoform OA3-312 of Leukocyte surface antigen CD47	4.028	3.96.E-03
Q08722-2	Isoform OA3-293 of Leukocyte surface antigen CD47	4.028	3.96.E-03
O43852-3	Isoform 3 of Calumenin	3.98	6.06.E-05
O43852	Calumenin	3.98	6.06.E-05
P11137	Microtubule-associated protein 2	3.973	7.56.E-04
P11137-3	Isoform 3 of Microtubule-associated protein 2	3.973	7.56.E-04
Q9C0E8-4	Isoform 4 of Endoplasmic reticulum junction formation protein lunapark	3.953	5.50.E-03
Q9C0E8	Endoplasmic reticulum junction formation protein lunapark	3.953	5.50.E-03
Q9C0E8-2	Isoform 2 of Endoplasmic reticulum junction formation protein lunapark	3.953	5.50.E-03
Q14315-2	Isoform 2 of Filamin-C	3.941	6.69.E-05
Q14315	Filamin-C	3.926	6.96.E-05
O43852-10	Isoform 10 of Calumenin	3.924	6.98.E-05
Q14108	Lysosome membrane protein 2	3.921	3.99.E-04
Q9UBH6	Xenotropic and polytropic retrovirus receptor 1	3.911	3.30.E-02
Q9NRZ5-2	Isoform 2 of 1-acyl-sn-glycerol-3-phosphate acyltransferase delta	3.872	6.81.E-03
O00562	Membrane-associated phosphatidylinositol transfer protein 1	3.8	4.37.E-02
O00562-2	Isoform 2 of Membrane-associated phosphatidylinositol transfer protein 1	3.8	4.37.E-02
O94919	Endonuclease domain-containing 1 protein	3.793	3.10.E-02
Q96JJ7-2	Isoform 2 of Protein disulfide-isomerase TMX3	3.793	2.38.E-02
O43852-12	Isoform 12 of Calumenin	3.78	2.66.E-05
Q15121-2	Isoform 2 of Astrocytic phosphoprotein PEA-15	3.757	6.50.E-04
Q15121	Astrocytic phosphoprotein PEA-15	3.757	6.50.E-04
Q9Y6Y0	Influenza virus NS1A-binding protein	3.755	7.19.E-04
Q92522	Histone H1.10	3.745	6.46.E-04
P12081-3	Isoform 3 of Histidine--tRNA ligase, cytoplasmic	3.723	1.93.E-02
P12081-2	Isoform 2 of Histidine--tRNA ligase, cytoplasmic	3.723	1.93.E-02
Q9NRZ5	1-acyl-sn-glycerol-3-phosphate acyltransferase delta	3.689	7.67.E-03
Q8NEY1-5	Isoform 5 of Neuron navigator 1	3.664	1.51.E-02
Q9NUJ1-3	Isoform 3 of Mycophenolic acid acyl-glucuronide esterase, mitochondrial	3.649	3.18.E-02

Q9BY43	Charged multivesicular body protein 4a	3.636	5.45.E-03
Q9BY43-2	Isoform 2 of Charged multivesicular body protein 4a	3.636	5.45.E-03
Q08722	Leukocyte surface antigen CD47	3.625	4.93.E-03
Q9NUJ1-2	Isoform 2 of Mycophenolic acid acyl-glucuronide esterase, mitochondrial	3.623	5.93.E-04
Q9UK08	Guanine nucleotide-binding protein G(I)/G(S)/G(O) subunit gamma-8	3.615	1.39.E-02
Q8NBX0	Saccharopine dehydrogenase-like oxidoreductase	3.602	8.75.E-03
Q7LG56-5	Isoform 5 of Ribonucleoside-diphosphate reductase subunit M2 B	3.6	5.77.E-03
O14639	Actin-binding LIM protein 1	3.582	4.21.E-02
O14639-2	Isoform 2 of Actin-binding LIM protein 1	3.582	4.21.E-02
O14639-6	Isoform 6 of Actin-binding LIM protein 1	3.582	4.21.E-02
O14639-5	Isoform 5 of Actin-binding LIM protein 1	3.582	4.62.E-02
O14639-3	Isoform 3 of Actin-binding LIM protein 1	3.582	4.62.E-02
O14639-4	Isoform 4 of Actin-binding LIM protein 1	3.582	4.62.E-02
P35237	Serpin B6	3.557	4.81.E-03
Q9NUJ1	Mycophenolic acid acyl-glucuronide esterase, mitochondrial	3.557	7.31.E-04
O95881	Thioredoxin domain-containing protein 12	3.543	1.97.E-03
P09486	SPARC	3.543	6.82.E-03
Q7LGA3-3	Isoform 3 of Heparan sulfate 2-O-sulfotransferase 1	3.514	3.05.E-02
Q7LGA3-2	Isoform 2 of Heparan sulfate 2-O-sulfotransferase 1	3.514	3.05.E-02
Q08623-2	Isoform 2 of Pseudouridine-5'-phosphatase	3.504	7.46.E-03
P08648	Integrin alpha-5	3.499	1.20.E-04
Q8NEY1-3	Isoform 3 of Neuron navigator 1	3.496	1.11.E-02
Q8NEY1	Neuron navigator 1	3.496	1.11.E-02
Q8NEY1-7	Isoform 7 of Neuron navigator 1	3.496	1.11.E-02
Q8NEY1-2	Isoform 2 of Neuron navigator 1	3.496	1.11.E-02
Q8NEY1-4	Isoform 4 of Neuron navigator 1	3.496	1.11.E-02
A6NMY6	Putative annexin A2-like protein	3.481	2.26.E-04
Q9UPT6-2	Isoform 2 of C-Jun-amino-terminal kinase-interacting protein 3	3.479	4.79.E-02
Q8N6N7	Acyl-CoA-binding domain-containing protein 7	3.477	4.74.E-02
P37802-2	Isoform 2 of Transgelin-2	3.471	2.32.E-04
P37802	Transgelin-2	3.471	2.32.E-04
Q04446	1,4-alpha-glucan-branching enzyme	3.449	3.19.E-02
P48735-2	Isoform 2 of Isocitrate dehydrogenase [NADP], mitochondrial	3.447	2.47.E-04
P48735	Isocitrate dehydrogenase [NADP], mitochondrial	3.447	2.47.E-04
P49748-2	Isoform 2 of Very long-chain specific acyl-CoA dehydrogenase, mitochondrial	3.439	1.66.E-02
Q15293	Reticulocalbin-1	3.413	2.72.E-04
Q15293-2	Isoform 2 of Reticulocalbin-1	3.413	2.72.E-04
O75915	PRA1 family protein 3	3.408	6.96.E-03
Q9NZU5	LIM and cysteine-rich domains protein 1	3.406	4.21.E-02
Q9NZU5-2	Isoform 2 of LIM and cysteine-rich domains protein 1	3.406	4.21.E-02
O43852-15	Isoform 15 of Calumenin	3.394	2.86.E-04
P07355	Annexin A2	3.383	2.95.E-04
Q9NZM1-6	Isoform 6 of Myoferlin	3.378	2.94.E-03
Q9NZM1	Myoferlin	3.378	2.94.E-03
Q7LG56	Ribonucleoside-diphosphate reductase subunit M2 B	3.357	1.03.E-02
Q8NBJ5	Procollagen galactosyltransferase 1	3.355	8.75.E-03
Q12792	Twinfilin-1	3.354	7.68.E-03
Q7LG56-6	Isoform 6 of Ribonucleoside-diphosphate reductase subunit M2 B	3.35	1.88.E-02
P08069	Insulin-like growth factor 1 receptor	3.346	1.17.E-02
P07355-2	Isoform 2 of Annexin A2	3.345	3.28.E-04
O14531	Dihydropyrimidinase-related protein 4	3.343	1.79.E-02
P78559-2	Isoform 2 of Microtubule-associated protein 1A	3.343	9.90.E-03
P78559	Microtubule-associated protein 1A	3.335	8.34.E-03
P05091-2	Isoform 2 of Aldehyde dehydrogenase, mitochondrial	3.331	1.54.E-03
Q9Y478	5'-AMP-activated protein kinase subunit beta-1	3.331	3.48.E-02
Q16775-2	Isoform 2 of Hydroxyacylglutathione hydrolase, mitochondrial	3.327	1.31.E-02
Q16775	Hydroxyacylglutathione hydrolase, mitochondrial	3.327	1.31.E-02
Q7LG56-2	Isoform 2 of Ribonucleoside-diphosphate reductase subunit M2 B	3.326	1.20.E-02
P05091	Aldehyde dehydrogenase, mitochondrial	3.317	1.70.E-03
P34741	Syndecan-2	3.315	4.11.E-02
P49748	Very long-chain specific acyl-CoA dehydrogenase, mitochondrial	3.292	1.47.E-02
P49748-3	Isoform 3 of Very long-chain specific acyl-CoA dehydrogenase, mitochondrial	3.292	1.47.E-02
Q9NT99	Leucine-rich repeat-containing protein 4B	3.271	4.90.E-02
Q9Y6M7-11	Isoform 11 of Sodium bicarbonate cotransporter 3	3.248	1.85.E-02
Q9Y6M7-10	Isoform 10 of Sodium bicarbonate cotransporter 3	3.248	1.85.E-02
Q9Y6M7-14	Isoform 14 of Sodium bicarbonate cotransporter 3	3.248	1.85.E-02
Q9C0E8-3	Isoform 3 of Endoplasmic reticulum junction formation protein lunapark	3.238	2.22.E-02
O75054-2	Isoform 2 of Immunoglobulin superfamily member 3	3.234	3.79.E-02
P27707	Deoxycytidine kinase	3.226	1.07.E-02

Q92558	Wiskott-Aldrich syndrome protein family member 1	3.218	1.51.E-02
Q9Y4F1-2	Isoform 2 of FERM, ARHGEF and pleckstrin domain-containing protein 1	3.197	1.53.E-02
Q6SZW1	NAD(+) hydrolase SARM1	3.171	2.59.E-02
Q13641	Trophoblast glycoprotein	3.171	1.83.E-02
Q7L099	Protein RUFY3	3.161	5.37.E-03
Q96JJ7	Protein disulfide-isomerase TMX3	3.161	2.30.E-02
Q12792-4	Isoform 4 of Twinfilin-1	3.142	2.43.E-02
Q9NZL9-3	Isoform 3 of Methionine adenosyltransferase 2 subunit beta	3.116	1.65.E-02
P02585	Troponin C, skeletal muscle	3.077	1.70.E-02
O15344	E3 ubiquitin-protein ligase Midline-1	3.071	8.55.E-03
Q9H871-2	Isoform 2 of E3 ubiquitin-protein transferase RMND5A	3.061	4.78.E-02
Q9BQB6-3	Isoform 3 of Vitamin K epoxide reductase complex subunit 1	3.06	2.94.E-02
Q9BQB6-2	Isoform 2 of Vitamin K epoxide reductase complex subunit 1	3.06	2.94.E-02
Q9BQB6	Vitamin K epoxide reductase complex subunit 1	3.06	2.94.E-02
Q96HE7	ERO1-like protein alpha	3.046	2.82.E-02
Q9Y4F1	FERM, ARHGEF and pleckstrin domain-containing protein 1	3.03	1.84.E-02
P49448	Glutamate dehydrogenase 2, mitochondrial	3.016	3.24.E-03
Q9UF33-2	Isoform 2 of Ephrin type-A receptor 6	2.982	1.70.E-02
Q9UF33-3	Isoform 3 of Ephrin type-A receptor 6	2.982	1.70.E-02
Q9UF33	Ephrin type-A receptor 6	2.982	1.70.E-02
Q86YR5-4	Isoform 4 of G-protein-signaling modulator 1	2.978	3.24.E-02
Q86YR5	G-protein-signaling modulator 1	2.978	3.24.E-02
Q7L099-3	Isoform 3 of Protein RUFY3	2.975	6.90.E-03
Q7L099-4	Isoform 4 of Protein RUFY3	2.975	6.90.E-03
Q9NPH2-2	Isoform 2 of Inositol-3-phosphate synthase 1	2.975	3.48.E-03
Q9NPH2	Inositol-3-phosphate synthase 1	2.975	3.39.E-03
Q9NPH2-3	Isoform 3 of Inositol-3-phosphate synthase 1	2.975	3.39.E-03
Q15126	Phosphomevalonate kinase	2.973	4.45.E-02
P30085-3	Isoform 3 of UMP-CMP kinase	2.971	2.30.E-02
P30085	UMP-CMP kinase	2.971	2.30.E-02
Q13642	Four and a half LIM domains protein 1	2.949	1.00.E-03
Q9Y6M7-5	Isoform 5 of Sodium bicarbonate cotransporter 3	2.949	2.18.E-02
P58546	Myotrophin	2.948	6.55.E-03
Q9Y6M7-12	Isoform 12 of Sodium bicarbonate cotransporter 3	2.948	1.98.E-02
Q9Y6M7	Sodium bicarbonate cotransporter 3	2.948	1.98.E-02
Q9Y6M7-13	Isoform 13 of Sodium bicarbonate cotransporter 3	2.948	1.98.E-02
Q9Y6M7-6	Isoform 6 of Sodium bicarbonate cotransporter 3	2.948	1.98.E-02
Q9Y6M7-8	Isoform 8 of Sodium bicarbonate cotransporter 3	2.948	1.98.E-02
Q9Y6M7-7	Isoform 7 of Sodium bicarbonate cotransporter 3	2.948	1.98.E-02
Q9Y6M7-4	Isoform 4 of Sodium bicarbonate cotransporter 3	2.948	1.98.E-02
Q9Y6M7-2	Isoform 2 of Sodium bicarbonate cotransporter 3	2.948	1.98.E-02
Q9Y6M7-9	Isoform 9 of Sodium bicarbonate cotransporter 3	2.948	1.98.E-02
Q9Y6M7-3	Isoform 3 of Sodium bicarbonate cotransporter 3	2.948	1.98.E-02
Q8NBF2	NHL repeat-containing protein 2	2.947	1.42.E-02
P30626	Sorcin	2.946	2.31.E-02
P30626-2	Isoform 2 of Sorcin	2.946	2.31.E-02
P30626-3	Isoform 3 of Sorcin	2.946	2.31.E-02
O15344-2	Isoform 2 of E3 ubiquitin-protein ligase Midline-1	2.928	2.57.E-02
P26232-3	Isoform 3 of Catenin alpha-2	2.918	8.74.E-03
Q14353-2	Isoform 2 of Guanidinoacetate N-methyltransferase	2.897	2.66.E-02
Q9Y4K0	Lysyl oxidase homolog 2	2.858	3.80.E-02
Q14353	Guanidinoacetate N-methyltransferase	2.843	2.43.E-02
Q8NBF2-2	Isoform 2 of NHL repeat-containing protein 2	2.841	3.85.E-02
P23368	NAD-dependent malic enzyme, mitochondrial	2.832	6.04.E-03
P23368-2	Isoform 2 of NAD-dependent malic enzyme, mitochondrial	2.827	5.71.E-03
Q14019	Coactosin-like protein	2.823	1.45.E-03
Q9NVE7	4'-phosphopantetheine phosphatase	2.812	2.53.E-02
Q4G0J3-3	Isoform 3 of La-related protein 7	2.807	4.01.E-02
Q4G0J3	La-related protein 7	2.807	4.01.E-02
P13674-3	Isoform 3 of Prolyl 4-hydroxylase subunit alpha-1	2.783	5.39.E-03
P13674	Prolyl 4-hydroxylase subunit alpha-1	2.783	6.13.E-03
Q8NFV4-4	Isoform 4 of Protein ABHD11	2.78	1.89.E-02
Q8NFV4	Protein ABHD11	2.78	1.89.E-02
Q8NFV4-6	Isoform 6 of Protein ABHD11	2.78	1.89.E-02
Q9NZL9	Methionine adenosyltransferase 2 subunit beta	2.779	2.97.E-02
P54687-2	Isoform 2 of Branched-chain-amino-acid aminotransferase, cytosolic	2.779	1.20.E-02
O43920	NADH dehydrogenase [ubiquinone] iron-sulfur protein 5	2.774	4.57.E-02
O75884	Serine hydrolase RBBP9	2.772	4.46.E-02
P26232-4	Isoform 4 of Catenin alpha-2	2.769	4.92.E-02

P51812	Ribosomal protein S6 kinase alpha-3	2.759	1.27.E-02
P50583	Bis(5'-nucleosyl)-tetraphosphatase [asymmetrical]	2.756	3.11.E-02
P26232-2	Isoform 2 of Catenin alpha-2	2.738	1.34.E-02
P26232-5	Isoform 5 of Catenin alpha-2	2.738	1.34.E-02
P13674-2	Isoform 2 of Prolyl 4-hydroxylase subunit alpha-1	2.73	6.26.E-03
Q96K76-3	Isoform 3 of Ubiquitin carboxyl-terminal hydrolase 47	2.727	1.67.E-03
Q07065	Cytoskeleton-associated protein 4	2.706	2.05.E-03
Q9HC38-3	Isoform 3 of Glyoxalase domain-containing protein 4	2.701	1.23.E-02
Q9NZM1-8	Isoform 8 of Myoferlin	2.673	3.93.E-02
P55084	Trifunctional enzyme subunit beta, mitochondrial	2.668	4.66.E-03
P55084-2	Isoform 2 of Trifunctional enzyme subunit beta, mitochondrial	2.668	4.66.E-03
Q13642-3	Isoform 3 of Four and a half LIM domains protein 1	2.664	2.43.E-03
P0DP25	Calmodulin-3	2.65	1.16.E-02
P07954	Fumarate hydratase, mitochondrial	2.649	8.09.E-03
P07954-2	Isoform Cytoplasmic of Fumarate hydratase, mitochondrial	2.649	8.09.E-03
Q13642-4	Isoform 4 of Four and a half LIM domains protein 1	2.644	2.47.E-03
Q13642-1	Isoform 1 of Four and a half LIM domains protein 1	2.644	2.47.E-03
Q13642-5	Isoform 5 of Four and a half LIM domains protein 1	2.644	2.47.E-03
Q5ZPR3-2	Isoform 2 of CD276 antigen	2.635	2.50.E-02
P07203	Glutathione peroxidase 1	2.631	1.46.E-02
P46821	Microtubule-associated protein 1B	2.616	2.69.E-03
Q9NR12-2	Isoform 2 of PDZ and LIM domain protein 7	2.613	3.66.E-02
P00367	Glutamate dehydrogenase 1, mitochondrial	2.597	5.57.E-03
P05556	Integrin beta-1	2.594	2.69.E-03
P07602-2	Isoform Sap-mu-6 of Prosaposin	2.593	3.40.E-02
P07602	Prosaposin	2.593	3.40.E-02
P07602-3	Isoform Sap-mu-9 of Prosaposin	2.593	3.40.E-02
P54687-4	Isoform 4 of Branched-chain-amino-acid aminotransferase, cytosolic	2.587	1.31.E-02
P05556-5	Isoform 5 of Integrin beta-1	2.586	1.40.E-03
P05556-2	Isoform 2 of Integrin beta-1	2.586	1.40.E-03
P05556-4	Isoform 4 of Integrin beta-1	2.586	1.40.E-03
P05556-3	Isoform 3 of Integrin beta-1	2.586	1.40.E-03
Q5EB52-3	Isoform 3 of Mesoderm-specific transcript homolog protein	2.579	4.21.E-02
O43852-8	Isoform 8 of Calumenin	2.576	4.27.E-02
O43852-7	Isoform 7 of Calumenin	2.576	4.27.E-02
O43852-4	Isoform 4 of Calumenin	2.576	4.27.E-02
O43852-2	Isoform 2 of Calumenin	2.576	4.27.E-02
Q00535	Cyclin-dependent-like kinase 5	2.575	3.12.E-02
P27482	Calmodulin-like protein 3	2.568	3.79.E-02
Q9H6V9-3	Isoform 3 of Lipid droplet-associated hydrolase	2.559	4.90.E-02
Q9HC38-2	Isoform 2 of Glyoxalase domain-containing protein 4	2.556	7.43.E-03
Q9HC38	Glyoxalase domain-containing protein 4	2.556	8.84.E-03
Q9NZL9-2	Isoform 2 of Methionine adenosyltransferase 2 subunit beta	2.542	3.91.E-02
Q9NZL9-4	Isoform 4 of Methionine adenosyltransferase 2 subunit beta	2.542	3.91.E-02
Q9NR12	PDZ and LIM domain protein 7	2.499	3.99.E-02
Q9UL25	Ras-related protein Rab-21	2.492	3.92.E-03
P60520	Gamma-aminobutyric acid receptor-associated protein-like 2	2.476	2.38.E-02
P00367-2	Isoform 2 of Glutamate dehydrogenase 1, mitochondrial	2.468	1.11.E-02
P07305	Histone H1.0	2.444	4.73.E-02
P07305-2	Isoform 2 of Histone H1.0	2.444	4.73.E-02
P35579	Myosin-9	2.438	4.63.E-03
P54687-5	Isoform 5 of Branched-chain-amino-acid aminotransferase, cytosolic	2.437	1.88.E-02
Q12765-2	Isoform 2 of Secernin-1	2.436	4.43.E-02
Q12765	Secernin-1	2.436	4.43.E-02
P00367-3	Isoform 3 of Glutamate dehydrogenase 1, mitochondrial	2.433	1.40.E-02
P28161	Glutathione S-transferase Mu 2	2.433	4.80.E-02
P28161-2	Isoform 2 of Glutathione S-transferase Mu 2	2.433	4.93.E-02
P31150	Rab GDP dissociation inhibitor alpha	2.416	4.66.E-03
Q6YN16-2	Isoform 2 of Hydroxysteroid dehydrogenase-like protein 2	2.408	4.33.E-02
Q6YN16	Hydroxysteroid dehydrogenase-like protein 2	2.408	4.33.E-02
O15031	Plexin-B2	2.396	2.75.E-02
Q07954	Prolow-density lipoprotein receptor-related protein 1	2.383	5.49.E-03
Q8WXH0-2	Isoform 2 of Nesprin-2	2.373	5.65.E-03
Q8WXH0	Nesprin-2	2.373	5.65.E-03
O94788	Retinal dehydrogenase 2	2.372	4.91.E-02
O94788-2	Isoform 2 of Retinal dehydrogenase 2	2.372	4.91.E-02
O94788-3	Isoform 3 of Retinal dehydrogenase 2	2.372	4.91.E-02
O94788-4	Isoform 4 of Retinal dehydrogenase 2	2.372	4.91.E-02
P35579-2	Isoform 2 of Myosin-9	2.362	5.85.E-03

P0CG47	Polyubiquitin-B	2.334	6.40.E-03
P0CG48	Polyubiquitin-C	2.334	6.40.E-03
Q13885	Tubulin beta-2A chain	2.328	3.11.E-02
P20020-1	Isoform D of Plasma membrane calcium-transporting ATPase 1	2.323	1.89.E-02
P20020-4	Isoform C of Plasma membrane calcium-transporting ATPase 1	2.323	1.89.E-02
P20020	Plasma membrane calcium-transporting ATPase 1	2.323	1.89.E-02
P20020-6	Isoform K of Plasma membrane calcium-transporting ATPase 1	2.323	1.89.E-02
P49257	Protein ERGIC-53	2.312	6.48.E-03
Q9H6V9-2	Isoform 2 of Lipid droplet-associated hydrolase	2.301	4.77.E-02
Q9H6V9	Lipid droplet-associated hydrolase	2.301	4.77.E-02
P33176	Kinesin-1 heavy chain	2.288	1.00.E-02
P20020-2	Isoform A of Plasma membrane calcium-transporting ATPase 1	2.254	2.57.E-02
P20020-5	Isoform E of Plasma membrane calcium-transporting ATPase 1	2.254	2.57.E-02
Q9H9B4	Sideroflexin-1	2.214	3.76.E-02
Q9NSE4	Isoleucine--tRNA ligase, mitochondrial	2.192	2.84.E-02
Q96G03	Phosphoglucomutase-2	2.19	1.01.E-02
Q9BRX8	Peroxiredoxin-like 2A	2.189	4.98.E-02
Q9BRX8-2	Isoform 2 of Peroxiredoxin-like 2A	2.189	4.98.E-02
P34949	Mannose-6-phosphate isomerase	2.181	4.10.E-02
Q02952	A-kinase anchor protein 12	2.162	1.10.E-02
Q02952-2	Isoform 2 of A-kinase anchor protein 12	2.162	1.10.E-02
Q02952-3	Isoform 3 of A-kinase anchor protein 12	2.162	1.10.E-02
Q9BS26	Endoplasmic reticulum resident protein 44	2.156	3.09.E-02
Q71UI9-3	Isoform 3 of Histone H2A.V	2.15	1.14.E-02
Q71UI9-4	Isoform 4 of Histone H2A.V	2.15	1.14.E-02
Q96K76	Ubiquitin carboxyl-terminal hydrolase 47	2.135	1.20.E-02
Q96K76-2	Isoform 2 of Ubiquitin carboxyl-terminal hydrolase 47	2.135	1.20.E-02
Q96K76-4	Isoform 4 of Ubiquitin carboxyl-terminal hydrolase 47	2.135	1.20.E-02
Q13177	Serine/threonine-protein kinase PAK 2	2.123	2.01.E-02
O95336	6-phosphogluconolactonase	2.103	3.73.E-02
P27824-3	Isoform 3 of Calnexin	2.099	1.35.E-02
Q16527	Cysteine and glycine-rich protein 2	2.085	2.22.E-02
Q16836-3	Isoform 3 of Hydroxyacyl-coenzyme A dehydrogenase, mitochondrial	2.081	1.99.E-02
Q16836-2	Isoform 2 of Hydroxyacyl-coenzyme A dehydrogenase, mitochondrial	2.081	1.99.E-02
Q16836	Hydroxyacyl-coenzyme A dehydrogenase, mitochondrial	2.081	1.99.E-02
P27824-2	Isoform 2 of Calnexin	2.058	1.53.E-02
P27824	Calnexin	2.058	1.53.E-02
P02786	Transferrin receptor protein 1	2.051	2.47.E-02
Q6EEV6	Small ubiquitin-related modifier 4	2.043	2.71.E-02
P55854-2	Isoform 2 of Small ubiquitin-related modifier 3	2.043	2.71.E-02
P55854	Small ubiquitin-related modifier 3	2.043	2.71.E-02
Q16698	2,4-dienoyl-CoA reductase, mitochondrial	2.033	3.97.E-02
Q16698-2	Isoform 2 of 2,4-dienoyl-CoA reductase, mitochondrial	2.033	3.97.E-02
Q32P28-3	Isoform 3 of Prolyl 3-hydroxylase 1	2.027	4.05.E-02
Q32P28-4	Isoform 4 of Prolyl 3-hydroxylase 1	2.027	4.05.E-02
Q32P28	Prolyl 3-hydroxylase 1	2.027	4.05.E-02
Q05682-5	Isoform 5 of Caldesmon	2.014	2.56.E-02
Q05682-4	Isoform 4 of Caldesmon	2.014	2.56.E-02
Q05682-6	Isoform 6 of Caldesmon	2.014	2.56.E-02
Q05682-2	Isoform 2 of Caldesmon	2.014	2.56.E-02
Q05682-3	Isoform 3 of Caldesmon	2.014	2.56.E-02
Q05682	Caldesmon	2.014	2.56.E-02

Supplementary Table S1. (c) Proteins with increased levels in the DAs (>2 fold)

Accession	Protein Name	Abundance Ratio: (DA) / (iPSC)	Abundance Ratio p-Value: (DA) / (iPSC)
P13591	Neural cell adhesion molecule 1	100	1.00.E-17
P13591-1	Isoform 2 of Neural cell adhesion molecule 1	100	1.00.E-17
Q16352	Alpha-internexin	100	1.00.E-17
P13591-4	Isoform 4 of Neural cell adhesion molecule 1	100	1.00.E-17
P13591-3	Isoform 3 of Neural cell adhesion molecule 1	100	1.00.E-17
P13591-5	Isoform 5 of Neural cell adhesion molecule 1	100	1.00.E-17
O43602-2	Isoform 2 of Neuronal migration protein doublecortin	100	1.00.E-17
P13591-6	Isoform 6 of Neural cell adhesion molecule 1	100	1.00.E-17
Q9UI15	Transgelin-3	100	1.00.E-17
P14618-2	Isoform M1 of Pyruvate kinase PKM	100	1.00.E-17
Q8WVB6-3	Isoform 3 of Chromosome transmission fidelity protein 18 homolog	100	1.00.E-17
Q8WVB6-2	Isoform 2 of Chromosome transmission fidelity protein 18 homolog	100	1.00.E-17
Q8WVB6	Chromosome transmission fidelity protein 18 homolog	100	1.00.E-17
Q7Z6L0	Proline-rich transmembrane protein 2	100	1.00.E-17
Q7Z6L0-2	Isoform 2 of Proline-rich transmembrane protein 2	100	1.00.E-17
Q7Z6L0-3	Isoform 3 of Proline-rich transmembrane protein 2	100	1.00.E-17
P23763	Vesicle-associated membrane protein 1	100	1.00.E-17
P23763-2	Isoform 3 of Vesicle-associated membrane protein 1	100	1.00.E-17
P23763-3	Isoform 2 of Vesicle-associated membrane protein 1	100	1.00.E-17
P26378	ELAV-like protein 4	100	1.00.E-17
P26378-1	Isoform 1 of ELAV-like protein 4	100	1.00.E-17
P26378-3	Isoform 3 of ELAV-like protein 4	100	1.00.E-17
P26378-5	Isoform 5 of ELAV-like protein 4	100	1.00.E-17
P26378-4	Isoform 4 of ELAV-like protein 4	100	1.00.E-17
P26378-2	Isoform 2 of ELAV-like protein 4	100	1.00.E-17
Q8IUH4	Palmitoyltransferase ZDHHC13	100	1.00.E-17
Q9H902-3	Isoform 3 of Receptor expression-enhancing protein 1	100	1.00.E-17
Q9H902	Receptor expression-enhancing protein 1	100	1.00.E-17
Q9H902-2	Isoform 2 of Receptor expression-enhancing protein 1	100	1.00.E-17
Q9GZT4	Serine racemase	100	1.00.E-17
O15217-2	Isoform 2 of Glutathione S-transferase A4	100	1.00.E-17
O94772-2	Isoform 2 of Lymphocyte antigen 6H	100	1.00.E-17
O94772	Lymphocyte antigen 6H	100	1.00.E-17
O75051-2	Isoform 2 of Plexin-A2	100	1.00.E-17
Q9HAR2-2	Isoform 2 of Adhesion G protein-coupled receptor L3	100	1.00.E-17
Q9HAR2-4	Isoform 4 of Adhesion G protein-coupled receptor L3	100	1.00.E-17
Q9HAR2	Adhesion G protein-coupled receptor L3	100	1.00.E-17
O60641-2	Isoform 2 of Clathrin coat assembly protein AP180	100	1.00.E-17
O43602	Neuronal migration protein doublecortin	100	1.00.E-17
Q9HAP6-2	Isoform 2 of Protein lin-7 homolog B	100	1.00.E-17
Q9ULB1	Neurexin-1	100	1.00.E-17
Q07866-8	Isoform S of Kinesin light chain 1	100	1.00.E-17
Q8N4Q0	Prostaglandin reductase 3	100	1.00.E-17
Q9NY59-2	Isoform 2 of Sphingomyelin phosphodiesterase 3	100	1.00.E-17
Q9NY59	Sphingomyelin phosphodiesterase 3	100	1.00.E-17
Q96SB3	Neurabin-2	100	1.00.E-17
Q9UPR5	Sodium/calcium exchanger 2	100	1.00.E-17
Q9BRK4	Leucine zipper putative tumor suppressor 2	100	1.00.E-17
Q9HAR2-3	Isoform 3 of Adhesion G protein-coupled receptor L3	100	1.00.E-17
P32418-2	Isoform 3 of Sodium/calcium exchanger 1	100	1.00.E-17
P32418-3	Isoform 7 of Sodium/calcium exchanger 1	100	1.00.E-17
P32418-4	Isoform 10 of Sodium/calcium exchanger 1	100	1.00.E-17
P32418	Sodium/calcium exchanger 1	100	1.00.E-17
P32418-5	Isoform 5 of Sodium/calcium exchanger 1	100	1.00.E-17
P52895	Aldo-keto reductase family 1 member C2	100	1.00.E-17
Q9ULB1-4	Isoform 4 of Neurexin-1	100	1.00.E-17
P58400-1	Isoform 3b of Neurexin-1-beta	100	1.00.E-17
Q5GH72	XK-related protein 7	100	1.00.E-17
O60641	Clathrin coat assembly protein AP180	100	1.00.E-17
Q8N4Q0-2	Isoform 2 of Prostaglandin reductase 3	100	1.00.E-17
P29320	Ephrin type-A receptor 3	100	1.00.E-17
P56159	GDNF family receptor alpha-1	100	1.00.E-17
P56159-2	Isoform 2 of GDNF family receptor alpha-1	100	1.00.E-17
P52895-2	Isoform 2 of Aldo-keto reductase family 1 member C2	100	1.00.E-17

Q6ZVF9	G protein-regulated inducer of neurite outgrowth 3	100	1.00.E-17
Q5T1Q4-2	Isoform 2 of Solute carrier family 35 member F1	100	1.00.E-17
Q5T1Q4	Solute carrier family 35 member F1	100	1.00.E-17
Q9UBS5	Gamma-aminobutyric acid type B receptor subunit 1	100	1.00.E-17
Q9UBS5-4	Isoform 1D of Gamma-aminobutyric acid type B receptor subunit 1	100	1.00.E-17
Q9UBS5-5	Isoform 1E of Gamma-aminobutyric acid type B receptor subunit 1	100	1.00.E-17
Q9UBS5-3	Isoform 1C of Gamma-aminobutyric acid type B receptor subunit 1	100	1.00.E-17
Q16526	Cryptochrome-1	100	1.00.E-17
Q9UM19	Hippocalcin-like protein 4	100	1.00.E-17
Q9Y6I9	Testis-expressed protein 264	100	1.00.E-17
Q9NTI2	Phospholipid-transporting ATPase IB	100	1.00.E-17
Q9NTI2-1	Isoform 1 of Phospholipid-transporting ATPase IB	100	1.00.E-17
Q9NTI2-3	Isoform 2 of Phospholipid-transporting ATPase IB	100	1.00.E-17
Q7Z6K5	Arpin	100	1.00.E-17
Q15173	Serine/threonine-protein phosphatase 2A 56 kDa regulatory subunit beta isoform	100	1.00.E-17
Q15173-2	Isoform Beta-2 of Serine/threonine-protein phosphatase 2A 56 kDa regulatory subunit beta isoform	100	1.00.E-17
O95670-3	Isoform 3 of V-type proton ATPase subunit G 2	100	1.00.E-17
Q9Y653-5	Isoform 5 of Adhesion G-protein coupled receptor G1	100	1.00.E-17
Q9Y653-2	Isoform 2 of Adhesion G-protein coupled receptor G1	100	1.00.E-17
Q9Y653	Adhesion G-protein coupled receptor G1	100	1.00.E-17
Q9Y653-3	Isoform 3 of Adhesion G-protein coupled receptor G1	100	1.00.E-17
O75334	Liprin-alpha-2	100	1.00.E-17
O75334-5	Isoform 5 of Liprin-alpha-2	100	1.00.E-17
O75334-2	Isoform 2 of Liprin-alpha-2	100	1.00.E-17
O75334-3	Isoform 3 of Liprin-alpha-2	100	1.00.E-17
O75334-4	Isoform 4 of Liprin-alpha-2	100	1.00.E-17
A1A5C7	Solute carrier family 22 member 23	100	1.00.E-17
Q8WXA3-4	Isoform 3 of RUN and FYVE domain-containing protein 2	100	1.00.E-17
O75334-6	Isoform 6 of Liprin-alpha-2	100	1.00.E-17
P54803	Galactocerebrosidase	100	1.00.E-17
Q8WXA3-5	Isoform 4 of RUN and FYVE domain-containing protein 2	100	1.00.E-17
O43426-5	Isoform 4 of Synaptojanin-1	100	1.00.E-17
Q9H4D0	Calsyntenin-2	100	1.00.E-17
P29320-2	Isoform 2 of Ephrin type-A receptor 3	100	1.00.E-17
P49441	Inositol polyphosphate 1-phosphatase	100	1.00.E-17
O95297-3	Isoform 3 of Myelin protein zero-like protein 1	100	1.00.E-17
Q8NFZ4	Neuroligin-2	100	1.00.E-17
O75093	Slit homolog 1 protein	100	1.00.E-17
Q5SZQ8-3	Isoform 3 of CUGBP Elav-like family member 3	100	1.00.E-17
Q96J87-4	Isoform 4 of CUGBP Elav-like family member 6	100	1.00.E-17
Q96J87-2	Isoform 2 of CUGBP Elav-like family member 6	100	1.00.E-17
Q96J87	CUGBP Elav-like family member 6	100	1.00.E-17
Q5SZQ8-2	Isoform 2 of CUGBP Elav-like family member 3	100	1.00.E-17
Q5SZQ8-4	Isoform 4 of CUGBP Elav-like family member 3	100	1.00.E-17
Q96J87-3	Isoform 3 of CUGBP Elav-like family member 6	100	1.00.E-17
Q5SZQ8	CUGBP Elav-like family member 3	100	1.00.E-17
Q9NWQ8	Phosphoprotein associated with glycosphingolipid-enriched microdomains 1	100	1.00.E-17
Q9UPN3	Microtubule-actin cross-linking factor 1, isoforms 1/2/3/5	100	1.00.E-17
Q9UPN3-4	Isoform 5 of Microtubule-actin cross-linking factor 1, isoforms 1/2/3/5	100	1.00.E-17
Q96B86-3	Isoform 2 of Repulsive guidance molecule A	100	1.00.E-17
Q96B86	Repulsive guidance molecule A	100	1.00.E-17
Q96B86-4	Isoform 3 of Repulsive guidance molecule A	100	1.00.E-17
Q9NZR2	Low-density lipoprotein receptor-related protein 1B	100	1.00.E-17
P55273	Cyclin-dependent kinase 4 inhibitor D	100	1.00.E-17
Q8NFT8	Delta and Notch-like epidermal growth factor-related receptor	100	1.00.E-17
Q13114	TNF receptor-associated factor 3	100	1.00.E-17
C9JRZ8	Aldo-keto reductase family 1 member B15	100	1.00.E-17
C9JRZ8-2	Isoform 2 of Aldo-keto reductase family 1 member B15	100	1.00.E-17
P05067-3	Isoform L-APP677 of Amyloid-beta precursor protein	100	1.00.E-17
P05067-10	Isoform APP639 of Amyloid-beta precursor protein	100	1.00.E-17
P05067-4	Isoform APP695 of Amyloid-beta precursor protein	100	1.00.E-17
Q96HU8	GTP-binding protein Di-Ras2	100	1.00.E-17
P47895	Aldehyde dehydrogenase family 1 member A3	100	1.00.E-17
O14576-4	Isoform 4 of Cytoplasmic dynein 1 intermediate chain 1	100	1.00.E-17
O14576-5	Isoform 5 of Cytoplasmic dynein 1 intermediate chain 1	100	1.00.E-17
O14576-2	Isoform 2 of Cytoplasmic dynein 1 intermediate chain 1	100	1.00.E-17

O14576-3	Isoform 3 of Cytoplasmic dynein 1 intermediate chain 1	100	1.00.E-17
O14576	Cytoplasmic dynein 1 intermediate chain 1	100	1.00.E-17
P43007	Neutral amino acid transporter A	100	1.00.E-17
P43007-2	Isoform 2 of Neutral amino acid transporter A	100	1.00.E-17
Q9H4G0-2	Isoform 2 of Band 4.1-like protein 1	100	1.00.E-17
Q99767-2	Isoform 2 of Amyloid-beta A4 precursor protein-binding family A member 2	100	1.00.E-17
Q99767	Amyloid-beta A4 precursor protein-binding family A member 2	100	1.00.E-17
A1A5C7-4	Isoform 4 of Solute carrier family 22 member 23	100	1.00.E-17
P23435	Cerebellin-1	100	1.00.E-17
O95479	GDH/6PGL endoplasmic bifunctional protein	100	1.00.E-17
O95479-2	Isoform 2 of GDH/6PGL endoplasmic bifunctional protein	100	1.00.E-17
Q9Y653-4	Isoform 4 of Adhesion G-protein coupled receptor G1	100	1.00.E-17
Q15111	Inactive phospholipase C-like protein 1	100	1.00.E-17
H3BS89	Transmembrane protein 178B	100	1.00.E-17
P81274	G-protein-signaling modulator 2	100	1.00.E-17
Q14982-4	Isoform 4 of Opioid-binding protein/cell adhesion molecule	100	1.00.E-17
Q14982-2	Isoform 2 of Opioid-binding protein/cell adhesion molecule	100	1.00.E-17
Q14982-3	Isoform 3 of Opioid-binding protein/cell adhesion molecule	100	1.00.E-17
Q14982	Opioid-binding protein/cell adhesion molecule	100	1.00.E-17
Q99946	Proline-rich transmembrane protein 1	100	1.00.E-17
Q02153	Guanylate cyclase soluble subunit beta-1	100	1.00.E-17
Q02153-3	Isoform 3 of Guanylate cyclase soluble subunit beta-1	100	1.00.E-17
Q02153-2	Isoform HSGC-2 of Guanylate cyclase soluble subunit beta-1	100	1.00.E-17
Q96N03-2	Isoform 2 of V-set and transmembrane domain-containing protein 2-like protein	100	1.00.E-17
Q96N03	V-set and transmembrane domain-containing protein 2-like protein	100	1.00.E-17
P61601	Neurocalcin-delta	100	1.00.E-17
Q5SYC1	Clavesin-2	100	1.00.E-17
Q96EQ0	Small glutamine-rich tetratricopeptide repeat-containing protein beta	100	1.00.E-17
Q9HCD6-3	Isoform 3 of Protein TANC2	100	1.00.E-17
Q9HCD6-4	Isoform 4 of Protein TANC2	100	1.00.E-17
Q9Y3E7-2	Isoform 2 of Charged multivesicular body protein 3	100	1.00.E-17
Q9Y3E7	Charged multivesicular body protein 3	100	1.00.E-17
Q9Y3E7-4	Isoform 4 of Charged multivesicular body protein 3	100	1.00.E-17
Q9Y3E7-3	Isoform 3 of Charged multivesicular body protein 3	100	1.00.E-17
O14770	Homeobox protein Meis2	100	1.00.E-17
O14770-8	Isoform 8 of Homeobox protein Meis2	100	1.00.E-17
O00470	Homeobox protein Meis1	100	1.00.E-17
O00470-2	Isoform 2 of Homeobox protein Meis1	100	1.00.E-17
O14770-7	Isoform 7 of Homeobox protein Meis2	100	1.00.E-17
O14770-5	Isoform 5 of Homeobox protein Meis2	100	1.00.E-17
O14770-3	Isoform 3 of Homeobox protein Meis2	100	1.00.E-17
O14770-6	Isoform 6 of Homeobox protein Meis2	100	1.00.E-17
O14770-4	Isoform 4 of Homeobox protein Meis2	100	1.00.E-17
O14770-2	Isoform 2 of Homeobox protein Meis2	100	1.00.E-17
Q9Y3R0-3	Isoform 3 of Glutamate receptor-interacting protein 1	100	1.00.E-17
Q9Y3R0-2	Isoform 2 of Glutamate receptor-interacting protein 1	100	1.00.E-17
Q9Y3R0	Glutamate receptor-interacting protein 1	100	1.00.E-17
O15294-3	Isoform 1 of UDP-N-acetylglucosamine--peptide N-acetylglucosaminyltransferase 110 kDa subunit	100	1.00.E-17
Q9NY72	Sodium channel subunit beta-3	100	1.00.E-17
Q13114-2	Isoform 2 of TNF receptor-associated factor 3	100	1.00.E-17
Q9P0B6	Coiled-coil domain-containing protein 167	100	1.00.E-17
Q9UKF7	Cytoplasmic phosphatidylinositol transfer protein 1	100	1.00.E-17
Q9UKF7-2	Isoform 2 of Cytoplasmic phosphatidylinositol transfer protein 1	100	1.00.E-17
P46108-2	Isoform Crk-I of Adapter molecule crk	100	1.00.E-17
P04062-2	Isoform Short of Lysosomal acid glucosylceramidase	100	1.00.E-17
P04062	Lysosomal acid glucosylceramidase	100	1.00.E-17
P04062-5	Isoform 5 of Lysosomal acid glucosylceramidase	100	1.00.E-17
P04062-4	Isoform 4 of Lysosomal acid glucosylceramidase	100	1.00.E-17
P47869	Gamma-aminobutyric acid receptor subunit alpha-2	100	1.00.E-17
P14867	Gamma-aminobutyric acid receptor subunit alpha-1	100	1.00.E-17
P47869-2	Isoform 2 of Gamma-aminobutyric acid receptor subunit alpha-2	100	1.00.E-17
P31644	Gamma-aminobutyric acid receptor subunit alpha-5	100	1.00.E-17
O95197-4	Isoform 4 of Reticulon-3	100	1.00.E-17
A1A5C7-2	Isoform 2 of Solute carrier family 22 member 23	100	1.00.E-17
A1A5C7-3	Isoform 3 of Solute carrier family 22 member 23	100	1.00.E-17
Q9P2S2-2	Isoform 2a of Neurexin-2	100	1.00.E-17

Q9P2S2	Neurexin-2	100	1.00.E-17
P58401	Neurexin-2-beta	100	1.00.E-17
Q13554-8	Isoform 8 of Calcium/calmodulin-dependent protein kinase type II subunit beta	100	1.00.E-17
Q13554-4	Isoform 3 of Calcium/calmodulin-dependent protein kinase type II subunit beta	100	1.00.E-17
Q13554-5	Isoform 5 of Calcium/calmodulin-dependent protein kinase type II subunit beta	100	1.00.E-17
Q13554-7	Isoform 7 of Calcium/calmodulin-dependent protein kinase type II subunit beta	100	1.00.E-17
Q13554	Calcium/calmodulin-dependent protein kinase type II subunit beta	100	1.00.E-17
Q13554-2	Isoform 1 of Calcium/calmodulin-dependent protein kinase type II subunit beta	100	1.00.E-17
Q13554-3	Isoform 2 of Calcium/calmodulin-dependent protein kinase type II subunit beta	100	1.00.E-17
Q15700-3	Isoform 3 of Disks large homolog 2	100	1.00.E-17
Q15700-5	Isoform 5 of Disks large homolog 2	100	1.00.E-17
Q15700	Disks large homolog 2	100	1.00.E-17
Q15700-2	Isoform 2 of Disks large homolog 2	100	1.00.E-17
Q15700-4	Isoform 4 of Disks large homolog 2	100	1.00.E-17
P04271	Protein S100-B	100	1.00.E-17
Q9UPW8	Protein unc-13 homolog A	100	1.00.E-17
O14795-2	Isoform 2 of Protein unc-13 homolog B	100	1.00.E-17
Q8NB66	Protein unc-13 homolog C	100	1.00.E-17
O14795	Protein unc-13 homolog B	100	1.00.E-17
Q8N350-4	Isoform 2 of Voltage-dependent calcium channel beta subunit-associated regulatory protein	100	1.00.E-17
Q8N350	Voltage-dependent calcium channel beta subunit-associated regulatory protein	100	1.00.E-17
Q53HC9	EARP and GARP complex-interacting protein 1	100	1.00.E-17
Q8NBJ7-5	Isoform 5 of Inactive C-alpha-formylglycine-generating enzyme 2	100	1.00.E-17
Q9BXJ8	Ion channel TACAN	100	1.00.E-17
Q9BXJ8-2	Isoform 2 of Ion channel TACAN	100	1.00.E-17
P23468-5	Isoform 5 of Receptor-type tyrosine-protein phosphatase delta	100	1.00.E-17
P23468-7	Isoform 7 of Receptor-type tyrosine-protein phosphatase delta	100	1.00.E-17
P23468-3	Isoform 3 of Receptor-type tyrosine-protein phosphatase delta	100	1.00.E-17
P23468	Receptor-type tyrosine-protein phosphatase delta	100	1.00.E-17
P23468-6	Isoform 6 of Receptor-type tyrosine-protein phosphatase delta	100	1.00.E-17
Q9Y4K3	TNF receptor-associated factor 6	100	1.00.E-17
Q9P2G3	Kelch-like protein 14	100	1.00.E-17
Q9P2G3-2	Isoform 2 of Kelch-like protein 14	100	1.00.E-17
Q8IYK8	GTP-binding protein REM 2	100	1.00.E-17
P13929-2	Isoform 2 of Beta-enolase	100	1.00.E-17
P22694-6	Isoform 6 of cAMP-dependent protein kinase catalytic subunit beta	100	1.00.E-17
P22694-9	Isoform 9 of cAMP-dependent protein kinase catalytic subunit beta	100	1.00.E-17
O14523	Phospholipid transfer protein C2CD2L	100	1.00.E-17
O14523-2	Isoform 2 of Phospholipid transfer protein C2CD2L	100	1.00.E-17
Q9NYQ7-2	Isoform 2 of Cadherin EGF LAG seven-pass G-type receptor 3	100	1.00.E-17
Q9NYQ7	Cadherin EGF LAG seven-pass G-type receptor 3	100	1.00.E-17
Q96DE0-4	Isoform 4 of U8 snoRNA-decapping enzyme	100	1.00.E-17
Q8N2G4-3	Isoform 3 of Ly6/PLAUR domain-containing protein 1	100	1.00.E-17
Q8N2G4	Ly6/PLAUR domain-containing protein 1	100	1.00.E-17
Q8N2G4-4	Isoform 4 of Ly6/PLAUR domain-containing protein 1	100	1.00.E-17
Q8N2G4-2	Isoform 2 of Ly6/PLAUR domain-containing protein 1	100	1.00.E-17
O00533	Neural cell adhesion molecule L1-like protein	100	1.00.E-17
O00533-2	Isoform 2 of Neural cell adhesion molecule L1-like protein	100	1.00.E-17
Q9H1C7	Cysteine-rich and transmembrane domain-containing protein 1	100	1.00.E-17
Q15111-2	Isoform 2 of Inactive phospholipase C-like protein 1	100	1.00.E-17
Q8IZR5-2	Isoform 2 of CKLF-like MARVEL transmembrane domain-containing protein 4	100	1.00.E-17
Q8IZR5-3	Isoform 3 of CKLF-like MARVEL transmembrane domain-containing protein 4	100	1.00.E-17
Q8IZR5	CKLF-like MARVEL transmembrane domain-containing protein 4	100	1.00.E-17
Q8N5H7-2	Isoform 2 of SH2 domain-containing protein 3C	100	1.00.E-17
Q8N5H7-3	Isoform 3 of SH2 domain-containing protein 3C	100	1.00.E-17
Q8N5H7	SH2 domain-containing protein 3C	100	1.00.E-17
Q8N5H7-5	Isoform 5 of SH2 domain-containing protein 3C	100	1.00.E-17
Q8N5H7-6	Isoform 6 of SH2 domain-containing protein 3C	100	1.00.E-17
Q8N5H7-4	Isoform 4 of SH2 domain-containing protein 3C	100	1.00.E-17
Q8WWM9	Cytoglobin	100	1.00.E-17
Q9NYI0-3	Isoform 3 of PH and SEC7 domain-containing protein 3	100	1.00.E-17
Q9NYI0-2	Isoform 2 of PH and SEC7 domain-containing protein 3	100	1.00.E-17
Q9NYI0	PH and SEC7 domain-containing protein 3	100	1.00.E-17
Q96Q04	Serine/threonine-protein kinase LMTK3	100	1.00.E-17
P30531	Sodium- and chloride-dependent GABA transporter 1	100	1.00.E-17
P0DMP2	SLIT-ROBO Rho GTPase-activating protein 2B	100	1.00.E-17
P0DJJ0	SLIT-ROBO Rho GTPase-activating protein 2C	100	1.00.E-17

Q9UPV7	PHD finger protein 24	100	1.00.E-17
O95630	STAM-binding protein	100	1.00.E-17
Q9BYT3	Serine/threonine-protein kinase 33	100	1.00.E-17
O94910-2	Isoform 2 of Adhesion G protein-coupled receptor L1	100	1.00.E-17
O94910	Adhesion G protein-coupled receptor L1	100	1.00.E-17
Q8TDW7-2	Isoform 2 of Protocadherin Fat 3	100	1.00.E-17
Q96S06	Lipase maturation factor 1	100	1.00.E-17
Q9Y4C0-3	Isoform 3a of Neurexin-3	100	1.00.E-17
Q9Y4C0-4	Isoform 4a of Neurexin-3	100	1.00.E-17
Q9Y4C0	Neurexin-3	100	1.00.E-17
Q5GH76	XK-related protein 4	100	1.00.E-17
P07101-4	Isoform 4 of Tyrosine 3-monooxygenase	100	1.00.E-17
P07101	Tyrosine 3-monooxygenase	100	1.00.E-17
P07101-5	Isoform 5 of Tyrosine 3-monooxygenase	100	1.00.E-17
P07101-6	Isoform 6 of Tyrosine 3-monooxygenase	100	1.00.E-17
P07101-3	Isoform 2 of Tyrosine 3-monooxygenase	100	1.00.E-17
P07101-2	Isoform 1 of Tyrosine 3-monooxygenase	100	1.00.E-17
Q04206-4	Isoform 4 of Transcription factor p65	100	1.00.E-17
Q6P6B1	Glutamate-rich protein 5	100	1.00.E-17
Q8IWE4	DCN1-like protein 3	100	1.00.E-17
Q969R2-5	Isoform 5 of Oxysterol-binding protein 2	100	1.00.E-17
Q969R2-4	Isoform 4 of Oxysterol-binding protein 2	100	1.00.E-17
O43581-4	Isoform 4 of Synaptotagmin-7	100	1.00.E-17
O43581	Synaptotagmin-7	100	1.00.E-17
O43581-6	Isoform 6 of Synaptotagmin-7	100	1.00.E-17
O00255-2	Isoform 2 of Menin	100	1.00.E-17
O00255-3	Isoform 3 of Menin	100	1.00.E-17
O00255	Menin	100	1.00.E-17
Q9UPU3	VPS10 domain-containing receptor SorCS3	100	1.00.E-17
Q9ULP0-4	Isoform 4 of Protein NDRG4	100	1.00.E-17
Q9ULP0	Protein NDRG4	100	1.00.E-17
Q9ULP0-2	Isoform 2 of Protein NDRG4	100	1.00.E-17
Q9ULP0-5	Isoform 5 of Protein NDRG4	100	1.00.E-17
Q96HH4	Transmembrane protein 169	100	1.00.E-17
P33897	ATP-binding cassette sub-family D member 1	100	1.00.E-17
Q8N2Q7	Neuroligin-1	100	1.00.E-17
Q8N2Q7-2	Isoform 2 of Neuroligin-1	100	1.00.E-17
Q86TC9-2	Isoform 2 of Myopalladin	100	1.00.E-17
Q86TC9	Myopalladin	100	1.00.E-17
P56373	P2X purinoceptor 3	100	1.00.E-17
O60229-5	Isoform 5 of Kalirin	100	1.00.E-17
Q6PHR2-3	Isoform 3 of Serine/threonine-protein kinase ULK3	100	1.00.E-17
Q6PHR2	Serine/threonine-protein kinase ULK3	100	1.00.E-17
Q6PHR2-2	Isoform 2 of Serine/threonine-protein kinase ULK3	100	1.00.E-17
Q6PHR2-4	Isoform 4 of Serine/threonine-protein kinase ULK3	100	1.00.E-17
Q9HCE9-2	Isoform 2 of Anoctamin-8	100	1.00.E-17
Q9HCE9	Anoctamin-8	100	1.00.E-17
Q8NHH9-4	Isoform 4 of Atlastin-2	100	1.00.E-17
Q5T4F4	Protrudin	100	1.00.E-17
Q5T4F4-8	Isoform 8 of Protrudin	100	1.00.E-17
Q5T4F4-6	Isoform 6 of Protrudin	100	1.00.E-17
Q5T4F4-2	Isoform 2 of Protrudin	100	1.00.E-17
Q5T4F4-5	Isoform 5 of Protrudin	100	1.00.E-17
Q5T4F4-7	Isoform 7 of Protrudin	100	1.00.E-17
Q5T4F4-3	Isoform 3 of Protrudin	100	1.00.E-17
Q8IVP5	FUN14 domain-containing protein 1	100	1.00.E-17
P37059	Estradiol 17-beta-dehydrogenase 2	100	1.00.E-17
Q8WXA3-1	Isoform 5 of RUN and FYVE domain-containing protein 2	100	1.00.E-17
Q8WXA3	RUN and FYVE domain-containing protein 2	100	1.00.E-17
Q8WXA3-3	Isoform 2 of RUN and FYVE domain-containing protein 2	100	1.00.E-17
P39210	Protein Mpv17	100	1.00.E-17
O94813-3	Isoform 3 of Slit homolog 2 protein	100	1.00.E-17
O94813	Slit homolog 2 protein	100	1.00.E-17
O94813-2	Isoform 2 of Slit homolog 2 protein	100	1.00.E-17
P58511	Small integral membrane protein 11A	100	1.00.E-17
Q14511-3	Isoform 3 of Enhancer of filamentation 1	100	1.00.E-17
Q14511	Enhancer of filamentation 1	100	1.00.E-17

Q9GZY8	Mitochondrial fission factor	100	1.00.E-17
Q9GZY8-5	Isoform 5 of Mitochondrial fission factor	100	1.00.E-17
Q9GZY8-2	Isoform 2 of Mitochondrial fission factor	100	1.00.E-17
Q8WX77	Insulin-like growth factor-binding protein-like 1	100	1.00.E-17
Q9H313-5	Isoform 5 of Protein tweety homolog 1	100	1.00.E-17
Q9H313-2	Isoform 2 of Protein tweety homolog 1	100	1.00.E-17
Q9Y6N8	Cadherin-10	100	1.00.E-17
Q12981-1	Isoform 3 of Vesicle transport protein SEC20	100	1.00.E-17
O76038	Secretagogin	100	1.00.E-17
Q86W92	Liprin-beta-1	100	1.00.E-17
Q9UMR5-3	Isoform 3 of Lysosomal thioesterase PPT2	100	1.00.E-17
Q9H7X7-2	Isoform 2 of Intraflagellar transport protein 22 homolog	100	1.00.E-17
Q12981	Vesicle transport protein SEC20	100	1.00.E-17
Q86W92-4	Isoform 4 of Liprin-beta-1	100	1.00.E-17
Q68CQ7-2	Isoform 2 of Glycosyltransferase 8 domain-containing protein 1	100	1.00.E-17
Q12981-2	Isoform 2 of Vesicle transport protein SEC20	100	1.00.E-17
Q12981-3	Isoform 4 of Vesicle transport protein SEC20	100	1.00.E-17
Q9C0E4	Glutamate receptor-interacting protein 2	100	1.00.E-17
Q68CQ7	Glycosyltransferase 8 domain-containing protein 1	100	1.00.E-17
Q13474	Dystrophin-related protein 2	100	1.00.E-17
Q9H313-3	Isoform 3 of Protein tweety homolog 1	100	1.00.E-17
P21980-3	Isoform 3 of Protein-glutamine gamma-glutamyltransferase 2	100	1.00.E-17
Q13474-2	Isoform 2 of Dystrophin-related protein 2	100	1.00.E-17
Q86W92-2	Isoform 2 of Liprin-beta-1	100	1.00.E-17
Q9UMR5	Lysosomal thioesterase PPT2	100	1.00.E-17
Q9HCM2	Plexin-A4	100	1.00.E-17
Q7RTP0	Magnesium transporter NIPA1	100	1.00.E-17
Q86W92-3	Isoform 3 of Liprin-beta-1	100	1.00.E-17
Q9ULB4	Cadherin-9	100	1.00.E-17
Q9H313	Protein tweety homolog 1	100	1.00.E-17
Q9H7X7	Intraflagellar transport protein 22 homolog	100	1.00.E-17
Q9C0E4-2	Isoform 2 of Glutamate receptor-interacting protein 2	100	1.00.E-17
Q9UMR5-2	Isoform 2 of Lysosomal thioesterase PPT2	100	1.00.E-17
Q6PI78	Transmembrane protein 65	100	1.00.E-17
P35080	Profilin-2	76.331	4.09.E-07
P09455	Retinol-binding protein 1	74.779	2.28.E-07
P09455-2	Isoform 2 of Retinol-binding protein 1	69.579	4.59.E-06
P09455-3	Isoform 3 of Retinol-binding protein 1	69.579	4.59.E-06
O00445	Synaptotagmin-5	58.424	2.02.E-05
O00445-2	Isoform 2 of Synaptotagmin-5	58.424	2.02.E-05
P08247	Synaptophysin	51.67	5.24.E-04
Q96FC7-2	Isoform 2 of Phytanoyl-CoA hydroxylase-interacting protein-like	46.438	3.22.E-04
P13637-2	Isoform 2 of Sodium/potassium-transporting ATPase subunit alpha-3	45.321	4.28.E-06
P13637-3	Isoform 3 of Sodium/potassium-transporting ATPase subunit alpha-3	45.321	4.28.E-06
P13637	Sodium/potassium-transporting ATPase subunit alpha-3	45.321	4.28.E-06
P17677-2	Isoform 2 of Neuromodulin	44.957	6.90.E-07
P17677	Neuromodulin	44.957	6.90.E-07
Q96MM6	Heat shock 70 kDa protein 12B	44.874	1.16.E-04
Q9H115	Beta-soluble NSF attachment protein	44.618	1.82.E-04
Q9H115-2	Isoform 2 of Beta-soluble NSF attachment protein	44.618	5.03.E-04
Q7L099-3	Isoform 3 of Protein RUFY3	44.023	9.26.E-06
Q7L099	Protein RUFY3	44.023	9.34.E-06
Q7L099-4	Isoform 4 of Protein RUFY3	44.023	4.91.E-06
O15075-2	Isoform 1 of Serine/threonine-protein kinase DCLK1	42.594	5.77.E-06
Q9P121-2	Isoform 2 of Neurotrimin	42.508	7.21.E-05
Q9P121-3	Isoform 3 of Neurotrimin	42.508	7.21.E-05
Q9P121-4	Isoform 4 of Neurotrimin	42.508	7.21.E-05
Q9P121	Neurotrimin	42.508	7.21.E-05
P14415	Sodium/potassium-transporting ATPase subunit beta-2	41.622	1.03.E-03
Q5SQI0-5	Isoform 5 of Alpha-tubulin N-acetyltransferase 1	39.505	1.10.E-03
Q5SQI0-4	Isoform 4 of Alpha-tubulin N-acetyltransferase 1	39.505	1.10.E-03
Q5SQI0	Alpha-tubulin N-acetyltransferase 1	39.505	1.10.E-03
Q5SQI0-2	Isoform 2 of Alpha-tubulin N-acetyltransferase 1	39.505	9.60.E-04
Q5SQI0-3	Isoform 3 of Alpha-tubulin N-acetyltransferase 1	39.505	7.92.E-04
Q5SQI0-7	Isoform 7 of Alpha-tubulin N-acetyltransferase 1	39.505	7.92.E-04
Q5SQI0-6	Isoform 6 of Alpha-tubulin N-acetyltransferase 1	39.505	7.92.E-04
Q06187-2	Isoform BTK-C of Tyrosine-protein kinase BTK	39.282	2.19.E-04

Q06187	Tyrosine-protein kinase BTK	39.282	2.19.E-04
P41217-3	Isoform 3 of OX-2 membrane glycoprotein	38.39	1.71.E-04
P41217	OX-2 membrane glycoprotein	38.39	1.71.E-04
P41217-2	Isoform 2 of OX-2 membrane glycoprotein	38.39	1.71.E-04
O15075-3	Isoform 3 of Serine/threonine-protein kinase DCLK1	37.117	1.72.E-05
P61266-2	Isoform 2 of Syntaxin-1B	35.447	7.51.E-05
P61266	Syntaxin-1B	35.447	7.51.E-05
Q04828	Aldo-keto reductase family 1 member C1	35.394	2.67.E-04
Q96FC7	Phytanoyl-CoA hydroxylase-interacting protein-like	34.771	3.46.E-05
Q7L099-2	Isoform 2 of Protein RUFY3	34.345	2.97.E-04
Q9UPY8-2	Isoform 2 of Microtubule-associated protein RP/EB family member 3	32.882	1.82.E-03
P11137-2	Isoform 2 of Microtubule-associated protein 2	32.577	2.98.E-05
Q2Y0W8-6	Isoform 6 of Electroneutral sodium bicarbonate exchanger 1	31.847	3.84.E-04
Q2Y0W8-8	Isoform 8 of Electroneutral sodium bicarbonate exchanger 1	31.847	3.84.E-04
Q2Y0W8-7	Isoform 7 of Electroneutral sodium bicarbonate exchanger 1	31.847	3.84.E-04
P29762	Cellular retinoic acid-binding protein 1	31.145	2.46.E-05
P52943	Cysteine-rich protein 2	31.042	9.91.E-04
P52943-2	Isoform 2 of Cysteine-rich protein 2	31.042	2.56.E-04
P42330	Aldo-keto reductase family 1 member C3	30.546	1.81.E-03
P62745	Rho-related GTP-binding protein RhoB	30.486	4.63.E-04
Q8TC26-2	Isoform 2 of Transmembrane protein 163	30.478	4.61.E-03
P59768	Guanine nucleotide-binding protein G(I)/G(S)/G(O) subunit gamma-2	30.318	5.29.E-04
P42330-2	Isoform 2 of Aldo-keto reductase family 1 member C3	29.986	1.20.E-04
Q9BPU6	Dihydropyrimidinase-related protein 5	29.586	3.09.E-05
Q2Y0W8	Electroneutral sodium bicarbonate exchanger 1	29.444	1.39.E-03
Q2Y0W8-3	Isoform 3 of Electroneutral sodium bicarbonate exchanger 1	29.444	1.39.E-03
Q2Y0W8-5	Isoform 5 of Electroneutral sodium bicarbonate exchanger 1	29.444	1.39.E-03
Q2Y0W8-2	Isoform 2 of Electroneutral sodium bicarbonate exchanger 1	29.444	1.39.E-03
Q2Y0W8-4	Isoform 4 of Electroneutral sodium bicarbonate exchanger 1	29.444	1.39.E-03
Q16799-3	Isoform RTN1-C of Reticulon-1	28.465	1.83.E-05
P07197	Neurofilament medium polypeptide	28.027	3.92.E-05
Q96MZ0-2	Isoform 2 of Ganglioside-induced differentiation-associated protein 1-like 1	27.573	2.50.E-03
Q8TC26	Transmembrane protein 163	27.403	3.88.E-03
P26232-3	Isoform 3 of Catenin alpha-2	27.084	4.16.E-05
Q9NZ53	Podocalyxin-like protein 2	26.759	4.37.E-04
P26232-6	Isoform 6 of Catenin alpha-2	26.438	9.29.E-05
P26232-4	Isoform 4 of Catenin alpha-2	26.438	1.64.E-04
P80404	4-aminobutyrate aminotransferase, mitochondrial	26.227	8.14.E-05
Q6ZTA4-2	Isoform 2 of Tripartite motif-containing protein 67	26.173	4.53.E-03
Q6ZTA4	Tripartite motif-containing protein 67	26.173	4.53.E-03
P51674-2	Isoform 2 of Neuronal membrane glycoprotein M6-a	26.137	6.17.E-05
P51674-3	Isoform 3 of Neuronal membrane glycoprotein M6-a	26.137	6.17.E-05
P26232-2	Isoform 2 of Catenin alpha-2	25.501	8.43.E-05
P26232-5	Isoform 5 of Catenin alpha-2	25.501	8.43.E-05
Q9UL26	Ras-related protein Rab-22A	25.17	4.68.E-04
P17516	Aldo-keto reductase family 1 member C4	24.693	1.64.E-04
P02768	Albumin	24.501	4.99.E-04
P02768-2	Isoform 2 of Albumin	24.501	4.99.E-04
P02768-3	Isoform 3 of Albumin	24.501	1.13.E-04
P07196	Neurofilament light polypeptide	23.743	7.98.E-05
Q99574	Neuroserpin	23.267	1.00.E-03
Q9H115-3	Isoform 3 of Beta-soluble NSF attachment protein	23.095	3.55.E-03
P56945-8	Isoform 8 of Breast cancer anti-estrogen resistance protein 1	23.024	1.38.E-03
P56945-4	Isoform 4 of Breast cancer anti-estrogen resistance protein 1	23.024	1.38.E-03
P56945-7	Isoform 7 of Breast cancer anti-estrogen resistance protein 1	23.024	1.38.E-03
P56945	Breast cancer anti-estrogen resistance protein 1	23.024	1.38.E-03
P56945-2	Isoform 2 of Breast cancer anti-estrogen resistance protein 1	23.024	1.38.E-03
P56945-3	Isoform 3 of Breast cancer anti-estrogen resistance protein 1	23.024	1.38.E-03
P56945-5	Isoform 5 of Breast cancer anti-estrogen resistance protein 1	23.024	1.38.E-03
P56945-6	Isoform 6 of Breast cancer anti-estrogen resistance protein 1	23.024	1.38.E-03
Q96PY5	Formin-like protein 2	22.865	1.06.E-02
O95466	Formin-like protein 1	22.865	1.06.E-02
O75914-4	Isoform 4 of Serine/threonine-protein kinase PAK 3	22.802	1.91.E-03
O75914-2	Isoform 2 of Serine/threonine-protein kinase PAK 3	22.802	1.91.E-03
O75914	Serine/threonine-protein kinase PAK 3	22.802	1.91.E-03
O75914-3	Isoform 3 of Serine/threonine-protein kinase PAK 3	22.802	1.91.E-03
Q8TBF2-3	Isoform 3 of Prostamide/prostaglandin F synthase	22.751	1.06.E-02

Q8TBF2-7	Isoform 6 of Prostamide/prostaglandin F synthase	22.751	1.06.E-02
Q8TBF2-6	Isoform 5 of Prostamide/prostaglandin F synthase	22.751	1.06.E-02
Q8TBF2	Prostamide/prostaglandin F synthase	22.751	1.06.E-02
Q8TBF2-4	Isoform 4 of Prostamide/prostaglandin F synthase	22.751	1.06.E-02
Q8TBF2-2	Isoform 2 of Prostamide/prostaglandin F synthase	22.751	1.06.E-02
P16870	Carboxypeptidase E	22.57	1.97.E-03
P16870-2	Isoform 2 of Carboxypeptidase E	22.57	1.97.E-03
Q9Y2S2-2	Isoform 2 of Lambda-crystallin homolog	22.404	2.98.E-03
Q9Y2S2	Lambda-crystallin homolog	22.404	2.98.E-03
Q96MZ0	Ganglioside-induced differentiation-associated protein 1-like 1	22.386	5.17.E-04
Q96MZ0-4	Isoform 3 of Ganglioside-induced differentiation-associated protein 1-like 1	22.386	5.17.E-04
Q9NQC3-2	Isoform B of Reticulon-4	21.845	4.74.E-03
P41732	Tetraspanin-7	21.829	2.48.E-03
O14531	Dihydropyrimidinase-related protein 4	21.515	4.17.E-05
P32004	Neural cell adhesion molecule L1	21.34	3.10.E-04
P32004-2	Isoform 2 of Neural cell adhesion molecule L1	21.34	3.11.E-04
P32004-3	Isoform 3 of Neural cell adhesion molecule L1	21.34	3.11.E-04
O15217	Glutathione S-transferase A4	21.275	5.52.E-03
Q9BR01	Sulfotransferase 4A1	21.053	1.62.E-03
Q9BR01-2	Isoform 2 of Sulfotransferase 4A1	21.053	1.62.E-03
P51674	Neuronal membrane glycoprotein M6-a	20.559	9.81.E-05
Q9Y2B0-2	Isoform 2 of Protein canopy homolog 2	20.52	1.29.E-02
Q9H4G0	Band 4.1-like protein 1	20.51	4.93.E-03
Q9H4G0-3	Isoform 3 of Band 4.1-like protein 1	20.51	3.61.E-03
Q9UPY8	Microtubule-associated protein RP/EB family member 3	19.529	1.58.E-03
P11137	Microtubule-associated protein 2	19.3	1.87.E-04
P11137-3	Isoform 3 of Microtubule-associated protein 2	19.3	1.87.E-04
P09471	Guanine nucleotide-binding protein G(o) subunit alpha	18.942	1.51.E-04
P09471-2	Isoform Alpha-2 of Guanine nucleotide-binding protein G(o) subunit alpha	18.942	1.51.E-04
Q7L775	EPM2A-interacting protein 1	18.775	2.55.E-04
P07197-2	Isoform 2 of Neurofilament medium polypeptide	18.724	2.11.E-04
P61764-2	Isoform 2 of Syntaxin-binding protein 1	18.579	2.21.E-04
P21579	Synaptotagmin-1	18.503	3.37.E-04
P22612	cAMP-dependent protein kinase catalytic subunit gamma	18.458	3.41.E-03
Q9ULP0-6	Isoform 6 of Protein NDRG4	17.719	6.42.E-04
Q9ULP0-3	Isoform 3 of Protein NDRG4	17.719	6.42.E-04
Q9ULP0-8	Isoform 8 of Protein NDRG4	17.719	4.47.E-04
Q9ULP0-7	Isoform 7 of Protein NDRG4	17.719	4.47.E-04
Q9UQ03-2	Isoform 2 of Coronin-2B	17.686	3.27.E-04
Q9UQ03	Coronin-2B	17.686	3.27.E-04
Q16623-2	Isoform 2 of Syntaxin-1A	17.379	2.26.E-03
Q16623	Syntaxin-1A	17.379	2.26.E-03
Q16623-3	Isoform 3 of Syntaxin-1A	17.379	2.26.E-03
Q86UW7-3	Isoform 3 of Calcium-dependent secretion activator 2	17.276	4.08.E-04
Q86UW7	Calcium-dependent secretion activator 2	17.276	4.08.E-04
Q86UW7-2	Isoform 2 of Calcium-dependent secretion activator 2	17.276	4.08.E-04
Q8N9I0	Synaptotagmin-2	17.034	9.24.E-03
Q75T13-2	Isoform 2 of GPI inositol-deacylase	16.776	7.02.E-03
Q14194-2	Isoform LCRMP-1 of Dihydropyrimidinase-related protein 1	16.587	3.39.E-04
Q6U841-4	Isoform 4 of Sodium-driven chloride bicarbonate exchanger	16.461	2.56.E-03
P08247-2	Isoform 2 of Synaptophysin	16.09	3.81.E-04
Q6ZVM7-2	Isoform 2 of TOM1-like protein 2	16.048	1.10.E-02
Q6ZVM7-4	Isoform 4 of TOM1-like protein 2	16.048	3.54.E-03
P61764	Syntaxin-binding protein 1	16.001	2.66.E-03
Q6ZVM7	TOM1-like protein 2	15.881	2.45.E-03
Q6ZVM7-5	Isoform 5 of TOM1-like protein 2	15.881	4.08.E-03
P17600	Synapsin-1	15.863	6.55.E-03
P17600-2	Isoform IB of Synapsin-1	15.863	6.55.E-03
Q9H492	Microtubule-associated proteins 1A/1B light chain 3A	15.818	1.28.E-02
Q9H492-2	Isoform 2 of Microtubule-associated proteins 1A/1B light chain 3A	15.818	1.28.E-02
P31150	Rab GDP dissociation inhibitor alpha	15.76	4.13.E-04
A1Z1Q3	ADP-ribose glycohydrolase MACROD2	15.738	2.97.E-03
A1Z1Q3-1	Isoform 1 of ADP-ribose glycohydrolase MACROD2	15.738	2.97.E-03
Q9BQT9-2	Isoform 2 of Calsyntenin-3	15.726	5.86.E-03
Q9BQT9	Calsyntenin-3	15.726	5.86.E-03
P46459-2	Isoform 2 of Vesicle-fusing ATPase	15.552	1.12.E-02
Q9H4G0-4	Isoform 4 of Band 4.1-like protein 1	15.303	7.67.E-03

Q9BW19	Kinesin-like protein KIFC1	14.73	7.68.E-04
Q9ULU8-5	Isoform 5 of Calcium-dependent secretion activator 1	14.714	1.68.E-03
Q9ULU8-4	Isoform 4 of Calcium-dependent secretion activator 1	14.708	4.39.E-03
Q9ULU8	Calcium-dependent secretion activator 1	14.708	4.39.E-03
Q9ULU8-3	Isoform 3 of Calcium-dependent secretion activator 1	14.708	4.75.E-03
Q9ULU8-2	Isoform 2 of Calcium-dependent secretion activator 1	14.708	4.75.E-03
O43295-2	Isoform 2 of SLIT-ROBO Rho GTPase-activating protein 3	14.673	4.48.E-03
O43295	SLIT-ROBO Rho GTPase-activating protein 3	14.673	5.20.E-03
Q14576-2	Isoform 2 of ELAV-like protein 3	14.628	8.81.E-04
Q75T13	GPI inositol-deacylase	14.495	1.69.E-02
Q6T4P5-4	Isoform 4 of Phospholipid phosphatase-related protein type 3	14.472	5.98.E-04
P63096-2	Isoform 2 of Guanine nucleotide-binding protein G(i) subunit alpha-1	14.366	9.86.E-03
P63096	Guanine nucleotide-binding protein G(i) subunit alpha-1	14.366	9.86.E-03
Q6ZVM7-3	Isoform 3 of TOM1-like protein 2	14.167	1.57.E-02
O15075	Serine/threonine-protein kinase DCLK1	14.107	3.64.E-03
O15075-4	Isoform 4 of Serine/threonine-protein kinase DCLK1	14.107	3.64.E-03
Q16799-2	Isoform RTN1-B of Reticulon-1	13.983	6.26.E-04
Q75T13-3	Isoform 3 of GPI inositol-deacylase	13.687	5.68.E-03
O60831	PRA1 family protein 2	13.63	8.80.E-04
Q96P47-6	Isoform 6 of Arf-GAP with GTPase, ANK repeat and PH domain-containing protein 3	13.579	6.08.E-03
Q96P47-3	Isoform 3 of Arf-GAP with GTPase, ANK repeat and PH domain-containing protein 3	13.579	6.08.E-03
P27816-3	Isoform 3 of Microtubule-associated protein 4	13.527	2.68.E-03
Q9HCM2-4	Isoform 4 of Plexin-A4	13.467	8.34.E-03
Q02410	Amyloid-beta A4 precursor protein-binding family A member 1	13.333	1.99.E-02
Q02410-2	Isoform 2 of Amyloid-beta A4 precursor protein-binding family A member 1	13.333	1.99.E-02
P31323	cAMP-dependent protein kinase type II-beta regulatory subunit	13.156	6.79.E-04
Q9Y2L6	FERM domain-containing protein 4B	13.142	3.69.E-02
Q9P2Q2	FERM domain-containing protein 4A	13.142	3.69.E-02
Q9Y2L6-2	Isoform 2 of FERM domain-containing protein 4B	13.142	3.69.E-02
P43004-2	Isoform 2 of Excitatory amino acid transporter 2	13.022	1.07.E-02
P43004	Excitatory amino acid transporter 2	13.022	1.07.E-02
P43004-3	Isoform 3 of Excitatory amino acid transporter 2	13.022	3.80.E-03
P06239-3	Isoform 3 of Tyrosine-protein kinase Lck	13.014	1.74.E-02
P06239	Tyrosine-protein kinase Lck	13.014	1.74.E-02
P51649-2	Isoform 2 of Succinate-semialdehyde dehydrogenase, mitochondrial	12.975	3.17.E-03
P51649	Succinate-semialdehyde dehydrogenase, mitochondrial	12.975	3.17.E-03
P21266	Glutathione S-transferase Mu 3	12.966	7.52.E-03
Q6T4P5-2	Isoform 2 of Phospholipid phosphatase-related protein type 3	12.939	1.21.E-02
Q6T4P5-3	Isoform 3 of Phospholipid phosphatase-related protein type 3	12.939	1.21.E-02
Q6T4P5	Phospholipid phosphatase-related protein type 3	12.939	1.21.E-02
P53801	Pituitary tumor-transforming gene 1 protein-interacting protein	12.933	2.43.E-02
Q0VDG4-2	Isoform 2 of Secernin-3	12.653	4.24.E-02
Q0VDG4	Secernin-3	12.653	4.24.E-02
Q9UK08	Guanine nucleotide-binding protein G(I)/G(S)/G(O) subunit gamma-8	12.361	1.30.E-02
O75061-3	Isoform 3 of Putative tyrosine-protein phosphatase auxilin	12.14	1.24.E-02
Q16799	Reticulon-1	11.885	1.05.E-03
O43295-3	Isoform 3 of SLIT-ROBO Rho GTPase-activating protein 3	11.864	1.34.E-02
Q8N9F7-3	Isoform 3 of Lysophospholipase D GDPD1	11.84	2.75.E-03
Q8N9F7-2	Isoform 2 of Lysophospholipase D GDPD1	11.84	2.75.E-03
Q13885	Tubulin beta-2A chain	11.483	1.33.E-03
Q8TAC9	Secretory carrier-associated membrane protein 5	11.473	6.51.E-03
Q8TAC9-2	Isoform 2 of Secretory carrier-associated membrane protein 5	11.473	6.51.E-03
Q9UH03-2	Isoform 2 of Neuronal-specific septin-3	11.465	3.01.E-02
Q9UH03	Neuronal-specific septin-3	11.465	3.01.E-02
P22694-10	Isoform 10 of cAMP-dependent protein kinase catalytic subunit beta	11.309	6.95.E-04
Q96SJ8	Tetraspanin-18	11.304	1.79.E-02
Q9UQ16-3	Isoform 3 of Dynamin-3	11.281	8.74.E-03
Q9UQ16	Dynamin-3	11.281	8.74.E-03
Q9UQ16-2	Isoform 2 of Dynamin-3	11.281	8.74.E-03
Q9UQ16-4	Isoform 4 of Dynamin-3	11.281	8.74.E-03
Q9UQ16-5	Isoform 5 of Dynamin-3	11.281	1.06.E-02
P40939-2	Isoform 2 of Trifunctional enzyme subunit alpha, mitochondrial	11.22	3.56.E-02
Q01814	Plasma membrane calcium-transporting ATPase 2	11.12	1.24.E-02
Q01814-6	Isoform ZB of Plasma membrane calcium-transporting ATPase 2	11.12	1.24.E-02
Q01814-3	Isoform YA of Plasma membrane calcium-transporting ATPase 2	11.12	1.24.E-02
Q01814-2	Isoform WA of Plasma membrane calcium-transporting ATPase 2	11.12	1.24.E-02
Q01814-8	Isoform XB of Plasma membrane calcium-transporting ATPase 2	11.12	1.24.E-02

Q01814-4	Isoform ZA of Plasma membrane calcium-transporting ATPase 2	11.12	1.24.E-02
Q01814-5	Isoform YB of Plasma membrane calcium-transporting ATPase 2	11.12	1.24.E-02
Q01814-7	Isoform XA of Plasma membrane calcium-transporting ATPase 2	11.12	1.24.E-02
Q9Y4B5-3	Isoform 3 of Microtubule cross-linking factor 1	10.889	1.62.E-02
Q9Y4B5	Microtubule cross-linking factor 1	10.889	1.62.E-02
Q9Y4B5-2	Isoform 2 of Microtubule cross-linking factor 1	10.889	1.62.E-02
Q8N5J2-4	Isoform 4 of Ubiquitin carboxyl-terminal hydrolase MINDY-1	10.868	3.78.E-02
Q8N5J2-2	Isoform 2 of Ubiquitin carboxyl-terminal hydrolase MINDY-1	10.868	3.78.E-02
Q8N5J2-3	Isoform 3 of Ubiquitin carboxyl-terminal hydrolase MINDY-1	10.868	3.78.E-02
Q8N5J2	Ubiquitin carboxyl-terminal hydrolase MINDY-1	10.868	3.78.E-02
Q9H3Z4	DnaJ homolog subfamily C member 5	10.857	2.58.E-02
Q9H3Z4-2	Isoform 2 of DnaJ homolog subfamily C member 5	10.857	3.91.E-03
Q8N9F7	Lysophospholipase D GDPD1	10.771	6.63.E-03
Q9H0Q3-2	Isoform 2 of FXYD domain-containing ion transport regulator 6	10.659	2.71.E-03
P20336	Ras-related protein Rab-3A	10.646	8.11.E-03
P0C7U0	Protein ELFN1	10.634	4.07.E-02
Q5R3F8	Protein phosphatase 1 regulatory subunit 29	10.634	4.07.E-02
Q9NQE9	Histidine triad nucleotide-binding protein 3	10.618	1.10.E-02
P08473	Neprilysin	10.616	1.79.E-02
P22694-3	Isoform 3 of cAMP-dependent protein kinase catalytic subunit beta	10.517	1.42.E-03
P22694-2	Isoform 2 of cAMP-dependent protein kinase catalytic subunit beta	10.517	1.42.E-03
P22694	cAMP-dependent protein kinase catalytic subunit beta	10.517	1.42.E-03
P22694-4	Isoform 4 of cAMP-dependent protein kinase catalytic subunit beta	10.517	1.42.E-03
Q9Y2Q0-3	Isoform 3 of Phospholipid-transporting ATPase IA	10.503	1.86.E-02
Q9Y2Q0	Phospholipid-transporting ATPase IA	10.503	2.40.E-02
Q9Y2Q0-2	Isoform 2 of Phospholipid-transporting ATPase IA	10.503	2.40.E-02
P26992	Ciliary neurotrophic factor receptor subunit alpha	10.496	6.83.E-03
P22694-7	Isoform 7 of cAMP-dependent protein kinase catalytic subunit beta	10.481	1.28.E-03
P22694-5	Isoform 5 of cAMP-dependent protein kinase catalytic subunit beta	10.481	1.28.E-03
Q9C0E8-4	Isoform 4 of Endoplasmic reticulum junction formation protein lunapark	10.446	2.38.E-02
Q9C0E8	Endoplasmic reticulum junction formation protein lunapark	10.446	2.38.E-02
Q9C0E8-2	Isoform 2 of Endoplasmic reticulum junction formation protein lunapark	10.446	2.38.E-02
Q9NQ86-4	Isoform 4 of E3 ubiquitin-protein ligase TRIM36	10.369	1.62.E-02
Q9NQ86	E3 ubiquitin-protein ligase TRIM36	10.369	1.62.E-02
O75054	Immunoglobulin superfamily member 3	10.36	2.76.E-02
P52799	Ephrin-B2	10.293	1.13.E-02
P63027	Vesicle-associated membrane protein 2	10.259	2.99.E-03
O94819	Kelch repeat and BTB domain-containing protein 11	10.253	8.14.E-03
Q8IXJ6-4	Isoform 4 of NAD-dependent protein deacetylase sirtuin-2	10.25	1.40.E-02
Q8IXJ6	NAD-dependent protein deacetylase sirtuin-2	10.25	1.40.E-02
Q8IXJ6-2	Isoform 2 of NAD-dependent protein deacetylase sirtuin-2	10.25	2.98.E-02
Q8IXJ6-3	Isoform 3 of NAD-dependent protein deacetylase sirtuin-2	10.25	2.98.E-02
Q8IXJ6-5	Isoform 5 of NAD-dependent protein deacetylase sirtuin-2	10.25	3.28.E-02
Q86Y82	Syntaxin-12	10.188	2.49.E-02
Q8IYB5-2	Isoform 2 of Stromal membrane-associated protein 1	10.064	1.37.E-02
Q8IYB5	Stromal membrane-associated protein 1	10.064	1.37.E-02
Q8IYB5-3	Isoform 3 of Stromal membrane-associated protein 1	10.064	1.37.E-02
Q9NQC3-6	Isoform D of Reticulon-4	9.935	2.17.E-03
Q13367-3	Isoform 3 of AP-3 complex subunit beta-2	9.864	9.52.E-03
Q13367	AP-3 complex subunit beta-2	9.864	9.52.E-03
Q13367-4	Isoform 4 of AP-3 complex subunit beta-2	9.864	9.52.E-03
Q5T4D3-4	Isoform 4 of Protein O-mannosyl-transferase TMTC4	9.678	4.50.E-02
Q5T4D3	Protein O-mannosyl-transferase TMTC4	9.678	4.50.E-02
Q5T4D3-2	Isoform 2 of Protein O-mannosyl-transferase TMTC4	9.678	4.50.E-02
Q5T4D3-3	Isoform 3 of Protein O-mannosyl-transferase TMTC4	9.678	4.50.E-02
P22694-8	Isoform 8 of cAMP-dependent protein kinase catalytic subunit beta	9.585	1.23.E-02
Q9P0M6	Core histone macro-H2A.2	9.535	4.37.E-02
O75061-2	Isoform 2 of Putative tyrosine-protein phosphatase auxilin	9.457	1.03.E-02
O75061-4	Isoform 4 of Putative tyrosine-protein phosphatase auxilin	9.457	1.03.E-02
O75061	Putative tyrosine-protein phosphatase auxilin	9.457	1.03.E-02
Q9C0E8-3	Isoform 3 of Endoplasmic reticulum junction formation protein lunapark	9.431	3.42.E-02
Q9NQC3-4	Isoform 6 of Reticulon-4	9.42	2.59.E-03
Q9BV23	Monoacylglycerol lipase ABHD6	9.358	1.91.E-02
P17612-2	Isoform 2 of cAMP-dependent protein kinase catalytic subunit alpha	9.292	1.48.E-02
P17612	cAMP-dependent protein kinase catalytic subunit alpha	9.292	1.48.E-02
Q96JE9-2	Isoform 2 of Microtubule-associated protein 6	9.254	1.90.E-02
Q9ULK5	Vang-like protein 2	9.252	1.57.E-02

Q05193	Dynamin-1	9.157	2.34.E-02
Q05193-3	Isoform 3 of Dynamin-1	9.157	2.34.E-02
Q5TF21	Protein SOGA3	9.07	1.93.E-02
P98172	Ephrin-B1	8.993	3.35.E-02
Q9UIW2	Plexin-A1	8.927	9.31.E-03
Q9H0Q3	FXYD domain-containing ion transport regulator 6	8.912	3.30.E-02
Q53FP2	Transmembrane protein 35A	8.908	9.66.E-03
Q9NQC3	Reticulon-4	8.891	3.13.E-03
Q9Y2B9	cAMP-dependent protein kinase inhibitor gamma	8.881	1.49.E-02
O75054-2	Isoform 2 of Immunoglobulin superfamily member 3	8.851	3.29.E-02
Q9Y281	Cofilin-2	8.817	1.41.E-02
Q9Y281-3	Isoform 3 of Cofilin-2	8.817	1.41.E-02
P29317-2	Isoform 2 of Ephrin type-A receptor 2	8.807	1.53.E-02
Q8TB36	Ganglioside-induced differentiation-associated protein 1	8.793	1.17.E-02
Q8TB36-2	Isoform 2 of Ganglioside-induced differentiation-associated protein 1	8.793	8.00.E-03
P05129	Protein kinase C gamma type	8.77	2.20.E-02
P05129-2	Isoform 2 of Protein kinase C gamma type	8.77	1.51.E-02
O75962-2	Isoform 2 of Triple functional domain protein	8.691	3.60.E-02
Q3SXM5	Inactive hydroxysteroid dehydrogenase-like protein 1	8.655	5.27.E-03
Q9H7P6	Multivesicular body subunit 12B	8.627	1.58.E-02
Q9H7P6-2	Isoform 2 of Multivesicular body subunit 12B	8.627	1.42.E-02
Q9NT99	Leucine-rich repeat-containing protein 4B	8.577	7.18.E-03
Q05193-2	Isoform 2 of Dynamin-1	8.575	2.27.E-02
Q05193-5	Isoform 4 of Dynamin-1	8.575	2.27.E-02
Q8WXF7	Atlastin-1	8.561	3.89.E-02
Q8WXF7-2	Isoform 2 of Atlastin-1	8.561	3.56.E-02
Q9UIJ7-3	Isoform 3 of GTP:AMP phosphotransferase AK3, mitochondrial	8.54	7.48.E-03
Q9UIJ7	GTP:AMP phosphotransferase AK3, mitochondrial	8.54	7.48.E-03
O15394	Neural cell adhesion molecule 2	8.496	3.76.E-02
O15394-2	Isoform 2 of Neural cell adhesion molecule 2	8.496	1.61.E-02
Q14576	ELAV-like protein 3	8.49	6.30.E-03
Q86YR5-3	Isoform 3 of G-protein-signaling modulator 1	8.481	3.58.E-02
Q9Y4I1-3	Isoform 3 of Unconventional myosin-Va	8.48	7.52.E-03
Q9Y4I1	Unconventional myosin-Va	8.48	7.52.E-03
Q9Y4I1-2	Isoform 2 of Unconventional myosin-Va	8.48	7.52.E-03
Q86VH2-3	Isoform 3 of Kinesin-like protein KIF27	8.453	1.34.E-02
Q86VH2	Kinesin-like protein KIF27	8.453	1.34.E-02
Q86VH2-2	Isoform 2 of Kinesin-like protein KIF27	8.453	1.34.E-02
Q2M1P5	Kinesin-like protein KIF7	8.453	1.34.E-02
Q13554-6	Isoform 6 of Calcium/calmodulin-dependent protein kinase type II subunit beta	8.412	1.55.E-02
O60462-2	Isoform A0 of Neuropilin-2	8.39	7.55.E-03
O60462	Neuropilin-2	8.39	7.55.E-03
O60462-3	Isoform A17 of Neuropilin-2	8.39	7.55.E-03
O60462-5	Isoform B5 of Neuropilin-2	8.39	1.55.E-02
O60462-4	Isoform B0 of Neuropilin-2	8.39	1.55.E-02
O75962-4	Isoform 4 of Triple functional domain protein	8.289	3.84.E-02
Q8TAC9-3	Isoform 3 of Secretory carrier-associated membrane protein 5	8.281	3.12.E-02
Q9H1E5	Thioredoxin-related transmembrane protein 4	8.26	7.25.E-03
Q96FJ2	Dynein light chain 2, cytoplasmic	8.219	6.32.E-03
P49418	Amphiphysin	8.214	1.82.E-02
P49418-2	Isoform 2 of Amphiphysin	8.214	1.96.E-02
O75962	Triple functional domain protein	8.214	3.92.E-02
O75962-5	Isoform 5 of Triple functional domain protein	8.214	3.92.E-02
P34903	Gamma-aminobutyric acid receptor subunit alpha-3	8.203	2.08.E-02
Q14108-2	Isoform 2 of Lysosome membrane protein 2	8.123	5.20.E-03
Q8TCZ2-2	Isoform 2 of CD99 antigen-like protein 2	8.119	2.52.E-02
Q8TCZ2	CD99 antigen-like protein 2	8.119	2.52.E-02
Q8TCZ2-5	Isoform 5 of CD99 antigen-like protein 2	8.119	2.52.E-02
Q8TCZ2-3	Isoform 3 of CD99 antigen-like protein 2	8.119	2.52.E-02
A6NHL2-2	Isoform 2 of Tubulin alpha chain-like 3	8.11	4.22.E-03
A6NHL2	Tubulin alpha chain-like 3	8.11	4.22.E-03
Q9BY67-2	Isoform 2 of Cell adhesion molecule 1	7.929	7.07.E-03
Q3SXM5-2	Isoform 2 of Inactive hydroxysteroid dehydrogenase-like protein 1	7.91	1.30.E-02
P53677	AP-3 complex subunit mu-2	7.9	3.85.E-02
P53677-2	Isoform 2 of AP-3 complex subunit mu-2	7.9	3.85.E-02
Q86YR5-4	Isoform 4 of G-protein-signaling modulator 1	7.878	2.80.E-02
Q86YR5	G-protein-signaling modulator 1	7.878	2.80.E-02

Q14108	Lysosome membrane protein 2	7.871	5.57.E-03
P26232	Catenin alpha-2	7.87	1.99.E-02
Q8TB96	T-cell immunomodulatory protein	7.841	2.41.E-02
Q75T13-4	Isoform 4 of GPI inositol-deacylase	7.802	2.40.E-02
Q9NTI5-5	Isoform 5 of Sister chromatid cohesion protein PDS5 homolog B	7.776	1.71.E-02
P04350	Tubulin beta-4A chain	7.75	4.87.E-03
O75911	Short-chain dehydrogenase/reductase 3	7.711	2.66.E-02
Q16775-2	Isoform 2 of Hydroxyacylglutathione hydrolase, mitochondrial	7.677	2.08.E-02
Q16775	Hydroxyacylglutathione hydrolase, mitochondrial	7.677	2.08.E-02
P84074	Neuron-specific calcium-binding protein hippocalcin	7.612	1.45.E-02
Q6SZW1	NAD(+) hydrolase SARM1	7.562	3.58.E-02
O94967	WD repeat-containing protein 47	7.519	2.48.E-02
O94967-2	Isoform 2 of WD repeat-containing protein 47	7.519	2.48.E-02
O94967-4	Isoform 4 of WD repeat-containing protein 47	7.519	4.92.E-02
O94967-3	Isoform 3 of WD repeat-containing protein 47	7.519	4.92.E-02
P84157-2	Isoform 2 of Matrix-remodeling-associated protein 7	7.498	4.81.E-02
P84157	Matrix-remodeling-associated protein 7	7.498	4.81.E-02
P84157-3	Isoform 3 of Matrix-remodeling-associated protein 7	7.498	4.81.E-02
P61925	cAMP-dependent protein kinase inhibitor alpha	7.458	8.66.E-03
Q8N158	Glypican-2	7.408	2.96.E-02
P51513-5	Isoform 3 of RNA-binding protein Nova-1	7.356	7.24.E-03
P51513	RNA-binding protein Nova-1	7.356	7.24.E-03
O14994	Synapsin-3	7.352	3.07.E-02
Q12926	ELAV-like protein 2	7.335	1.11.E-02
Q12926-2	Isoform 2 of ELAV-like protein 2	7.335	1.28.E-02
Q8NC96-2	Isoform 2 of Adaptin ear-binding coat-associated protein 1	7.323	3.10.E-02
Q16854	Deoxyguanosine kinase, mitochondrial	7.318	2.97.E-02
Q16854-2	Isoform 2 of Deoxyguanosine kinase, mitochondrial	7.318	2.97.E-02
Q13449	Limbic system-associated membrane protein	7.31	3.01.E-02
Q8N126	Cell adhesion molecule 3	7.307	2.28.E-02
Q8N126-2	Isoform 2 of Cell adhesion molecule 3	7.307	2.28.E-02
Q8N126-3	Isoform 3 of Cell adhesion molecule 3	7.307	2.66.E-02
Q8WY54	Protein phosphatase 1E	7.273	1.46.E-02
P51513-2	Isoform 2 of RNA-binding protein Nova-1	7.25	2.54.E-02
Q9NUJ1-3	Isoform 3 of Mycophenolic acid acyl-glucuronide esterase, mitochondrial	7.223	2.74.E-02
O94805	Actin-like protein 6B	7.205	3.04.E-02
P00568	Adenylate kinase isoenzyme 1	7.184	1.20.E-02
Q9GZQ8	Microtubule-associated proteins 1A/1B light chain 3B	7.179	5.84.E-03
A6NCE7	Microtubule-associated proteins 1A/1B light chain 3 beta 2	7.179	5.84.E-03
O00562	Membrane-associated phosphatidylinositol transfer protein 1	7.118	2.13.E-02
O00562-2	Isoform 2 of Membrane-associated phosphatidylinositol transfer protein 1	7.118	2.13.E-02
O60282	Kinesin heavy chain isoform 5C	7.079	6.44.E-03
Q92903	Phosphatidate cytidylyltransferase 1	7.066	4.04.E-02
O43854-2	Isoform 2 of EGF-like repeat and discoidin I-like domain-containing protein 3	7.054	1.07.E-02
O43854	EGF-like repeat and discoidin I-like domain-containing protein 3	7.054	1.07.E-02
O60282-2	Isoform 2 of Kinesin heavy chain isoform 5C	7.039	6.55.E-03
P20020-1	Isoform D of Plasma membrane calcium-transporting ATPase 1	6.98	8.90.E-03
P20020-4	Isoform C of Plasma membrane calcium-transporting ATPase 1	6.98	8.90.E-03
P20020	Plasma membrane calcium-transporting ATPase 1	6.98	8.06.E-03
P20020-6	Isoform K of Plasma membrane calcium-transporting ATPase 1	6.98	8.06.E-03
Q7Z2K8	G protein-regulated inducer of neurite outgrowth 1	6.957	1.31.E-02
Q96RD7	Pannexin-1	6.954	1.05.E-02
Q96RD7-2	Isoform 2 of Pannexin-1	6.923	4.34.E-02
P12235	ADP/ATP translocase 1	6.916	3.39.E-02
Q7L0J3	Synaptic vesicle glycoprotein 2A	6.884	2.38.E-02
Q14168-4	Isoform 4 of MAGUK p55 subfamily member 2	6.849	3.93.E-02
Q14168-2	Isoform 2 of MAGUK p55 subfamily member 2	6.849	3.93.E-02
Q14168	MAGUK p55 subfamily member 2	6.849	3.93.E-02
Q14168-3	Isoform 3 of MAGUK p55 subfamily member 2	6.849	3.93.E-02
Q14168-6	Isoform 6 of MAGUK p55 subfamily member 2	6.849	3.93.E-02
Q96PU5-3	Isoform 3 of E3 ubiquitin-protein ligase NEDD4-like	6.848	1.92.E-02
Q96PU5-9	Isoform 8 of E3 ubiquitin-protein ligase NEDD4-like	6.848	2.94.E-02
Q96PU5-4	Isoform 4 of E3 ubiquitin-protein ligase NEDD4-like	6.848	2.70.E-02
Q9Y243	RAC-gamma serine/threonine-protein kinase	6.846	2.39.E-02
Q9Y243-2	Isoform 2 of RAC-gamma serine/threonine-protein kinase	6.846	2.39.E-02
P37235	Hippocalcin-like protein 1	6.835	3.96.E-02
O95989	Diphosphoinositol polyphosphate phosphohydrolase 1	6.792	2.17.E-02

Q96GQ5	RUS family member 1	6.782	3.55.E-02
P20020-2	Isoform A of Plasma membrane calcium-transporting ATPase 1	6.78	9.70.E-03
P20020-5	Isoform E of Plasma membrane calcium-transporting ATPase 1	6.78	9.70.E-03
Q9BRK0	Receptor expression-enhancing protein 2	6.768	3.80.E-02
Q9BRK0-2	Isoform 2 of Receptor expression-enhancing protein 2	6.768	3.80.E-02
O14786-3	Isoform 3 of Neuropilin-1	6.754	2.76.E-02
O14786	Neuropilin-1	6.754	2.76.E-02
O14786-2	Isoform 2 of Neuropilin-1	6.754	2.76.E-02
P05026-2	Isoform 2 of Sodium/potassium-transporting ATPase subunit beta-1	6.753	8.83.E-03
P05026	Sodium/potassium-transporting ATPase subunit beta-1	6.753	8.83.E-03
Q99755-2	Isoform 2 of Phosphatidylinositol 4-phosphate 5-kinase type-1 alpha	6.749	3.48.E-02
Q13557-4	Isoform Delta 4 of Calcium/calmodulin-dependent protein kinase type II subunit delta	6.738	1.96.E-02
Q13557-3	Isoform Delta 3 of Calcium/calmodulin-dependent protein kinase type II subunit delta	6.738	1.96.E-02
Q13557-9	Isoform Delta 7 of Calcium/calmodulin-dependent protein kinase type II subunit delta	6.738	1.96.E-02
Q13557-5	Isoform Delta 8 of Calcium/calmodulin-dependent protein kinase type II subunit delta	6.738	1.96.E-02
Q96PU5-5	Isoform 5 of E3 ubiquitin-protein ligase NEDD4-like	6.727	1.64.E-02
Q96PU5-6	Isoform 6 of E3 ubiquitin-protein ligase NEDD4-like	6.727	1.58.E-02
Q96PU5-2	Isoform 2 of E3 ubiquitin-protein ligase NEDD4-like	6.727	1.60.E-02
Q96PU5	E3 ubiquitin-protein ligase NEDD4-like	6.727	1.60.E-02
Q96PU5-7	Isoform 7 of E3 ubiquitin-protein ligase NEDD4-like	6.727	1.63.E-02
P46821	Microtubule-associated protein 1B	6.714	7.56.E-03
Q9NR96-4	Isoform 4 of Toll-like receptor 9	6.691	2.44.E-02
Q7L0J3-2	Isoform 2 of Synaptic vesicle glycoprotein 2A	6.684	3.05.E-02
Q8NC96	Adaptin ear-binding coat-associated protein 1	6.645	3.51.E-02
P12036	Neurofilament heavy polypeptide	6.641	1.02.E-02
P12036-2	Isoform 2 of Neurofilament heavy polypeptide	6.641	1.02.E-02
Q9UI08-3	Isoform 3 of Ena/VASP-like protein	6.555	4.13.E-02
Q9UI08-4	Isoform 4 of Ena/VASP-like protein	6.555	4.13.E-02
P78356-2	Isoform 2 of Phosphatidylinositol 5-phosphate 4-kinase type-2 beta	6.536	1.33.E-02
Q9BY67-3	Isoform 3 of Cell adhesion molecule 1	6.528	1.14.E-02
Q9BY67-4	Isoform 4 of Cell adhesion molecule 1	6.528	1.14.E-02
Q9BY67	Cell adhesion molecule 1	6.528	1.14.E-02
Q9BY67-5	Isoform 5 of Cell adhesion molecule 1	6.528	1.14.E-02
Q9Y4J8-7	Isoform 7 of Dystrobrevin alpha	6.519	2.85.E-02
Q9Y4J8-9	Isoform 9 of Dystrobrevin alpha	6.519	2.85.E-02
Q13557-10	Isoform Delta 10 of Calcium/calmodulin-dependent protein kinase type II subunit delta	6.456	2.28.E-02
Q13557-6	Isoform Delta 9 of Calcium/calmodulin-dependent protein kinase type II subunit delta	6.456	2.28.E-02
Q13557-11	Isoform Delta 11 of Calcium/calmodulin-dependent protein kinase type II subunit delta	6.456	2.36.E-02
Q13557	Calcium/calmodulin-dependent protein kinase type II subunit delta	6.456	2.36.E-02
Q13557-12	Isoform Delta 12 of Calcium/calmodulin-dependent protein kinase type II subunit delta	6.456	2.36.E-02
Q13557-8	Isoform Delta 6 of Calcium/calmodulin-dependent protein kinase type II subunit delta	6.456	2.36.E-02
Q9UPZ6	Thrombospondin type-1 domain-containing protein 7A	6.41	2.82.E-02
Q8TDJ6-2	Isoform 2 of DmX-like protein 2	6.404	4.34.E-02
P07203-2	Isoform 2 of Glutathione peroxidase 1	6.391	3.85.E-02
O95057	GTP-binding protein Di-Ras1	6.374	4.46.E-02
Q9UBH6	Xenotropic and polytropic retrovirus receptor 1	6.334	1.34.E-02
O60462-6	Isoform s9 of Neuropilin-2	6.333	3.77.E-02
Q13423	NAD(P) transhydrogenase, mitochondrial	6.309	4.44.E-02
Q9UBH6-2	Isoform 2 of Xenotropic and polytropic retrovirus receptor 1	6.268	1.72.E-02
O60229-2	Isoform 2 of Kalirin	6.262	3.10.E-02
O75915	PRA1 family protein 3	6.255	2.97.E-02
O95837	Guanine nucleotide-binding protein subunit alpha-14	6.239	3.07.E-02
Q96P47	Arf-GAP with GTPase, ANK repeat and PH domain-containing protein 3	6.204	4.68.E-02
Q96P47-4	Isoform 4 of Arf-GAP with GTPase, ANK repeat and PH domain-containing protein 3	6.204	4.68.E-02
Q96P47-2	Isoform 2 of Arf-GAP with GTPase, ANK repeat and PH domain-containing protein 3	6.204	4.68.E-02
Q15286-2	Isoform 2 of Ras-related protein Rab-35	6.187	2.91.E-02
Q07866-4	Isoform J of Kinesin light chain 1	6.175	9.69.E-03
Q07866-5	Isoform K of Kinesin light chain 1	6.175	9.69.E-03
Q07866-10	Isoform D of Kinesin light chain 1	6.175	9.69.E-03
Q07866-9	Isoform I of Kinesin light chain 1	6.175	9.69.E-03
Q07866-2	Isoform C of Kinesin light chain 1	6.175	9.69.E-03
Q07866	Kinesin light chain 1	6.175	9.69.E-03
Q07866-7	Isoform P of Kinesin light chain 1	6.175	9.69.E-03
Q07866-6	Isoform N of Kinesin light chain 1	6.175	9.69.E-03
Q6PCE3	Glucose 1,6-bisphosphate synthase	6.149	9.92.E-03
P50148	Guanine nucleotide-binding protein G(q) subunit alpha	6.136	2.07.E-02
Q9H0Q0	CYFIP-related Rac1 interactor A	6.118	4.13.E-02

P15848	Arylsulfatase B	6.076	4.42.E-02
Q07866-3	Isoform G of Kinesin light chain 1	6.051	1.03.E-02
Q13491-4	Isoform 4 of Neuronal membrane glycoprotein M6-b	5.999	4.99.E-02
P50583	Bis(5'-nucleosyl)-tetraphosphatase [asymmetrical]	5.983	1.83.E-02
Q9H598	Vesicular inhibitory amino acid transporter	5.952	3.77.E-02
Q96GZ6	Solute carrier family 41 member 3	5.933	4.70.E-02
Q96GZ6-9	Isoform 9 of Solute carrier family 41 member 3	5.933	4.70.E-02
Q96GZ6-5	Isoform 5 of Solute carrier family 41 member 3	5.933	4.70.E-02
Q96GZ6-7	Isoform 7 of Solute carrier family 41 member 3	5.933	4.70.E-02
Q96GZ6-6	Isoform 6 of Solute carrier family 41 member 3	5.933	4.70.E-02
Q96GZ6-2	Isoform 2 of Solute carrier family 41 member 3	5.933	4.70.E-02
O76062	Delta(14)-sterol reductase TM7SF2	5.83	4.95.E-02
O76062-2	Isoform 2 of Delta(14)-sterol reductase TM7SF2	5.83	4.95.E-02
O75884	Serine hydrolase RBBP9	5.816	1.57.E-02
O43237	Cytoplasmic dynein 1 light intermediate chain 2	5.808	1.75.E-02
O95865	N(G),N(G)-dimethylarginine dimethylaminohydrolase 2	5.807	3.62.E-02
Q92614-5	Isoform 5 of Unconventional myosin-XVIIIa	5.767	2.30.E-02
Q9BWM7	Sideroflexin-3	5.749	1.88.E-02
Q9NUJ1	Mycophenolic acid acyl-glucuronide esterase, mitochondrial	5.736	2.43.E-02
P29992	Guanine nucleotide-binding protein subunit alpha-11	5.727	1.69.E-02
Q9NUJ1-2	Isoform 2 of Mycophenolic acid acyl-glucuronide esterase, mitochondrial	5.691	2.69.E-02
Q96GQ5-2	Isoform 2 of RUS family member 1	5.666	4.64.E-02
P46459	Vesicle-fusing ATPase	5.653	1.25.E-02
Q9UPT6	C-Jun-amino-terminal kinase-interacting protein 3	5.641	4.85.E-02
Q92558	Wiskott-Aldrich syndrome protein family member 1	5.633	1.54.E-02
Q5JWF2	Guanine nucleotide-binding protein G(s) subunit alpha isoforms XLas	5.622	1.04.E-02
P63092-4	Isoform 4 of Guanine nucleotide-binding protein G(s) subunit alpha isoforms short	5.622	1.04.E-02
P63092	Guanine nucleotide-binding protein G(s) subunit alpha isoforms short	5.622	1.04.E-02
Q92859	Neogenin	5.622	2.47.E-02
Q92859-4	Isoform 4 of Neogenin	5.622	2.47.E-02
Q92859-3	Isoform 3 of Neogenin	5.622	2.47.E-02
Q92859-2	Isoform 2 of Neogenin	5.622	2.47.E-02
O43861-2	Isoform 2 of Probable phospholipid-transporting ATPase IIB	5.612	3.70.E-02
O43861	Probable phospholipid-transporting ATPase IIB	5.612	3.70.E-02
O43301	Heat shock 70 kDa protein 12A	5.606	1.28.E-02
Q59EK9-2	Isoform 2 of RUN domain-containing protein 3A	5.571	3.92.E-02
Q59EK9-3	Isoform 3 of RUN domain-containing protein 3A	5.571	3.92.E-02
Q59EK9	RUN domain-containing protein 3A	5.571	3.92.E-02
Q59EK9-4	Isoform 4 of RUN domain-containing protein 3A	5.571	3.92.E-02
Q9UQB3	Catenin delta-2	5.559	2.05.E-02
Q9UQB3-2	Isoform 2 of Catenin delta-2	5.559	2.10.E-02
Q7Z2K8-2	Isoform 2 of G protein-regulated inducer of neurite outgrowth 1	5.548	1.96.E-02
P63092-3	Isoform 3 of Guanine nucleotide-binding protein G(s) subunit alpha isoforms short	5.5	1.47.E-02
Q5JWF2-2	Isoform XLas-2 of Guanine nucleotide-binding protein G(s) subunit alpha isoforms XLas	5.5	1.47.E-02
P63092-2	Isoform Gnas-2 of Guanine nucleotide-binding protein G(s) subunit alpha isoforms short	5.5	1.47.E-02
O43237-2	Isoform 2 of Cytoplasmic dynein 1 light intermediate chain 2	5.479	2.85.E-02
Q13555-8	Isoform 8 of Calcium/calmodulin-dependent protein kinase type II subunit gamma	5.444	2.51.E-02
Q13555-11	Isoform 11 of Calcium/calmodulin-dependent protein kinase type II subunit gamma	5.444	2.51.E-02
Q13555-4	Isoform 4 of Calcium/calmodulin-dependent protein kinase type II subunit gamma	5.444	2.51.E-02
Q13555-5	Isoform 5 of Calcium/calmodulin-dependent protein kinase type II subunit gamma	5.444	2.51.E-02
Q13555-7	Isoform 7 of Calcium/calmodulin-dependent protein kinase type II subunit gamma	5.444	2.51.E-02
Q13555	Calcium/calmodulin-dependent protein kinase type II subunit gamma	5.444	2.51.E-02
Q13555-10	Isoform 10 of Calcium/calmodulin-dependent protein kinase type II subunit gamma	5.444	2.51.E-02
Q13555-2	Isoform 2 of Calcium/calmodulin-dependent protein kinase type II subunit gamma	5.444	2.51.E-02
Q13555-6	Isoform 6 of Calcium/calmodulin-dependent protein kinase type II subunit gamma	5.444	2.51.E-02
Q13555-9	Isoform 9 of Calcium/calmodulin-dependent protein kinase type II subunit gamma	5.444	2.51.E-02
Q13555-3	Isoform 3 of Calcium/calmodulin-dependent protein kinase type II subunit gamma	5.444	2.51.E-02
Q9Y625	Glypican-6	5.426	4.40.E-02
O43310	CBP80/20-dependent translation initiation factor	5.393	4.43.E-02
O43310-2	Isoform 2 of CBP80/20-dependent translation initiation factor	5.393	4.43.E-02
O75884-2	Isoform 2 of Serine hydrolase RBBP9	5.389	2.09.E-02
Q92614	Unconventional myosin-XVIIIa	5.313	2.40.E-02
Q92614-4	Isoform 4 of Unconventional myosin-XVIIIa	5.313	2.40.E-02
Q8WZA9	Immunity-related GTPase family Q protein	5.306	2.37.E-02
Q92614-3	Isoform 3 of Unconventional myosin-XVIIIa	5.298	2.57.E-02
P48735-2	Isoform 2 of Isocitrate dehydrogenase [NADP], mitochondrial	5.19	1.59.E-02
P48735	Isocitrate dehydrogenase [NADP], mitochondrial	5.19	1.59.E-02

P35612-9	Isoform 9 of Beta-adducin	5.143	1.19.E-02
P35612-2	Isoform 2 of Beta-adducin	5.143	1.19.E-02
P35612-8	Isoform 8 of Beta-adducin	5.143	1.19.E-02
Q9NX46	ADP-ribose glycohydrolase ARH3	5.109	4.47.E-02
Q9Y2H0-1	Isoform 2 of Disks large-associated protein 4	5.108	4.60.E-02
Q9Y2H0-3	Isoform 3 of Disks large-associated protein 4	5.108	4.60.E-02
Q9Y2H0	Disks large-associated protein 4	5.108	4.60.E-02
Q15286	Ras-related protein Rab-35	5.041	3.95.E-02
Q92614-2	Isoform 2 of Unconventional myosin-XVIIIa	5.034	3.39.E-02
Q6QEF8-3	Isoform 3 of Coronin-6	5.027	2.46.E-02
Q6QEF8	Coronin-6	5.027	2.46.E-02
Q6QEF8-5	Isoform 5 of Coronin-6	5.027	2.46.E-02
Q6QEF8-4	Isoform 4 of Coronin-6	5.027	2.46.E-02
Q6QEF8-2	Isoform 2 of Coronin-6	5.027	2.46.E-02
Q8WUD1	Ras-related protein Rab-2B	5.002	2.67.E-02
Q8WUD1-2	Isoform 2 of Ras-related protein Rab-2B	5.002	2.67.E-02
O95197-5	Isoform 5 of Reticulon-3	4.99	4.93.E-02
O95197-3	Isoform 3 of Reticulon-3	4.99	4.93.E-02
O95197-6	Isoform 6 of Reticulon-3	4.99	4.93.E-02
P35612-4	Isoform 4 of Beta-adducin	4.978	1.62.E-02
P35612-3	Isoform 3 of Beta-adducin	4.978	1.62.E-02
Q92823-6	Isoform 6 of Neuronal cell adhesion molecule	4.965	2.54.E-02
Q92823-3	Isoform 3 of Neuronal cell adhesion molecule	4.965	2.54.E-02
Q92823	Neuronal cell adhesion molecule	4.965	2.54.E-02
Q92823-5	Isoform 5 of Neuronal cell adhesion molecule	4.965	2.54.E-02
Q92823-2	Isoform 2 of Neuronal cell adhesion molecule	4.965	2.54.E-02
Q92823-4	Isoform 4 of Neuronal cell adhesion molecule	4.965	2.54.E-02
Q9NRX5	Serine incorporator 1	4.947	4.37.E-02
Q5SW79-2	Isoform 2 of Centrosomal protein of 170 kDa	4.887	2.31.E-02
Q5SW79-3	Isoform 3 of Centrosomal protein of 170 kDa	4.887	2.31.E-02
P46939-4	Isoform Up140 of Utrophin	4.875	3.74.E-02
Q9ULH0-2	Isoform 2 of Kinase D-interacting substrate of 220 kDa	4.835	3.28.E-02
Q9NZN3-2	Isoform 2 of EH domain-containing protein 3	4.834	4.64.E-02
Q15126	Phosphomevalonate kinase	4.821	3.08.E-02
Q9UHQ9	NADH-cytochrome b5 reductase 1	4.813	2.39.E-02
Q9ULH0-3	Isoform 3 of Kinase D-interacting substrate of 220 kDa	4.811	3.02.E-02
O75110	Probable phospholipid-transporting ATPase IIA	4.699	3.01.E-02
Q9ULV0	Unconventional myosin-Vb	4.68	2.75.E-02
P61019	Ras-related protein Rab-2A	4.674	2.65.E-02
Q9ULH0-4	Isoform 4 of Kinase D-interacting substrate of 220 kDa	4.633	3.34.E-02
Q9ULH0	Kinase D-interacting substrate of 220 kDa	4.633	3.34.E-02
P38405	Guanine nucleotide-binding protein G(olf) subunit alpha	4.632	3.11.E-02
P38405-2	Isoform 2 of Guanine nucleotide-binding protein G(olf) subunit alpha	4.632	3.11.E-02
Q71U36-2	Isoform 2 of Tubulin alpha-1A chain	4.604	2.20.E-02
P35612-6	Isoform 6 of Beta-adducin	4.575	4.80.E-02
P35612-5	Isoform 5 of Beta-adducin	4.575	4.80.E-02
Q93050	V-type proton ATPase 116 kDa subunit a1	4.538	4.38.E-02
Q93050-1	Isoform 2 of V-type proton ATPase 116 kDa subunit a1	4.538	4.38.E-02
Q9NQX4	Unconventional myosin-Vc	4.529	3.04.E-02
Q5SW79	Centrosomal protein of 170 kDa	4.491	2.21.E-02
P28472-2	Isoform 2 of Gamma-aminobutyric acid receptor subunit beta-3	4.484	3.55.E-02
P28472-3	Isoform 3 of Gamma-aminobutyric acid receptor subunit beta-3	4.484	3.55.E-02
P28472-4	Isoform 4 of Gamma-aminobutyric acid receptor subunit beta-3	4.484	3.55.E-02
P28472	Gamma-aminobutyric acid receptor subunit beta-3	4.484	3.55.E-02
P35612	Beta-adducin	4.451	2.66.E-02
Q8IU85-2	Isoform 2 of Calcium/calmodulin-dependent protein kinase type 1D	4.443	3.23.E-02
Q8IU85	Calcium/calmodulin-dependent protein kinase type 1D	4.443	3.23.E-02
Q13491-3	Isoform 3 of Neuronal membrane glycoprotein M6-b	4.437	3.76.E-02
Q13491	Neuronal membrane glycoprotein M6-b	4.437	3.77.E-02
Q13491-2	Isoform 2 of Neuronal membrane glycoprotein M6-b	4.437	3.77.E-02
P35080-2	Isoform IIb of Profilin-2	4.435	4.09.E-02
Q93050-3	Isoform 3 of V-type proton ATPase 116 kDa subunit a1	4.434	4.64.E-02
P78310-5	Isoform 5 of Coxsackievirus and adenovirus receptor	4.398	2.50.E-02
P19022	Cadherin-2	4.395	2.43.E-02
P19022-2	Isoform 2 of Cadherin-2	4.395	2.43.E-02
Q92820	Gamma-glutamyl hydrolase	4.388	2.48.E-02
P78310-4	Isoform 4 of Coxsackievirus and adenovirus receptor	4.365	2.67.E-02

O00499-3	Isoform IIC1 of Myc box-dependent-interacting protein 1	4.281	3.58.E-02
O00499-5	Isoform IID of Myc box-dependent-interacting protein 1	4.281	3.58.E-02
O00499	Myc box-dependent-interacting protein 1	4.281	3.58.E-02
O00499-8	Isoform BIN1 of Myc box-dependent-interacting protein 1	4.281	4.09.E-02
O00499-7	Isoform II3 of Myc box-dependent-interacting protein 1	4.281	4.09.E-02
O00499-2	Isoform IIB of Myc box-dependent-interacting protein 1	4.281	4.09.E-02
O00499-10	Isoform BIN1-13 of Myc box-dependent-interacting protein 1	4.281	4.09.E-02
O00499-9	Isoform BIN1-10-13 of Myc box-dependent-interacting protein 1	4.281	4.09.E-02
O00499-4	Isoform IIC2 of Myc box-dependent-interacting protein 1	4.281	4.09.E-02
O00499-11	Isoform BIN1+12A of Myc box-dependent-interacting protein 1	4.281	4.09.E-02
O00499-6	Isoform II2 of Myc box-dependent-interacting protein 1	4.281	4.09.E-02
Q13509	Tubulin beta-3 chain	4.268	2.68.E-02
O43761	Synaptogyrin-3	4.229	3.75.E-02
Q8N111	Cell cycle exit and neuronal differentiation protein 1	4.215	3.69.E-02
Q8NBX0	Saccharopine dehydrogenase-like oxidoreductase	4.17	4.29.E-02
Q9H9B4	Sideroflexin-1	4.167	2.89.E-02
Q14011-2	Isoform 2 of Cold-inducible RNA-binding protein	4.152	3.19.E-02
P40424-2	Isoform PBX1b of Pre-B-cell leukemia transcription factor 1	4.124	2.93.E-02
P40424	Pre-B-cell leukemia transcription factor 1	4.124	2.93.E-02
P40424-3	Isoform 3 of Pre-B-cell leukemia transcription factor 1	4.124	2.93.E-02
P40425	Pre-B-cell leukemia transcription factor 2	4.124	2.93.E-02
Q6DN90-3	Isoform 3 of IQ motif and SEC7 domain-containing protein 1	4.115	4.33.E-02
Q6DN90-2	Isoform 2 of IQ motif and SEC7 domain-containing protein 1	4.115	4.33.E-02
Q6DN90	IQ motif and SEC7 domain-containing protein 1	4.115	4.33.E-02
Q13509-2	Isoform 2 of Tubulin beta-3 chain	4.11	2.95.E-02
P78310-6	Isoform 6 of Coxsackievirus and adenovirus receptor	4.043	3.12.E-02
P78310-2	Isoform 2 of Coxsackievirus and adenovirus receptor	4.043	3.12.E-02
P61019-2	Isoform 2 of Ras-related protein Rab-2A	3.964	3.49.E-02
Q16555-2	Isoform 2 of Dihydropyrimidinase-related protein 2	3.952	3.26.E-02
Q16555	Dihydropyrimidinase-related protein 2	3.952	3.26.E-02
Q14011	Cold-inducible RNA-binding protein	3.929	3.33.E-02
P78310	Coxsackievirus and adenovirus receptor	3.925	3.19.E-02
P78310-7	Isoform 7 of Coxsackievirus and adenovirus receptor	3.847	3.57.E-02
O75051	Plexin-A2	3.739	4.71.E-02
Q14204	Cytoplasmic dynein 1 heavy chain 1	3.72	3.80.E-02
Q9BVA1	Tubulin beta-2B chain	3.717	3.80.E-02
Q9NUQ9	CYFIP-related Rac1 interactor B	3.713	4.52.E-02
O95197-2	Isoform 2 of Reticulon-3	3.708	3.97.E-02
O95197-7	Isoform 7 of Reticulon-3	3.708	3.97.E-02
O95197	Reticulon-3	3.708	3.97.E-02
P19087	Guanine nucleotide-binding protein G(t) subunit alpha-2	3.645	4.75.E-02
A8MTJ3	Guanine nucleotide-binding protein G(t) subunit alpha-3	3.645	4.75.E-02
P11488	Guanine nucleotide-binding protein G(t) subunit alpha-1	3.645	4.75.E-02
Q14203-2	Isoform p135 of Dynactin subunit 1	3.581	4.16.E-02
Q14019	Coactosin-like protein	3.494	4.42.E-02
Q99798	Aconitate hydratase, mitochondrial	3.48	4.46.E-02
Q14203-6	Isoform 6 of Dynactin subunit 1	3.462	4.52.E-02
Q14203	Dynactin subunit 1	3.462	4.52.E-02
Q9BPW8	Protein NipSnap homolog 1	3.45	4.56.E-02
Q14117	Dihydropyrimidinase	3.328	4.32.E-02

Supplementary Table S1. (d) Proteins with decreased levels in the NSCs (<0.5 fold)

Accession	Protein Name	Abundance Ratio: (NSC) / (iPSC)	Abundance Ratio p-Value: (NSC) / (iPSC)
Q14244-3	Isoform 3 of Ensconsin	0.017	1.00.E-17
Q14244	Ensconsin	0.017	1.00.E-17
O75830	Serpin I2	0.016	1.00.E-17
P50453	Serpin B9	0.016	1.00.E-17
O00592	Podocalyxin	0.016	1.00.E-17
P30740	Leukocyte elastase inhibitor	0.016	1.00.E-17
O00592-2	Isoform 2 of Podocalyxin	0.016	1.00.E-17
P30740-2	Isoform 2 of Leukocyte elastase inhibitor	0.016	1.00.E-17
O75362	Zinc finger protein 217	0.01	1.00.E-17
Q96SZ4	Zinc finger and SCAN domain-containing protein 10	0.01	1.00.E-17
Q86UZ6	Zinc finger and BTB domain-containing protein 46	0.01	1.00.E-17
P30291	Wee1-like protein kinase	0.01	1.00.E-17
Q8IWA0	WD repeat-containing protein 75	0.01	1.00.E-17
Q96D96	Voltage-gated hydrogen channel 1	0.01	1.00.E-17
P62760	Visinin-like protein 1	0.01	1.00.E-17
P98155	Very low-density lipoprotein receptor	0.01	1.00.E-17
P13611	Versican core protein	0.01	1.00.E-17
O00160	Unconventional myosin-If	0.01	1.00.E-17
Q5T0Z8	Uncharacterized protein C6orf132	0.01	1.00.E-17
Q8N5I9	Uncharacterized protein C12orf45	0.01	1.00.E-17
Q9UHP3	Ubiquitin carboxyl-terminal hydrolase 25	0.01	1.00.E-17
Q9BVJ6	U3 small nucleolar RNA-associated protein 14 homolog A	0.01	1.00.E-17
P29350	Tyrosine-protein phosphatase non-receptor type 6	0.01	1.00.E-17
Q9UIG0	Tyrosine-protein kinase BAZ1B	0.01	1.00.E-17
Q86T03	Type 1 phosphatidylinositol 4,5-bisphosphate 4-phosphatase	0.01	1.00.E-17
Q9NZR1	Tropomodulin-2	0.01	1.00.E-17
Q6ZTA4	Tripartite motif-containing protein 67	0.01	1.00.E-17
Q9UN79	Transcription factor SOX-13	0.01	1.00.E-17
Q8NFU3	Thiosulfate:glutathione sulfurtransferase	0.01	1.00.E-17
Q86YL5	Testis development-related protein	0.01	1.00.E-17
O00445	Synaptotagmin-5	0.01	1.00.E-17
Q8N4V2	Synaptic vesicle 2-related protein	0.01	1.00.E-17
Q9Y5Y6	Suppressor of tumorigenicity 14 protein	0.01	1.00.E-17
Q9BX66	Sorbin and SH3 domain-containing protein 1	0.01	1.00.E-17
Q5K4L6	Solute carrier family 27 member 3	0.01	1.00.E-17
Q9BXS9	Solute carrier family 26 member 6	0.01	1.00.E-17
Q969I6	Sodium-coupled neutral amino acid transporter 4	0.01	1.00.E-17
Q9H2S1	Small conductance calcium-activated potassium channel protein 2	0.01	1.00.E-17
O75886	Signal transducing adapter molecule 2	0.01	1.00.E-17
Q9NUL5	Shiftless antiviral inhibitor of ribosomal frameshifting protein	0.01	1.00.E-17
Q9Y3L3	SH3 domain-binding protein 1	0.01	1.00.E-17
P48594	Serpin B4	0.01	1.00.E-17
P29508	Serpin B3	0.01	1.00.E-17
P48454	Serine/threonine-protein phosphatase 2B catalytic subunit gamma isoform	0.01	1.00.E-17
Q9Y388	RNA-binding motif protein, X-linked 2	0.01	1.00.E-17
Q8WVC0	RNA polymerase-associated protein LEO1	0.01	1.00.E-17
P62745	Rho-related GTP-binding protein RhoB	0.01	1.00.E-17
A5YM69	Rho guanine nucleotide exchange factor 35	0.01	1.00.E-17
Q9P227	Rho GTPase-activating protein 23	0.01	1.00.E-17
Q14CB8	Rho GTPase-activating protein 19	0.01	1.00.E-17
Q96D15	Reticulocalbin-3	0.01	1.00.E-17
Q6IQ49	Replication stress response regulator SDE2	0.01	1.00.E-17
Q9H426	Regulating synaptic membrane exocytosis protein 4	0.01	1.00.E-17
Q06416	Putative POU domain, class 5, transcription factor 1B	0.01	1.00.E-17
Q08174	Protocadherin-1	0.01	1.00.E-17
Q5TEJ8	Protein THEMIS2	0.01	1.00.E-17
Q6PI26	Protein SHQ1 homolog	0.01	1.00.E-17
Q9ULR3	Protein phosphatase 1H	0.01	1.00.E-17
Q9BZQ8	Protein Niban 1	0.01	1.00.E-17
Q9UKS6	Protein kinase C and casein kinase substrate in neurons protein 3	0.01	1.00.E-17
Q9BY11	Protein kinase C and casein kinase substrate in neurons protein 1	0.01	1.00.E-17
A6ND36	Protein FAM83G	0.01	1.00.E-17
P98173	Protein FAM3A	0.01	1.00.E-17
Q9NSI2	Protein FAM207A	0.01	1.00.E-17
Q9UHG2	ProSAAS	0.01	1.00.E-17
Q15652	Probable JmjC domain-containing histone demethylation protein 2C	0.01	1.00.E-17

Q8TBB6	Probable cationic amino acid transporter	0.01	1.00.E-17
Q01860	POU domain, class 5, transcription factor 1	0.01	1.00.E-17
Q8TD55	Pleckstrin homology domain-containing family O member 2	0.01	1.00.E-17
Q9NRG1	Phosphoribosyltransferase domain-containing protein 1	0.01	1.00.E-17
O14986	Phosphatidylinositol 4-phosphate 5-kinase type-1 beta	0.01	1.00.E-17
Q92626	Peroxidasin homolog	0.01	1.00.E-17
Q9H2H8	Peptidyl-prolyl cis-trans isomerase-like 3	0.01	1.00.E-17
Q53GG5	PDZ and LIM domain protein 3	0.01	1.00.E-17
Q96AD5	Patatin-like phospholipase domain-containing protein 2	0.01	1.00.E-17
Q9NWT1	p21-activated protein kinase-interacting protein 1	0.01	1.00.E-17
O43913	Origin recognition complex subunit 5	0.01	1.00.E-17
Q9BTX1	Nucleoporin NDC1	0.01	1.00.E-17
Q9NVX2	Notchless protein homolog 1	0.01	1.00.E-17
O95897	Noelin-2	0.01	1.00.E-17
P35228	Nitric oxide synthase, inducible	0.01	1.00.E-17
Q15773	Myeloid leukemia factor 2	0.01	1.00.E-17
Q9H7C9	Mth938 domain-containing protein	0.01	1.00.E-17
Q9NPA3	Mid1-interacting protein 1	0.01	1.00.E-17
P40967	Melanocyte protein PMEL	0.01	1.00.E-17
Q8NDA8	Maestro heat-like repeat-containing protein family member 1	0.01	1.00.E-17
Q9UN81	LINE-1 retrotransposable element ORF1 protein	0.01	1.00.E-17
Q9Y2S2	Lambda-crystallin homolog	0.01	1.00.E-17
O00515	Ladinin-1	0.01	1.00.E-17
O43291	Kunitz-type protease inhibitor 2	0.01	1.00.E-17
O43278	Kunitz-type protease inhibitor 1	0.01	1.00.E-17
P13611-5	Isoform Vint of Versican core protein	0.01	1.00.E-17
P13611-4	Isoform V3 of Versican core protein	0.01	1.00.E-17
P13611-3	Isoform V2 of Versican core protein	0.01	1.00.E-17
P13611-2	Isoform V1 of Versican core protein	0.01	1.00.E-17
Q9UHP3-3	Isoform USP25m of Ubiquitin carboxyl-terminal hydrolase 25	0.01	1.00.E-17
Q9UHP3-1	Isoform USP25b of Ubiquitin carboxyl-terminal hydrolase 25	0.01	1.00.E-17
P98155-2	Isoform Short of Very low-density lipoprotein receptor	0.01	1.00.E-17
Q9NZ52-2	Isoform Short of ADP-ribosylation factor-binding protein GGA3	0.01	1.00.E-17
Q6ZT62-2	Isoform Short BGIN of Bargin	0.01	1.00.E-17
Q9UJQ4-2	Isoform SALL4B of Sal-like protein 4	0.01	1.00.E-17
P41134-2	Isoform ID-B of DNA-binding protein inhibitor ID-1	0.01	1.00.E-17
P42263-2	Isoform Flip of Glutamate receptor 3	0.01	1.00.E-17
P42262-2	Isoform Flip of Glutamate receptor 2	0.01	1.00.E-17
Q9NSI2-2	Isoform B of Protein FAM207A	0.01	1.00.E-17
Q01860-2	Isoform B of POU domain, class 5, transcription factor 1	0.01	1.00.E-17
P21802-15	Isoform 9 of Fibroblast growth factor receptor 2	0.01	1.00.E-17
P55327-8	Isoform 8 of Tumor protein D52	0.01	1.00.E-17
Q9BX66-7	Isoform 7 of Sorbin and SH3 domain-containing protein 1	0.01	1.00.E-17
Q9BXS9-7	Isoform 7 of Solute carrier family 26 member 6	0.01	1.00.E-17
Q14CB8-7	Isoform 7 of Rho GTPase-activating protein 19	0.01	1.00.E-17
Q8NDA8-7	Isoform 7 of Maestro heat-like repeat-containing protein family member 1	0.01	1.00.E-17
P20839-7	Isoform 7 of Inosine-5'-monophosphate dehydrogenase 1	0.01	1.00.E-17
P21802-8	Isoform 7 of Fibroblast growth factor receptor 2	0.01	1.00.E-17
Q9BXS9-6	Isoform 6 of Solute carrier family 26 member 6	0.01	1.00.E-17
Q14CB8-6	Isoform 6 of Rho GTPase-activating protein 19	0.01	1.00.E-17
Q9BTX1-6	Isoform 6 of Nucleoporin NDC1	0.01	1.00.E-17
P20839-6	Isoform 6 of Inosine-5'-monophosphate dehydrogenase 1	0.01	1.00.E-17
P21802-6	Isoform 6 of Fibroblast growth factor receptor 2	0.01	1.00.E-17
Q7Z2W4-5	Isoform 5 of Zinc finger CCCH-type antiviral protein 1	0.01	1.00.E-17
O60343-5	Isoform 5 of TBC1 domain family member 4	0.01	1.00.E-17
Q9BXS9-5	Isoform 5 of Solute carrier family 26 member 6	0.01	1.00.E-17
Q14CB8-5	Isoform 5 of Rho GTPase-activating protein 19	0.01	1.00.E-17
Q9BTX1-5	Isoform 5 of Nucleoporin NDC1	0.01	1.00.E-17
P40967-5	Isoform 5 of Melanocyte protein PMEL	0.01	1.00.E-17
Q8NDA8-5	Isoform 5 of Maestro heat-like repeat-containing protein family member 1	0.01	1.00.E-17
P20839-5	Isoform 5 of Inosine-5'-monophosphate dehydrogenase 1	0.01	1.00.E-17
P21802-5	Isoform 5 of Fibroblast growth factor receptor 2	0.01	1.00.E-17
Q66PJ3-5	Isoform 5 of ADP-ribosylation factor-like protein 6-interacting protein 4	0.01	1.00.E-17
Q7Z2W4-4	Isoform 4 of Zinc finger CCCH-type antiviral protein 1	0.01	1.00.E-17
Q96D96-4	Isoform 4 of Voltage-gated hydrogen channel 1	0.01	1.00.E-17
P29350-4	Isoform 4 of Tyrosine-protein phosphatase non-receptor type 6	0.01	1.00.E-17
Q8NFU3-4	Isoform 4 of Thiosulfate:glutathione sulfurtransferase	0.01	1.00.E-17
O60343-4	Isoform 4 of TBC1 domain family member 4	0.01	1.00.E-17
Q9BXS9-4	Isoform 4 of Solute carrier family 26 member 6	0.01	1.00.E-17

Q5VT52-4	Isoform 4 of Regulation of nuclear pre-mRNA domain-containing protein 2	0.01	1.00.E-17
Q9BTX1-4	Isoform 4 of Nucleoporin NDC1	0.01	1.00.E-17
P40967-4	Isoform 4 of Melanocyte protein PMEL	0.01	1.00.E-17
Q8NDA8-4	Isoform 4 of Maestro heat-like repeat-containing protein family member 1	0.01	1.00.E-17
P20839-4	Isoform 4 of Inosine-5'-monophosphate dehydrogenase 1	0.01	1.00.E-17
Q14451-4	Isoform 4 of Growth factor receptor-bound protein 7	0.01	1.00.E-17
P42262-4	Isoform 4 of Glutamate receptor 2	0.01	1.00.E-17
P22607-4	Isoform 4 of Fibroblast growth factor receptor 3	0.01	1.00.E-17
P21802-4	Isoform 4 of Fibroblast growth factor receptor 2	0.01	1.00.E-17
Q9NZ52-4	Isoform 4 of ADP-ribosylation factor-binding protein GGA3	0.01	1.00.E-17
Q96SZ4-3	Isoform 3 of Zinc finger and SCAN domain-containing protein 10	0.01	1.00.E-17
Q96D96-3	Isoform 3 of Voltage-gated hydrogen channel 1	0.01	1.00.E-17
Q9BVJ6-3	Isoform 3 of U3 small nucleolar RNA-associated protein 14 homolog A	0.01	1.00.E-17
P29350-2	Isoform 3 of Tyrosine-protein phosphatase non-receptor type 6	0.01	1.00.E-17
Q12792-3	Isoform 3 of Twinfilin-1	0.01	1.00.E-17
P07951-3	Isoform 3 of Tropomyosin beta chain	0.01	1.00.E-17
Q8NFU3-3	Isoform 3 of Thiosulfate:glutathione sulfurtransferase	0.01	1.00.E-17
Q9BX66-3	Isoform 3 of Sorbin and SH3 domain-containing protein 1	0.01	1.00.E-17
Q5K4L6-3	Isoform 3 of Solute carrier family 27 member 3	0.01	1.00.E-17
Q9BXS9-3	Isoform 3 of Solute carrier family 26 member 6	0.01	1.00.E-17
P50452-3	Isoform 3 of Serpin B8	0.01	1.00.E-17
P48454-3	Isoform 3 of Serine/threonine-protein phosphatase 2B catalytic subunit gamma isoform	0.01	1.00.E-17
Q8TAC9-3	Isoform 3 of Secretory carrier-associated membrane protein 5	0.01	1.00.E-17
Q14CB8-3	Isoform 3 of Rho GTPase-activating protein 19	0.01	1.00.E-17
Q6IQ49-3	Isoform 3 of Replication stress response regulator SDE2	0.01	1.00.E-17
O75061-3	Isoform 3 of Putative tyrosine-protein phosphatase auxilin	0.01	1.00.E-17
P98173-3	Isoform 3 of Protein FAM3A	0.01	1.00.E-17
O00469-3	Isoform 3 of Procollagen-lysine,2-oxoglutarate 5-dioxygenase 2	0.01	1.00.E-17
Q15652-3	Isoform 3 of Probable JmjC domain-containing histone demethylation protein 2C	0.01	1.00.E-17
O14986-3	Isoform 3 of Phosphatidylinositol 4-phosphate 5-kinase type-1 beta	0.01	1.00.E-17
Q53GG5-3	Isoform 3 of PDZ and LIM domain protein 3	0.01	1.00.E-17
Q9H7C9-3	Isoform 3 of Mth938 domain-containing protein	0.01	1.00.E-17
P40967-3	Isoform 3 of Melanocyte protein PMEL	0.01	1.00.E-17
P20839-3	Isoform 3 of Inosine-5'-monophosphate dehydrogenase 1	0.01	1.00.E-17
Q01973-3	Isoform 3 of Inactive tyrosine-protein kinase transmembrane receptor ROR1	0.01	1.00.E-17
P04233-3	Isoform 3 of HLA class II histocompatibility antigen gamma chain	0.01	1.00.E-17
Q14451-3	Isoform 3 of Growth factor receptor-bound protein 7	0.01	1.00.E-17
P42262-3	Isoform 3 of Glutamate receptor 2	0.01	1.00.E-17
P22607-3	Isoform 3 of Fibroblast growth factor receptor 3	0.01	1.00.E-17
P21802-3	Isoform 3 of Fibroblast growth factor receptor 2	0.01	1.00.E-17
P21709-3	Isoform 3 of Ephrin type-A receptor 1	0.01	1.00.E-17
Q8NI60-3	Isoform 3 of Atypical kinase COQ8A, mitochondrial	0.01	1.00.E-17
Q9ULZ3-3	Isoform 3 of Apoptosis-associated speck-like protein containing a CARD	0.01	1.00.E-17
Q9C0B1-3	Isoform 3 of Alpha-ketoglutarate-dependent dioxygenase FTO	0.01	1.00.E-17
Q9NZ52-3	Isoform 3 of ADP-ribosylation factor-binding protein GGA3	0.01	1.00.E-17
P12110-3	Isoform 2C2A' of Collagen alpha-2(VI) chain	0.01	1.00.E-17
P12110-2	Isoform 2C2A of Collagen alpha-2(VI) chain	0.01	1.00.E-17
Q13433-2	Isoform 2 of Zinc transporter ZIP6	0.01	1.00.E-17
Q96IQ9-2	Isoform 2 of Zinc finger protein 414	0.01	1.00.E-17
P30291-2	Isoform 2 of Wee1-like protein kinase	0.01	1.00.E-17
P09327-2	Isoform 2 of Villin-1	0.01	1.00.E-17
Q9BVJ6-2	Isoform 2 of U3 small nucleolar RNA-associated protein 14 homolog A	0.01	1.00.E-17
P29350-3	Isoform 2 of Tyrosine-protein phosphatase non-receptor type 6	0.01	1.00.E-17
Q9UIG0-2	Isoform 2 of Tyrosine-protein kinase BAZ1B	0.01	1.00.E-17
Q86T03-2	Isoform 2 of Type 1 phosphatidylinositol 4,5-bisphosphate 4-phosphatase	0.01	1.00.E-17
P07951-2	Isoform 2 of Tropomyosin beta chain	0.01	1.00.E-17
Q9NZR1-2	Isoform 2 of Tropomodulin-2	0.01	1.00.E-17
Q6ZTA4-2	Isoform 2 of Tripartite motif-containing protein 67	0.01	1.00.E-17
Q8NFU3-2	Isoform 2 of Thiosulfate:glutathione sulfurtransferase	0.01	1.00.E-17
Q86YL5-2	Isoform 2 of Testis development-related protein	0.01	1.00.E-17
O00445-2	Isoform 2 of Synaptotagmin-5	0.01	1.00.E-17
P08247-2	Isoform 2 of Synaptophysin	0.01	1.00.E-17
Q9BX66-2	Isoform 2 of Sorbin and SH3 domain-containing protein 1	0.01	1.00.E-17
Q5K4L6-2	Isoform 2 of Solute carrier family 27 member 3	0.01	1.00.E-17
Q9BXS9-2	Isoform 2 of Solute carrier family 26 member 6	0.01	1.00.E-17
Q9H2S1-2	Isoform 2 of Small conductance calcium-activated potassium channel protein 2	0.01	1.00.E-17
Q9NUL5-2	Isoform 2 of Shiftless antiviral inhibitor of ribosomal frameshifting protein	0.01	1.00.E-17
Q9Y3L3-2	Isoform 2 of SH3 domain-binding protein 1	0.01	1.00.E-17
P29508-2	Isoform 2 of Serpin B3	0.01	1.00.E-17

P48454-2	Isoform 2 of Serine/threonine-protein phosphatase 2B catalytic subunit gamma isoform	0.01	1.00.E-17
Q8WVC0-2	Isoform 2 of RNA polymerase-associated protein LEO1	0.01	1.00.E-17
Q14137-2	Isoform 2 of Ribosome biogenesis protein BOP1	0.01	1.00.E-17
Q9P227-2	Isoform 2 of Rho GTPase-activating protein 23	0.01	1.00.E-17
Q14CB8-2	Isoform 2 of Rho GTPase-activating protein 19	0.01	1.00.E-17
Q6IQ49-2	Isoform 2 of Replication stress response regulator SDE2	0.01	1.00.E-17
Q9H426-2	Isoform 2 of Regulating synaptic membrane exocytosis protein 4	0.01	1.00.E-17
Q08174-2	Isoform 2 of Protocadherin-1	0.01	1.00.E-17
Q6PI26-2	Isoform 2 of Protein SHQ1 homolog	0.01	1.00.E-17
Q96G01-2	Isoform 2 of Protein bicaudal D homolog 1	0.01	1.00.E-17
Q15652-2	Isoform 2 of Probable JmjC domain-containing histone demethylation protein 2C	0.01	1.00.E-17
Q8TD55-2	Isoform 2 of Pleckstrin homology domain-containing family O member 2	0.01	1.00.E-17
Q9NRG1-2	Isoform 2 of Phosphoribosyltransferase domain-containing protein 1	0.01	1.00.E-17
O14986-2	Isoform 2 of Phosphatidylinositol 4-phosphate 5-kinase type-1 beta	0.01	1.00.E-17
Q53GG5-2	Isoform 2 of PDZ and LIM domain protein 3	0.01	1.00.E-17
Q96AD5-2	Isoform 2 of Patatin-like phospholipase domain-containing protein 2	0.01	1.00.E-17
O43913-2	Isoform 2 of Origin recognition complex subunit 5	0.01	1.00.E-17
Q9BTX1-2	Isoform 2 of Nucleoporin NDC1	0.01	1.00.E-17
P35228-2	Isoform 2 of Nitric oxide synthase, inducible	0.01	1.00.E-17
O14745-2	Isoform 2 of Na(+)/H(+) exchange regulatory cofactor NHE-RF1	0.01	1.00.E-17
Q9H7C9-2	Isoform 2 of Mth938 domain-containing protein	0.01	1.00.E-17
P40967-2	Isoform 2 of Melanocyte protein PMEL	0.01	1.00.E-17
Q8NDA8-2	Isoform 2 of Maestro heat-like repeat-containing protein family member 1	0.01	1.00.E-17
Q9Y2S2-2	Isoform 2 of Lambda-crystallin homolog	0.01	1.00.E-17
O43291-2	Isoform 2 of Kunitz-type protease inhibitor 2	0.01	1.00.E-17
O43278-2	Isoform 2 of Kunitz-type protease inhibitor 1	0.01	1.00.E-17
P29218-2	Isoform 2 of Inositol monophosphatase 1	0.01	1.00.E-17
P20839-2	Isoform 2 of Inosine-5'-monophosphate dehydrogenase 1	0.01	1.00.E-17
Q9NQS7-2	Isoform 2 of Inner centromere protein	0.01	1.00.E-17
P04233-2	Isoform 2 of HLA class II histocompatibility antigen gamma chain	0.01	1.00.E-17
Q9Y5Z4-2	Isoform 2 of Heme-binding protein 2	0.01	1.00.E-17
Q14451-2	Isoform 2 of Growth factor receptor-bound protein 7	0.01	1.00.E-17
Q9NZH0-2	Isoform 2 of G-protein coupled receptor family C group 5 member B	0.01	1.00.E-17
P48507-2	Isoform 2 of Glutamate--cysteine ligase regulatory subunit	0.01	1.00.E-17
Q14192-2	Isoform 2 of Four and a half LIM domains protein 2	0.01	1.00.E-17
P22607-2	Isoform 2 of Fibroblast growth factor receptor 3	0.01	1.00.E-17
P21802-2	Isoform 2 of Fibroblast growth factor receptor 2	0.01	1.00.E-17
Q9H6T0-2	Isoform 2 of Epithelial splicing regulatory protein 2	0.01	1.00.E-17
P21709-2	Isoform 2 of Ephrin type-A receptor 1	0.01	1.00.E-17
Q96JJ3-3	Isoform 2 of Engulfment and cell motility protein 2	0.01	1.00.E-17
Q9NZN3-2	Isoform 2 of EH domain-containing protein 3	0.01	1.00.E-17
Q8IYM9-2	Isoform 2 of E3 ubiquitin-protein ligase TRIM22	0.01	1.00.E-17
Q86Y13-2	Isoform 2 of E3 ubiquitin-protein ligase DZIP3	0.01	1.00.E-17
O43174-2	Isoform 2 of Cytochrome P450 26A1	0.01	1.00.E-17
Q7Z5L2-2	Isoform 2 of Coiled-coil domain-containing protein R3HCC1L	0.01	1.00.E-17
P22223-2	Isoform 2 of Cadherin-3	0.01	1.00.E-17
Q10589-2	Isoform 2 of Bone marrow stromal antigen 2	0.01	1.00.E-17
Q9Y6X9-2	Isoform 2 of ATPase MORC2	0.01	1.00.E-17
Q9ULZ3-2	Isoform 2 of Apoptosis-associated speck-like protein containing a CARD	0.01	1.00.E-17
Q9NQW6-2	Isoform 2 of Anillin	0.01	1.00.E-17
Q9Y3D8-2	Isoform 2 of Adenylate kinase isoenzyme 6	0.01	1.00.E-17
O43741-2	Isoform 2 of 5'-AMP-activated protein kinase subunit beta-2	0.01	1.00.E-17
P21802-23	Isoform 17 of Fibroblast growth factor receptor 2	0.01	1.00.E-17
P21802-22	Isoform 16 of Fibroblast growth factor receptor 2	0.01	1.00.E-17
P21802-21	Isoform 15 of Fibroblast growth factor receptor 2	0.01	1.00.E-17
P21802-20	Isoform 14 of Fibroblast growth factor receptor 2	0.01	1.00.E-17
P21802-18	Isoform 12 of Fibroblast growth factor receptor 2	0.01	1.00.E-17
Q9BX66-11	Isoform 11 of Sorbin and SH3 domain-containing protein 1	0.01	1.00.E-17
P21802-17	Isoform 11 of Fibroblast growth factor receptor 2	0.01	1.00.E-17
P21802-16	Isoform 10 of Fibroblast growth factor receptor 2	0.01	1.00.E-17
P20839	Inosine-5'-monophosphate dehydrogenase 1	0.01	1.00.E-17
Q9NQS7	Inner centromere protein	0.01	1.00.E-17
P14902	Indoleamine 2,3-dioxygenase 1	0.01	1.00.E-17
Q9UIU6	Homeobox protein SIX4	0.01	1.00.E-17
P04233	HLA class II histocompatibility antigen gamma chain	0.01	1.00.E-17
Q9Y241	HIG1 domain family member 1A, mitochondrial	0.01	1.00.E-17
Q14451	Growth factor receptor-bound protein 7	0.01	1.00.E-17
Q9NZH0	G-protein coupled receptor family C group 5 member B	0.01	1.00.E-17
P51810	G-protein coupled receptor 143	0.01	1.00.E-17

P0CG30	Glutathione S-transferase theta-2B	0.01	1.00.E-17
P0CG29	Glutathione S-transferase theta-2	0.01	1.00.E-17
P48507	Glutamate--cysteine ligase regulatory subunit	0.01	1.00.E-17
P48058	Glutamate receptor 4	0.01	1.00.E-17
P42263	Glutamate receptor 3	0.01	1.00.E-17
P42262	Glutamate receptor 2	0.01	1.00.E-17
O76070	Gamma-synuclein	0.01	1.00.E-17
P58549	FXYD domain-containing ion transport regulator 7	0.01	1.00.E-17
Q13643	Four and a half LIM domains protein 3	0.01	1.00.E-17
P22607	Fibroblast growth factor receptor 3	0.01	1.00.E-17
P21802	Fibroblast growth factor receptor 2	0.01	1.00.E-17
Q9NVQ4	Fas apoptotic inhibitory molecule 1	0.01	1.00.E-17
Q9H6T0	Epithelial splicing regulatory protein 2	0.01	1.00.E-17
Q15768	Ephrin-B3	0.01	1.00.E-17
P21709	Ephrin type-A receptor 1	0.01	1.00.E-17
Q96JJ3	Engulfment and cell motility protein 2	0.01	1.00.E-17
Q8IYM9	E3 ubiquitin-protein ligase TRIM22	0.01	1.00.E-17
Q9P0K9	DOMON domain-containing protein FRRS1L	0.01	1.00.E-17
P41134	DNA-binding protein inhibitor ID-1	0.01	1.00.E-17
Q7L190	Developmental pluripotency-associated protein 4	0.01	1.00.E-17
Q6UX07	Dehydrogenase/reductase SDR family member 13	0.01	1.00.E-17
O43174	Cytochrome P450 26A1	0.01	1.00.E-17
P12110	Collagen alpha-2(VI) chain	0.01	1.00.E-17
Q7Z5L2	Coiled-coil domain-containing protein R3HCC1L	0.01	1.00.E-17
Q9UBR2	Cathepsin Z	0.01	1.00.E-17
P26232	Catenin alpha-2	0.01	1.00.E-17
P22223	Cadherin-3	0.01	1.00.E-17
Q96CX2	BTB/POZ domain-containing protein KCTD12	0.01	1.00.E-17
Q10589	Bone marrow stromal antigen 2	0.01	1.00.E-17
Q5T5X7	BEN domain-containing protein 3	0.01	1.00.E-17
O95999	B-cell lymphoma/leukemia 10	0.01	1.00.E-17
Q6ZT62	Bargin	0.01	1.00.E-17
O14965	Aurora kinase A	0.01	1.00.E-17
Q8NI60	Atypical kinase COQ8A, mitochondrial	0.01	1.00.E-17
Q9Y6X9	ATPase MORC2	0.01	1.00.E-17
Q9ULZ3	Apoptosis-associated speck-like protein containing a CARD	0.01	1.00.E-17
P02656	Apolipoprotein C-III	0.01	1.00.E-17
P59780	AP-3 complex subunit sigma-2	0.01	1.00.E-17
O75843	AP-1 complex subunit gamma-like 2	0.01	1.00.E-17
Q9NQW6	Anillin	0.01	1.00.E-17
Q9NZ52	ADP-ribosylation factor-binding protein GGA3	0.01	1.00.E-17
Q9Y3D8	Adenylate kinase isoenzyme 6	0.01	1.00.E-17
P14621	Acylphosphatase-2	0.01	1.00.E-17
O43741	5'-AMP-activated protein kinase subunit beta-2	0.01	1.00.E-17
P31947	14-3-3 protein sigma	0.01	1.00.E-17
Q14244-2	Isoform 2 of Ensconsin	0.017	3.11.E-15
P12830-2	Isoform 2 of Cadherin-1	0.027	5.77.E-15
Q14244-7	Isoform 7 of Ensconsin	0.017	9.39.E-14
Q14244-6	Isoform 6 of Ensconsin	0.017	9.39.E-14
Q14244-5	Isoform 5 of Ensconsin	0.017	9.39.E-14
Q14244-4	Isoform 4 of Ensconsin	0.017	6.17.E-13
Q8N1A0	Keratin-like protein KRT222	0.035	1.64.E-12
P35900	Keratin, type I cytoskeletal 20	0.035	1.64.E-12
Q8N1A0-2	Isoform 2 of Keratin-like protein KRT222	0.035	1.64.E-12
P16422	Epithelial cell adhesion molecule	0.044	3.24.E-11
Q5T7N2	LINE-1 type transposase domain-containing protein 1	0.046	6.22.E-11
Q96SQ9-2	Isoform 2 of Cytochrome P450 2S1	0.048	1.05.E-10
Q96SQ9	Cytochrome P450 2S1	0.048	1.05.E-10
Q9Y6Q5	AP-1 complex subunit mu-2	0.053	1.55.E-10
P55327	Tumor protein D52	0.027	5.92.E-10
P55327-7	Isoform 7 of Tumor protein D52	0.027	5.92.E-10
P55327-6	Isoform 6 of Tumor protein D52	0.027	5.92.E-10
P55327-5	Isoform 5 of Tumor protein D52	0.027	5.92.E-10
P55327-4	Isoform 4 of Tumor protein D52	0.027	5.92.E-10
P55327-3	Isoform 3 of Tumor protein D52	0.027	5.92.E-10
P55327-2	Isoform 2 of Tumor protein D52	0.027	5.92.E-10
P19012-2	Isoform 2 of Keratin, type I cytoskeletal 15	0.082	8.45.E-09
Q96QD8	Sodium-coupled neutral amino acid transporter 2	0.069	1.31.E-08
P12830	Cadherin-1	0.087	1.44.E-08

Q8TDB8-3	Isoform 3 of Solute carrier family 2, facilitated glucose transporter member 14	0.082	1.58.E-08
Q8WY07	Cationic amino acid transporter 3	0.068	3.74.E-08
P02649	Apolipoprotein E	0.078	4.09.E-08
Q9Y6Q5-2	Isoform 2 of AP-1 complex subunit mu-2	0.075	5.69.E-08
O43272	Proline dehydrogenase 1, mitochondrial	0.047	6.58.E-08
Q16851	UTP--glucose-1-phosphate uridylyltransferase	0.081	1.13.E-07
Q08431	Lactadherin	0.103	1.43.E-07
O43272-1	Isoform 3 of Proline dehydrogenase 1, mitochondrial	0.047	1.50.E-07
O43272-2	Isoform 2 of Proline dehydrogenase 1, mitochondrial	0.047	1.50.E-07
Q9UHI5	Large neutral amino acids transporter small subunit 2	0.041	3.40.E-07
Q9H9Z2	Protein lin-28 homolog A	0.091	4.12.E-07
Q08431-3	Isoform 3 of Lactadherin	0.103	4.15.E-07
Q86YL7	Podoplanin	0.063	4.98.E-07
Q86YL7-6	Isoform 6 of Podoplanin	0.063	4.98.E-07
Q86YL7-5	Isoform 5 of Podoplanin	0.063	4.98.E-07
Q86YL7-4	Isoform 4 of Podoplanin	0.063	4.98.E-07
Q86YL7-3	Isoform 3 of Podoplanin	0.063	4.98.E-07
Q08431-4	Isoform 4 of Lactadherin	0.107	6.09.E-07
Q9UBC3-6	Isoform 6 of DNA (cytosine-5)-methyltransferase 3B	0.084	1.72.E-06
Q9UBC3-5	Isoform 5 of DNA (cytosine-5)-methyltransferase 3B	0.084	1.72.E-06
Q9UBC3-2	Isoform 2 of DNA (cytosine-5)-methyltransferase 3B	0.084	1.72.E-06
Q9UBC3	DNA (cytosine-5)-methyltransferase 3B	0.084	1.72.E-06
P00338	L-lactate dehydrogenase A chain	0.103	1.72.E-06
P00338-3	Isoform 3 of L-lactate dehydrogenase A chain	0.103	1.72.E-06
P00338-4	Isoform 4 of L-lactate dehydrogenase A chain	0.104	1.83.E-06
Q16401-2	Isoform 2 of 26S proteasome non-ATPase regulatory subunit 5	0.117	2.50.E-06
Q16401	26S proteasome non-ATPase regulatory subunit 5	0.117	2.50.E-06
O14745	Na(+)/H(+) exchange regulatory cofactor NHE-RF1	0.104	2.51.E-06
Q08431-2	Isoform 2 of Lactadherin	0.118	2.72.E-06
P13646	Keratin, type I cytoskeletal 13	0.114	3.30.E-06
P13646-3	Isoform 3 of Keratin, type I cytoskeletal 13	0.114	3.30.E-06
P00338-5	Isoform 5 of L-lactate dehydrogenase A chain	0.111	3.61.E-06
P00338-2	Isoform 2 of L-lactate dehydrogenase A chain	0.111	3.61.E-06
Q93062-3	Isoform C of RNA-binding protein with multiple splicing	0.129	5.66.E-06
Q99456	Keratin, type I cytoskeletal 12	0.124	6.55.E-06
Q04695	Keratin, type I cytoskeletal 17	0.122	6.65.E-06
P13646-2	Isoform 2 of Keratin, type I cytoskeletal 13	0.122	6.69.E-06
Q93062	RNA-binding protein with multiple splicing	0.129	6.86.E-06
Q93062-5	Isoform E of RNA-binding protein with multiple splicing	0.129	6.86.E-06
Q93062-4	Isoform D of RNA-binding protein with multiple splicing	0.129	6.86.E-06
Q93062-2	Isoform B of RNA-binding protein with multiple splicing	0.129	6.86.E-06
Q9UBC3-4	Isoform 4 of DNA (cytosine-5)-methyltransferase 3B	0.084	7.07.E-06
Q9UBC3-3	Isoform 3 of DNA (cytosine-5)-methyltransferase 3B	0.084	7.07.E-06
Q96QD8-2	Isoform 2 of Sodium-coupled neutral amino acid transporter 2	0.082	7.43.E-06
Q9Y624	Junctional adhesion molecule A	0.135	8.21.E-06
Q9UBC3-7	Isoform 7 of DNA (cytosine-5)-methyltransferase 3B	0.077	8.91.E-06
P08727	Keratin, type I cytoskeletal 19	0.122	1.02.E-05
Q99541	Perilipin-2	0.095	1.08.E-05
Q9UBC3-8	Isoform 8 of DNA (cytosine-5)-methyltransferase 3B	0.077	1.09.E-05
P19012	Keratin, type I cytoskeletal 15	0.125	1.12.E-05
Q9UHI5-4	Isoform 4 of Large neutral amino acids transporter small subunit 2	0.041	1.16.E-05
Q9UHI5-3	Isoform 3 of Large neutral amino acids transporter small subunit 2	0.041	1.16.E-05
Q9UHI5-2	Isoform 2 of Large neutral amino acids transporter small subunit 2	0.041	1.16.E-05
P10620	Microsomal glutathione S-transferase 1	0.143	1.36.E-05
Q6ZRY4	RNA-binding protein with multiple splicing 2	0.142	1.55.E-05
P09038-3	Isoform 4 of Fibroblast growth factor 2	0.085	1.95.E-05
P09038-2	Isoform 3 of Fibroblast growth factor 2	0.085	1.95.E-05
P09038-1	Isoform 2 of Fibroblast growth factor 2	0.085	1.95.E-05
P09038	Fibroblast growth factor 2	0.085	1.95.E-05
P10620-2	Isoform 2 of Microsomal glutathione S-transferase 1	0.143	1.98.E-05
Q01973-2	Isoform Short of Inactive tyrosine-protein kinase transmembrane receptor ROR1	0.087	1.99.E-05
Q8WUP2-3	Isoform 3 of Filamin-binding LIM protein 1	0.143	2.10.E-05
Q8WUP2-2	Isoform 2 of Filamin-binding LIM protein 1	0.143	2.10.E-05
Q8WUP2	Filamin-binding LIM protein 1	0.143	2.10.E-05
P80723	Brain acid soluble protein 1	0.101	2.37.E-05
Q13277	Syntaxin-3	0.087	2.72.E-05
Q13277-2	Isoform B of Syntaxin-3	0.087	2.72.E-05
Q13277-3	Isoform 3 of Syntaxin-3	0.087	2.72.E-05
P50579	Methionine aminopeptidase 2	0.099	3.07.E-05

Q01973	Inactive tyrosine-protein kinase transmembrane receptor ROR1	0.09	3.27.E-05
Q9UDR5	Alpha-aminoadipic semialdehyde synthase, mitochondrial	0.138	3.34.E-05
Q6BCY4	NADH-cytochrome b5 reductase 2	0.086	3.41.E-05
Q6BCY4-2	Isoform 2 of NADH-cytochrome b5 reductase 2	0.086	3.41.E-05
P46013-2	Isoform Short of Proliferation marker protein Ki-67	0.102	3.75.E-05
Q2M2I5	Keratin, type I cytoskeletal 24	0.156	4.60.E-05
O76015	Keratin, type I cuticular Ha8	0.156	4.60.E-05
O76014	Keratin, type I cuticular Ha7	0.156	4.60.E-05
O76013	Keratin, type I cuticular Ha6	0.156	4.60.E-05
Q14525	Keratin, type I cuticular Ha3-II	0.156	4.60.E-05
Q14532	Keratin, type I cuticular Ha2	0.156	4.60.E-05
Q15323	Keratin, type I cuticular Ha1	0.156	4.60.E-05
O76013-2	Isoform 2 of Keratin, type I cuticular Ha6	0.156	4.60.E-05
Q9Y624-2	Isoform 2 of Junctional adhesion molecule A	0.156	5.41.E-05
Q9C0H6	Kelch-like protein 4	0.093	5.45.E-05
Q9C0H6-2	Isoform 2 of Kelch-like protein 4	0.093	5.45.E-05
Q6P5R6	60S ribosomal protein L22-like 1	0.115	5.50.E-05
P80723-2	Isoform 2 of Brain acid soluble protein 1	0.101	5.77.E-05
P17302	Gap junction alpha-1 protein	0.183	5.88.E-05
Q9UJQ4	Sal-like protein 4	0.105	5.94.E-05
O00151	PDZ and LIM domain protein 1	0.15	7.30.E-05
P50579-3	Isoform 3 of Methionine aminopeptidase 2	0.099	7.58.E-05
Q92673	Sortilin-related receptor	0.163	7.73.E-05
P27105-2	Isoform 2 of Erythrocyte band 7 integral membrane protein	0.131	7.76.E-05
Q9UK22	F-box only protein 2	0.168	9.32.E-05
P49848	Transcription initiation factor TFIID subunit 6	0.088	9.72.E-05
P49848-4	Isoform 4 of Transcription initiation factor TFIID subunit 6	0.088	9.72.E-05
P49848-3	Isoform 3 of Transcription initiation factor TFIID subunit 6	0.088	9.72.E-05
P54652	Heat shock-related 70 kDa protein 2	0.092	9.94.E-05
O94808	Glutamine--fructose-6-phosphate aminotransferase [isomerizing] 2	0.152	1.00.E-04
Q14126	Desmoglein-2	0.194	1.03.E-04
Q16706	Alpha-mannosidase 2	0.173	1.13.E-04
P12532-2	Isoform 2 of Creatine kinase U-type, mitochondrial	0.175	1.24.E-04
P12532	Creatine kinase U-type, mitochondrial	0.175	1.24.E-04
P40121	Macrophage-capping protein	0.178	1.54.E-04
P40121-2	Isoform 2 of Macrophage-capping protein	0.178	1.54.E-04
P02452	Collagen alpha-1(I) chain	0.13	1.61.E-04
Q9UNN8	Endothelial protein C receptor	0.118	1.80.E-04
P22413	Ectonucleotide pyrophosphatase/phosphodiesterase family member 1	0.089	1.83.E-04
P09493-9	Isoform 9 of Tropomyosin alpha-1 chain	0.117	1.92.E-04
P09493-8	Isoform 8 of Tropomyosin alpha-1 chain	0.117	1.92.E-04
P09493-7	Isoform 7 of Tropomyosin alpha-1 chain	0.117	1.92.E-04
P09493-3	Isoform 3 of Tropomyosin alpha-1 chain	0.117	1.92.E-04
P09493-2	Isoform 2 of Tropomyosin alpha-1 chain	0.117	1.92.E-04
Q0VF96-2	Isoform 2 of Cingulin-like protein 1	0.134	1.94.E-04
Q9Y3Z3	Deoxynucleoside triphosphate triphosphohydrolase SAMHD1	0.204	2.12.E-04
Q9Y3Z3-4	Isoform 4 of Deoxynucleoside triphosphate triphosphohydrolase SAMHD1	0.205	2.12.E-04
Q2Q1W2	E3 ubiquitin-protein ligase TRIM71	0.17	2.20.E-04
Q92925	SWI/SNF-related matrix-associated actin-dependent regulator of chromatin subfamily D member 2	0.135	2.23.E-04
Q92925-3	Isoform 3 of SWI/SNF-related matrix-associated actin-dependent regulator of chromatin subfamily D member 2	0.135	2.23.E-04
Q92925-2	Isoform 2 of SWI/SNF-related matrix-associated actin-dependent regulator of chromatin subfamily D member 2	0.135	2.23.E-04
Q5UIP0	Telomere-associated protein RIF1	0.191	2.34.E-04
Q5UIP0-2	Isoform 2 of Telomere-associated protein RIF1	0.191	2.34.E-04
Q5T6F2	Ubiquitin-associated protein 2	0.159	2.36.E-04
P09525-2	Isoform 2 of Annexin A4	0.122	2.39.E-04
Q9Y3Z3-3	Isoform 3 of Deoxynucleoside triphosphate triphosphohydrolase SAMHD1	0.196	2.59.E-04
P58107	Epiplakin	0.179	2.77.E-04
Q9BYZ2	L-lactate dehydrogenase A-like 6B	0.16	2.82.E-04
Q8TDB8	Solute carrier family 2, facilitated glucose transporter member 14	0.188	2.99.E-04
Q8TDB8-5	Isoform 5 of Solute carrier family 2, facilitated glucose transporter member 14	0.188	2.99.E-04
Q8TDB8-4	Isoform 4 of Solute carrier family 2, facilitated glucose transporter member 14	0.188	2.99.E-04
Q8TDB8-2	Isoform 2 of Solute carrier family 2, facilitated glucose transporter member 14	0.188	2.99.E-04
Q96IZ0	PRKC apoptosis WT1 regulator protein	0.185	3.18.E-04
Q0VF96	Cingulin-like protein 1	0.134	3.51.E-04
Q92485	Acid sphingomyelinase-like phosphodiesterase 3b	0.131	4.22.E-04
P60903	Protein S100-A10	0.207	4.46.E-04
P35558	Phosphoenolpyruvate carboxykinase, cytosolic [GTP]	0.112	4.47.E-04
P35558-2	Isoform 2 of Phosphoenolpyruvate carboxykinase, cytosolic [GTP]	0.112	4.47.E-04

Q03135-2	Isoform 2 of Caveolin-1	0.152	4.50.E-04
Q03135	Caveolin-1	0.152	4.50.E-04
Q13547	Histone deacetylase 1	0.206	4.57.E-04
P50579-2	Isoform 2 of Methionine aminopeptidase 2	0.069	4.60.E-04
Q9BV40	Vesicle-associated membrane protein 8	0.133	4.65.E-04
P29762	Cellular retinoic acid-binding protein 1	0.188	5.09.E-04
O15446-2	Isoform 2 of DNA-directed RNA polymerase I subunit RPA34	0.159	5.10.E-04
O15446	DNA-directed RNA polymerase I subunit RPA34	0.159	5.10.E-04
Q9P2M7	Cingulin	0.202	6.04.E-04
Q15833	Syntaxin-binding protein 2	0.189	6.22.E-04
Q15833-3	Isoform 3 of Syntaxin-binding protein 2	0.189	6.22.E-04
Q15833-2	Isoform 2 of Syntaxin-binding protein 2	0.189	6.22.E-04
Q9NRW3	DNA dC->dU-editing enzyme APOBEC-3C	0.096	6.34.E-04
Q01970-2	Isoform 2 of 1-phosphatidylinositol 4,5-bisphosphate phosphodiesterase beta-3	0.22	6.61.E-04
Q5VZK9-2	Isoform 2 of F-actin-uncapping protein LRRC16A	0.076	6.72.E-04
Q5VZK9	F-actin-uncapping protein LRRC16A	0.076	6.72.E-04
P10321-2	Isoform 2 of HLA class I histocompatibility antigen, C alpha chain	0.211	6.77.E-04
O14494	Phospholipid phosphatase 1	0.125	6.90.E-04
O14494-2	Isoform 2 of Phospholipid phosphatase 1	0.125	6.90.E-04
Q01628	Interferon-induced transmembrane protein 3	0.235	7.02.E-04
Q8IVW6-4	Isoform 4 of AT-rich interactive domain-containing protein 3B	0.133	7.23.E-04
Q8IVW6-3	Isoform 3 of AT-rich interactive domain-containing protein 3B	0.133	7.23.E-04
Q8IVW6	AT-rich interactive domain-containing protein 3B	0.133	7.23.E-04
P13797-3	Isoform 3 of Plastin-3	0.196	7.26.E-04
Q05682-6	Isoform 6 of Caldesmon	0.209	7.60.E-04
Q05682-5	Isoform 5 of Caldesmon	0.209	7.60.E-04
Q05682-4	Isoform 4 of Caldesmon	0.209	7.60.E-04
Q05682-3	Isoform 3 of Caldesmon	0.209	7.60.E-04
Q05682-2	Isoform 2 of Caldesmon	0.209	7.60.E-04
Q05682	Caldesmon	0.209	7.60.E-04
Q9Y5K6	CD2-associated protein	0.15	7.65.E-04
O75475	PC4 and SFRS1-interacting protein	0.208	7.79.E-04
Q9P2M7-2	Isoform 2 of Cingulin	0.158	7.99.E-04
P10321	HLA class I histocompatibility antigen, C alpha chain	0.218	8.01.E-04
Q01970	1-phosphatidylinositol 4,5-bisphosphate phosphodiesterase beta-3	0.219	8.34.E-04
P13797	Plastin-3	0.2	8.42.E-04
P13797-2	Isoform 2 of Plastin-3	0.2	8.42.E-04
P29692-3	Isoform 3 of Elongation factor 1-delta	0.245	8.44.E-04
P13796	Plastin-2	0.201	8.69.E-04
Q12965	Unconventional myosin-Ie	0.217	9.23.E-04
P56747	Claudin-6	0.219	9.92.E-04
P26447	Protein S100-A4	0.154	1.00.E-03
Q2KHR3	Glutamine and serine-rich protein 1	0.116	1.02.E-03
O15347	High mobility group protein B3	0.218	1.10.E-03
Q9NUQ3	Gamma-taxilin	0.188	1.10.E-03
P38646	Stress-70 protein, mitochondrial	0.209	1.16.E-03
Q59GN2	Putative 60S ribosomal protein L39-like 5	0.245	1.21.E-03
P62891	60S ribosomal protein L39	0.245	1.21.E-03
P11169	Solute carrier family 2, facilitated glucose transporter member 3	0.21	1.22.E-03
Q5TH69	Brefeldin A-inhibited guanine nucleotide-exchange protein 3	0.185	1.30.E-03
Q96G01	Protein bicaudal D homolog 1	0.164	1.30.E-03
Q96G01-4	Isoform 4 of Protein bicaudal D homolog 1	0.164	1.30.E-03
Q96G01-3	Isoform 3 of Protein bicaudal D homolog 1	0.164	1.30.E-03
Q15303	Receptor tyrosine-protein kinase erbB-4	0.139	1.33.E-03
Q15303-4	Isoform JM-B CYT-2 of Receptor tyrosine-protein kinase erbB-4	0.139	1.33.E-03
Q15303-2	Isoform JM-B CYT-1 of Receptor tyrosine-protein kinase erbB-4	0.139	1.33.E-03
Q15303-3	Isoform JM-A CYT-2 of Receptor tyrosine-protein kinase erbB-4	0.139	1.33.E-03
P60660-2	Isoform Smooth muscle of Myosin light polypeptide 6	0.226	1.35.E-03
P27105	Erythrocyte band 7 integral membrane protein	0.158	1.46.E-03
Q9P0V3-2	Isoform 2 of SH3 domain-binding protein 4	0.164	1.47.E-03
Q01813-2	Isoform 2 of ATP-dependent 6-phosphofructokinase, platelet type	0.217	1.58.E-03
Q01813	ATP-dependent 6-phosphofructokinase, platelet type	0.217	1.58.E-03
Q8WX94	NACHT, LRR and PYD domains-containing protein 7	0.24	1.60.E-03
Q8WX94-3	Isoform 3 of NACHT, LRR and PYD domains-containing protein 7	0.24	1.60.E-03
Q8WX94-2	Isoform 2 of NACHT, LRR and PYD domains-containing protein 7	0.24	1.60.E-03
Q5T6F2-2	Isoform 2 of Ubiquitin-associated protein 2	0.165	1.61.E-03
P04181	Ornithine aminotransferase, mitochondrial	0.231	1.61.E-03
Q14676	Mediator of DNA damage checkpoint protein 1	0.201	1.62.E-03
Q14676-4	Isoform 4 of Mediator of DNA damage checkpoint protein 1	0.201	1.62.E-03

Q14676-3	Isoform 3 of Mediator of DNA damage checkpoint protein 1	0.201	1.62.E-03
Q14676-2	Isoform 2 of Mediator of DNA damage checkpoint protein 1	0.201	1.62.E-03
P37840-2	Isoform 2-4 of Alpha-synuclein	0.174	1.66.E-03
P37840	Alpha-synuclein	0.174	1.66.E-03
O60885	Bromodomain-containing protein 4	0.177	1.70.E-03
Q9BXF3-3	Isoform C of Cat eye syndrome critical region protein 2	0.177	1.73.E-03
Q9BXF3	Cat eye syndrome critical region protein 2	0.177	1.73.E-03
Q9UBD5-3	Isoform 3 of Origin recognition complex subunit 3	0.191	1.74.E-03
Q9H1E3	Nuclear ubiquitous casein and cyclin-dependent kinase substrate 1	0.249	1.86.E-03
Q9UBD5	Origin recognition complex subunit 3	0.191	1.92.E-03
Q9UBD5-2	Isoform 2 of Origin recognition complex subunit 3	0.191	1.92.E-03
P06454	Prothymosin alpha	0.224	1.96.E-03
P06454-2	Isoform 2 of Prothymosin alpha	0.224	1.96.E-03
P37840-3	Isoform 2-5 of Alpha-synuclein	0.147	1.98.E-03
P14635-2	Isoform 2 of G2/mitotic-specific cyclin-B1	0.187	2.06.E-03
P14635	G2/mitotic-specific cyclin-B1	0.187	2.06.E-03
Q92878-3	Isoform 3 of DNA repair protein RAD50	0.25	2.08.E-03
Q92878-2	Isoform 2 of DNA repair protein RAD50	0.25	2.08.E-03
Q92878	DNA repair protein RAD50	0.25	2.08.E-03
Q6IS14	Eukaryotic translation initiation factor 5A-1-like	0.228	2.23.E-03
C9JQL5	Putative dispanin subfamily A member 2d	0.152	2.37.E-03
O95208-5	Isoform 4 of Epsin-2	0.268	2.38.E-03
Q15642-2	Isoform 2 of Cdc42-interacting protein 4	0.259	2.40.E-03
Q15642	Cdc42-interacting protein 4	0.259	2.40.E-03
P15259	Phosphoglycerate mutase 2	0.249	2.43.E-03
P05114	Non-histone chromosomal protein HMG-14	0.144	2.48.E-03
Q16143	Beta-synuclein	0.174	2.63.E-03
Q15404-2	Isoform 2 of Ras suppressor protein 1	0.25	2.72.E-03
Q99590	Protein SCAF11	0.148	2.76.E-03
Q99590-2	Isoform 2 of Protein SCAF11	0.148	2.76.E-03
P17096-3	Isoform HMG-R of High mobility group protein HMG-I/HMG-Y	0.225	2.90.E-03
P17096	High mobility group protein HMG-I/HMG-Y	0.225	2.90.E-03
P63220	40S ribosomal protein S21	0.236	2.92.E-03
Q92485-2	Isoform 2 of Acid sphingomyelinase-like phosphodiesterase 3b	0.185	2.92.E-03
A6NDB9	Paralemmin-3	0.186	3.00.E-03
Q13740-2	Isoform 2 of CD166 antigen	0.217	3.02.E-03
Q13740	CD166 antigen	0.217	3.02.E-03
Q14192	Four and a half LIM domains protein 2	0.254	3.07.E-03
P09493-5	Isoform 5 of Tropomyosin alpha-1 chain	0.177	3.07.E-03
Q16822-3	Isoform 3 of Phosphoenolpyruvate carboxykinase [GTP], mitochondrial	0.255	3.09.E-03
Q01650	Large neutral amino acids transporter small subunit 1	0.285	3.10.E-03
Q96P48-3	Isoform 3 of Arf-GAP with Rho-GAP domain, ANK repeat and PH domain-containing protein 1	0.206	3.12.E-03
Q96P48	Arf-GAP with Rho-GAP domain, ANK repeat and PH domain-containing protein 1	0.206	3.12.E-03
Q9H6Y2	WD repeat-containing protein 55	0.127	3.22.E-03
Q9H6Y2-2	Isoform 2 of WD repeat-containing protein 55	0.127	3.22.E-03
Q96RL1-3	Isoform 3 of BRCA1-A complex subunit RAP80	0.119	3.29.E-03
Q96RL1-2	Isoform 2 of BRCA1-A complex subunit RAP80	0.119	3.29.E-03
Q96RL1	BRCA1-A complex subunit RAP80	0.119	3.29.E-03
Q96FE5	Leucine-rich repeat and immunoglobulin-like domain-containing nogo receptor-interacting protein 1	0.225	3.42.E-03
Q96FE5-2	Isoform 2 of Leucine-rich repeat and immunoglobulin-like domain-containing nogo receptor-interacting protein 1	0.225	3.42.E-03
Q16822	Phosphoenolpyruvate carboxykinase [GTP], mitochondrial	0.274	3.50.E-03
O95484	Claudin-9	0.194	3.54.E-03
O14493	Claudin-4	0.194	3.54.E-03
O15551	Claudin-3	0.194	3.54.E-03
Q9BTC8	Metastasis-associated protein MTA3	0.261	3.72.E-03
Q9BTC8-2	Isoform 2 of Metastasis-associated protein MTA3	0.261	3.72.E-03
O15533	Tapasin	0.136	3.77.E-03
O15533-3	Isoform 3 of Tapasin	0.136	3.77.E-03
O15533-2	Isoform 2 of Tapasin	0.136	3.77.E-03
Q15404	Ras suppressor protein 1	0.254	3.80.E-03
Q15642-4	Isoform 4 of Cdc42-interacting protein 4	0.273	3.84.E-03
Q15642-3	Isoform 3 of Cdc42-interacting protein 4	0.273	3.84.E-03
Q8N0Y7	Probable phosphoglycerate mutase 4	0.257	4.02.E-03
P40818	Ubiquitin carboxyl-terminal hydrolase 8	0.204	4.15.E-03
P40818-2	Isoform 2 of Ubiquitin carboxyl-terminal hydrolase 8	0.204	4.15.E-03
Q05639	Elongation factor 1-alpha 2	0.281	4.19.E-03
P51911-2	Isoform 2 of Calponin-1	0.202	4.24.E-03
P51911	Calponin-1	0.202	4.24.E-03

P08195-4	Isoform 4 of 4F2 cell-surface antigen heavy chain	0.25	4.28.E-03
P08195-3	Isoform 3 of 4F2 cell-surface antigen heavy chain	0.25	4.28.E-03
P08195	4F2 cell-surface antigen heavy chain	0.25	4.28.E-03
Q9BXB5	Oxysterol-binding protein-related protein 10	0.218	4.58.E-03
Q9BXB5-2	Isoform 2 of Oxysterol-binding protein-related protein 10	0.218	4.58.E-03
P53634-3	Isoform 3 of Dipeptidyl peptidase 1	0.289	4.65.E-03
P53634-2	Isoform 2 of Dipeptidyl peptidase 1	0.289	4.65.E-03
P23588-2	Isoform 2 of Eukaryotic translation initiation factor 4B	0.286	4.70.E-03
P23588	Eukaryotic translation initiation factor 4B	0.286	4.70.E-03
P08195-2	Isoform 2 of 4F2 cell-surface antigen heavy chain	0.253	4.77.E-03
Q9H1E3-2	Isoform 2 of Nuclear ubiquitous casein and cyclin-dependent kinase substrate 1	0.237	4.89.E-03
P18669	Phosphoglycerate mutase 1	0.255	4.94.E-03
Q9BXF3-2	Isoform B of Cat eye syndrome critical region protein 2	0.177	5.05.E-03
P46013	Proliferation marker protein Ki-67	0.217	5.10.E-03
Q9BX66-9	Isoform 9 of Sorbin and SH3 domain-containing protein 1	0.172	5.14.E-03
Q9BX66-8	Isoform 8 of Sorbin and SH3 domain-containing protein 1	0.172	5.14.E-03
Q9BX66-6	Isoform 6 of Sorbin and SH3 domain-containing protein 1	0.172	5.14.E-03
Q9BX66-5	Isoform 5 of Sorbin and SH3 domain-containing protein 1	0.172	5.14.E-03
Q9BX66-4	Isoform 4 of Sorbin and SH3 domain-containing protein 1	0.172	5.14.E-03
Q9BX66-12	Isoform 12 of Sorbin and SH3 domain-containing protein 1	0.172	5.14.E-03
Q9BX66-10	Isoform 10 of Sorbin and SH3 domain-containing protein 1	0.172	5.14.E-03
Q9H910	Jupiter microtubule associated homolog 2	0.289	5.32.E-03
Q9H910-3	Isoform 3 of Jupiter microtubule associated homolog 2	0.289	5.32.E-03
Q13740-4	Isoform 4 of CD166 antigen	0.217	5.44.E-03
P05787	Keratin, type II cytoskeletal 8	0.26	5.62.E-03
P05787-2	Isoform 2 of Keratin, type II cytoskeletal 8	0.26	5.62.E-03
Q9BTT0-3	Isoform 3 of Acidic leucine-rich nuclear phosphoprotein 32 family member E	0.287	5.68.E-03
Q96P48-5	Isoform 5 of Arf-GAP with Rho-GAP domain, ANK repeat and PH domain-containing protein 1	0.22	5.70.E-03
P17540	Creatine kinase S-type, mitochondrial	0.219	5.77.E-03
P07099	Epoxide hydrolase 1	0.301	5.91.E-03
Q9NUQ6-4	Isoform 4 of SPATS2-like protein	0.21	5.94.E-03
Q9NUQ6-3	Isoform 3 of SPATS2-like protein	0.21	5.94.E-03
Q14677-2	Isoform 2 of Clathrin interactor 1	0.32	5.99.E-03
E9PAV3	Nascent polypeptide-associated complex subunit alpha, muscle-specific form	0.259	6.06.E-03
Q13765	Nascent polypeptide-associated complex subunit alpha	0.259	6.06.E-03
E9PAV3-2	Isoform skNAC-2 of Nascent polypeptide-associated complex subunit alpha, muscle-specific form	0.259	6.06.E-03
Q9Y3B9	RRP15-like protein	0.144	6.16.E-03
Q01469	Fatty acid-binding protein 5	0.264	6.23.E-03
P53350	Serine/threonine-protein kinase PLK1	0.285	6.25.E-03
Q9NUQ6	SPATS2-like protein	0.21	6.26.E-03
Q9NUQ6-2	Isoform 2 of SPATS2-like protein	0.21	6.26.E-03
P17706	Tyrosine-protein phosphatase non-receptor type 2	0.239	6.34.E-03
P17706-4	Isoform 4 of Tyrosine-protein phosphatase non-receptor type 2	0.239	6.34.E-03
P17706-3	Isoform 3 of Tyrosine-protein phosphatase non-receptor type 2	0.239	6.34.E-03
P17706-2	Isoform 2 of Tyrosine-protein phosphatase non-receptor type 2	0.239	6.34.E-03
Q96EB6	NAD-dependent protein deacetylase sirtuin-1	0.297	6.41.E-03
P15311	Ezrin	0.265	6.41.E-03
Q96F85-2	Isoform 2 of CB1 cannabinoid receptor-interacting protein 1	0.231	6.47.E-03
Q96F85	CB1 cannabinoid receptor-interacting protein 1	0.231	6.47.E-03
P06703	Protein S100-A6	0.256	6.62.E-03
Q15642-5	Isoform 5 of Cdc42-interacting protein 4	0.273	6.79.E-03
Q9H910-2	Isoform 2 of Jupiter microtubule associated homolog 2	0.299	6.93.E-03
Q02790	Peptidyl-prolyl cis-trans isomerase FKBP4	0.269	7.03.E-03
P31641-2	Isoform 2 of Sodium- and chloride-dependent taurine transporter	0.232	7.10.E-03
Q9NUQ3-2	Isoform 2 of Gamma-taxilin	0.193	7.46.E-03
P49641-2	Isoform 2 of Alpha-mannosidase 2x	0.225	7.61.E-03
P49641-1	Isoform 1 of Alpha-mannosidase 2x	0.225	7.61.E-03
P49641	Alpha-mannosidase 2x	0.225	7.61.E-03
Q9BZK3	Putative nascent polypeptide-associated complex subunit alpha-like protein	0.312	7.73.E-03
O15230	Laminin subunit alpha-5	0.226	7.79.E-03
Q9NW08-2	Isoform 2 of DNA-directed RNA polymerase III subunit RPC2	0.261	8.33.E-03
Q96P48-7	Isoform 7 of Arf-GAP with Rho-GAP domain, ANK repeat and PH domain-containing protein 1	0.22	8.46.E-03
Q96P48-4	Isoform 4 of Arf-GAP with Rho-GAP domain, ANK repeat and PH domain-containing protein 1	0.22	8.46.E-03
Q96P48-2	Isoform 2 of Arf-GAP with Rho-GAP domain, ANK repeat and PH domain-containing protein 1	0.22	8.46.E-03
Q96P48-1	Isoform 1 of Arf-GAP with Rho-GAP domain, ANK repeat and PH domain-containing protein 1	0.22	8.46.E-03
P10909-6	Isoform 6 of Clusterin	0.287	8.65.E-03
P10909-5	Isoform 5 of Clusterin	0.287	8.65.E-03
P10909-4	Isoform 4 of Clusterin	0.287	8.65.E-03
P10909-2	Isoform 2 of Clusterin	0.287	8.65.E-03

P10909	Clusterin	0.287	8.65.E-03
Q9C0C2-2	Isoform 2 of 182 kDa tankyrase-1-binding protein	0.333	8.68.E-03
P24534	Elongation factor 1-beta	0.278	8.83.E-03
Q92882	Osteoclast-stimulating factor 1	0.188	8.87.E-03
Q9NRG0	Chromatin accessibility complex protein 1	0.156	8.94.E-03
P17693-4	Isoform 4 of HLA class I histocompatibility antigen, alpha chain G	0.231	9.06.E-03
Q96HR3	Mediator of RNA polymerase II transcription subunit 30	0.234	9.20.E-03
Q96HR3-2	Isoform 2 of Mediator of RNA polymerase II transcription subunit 30	0.234	9.20.E-03
Q9P258	Protein RCC2	0.296	9.24.E-03
Q14677-3	Isoform 3 of Clathrin interactor 1	0.331	9.26.E-03
Q14677	Clathrin interactor 1	0.331	9.26.E-03
Q13685	Angio-associated migratory cell protein	0.313	9.57.E-03
P06753	Tropomyosin alpha-3 chain	0.336	9.82.E-03
P04626	Receptor tyrosine-protein kinase erbB-2	0.309	9.91.E-03
P04626-5	Isoform 5 of Receptor tyrosine-protein kinase erbB-2	0.309	9.91.E-03
P04626-4	Isoform 4 of Receptor tyrosine-protein kinase erbB-2	0.309	9.91.E-03
Q92777	Synapsin-2	0.23	9.93.E-03
Q92777-2	Isoform IIb of Synapsin-2	0.23	9.93.E-03
P10909-3	Isoform 3 of Clusterin	0.287	1.00.E-02
Q9NX55-3	Isoform 3 of Huntingtin-interacting protein K	0.237	1.01.E-02
P33316	Deoxyuridine 5'-triphosphate nucleotidohydrolase, mitochondrial	0.284	1.01.E-02
P09874	Poly [ADP-ribose] polymerase 1	0.285	1.03.E-02
Q16763	Ubiquitin-conjugating enzyme E2 S	0.247	1.04.E-02
P09493-10	Isoform 10 of Tropomyosin alpha-1 chain	0.306	1.07.E-02
P09493	Tropomyosin alpha-1 chain	0.331	1.11.E-02
P12268	Inosine-5'-monophosphate dehydrogenase 2	0.348	1.15.E-02
Q2VPK5-5	Isoform 3 of Cytoplasmic tRNA 2-thiolation protein 2	0.194	1.15.E-02
P63241	Eukaryotic translation initiation factor 5A-1	0.29	1.15.E-02
P16989-2	Isoform 2 of Y-box-binding protein 3	0.292	1.20.E-02
O60361	Putative nucleoside diphosphate kinase	0.261	1.20.E-02
O43683	Mitotic checkpoint serine/threonine-protein kinase BUB1	0.248	1.22.E-02
O43683-3	Isoform 3 of Mitotic checkpoint serine/threonine-protein kinase BUB1	0.248	1.22.E-02
O43683-2	Isoform 2 of Mitotic checkpoint serine/threonine-protein kinase BUB1	0.248	1.22.E-02
P04626-6	Isoform 6 of Receptor tyrosine-protein kinase erbB-2	0.333	1.22.E-02
Q92833	Protein Jumonji	0.237	1.23.E-02
Q92833-3	Isoform 3 of Protein Jumonji	0.237	1.23.E-02
P84101	Small EDRK-rich factor 2	0.239	1.23.E-02
P84101-4	Isoform 4 of Small EDRK-rich factor 2	0.239	1.23.E-02
P84101-3	Isoform 3 of Small EDRK-rich factor 2	0.239	1.23.E-02
P84101-2	Isoform 2 of Small EDRK-rich factor 2	0.239	1.23.E-02
Q01629	Interferon-induced transmembrane protein 2	0.329	1.24.E-02
P13164	Interferon-induced transmembrane protein 1	0.329	1.24.E-02
Q15398-3	Isoform 3 of Disks large-associated protein 5	0.286	1.25.E-02
Q969T9	WW domain-binding protein 2	0.251	1.28.E-02
Q9NX02-4	Isoform 4 of NACHT, LRR and PYD domains-containing protein 2	0.304	1.29.E-02
Q8N4J0	Carnosine N-methyltransferase	0.22	1.30.E-02
P08729	Keratin, type II cytoskeletal 7	0.358	1.37.E-02
Q9C0C2	182 kDa tankyrase-1-binding protein	0.318	1.38.E-02
Q9NX02-3	Isoform 3 of NACHT, LRR and PYD domains-containing protein 2	0.303	1.38.E-02
P09493-4	Isoform 4 of Tropomyosin alpha-1 chain	0.321	1.40.E-02
P16989	Y-box-binding protein 3	0.3	1.41.E-02
P53634	Dipeptidyl peptidase 1	0.3	1.41.E-02
O76080	AN1-type zinc finger protein 5	0.223	1.45.E-02
P50897-2	Isoform 2 of Palmitoyl-protein thioesterase 1	0.324	1.47.E-02
P08123	Collagen alpha-2(I) chain	0.236	1.47.E-02
Q9NU22	Midasin	0.321	1.47.E-02
Q7Z2W4	Zinc finger CCCH-type antiviral protein 1	0.342	1.47.E-02
Q7Z2W4-2	Isoform 2 of Zinc finger CCCH-type antiviral protein 1	0.342	1.47.E-02
Q14202	Zinc finger MYM-type protein 3	0.264	1.48.E-02
Q14202-3	Isoform 3 of Zinc finger MYM-type protein 3	0.264	1.48.E-02
Q14202-2	Isoform 2 of Zinc finger MYM-type protein 3	0.264	1.48.E-02
Q8WTS1	1-acylglycerol-3-phosphate O-acyltransferase ABHD5	0.214	1.51.E-02
Q9NX02	NACHT, LRR and PYD domains-containing protein 2	0.301	1.56.E-02
Q9NX02-5	Isoform 5 of NACHT, LRR and PYD domains-containing protein 2	0.301	1.56.E-02
Q9NX02-2	Isoform 2 of NACHT, LRR and PYD domains-containing protein 2	0.301	1.56.E-02
Q9GZV4	Eukaryotic translation initiation factor 5A-2	0.305	1.57.E-02
Q9H444	Charged multivesicular body protein 4b	0.297	1.58.E-02
Q969T9-2	Isoform 2 of WW domain-binding protein 2	0.275	1.60.E-02
P04181-2	Isoform 2 of Ornithine aminotransferase, mitochondrial	0.193	1.60.E-02

Q9NRF8	CTP synthase 2	0.345	1.61.E-02
O60934	Nibrin	0.243	1.61.E-02
P06753-5	Isoform 5 of Tropomyosin alpha-3 chain	0.307	1.61.E-02
Q96E39	RNA binding motif protein, X-linked-like-1	0.302	1.62.E-02
Q9NSB2	Keratin, type II cuticular Hb4	0.351	1.64.E-02
Q8TDQ7-3	Isoform 3 of Glucosamine-6-phosphate isomerase 2	0.253	1.64.E-02
Q8TDQ7-2	Isoform 2 of Glucosamine-6-phosphate isomerase 2	0.253	1.64.E-02
P46926-2	Isoform 2 of Glucosamine-6-phosphate isomerase 1	0.253	1.64.E-02
Q8TDQ7	Glucosamine-6-phosphate isomerase 2	0.253	1.64.E-02
P46926	Glucosamine-6-phosphate isomerase 1	0.253	1.64.E-02
A0MZ66-2	Isoform 2 of Shootin-1	0.277	1.65.E-02
Q13740-3	Isoform 3 of CD166 antigen	0.259	1.66.E-02
P50552	Vasodilator-stimulated phosphoprotein	0.371	1.72.E-02
Q5T3I0-3	Isoform 3 of G patch domain-containing protein 4	0.175	1.72.E-02
Q5T3I0	G patch domain-containing protein 4	0.175	1.72.E-02
P63241-2	Isoform 2 of Eukaryotic translation initiation factor 5A-1	0.31	1.72.E-02
P61916-2	Isoform 2 of NPC intracellular cholesterol transporter 2	0.349	1.73.E-02
Q15043	Metal cation symporter ZIP14	0.272	1.73.E-02
Q15043-2	Isoform 3 of Metal cation symporter ZIP14	0.272	1.73.E-02
Q15043-3	Isoform 2 of Metal cation symporter ZIP14	0.272	1.73.E-02
P07339	Cathepsin D	0.323	1.76.E-02
O43493	Trans-Golgi network integral membrane protein 2	0.302	1.76.E-02
O43493-3	Isoform TGN48 of Trans-Golgi network integral membrane protein 2	0.302	1.76.E-02
O43493-7	Isoform 7 of Trans-Golgi network integral membrane protein 2	0.302	1.76.E-02
O43493-5	Isoform 5 of Trans-Golgi network integral membrane protein 2	0.302	1.76.E-02
O43493-4	Isoform 4 of Trans-Golgi network integral membrane protein 2	0.302	1.76.E-02
Q92785	Zinc finger protein ubi-d4	0.27	1.80.E-02
Q92785-2	Isoform 2 of Zinc finger protein ubi-d4	0.27	1.80.E-02
Q93008	Probable ubiquitin carboxyl-terminal hydrolase FAF-X	0.312	1.80.E-02
Q93008-1	Isoform 2 of Probable ubiquitin carboxyl-terminal hydrolase FAF-X	0.312	1.80.E-02
P63267-2	Isoform 2 of Actin, gamma-enteric smooth muscle	0.355	1.82.E-02
P31641	Sodium- and chloride-dependent taurine transporter	0.287	1.83.E-02
Q9BZA5	Putative gamma-taxilin 2	0.188	1.85.E-02
Q9UQN3-2	Isoform 2 of Charged multivesicular body protein 2b	0.203	1.86.E-02
Q9UQN3	Charged multivesicular body protein 2b	0.203	1.86.E-02
Q96PV0	Ras/Rap GTPase-activating protein SynGAP	0.274	1.89.E-02
Q96PV0-4	Isoform 4 of Ras/Rap GTPase-activating protein SynGAP	0.274	1.89.E-02
Q96PV0-3	Isoform 3 of Ras/Rap GTPase-activating protein SynGAP	0.274	1.89.E-02
Q96PV0-2	Isoform 2 of Ras/Rap GTPase-activating protein SynGAP	0.274	1.89.E-02
Q9UJK0	18S rRNA aminocarboxypropyltransferase	0.191	1.92.E-02
Q9HCE3	Zinc finger protein 532	0.269	1.94.E-02
P09493-6	Isoform 6 of Tropomyosin alpha-1 chain	0.356	1.96.E-02
O43768-7	Isoform 7 of Alpha-endosulfine	0.304	1.97.E-02
O43768-6	Isoform 6 of Alpha-endosulfine	0.304	1.97.E-02
O43768-5	Isoform 5 of Alpha-endosulfine	0.304	1.97.E-02
O43768-9	Isoform 9 of Alpha-endosulfine	0.311	1.97.E-02
O43768-4	Isoform 4 of Alpha-endosulfine	0.311	1.97.E-02
O43768-3	Isoform 3 of Alpha-endosulfine	0.311	1.97.E-02
O43768-2	Isoform 2 of Alpha-endosulfine	0.311	1.97.E-02
O43768	Alpha-endosulfine	0.311	1.97.E-02
Q96GD4-3	Isoform 3 of Aurora kinase B	0.253	1.98.E-02
Q8N9N8	Probable RNA-binding protein EIF1AD	0.277	2.03.E-02
P06753-4	Isoform 4 of Tropomyosin alpha-3 chain	0.32	2.06.E-02
P62070-2	Isoform 2 of Ras-related protein R-Ras2	0.366	2.07.E-02
P54289	Voltage-dependent calcium channel subunit alpha-2/delta-1	0.291	2.08.E-02
P54289-5	Isoform 5 of Voltage-dependent calcium channel subunit alpha-2/delta-1	0.291	2.08.E-02
P54289-4	Isoform 4 of Voltage-dependent calcium channel subunit alpha-2/delta-1	0.291	2.08.E-02
P54289-3	Isoform 3 of Voltage-dependent calcium channel subunit alpha-2/delta-1	0.291	2.08.E-02
P54289-2	Isoform 2 of Voltage-dependent calcium channel subunit alpha-2/delta-1	0.291	2.08.E-02
Q8NBF6-2	Isoform 2 of Late secretory pathway protein AVL9 homolog	0.288	2.10.E-02
Q8NEY8-7	Isoform 7 of Periphilin-1	0.192	2.16.E-02
Q9Y6A5	Transforming acidic coiled-coil-containing protein 3	0.372	2.18.E-02
Q9BRP8	Partner of Y14 and mago	0.391	2.22.E-02
Q9BRP8-2	Isoform 2 of Partner of Y14 and mago	0.391	2.22.E-02
Q7Z2W4-3	Isoform 3 of Zinc finger CCCH-type antiviral protein 1	0.342	2.22.E-02
Q15398-1	Isoform 2 of Disks large-associated protein 5	0.331	2.24.E-02
Q15398	Disks large-associated protein 5	0.331	2.24.E-02
Q13572	Inositol-tetrakisphosphate 1-kinase	0.344	2.27.E-02
P06753-2	Isoform 2 of Tropomyosin alpha-3 chain	0.325	2.27.E-02

P57764	Gasdermin-D	0.317	2.29.E-02
P06753-7	Isoform 7 of Tropomyosin alpha-3 chain	0.327	2.33.E-02
P05783	Keratin, type I cytoskeletal 18	0.327	2.35.E-02
Q9NQP4	Prefoldin subunit 4	0.328	2.42.E-02
Q9Y223-4	Isoform 4 of Bifunctional UDP-N-acetylglucosamine 2-epimerase/N-acetylmannosamine kinase	0.283	2.45.E-02
P06753-3	Isoform 3 of Tropomyosin alpha-3 chain	0.33	2.46.E-02
Q12841-2	Isoform 2 of Follistatin-related protein 1	0.282	2.47.E-02
Q12841	Follistatin-related protein 1	0.282	2.47.E-02
Q9H2E6	Semaphorin-6A	0.292	2.50.E-02
Q9H2E6-2	Isoform 2 of Semaphorin-6A	0.292	2.50.E-02
P09234	U1 small nuclear ribonucleoprotein C	0.295	2.51.E-02
Q14978-3	Isoform 3 of Nucleolar and coiled-body phosphoprotein 1	0.375	2.51.E-02
Q9NZM3-2	Isoform 2 of Intersectin-2	0.289	2.52.E-02
Q9NZM3	Intersectin-2	0.289	2.52.E-02
Q96AC1-2	Isoform 2 of Fermitin family homolog 2	0.343	2.53.E-02
O14497-3	Isoform 3 of AT-rich interactive domain-containing protein 1A	0.34	2.56.E-02
P99999	Cytochrome c	0.346	2.58.E-02
O75475-3	Isoform 3 of PC4 and SFRS1-interacting protein	0.284	2.60.E-02
O75475-2	Isoform 2 of PC4 and SFRS1-interacting protein	0.284	2.60.E-02
P22455-3	Isoform 3 of Fibroblast growth factor receptor 4	0.306	2.61.E-02
P22455-2	Isoform 2 of Fibroblast growth factor receptor 4	0.306	2.61.E-02
P22455	Fibroblast growth factor receptor 4	0.306	2.61.E-02
Q9Y2X9	Zinc finger protein 281	0.263	2.63.E-02
Q9Y2X9-2	Isoform 2 of Zinc finger protein 281	0.263	2.63.E-02
Q96C01	Protein FAM136A	0.381	2.63.E-02
Q9HC62	Sentrin-specific protease 2	0.208	2.65.E-02
Q9HC62-2	Isoform 2 of Sentrin-specific protease 2	0.208	2.65.E-02
Q8TE77-2	Isoform 2 of Protein phosphatase Slingshot homolog 3	0.293	2.65.E-02
P04626-3	Isoform 3 of Receptor tyrosine-protein kinase erbB-2	0.361	2.66.E-02
P04626-2	Isoform 2 of Receptor tyrosine-protein kinase erbB-2	0.361	2.66.E-02
O43399	Tumor protein D54	0.364	2.66.E-02
O43399-7	Isoform 7 of Tumor protein D54	0.364	2.66.E-02
O43399-5	Isoform 5 of Tumor protein D54	0.364	2.66.E-02
Q9NXC5-2	Isoform 2 of GATOR complex protein MIOS	0.262	2.69.E-02
P06213	Insulin receptor	0.262	2.70.E-02
O43581-5	Isoform 5 of Synaptotagmin-7	0.171	2.72.E-02
O43581-3	Isoform 3 of Synaptotagmin-7	0.171	2.72.E-02
O43581-2	Isoform 2 of Synaptotagmin-7	0.171	2.72.E-02
P60174-4	Isoform 4 of Triosephosphate isomerase	0.336	2.73.E-02
P28066-2	Isoform 2 of Proteasome subunit alpha type-5	0.399	2.74.E-02
Q9NZN4-2	Isoform 2 of EH domain-containing protein 2	0.235	2.83.E-02
Q9NZN4	EH domain-containing protein 2	0.235	2.83.E-02
Q13572-2	Isoform 2 of Inositol-tetrakisphosphate 1-kinase	0.334	2.83.E-02
O00507	Probable ubiquitin carboxyl-terminal hydrolase FAF-Y	0.339	2.87.E-02
Q8NFC6	Biorientation of chromosomes in cell division protein 1-like 1	0.335	2.91.E-02
Q96F24	Nuclear receptor-binding factor 2	0.302	2.92.E-02
Q96F24-3	Isoform 3 of Nuclear receptor-binding factor 2	0.302	2.92.E-02
Q06210-2	Isoform 2 of Glutamine--fructose-6-phosphate aminotransferase [isomerizing] 1	0.34	2.92.E-02
Q06210	Glutamine--fructose-6-phosphate aminotransferase [isomerizing] 1	0.34	2.92.E-02
Q14978	Nucleolar and coiled-body phosphoprotein 1	0.384	2.94.E-02
Q14978-2	Isoform Beta of Nucleolar and coiled-body phosphoprotein 1	0.384	2.94.E-02
Q9NZN8-5	Isoform 5 of CCR4-NOT transcription complex subunit 2	0.329	2.94.E-02
Q7Z3K3-4	Isoform 4 of Pogo transposable element with ZNF domain	0.293	2.95.E-02
Q96AC1-3	Isoform 3 of Fermitin family homolog 2	0.348	2.95.E-02
Q96AC1	Fermitin family homolog 2	0.348	2.95.E-02
Q13574-7	Isoform 7 of Diacylglycerol kinase zeta	0.186	2.96.E-02
Q13574-6	Isoform 6 of Diacylglycerol kinase zeta	0.186	2.96.E-02
Q13574-5	Isoform 5 of Diacylglycerol kinase zeta	0.186	2.96.E-02
Q13574-4	Isoform 4 of Diacylglycerol kinase zeta	0.186	2.96.E-02
Q13574-3	Isoform 3 of Diacylglycerol kinase zeta	0.186	2.96.E-02
Q13574-1	Isoform 2 of Diacylglycerol kinase zeta	0.186	2.96.E-02
Q13574	Diacylglycerol kinase zeta	0.186	2.96.E-02
O75717-2	Isoform 2 of WD repeat and HMG-box DNA-binding protein 1	0.357	2.97.E-02
O00507-2	Isoform Short of Probable ubiquitin carboxyl-terminal hydrolase FAF-Y	0.341	2.98.E-02
Q8TAP9	M-phase-specific PLK1-interacting protein	0.272	3.00.E-02
Q8IUC4	Rhophilin-2	0.319	3.01.E-02
Q8IUC4-2	Isoform 2 of Rhophilin-2	0.319	3.01.E-02
P82979	SAP domain-containing ribonucleoprotein	0.39	3.03.E-02
P11388-4	Isoform 4 of DNA topoisomerase 2-alpha	0.351	3.04.E-02

P11388-3	Isoform 3 of DNA topoisomerase 2-alpha	0.351	3.04.E-02
P11388-2	Isoform 2 of DNA topoisomerase 2-alpha	0.351	3.04.E-02
P11388	DNA topoisomerase 2-alpha	0.351	3.04.E-02
A1L390	Pleckstrin homology domain-containing family G member 3	0.311	3.06.E-02
A1L390-3	Isoform 3 of Pleckstrin homology domain-containing family G member 3	0.311	3.06.E-02
O43493-6	Isoform 6 of Trans-Golgi network integral membrane protein 2	0.286	3.06.E-02
Q8NBF6	Late secretory pathway protein AVL9 homolog	0.34	3.09.E-02
Q8WW12-2	Isoform 2 of PEST proteolytic signal-containing nuclear protein	0.383	3.12.E-02
Q96RD6	Pannexin-2	0.36	3.14.E-02
Q96RD6-2	Isoform 2 of Pannexin-2	0.36	3.14.E-02
Q96RD6-1	Isoform 1 of Pannexin-2	0.36	3.14.E-02
P49411	Elongation factor Tu, mitochondrial	0.345	3.15.E-02
P02792	Ferritin light chain	0.294	3.18.E-02
Q9BTK6	PAXIP1-associated glutamate-rich protein 1	0.194	3.19.E-02
A8MUU1	Putative fatty acid-binding protein 5-like protein 3	0.394	3.22.E-02
Q99543-2	Isoform 2 of DnaJ homolog subfamily C member 2	0.373	3.23.E-02
Q13416	Origin recognition complex subunit 2	0.271	3.23.E-02
O14683	Tumor protein p53-inducible protein 11	0.4	3.27.E-02
Q99661	Kinesin-like protein KIF2C	0.325	3.31.E-02
Q99543	DnaJ homolog subfamily C member 2	0.391	3.33.E-02
Q13428-5	Isoform 5 of Treacle protein	0.386	3.34.E-02
Q9UI95	Mitotic spindle assembly checkpoint protein MAD2B	0.311	3.46.E-02
Q15437	Protein transport protein Sec23B	0.401	3.52.E-02
Q92764	Keratin, type I cuticular Ha5	0.385	3.55.E-02
Q6KB66	Keratin, type II cytoskeletal 80	0.378	3.56.E-02
Q6KB66-3	Isoform 3 of Keratin, type II cytoskeletal 80	0.378	3.56.E-02
Q6KB66-2	Isoform 2 of Keratin, type II cytoskeletal 80	0.378	3.56.E-02
Q9GZN7	Protein rogdi homolog	0.315	3.60.E-02
P50897	Palmitoyl-protein thioesterase 1	0.401	3.60.E-02
Q9HD15	Steroid receptor RNA activator 1	0.331	3.63.E-02
P85298	Rho GTPase-activating protein 8	0.305	3.66.E-02
P85298-5	Isoform 5 of Rho GTPase-activating protein 8	0.305	3.66.E-02
P85298-4	Isoform 4 of Rho GTPase-activating protein 8	0.305	3.66.E-02
P85298-3	Isoform 3 of Rho GTPase-activating protein 8	0.305	3.66.E-02
P85298-2	Isoform 2 of Rho GTPase-activating protein 8	0.305	3.66.E-02
Q13428	Treacle protein	0.372	3.67.E-02
Q13428-7	Isoform 7 of Treacle protein	0.372	3.67.E-02
Q13428-6	Isoform 6 of Treacle protein	0.372	3.67.E-02
Q13428-4	Isoform 4 of Treacle protein	0.372	3.67.E-02
Q13428-3	Isoform 3 of Treacle protein	0.372	3.67.E-02
Q8N4C6	Ninein	0.337	3.67.E-02
Q8N4C6-11	Isoform 9 of Ninein	0.337	3.67.E-02
Q8N4C6-9	Isoform 8 of Ninein	0.337	3.67.E-02
Q8N4C6-7	Isoform 7 of Ninein	0.337	3.67.E-02
Q8N4C6-6	Isoform 6 of Ninein	0.337	3.67.E-02
Q8N4C6-5	Isoform 5 of Ninein	0.337	3.67.E-02
Q8N4C6-4	Isoform 4 of Ninein	0.337	3.67.E-02
Q8N4C6-10	Isoform 3 of Ninein	0.337	3.67.E-02
Q8N4C6-2	Isoform 2 of Ninein	0.337	3.67.E-02
Q2NKX8	DNA excision repair protein ERCC-6-like	0.333	3.69.E-02
P52292	Importin subunit alpha-1	0.355	3.70.E-02
P61916	NPC intracellular cholesterol transporter 2	0.426	3.70.E-02
P06748-3	Isoform 3 of Nucleophosmin	0.355	3.70.E-02
O75717	WD repeat and HMG-box DNA-binding protein 1	0.355	3.73.E-02
Q9P2E9	Ribosome-binding protein 1	0.356	3.73.E-02
Q9P2E9-3	Isoform 2 of Ribosome-binding protein 1	0.356	3.73.E-02
Q8TE77	Protein phosphatase Slingshot homolog 3	0.347	3.74.E-02
Q8TE77-3	Isoform 3 of Protein phosphatase Slingshot homolog 3	0.347	3.74.E-02
Q02487-2	Isoform 2B of Desmocollin-2	0.294	3.78.E-02
Q02487	Desmocollin-2	0.294	3.78.E-02
P04439-2	Isoform 2 of HLA class I histocompatibility antigen, A alpha chain	0.405	3.81.E-02
P04439	HLA class I histocompatibility antigen, A alpha chain	0.405	3.81.E-02
P62253	Ubiquitin-conjugating enzyme E2 G1	0.395	3.85.E-02
Q96EB6-2	Isoform 2 of NAD-dependent protein deacetylase sirtuin-1	0.395	3.87.E-02
P61604	10 kDa heat shock protein, mitochondrial	0.359	3.95.E-02
P06748	Nucleophosmin	0.359	3.96.E-02
P06748-2	Isoform 2 of Nucleophosmin	0.359	3.96.E-02
Q9Y485	DmX-like protein 1	0.203	4.01.E-02
Q9NZZ3-2	Isoform 2 of Charged multivesicular body protein 5	0.336	4.02.E-02

Q9NZZ3	Charged multivesicular body protein 5	0.336	4.02.E-02
P60174	Triosephosphate isomerase	0.361	4.04.E-02
P60174-1	Isoform 2 of Triosephosphate isomerase	0.361	4.04.E-02
Q6P1M3	LLGL scribble cell polarity complex component 2	0.329	4.06.E-02
Q6P1M3-2	Isoform A of LLGL scribble cell polarity complex component 2	0.329	4.06.E-02
Q15058	Kinesin-like protein KIF14	0.317	4.07.E-02
O00443-2	Isoform 2 of Phosphatidylinositol 4-phosphate 3-kinase C2 domain-containing subunit alpha	0.318	4.08.E-02
P48431	Transcription factor SOX-2	0.369	4.10.E-02
P56545-3	Isoform 3 of C-terminal-binding protein 2	0.374	4.11.E-02
P56545-2	Isoform 2 of C-terminal-binding protein 2	0.374	4.11.E-02
P56545	C-terminal-binding protein 2	0.374	4.11.E-02
Q8N108-19	Isoform 9 of Mesoderm induction early response protein 1	0.325	4.11.E-02
P06213-2	Isoform Short of Insulin receptor	0.264	4.13.E-02
P27695	DNA-(apurinic or apyrimidinic site) endonuclease	0.421	4.20.E-02
Q86V59	Paraneoplastic antigen-like protein 8A	0.238	4.22.E-02
P48163	NADP-dependent malic enzyme	0.336	4.25.E-02
Q8ND30	Liprin-beta-2	0.2	4.26.E-02
Q86V48	Leucine zipper protein 1	0.351	4.28.E-02
Q86V48-3	Isoform 3 of Leucine zipper protein 1	0.351	4.28.E-02
Q86V48-2	Isoform 2 of Leucine zipper protein 1	0.351	4.28.E-02
P05413	Fatty acid-binding protein, heart	0.384	4.29.E-02
O75607	Nucleoplasmin-3	0.414	4.31.E-02
Q7Z406-4	Isoform 4 of Myosin-14	0.387	4.35.E-02
O14757-3	Isoform 3 of Serine/threonine-protein kinase Chk1	0.38	4.40.E-02
Q8TDZ2-4	Isoform 4 of [F-actin]-monooxygenase MICAL1	0.406	4.41.E-02
Q8TDZ2	[F-actin]-monooxygenase MICAL1	0.406	4.41.E-02
Q13428-8	Isoform 8 of Treacle protein	0.388	4.44.E-02
Q13428-2	Isoform 2 of Treacle protein	0.388	4.44.E-02
Q9UHR4	Brain-specific angiogenesis inhibitor 1-associated protein 2-like protein 1	0.306	4.55.E-02
O14757	Serine/threonine-protein kinase Chk1	0.385	4.55.E-02
O14757-2	Isoform 2 of Serine/threonine-protein kinase Chk1	0.385	4.55.E-02
Q9NVA2	Septin-11	0.301	4.69.E-02
Q9NVA2-2	Isoform 2 of Septin-11	0.301	4.69.E-02
Q9Y2S6	Translation machinery-associated protein 7	0.391	4.72.E-02
Q01658	Protein Dr1	0.354	4.74.E-02
Q8N108	Mesoderm induction early response protein 1	0.331	4.75.E-02
Q8N108-18	Isoform 8 of Mesoderm induction early response protein 1	0.331	4.75.E-02
Q8N108-17	Isoform 7 of Mesoderm induction early response protein 1	0.331	4.75.E-02
Q8N108-16	Isoform 6 of Mesoderm induction early response protein 1	0.331	4.75.E-02
Q8N108-15	Isoform 5 of Mesoderm induction early response protein 1	0.331	4.75.E-02
Q8N108-14	Isoform 4 of Mesoderm induction early response protein 1	0.331	4.75.E-02
Q8N108-13	Isoform 3 of Mesoderm induction early response protein 1	0.331	4.75.E-02
Q8N108-12	Isoform 2 of Mesoderm induction early response protein 1	0.331	4.75.E-02
Q01804	OTU domain-containing protein 4	0.361	4.80.E-02
Q01804-3	Isoform 3 of OTU domain-containing protein 4	0.361	4.80.E-02
Q01804-5	Isoform 2 of OTU domain-containing protein 4	0.361	4.80.E-02
P08590	Myosin light chain 3	0.426	4.81.E-02
P05976	Myosin light chain 1/3, skeletal muscle isoform	0.426	4.81.E-02
P05976-2	Isoform MLC3 of Myosin light chain 1/3, skeletal muscle isoform	0.426	4.81.E-02
Q6NXG1-4	Isoform 4 of Epithelial splicing regulatory protein 1	0.423	4.84.E-02
Q6NXG1-2	Isoform 2 of Epithelial splicing regulatory protein 1	0.423	4.84.E-02
P17931	Galectin-3	0.312	4.85.E-02
P53999	Activated RNA polymerase II transcriptional coactivator p15	0.423	4.89.E-02
Q96NW4	Ankyrin repeat domain-containing protein 27	0.277	4.91.E-02
P11362-4	Isoform 8 of Fibroblast growth factor receptor 1	0.403	4.94.E-02
P11362-3	Isoform 6 of Fibroblast growth factor receptor 1	0.403	4.94.E-02
P11362-2	Isoform 4 of Fibroblast growth factor receptor 1	0.403	4.94.E-02
P11362-21	Isoform 21 of Fibroblast growth factor receptor 1	0.403	4.94.E-02
P11362-20	Isoform 20 of Fibroblast growth factor receptor 1	0.403	4.94.E-02
P11362-19	Isoform 19 of Fibroblast growth factor receptor 1	0.403	4.94.E-02
P11362-14	Isoform 15 of Fibroblast growth factor receptor 1	0.403	4.94.E-02
P11362-7	Isoform 14 of Fibroblast growth factor receptor 1	0.403	4.94.E-02
P11362-6	Isoform 12 of Fibroblast growth factor receptor 1	0.403	4.94.E-02
P11362-5	Isoform 10 of Fibroblast growth factor receptor 1	0.403	4.94.E-02
P11362	Fibroblast growth factor receptor 1	0.403	4.94.E-02
Q6NXG1-3	Isoform 3 of Epithelial splicing regulatory protein 1	0.423	4.97.E-02
Q6NXG1	Epithelial splicing regulatory protein 1	0.423	4.98.E-02
Q9Y223-2	Isoform 2 of Bifunctional UDP-N-acetylglucosamine 2-epimerase/N-acetylmannosamine kinase	0.356	4.99.E-02
Q9Y223	Bifunctional UDP-N-acetylglucosamine 2-epimerase/N-acetylmannosamine kinase	0.356	4.99.E-02

Q58FF7	Putative heat shock protein HSP 90-beta-3	0.376	5.00.E-02

Supplementary Table S1. (e) Proteins with decreased levels in the NPCs (<0.5 fold)

Accession	Protein Name	Abundance Ratio: (NPC) / (iPSC)	Abundance Ratio p-Value: (NPC) / (iPSC)
Q9NVI1	Fanconi anemia group I protein	0.336	4.88.E-02
O60739	Eukaryotic translation initiation factor 1b	0.333	4.42.E-02
P19012	Keratin, type I cytoskeletal 15	0.331	4.53.E-02
Q9NVI1-1	Isoform 1 of Fanconi anemia group I protein	0.331	4.77.E-02
Q9NVI1-2	Isoform 2 of Fanconi anemia group I protein	0.331	4.77.E-02
P49321-4	Isoform 4 of Nuclear autoantigenic sperm protein	0.328	4.67.E-02
O14683	Tumor protein p53-inducible protein 11	0.328	4.73.E-02
P07384	Calpain-1 catalytic subunit	0.326	4.46.E-02
Q9UN86	Ras GTPase-activating protein-binding protein 2	0.322	4.10.E-02
Q92667	A-kinase anchor protein 1, mitochondrial	0.322	3.88.E-02
A2RTX5	Threonine--tRNA ligase 2, cytoplasmic	0.322	4.79.E-02
A2RTX5-2	Isoform 2 of Threonine--tRNA ligase 2, cytoplasmic	0.322	4.79.E-02
P10620	Microsomal glutathione S-transferase 1	0.321	4.06.E-02
P10620-2	Isoform 2 of Microsomal glutathione S-transferase 1	0.321	4.07.E-02
P18583-10	Isoform J of Protein SON	0.32	3.57.E-02
P18583-3	Isoform B of Protein SON	0.32	3.57.E-02
P18583-4	Isoform C of Protein SON	0.32	3.57.E-02
P18583-6	Isoform E of Protein SON	0.32	3.78.E-02
Q96AC1-2	Isoform 2 of Fermitin family homolog 2	0.319	4.37.E-02
Q9C0A0-2	Isoform 2 of Contactin-associated protein-like 4	0.318	4.56.E-02
Q9C0A0	Contactin-associated protein-like 4	0.318	4.56.E-02
Q9BRP8	Partner of Y14 and mago	0.317	3.64.E-02
Q9BRP8-2	Isoform 2 of Partner of Y14 and mago	0.317	3.64.E-02
P13796	Plastin-2	0.316	3.75.E-02
P23193	Transcription elongation factor A protein 1	0.311	2.67.E-02
P13797	Plastin-3	0.31	3.34.E-02
O00151	PDZ and LIM domain protein 1	0.31	2.82.E-02
Q14126	Desmoglein-2	0.31	2.81.E-02
Q8IZL8	Proline-, glutamic acid- and leucine-rich protein 1	0.309	2.86.E-02
Q58FF7	Putative heat shock protein HSP 90-beta-3	0.308	3.20.E-02
P13797-2	Isoform 2 of Plastin-3	0.308	3.23.E-02
P0DN79	Cystathionine beta-synthase-like protein	0.308	3.22.E-02
P35520-2	Isoform 2 of Cystathionine beta-synthase	0.308	3.22.E-02
P13797-3	Isoform 3 of Plastin-3	0.307	3.17.E-02
Q9UN86-2	Isoform B of Ras GTPase-activating protein-binding protein 2	0.306	2.99.E-02
Q99543	DnaJ homolog subfamily C member 2	0.306	4.74.E-02
Q9BX40	Protein LSM14 homolog B	0.305	4.23.E-02
P49642	DNA primase small subunit	0.305	3.72.E-02
Q02790	Peptidyl-prolyl cis-trans isomerase FKBP4	0.304	2.96.E-02
Q13283	Ras GTPase-activating protein-binding protein 1	0.301	2.77.E-02
Q14117	Dihydropyrimidinase	0.3	2.30.E-02
P52789	Hexokinase-2	0.299	2.97.E-02
Q9P1Y5-2	Isoform 2 of Calmodulin-regulated spectrin-associated protein 3	0.299	4.36.E-02
Q9P1Y5	Calmodulin-regulated spectrin-associated protein 3	0.299	4.36.E-02
Q8NC51-3	Isoform 3 of Plasminogen activator inhibitor 1 RNA-binding protein	0.297	2.01.E-02
Q8NC51-4	Isoform 4 of Plasminogen activator inhibitor 1 RNA-binding protein	0.297	2.01.E-02
O43399-5	Isoform 5 of Tumor protein D54	0.296	3.70.E-02
O43399-7	Isoform 7 of Tumor protein D54	0.296	3.70.E-02
O43399	Tumor protein D54	0.296	3.70.E-02
P35900	Keratin, type I cytoskeletal 20	0.296	3.08.E-02
Q8N1A0	Keratin-like protein KRT222	0.296	3.08.E-02
Q8N1A0-2	Isoform 2 of Keratin-like protein KRT222	0.296	3.08.E-02
Q99543-2	Isoform 2 of DnaJ homolog subfamily C member 2	0.295	4.21.E-02
P62841	40S ribosomal protein S15	0.292	2.26.E-02
Q7Z2W4-3	Isoform 3 of Zinc finger CCCH-type antiviral protein 1	0.292	4.59.E-02
O60343-3	Isoform 3 of TBC1 domain family member 4	0.289	3.79.E-02
O60343	TBC1 domain family member 4	0.289	3.79.E-02
O60343-2	Isoform 2 of TBC1 domain family member 4	0.289	3.79.E-02
P38646	Stress-70 protein, mitochondrial	0.288	2.14.E-02
P08727	Keratin, type I cytoskeletal 19	0.287	2.05.E-02
O00507-2	Isoform Short of Probable ubiquitin carboxyl-terminal hydrolase FAF-Y	0.285	1.96.E-02
Q8WU90	Zinc finger CCCH domain-containing protein 15	0.285	4.77.E-02
O00507	Probable ubiquitin carboxyl-terminal hydrolase FAF-Y	0.284	1.94.E-02
Q9BX40-2	Isoform 2 of Protein LSM14 homolog B	0.284	2.40.E-02
Q13572	Inositol-tetrakisphosphate 1-kinase	0.284	3.70.E-02
P49643	DNA primase large subunit	0.284	3.87.E-02
P60953-1	Isoform 1 of Cell division control protein 42 homolog	0.283	1.49.E-02
Q96E17	Ras-related protein Rab-3C	0.283	3.82.E-02

Q13542	Eukaryotic translation initiation factor 4E-binding protein 2	0.283	3.89.E-02
P07339	Cathepsin D	0.282	2.21.E-02
P11233	Ras-related protein Ral-A	0.282	3.58.E-02
C9JLW8	Mapk-regulated corepressor-interacting protein 1	0.282	4.41.E-02
Q04695	Keratin, type I cytoskeletal 17	0.281	1.81.E-02
Q9BTC8-2	Isoform 2 of Metastasis-associated protein MTA3	0.281	3.24.E-02
Q9BTC8	Metastasis-associated protein MTA3	0.281	3.24.E-02
Q9NRZ9-4	Isoform 4 of Lymphoid-specific helicase	0.278	3.71.E-02
P60953	Cell division control protein 42 homolog	0.277	1.84.E-02
P33991	DNA replication licensing factor MCM4	0.277	1.63.E-02
Q15398-1	Isoform 2 of Disks large-associated protein 5	0.276	3.52.E-02
Q15398	Disks large-associated protein 5	0.276	3.52.E-02
P23588	Eukaryotic translation initiation factor 4B	0.275	1.25.E-02
P23588-2	Isoform 2 of Eukaryotic translation initiation factor 4B	0.275	1.25.E-02
Q9NX55	Huntingtin-interacting protein K	0.273	2.86.E-02
Q86X29-3	Isoform 3 of Lipolysis-stimulated lipoprotein receptor	0.273	3.59.E-02
P04216	Thy-1 membrane glycoprotein	0.272	3.31.E-02
P61916	NPC intracellular cholesterol transporter 2	0.271	1.56.E-02
Q9P258	Protein RCC2	0.27	1.24.E-02
Q14157-3	Isoform 3 of Ubiquitin-associated protein 2-like	0.269	1.27.E-02
Q14157-4	Isoform 4 of Ubiquitin-associated protein 2-like	0.269	1.27.E-02
Q14157	Ubiquitin-associated protein 2-like	0.269	1.27.E-02
Q14157-5	Isoform 5 of Ubiquitin-associated protein 2-like	0.269	1.27.E-02
Q14157-1	Isoform 2 of Ubiquitin-associated protein 2-like	0.269	1.27.E-02
Q15398-3	Isoform 3 of Disks large-associated protein 5	0.269	3.29.E-02
P50552	Vasodilator-stimulated phosphoprotein	0.268	1.34.E-02
P63241-2	Isoform 2 of Eukaryotic translation initiation factor 5A-1	0.267	1.30.E-02
P63241	Eukaryotic translation initiation factor 5A-1	0.267	1.30.E-02
Q96EP5-2	Isoform 2 of DAZ-associated protein 1	0.266	1.29.E-02
Q96EP5	DAZ-associated protein 1	0.266	1.29.E-02
Q93008	Probable ubiquitin carboxyl-terminal hydrolase FAF-X	0.265	1.23.E-02
Q93008-1	Isoform 2 of Probable ubiquitin carboxyl-terminal hydrolase FAF-X	0.265	1.23.E-02
P13646	Keratin, type I cytoskeletal 13	0.262	1.31.E-02
P13646-3	Isoform 3 of Keratin, type I cytoskeletal 13	0.262	1.31.E-02
P06748	Nucleophosmin	0.261	1.11.E-02
P06748-2	Isoform 2 of Nucleophosmin	0.261	1.11.E-02
P20290	Transcription factor BTF3	0.261	2.89.E-02
P20290-2	Isoform 2 of Transcription factor BTF3	0.261	2.89.E-02
P19012-2	Isoform 2 of Keratin, type I cytoskeletal 15	0.26	1.00.E-02
O95905-3	Isoform 3 of Protein ecdysoneless homolog	0.26	1.98.E-02
O95905	Protein ecdysoneless homolog	0.26	1.98.E-02
P50897-2	Isoform 2 of Palmitoyl-protein thioesterase 1	0.258	2.12.E-02
P42766	60S ribosomal protein L35	0.257	9.86.E-03
Q9NRZ9-7	Isoform 7 of Lymphoid-specific helicase	0.257	2.27.E-02
Q9NRZ9-8	Isoform 8 of Lymphoid-specific helicase	0.257	2.29.E-02
Q99661	Kinesin-like protein KIF2C	0.254	4.27.E-02
Q6IS14	Eukaryotic translation initiation factor 5A-1-like	0.253	8.82.E-03
O14777	Kinetochore protein NDC80 homolog	0.252	2.37.E-02
Q96KP1	Exocyst complex component 2	0.252	2.51.E-02
P27105	Erythrocyte band 7 integral membrane protein	0.252	4.77.E-02
Q99614	Tetratricopeptide repeat protein 1	0.25	2.04.E-02
P25205	DNA replication licensing factor MCM3	0.249	7.90.E-03
Q96T23-2	Isoform 2 of Remodeling and spacing factor 1	0.249	3.55.E-02
Q96T23	Remodeling and spacing factor 1	0.249	3.55.E-02
P25205-2	Isoform 2 of DNA replication licensing factor MCM3	0.248	7.60.E-03
A0A075B767	Peptidyl-prolyl cis-trans isomerase A-like 4H	0.248	3.59.E-02
Q9NVR5	Protein kintoun	0.247	4.94.E-02
P40121	Macrophage-capping protein	0.244	1.54.E-02
P40121-2	Isoform 2 of Macrophage-capping protein	0.244	1.54.E-02
P33993-2	Isoform 2 of DNA replication licensing factor MCM7	0.243	6.61.E-03
Q9GZV4	Eukaryotic translation initiation factor 5A-2	0.242	6.49.E-03
Q96QD8	Sodium-coupled neutral amino acid transporter 2	0.242	1.47.E-02
Q01469	Fatty acid-binding protein 5	0.241	6.20.E-03
Q14978-3	Isoform 3 of Nucleolar and coiled-body phosphoprotein 1	0.241	5.39.E-03
Q9H1E3	Nuclear ubiquitous casein and cyclin-dependent kinase substrate 1	0.241	2.22.E-02
Q9NRZ9-5	Isoform 5 of Lymphoid-specific helicase	0.241	1.73.E-02
Q9NRZ9-3	Isoform 3 of Lymphoid-specific helicase	0.241	1.79.E-02
Q9NRZ9-2	Isoform 2 of Lymphoid-specific helicase	0.241	1.73.E-02
Q9NRZ9	Lymphoid-specific helicase	0.241	1.73.E-02
Q9NRZ9-6	Isoform 6 of Lymphoid-specific helicase	0.241	1.79.E-02
P06748-3	Isoform 3 of Nucleophosmin	0.24	6.05.E-03

Q8TDZ2	[F-actin]-monooxygenase MICAL1	0.24	1.83.E-02
Q8TDZ2-4	Isoform 4 of [F-actin]-monooxygenase MICAL1	0.24	1.83.E-02
Q8TDZ2-2	Isoform 2 of [F-actin]-monooxygenase MICAL1	0.24	2.56.E-02
P00374	Dihydrofolate reductase	0.239	6.72.E-03
Q9Y3Z3	Deoxynucleoside triphosphate triphosphohydrolase SAMHD1	0.238	5.48.E-03
Q9Y3Z3-4	Isoform 4 of Deoxynucleoside triphosphate triphosphohydrolase SAMHD1	0.238	6.78.E-03
Q14195-2	Isoform LCRMP-4 of Dihydropyrimidinase-related protein 3	0.237	5.56.E-03
Q14566	DNA replication licensing factor MCM6	0.237	5.53.E-03
Q9NVU7-2	Isoform 2 of Protein SDA1 homolog	0.237	2.27.E-02
P33993	DNA replication licensing factor MCM7	0.237	5.50.E-03
Q9UK22	F-box only protein 2	0.237	2.47.E-02
P47813	Eukaryotic translation initiation factor 1A, X-chromosomal	0.236	5.60.E-03
Q9H1E3-2	Isoform 2 of Nuclear ubiquitous casein and cyclin-dependent kinase substrate 1	0.236	1.13.E-02
Q12965	Unconventional myosin-Ie	0.234	2.31.E-02
Q9GZU8	PSME3-interacting protein	0.234	4.89.E-02
Q96F24	Nuclear receptor-binding factor 2	0.233	4.53.E-02
Q96F24-3	Isoform 3 of Nuclear receptor-binding factor 2	0.233	4.53.E-02
Q9UN81	LINE-1 retrotransposable element ORF1 protein	0.232	3.90.E-02
Q8TE77	Protein phosphatase Slingshot homolog 3	0.23	3.96.E-02
O95453	Poly(A)-specific ribonuclease PARN	0.23	4.53.E-02
O95453-2	Isoform 2 of Poly(A)-specific ribonuclease PARN	0.23	4.53.E-02
O95453-4	Isoform 4 of Poly(A)-specific ribonuclease PARN	0.23	4.53.E-02
O95453-3	Isoform 3 of Poly(A)-specific ribonuclease PARN	0.23	4.53.E-02
Q8TE77-3	Isoform 3 of Protein phosphatase Slingshot homolog 3	0.23	3.96.E-02
Q8TE77-2	Isoform 2 of Protein phosphatase Slingshot homolog 3	0.23	4.15.E-02
P49736	DNA replication licensing factor MCM2	0.229	4.31.E-03
Q16401	26S proteasome non-ATPase regulatory subunit 5	0.229	2.07.E-02
Q16401-2	Isoform 2 of 26S proteasome non-ATPase regulatory subunit 5	0.229	2.07.E-02
Q14978	Nucleolar and coiled-body phosphoprotein 1	0.228	3.53.E-03
Q14978-2	Isoform Beta of Nucleolar and coiled-body phosphoprotein 1	0.228	3.53.E-03
Q92777	Synapsin-2	0.228	4.36.E-02
Q92777-2	Isoform IIb of Synapsin-2	0.228	4.36.E-02
Q96RF0-2	Isoform 2 of Sorting nexin-18	0.228	4.41.E-02
Q96RF0	Sorting nexin-18	0.228	4.41.E-02
Q96RF0-3	Isoform 3 of Sorting nexin-18	0.228	4.41.E-02
Q5VV41	Rho guanine nucleotide exchange factor 16	0.227	1.04.E-02
P62380	TATA box-binding protein-like protein 1	0.227	4.84.E-02
Q5VV41-2	Isoform 2 of Rho guanine nucleotide exchange factor 16	0.227	1.42.E-02
Q8NC51	Plasminogen activator inhibitor 1 RNA-binding protein	0.226	3.89.E-03
Q3B7T1-5	Isoform 4 of Erythroid differentiation-related factor 1	0.226	2.63.E-02
Q3B7T1	Erythroid differentiation-related factor 1	0.226	2.63.E-02
Q9ULE6	Paladin	0.225	2.66.E-02
A1L390-3	Isoform 3 of Pleckstrin homology domain-containing family G member 3	0.225	3.44.E-02
A1L390	Pleckstrin homology domain-containing family G member 3	0.225	3.44.E-02
Q14195	Dihydropyrimidinase-related protein 3	0.224	3.61.E-03
P33993-3	Isoform 3 of DNA replication licensing factor MCM7	0.224	3.60.E-03
Q9NX02-4	Isoform 4 of NACHT, LRR and PYD domains-containing protein 2	0.224	3.98.E-03
P31350-2	Isoform 2 of Ribonucleoside-diphosphate reductase subunit M2	0.224	9.55.E-03
P31350	Ribonucleoside-diphosphate reductase subunit M2	0.224	9.55.E-03
Q9NX02-5	Isoform 5 of NACHT, LRR and PYD domains-containing protein 2	0.223	4.24.E-03
Q9NX02	NACHT, LRR and PYD domains-containing protein 2	0.223	4.24.E-03
Q9NX02-2	Isoform 2 of NACHT, LRR and PYD domains-containing protein 2	0.223	4.24.E-03
Q9NUQ6-2	Isoform 2 of SPATS2-like protein	0.223	1.05.E-02
Q9NUQ6	SPATS2-like protein	0.223	1.05.E-02
Q9BTT0-3	Isoform 3 of Acidic leucine-rich nuclear phosphoprotein 32 family member E	0.222	1.02.E-02
Q96AT1	Uncharacterized protein KIAA1143	0.222	2.55.E-02
P49643-2	Isoform 2 of DNA primase large subunit	0.221	3.05.E-02
Q9NX02-3	Isoform 3 of NACHT, LRR and PYD domains-containing protein 2	0.22	3.54.E-03
P53350	Serine/threonine-protein kinase PLK1	0.22	8.71.E-03
Q8WW12-2	Isoform 2 of PEST proteolytic signal-containing nuclear protein	0.219	1.24.E-02
Q8WU90-2	Isoform 2 of Zinc finger CCCH domain-containing protein 15	0.219	3.69.E-02
P33992	DNA replication licensing factor MCM5	0.218	2.96.E-03
Q9NU22	Midasin	0.218	2.78.E-03
Q86X29-2	Isoform 2 of Lipolysis-stimulated lipoprotein receptor	0.218	3.38.E-02
Q96GA3	Protein LTV1 homolog	0.217	1.15.E-02
Q8NC51-2	Isoform 2 of Plasminogen activator inhibitor 1 RNA-binding protein	0.215	2.66.E-03
O43291-2	Isoform 2 of Kunitz-type protease inhibitor 2	0.215	3.45.E-02
O43291	Kunitz-type protease inhibitor 2	0.215	3.45.E-02
P13646-2	Isoform 2 of Keratin, type I cytoskeletal 13	0.214	2.68.E-03
P63215	Guanine nucleotide-binding protein G(I)/G(S)/G(O) subunit gamma-3	0.212	2.36.E-02
Q13572-2	Isoform 2 of Inositol-tetrakisphosphate 1-kinase	0.212	8.08.E-03

Q5W0B1	ORC ubiquitin ligase 1	0.212	2.31.E-02
Q5JTD0	Tight junction-associated protein 1	0.211	1.99.E-02
Q5JTD0-2	Isoform 2 of Tight junction-associated protein 1	0.211	1.99.E-02
Q9NZN8-5	Isoform 5 of CCR4-NOT transcription complex subunit 2	0.211	2.64.E-02
Q8IVW6-4	Isoform 4 of AT-rich interactive domain-containing protein 3B	0.21	3.89.E-02
Q8IVW6	AT-rich interactive domain-containing protein 3B	0.21	3.89.E-02
Q8IVW6-3	Isoform 3 of AT-rich interactive domain-containing protein 3B	0.21	3.89.E-02
Q5UIP0-2	Isoform 2 of Telomere-associated protein RIF1	0.208	9.20.E-03
Q5UIP0	Telomere-associated protein RIF1	0.208	9.20.E-03
O95905-2	Isoform 2 of Protein ecdysoneless homolog	0.207	9.27.E-03
O15234	Protein CASC3	0.207	3.84.E-02
O15446-2	Isoform 2 of DNA-directed RNA polymerase I subunit RPA34	0.207	2.35.E-02
O15446	DNA-directed RNA polymerase I subunit RPA34	0.207	2.35.E-02
P25398	40S ribosomal protein S12	0.206	1.88.E-03
O95249	Golgi SNAP receptor complex member 1	0.206	4.00.E-02
Q9H9Z2	Protein lin-28 homolog A	0.205	1.77.E-03
P51693-2	Isoform 2 of Amyloid-like protein 1	0.205	4.32.E-02
P51693	Amyloid-like protein 1	0.205	4.32.E-02
Q96EB6-2	Isoform 2 of NAD-dependent protein deacetylase sirtuin-1	0.204	6.88.E-03
P20337	Ras-related protein Rab-3B	0.203	6.17.E-03
Q9Y2S6	Translation machinery-associated protein 7	0.203	7.07.E-03
P60174-1	Isoform 2 of Triosephosphate isomerase	0.202	1.60.E-03
P60174	Triosephosphate isomerase	0.202	1.60.E-03
P56537-2	Isoform 2 of Eukaryotic translation initiation factor 6	0.202	2.18.E-03
Q01970-2	Isoform 2 of 1-phosphatidylinositol 4,5-bisphosphate phosphodiesterase beta-3	0.201	1.06.E-02
Q01970	1-phosphatidylinositol 4,5-bisphosphate phosphodiesterase beta-3	0.201	8.51.E-03
Q9H910-3	Isoform 3 of Jupiter microtubule associated homolog 2	0.201	2.24.E-03
Q9H910-2	Isoform 2 of Jupiter microtubule associated homolog 2	0.201	2.13.E-03
Q9H910	Jupiter microtubule associated homolog 2	0.201	2.24.E-03
Q8TDP1	Ribonuclease H2 subunit C	0.201	4.26.E-02
P18065	Insulin-like growth factor-binding protein 2	0.2	4.67.E-02
Q59GN2	Putative 60S ribosomal protein L39-like 5	0.199	1.18.E-03
P62891	60S ribosomal protein L39	0.199	1.18.E-03
Q13685	Angio-associated migratory cell protein	0.199	5.00.E-03
Q9NUQ3-2	Isoform 2 of Gamma-taxilin	0.199	3.79.E-02
Q08431-2	Isoform 2 of Lactadherin	0.198	1.76.E-03
Q8TDZ2-3	Isoform 3 of [F-actin]-monooxygenase MICAL1	0.198	2.97.E-02
O43683-3	Isoform 3 of Mitotic checkpoint serine/threonine-protein kinase BUB1	0.198	2.84.E-02
O43683	Mitotic checkpoint serine/threonine-protein kinase BUB1	0.198	2.84.E-02
O43683-2	Isoform 2 of Mitotic checkpoint serine/threonine-protein kinase BUB1	0.198	2.84.E-02
Q5T6F2-2	Isoform 2 of Ubiquitin-associated protein 2	0.197	1.84.E-02
Q96RS0	Trimethylguanosine synthase	0.197	2.33.E-02
Q8TE73	Dynein heavy chain 5, axonemal	0.196	2.81.E-02
O43768-8	Isoform 8 of Alpha-endosulfine	0.195	2.83.E-02
P61916-2	Isoform 2 of NPC intracellular cholesterol transporter 2	0.194	1.61.E-03
O14530-2	Isoform 2 of Thioredoxin domain-containing protein 9	0.194	1.82.E-02
O14530	Thioredoxin domain-containing protein 9	0.194	1.82.E-02
P52732	Kinesin-like protein KIF11	0.193	1.54.E-03
P46013	Proliferation marker protein Ki-67	0.192	5.97.E-03
Q8TDB8-3	Isoform 3 of Solute carrier family 2, facilitated glucose transporter member 14	0.191	1.55.E-03
Q9Y4C1	Lysine-specific demethylase 3A	0.191	4.41.E-02
Q9Y5Y0-2	Isoform 2 of Feline leukemia virus subgroup C receptor-related protein 1	0.191	2.36.E-02
O00160	Unconventional myosin-If	0.191	2.94.E-02
Q8WX94	NACHT, LRR and PYD domains-containing protein 7	0.19	2.41.E-03
Q8WX94-2	Isoform 2 of NACHT, LRR and PYD domains-containing protein 7	0.19	2.41.E-03
Q8WX94-3	Isoform 3 of NACHT, LRR and PYD domains-containing protein 7	0.19	2.41.E-03
P0CG13	Chromosome transmission fidelity protein 8 homolog	0.19	3.75.E-02
Q15833-2	Isoform 2 of Syntaxin-binding protein 2	0.189	4.93.E-03
Q15833-3	Isoform 3 of Syntaxin-binding protein 2	0.189	4.93.E-03
Q15833	Syntaxin-binding protein 2	0.189	4.93.E-03
O14745	Na(+)/H(+) exchange regulatory cofactor NHE-RF1	0.189	2.54.E-03
Q8NCB2	CaM kinase-like vesicle-associated protein	0.188	5.67.E-03
Q8NCB2-2	Isoform 2 of CaM kinase-like vesicle-associated protein	0.188	5.67.E-03
Q8NCB2-3	Isoform 3 of CaM kinase-like vesicle-associated protein	0.188	5.67.E-03
P60174-4	Isoform 4 of Triosephosphate isomerase	0.187	8.08.E-04
O60566-2	Isoform 2 of Mitotic checkpoint serine/threonine-protein kinase BUB1 beta	0.187	3.58.E-03
Q9NUQ3	Gamma-taxilin	0.187	1.63.E-02
P05186-2	Isoform 2 of Alkaline phosphatase, tissue-nonspecific isozyme	0.186	8.34.E-04
Q9H8V3-2	Isoform 2 of Protein ECT2	0.186	5.14.E-03
Q9H8V3	Protein ECT2	0.186	5.14.E-03
Q9H8V3-3	Isoform 3 of Protein ECT2	0.186	5.14.E-03

Q9H8V3-4	Isoform 4 of Protein ECT2	0.186	5.14.E-03
O14602	Eukaryotic translation initiation factor 1A, Y-chromosomal	0.186	9.86.E-04
P63220	40S ribosomal protein S21	0.184	7.26.E-04
P09234	U1 small nuclear ribonucleoprotein C	0.183	1.90.E-02
Q659C4-9	Isoform 9 of La-related protein 1B	0.183	2.60.E-02
O94832	Unconventional myosin-Id	0.183	3.56.E-02
P05186-3	Isoform 3 of Alkaline phosphatase, tissue-nonspecific isozyme	0.182	5.35.E-04
P05186	Alkaline phosphatase, tissue-nonspecific isozyme	0.182	5.41.E-04
O75717	WD repeat and HMG-box DNA-binding protein 1	0.182	3.59.E-04
P10321-2	Isoform 2 of HLA class I histocompatibility antigen, C alpha chain	0.182	2.58.E-03
O75607	Nucleoplasmin-3	0.181	3.36.E-03
Q9ULX3	RNA-binding protein NOB1	0.179	4.40.E-03
Q9NVI1-4	Isoform 4 of Fanconi anemia group I protein	0.179	1.04.E-02
Q3B7T1-3	Isoform 2 of Erythroid differentiation-related factor 1	0.177	3.15.E-02
P52292	Importin subunit alpha-1	0.176	4.76.E-04
Q8NC60	Nitric oxide-associated protein 1	0.176	1.31.E-02
Q08431-4	Isoform 4 of Lactadherin	0.175	7.25.E-04
Q01658	Protein Dr1	0.175	2.69.E-03
Q13243-2	Isoform SRP40-2 of Serine/arginine-rich splicing factor 5	0.175	2.45.E-03
Q6BCY4-2	Isoform 2 of NADH-cytochrome b5 reductase 2	0.175	3.37.E-03
Q6BCY4	NADH-cytochrome b5 reductase 2	0.175	3.37.E-03
O15347	High mobility group protein B3	0.174	2.70.E-03
Q08431-3	Isoform 3 of Lactadherin	0.173	7.02.E-04
Q0VF96-2	Isoform 2 of Cingulin-like protein 1	0.173	1.18.E-02
Q13765	Nascent polypeptide-associated complex subunit alpha	0.172	5.26.E-04
E9PAV3	Nascent polypeptide-associated complex subunit alpha, muscle-specific form	0.172	5.26.E-04
E9PAV3-2	Isoform skNAC-2 of Nascent polypeptide-associated complex subunit alpha, muscle-specific form	0.172	5.26.E-04
P29762	Cellular retinoic acid-binding protein 1	0.171	3.85.E-04
Q08431	Lactadherin	0.171	4.42.E-04
Q96EB6	NAD-dependent protein deacetylase sirtuin-1	0.171	6.68.E-04
O75717-2	Isoform 2 of WD repeat and HMG-box DNA-binding protein 1	0.171	4.28.E-04
Q99541	Perilipin-2	0.17	8.93.E-03
O14578	Citron Rho-interacting kinase	0.169	1.54.E-02
O14578-3	Isoform 3 of Citron Rho-interacting kinase	0.169	1.54.E-02
O14578-4	Isoform 4 of Citron Rho-interacting kinase	0.169	1.54.E-02
Q13283-2	Isoform 2 of Ras GTPase-activating protein-binding protein 1	0.167	3.46.E-04
O00592	Podocalyxin	0.167	3.04.E-04
O00592-2	Isoform 2 of Podocalyxin	0.167	3.04.E-04
Q9Y3Z3-3	Isoform 3 of Deoxynucleoside triphosphate triphosphohydrolase SAMHD1	0.166	1.37.E-03
Q8IUD2-5	Isoform 5 of ELKS/Rab6-interacting/CAST family member 1	0.166	8.33.E-03
O14745-2	Isoform 2 of Na(+)/H(+) exchange regulatory cofactor NHE-RF1	0.164	2.55.E-03
Q2Q1W2	E3 ubiquitin-protein ligase TRIM71	0.162	2.00.E-04
P52435	DNA-directed RNA polymerase II subunit RPB11-a	0.161	3.39.E-03
Q5JUX0	Spindlin-3	0.159	8.52.E-03
Q0VF96	Cingulin-like protein 1	0.159	3.18.E-03
P48594	Serpin B4	0.157	9.92.E-03
P07910	Heterogeneous nuclear ribonucleoproteins C1/C2	0.157	6.59.E-03
P29508-2	Isoform 2 of Serpin B3	0.157	1.01.E-02
P29508	Serpin B3	0.157	9.92.E-03
P00966	Argininosuccinate synthase	0.156	2.45.E-04
Q5T6F2	Ubiquitin-associated protein 2	0.156	1.64.E-03
Q3B7T1-4	Isoform 3 of Erythroid differentiation-related factor 1	0.156	3.58.E-02
O94808	Glutamine--fructose-6-phosphate aminotransferase [isomerizing] 2	0.154	1.39.E-03
P06454-2	Isoform 2 of Prothymosin alpha	0.151	1.05.E-04
P06454	Prothymosin alpha	0.151	1.05.E-04
Q8IWV8-2	Isoform 2 of E3 ubiquitin-protein ligase UBR2	0.151	4.73.E-02
Q99808-2	Isoform 2 of Equilibrative nucleoside transporter 1	0.149	2.14.E-03
Q99808	Equilibrative nucleoside transporter 1	0.149	2.14.E-03
Q9BWW4	Single-stranded DNA-binding protein 3	0.148	5.73.E-03
Q9BWW4-2	Isoform 2 of Single-stranded DNA-binding protein 3	0.148	5.73.E-03
Q9BWW4-3	Isoform 3 of Single-stranded DNA-binding protein 3	0.148	5.73.E-03
O95999	B-cell lymphoma/leukemia 10	0.148	1.69.E-02
P51153	Ras-related protein Rab-13	0.146	3.28.E-03
O14757	Serine/threonine-protein kinase Chk1	0.145	1.14.E-03
O14757-2	Isoform 2 of Serine/threonine-protein kinase Chk1	0.145	1.14.E-03
O14757-3	Isoform 3 of Serine/threonine-protein kinase Chk1	0.145	1.60.E-03
O43768-2	Isoform 2 of Alpha-endosulfine	0.143	8.11.E-04
O43768-9	Isoform 9 of Alpha-endosulfine	0.143	8.11.E-04
Q2NKX8	DNA excision repair protein ERCC-6-like	0.143	3.82.E-03
O43768	Alpha-endosulfine	0.143	8.11.E-04
O43768-3	Isoform 3 of Alpha-endosulfine	0.143	8.11.E-04

O43768-4	Isoform 4 of Alpha-endosulfine	0.143	8.11.E-04
A9QM74	Importin subunit alpha-8	0.142	7.69.E-03
O43278-2	Isoform 2 of Kunitz-type protease inhibitor 1	0.142	1.40.E-03
O43278	Kunitz-type protease inhibitor 1	0.142	1.40.E-03
Q16763	Ubiquitin-conjugating enzyme E2 S	0.14	5.22.E-03
Q9Y672	Dolichyl pyrophosphate Man9GlcNAc2 alpha-1,3-glucosyltransferase	0.14	5.89.E-03
Q9Y6A5	Transforming acidic coiled-coil-containing protein 3	0.139	2.00.E-04
Q96F85	CB1 cannabinoid receptor-interacting protein 1	0.138	2.41.E-03
Q96F85-2	Isoform 2 of CB1 cannabinoid receptor-interacting protein 1	0.138	2.41.E-03
Q9C0H6	Kelch-like protein 4	0.136	6.54.E-03
Q9C0H6-2	Isoform 2 of Kelch-like protein 4	0.136	6.54.E-03
Q9HD42-2	Isoform 2 of Charged multivesicular body protein 1a	0.135	5.12.E-03
P10321	HLA class I histocompatibility antigen, C alpha chain	0.135	1.16.E-04
P62854	40S ribosomal protein S26	0.134	3.34.E-05
Q9BX66-12	Isoform 12 of Sorbin and SH3 domain-containing protein 1	0.134	8.71.E-03
Q9BX66-4	Isoform 4 of Sorbin and SH3 domain-containing protein 1	0.134	8.24.E-03
Q9BX66-8	Isoform 8 of Sorbin and SH3 domain-containing protein 1	0.134	8.24.E-03
Q9BX66-5	Isoform 5 of Sorbin and SH3 domain-containing protein 1	0.134	8.24.E-03
Q9BX66-6	Isoform 6 of Sorbin and SH3 domain-containing protein 1	0.134	8.24.E-03
Q9BX66-9	Isoform 9 of Sorbin and SH3 domain-containing protein 1	0.134	8.24.E-03
Q9BX66-10	Isoform 10 of Sorbin and SH3 domain-containing protein 1	0.134	8.24.E-03
P54289-3	Isoform 3 of Voltage-dependent calcium channel subunit alpha-2/delta-1	0.132	4.14.E-03
P54289	Voltage-dependent calcium channel subunit alpha-2/delta-1	0.132	4.14.E-03
P54289-4	Isoform 4 of Voltage-dependent calcium channel subunit alpha-2/delta-1	0.132	4.14.E-03
P54289-5	Isoform 5 of Voltage-dependent calcium channel subunit alpha-2/delta-1	0.132	4.14.E-03
P54289-2	Isoform 2 of Voltage-dependent calcium channel subunit alpha-2/delta-1	0.132	4.14.E-03
Q9H2D6	TRIO and F-actin-binding protein	0.132	2.60.E-02
Q9H2D6-4	Isoform 5 of TRIO and F-actin-binding protein	0.132	2.60.E-02
Q9H2D6-3	Isoform 4 of TRIO and F-actin-binding protein	0.132	2.60.E-02
Q9H2D6-2	Isoform 3 of TRIO and F-actin-binding protein	0.132	2.60.E-02
Q96G01	Protein bicaudal D homolog 1	0.129	4.07.E-03
Q96G01-4	Isoform 4 of Protein bicaudal D homolog 1	0.129	4.07.E-03
Q96G01-3	Isoform 3 of Protein bicaudal D homolog 1	0.129	4.07.E-03
P84101-2	Isoform 2 of Small EDRK-rich factor 2	0.127	4.64.E-03
P84101	Small EDRK-rich factor 2	0.127	4.64.E-03
P84101-4	Isoform 4 of Small EDRK-rich factor 2	0.127	4.64.E-03
P84101-3	Isoform 3 of Small EDRK-rich factor 2	0.127	4.64.E-03
P33316	Deoxyuridine 5'-triphosphate nucleotidohydrolase, mitochondrial	0.126	1.75.E-05
P33981	Dual specificity protein kinase TTK	0.126	2.38.E-03
P33981-2	Isoform 2 of Dual specificity protein kinase TTK	0.126	2.38.E-03
P00338-4	Isoform 4 of L-lactate dehydrogenase A chain	0.125	1.59.E-05
Q8IVT5-3	Isoform 3 of Kinase suppressor of Ras 1	0.125	3.80.E-03
Q8IVT5-4	Isoform 4 of Kinase suppressor of Ras 1	0.125	3.80.E-03
Q96QD8-2	Isoform 2 of Sodium-coupled neutral amino acid transporter 2	0.125	3.24.E-03
O43768-6	Isoform 6 of Alpha-endosulfine	0.125	3.03.E-04
O43768-7	Isoform 7 of Alpha-endosulfine	0.125	3.03.E-04
O43768-5	Isoform 5 of Alpha-endosulfine	0.125	3.03.E-04
O95249-2	Isoform 2 of Golgi SNAP receptor complex member 1	0.124	3.25.E-02
P50579-3	Isoform 3 of Methionine aminopeptidase 2	0.123	2.98.E-04
P50579	Methionine aminopeptidase 2	0.123	2.68.E-04
Q9UDR5	Alpha-aminoadipic semialdehyde synthase, mitochondrial	0.121	1.17.E-05
O76074-2	Isoform PDE5A2 of cGMP-specific 3',5'-cyclic phosphodiesterase	0.121	1.66.E-03
O76074	cGMP-specific 3',5'-cyclic phosphodiesterase	0.121	1.66.E-03
O95379-4	Isoform 4 of Tumor necrosis factor alpha-induced protein 8	0.121	1.04.E-02
O95379-3	Isoform 3 of Tumor necrosis factor alpha-induced protein 8	0.121	1.04.E-02
Q9BVC3	Sister chromatid cohesion protein DCC1	0.121	4.59.E-03
O95379-2	Isoform 2 of Tumor necrosis factor alpha-induced protein 8	0.121	1.04.E-02
O95379	Tumor necrosis factor alpha-induced protein 8	0.121	1.04.E-02
Q9H4H8	Protein FAM83D	0.121	7.63.E-03
Q9H4H8-2	Isoform 2 of Protein FAM83D	0.121	7.63.E-03
P00338	L-lactate dehydrogenase A chain	0.119	9.18.E-06
P00338-3	Isoform 3 of L-lactate dehydrogenase A chain	0.119	9.18.E-06
P00338-2	Isoform 2 of L-lactate dehydrogenase A chain	0.119	9.35.E-06
P00338-5	Isoform 5 of L-lactate dehydrogenase A chain	0.119	9.35.E-06
Q969L2	Protein MAL2	0.117	2.33.E-03
Q9BZK3	Putative nascent polypeptide-associated complex subunit alpha-like protein	0.116	2.39.E-05
P17693-4	Isoform 4 of HLA class I histocompatibility antigen, alpha chain G	0.115	2.35.E-03
P81605	Dermcidin	0.111	1.92.E-04
P81605-2	Isoform 2 of Dermcidin	0.111	1.92.E-04
P80723	Brain acid soluble protein 1	0.11	1.39.E-04
Q9ULZ3	Apoptosis-associated speck-like protein containing a CARD	0.109	2.58.E-04

Q9ULZ3-3	Isoform 3 of Apoptosis-associated speck-like protein containing a CARD	0.109	1.34.E-04
Q9ULZ3-2	Isoform 2 of Apoptosis-associated speck-like protein containing a CARD	0.109	2.58.E-04
Q96GD4-2	Isoform 2 of Aurora kinase B	0.107	1.60.E-03
Q96GD4-3	Isoform 3 of Aurora kinase B	0.107	2.60.E-03
Q96GD4-5	Isoform 5 of Aurora kinase B	0.107	1.60.E-03
Q96GD4-4	Isoform 4 of Aurora kinase B	0.107	1.60.E-03
Q96GD4	Aurora kinase B	0.107	1.60.E-03
P84243	Histone H3.3	0.104	4.22.E-03
P80723-2	Isoform 2 of Brain acid soluble protein 1	0.102	7.14.E-04
P41567	Eukaryotic translation initiation factor 1	0.101	1.24.E-03
Q92833-3	Isoform 3 of Protein Jumonji	0.101	1.90.E-03
Q92833	Protein Jumonji	0.101	1.90.E-03
P07951-3	Isoform 3 of Tropomyosin beta chain	0.099	3.43.E-04
Q6ZRY4	RNA-binding protein with multiple splicing 2	0.098	7.75.E-05
Q9Y5K6	CD2-associated protein	0.096	9.03.E-05
Q92485	Acid sphingomyelinase-like phosphodiesterase 3b	0.095	3.16.E-05
P56270	Myc-associated zinc finger protein	0.094	4.70.E-03
P56270-3	Isoform 3 of Myc-associated zinc finger protein	0.094	4.70.E-03
P56270-4	Isoform 4 of Myc-associated zinc finger protein	0.094	4.70.E-03
Q2KHR3	Glutamine and serine-rich protein 1	0.094	2.66.E-03
P56270-2	Isoform 2 of Myc-associated zinc finger protein	0.094	4.70.E-03
Q6DKI1	60S ribosomal protein L7-like 1	0.093	3.03.E-03
Q9Y5J1	U3 small nucleolar RNA-associated protein 18 homolog	0.093	9.33.E-03
P50453	Serpin B9	0.092	4.29.E-07
P12532-2	Isoform 2 of Creatine kinase U-type, mitochondrial	0.092	2.79.E-06
Q93062-2	Isoform B of RNA-binding protein with multiple splicing	0.09	1.48.E-06
Q93062-4	Isoform D of RNA-binding protein with multiple splicing	0.09	1.48.E-06
Q93062-5	Isoform E of RNA-binding protein with multiple splicing	0.09	1.48.E-06
Q93062	RNA-binding protein with multiple splicing	0.09	1.48.E-06
Q93062-3	Isoform C of RNA-binding protein with multiple splicing	0.09	1.31.E-06
Q9Y6Q5	AP-1 complex subunit mu-2	0.089	2.62.E-05
Q9Y6Q5-2	Isoform 2 of AP-1 complex subunit mu-2	0.089	5.53.E-05
P04183	Thymidine kinase, cytosolic	0.089	2.29.E-03
Q5JNZ5	Putative 40S ribosomal protein S26-like 1	0.088	2.56.E-07
Q8WY07	Cationic amino acid transporter 3	0.086	2.38.E-05
P50579-2	Isoform 2 of Methionine aminopeptidase 2	0.086	1.57.E-02
P12532	Creatine kinase U-type, mitochondrial	0.084	6.47.E-07
Q9H0R3	Transmembrane protein 222	0.083	5.09.E-04
P30740-2	Isoform 2 of Leukocyte elastase inhibitor	0.082	7.28.E-06
O75830	Serpin I2	0.082	7.28.E-06
P30740	Leukocyte elastase inhibitor	0.082	7.28.E-06
P55327-7	Isoform 7 of Tumor protein D52	0.081	8.31.E-06
P55327-4	Isoform 4 of Tumor protein D52	0.081	8.31.E-06
P55327-3	Isoform 3 of Tumor protein D52	0.081	8.31.E-06
P55327-5	Isoform 5 of Tumor protein D52	0.081	8.31.E-06
P55327-2	Isoform 2 of Tumor protein D52	0.081	8.31.E-06
P55327-6	Isoform 6 of Tumor protein D52	0.081	8.31.E-06
P55327	Tumor protein D52	0.081	8.31.E-06
P17540	Creatine kinase S-type, mitochondrial	0.079	8.52.E-06
Q16851	UTP--glucose-1-phosphate uridylyltransferase	0.076	3.86.E-08
P21926	CD9 antigen	0.074	2.45.E-04
Q01973	Inactive tyrosine-protein kinase transmembrane receptor ROR1	0.07	2.55.E-06
O95070	Protein YIF1A	0.07	1.60.E-04
Q01973-2	Isoform Short of Inactive tyrosine-protein kinase transmembrane receptor ROR1	0.07	1.86.E-04
Q6P5R6	60S ribosomal protein L22-like 1	0.065	3.75.E-05
Q8TDC3-3	Isoform 3 of Serine/threonine-protein kinase BRSK1	0.063	5.18.E-04
P10606	Cytochrome c oxidase subunit 5B, mitochondrial	0.059	8.06.E-07
Q9BYZ2	L-lactate dehydrogenase A-like 6B	0.058	9.42.E-07
Q96SQ9-2	Isoform 2 of Cytochrome P450 2S1	0.057	5.78.E-10
Q96SQ9	Cytochrome P450 2S1	0.057	5.78.E-10
Q5K4L6-2	Isoform 2 of Solute carrier family 27 member 3	0.054	1.11.E-04
Q5K4L6	Solute carrier family 27 member 3	0.054	1.11.E-04
Q5T7N2	LINE-1 type transposase domain-containing protein 1	0.052	1.50.E-10
Q5VZK9-2	Isoform 2 of F-actin-uncapping protein LRRC16A	0.046	2.05.E-03
Q5VZK9	F-actin-uncapping protein LRRC16A	0.046	2.05.E-03
O43272-2	Isoform 2 of Proline dehydrogenase 1, mitochondrial	0.043	6.99.E-06
O43272	Proline dehydrogenase 1, mitochondrial	0.043	7.29.E-07
O43272-1	Isoform 3 of Proline dehydrogenase 1, mitochondrial	0.043	6.99.E-06
P12830-2	Isoform 2 of Cadherin-1	0.035	1.10.E-11
P50452-3	Isoform 3 of Serpin B8	0.029	2.29.E-09
Q86VV8	Rotatin	0.024	1.02.E-14

Q86VV8-4	Isoform 4 of Rotatin	0.024	1.02.E-14
Q86VV8-3	Isoform 3 of Rotatin	0.024	1.02.E-14
Q86VV8-2	Isoform 2 of Rotatin	0.024	1.02.E-14
P08247	Synaptophysin	0.01	1.00.E-17
P08247-2	Isoform 2 of Synaptophysin	0.01	1.00.E-17
Q16623-2	Isoform 2 of Syntaxin-1A	0.01	1.00.E-17
Q16623	Syntaxin-1A	0.01	1.00.E-17
Q16623-3	Isoform 3 of Syntaxin-1A	0.01	1.00.E-17
Q96MZ0-2	Isoform 2 of Ganglioside-induced differentiation-associated protein 1-like 1	0.01	1.00.E-17
O75061-3	Isoform 3 of Putative tyrosine-protein phosphatase auxilin	0.01	1.00.E-17
O75886	Signal transducing adapter molecule 2	0.01	1.00.E-17
O15075	Serine/threonine-protein kinase DCLK1	0.01	1.00.E-17
O15075-4	Isoform 4 of Serine/threonine-protein kinase DCLK1	0.01	1.00.E-17
Q9UL26	Ras-related protein Rab-22A	0.01	1.00.E-17
Q99574	Neuroserpin	0.01	1.00.E-17
O75911	Short-chain dehydrogenase/reductase 3	0.01	1.00.E-17
Q96FE5	Leucine-rich repeat and immunoglobulin-like domain-containing nogo receptor-interacting protein 1	0.01	1.00.E-17
Q96FE5-2	Isoform 2 of Leucine-rich repeat and immunoglobulin-like domain-containing nogo receptor-interacting protein 1	0.01	1.00.E-17
P78352	Disks large homolog 4	0.01	1.00.E-17
P78352-3	Isoform 3 of Disks large homolog 4	0.01	1.00.E-17
P78352-2	Isoform 2 of Disks large homolog 4	0.01	1.00.E-17
Q96P47-6	Isoform 6 of Arf-GAP with GTPase, ANK repeat and PH domain-containing protein 3	0.01	1.00.E-17
Q96P47-3	Isoform 3 of Arf-GAP with GTPase, ANK repeat and PH domain-containing protein 3	0.01	1.00.E-17
Q06481-5	Isoform 5 of Amyloid-like protein 2	0.01	1.00.E-17
P25686-2	Isoform 2 of DnaJ homolog subfamily B member 2	0.01	1.00.E-17
P25686	DnaJ homolog subfamily B member 2	0.01	1.00.E-17
Q8N4V2	Synaptic vesicle 2-related protein	0.01	1.00.E-17
Q7Z2W4-4	Isoform 4 of Zinc finger CCCH-type antiviral protein 1	0.01	1.00.E-17
Q7Z2W4-5	Isoform 5 of Zinc finger CCCH-type antiviral protein 1	0.01	1.00.E-17
P52790	Hexokinase-3	0.01	1.00.E-17
Q9NZR1	Tropomodulin-2	0.01	1.00.E-17
Q9NZR1-2	Isoform 2 of Tropomodulin-2	0.01	1.00.E-17
Q14155	Rho guanine nucleotide exchange factor 7	0.01	1.00.E-17
Q14155-2	Isoform 2 of Rho guanine nucleotide exchange factor 7	0.01	1.00.E-17
Q14155-3	Isoform 3 of Rho guanine nucleotide exchange factor 7	0.01	1.00.E-17
O76070	Gamma-synuclein	0.01	1.00.E-17
P54277-3	Isoform 3 of PMS1 protein homolog 1	0.01	1.00.E-17
P54277	PMS1 protein homolog 1	0.01	1.00.E-17
P54277-4	Isoform 4 of PMS1 protein homolog 1	0.01	1.00.E-17
P54277-2	Isoform 2 of PMS1 protein homolog 1	0.01	1.00.E-17
Q5TH69	Brefeldin A-inhibited guanine nucleotide-exchange protein 3	0.01	1.00.E-17
Q8TBB6	Probable cationic amino acid transporter	0.01	1.00.E-17
O60262	Guanine nucleotide-binding protein G(I)/G(S)/G(O) subunit gamma-7	0.01	1.00.E-17
Q96QD9	UAP56-interacting factor	0.01	1.00.E-17
Q96QD9-2	Isoform 2 of UAP56-interacting factor	0.01	1.00.E-17
Q96QD9-4	Isoform 4 of UAP56-interacting factor	0.01	1.00.E-17
Q6UX07	Dehydrogenase/reductase SDR family member 13	0.01	1.00.E-17
Q86YS3-2	Isoform 2 of Rab11 family-interacting protein 4	0.01	1.00.E-17
Q86YS3	Rab11 family-interacting protein 4	0.01	1.00.E-17
Q96PY5	Formin-like protein 2	0.01	1.00.E-17
O95466	Formin-like protein 1	0.01	1.00.E-17
P29218-2	Isoform 2 of Inositol monophosphatase 1	0.01	1.00.E-17
Q96RT6	cTAGE family member 2	0.01	1.00.E-17
Q9UPN9-2	Isoform Beta of E3 ubiquitin-protein ligase TRIM33	0.01	1.00.E-17
P48454-2	Isoform 2 of Serine/threonine-protein phosphatase 2B catalytic subunit gamma isoform	0.01	1.00.E-17
P48454	Serine/threonine-protein phosphatase 2B catalytic subunit gamma isoform	0.01	1.00.E-17
P48454-3	Isoform 3 of Serine/threonine-protein phosphatase 2B catalytic subunit gamma isoform	0.01	1.00.E-17
Q9P0K9	DOMON domain-containing protein FRRS1L	0.01	1.00.E-17
Q8WWC4	m-AAA protease-interacting protein 1, mitochondrial	0.01	1.00.E-17
P02656	Apolipoprotein C-III	0.01	1.00.E-17
Q9NV70	Exocyst complex component 1	0.01	1.00.E-17
Q9NV70-2	Isoform 2 of Exocyst complex component 1	0.01	1.00.E-17
P09110	3-ketoacyl-CoA thiolase, peroxisomal	0.01	1.00.E-17
Q9NZZ3	Charged multivesicular body protein 5	0.01	1.00.E-17
Q9NZZ3-2	Isoform 2 of Charged multivesicular body protein 5	0.01	1.00.E-17
Q8N6C5	Immunoglobulin superfamily member 1	0.01	1.00.E-17
Q8N6C5-2	Isoform 2 of Immunoglobulin superfamily member 1	0.01	1.00.E-17
Q8N6C5-4	Isoform 4 of Immunoglobulin superfamily member 1	0.01	1.00.E-17
Q9Y6M4-3	Isoform 3 of Casein kinase I isoform gamma-3	0.01	1.00.E-17
Q9Y6M4	Casein kinase I isoform gamma-3	0.01	1.00.E-17

Q9Y6M4-5	Isoform 5 of Casein kinase I isoform gamma-3	0.01	1.00.E-17
Q9Y6M4-2	Isoform 2 of Casein kinase I isoform gamma-3	0.01	1.00.E-17
P55327-8	Isoform 8 of Tumor protein D52	0.01	1.00.E-17
Q9P227	Rho GTPase-activating protein 23	0.01	1.00.E-17
Q9P227-2	Isoform 2 of Rho GTPase-activating protein 23	0.01	1.00.E-17
Q12840	Kinesin heavy chain isoform 5A	0.01	1.00.E-17
Q9BVQ7	Spermatogenesis-associated protein 5-like protein 1	0.01	1.00.E-17
Q5K4L6-3	Isoform 3 of Solute carrier family 27 member 3	0.01	1.00.E-17
Q15596	Nuclear receptor coactivator 2	0.01	1.00.E-17
Q9BXM9-2	Isoform 2 of FSD1-like protein	0.01	1.00.E-17
Q9BXM9-3	Isoform 3 of FSD1-like protein	0.01	1.00.E-17
Q9BXM9	FSD1-like protein	0.01	1.00.E-17
Q9Y5B6-3	Isoform 3 of PAX3- and PAX7-binding protein 1	0.01	1.00.E-17
Q9NZ52-2	Isoform Short of ADP-ribosylation factor-binding protein GGA3	0.01	1.00.E-17
Q9NZ52	ADP-ribosylation factor-binding protein GGA3	0.01	1.00.E-17
Q9NZ52-4	Isoform 4 of ADP-ribosylation factor-binding protein GGA3	0.01	1.00.E-17
Q9NZ52-3	Isoform 3 of ADP-ribosylation factor-binding protein GGA3	0.01	1.00.E-17
Q96AW1	Vesicular, overexpressed in cancer, prosurvival protein 1	0.01	1.00.E-17
Q96AW1-2	Isoform 2 of Vesicular, overexpressed in cancer, prosurvival protein 1	0.01	1.00.E-17
Q96AW1-4	Isoform 4 of Vesicular, overexpressed in cancer, prosurvival protein 1	0.01	1.00.E-17
Q96AW1-3	Isoform 3 of Vesicular, overexpressed in cancer, prosurvival protein 1	0.01	1.00.E-17
O43174	Cytochrome P450 26A1	0.01	1.00.E-17
O43174-2	Isoform 2 of Cytochrome P450 26A1	0.01	1.00.E-17
Q6ZTA4-2	Isoform 2 of Tripartite motif-containing protein 67	0.01	1.00.E-17
Q6ZTA4	Tripartite motif-containing protein 67	0.01	1.00.E-17
Q9NX76	CKLF-like MARVEL transmembrane domain-containing protein 6	0.01	1.00.E-17
O15240	Neurosecretory protein VGF	0.01	1.00.E-17
Q9BX66	Sorbin and SH3 domain-containing protein 1	0.01	1.00.E-17
Q9BX66-3	Isoform 3 of Sorbin and SH3 domain-containing protein 1	0.01	1.00.E-17
Q9BX66-2	Isoform 2 of Sorbin and SH3 domain-containing protein 1	0.01	1.00.E-17
Q9BX66-11	Isoform 11 of Sorbin and SH3 domain-containing protein 1	0.01	1.00.E-17
Q9BX66-7	Isoform 7 of Sorbin and SH3 domain-containing protein 1	0.01	1.00.E-17
Q66PJ3-5	Isoform 5 of ADP-ribosylation factor-like protein 6-interacting protein 4	0.01	1.00.E-17
O15321	Transmembrane 9 superfamily member 1	0.01	1.00.E-17
O15321-2	Isoform 2 of Transmembrane 9 superfamily member 1	0.01	1.00.E-17
O00750	Phosphatidylinositol 4-phosphate 3-kinase C2 domain-containing subunit beta	0.01	1.00.E-17
Q6IBW4	Condensin-2 complex subunit H2	0.01	1.00.E-17
Q6IBW4-2	Isoform 2 of Condensin-2 complex subunit H2	0.01	1.00.E-17
Q6IBW4-4	Isoform 4 of Condensin-2 complex subunit H2	0.01	1.00.E-17
O00308-2	Isoform 2 of NEDD4-like E3 ubiquitin-protein ligase WWP2	0.01	1.00.E-17
O00308-4	Isoform 4 of NEDD4-like E3 ubiquitin-protein ligase WWP2	0.01	1.00.E-17
O00308	NEDD4-like E3 ubiquitin-protein ligase WWP2	0.01	1.00.E-17
Q9H6T0-2	Isoform 2 of Epithelial splicing regulatory protein 2	0.01	1.00.E-17
Q9H6T0	Epithelial splicing regulatory protein 2	0.01	1.00.E-17
Q9NXC5-2	Isoform 2 of GATOR complex protein MIOS	0.01	1.00.E-17
Q08174	Protocadherin-1	0.01	1.00.E-17
Q08174-2	Isoform 2 of Protocadherin-1	0.01	1.00.E-17
Q969T7-2	Isoform 2 of 7-methylguanosine phosphate-specific 5'-nucleotidase	0.01	1.00.E-17
Q969T7	7-methylguanosine phosphate-specific 5'-nucleotidase	0.01	1.00.E-17
Q9UKA9-4	Isoform 4 of Polypyrimidine tract-binding protein 2	0.01	1.00.E-17
Q9UKA9-6	Isoform 6 of Polypyrimidine tract-binding protein 2	0.01	1.00.E-17
Q9UKA9-3	Isoform 3 of Polypyrimidine tract-binding protein 2	0.01	1.00.E-17
Q8N4S0-2	Isoform 2 of Coiled-coil domain-containing protein 82	0.01	1.00.E-17
Q8N4S0	Coiled-coil domain-containing protein 82	0.01	1.00.E-17
Q86Y13-2	Isoform 2 of E3 ubiquitin-protein ligase DZIP3	0.01	1.00.E-17
P78312	Protein FAM193A	0.01	1.00.E-17
P78312-5	Isoform 5 of Protein FAM193A	0.01	1.00.E-17
P78312-3	Isoform 3 of Protein FAM193A	0.01	1.00.E-17
P78312-6	Isoform 6 of Protein FAM193A	0.01	1.00.E-17
P78312-2	Isoform 2 of Protein FAM193A	0.01	1.00.E-17
Q96KQ7-2	Isoform 2 of Histone-lysine N-methyltransferase EHMT2	0.01	1.00.E-17
Q96KQ7	Histone-lysine N-methyltransferase EHMT2	0.01	1.00.E-17
Q96KQ7-3	Isoform 3 of Histone-lysine N-methyltransferase EHMT2	0.01	1.00.E-17
P30038-2	Isoform 2 of Delta-1-pyrroline-5-carboxylate dehydrogenase, mitochondrial	0.01	1.00.E-17
Q8N4S7	Progestin and adipoQ receptor family member 4	0.01	1.00.E-17
Q8N4S7-3	Isoform 3 of Progestin and adipoQ receptor family member 4	0.01	1.00.E-17
Q8N4S7-2	Isoform 2 of Progestin and adipoQ receptor family member 4	0.01	1.00.E-17
Q9H7Z3	Nuclear exosome regulator NRDE2	0.01	1.00.E-17
Q92485-2	Isoform 2 of Acid sphingomyelinase-like phosphodiesterase 3b	0.01	1.00.E-17
Q9GZM3-2	Isoform 2 of DNA-directed RNA polymerase II subunit RPB11-b1	0.01	1.00.E-17
O94874-3	Isoform 3 of E3 UFM1-protein ligase 1	0.01	1.00.E-17

Q9UHI6-2	Isoform 2 of Probable ATP-dependent RNA helicase DDX20	0.01	1.00.E-17
Q8N5C6	S1 RNA-binding domain-containing protein 1	0.01	1.00.E-17
Q96PV6-1	Isoform 2 of Leukocyte receptor cluster member 8	0.01	1.00.E-17
P22607-2	Isoform 2 of Fibroblast growth factor receptor 3	0.01	1.00.E-17
Q86U28	Iron-sulfur cluster assembly 2 homolog, mitochondrial	0.01	1.00.E-17
P07741-2	Isoform 2 of Adenine phosphoribosyltransferase	0.01	1.00.E-17
Q8IWA0	WD repeat-containing protein 75	0.01	1.00.E-17
O60645	Exocyst complex component 3	0.01	1.00.E-17
Q8TD55	Pleckstrin homology domain-containing family O member 2	0.01	1.00.E-17
Q13439	Golgin subfamily A member 4	0.01	1.00.E-17
Q9Y597	BTB/POZ domain-containing protein KCTD3	0.01	1.00.E-17
O60343-4	Isoform 4 of TBC1 domain family member 4	0.01	1.00.E-17
Q9BT73	Proteasome assembly chaperone 3	0.01	1.00.E-17
O95926	Pre-mRNA-splicing factor SYF2	0.01	1.00.E-17
O14965	Aurora kinase A	0.01	1.00.E-17
Q8IXQ5-4	Isoform 4 of Kelch-like protein 7	0.01	1.00.E-17
P15884-5	Isoform B-delta of Transcription factor 4	0.01	1.00.E-17
Q8NFJ9-3	Isoform 2 of Bardet-Biedl syndrome 1 protein	0.01	1.00.E-17
Q9Y3B8-2	Isoform 2 of Oligoribonuclease, mitochondrial	0.01	1.00.E-17
Q8NFJ9	Bardet-Biedl syndrome 1 protein	0.01	1.00.E-17
Q8WVC0-2	Isoform 2 of RNA polymerase-associated protein LEO1	0.01	1.00.E-17
Q8IXQ5	Kelch-like protein 7	0.01	1.00.E-17
Q8IXQ5-3	Isoform 3 of Kelch-like protein 7	0.01	1.00.E-17
Q9NQS7-2	Isoform 2 of Inner centromere protein	0.01	1.00.E-17
Q9NSI2	Protein FAM207A	0.01	1.00.E-17
P21802-23	Isoform 17 of Fibroblast growth factor receptor 2	0.01	1.00.E-17
Q9H078-3	Isoform 3 of Caseinolytic peptidase B protein homolog	0.01	1.00.E-17
P57081-3	Isoform 3 of tRNA (guanine-N(7)-)-methyltransferase non-catalytic subunit WDR4	0.01	1.00.E-17
Q6DKI1-2	Isoform 2 of 60S ribosomal protein L7-like 1	0.01	1.00.E-17
O75143-2	Isoform 2 of Autophagy-related protein 13	0.01	1.00.E-17
Q9H6D7-4	Isoform 4 of HAUS augmin-like complex subunit 4	0.01	1.00.E-17
P15884-6	Isoform A- of Transcription factor 4	0.01	1.00.E-17
O75362	Zinc finger protein 217	0.01	1.00.E-17
P21802-3	Isoform 3 of Fibroblast growth factor receptor 2	0.01	1.00.E-17
Q8IXQ5-2	Isoform 2 of Kelch-like protein 7	0.01	1.00.E-17
P20839-2	Isoform 2 of Inosine-5'-monophosphate dehydrogenase 1	0.01	1.00.E-17
O14732-2	Isoform 2 of Inositol monophosphatase 2	0.01	1.00.E-17
Q96PV7-2	Isoform 2 of Protein FAM193B	0.01	1.00.E-17
Q8IYM9-2	Isoform 2 of E3 ubiquitin-protein ligase TRIM22	0.01	1.00.E-17
Q86XN8	RNA-binding protein MEX3D	0.01	1.00.E-17
Q5VZ89-5	Isoform 5 of DENN domain-containing protein 4C	0.01	1.00.E-17
O95816-2	Isoform 2 of BAG family molecular chaperone regulator 2	0.01	1.00.E-17
Q96EL2	28S ribosomal protein S24, mitochondrial	0.01	1.00.E-17
Q9H078-5	Isoform 5 of Caseinolytic peptidase B protein homolog	0.01	1.00.E-17
P85298-5	Isoform 5 of Rho GTPase-activating protein 8	0.01	1.00.E-17
Q8IYM9	E3 ubiquitin-protein ligase TRIM22	0.01	1.00.E-17
Q14CB8-3	Isoform 3 of Rho GTPase-activating protein 19	0.01	1.00.E-17
Q9NUL5	Shiftless antiviral inhibitor of ribosomal frameshifting protein	0.01	1.00.E-17
Q9H2H8	Peptidyl-prolyl cis-trans isomerase-like 3	0.01	1.00.E-17
P45983-4	Isoform 4 of Mitogen-activated protein kinase 8	0.01	1.00.E-17
Q9UJQ4-2	Isoform SALL4B of Sal-like protein 4	0.01	1.00.E-17
P21802-2	Isoform 2 of Fibroblast growth factor receptor 2	0.01	1.00.E-17
P22607-4	Isoform 4 of Fibroblast growth factor receptor 3	0.01	1.00.E-17
P22607	Fibroblast growth factor receptor 3	0.01	1.00.E-17
Q9Y5X3-2	Isoform 2 of Sorting nexin-5	0.01	1.00.E-17
P29350-2	Isoform 3 of Tyrosine-protein phosphatase non-receptor type 6	0.01	1.00.E-17
Q14CB8	Rho GTPase-activating protein 19	0.01	1.00.E-17
Q14192-2	Isoform 2 of Four and a half LIM domains protein 2	0.01	1.00.E-17
Q8N4C6-4	Isoform 4 of Ninein	0.01	1.00.E-17
Q6YHU6-5	Isoform 5 of Thyroid adenoma-associated protein	0.01	1.00.E-17
P14902	Indoleamine 2,3-dioxygenase 1	0.01	1.00.E-17
P49848	Transcription initiation factor TFIID subunit 6	0.01	1.00.E-17
Q96SZ4-3	Isoform 3 of Zinc finger and SCAN domain-containing protein 10	0.01	1.00.E-17
Q8N5I9	Uncharacterized protein C12orf45	0.01	1.00.E-17
P29350-4	Isoform 4 of Tyrosine-protein phosphatase non-receptor type 6	0.01	1.00.E-17
A5YM69	Rho guanine nucleotide exchange factor 35	0.01	1.00.E-17
Q9UMX1-2	Isoform 2 of Suppressor of fused homolog	0.01	1.00.E-17
O15287	Fanconi anemia group G protein	0.01	1.00.E-17
Q6EEV4	DNA-directed RNA polymerase II subunit GRINL1A, isoforms 4/5	0.01	1.00.E-17
P49848-4	Isoform 4 of Transcription initiation factor TFIID subunit 6	0.01	1.00.E-17
P15884-9	Isoform D- of Transcription factor 4	0.01	1.00.E-17

P98173-3	Isoform 3 of Protein FAM3A	0.01	1.00.E-17
Q9Y3B8-3	Isoform 3 of Oligoribonuclease, mitochondrial	0.01	1.00.E-17
Q14CB8-7	Isoform 7 of Rho GTPase-activating protein 19	0.01	1.00.E-17
O75143-3	Isoform 3 of Autophagy-related protein 13	0.01	1.00.E-17
Q9NVQ4	Fas apoptotic inhibitory molecule 1	0.01	1.00.E-17
P52198	Rho-related GTP-binding protein RhoN	0.01	1.00.E-17
Q9NRR3	CDC42 small effector protein 2	0.01	1.00.E-17
Q9UIU6	Homeobox protein SIX4	0.01	1.00.E-17
Q8N5C6-2	Isoform 2 of S1 RNA-binding domain-containing protein 1	0.01	1.00.E-17
Q16875-2	Isoform 2 of 6-phosphofructo-2-kinase/fructose-2,6-bisphosphatase 3	0.01	1.00.E-17
Q8NFU3	Thiosulfate:glutathione sulfurtransferase	0.01	1.00.E-17
Q9NSP4-3	Isoform 3 of Centromere protein M	0.01	1.00.E-17
Q15751	Probable E3 ubiquitin-protein ligase HERC1	0.01	1.00.E-17
Q8NFU3-3	Isoform 3 of Thiosulfate:glutathione sulfurtransferase	0.01	1.00.E-17
Q9UBN4-6	Isoform Zeta of Short transient receptor potential channel 4	0.01	1.00.E-17
Q86XZ4	Spermatogenesis-associated serine-rich protein 2	0.01	1.00.E-17
Q14451-4	Isoform 4 of Growth factor receptor-bound protein 7	0.01	1.00.E-17
P49848-3	Isoform 3 of Transcription initiation factor TFIID subunit 6	0.01	1.00.E-17
P85298-4	Isoform 4 of Rho GTPase-activating protein 8	0.01	1.00.E-17
Q9NPA3	Mid1-interacting protein 1	0.01	1.00.E-17
Q9BXS6-6	Isoform 6 of Nucleolar and spindle-associated protein 1	0.01	1.00.E-17
Q06416	Putative POU domain, class 5, transcription factor 1B	0.01	1.00.E-17
P41134	DNA-binding protein inhibitor ID-1	0.01	1.00.E-17
Q96CP6-3	Isoform 3 of Protein Aster-A	0.01	1.00.E-17
P20839-3	Isoform 3 of Inosine-5'-monophosphate dehydrogenase 1	0.01	1.00.E-17
Q6EEV4-2	Isoform 5 of DNA-directed RNA polymerase II subunit GRINL1A, isoforms 4/5	0.01	1.00.E-17
Q01860	POU domain, class 5, transcription factor 1	0.01	1.00.E-17
Q96PV6	Leukocyte receptor cluster member 8	0.01	1.00.E-17
P04040	Catalase	0.01	1.00.E-17
Q14244-7	Isoform 7 of Ensconsin	0.01	1.00.E-17
Q9BXS6	Nucleolar and spindle-associated protein 1	0.01	1.00.E-17
P20839-5	Isoform 5 of Inosine-5'-monophosphate dehydrogenase 1	0.01	1.00.E-17
Q96PV7-3	Isoform 3 of Protein FAM193B	0.01	1.00.E-17
Q9NVM4-4	Isoform 4 of Protein arginine N-methyltransferase 7	0.01	1.00.E-17
Q9UBR2	Cathepsin Z	0.01	1.00.E-17
P21709-2	Isoform 2 of Ephrin type-A receptor 1	0.01	1.00.E-17
Q9Y2X9-2	Isoform 2 of Zinc finger protein 281	0.01	1.00.E-17
Q71DI3	Histone H3.2	0.01	1.00.E-17
Q9NRG0	Chromatin accessibility complex protein 1	0.01	1.00.E-17
Q92833-2	Isoform 2 of Protein Jumonji	0.01	1.00.E-17
Q14CB8-6	Isoform 6 of Rho GTPase-activating protein 19	0.01	1.00.E-17
Q14244-3	Isoform 3 of Ensconsin	0.01	1.00.E-17
P21802-17	Isoform 11 of Fibroblast growth factor receptor 2	0.01	1.00.E-17
Q05513-3	Isoform 3 of Protein kinase C zeta type	0.01	1.00.E-17
Q8N4C6-7	Isoform 7 of Ninein	0.01	1.00.E-17
Q8NFU3-2	Isoform 2 of Thiosulfate:glutathione sulfurtransferase	0.01	1.00.E-17
Q5VZ89-3	Isoform 3 of DENN domain-containing protein 4C	0.01	1.00.E-17
Q8N4C6-11	Isoform 9 of Ninein	0.01	1.00.E-17
Q9H6D7-2	Isoform 2 of HAUS augmin-like complex subunit 4	0.01	1.00.E-17
O15155-2	Isoform 2 of BET1 homolog	0.01	1.00.E-17
Q5T5X7	BEN domain-containing protein 3	0.01	1.00.E-17
Q7L190	Developmental pluripotency-associated protein 4	0.01	1.00.E-17
Q9UBU8	Mortality factor 4-like protein 1	0.01	1.00.E-17
Q8N4C6	Ninein	0.01	1.00.E-17
P15884-8	Isoform H- of Transcription factor 4	0.01	1.00.E-17
Q9UKK9	ADP-sugar pyrophosphatase	0.01	1.00.E-17
Q9UBU8-2	Isoform 2 of Mortality factor 4-like protein 1	0.01	1.00.E-17
Q9BZX2-2	Isoform 2 of Uridine-cytidine kinase 2	0.01	1.00.E-17
Q96SZ4	Zinc finger and SCAN domain-containing protein 10	0.01	1.00.E-17
Q9NQS7	Inner centromere protein	0.01	1.00.E-17
Q8NFU3-4	Isoform 4 of Thiosulfate:glutathione sulfurtransferase	0.01	1.00.E-17
P21802-16	Isoform 10 of Fibroblast growth factor receptor 2	0.01	1.00.E-17
Q9NRG1-2	Isoform 2 of Phosphoribosyltransferase domain-containing protein 1	0.01	1.00.E-17
Q969K3	E3 ubiquitin-protein ligase RNF34	0.01	1.00.E-17
Q6YHU6-3	Isoform 3 of Thyroid adenoma-associated protein	0.01	1.00.E-17
Q6IQ49	Replication stress response regulator SDE2	0.01	1.00.E-17
P21802-18	Isoform 12 of Fibroblast growth factor receptor 2	0.01	1.00.E-17
Q6IQ49-3	Isoform 3 of Replication stress response regulator SDE2	0.01	1.00.E-17
P15884-7	Isoform G- of Transcription factor 4	0.01	1.00.E-17
P15884-4	Isoform B+delta of Transcription factor 4	0.01	1.00.E-17
O00308-3	Isoform 3 of NEDD4-like E3 ubiquitin-protein ligase WWP2	0.01	1.00.E-17

Q13309-4	Isoform 3 of S-phase kinase-associated protein 2	0.01	1.00.E-17
Q16875-4	Isoform 4 of 6-phosphofructo-2-kinase/fructose-2,6-bisphosphatase 3	0.01	1.00.E-17
P85298-2	Isoform 2 of Rho GTPase-activating protein 8	0.01	1.00.E-17
Q9NQW6	Anillin	0.01	1.00.E-17
P07741	Adenine phosphoribosyltransferase	0.01	1.00.E-17
Q96PV7	Protein FAM193B	0.01	1.00.E-17
Q8N4C6-2	Isoform 2 of Ninein	0.01	1.00.E-17
Q14137-2	Isoform 2 of Ribosome biogenesis protein BOP1	0.01	1.00.E-17
P15884-2	Isoform SEF2-1A of Transcription factor 4	0.01	1.00.E-17
Q9H078-4	Isoform 4 of Caseinolytic peptidase B protein homolog	0.01	1.00.E-17
P04233	HLA class II histocompatibility antigen gamma chain	0.01	1.00.E-17
P21709	Ephrin type-A receptor 1	0.01	1.00.E-17
O00311	Cell division cycle 7-related protein kinase	0.01	1.00.E-17
Q8N4C6-9	Isoform 8 of Ninein	0.01	1.00.E-17
Q8NFJ9-2	Isoform 3 of Bardet-Biedl syndrome 1 protein	0.01	1.00.E-17
O75143-4	Isoform 4 of Autophagy-related protein 13	0.01	1.00.E-17
Q9H078	Caseinolytic peptidase B protein homolog	0.01	1.00.E-17
Q15652-3	Isoform 3 of Probable JmjC domain-containing histone demethylation protein 2C	0.01	1.00.E-17
Q8NI60	Atypical kinase COQ8A, mitochondrial	0.01	1.00.E-17
Q9BXS6-4	Isoform 4 of Nucleolar and spindle-associated protein 1	0.01	1.00.E-17
Q99417	c-Myc-binding protein	0.01	1.00.E-17
Q9P219	Protein Daple	0.01	1.00.E-17
Q9H2S1	Small conductance calcium-activated potassium channel protein 2	0.01	1.00.E-17
P57081-2	Isoform 2 of tRNA (guanine-N(7)-)-methyltransferase non-catalytic subunit WDR4	0.01	1.00.E-17
P04233-3	Isoform 3 of HLA class II histocompatibility antigen gamma chain	0.01	1.00.E-17
P21802	Fibroblast growth factor receptor 2	0.01	1.00.E-17
Q01081-2	Isoform 2 of Splicing factor U2AF 35 kDa subunit	0.01	1.00.E-17
P21802-6	Isoform 6 of Fibroblast growth factor receptor 2	0.01	1.00.E-17
O75143-5	Isoform 5 of Autophagy-related protein 13	0.01	1.00.E-17
Q9NX24	H/ACA ribonucleoprotein complex subunit 2	0.01	1.00.E-17
Q5U5Q3	RNA-binding E3 ubiquitin-protein ligase MEX3C	0.01	1.00.E-17
Q9H078-2	Isoform 2 of Caseinolytic peptidase B protein homolog	0.01	1.00.E-17
Q6N069-5	Isoform 5 of N-alpha-acetyltransferase 16, NatA auxiliary subunit	0.01	1.00.E-17
Q9BXS6-5	Isoform 5 of Nucleolar and spindle-associated protein 1	0.01	1.00.E-17
O43818	U3 small nucleolar RNA-interacting protein 2	0.01	1.00.E-17
Q969K3-2	Isoform 2 of E3 ubiquitin-protein ligase RNF34	0.01	1.00.E-17
Q13112	Chromatin assembly factor 1 subunit B	0.01	1.00.E-17
Q9Y5Y6	Suppressor of tumorigenicity 14 protein	0.01	1.00.E-17
Q8N4C6-5	Isoform 5 of Ninein	0.01	1.00.E-17
Q9BXF3-2	Isoform B of Cat eye syndrome critical region protein 2	0.01	1.00.E-17
Q9P0V3-2	Isoform 2 of SH3 domain-binding protein 4	0.01	1.00.E-17
Q9GZX6	Interleukin-22	0.01	1.00.E-17
Q12834	Cell division cycle protein 20 homolog	0.01	1.00.E-17
P85298-3	Isoform 3 of Rho GTPase-activating protein 8	0.01	1.00.E-17
Q9Y597-2	Isoform 2 of BTB/POZ domain-containing protein KCTD3	0.01	1.00.E-17
Q8ND30	Liprin-beta-2	0.01	1.00.E-17
Q7Z5L2	Coiled-coil domain-containing protein R3HCC1L	0.01	1.00.E-17
P02679-2	Isoform Gamma-A of Fibrinogen gamma chain	0.01	1.00.E-17
P21802-5	Isoform 5 of Fibroblast growth factor receptor 2	0.01	1.00.E-17
O95239	Chromosome-associated kinesin KIF4A	0.01	1.00.E-17
Q9BU61-2	Isoform b of NADH dehydrogenase [ubiquinone] 1 alpha subcomplex assembly factor 3	0.01	1.00.E-17
Q6ZN04	RNA-binding protein MEX3B	0.01	1.00.E-17
P85298	Rho GTPase-activating protein 8	0.01	1.00.E-17
Q96G01-2	Isoform 2 of Protein bicaudal D homolog 1	0.01	1.00.E-17
P15884-11	Isoform 11 of Transcription factor 4	0.01	1.00.E-17
Q9H6Y2-2	Isoform 2 of WD repeat-containing protein 55	0.01	1.00.E-17
Q15652	Probable JmjC domain-containing histone demethylation protein 2C	0.01	1.00.E-17
Q96D96	Voltage-gated hydrogen channel 1	0.01	1.00.E-17
Q9BSH4	Translational activator of cytochrome c oxidase 1	0.01	1.00.E-17
P04233-2	Isoform 2 of HLA class II histocompatibility antigen gamma chain	0.01	1.00.E-17
Q9BWG4	Single-stranded DNA-binding protein 4	0.01	1.00.E-17
Q5VZ89-7	Isoform 2 of DENN domain-containing protein 4C	0.01	1.00.E-17
P08123	Collagen alpha-2(I) chain	0.01	1.00.E-17
Q96GK7	Fumarylacetoacetate hydrolase domain-containing protein 2A	0.01	1.00.E-17
Q9NVX2	Notchless protein homolog 1	0.01	1.00.E-17
Q14244-6	Isoform 6 of Ensconsin	0.01	1.00.E-17
P15884-14	Isoform C- of Transcription factor 4	0.01	1.00.E-17
Q14CB8-5	Isoform 5 of Rho GTPase-activating protein 19	0.01	1.00.E-17
Q01860-2	Isoform B of POU domain, class 5, transcription factor 1	0.01	1.00.E-17
Q96D96-3	Isoform 3 of Voltage-gated hydrogen channel 1	0.01	1.00.E-17
P02679	Fibrinogen gamma chain	0.01	1.00.E-17

Q14244-4	Isoform 4 of Ensconsin	0.01	1.00.E-17
Q14451-2	Isoform 2 of Growth factor receptor-bound protein 7	0.01	1.00.E-17
O95926-2	Isoform 2 of Pre-mRNA-splicing factor SYF2	0.01	1.00.E-17
Q14244-5	Isoform 5 of Ensconsin	0.01	1.00.E-17
P15884-13	Isoform 13 of Transcription factor 4	0.01	1.00.E-17
Q8N4C6-10	Isoform 3 of Ninein	0.01	1.00.E-17
P57081	tRNA (guanine-N(7)-)-methyltransferase non-catalytic subunit WDR4	0.01	1.00.E-17
Q9BXS6-2	Isoform 2 of Nucleolar and spindle-associated protein 1	0.01	1.00.E-17
Q9Y3Q3	Transmembrane emp24 domain-containing protein 3	0.01	1.00.E-17
Q12962	Transcription initiation factor TFIID subunit 10	0.01	1.00.E-17
P58004	Sestrin-2	0.01	1.00.E-17
P29350-3	Isoform 2 of Tyrosine-protein phosphatase non-receptor type 6	0.01	1.00.E-17
Q9NVM4-2	Isoform 2 of Protein arginine N-methyltransferase 7	0.01	1.00.E-17
Q8TD55-2	Isoform 2 of Pleckstrin homology domain-containing family O member 2	0.01	1.00.E-17
Q9UJQ4	Sal-like protein 4	0.01	1.00.E-17
P15884-16	Isoform I- of Transcription factor 4	0.01	1.00.E-17
O95239-2	Isoform 2 of Chromosome-associated kinesin KIF4A	0.01	1.00.E-17
P29350	Tyrosine-protein phosphatase non-receptor type 6	0.01	1.00.E-17
P21802-8	Isoform 7 of Fibroblast growth factor receptor 2	0.01	1.00.E-17
P98173	Protein FAM3A	0.01	1.00.E-17
P15884-10	Isoform F- of Transcription factor 4	0.01	1.00.E-17
Q8WXC6	COP9 signalosome complex subunit 9	0.01	1.00.E-17
Q9C0B1-3	Isoform 3 of Alpha-ketoglutarate-dependent dioxygenase FTO	0.01	1.00.E-17
Q14244-2	Isoform 2 of Ensconsin	0.01	1.00.E-17
Q9BXS6-3	Isoform 3 of Nucleolar and spindle-associated protein 1	0.01	1.00.E-17
Q14CB8-2	Isoform 2 of Rho GTPase-activating protein 19	0.01	1.00.E-17
Q86XN8-2	Isoform 2 of RNA-binding protein MEX3D	0.01	1.00.E-17
Q96CP6-2	Isoform 2 of Protein Aster-A	0.01	1.00.E-17
P31947	14-3-3 protein sigma	0.01	1.00.E-17
Q9UBN4	Short transient receptor potential channel 4	0.01	1.00.E-17
Q969I6	Sodium-coupled neutral amino acid transporter 4	0.01	1.00.E-17
P59780	AP-3 complex subunit sigma-2	0.01	1.00.E-17
P55210	Caspase-7	0.01	1.00.E-17
Q13439-4	Isoform 4 of Golgin subfamily A member 4	0.01	1.00.E-17
Q9H2S1-2	Isoform 2 of Small conductance calcium-activated potassium channel protein 2	0.01	1.00.E-17
Q9Y2X9	Zinc finger protein 281	0.01	1.00.E-17
P45983-3	Isoform 3 of Mitogen-activated protein kinase 8	0.01	1.00.E-17
P22607-3	Isoform 3 of Fibroblast growth factor receptor 3	0.01	1.00.E-17
Q86U28-2	Isoform 2 of Iron-sulfur cluster assembly 2 homolog, mitochondrial	0.01	1.00.E-17
Q9Y6X9-2	Isoform 2 of ATPase MORC2	0.01	1.00.E-17
Q16875-3	Isoform 3 of 6-phosphofructo-2-kinase/fructose-2,6-bisphosphatase 3	0.01	1.00.E-17
Q9Y6X9	ATPase MORC2	0.01	1.00.E-17
P20839	Inosine-5'-monophosphate dehydrogenase 1	0.01	1.00.E-17
Q05513	Protein kinase C zeta type	0.01	1.00.E-17
Q9BY11	Protein kinase C and casein kinase substrate in neurons protein 1	0.01	1.00.E-17
Q9UJK0	18S rRNA aminocarboxypropyltransferase	0.01	1.00.E-17
Q9BWG4-2	Isoform 2 of Single-stranded DNA-binding protein 4	0.01	1.00.E-17
Q13439-5	Isoform 5 of Golgin subfamily A member 4	0.01	1.00.E-17
Q9Y3B8	Oligoribonuclease, mitochondrial	0.01	1.00.E-17
Q8N4C6-6	Isoform 6 of Ninein	0.01	1.00.E-17
A6ND36	Protein FAM83G	0.01	1.00.E-17
P21802-21	Isoform 15 of Fibroblast growth factor receptor 2	0.01	1.00.E-17
Q9NVM9	Integrator complex subunit 13	0.01	1.00.E-17
Q15652-2	Isoform 2 of Probable JmjC domain-containing histone demethylation protein 2C	0.01	1.00.E-17
Q9UBU8-3	Isoform 3 of Mortality factor 4-like protein 1	0.01	1.00.E-17
Q14244	Ensconsin	0.01	1.00.E-17
Q96CP6	Protein Aster-A	0.01	1.00.E-17
P20839-4	Isoform 4 of Inosine-5'-monophosphate dehydrogenase 1	0.01	1.00.E-17
Q9BV40	Vesicle-associated membrane protein 8	0.01	1.00.E-17
Q6YHU6-6	Isoform 6 of Thyroid adenoma-associated protein	0.01	1.00.E-17
P21802-15	Isoform 9 of Fibroblast growth factor receptor 2	0.01	1.00.E-17
Q9NXS2-3	Isoform 2 of Glutaminyl-peptide cyclotransferase-like protein	0.01	1.00.E-17
Q9NQW6-2	Isoform 2 of Anillin	0.01	1.00.E-17
P21802-22	Isoform 16 of Fibroblast growth factor receptor 2	0.01	1.00.E-17
Q14451	Growth factor receptor-bound protein 7	0.01	1.00.E-17
Q9NVM9-2	Isoform 2 of Integrator complex subunit 13	0.01	1.00.E-17
Q9NSI2-2	Isoform B of Protein FAM207A	0.01	1.00.E-17
P80365	Corticosteroid 11-beta-dehydrogenase isozyme 2	0.01	1.00.E-17
Q8WVC0	RNA polymerase-associated protein LEO1	0.01	1.00.E-17
P21709-3	Isoform 3 of Ephrin type-A receptor 1	0.01	1.00.E-17
Q9BU61	NADH dehydrogenase [ubiquinone] 1 alpha subcomplex assembly factor 3	0.01	1.00.E-17

Q16875	6-phosphofructo-2-kinase/fructose-2,6-bisphosphatase 3	0.01	1.00.E-17
Q9UBN4-5	Isoform Epsilon of Short transient receptor potential channel 4	0.01	1.00.E-17
O60343-5	Isoform 5 of TBC1 domain family member 4	0.01	1.00.E-17
Q6YHU6-2	Isoform 2 of Thyroid adenoma-associated protein	0.01	1.00.E-17
P15884	Transcription factor 4	0.01	1.00.E-17
P15884-15	Isoform C-delta of Transcription factor 4	0.01	1.00.E-17
Q86UZ6	Zinc finger and BTB domain-containing protein 46	0.01	1.00.E-17
Q5VZ89	DENN domain-containing protein 4C	0.01	1.00.E-17
Q9NVM4	Protein arginine N-methyltransferase 7	0.01	1.00.E-17
Q9H469	F-box/LRR-repeat protein 15	0.01	1.00.E-17
Q9NZN4-2	Isoform 2 of EH domain-containing protein 2	0.01	1.00.E-17
P55210-4	Isoform 4 of Caspase-7	0.01	1.00.E-17
P41134-2	Isoform ID-B of DNA-binding protein inhibitor ID-1	0.01	1.00.E-17
P15884-12	Isoform E- of Transcription factor 4	0.01	1.00.E-17
Q9H6Y2	WD repeat-containing protein 55	0.01	1.00.E-17
Q14451-3	Isoform 3 of Growth factor receptor-bound protein 7	0.01	1.00.E-17
Q96D96-4	Isoform 4 of Voltage-gated hydrogen channel 1	0.01	1.00.E-17
Q9NVM4-3	Isoform 3 of Protein arginine N-methyltransferase 7	0.01	1.00.E-17
P51810	G-protein coupled receptor 143	0.01	1.00.E-17
P55957-3	Isoform 3 of BH3-interacting domain death agonist	0.01	1.00.E-17
Q8NI60-3	Isoform 3 of Atypical kinase COQ8A, mitochondrial	0.01	1.00.E-17
P09327-2	Isoform 2 of Villin-1	0.01	1.00.E-17
Q13439-3	Isoform 3 of Golgin subfamily A member 4	0.01	1.00.E-17
O14732	Inositol monophosphatase 2	0.01	1.00.E-17
Q8N442	Translation factor GUF1, mitochondrial	0.01	1.00.E-17
Q5T0Z8	Uncharacterized protein C6orf132	0.01	1.00.E-17
Q86XN8-3	Isoform 3 of RNA-binding protein MEX3D	0.01	1.00.E-17
P20839-6	Isoform 6 of Inosine-5'-monophosphate dehydrogenase 1	0.01	1.00.E-17
Q14653-5	Isoform 5 of Interferon regulatory factor 3	0.01	1.00.E-17
Q9UMX1-3	Isoform 3 of Suppressor of fused homolog	0.01	1.00.E-17
P20839-7	Isoform 7 of Inosine-5'-monophosphate dehydrogenase 1	0.01	1.00.E-17
Q6YHU6	Thyroid adenoma-associated protein	0.01	1.00.E-17
P21802-20	Isoform 14 of Fibroblast growth factor receptor 2	0.01	1.00.E-17
Q14CZ7	FAST kinase domain-containing protein 3, mitochondrial	0.01	1.00.E-17
Q9NRG1	Phosphoribosyltransferase domain-containing protein 1	0.01	1.00.E-17
Q01973-3	Isoform 3 of Inactive tyrosine-protein kinase transmembrane receptor ROR1	0.01	1.00.E-17
O75843	AP-1 complex subunit gamma-like 2	0.01	1.00.E-17
Q9NUL5-2	Isoform 2 of Shiftless antiviral inhibitor of ribosomal frameshifting protein	0.01	1.00.E-17
Q6IQ49-2	Isoform 2 of Replication stress response regulator SDE2	0.01	1.00.E-17
Q7Z5L2-2	Isoform 2 of Coiled-coil domain-containing protein R3HCC1L	0.01	1.00.E-17
P21802-4	Isoform 4 of Fibroblast growth factor receptor 2	0.01	1.00.E-17
O75143	Autophagy-related protein 13	0.01	1.00.E-17
P55210-3	Isoform Alpha' of Caspase-7	0.01	1.00.E-17
Q9H501	ESF1 homolog	0.01	1.00.E-17
P30414	NK-tumor recognition protein	0.01	1.00.E-17
Q9H6D7-3	Isoform 3 of HAUS augmin-like complex subunit 4	0.01	1.00.E-17
P15884-3	Isoform SEF2-1D of Transcription factor 4	0.01	1.00.E-17
Q9NZN4	EH domain-containing protein 2	0.01	1.00.E-17

Supplementary Table S1. (f) Proteins with decreased levels in the DAs (<0.5 fold)

Accession	Protein Name	Abundance Ratio: (DA) / (iPSC)	Abundance Ratio p-Value: (DA) / (iPSC)
P49736	DNA replication licensing factor MCM2	0.029	4.57.E-03
P06748	Nucleophosmin	0.053	2.71.E-02
P06748-2	Isoform 2 of Nucleophosmin	0.053	2.71.E-02
P06748-3	Isoform 3 of Nucleophosmin	0.051	2.42.E-02
Q14566	DNA replication licensing factor MCM6	0.035	8.36.E-03
P25205	DNA replication licensing factor MCM3	0.029	4.67.E-03
P25205-2	Isoform 2 of DNA replication licensing factor MCM3	0.029	4.90.E-03
P05787	Keratin, type II cytoskeletal 8	0.051	2.46.E-02
P05787-2	Isoform 2 of Keratin, type II cytoskeletal 8	0.051	2.46.E-02
P05783	Keratin, type I cytoskeletal 18	0.046	1.85.E-02
P12004	Proliferating cell nuclear antigen	0.041	1.37.E-02
Q9UDR5	Alpha-aminoadipic semialdehyde synthase, mitochondrial	0.045	1.77.E-02
P08727	Keratin, type I cytoskeletal 19	0.042	1.39.E-02
P33991	DNA replication licensing factor MCM4	0.029	4.80.E-03
P52292	Importin subunit alpha-1	0.036	8.93.E-03
Q8NC51	Plasminogen activator inhibitor 1 RNA-binding protein	0.04	1.26.E-02
Q8NC51-2	Isoform 2 of Plasminogen activator inhibitor 1 RNA-binding protein	0.037	1.03.E-02
P0DN79	Cystathionine beta-synthase-like protein	0.05	2.36.E-02
P35520-2	Isoform 2 of Cystathionine beta-synthase	0.05	2.36.E-02
P06454-2	Isoform 2 of Prothymosin alpha	0.062	3.19.E-02
P06454	Prothymosin alpha	0.062	3.19.E-02
Q9NR30	Nucleolar RNA helicase 2	0.044	1.69.E-02
Q9NR30-2	Isoform 2 of Nucleolar RNA helicase 2	0.046	1.85.E-02
Q9H9Z2	Protein lin-28 homolog A	0.01	1.14.E-04
P33992	DNA replication licensing factor MCM5	0.029	4.90.E-03
P06493-2	Isoform 2 of Cyclin-dependent kinase 1	0.066	4.85.E-02
Q9Y5B9	FACT complex subunit SPT16	0.046	1.82.E-02
P19012	Keratin, type I cytoskeletal 15	0.05	2.05.E-02
P33993	DNA replication licensing factor MCM7	0.03	5.52.E-03
P05186-3	Isoform 3 of Alkaline phosphatase, tissue-nonspecific isozyme	0.055	6.46.E-03
P05186	Alkaline phosphatase, tissue-nonspecific isozyme	0.059	9.39.E-03
P63220	40S ribosomal protein S21	0.048	2.09.E-02
Q04695	Keratin, type I cytoskeletal 17	0.05	2.14.E-02
P05186-2	Isoform 2 of Alkaline phosphatase, tissue-nonspecific isozyme	0.064	1.80.E-02
P18858-3	Isoform 3 of DNA ligase 1	0.073	4.01.E-02
P33552	Cyclin-dependent kinases regulatory subunit 2	0.057	2.91.E-02
Q13283-2	Isoform 2 of Ras GTPase-activating protein-binding protein 1	0.091	4.67.E-02
Q13765	Nascent polypeptide-associated complex subunit alpha	0.065	3.92.E-02
E9PAV3	Nascent polypeptide-associated complex subunit alpha, muscle-specific form	0.065	3.92.E-02
E9PAV3-2	Isoform skNAC-2 of Nascent polypeptide-associated complex subunit alpha, muscle-specific form	0.065	3.92.E-02
Q8TDB8-3	Isoform 3 of Solute carrier family 2, facilitated glucose transporter member 14	0.079	3.82.E-02
P33316	Deoxyuridine 5'-triphosphate nucleotidohydrolase, mitochondrial	0.048	2.05.E-02
P33993-3	Isoform 3 of DNA replication licensing factor MCM7	0.03	5.08.E-03
Q08945	FACT complex subunit SSRP1	0.056	3.07.E-02
Q16576-2	Isoform 2 of Histone-binding protein RBBP7	0.087	4.84.E-02
Q9P258	Protein RCC2	0.065	2.04.E-02
P13646-2	Isoform 2 of Keratin, type I cytoskeletal 13	0.05	1.22.E-02
P13646	Keratin, type I cytoskeletal 13	0.051	1.68.E-02
P13646-3	Isoform 3 of Keratin, type I cytoskeletal 13	0.051	1.68.E-02
P56747	Claudin-6	0.071	2.85.E-02
Q8NC51-3	Isoform 3 of Plasminogen activator inhibitor 1 RNA-binding protein	0.084	2.77.E-02
Q8NC51-4	Isoform 4 of Plasminogen activator inhibitor 1 RNA-binding protein	0.084	2.77.E-02
P18583	Protein SON	0.094	4.82.E-02
P18583-9	Isoform I of Protein SON	0.094	4.82.E-02
P18583-7	Isoform G of Protein SON	0.094	4.82.E-02
Q9BTE3	Mini-chromosome maintenance complex-binding protein	0.094	4.40.E-02
Q9BTE3-2	Isoform 2 of Mini-chromosome maintenance complex-binding protein	0.094	4.46.E-02
P33993-2	Isoform 2 of DNA replication licensing factor MCM7	0.03	5.52.E-03
Q9NX02-5	Isoform 5 of NACHT, LRR and PYD domains-containing protein 2	0.068	4.42.E-02
Q9NX02	NACHT, LRR and PYD domains-containing protein 2	0.068	4.42.E-02
Q9NX02-4	Isoform 4 of NACHT, LRR and PYD domains-containing protein 2	0.068	4.98.E-02
Q9NX02-2	Isoform 2 of NACHT, LRR and PYD domains-containing protein 2	0.068	4.42.E-02
Q15323	Keratin, type I cuticular Ha1	0.049	8.99.E-03

Q2M2I5	Keratin, type I cytoskeletal 24	0.049	8.99.E-03
O76015	Keratin, type I cuticular Ha8	0.049	8.99.E-03
Q14532	Keratin, type I cuticular Ha2	0.049	8.99.E-03
Q14525	Keratin, type I cuticular Ha3-II	0.049	8.99.E-03
O76013	Keratin, type I cuticular Ha6	0.049	8.99.E-03
O76013-2	Isoform 2 of Keratin, type I cuticular Ha6	0.049	8.99.E-03
O76014	Keratin, type I cuticular Ha7	0.049	8.99.E-03
Q59GN2	Putative 60S ribosomal protein L39-like 5	0.068	1.61.E-02
P62891	60S ribosomal protein L39	0.068	1.61.E-02
Q9BQG0-2	Isoform 2 of Myb-binding protein 1A	0.093	4.99.E-02
Q9BQG0	Myb-binding protein 1A	0.093	4.99.E-02
Q99456	Keratin, type I cytoskeletal 12	0.046	1.80.E-02
O14602	Eukaryotic translation initiation factor 1A, Y-chromosomal	0.063	2.16.E-02
Q8IZL8	Proline-, glutamic acid- and leucine-rich protein 1	0.069	2.99.E-02
Q9NX02-3	Isoform 3 of NACHT, LRR and PYD domains-containing protein 2	0.068	2.98.E-02
O00592	Podocalyxin	0.014	3.76.E-04
O00592-2	Isoform 2 of Podocalyxin	0.014	3.76.E-04
Q05682-5	Isoform 5 of Caldesmon	0.048	7.59.E-03
Q05682-4	Isoform 4 of Caldesmon	0.048	7.59.E-03
Q05682-6	Isoform 6 of Caldesmon	0.048	7.59.E-03
Q05682-2	Isoform 2 of Caldesmon	0.048	7.59.E-03
Q05682-3	Isoform 3 of Caldesmon	0.048	7.59.E-03
Q05682	Caldesmon	0.048	7.59.E-03
Q96SQ9-2	Isoform 2 of Cytochrome P450 2S1	0.016	5.56.E-04
Q96SQ9	Cytochrome P450 2S1	0.016	5.56.E-04
Q9BTE3-3	Isoform 3 of Mini-chromosome maintenance complex-binding protein	0.077	3.52.E-02
P18583-10	Isoform J of Protein SON	0.086	4.65.E-02
P18583-3	Isoform B of Protein SON	0.086	4.65.E-02
P18583-4	Isoform C of Protein SON	0.086	4.65.E-02
Q5T7N2	LINE-1 type transposase domain-containing protein 1	0.041	1.30.E-02
Q01628	Interferon-induced transmembrane protein 3	0.054	1.12.E-02
P18583-6	Isoform E of Protein SON	0.084	4.55.E-02
Q9BV38	WD repeat-containing protein 18	0.087	3.85.E-02
P35250	Replication factor C subunit 2	0.086	4.90.E-02
Q9BQI0	Allograft inflammatory factor 1-like	0.072	4.75.E-02
Q9Y624	Junctional adhesion molecule A	0.058	1.82.E-02
Q9BZK3	Putative nascent polypeptide-associated complex subunit alpha-like protein	0.037	3.91.E-03
P12830	Cadherin-1	0.048	1.03.E-02
Q8WX94	NACHT, LRR and PYD domains-containing protein 7	0.059	3.00.E-02
Q8WX94-2	Isoform 2 of NACHT, LRR and PYD domains-containing protein 7	0.059	3.00.E-02
Q8WX94-3	Isoform 3 of NACHT, LRR and PYD domains-containing protein 7	0.059	3.00.E-02
P30154-3	Isoform 3 of Serine/threonine-protein phosphatase 2A 65 kDa regulatory subunit A beta isoform	0.083	4.72.E-02
P30154-2	Isoform 2 of Serine/threonine-protein phosphatase 2A 65 kDa regulatory subunit A beta isoform	0.083	4.72.E-02
P30154	Serine/threonine-protein phosphatase 2A 65 kDa regulatory subunit A beta isoform	0.083	4.72.E-02
Q9BQI0-3	Isoform 3 of Allograft inflammatory factor 1-like	0.065	4.02.E-02
Q9BYZ2	L-lactate dehydrogenase A-like 6B	0.071	4.35.E-02
O76021-2	Isoform 2 of Ribosomal L1 domain-containing protein 1	0.056	4.38.E-02
Q14126	Desmoglein-2	0.052	1.05.E-02
P40938	Replication factor C subunit 3	0.083	4.45.E-02
P19012-2	Isoform 2 of Keratin, type I cytoskeletal 15	0.039	5.35.E-03
Q9Y467	Sal-like protein 2	0.059	2.89.E-02
Q8WY07	Cationic amino acid transporter 3	0.073	4.58.E-02
Q9H910-3	Isoform 3 of Jupiter microtubule associated homolog 2	0.084	3.99.E-02
Q9H910-2	Isoform 2 of Jupiter microtubule associated homolog 2	0.084	4.46.E-02
Q9H910	Jupiter microtubule associated homolog 2	0.084	3.99.E-02
O95905-3	Isoform 3 of Protein ecdysoneless homolog	0.08	4.02.E-02
O95905	Protein ecdysoneless homolog	0.08	4.02.E-02
O95905-2	Isoform 2 of Protein ecdysoneless homolog	0.08	3.98.E-02
P10620	Microsomal glutathione S-transferase 1	0.044	8.30.E-03
Q99543	DnaJ homolog subfamily C member 2	0.054	3.47.E-02
Q99543-2	Isoform 2 of DnaJ homolog subfamily C member 2	0.054	3.37.E-02
Q9Y4W2-3	Isoform 3 of Ribosomal biogenesis protein LAS1L	0.052	2.92.E-02
Q9Y4W2	Ribosomal biogenesis protein LAS1L	0.052	2.38.E-02
Q9Y4W2-2	Isoform 2 of Ribosomal biogenesis protein LAS1L	0.052	2.38.E-02
Q99584	Protein S100-A13	0.025	7.25.E-03
P40938-2	Isoform 2 of Replication factor C subunit 3	0.061	3.13.E-02

P12830-2	Isoform 2 of Cadherin-1	0.041	5.57.E-03
O15013-5	Isoform 5 of Rho guanine nucleotide exchange factor 10	0.066	2.11.E-02
O15013-4	Isoform 4 of Rho guanine nucleotide exchange factor 10	0.066	2.11.E-02
O15013-7	Isoform 3 of Rho guanine nucleotide exchange factor 10	0.066	2.11.E-02
O15013	Rho guanine nucleotide exchange factor 10	0.066	2.11.E-02
O15013-6	Isoform 2 of Rho guanine nucleotide exchange factor 10	0.066	2.11.E-02
Q96EB6	NAD-dependent protein deacetylase sirtuin-1	0.069	2.92.E-02
Q9Y2S6	Translation machinery-associated protein 7	0.04	9.79.E-03
P17302	Gap junction alpha-1 protein	0.018	2.62.E-04
O15347	High mobility group protein B3	0.043	2.49.E-02
Q14258	E3 ubiquitin/ISG15 ligase TRIM25	0.053	2.43.E-02
O14745	Na(+)/H(+) exchange regulatory cofactor NHE-RF1	0.066	4.31.E-02
O76074-2	Isoform PDE5A2 of cGMP-specific 3',5'-cyclic phosphodiesterase	0.03	8.83.E-03
O76074	cGMP-specific 3',5'-cyclic phosphodiesterase	0.03	8.83.E-03
O60869-2	Isoform 2 of Endothelial differentiation-related factor 1	0.059	4.65.E-02
O60869	Endothelial differentiation-related factor 1	0.059	4.65.E-02
O60869-3	Isoform 3 of Endothelial differentiation-related factor 1	0.059	4.65.E-02
Q99848	Probable rRNA-processing protein EBP2	0.067	2.64.E-02
P26358	DNA (cytosine-5)-methyltransferase 1	0.072	4.20.E-02
P26358-2	Isoform 2 of DNA (cytosine-5)-methyltransferase 1	0.072	4.20.E-02
P51153	Ras-related protein Rab-13	0.066	2.65.E-02
P09038-1	Isoform 2 of Fibroblast growth factor 2	0.063	3.44.E-02
P09038-3	Isoform 4 of Fibroblast growth factor 2	0.063	3.44.E-02
P09038	Fibroblast growth factor 2	0.063	3.44.E-02
P09038-2	Isoform 3 of Fibroblast growth factor 2	0.063	3.44.E-02
Q99538-3	Isoform 3 of Legumain	0.03	2.11.E-03
Q99538	Legumain	0.03	2.11.E-03
Q99538-2	Isoform 2 of Legumain	0.03	2.11.E-03
Q9Y4W2-4	Isoform 4 of Ribosomal biogenesis protein LAS1L	0.052	1.89.E-02
O75792	Ribonuclease H2 subunit A	0.065	3.06.E-02
Q8TDD1	ATP-dependent RNA helicase DDX54	0.079	3.99.E-02
Q8TDD1-2	Isoform 2 of ATP-dependent RNA helicase DDX54	0.079	3.99.E-02
Q7Z2W4-3	Isoform 3 of Zinc finger CCCH-type antiviral protein 1	0.046	1.43.E-02
Q9BRP8	Partner of Y14 and mago	0.05	1.39.E-02
Q9BRP8-2	Isoform 2 of Partner of Y14 and mago	0.05	1.39.E-02
Q9H1E3	Nuclear ubiquitous casein and cyclin-dependent kinase substrate 1	0.034	6.59.E-03
Q9H1E3-2	Isoform 2 of Nuclear ubiquitous casein and cyclin-dependent kinase substrate 1	0.034	3.12.E-03
Q9NX55-3	Isoform 3 of Huntingtin-interacting protein K	0.045	2.18.E-02
P09493-4	Isoform 4 of Tropomyosin alpha-1 chain	0.041	6.28.E-03
P09493-10	Isoform 10 of Tropomyosin alpha-1 chain	0.038	3.81.E-03
Q9NUQ6-3	Isoform 3 of SPATS2-like protein	0.034	1.45.E-02
Q9NUQ6-2	Isoform 2 of SPATS2-like protein	0.034	3.44.E-03
Q9NUQ6	SPATS2-like protein	0.034	3.44.E-03
Q9NUQ6-4	Isoform 4 of SPATS2-like protein	0.034	1.45.E-02
Q96EY4	Translation machinery-associated protein 16	0.073	4.41.E-02
Q6P5R6	60S ribosomal protein L22-like 1	0.054	2.94.E-02
Q9H583	HEAT repeat-containing protein 1	0.067	2.54.E-02
Q8NFC6	Biorientation of chromosomes in cell division protein 1-like 1	0.082	4.69.E-02
Q15642-5	Isoform 5 of Cdc42-interacting protein 4	0.05	1.12.E-02
Q8WU90	Zinc finger CCCH domain-containing protein 15	0.035	6.23.E-03
Q9NVZ3-4	Isoform 4 of Adaptin ear-binding coat-associated protein 2	0.087	4.63.E-02
Q8NBT2-2	Isoform 2 of Kinetochore protein Spc24	0.078	4.25.E-02
Q9BRX5	DNA replication complex GINS protein PSF3	0.051	1.01.E-02
Q9NRN5-3	Isoform 3 of Olfactomedin-like protein 3	0.026	4.63.E-03
Q9NRN5	Olfactomedin-like protein 3	0.026	4.63.E-03
Q9NRN5-2	Isoform 2 of Olfactomedin-like protein 3	0.026	4.63.E-03
Q7Z2T5-2	Isoform 2 of TRMT1-like protein	0.063	2.90.E-02
Q96P48-2	Isoform 2 of Arf-GAP with Rho-GAP domain, ANK repeat and PH domain-containing protein 1	0.054	1.22.E-02
Q96P48	Arf-GAP with Rho-GAP domain, ANK repeat and PH domain-containing protein 1	0.054	1.29.E-02
Q96P48-7	Isoform 7 of Arf-GAP with Rho-GAP domain, ANK repeat and PH domain-containing protein 1	0.054	1.22.E-02
Q96P48-4	Isoform 4 of Arf-GAP with Rho-GAP domain, ANK repeat and PH domain-containing protein 1	0.054	1.22.E-02
Q96P48-3	Isoform 3 of Arf-GAP with Rho-GAP domain, ANK repeat and PH domain-containing protein 1	0.054	1.29.E-02
Q96P48-1	Isoform 1 of Arf-GAP with Rho-GAP domain, ANK repeat and PH domain-containing protein 1	0.054	1.22.E-02
Q96P48-5	Isoform 5 of Arf-GAP with Rho-GAP domain, ANK repeat and PH domain-containing protein 1	0.054	2.02.E-02
P21926	CD9 antigen	0.064	2.95.E-02
Q8NI36	WD repeat-containing protein 36	0.086	4.89.E-02

P11532-7	Isoform 12 of Dystrophin	0.075	4.40.E-02
P11532-8	Isoform 13 of Dystrophin	0.075	4.40.E-02
P11532-18	Isoform 17 of Dystrophin	0.075	4.40.E-02
P11532-6	Isoform 14 of Dystrophin	0.075	4.40.E-02
P11532-5	Isoform 15 of Dystrophin	0.075	4.40.E-02
P11532-9	Isoform 16 of Dystrophin	0.075	4.40.E-02
O60343-3	Isoform 3 of TBC1 domain family member 4	0.044	8.11.E-03
O60343	TBC1 domain family member 4	0.044	8.11.E-03
O60343-2	Isoform 2 of TBC1 domain family member 4	0.044	8.11.E-03
Q27J81-3	Isoform 3 of Inverted formin-2	0.082	4.68.E-02
Q56P03	E2F-associated phosphoprotein	0.083	4.79.E-02
Q8IWJ2	GRIP and coiled-coil domain-containing protein 2	0.06	2.44.E-02
Q03135-2	Isoform 2 of Caveolin-1	0.025	4.50.E-03
Q03135	Caveolin-1	0.025	4.50.E-03
P04183	Thymidine kinase, cytosolic	0.048	1.88.E-02
P09493	Tropomyosin alpha-1 chain	0.032	1.88.E-03
P56182	Ribosomal RNA processing protein 1 homolog A	0.038	1.49.E-02
Q5C9Z4	Nucleolar MIF4G domain-containing protein 1	0.072	3.68.E-02
Q13485	Mothers against decapentaplegic homolog 4	0.053	2.23.E-02
Q3B7T1-5	Isoform 4 of Erythroid differentiation-related factor 1	0.074	4.63.E-02
Q3B7T1-3	Isoform 2 of Erythroid differentiation-related factor 1	0.074	4.31.E-02
Q3B7T1	Erythroid differentiation-related factor 1	0.074	4.63.E-02
Q3B7T1-4	Isoform 3 of Erythroid differentiation-related factor 1	0.074	4.59.E-02
P54687	Branched-chain-amino-acid aminotransferase, cytosolic	0.061	3.50.E-02
P54687-3	Isoform 3 of Branched-chain-amino-acid aminotransferase, cytosolic	0.061	3.50.E-02
P49643	DNA primase large subunit	0.022	5.86.E-03
Q15542-2	Isoform Short of Transcription initiation factor TFIID subunit 5	0.07	4.57.E-02
Q15542	Transcription initiation factor TFIID subunit 5	0.07	4.57.E-02
Q9H900	Protein zwilch homolog	0.034	7.36.E-03
Q9H900-2	Isoform 2 of Protein zwilch homolog	0.031	2.47.E-03
O15234	Protein CASC3	0.056	1.85.E-02
Q5JTD0-4	Isoform 4 of Tight junction-associated protein 1	0.079	4.79.E-02
O00443	Phosphatidylinositol 4-phosphate 3-kinase C2 domain-containing subunit alpha	0.066	2.60.E-02
P50579-3	Isoform 3 of Methionine aminopeptidase 2	0.029	2.00.E-03
P50579	Methionine aminopeptidase 2	0.029	2.66.E-03
Q96II8-4	Isoform 4 of DISP complex protein LRCH3	0.01	1.00.E-17
O75970	Multiple PDZ domain protein	0.01	1.00.E-17
Q4LE39	AT-rich interactive domain-containing protein 4B	0.01	1.00.E-17
O75616	GTPase Era, mitochondrial	0.01	1.00.E-17
Q68EM7-6	Isoform 6 of Rho GTPase-activating protein 17	0.01	1.00.E-17
Q9H9Y6-3	Isoform 3 of DNA-directed RNA polymerase I subunit RPA2	0.025	1.89.E-03
Q9ULU4	Protein kinase C-binding protein 1	0.01	1.00.E-17
P30291-2	Isoform 2 of Wee1-like protein kinase	0.01	1.00.E-17
Q8N5C6	S1 RNA-binding domain-containing protein 1	0.01	1.00.E-17
Q16647	Prostacyclin synthase	0.01	1.00.E-17
Q96RL1-2	Isoform 2 of BRCA1-A complex subunit RAP80	0.01	1.00.E-17
Q16342	Programmed cell death protein 2	0.01	1.00.E-17
Q9BXS9-4	Isoform 4 of Solute carrier family 26 member 6	0.01	1.00.E-17
Q9Y221-2	Isoform 2 of 60S ribosome subunit biogenesis protein NIP7 homolog	0.01	1.00.E-17
Q9UP95-5	Isoform 5 of Solute carrier family 12 member 4	0.01	1.00.E-17
P22607-2	Isoform 2 of Fibroblast growth factor receptor 3	0.01	1.00.E-17
Q86U28	Iron-sulfur cluster assembly 2 homolog, mitochondrial	0.01	1.00.E-17
Q8IWA0	WD repeat-containing protein 75	0.01	1.00.E-17
Q13111-2	Isoform 2 of Chromatin assembly factor 1 subunit A	0.01	1.00.E-17
O75391	Sperm-associated antigen 7	0.01	1.00.E-17
Q8TD55	Pleckstrin homology domain-containing family O member 2	0.01	1.00.E-17
O43913-2	Isoform 2 of Origin recognition complex subunit 5	0.01	1.00.E-17
P48507-2	Isoform 2 of Glutamate--cysteine ligase regulatory subunit	0.01	1.00.E-17
Q13439	Golgin subfamily A member 4	0.01	1.00.E-17
Q9ULU4-14	Isoform 14 of Protein kinase C-binding protein 1	0.01	1.00.E-17
Q96SZ6-3	Isoform 3 of Mitochondrial tRNA methylthiotransferase CDK5RAP1	0.01	1.00.E-17
O60343-4	Isoform 4 of TBC1 domain family member 4	0.01	1.00.E-17
Q99590	Protein SCAF11	0.01	1.00.E-17
Q9BT73	Proteasome assembly chaperone 3	0.01	1.00.E-17
Q6XZF7	Dynamin-binding protein	0.01	1.00.E-17
Q15746-2	Isoform 2 of Myosin light chain kinase, smooth muscle	0.01	1.00.E-17

Q14202	Zinc finger MYM-type protein 3	0.01	1.00.E-17
Q15910-3	Isoform 3 of Histone-lysine N-methyltransferase EZH2	0.01	1.00.E-17
Q86XI2	Condensin-2 complex subunit G2	0.01	1.00.E-17
O95926	Pre-mRNA-splicing factor SYF2	0.01	1.00.E-17
O14965	Aurora kinase A	0.01	1.00.E-17
Q15269	Periodic tryptophan protein 2 homolog	0.01	1.00.E-17
Q9NWU5-3	Isoform 3 of 39S ribosomal protein L22, mitochondrial	0.01	1.00.E-17
Q2NKX8	DNA excision repair protein ERCC-6-like	0.01	1.00.E-17
Q8IXQ5-4	Isoform 4 of Kelch-like protein 7	0.01	1.00.E-17
O15230-2	Isoform 2 of Laminin subunit alpha-5	0.01	1.00.E-17
P15884-5	Isoform B-delta of Transcription factor 4	0.01	1.00.E-17
Q8NFJ9-3	Isoform 2 of Bardet-Biedl syndrome 1 protein	0.01	1.00.E-17
Q9NRZ9-9	Isoform 9 of Lymphoid-specific helicase	0.01	1.00.E-17
Q9NVH2	Integrator complex subunit 7	0.01	1.00.E-17
Q9BXS9-3	Isoform 3 of Solute carrier family 26 member 6	0.01	1.00.E-17
Q96DZ1	Endoplasmic reticulum lectin 1	0.01	1.00.E-17
Q8TBB5-3	Isoform 3 of Kelch domain-containing protein 4	0.01	1.00.E-17
Q9Y3B8-2	Isoform 2 of Oligoribonuclease, mitochondrial	0.01	1.00.E-17
Q8NFJ9	Bardet-Biedl syndrome 1 protein	0.01	1.00.E-17
P84101-2	Isoform 2 of Small EDRK-rich factor 2	0.01	1.00.E-17
Q8WVC0-2	Isoform 2 of RNA polymerase-associated protein LEO1	0.01	1.00.E-17
Q8WUP2-3	Isoform 3 of Filamin-binding LIM protein 1	0.01	1.00.E-17
Q8TE77	Protein phosphatase Slingshot homolog 3	0.01	1.00.E-17
Q8IXQ5	Kelch-like protein 7	0.01	1.00.E-17
O14647	Chromodomain-helicase-DNA-binding protein 2	0.01	1.00.E-17
Q8IXQ5-3	Isoform 3 of Kelch-like protein 7	0.01	1.00.E-17
Q9NVH2-3	Isoform 3 of Integrator complex subunit 7	0.01	1.00.E-17
Q9BT25-3	Isoform 3 of HAUS augmin-like complex subunit 8	0.01	1.00.E-17
Q96FC9-4	Isoform 4 of ATP-dependent DNA helicase DDX11	0.01	1.00.E-17
Q14676-2	Isoform 2 of Mediator of DNA damage checkpoint protein 1	0.05	2.46.E-02
Q5HYK3	2-methoxy-6-polyprenyl-1,4-benzoquinol methylase, mitochondrial	0.01	1.00.E-17
Q9P2D1	Chromodomain-helicase-DNA-binding protein 7	0.01	1.00.E-17
Q9UET6	Putative tRNA (cytidine(32)/guanosine(34)-2'-O)-methyltransferase	0.01	1.00.E-17
Q9UJ83-2	Isoform 2 of 2-hydroxyacyl-CoA lyase 1	0.01	1.00.E-17
Q15742	NGFI-A-binding protein 2	0.01	1.00.E-17
Q9NQS7-2	Isoform 2 of Inner centromere protein	0.01	1.00.E-17
Q9NSI2	Protein FAM207A	0.01	1.00.E-17
Q9BRP4-2	Isoform 2 of Proteasomal ATPase-associated factor 1	0.01	1.00.E-17
Q9ULU4-18	Isoform 18 of Protein kinase C-binding protein 1	0.01	1.00.E-17
Q8N108	Mesoderm induction early response protein 1	0.01	1.00.E-17
Q9H813	Proton-activated chloride channel	0.01	1.00.E-17
O76080	AN1-type zinc finger protein 5	0.01	1.00.E-17
Q92925-3	Isoform 3 of SWI/SNF-related matrix-associated actin-dependent regulator of chromatin subfamily D member 2	0.01	1.00.E-17
O15446-2	Isoform 2 of DNA-directed RNA polymerase I subunit RPA34	0.01	1.00.E-17
Q9UIG0	Tyrosine-protein kinase BAZ1B	0.01	1.00.E-17
Q9BRX5-2	Isoform 2 of DNA replication complex GINS protein PSF3	0.01	1.00.E-17
Q9UN81	LINE-1 retrotransposable element ORF1 protein	0.01	1.00.E-17
Q92636-2	Isoform 2 of Protein FAN	0.01	1.00.E-17
Q96QT6-2	Isoform 2 of PHD finger protein 12	0.01	1.00.E-17
P33981	Dual specificity protein kinase TTK	0.01	1.00.E-17
Q96L91-2	Isoform 2 of E1A-binding protein p400	0.01	1.00.E-17
Q9H426-2	Isoform 2 of Regulating synaptic membrane exocytosis protein 4	0.01	1.00.E-17
O75970-2	Isoform 2 of Multiple PDZ domain protein	0.01	1.00.E-17
Q6PGN9-2	Isoform A of Proline/serine-rich coiled-coil protein 1	0.01	1.00.E-17
Q4LE39-4	Isoform 4 of AT-rich interactive domain-containing protein 4B	0.01	1.00.E-17
Q5VZL5	Zinc finger MYM-type protein 4	0.01	1.00.E-17
P21802-23	Isoform 17 of Fibroblast growth factor receptor 2	0.01	1.00.E-17
Q13574-4	Isoform 4 of Diacylglycerol kinase zeta	0.01	1.00.E-17
Q9H078-3	Isoform 3 of Caseinolytic peptidase B protein homolog	0.01	1.00.E-17
Q9BRF8	Serine/threonine-protein phosphatase CPPED1	0.01	1.00.E-17
P30307-2	Isoform 2 of M-phase inducer phosphatase 3	0.01	1.00.E-17
Q8WW01-2	Isoform 2 of tRNA-splicing endonuclease subunit Sen15	0.01	1.00.E-17
P57081-3	Isoform 3 of tRNA (guanine-N(7)-)-methyltransferase non-catalytic subunit WDR4	0.01	1.00.E-17
Q96GD4-2	Isoform 2 of Aurora kinase B	0.01	1.00.E-17
Q8TD26	Chromodomain-helicase-DNA-binding protein 6	0.01	1.00.E-17

Q9NVU0-3	Isoform 3 of DNA-directed RNA polymerase III subunit RPC5	0.043	2.42.E-02
Q9UP95	Solute carrier family 12 member 4	0.01	1.00.E-17
Q96L91-3	Isoform 3 of E1A-binding protein p400	0.01	1.00.E-17
Q9ULU4-16	Isoform 16 of Protein kinase C-binding protein 1	0.01	1.00.E-17
Q05932-4	Isoform 4 of Folylpolyglutamate synthase, mitochondrial	0.037	1.12.E-02
P15884-6	Isoform A- of Transcription factor 4	0.01	1.00.E-17
O75362	Zinc finger protein 217	0.01	1.00.E-17
Q6ZT62-2	Isoform Short BGIN of Bargin	0.01	1.00.E-17
Q9NNW5	WD repeat-containing protein 6	0.01	1.00.E-17
P21802-3	Isoform 3 of Fibroblast growth factor receptor 2	0.01	1.00.E-17
Q8IXQ5-2	Isoform 2 of Kelch-like protein 7	0.01	1.00.E-17
P49643-2	Isoform 2 of DNA primase large subunit	0.01	1.00.E-17
P20839-2	Isoform 2 of Inosine-5'-monophosphate dehydrogenase 1	0.01	1.00.E-17
Q8NEY8-7	Isoform 7 of Periphilin-1	0.01	1.00.E-17
O14732-2	Isoform 2 of Inositol monophosphatase 2	0.01	1.00.E-17
Q96GD4-3	Isoform 3 of Aurora kinase B	0.01	1.00.E-17
Q9UNN8	Endothelial protein C receptor	0.01	1.00.E-17
P55211-4	Isoform 4 of Caspase-9	0.01	1.00.E-17
Q96PV7-2	Isoform 2 of Protein FAM193B	0.01	1.00.E-17
Q04721	Neurogenic locus notch homolog protein 2	0.01	1.00.E-17
Q5TA45-5	Isoform 5 of Integrator complex subunit 11	0.01	1.00.E-17
Q14696	LRP chaperone MESD	0.01	1.00.E-17
Q7Z6K3	Protein prenyltransferase alpha subunit repeat-containing protein 1	0.01	1.00.E-17
Q8IYM9-2	Isoform 2 of E3 ubiquitin-protein ligase TRIM22	0.01	1.00.E-17
Q9H9P8	L-2-hydroxyglutarate dehydrogenase, mitochondrial	0.01	1.00.E-17
Q86Y37-4	Isoform 4 of CDK2-associated and cullin domain-containing protein 1	0.01	1.00.E-17
Q09666	Neuroblast differentiation-associated protein AHNAK	0.01	1.00.E-17
P26447	Protein S100-A4	0.03	2.14.E-03
O14757-3	Isoform 3 of Serine/threonine-protein kinase Chk1	0.01	1.00.E-17
Q9ULU4-12	Isoform 12 of Protein kinase C-binding protein 1	0.01	1.00.E-17
Q96RL1-3	Isoform 3 of BRCA1-A complex subunit RAP80	0.01	1.00.E-17
Q9BT25	HAUS augmin-like complex subunit 8	0.01	1.00.E-17
Q5VZ89-5	Isoform 5 of DENN domain-containing protein 4C	0.01	1.00.E-17
P0CG30	Glutathione S-transferase theta-2B	0.01	1.00.E-17
Q86X29-3	Isoform 3 of Lipolysis-stimulated lipoprotein receptor	0.01	1.00.E-17
O95816-2	Isoform 2 of BAG family molecular chaperone regulator 2	0.01	1.00.E-17
Q8WWK9-6	Isoform 4 of Cytoskeleton-associated protein 2	0.01	1.00.E-17
Q9UJ83-3	Isoform 3 of 2-hydroxyacyl-CoA lyase 1	0.01	1.00.E-17
Q86XI2-2	Isoform 2 of Condensin-2 complex subunit G2	0.01	1.00.E-17
Q06547-2	Isoform 2 of GA-binding protein subunit beta-1	0.01	1.00.E-17
Q13277	Syntaxin-3	0.01	1.00.E-17
Q9H078-5	Isoform 5 of Caseinolytic peptidase B protein homolog	0.01	1.00.E-17
Q00534	Cyclin-dependent kinase 6	0.01	1.00.E-17
P85298-5	Isoform 5 of Rho GTPase-activating protein 8	0.01	1.00.E-17
O43505	Beta-1,4-glucuronyltransferase 1	0.01	1.00.E-17
Q96KG7-2	Isoform 2 of Multiple epidermal growth factor-like domains protein 10	0.01	1.00.E-17
Q8NHQ9	ATP-dependent RNA helicase DDX55	0.01	1.00.E-17
Q9Y2X0-2	Isoform 2 of Mediator of RNA polymerase II transcription subunit 16	0.01	1.00.E-17
Q9Y385	Ubiquitin-conjugating enzyme E2 J1	0.01	1.00.E-17
Q96GD4-5	Isoform 5 of Aurora kinase B	0.01	1.00.E-17
P0CG13	Chromosome transmission fidelity protein 8 homolog	0.01	1.00.E-17
Q8IYM9	E3 ubiquitin-protein ligase TRIM22	0.01	1.00.E-17
Q53GG5-2	Isoform 2 of PDZ and LIM domain protein 3	0.01	1.00.E-17
Q14CB8-3	Isoform 3 of Rho GTPase-activating protein 19	0.01	1.00.E-17
Q9H2H8	Peptidyl-prolyl cis-trans isomerase-like 3	0.01	1.00.E-17
Q8NDV7-6	Isoform 6 of Trinucleotide repeat-containing gene 6A protein	0.01	1.00.E-17
P45983-4	Isoform 4 of Mitogen-activated protein kinase 8	0.01	1.00.E-17
Q92540	Protein SMG7	0.01	1.00.E-17
Q9Y2X0-3	Isoform 3 of Mediator of RNA polymerase II transcription subunit 16	0.01	1.00.E-17
Q9UJQ4-2	Isoform SALL4B of Sal-like protein 4	0.01	1.00.E-17
P21802-2	Isoform 2 of Fibroblast growth factor receptor 2	0.01	1.00.E-17
P22607-4	Isoform 4 of Fibroblast growth factor receptor 3	0.01	1.00.E-17
Q9H4L5-7	Isoform 2c of Oxysterol-binding protein-related protein 3	0.01	1.00.E-17
P22607	Fibroblast growth factor receptor 3	0.01	1.00.E-17
Q14678-2	Isoform 2 of KN motif and ankyrin repeat domain-containing protein 1	0.01	1.00.E-17
Q92759-2	Isoform 2 of General transcription factor IIH subunit 4	0.01	1.00.E-17

Q9P0N9	TBC1 domain family member 7	0.01	1.00.E-17
Q9Y5X3-2	Isoform 2 of Sorting nexin-5	0.01	1.00.E-17
Q9Y3Q8-2	Isoform 2 of TSC22 domain family protein 4	0.01	1.00.E-17
P29350-2	Isoform 3 of Tyrosine-protein phosphatase non-receptor type 6	0.01	1.00.E-17
Q14CB8	Rho GTPase-activating protein 19	0.01	1.00.E-17
Q9H6R3-2	Isoform 2 of Acyl-CoA synthetase short-chain family member 3, mitochondrial	0.01	1.00.E-17
Q5VZK9-2	Isoform 2 of F-actin-uncapping protein LRRC16A	0.01	1.00.E-17
Q14192-2	Isoform 2 of Four and a half LIM domains protein 2	0.01	1.00.E-17
Q8N4C6-4	Isoform 4 of Ninein	0.01	1.00.E-17
P33316-2	Isoform 2 of Deoxyuridine 5'-triphosphate nucleotidohydrolase, mitochondrial	0.01	1.00.E-17
Q13277-3	Isoform 3 of Syntaxin-3	0.01	1.00.E-17
Q96II8-2	Isoform 2 of DISP complex protein LRCH3	0.01	1.00.E-17
Q6YHU6-5	Isoform 5 of Thyroid adenoma-associated protein	0.01	1.00.E-17
P20585	DNA mismatch repair protein Msh3	0.01	1.00.E-17
Q9NUL7	Probable ATP-dependent RNA helicase DDX28	0.01	1.00.E-17
P18440	Arylamine N-acetyltransferase 1	0.01	1.00.E-17
P14902	Indoleamine 2,3-dioxygenase 1	0.01	1.00.E-17
Q9ULU4-15	Isoform 15 of Protein kinase C-binding protein 1	0.01	1.00.E-17
P49848	Transcription initiation factor TFIID subunit 6	0.01	1.00.E-17
Q9HCN4	GPN-loop GTPase 1	0.01	1.00.E-17
Q96RT1-8	Isoform 8 of Erbin	0.01	1.00.E-17
Q68EM7-2	Isoform 2 of Rho GTPase-activating protein 17	0.01	1.00.E-17
O75691	Small subunit processome component 20 homolog	0.01	1.00.E-17
Q96SZ4-3	Isoform 3 of Zinc finger and SCAN domain-containing protein 10	0.01	1.00.E-17
Q8N5I9	Uncharacterized protein C12orf45	0.01	1.00.E-17
Q9BST9-2	Isoform 2 of Rhotekin	0.01	1.00.E-17
Q9ULU4-10	Isoform 10 of Protein kinase C-binding protein 1	0.01	1.00.E-17
Q9ULU4-9	Isoform 9 of Protein kinase C-binding protein 1	0.01	1.00.E-17
P56270	Myc-associated zinc finger protein	0.01	1.00.E-17
O60885	Bromodomain-containing protein 4	0.01	1.00.E-17
Q96AD5-2	Isoform 2 of Patatin-like phospholipase domain-containing protein 2	0.01	1.00.E-17
P29350-4	Isoform 4 of Tyrosine-protein phosphatase non-receptor type 6	0.01	1.00.E-17
A5YM69	Rho guanine nucleotide exchange factor 35	0.01	1.00.E-17
Q9UMX1-2	Isoform 2 of Suppressor of fused homolog	0.01	1.00.E-17
Q9UIG0-2	Isoform 2 of Tyrosine-protein kinase BAZ1B	0.01	1.00.E-17
O15287	Fanconi anemia group G protein	0.01	1.00.E-17
Q9NQ55-2	Isoform 2 of Suppressor of SWI4 1 homolog	0.04	1.48.E-02
P49848-4	Isoform 4 of Transcription initiation factor TFIID subunit 6	0.01	1.00.E-17
Q6VN20	Ran-binding protein 10	0.01	1.00.E-17
O43741-2	Isoform 2 of 5'-AMP-activated protein kinase subunit beta-2	0.01	1.00.E-17
Q8WUB8	PHD finger protein 10	0.053	1.38.E-02
Q92626	Peroxidasin homolog	0.01	1.00.E-17
P15884-9	Isoform D- of Transcription factor 4	0.01	1.00.E-17
Q5F1R6-3	Isoform 3 of DnaJ homolog subfamily C member 21	0.01	1.00.E-17
P98173-3	Isoform 3 of Protein FAM3A	0.01	1.00.E-17
Q6VN20-2	Isoform 2 of Ran-binding protein 10	0.01	1.00.E-17
Q9Y3B8-3	Isoform 3 of Oligoribonuclease, mitochondrial	0.01	1.00.E-17
Q8N9M5	Transmembrane protein 102	0.01	1.00.E-17
P48594	Serpin B4	0.01	1.00.E-17
P09493-2	Isoform 2 of Tropomyosin alpha-1 chain	0.012	4.24.E-05
Q14CB8-7	Isoform 7 of Rho GTPase-activating protein 19	0.01	1.00.E-17
Q9Y6X3	MAU2 chromatid cohesion factor homolog	0.01	1.00.E-17
Q92540-4	Isoform 4 of Protein SMG7	0.01	1.00.E-17
Q9NVQ4	Fas apoptotic inhibitory molecule 1	0.01	1.00.E-17
Q13574-1	Isoform 2 of Diacylglycerol kinase zeta	0.01	1.00.E-17
P24844-2	Isoform 2 of Myosin regulatory light polypeptide 9	0.01	1.00.E-17
Q9NRR3	CDC42 small effector protein 2	0.01	1.00.E-17
O00762-4	Isoform 4 of Ubiquitin-conjugating enzyme E2 C	0.01	1.00.E-17
Q9UIU6	Homeobox protein SIX4	0.01	1.00.E-17
Q8N5C6-2	Isoform 2 of S1 RNA-binding domain-containing protein 1	0.01	1.00.E-17
Q96QD8	Sodium-coupled neutral amino acid transporter 2	0.018	2.84.E-03
O94808	Glutamine--fructose-6-phosphate aminotransferase [isomerizing] 2	0.065	3.51.E-02
Q9Y221	60S ribosome subunit biogenesis protein NIP7 homolog	0.01	1.00.E-17
Q96RT1-5	Isoform 5 of Erbin	0.01	1.00.E-17
Q9NS87-3	Isoform 3 of Kinesin-like protein KIF15	0.01	1.00.E-17
Q16875-2	Isoform 2 of 6-phosphofructo-2-kinase/fructose-2,6-bisphosphatase 3	0.01	1.00.E-17

Q13574-7	Isoform 7 of Diacylglycerol kinase zeta	0.01	1.00.E-17
Q9Y485	DmX-like protein 1	0.01	1.00.E-17
Q13888	General transcription factor IIH subunit 2	0.01	1.00.E-17
Q9Y5Y0-2	Isoform 2 of Feline leukemia virus subgroup C receptor-related protein 1	0.01	1.00.E-17
Q8NFU3	Thiosulfate:glutathione sulfurtransferase	0.01	1.00.E-17
O14578	Citron Rho-interacting kinase	0.01	1.00.E-17
P56270-3	Isoform 3 of Myc-associated zinc finger protein	0.01	1.00.E-17
O75970-5	Isoform 4 of Multiple PDZ domain protein	0.01	1.00.E-17
Q96GD4-4	Isoform 4 of Aurora kinase B	0.01	1.00.E-17
Q99569	Plakophilin-4	0.01	1.00.E-17
Q86TS9	39S ribosomal protein L52, mitochondrial	0.01	1.00.E-17
Q12965	Unconventional myosin-Ie	0.01	1.00.E-17
Q9NSP4-3	Isoform 3 of Centromere protein M	0.01	1.00.E-17
Q8NFU3-3	Isoform 3 of Thiosulfate:glutathione sulfurtransferase	0.01	1.00.E-17
Q9UBN4-6	Isoform Zeta of Short transient receptor potential channel 4	0.01	1.00.E-17
Q92759	General transcription factor IIH subunit 4	0.01	1.00.E-17
Q68EM7-4	Isoform 4 of Rho GTPase-activating protein 17	0.01	1.00.E-17
Q9ULU4-6	Isoform 6 of Protein kinase C-binding protein 1	0.01	1.00.E-17
P12110-2	Isoform 2C2A of Collagen alpha-2(VI) chain	0.01	1.00.E-17
P56270-4	Isoform 4 of Myc-associated zinc finger protein	0.01	1.00.E-17
Q86XZ4	Spermatogenesis-associated serine-rich protein 2	0.01	1.00.E-17
Q9BST9	Rhotekin	0.01	1.00.E-17
Q9BRP4	Proteasomal ATPase-associated factor 1	0.01	1.00.E-17
Q8N108-19	Isoform 9 of Mesoderm induction early response protein 1	0.01	1.00.E-17
Q14451-4	Isoform 4 of Growth factor receptor-bound protein 7	0.01	1.00.E-17
P49848-3	Isoform 3 of Transcription initiation factor TFIID subunit 6	0.01	1.00.E-17
Q9UET6-2	Isoform 2 of Putative tRNA (cytidine(32)/guanosine(34)-2'-O)-methyltransferase	0.01	1.00.E-17
Q9HCK8	Chromodomain-helicase-DNA-binding protein 8	0.01	1.00.E-17
Q99959-2	Isoform 1 of Plakophilin-2	0.01	1.00.E-17
P85298-4	Isoform 4 of Rho GTPase-activating protein 8	0.01	1.00.E-17
Q8NFM7-3	Isoform 3 of Interleukin-17 receptor D	0.01	1.00.E-17
Q96IQ9-2	Isoform 2 of Zinc finger protein 414	0.01	1.00.E-17
P35900	Keratin, type I cytoskeletal 20	0.058	3.74.E-02
Q86YL5	Testis development-related protein	0.01	1.00.E-17
Q86TS9-2	Isoform 2 of 39S ribosomal protein L52, mitochondrial	0.01	1.00.E-17
Q8NDV7-5	Isoform 5 of Trinucleotide repeat-containing gene 6A protein	0.01	1.00.E-17
Q5F1R6	DnaJ homolog subfamily C member 21	0.01	1.00.E-17
Q9NPA3	Mid1-interacting protein 1	0.01	1.00.E-17
Q9BXS6-6	Isoform 6 of Nucleolar and spindle-associated protein 1	0.01	1.00.E-17
Q6ZRY4	RNA-binding protein with multiple splicing 2	0.046	2.94.E-02
Q92636	Protein FAN	0.01	1.00.E-17
P50548-2	Isoform 2 of ETS domain-containing transcription factor ERF	0.01	1.00.E-17
Q9H6S3-3	Isoform 3 of Epidermal growth factor receptor kinase substrate 8-like protein 2	0.01	1.00.E-17
Q06416	Putative POU domain, class 5, transcription factor 1B	0.01	1.00.E-17
O95453	Poly(A)-specific ribonuclease PARN	0.086	4.93.E-02
P41134	DNA-binding protein inhibitor ID-1	0.01	1.00.E-17
Q9NR33	DNA polymerase epsilon subunit 4	0.01	1.00.E-17
P20839-3	Isoform 3 of Inosine-5'-monophosphate dehydrogenase 1	0.01	1.00.E-17
O60287	Nucleolar pre-ribosomal-associated protein 1	0.01	1.00.E-17
Q9UHI5	Large neutral amino acids transporter small subunit 2	0.01	1.00.E-17
Q01860	POU domain, class 5, transcription factor 1	0.01	1.00.E-17
Q96T23-2	Isoform 2 of Remodeling and spacing factor 1	0.01	1.00.E-17
Q9ULU4-21	Isoform 21 of Protein kinase C-binding protein 1	0.01	1.00.E-17
P04818-2	Isoform 2 of Thymidylate synthase	0.03	1.03.E-02
Q14244-7	Isoform 7 of Ensconsin	0.01	1.00.E-17
P24844	Myosin regulatory light polypeptide 9	0.01	1.00.E-17
Q9BXS6	Nucleolar and spindle-associated protein 1	0.01	1.00.E-17
Q9NW08	DNA-directed RNA polymerase III subunit RPC2	0.051	1.18.E-02
P20839-5	Isoform 5 of Inosine-5'-monophosphate dehydrogenase 1	0.01	1.00.E-17
Q96LB3	Intraflagellar transport protein 74 homolog	0.01	1.00.E-17
Q9BTK6	PAXIP1-associated glutamate-rich protein 1	0.01	1.00.E-17
Q4LE39-3	Isoform 3 of AT-rich interactive domain-containing protein 4B	0.01	1.00.E-17
Q96PV7-3	Isoform 3 of Protein FAM193B	0.01	1.00.E-17
Q9NVM4-4	Isoform 4 of Protein arginine N-methyltransferase 7	0.01	1.00.E-17
Q969X6-2	Isoform 2 of U3 small nucleolar RNA-associated protein 4 homolog	0.01	1.00.E-17
P82970	High mobility group nucleosome-binding domain-containing protein 5	0.01	1.00.E-17

Q96SZ6	Mitochondrial tRNA methylthiotransferase CDK5RAP1	0.01	1.00.E-17
P12110-3	Isoform 2C2A' of Collagen alpha-2(VI) chain	0.01	1.00.E-17
A9QM74	Importin subunit alpha-8	0.01	1.00.E-17
Q9UBR2	Cathepsin Z	0.01	1.00.E-17
P21709-2	Isoform 2 of Ephrin type-A receptor 1	0.01	1.00.E-17
Q9ULU4-22	Isoform 22 of Protein kinase C-binding protein 1	0.01	1.00.E-17
Q92925	SWI/SNF-related matrix-associated actin-dependent regulator of chromatin subfamily D member 2	0.01	1.00.E-17
Q15057	Arf-GAP with coiled-coil, ANK repeat and PH domain-containing protein 2	0.01	1.00.E-17
P58340-2	Isoform 2 of Myeloid leukemia factor 1	0.01	1.00.E-17
Q71DI3	Histone H3.2	0.01	1.00.E-17
Q9H7E9-2	Isoform 2 of UPF0488 protein C8orf33	0.01	1.00.E-17
Q9NRG0	Chromatin accessibility complex protein 1	0.01	1.00.E-17
Q96FC9-2	Isoform 2 of ATP-dependent DNA helicase DDX11	0.01	1.00.E-17
Q14676-4	Isoform 4 of Mediator of DNA damage checkpoint protein 1	0.05	2.46.E-02
Q9Y219	Protein jagged-2	0.01	1.00.E-17
Q86V48	Leucine zipper protein 1	0.01	1.00.E-17
Q15746	Myosin light chain kinase, smooth muscle	0.01	1.00.E-17
Q5VSY0-2	Isoform 2 of G kinase-anchoring protein 1	0.01	1.00.E-17
Q5TEJ8	Protein THEMIS2	0.01	1.00.E-17
Q68EM7-5	Isoform 5 of Rho GTPase-activating protein 17	0.01	1.00.E-17
O95379-4	Isoform 4 of Tumor necrosis factor alpha-induced protein 8	0.041	9.58.E-03
Q13574-6	Isoform 6 of Diacylglycerol kinase zeta	0.01	1.00.E-17
Q8IVW6-4	Isoform 4 of AT-rich interactive domain-containing protein 3B	0.01	1.00.E-17
Q15746-3	Isoform 3A of Myosin light chain kinase, smooth muscle	0.01	1.00.E-17
Q9ULU4-4	Isoform 4 of Protein kinase C-binding protein 1	0.01	1.00.E-17
Q13574	Diacylglycerol kinase zeta	0.01	1.00.E-17
Q92833-2	Isoform 2 of Protein Jumonji	0.01	1.00.E-17
P52630	Signal transducer and activator of transcription 2	0.01	1.00.E-17
Q14CB8-6	Isoform 6 of Rho GTPase-activating protein 19	0.01	1.00.E-17
Q9BXW9-4	Isoform 4 of Fanconi anemia group D2 protein	0.01	1.00.E-17
Q96NW4	Ankyrin repeat domain-containing protein 27	0.01	1.00.E-17
Q14244-3	Isoform 3 of Ensconsin	0.01	1.00.E-17
P21802-17	Isoform 11 of Fibroblast growth factor receptor 2	0.01	1.00.E-17
Q05513-3	Isoform 3 of Protein kinase C zeta type	0.056	2.69.E-02
Q96AT1	Uncharacterized protein KIAA1143	0.042	1.46.E-02
Q8IWD4	Coiled-coil domain-containing protein 117	0.01	1.00.E-17
Q8N4C6-7	Isoform 7 of Ninein	0.01	1.00.E-17
Q8NFU3-2	Isoform 2 of Thiosulfate:glutathione sulfurtransferase	0.01	1.00.E-17
Q5VZ89-3	Isoform 3 of DENN domain-containing protein 4C	0.01	1.00.E-17
O95273-2	Isoform 2 of Cyclin-D1-binding protein 1	0.01	1.00.E-17
Q9Y651	Transcription factor SOX-21	0.01	1.00.E-17
Q9BTX1-4	Isoform 4 of Nucleoporin NDC1	0.01	1.00.E-17
Q53GG5	PDZ and LIM domain protein 3	0.01	1.00.E-17
Q8N4C6-11	Isoform 9 of Ninein	0.01	1.00.E-17
O15155-2	Isoform 2 of BET1 homolog	0.01	1.00.E-17
Q14145	Kelch-like ECH-associated protein 1	0.01	1.00.E-17
Q13111	Chromatin assembly factor 1 subunit A	0.01	1.00.E-17
Q8N108-17	Isoform 7 of Mesoderm induction early response protein 1	0.01	1.00.E-17
Q5VSY0	G kinase-anchoring protein 1	0.01	1.00.E-17
Q5T5X7	BEN domain-containing protein 3	0.01	1.00.E-17
Q9H6U8-2	Isoform 2 of Alpha-1,2-mannosyltransferase ALG9	0.01	1.00.E-17
Q7L190	Developmental pluripotency-associated protein 4	0.01	1.00.E-17
Q9UBU8	Mortality factor 4-like protein 1	0.01	1.00.E-17
Q9UP95-3	Isoform 3 of Solute carrier family 12 member 4	0.01	1.00.E-17
Q9BXS9-7	Isoform 7 of Solute carrier family 26 member 6	0.01	1.00.E-17
Q8N4C6	Ninein	0.01	1.00.E-17
P15884-8	Isoform H- of Transcription factor 4	0.01	1.00.E-17
Q9BUL5-2	Isoform 2 of PHD finger protein 23	0.01	1.00.E-17
Q9UKK9	ADP-sugar pyrophosphatase	0.01	1.00.E-17
Q15746-6	Isoform Del-1790 of Myosin light chain kinase, smooth muscle	0.01	1.00.E-17
Q86YL5-2	Isoform 2 of Testis development-related protein	0.01	1.00.E-17
Q9UBU8-2	Isoform 2 of Mortality factor 4-like protein 1	0.01	1.00.E-17
Q9BZX2-2	Isoform 2 of Uridine-cytidine kinase 2	0.01	1.00.E-17
Q96SZ4	Zinc finger and SCAN domain-containing protein 10	0.01	1.00.E-17
Q5VZL5-2	Isoform 2 of Zinc finger MYM-type protein 4	0.01	1.00.E-17

Q15054	DNA polymerase delta subunit 3	0.01	1.00.E-17
Q8TBB5-2	Isoform 2 of Kelch domain-containing protein 4	0.01	1.00.E-17
E9PRG8	Uncharacterized protein C11orf98	0.01	1.00.E-17
Q7Z7K6	Centromere protein V	0.01	1.00.E-17
Q96QT6-3	Isoform 3 of PHD finger protein 12	0.01	1.00.E-17
Q9NQS7	Inner centromere protein	0.01	1.00.E-17
Q9BXS9-6	Isoform 6 of Solute carrier family 26 member 6	0.01	1.00.E-17
O60291-3	Isoform 3 of E3 ubiquitin-protein ligase MGRN1	0.01	1.00.E-17
P57076	Cilia- and flagella-associated protein 298	0.01	1.00.E-17
Q8NFU3-4	Isoform 4 of Thiosulfate:glutathione sulfurtransferase	0.01	1.00.E-17
P21802-16	Isoform 10 of Fibroblast growth factor receptor 2	0.01	1.00.E-17
Q9H4L5-3	Isoform 1c of Oxysterol-binding protein-related protein 3	0.01	1.00.E-17
Q8WXD5	Gem-associated protein 6	0.01	1.00.E-17
P50548	ETS domain-containing transcription factor ERF	0.01	1.00.E-17
Q9NRG1-2	Isoform 2 of Phosphoribosyltransferase domain-containing protein 1	0.01	1.00.E-17
Q969K3	E3 ubiquitin-protein ligase RNF34	0.01	1.00.E-17
Q9BVJ6-3	Isoform 3 of U3 small nucleolar RNA-associated protein 14 homolog A	0.01	1.00.E-17
Q9BXS9	Solute carrier family 26 member 6	0.01	1.00.E-17
Q6YHU6-3	Isoform 3 of Thyroid adenoma-associated protein	0.01	1.00.E-17
Q99986	Serine/threonine-protein kinase VRK1	0.01	1.00.E-17
Q6IQ49	Replication stress response regulator SDE2	0.01	1.00.E-17
Q9HCN4-2	Isoform 2 of GPN-loop GTPase 1	0.01	1.00.E-17
Q9P0N9-4	Isoform 4 of TBC1 domain family member 7	0.01	1.00.E-17
Q63HN8	E3 ubiquitin-protein ligase RNF213	0.01	1.00.E-17
Q6P1K8	General transcription factor IIH subunit 2-like protein	0.01	1.00.E-17
P21802-18	Isoform 12 of Fibroblast growth factor receptor 2	0.01	1.00.E-17
Q9ULU4-19	Isoform 19 of Protein kinase C-binding protein 1	0.01	1.00.E-17
P32243-2	Isoform 2 of Homeobox protein OTX2	0.01	1.00.E-17
Q6GQQ9	OTU domain-containing protein 7B	0.072	3.81.E-02
Q6IQ49-3	Isoform 3 of Replication stress response regulator SDE2	0.01	1.00.E-17
Q9ULU4-3	Isoform 3 of Protein kinase C-binding protein 1	0.01	1.00.E-17
O14578-3	Isoform 3 of Citron Rho-interacting kinase	0.01	1.00.E-17
P15884-7	Isoform G- of Transcription factor 4	0.01	1.00.E-17
P15884-4	Isoform B+delta of Transcription factor 4	0.01	1.00.E-17
P82094	TATA element modulatory factor	0.01	1.00.E-17
Q9UJX3	Anaphase-promoting complex subunit 7	0.01	1.00.E-17
O43278-2	Isoform 2 of Kunitz-type protease inhibitor 1	0.01	1.00.E-17
Q9H9Y6-2	Isoform 2 of DNA-directed RNA polymerase I subunit RPA2	0.025	1.89.E-03
P26374	Rab proteins geranylgeranyltransferase component A 2	0.01	1.00.E-17
Q9NRZ9-7	Isoform 7 of Lymphoid-specific helicase	0.01	1.00.E-17
Q6P1Q9-2	Isoform 2 of tRNA N(3)-methylcytidine methyltransferase METTL2B	0.01	1.00.E-17
Q96EV2	RNA-binding protein 33	0.01	1.00.E-17
Q9H6U8-4	Isoform 4 of Alpha-1,2-mannosyltransferase ALG9	0.01	1.00.E-17
O75127	Pentatricopeptide repeat-containing protein 1, mitochondrial	0.01	1.00.E-17
Q13309-4	Isoform 3 of S-phase kinase-associated protein 2	0.01	1.00.E-17
Q96SZ6-6	Isoform 6 of Mitochondrial tRNA methylthiotransferase CDK5RAP1	0.01	1.00.E-17
Q9BU76-3	Isoform 3 of Multiple myeloma tumor-associated protein 2	0.01	1.00.E-17
P30307	M-phase inducer phosphatase 3	0.01	1.00.E-17
Q16875-4	Isoform 4 of 6-phosphofructo-2-kinase/fructose-2,6-bisphosphatase 3	0.01	1.00.E-17
P85298-2	Isoform 2 of Rho GTPase-activating protein 8	0.01	1.00.E-17
Q9ULU4-17	Isoform 17 of Protein kinase C-binding protein 1	0.01	1.00.E-17
Q9NQW6	Anillin	0.01	1.00.E-17
P55211	Caspase-9	0.01	1.00.E-17
O75448	Mediator of RNA polymerase II transcription subunit 24	0.01	1.00.E-17
Q13643	Four and a half LIM domains protein 3	0.01	1.00.E-17
Q6N069	N-alpha-acetyltransferase 16, NatA auxiliary subunit	0.01	1.00.E-17
P36551-2	Isoform 2 of Oxygen-dependent coproporphyrinogen-III oxidase, mitochondrial	0.01	1.00.E-17
Q9H7E9	UPF0488 protein C8orf33	0.01	1.00.E-17
Q96L91-5	Isoform 5 of E1A-binding protein p400	0.01	1.00.E-17
Q9NVH2-2	Isoform 2 of Integrator complex subunit 7	0.01	1.00.E-17
Q96PV7	Protein FAM193B	0.01	1.00.E-17
Q96DR4	StAR-related lipid transfer protein 4	0.01	1.00.E-17
Q9UNX4	WD repeat-containing protein 3	0.01	1.00.E-17
Q8N4C6-2	Isoform 2 of Ninein	0.01	1.00.E-17
Q5JSJ4	Integrator complex subunit 6-like	0.01	1.00.E-17
Q96SZ6-2	Isoform 2 of Mitochondrial tRNA methylthiotransferase CDK5RAP1	0.01	1.00.E-17

Q14137-2	Isoform 2 of Ribosome biogenesis protein BOP1	0.01	1.00.E-17
P15884-2	Isoform SEF2-1A of Transcription factor 4	0.01	1.00.E-17
Q9H078-4	Isoform 4 of Caseinolytic peptidase B protein homolog	0.01	1.00.E-17
O95453-2	Isoform 2 of Poly(A)-specific ribonuclease PARN	0.086	4.93.E-02
P04233	HLA class II histocompatibility antigen gamma chain	0.01	1.00.E-17
Q9H426	Regulating synaptic membrane exocytosis protein 4	0.01	1.00.E-17
P21709	Ephrin type-A receptor 1	0.01	1.00.E-17
O00311	Cell division cycle 7-related protein kinase	0.01	1.00.E-17
Q8N108-16	Isoform 6 of Mesoderm induction early response protein 1	0.01	1.00.E-17
A8MPP1	Putative ATP-dependent RNA helicase DDX11-like protein 8	0.01	1.00.E-17
Q9NRW3	DNA dC->dU-editing enzyme APOBEC-3C	0.069	2.97.E-02
Q8N4C6-9	Isoform 8 of Ninein	0.01	1.00.E-17
Q8WW01	tRNA-splicing endonuclease subunit Sen15	0.01	1.00.E-17
Q8WXX5	DnaJ homolog subfamily C member 9	0.01	1.00.E-17
Q96L91	E1A-binding protein p400	0.01	1.00.E-17
O15230	Laminin subunit alpha-5	0.071	3.28.E-02
Q8NFJ9-2	Isoform 3 of Bardet-Biedl syndrome 1 protein	0.01	1.00.E-17
Q96II8-3	Isoform 3 of DISP complex protein LRCH3	0.01	1.00.E-17
Q6GQQ9-2	Isoform 2 of OTU domain-containing protein 7B	0.072	3.81.E-02
Q7Z3T8-3	Isoform 2 of Zinc finger FYVE domain-containing protein 16	0.01	1.00.E-17
Q92771	Putative ATP-dependent RNA helicase DDX12	0.01	1.00.E-17
Q96RT1-6	Isoform 6 of Erbin	0.01	1.00.E-17
Q15910-2	Isoform 2 of Histone-lysine N-methyltransferase EZH2	0.01	1.00.E-17
Q9H078	Caseinolytic peptidase B protein homolog	0.01	1.00.E-17
Q15652-3	Isoform 3 of Probable JmjC domain-containing histone demethylation protein 2C	0.01	1.00.E-17
Q9BTX1-5	Isoform 5 of Nucleoporin NDC1	0.01	1.00.E-17
Q8TBB5	Kelch domain-containing protein 4	0.01	1.00.E-17
O14493	Claudin-4	0.01	1.00.E-17
Q8TE68	Epidermal growth factor receptor kinase substrate 8-like protein 1	0.01	1.00.E-17
P58340	Myeloid leukemia factor 1	0.01	1.00.E-17
Q8NI60	Atypical kinase COQ8A, mitochondrial	0.01	1.00.E-17
Q9Y3E5	Peptidyl-tRNA hydrolase 2, mitochondrial	0.01	1.00.E-17
Q9BXS6-4	Isoform 4 of Nucleolar and spindle-associated protein 1	0.01	1.00.E-17
Q9Y3V2	RWD domain-containing protein 3	0.01	1.00.E-17
Q99417	c-Myc-binding protein	0.01	1.00.E-17
Q96EV2-2	Isoform 2 of RNA-binding protein 33	0.01	1.00.E-17
Q8WUB8-2	Isoform 2 of PHD finger protein 10	0.053	1.38.E-02
P40967-3	Isoform 3 of Melanocyte protein PMEL	0.01	1.00.E-17
Q2KHR3	Glutamine and serine-rich protein 1	0.01	1.00.E-17
Q9P219	Protein Daple	0.01	1.00.E-17
Q8TE68-3	Isoform 3 of Epidermal growth factor receptor kinase substrate 8-like protein 1	0.01	1.00.E-17
Q6N069-2	Isoform 2 of N-alpha-acetyltransferase 16, NatA auxiliary subunit	0.01	1.00.E-17
Q6PGN9	Proline/serine-rich coiled-coil protein 1	0.01	1.00.E-17
P0CG29	Glutathione S-transferase theta-2	0.01	1.00.E-17
P04818-3	Isoform 3 of Thymidylate synthase	0.01	1.00.E-17
Q96QD8-2	Isoform 2 of Sodium-coupled neutral amino acid transporter 2	0.018	3.42.E-03
Q9H2S1	Small conductance calcium-activated potassium channel protein 2	0.01	1.00.E-17
Q9UBP6	tRNA (guanine-N(7)-)-methyltransferase	0.01	1.00.E-17
Q9H4L5	Oxysterol-binding protein-related protein 3	0.01	1.00.E-17
Q9ULU4-8	Isoform 8 of Protein kinase C-binding protein 1	0.01	1.00.E-17
Q9GZP9	Derlin-2	0.01	1.00.E-17
O15551	Claudin-3	0.01	1.00.E-17
P57081-2	Isoform 2 of tRNA (guanine-N(7)-)-methyltransferase non-catalytic subunit WDR4	0.01	1.00.E-17
Q9UHP3-1	Isoform USP25b of Ubiquitin carboxyl-terminal hydrolase 25	0.01	1.00.E-17
P04233-3	Isoform 3 of HLA class II histocompatibility antigen gamma chain	0.01	1.00.E-17
P21802	Fibroblast growth factor receptor 2	0.01	1.00.E-17
P21802-6	Isoform 6 of Fibroblast growth factor receptor 2	0.01	1.00.E-17
Q16342-3	Isoform 3 of Programmed cell death protein 2	0.01	1.00.E-17
P30307-4	Isoform 5 of M-phase inducer phosphatase 3	0.01	1.00.E-17
Q3L8U1-2	Isoform 2 of Chromodomain-helicase-DNA-binding protein 9	0.01	1.00.E-17
Q9H6S3-2	Isoform 2 of Epidermal growth factor receptor kinase substrate 8-like protein 2	0.01	1.00.E-17
Q5VZL5-3	Isoform 3 of Zinc finger MYM-type protein 4	0.01	1.00.E-17
Q9NX24	H/ACA ribonucleoprotein complex subunit 2	0.01	1.00.E-17
Q9H078-2	Isoform 2 of Caseinolytic peptidase B protein homolog	0.01	1.00.E-17
Q99569-2	Isoform 2 of Plakophilin-4	0.01	1.00.E-17
Q9UL03-3	Isoform 3 of Integrator complex subunit 6	0.01	1.00.E-17

Q9NP50	SIN3-HDAC complex-associated factor	0.01	1.00.E-17
Q9UJ83	2-hydroxyacyl-CoA lyase 1	0.01	1.00.E-17
Q96FC9-5	Isoform 5 of ATP-dependent DNA helicase DDX11	0.01	1.00.E-17
Q6NW34	Nucleolus and neural progenitor protein	0.01	1.00.E-17
Q68EM7	Rho GTPase-activating protein 17	0.01	1.00.E-17
Q8WYA0-4	Isoform CDV-1 of Intraflagellar transport protein 81 homolog	0.01	1.00.E-17
Q9UHP3	Ubiquitin carboxyl-terminal hydrolase 25	0.01	1.00.E-17
Q9NQ55	Suppressor of SWI4 1 homolog	0.04	1.48.E-02
Q969X6	U3 small nucleolar RNA-associated protein 4 homolog	0.01	1.00.E-17
Q6N069-5	Isoform 5 of N-alpha-acetyltransferase 16, NatA auxiliary subunit	0.01	1.00.E-17
Q96GD4	Aurora kinase B	0.01	1.00.E-17
Q9P0N9-3	Isoform 3 of TBC1 domain family member 7	0.01	1.00.E-17
Q96RT1-4	Isoform 4 of Erbin	0.01	1.00.E-17
Q9GZV8	PR domain zinc finger protein 14	0.01	1.00.E-17
Q9UHI5-4	Isoform 4 of Large neutral amino acids transporter small subunit 2	0.01	1.00.E-17
Q6XZF7-2	Isoform 2 of Dynamin-binding protein	0.01	1.00.E-17
Q9H6R4-3	Isoform 3 of Nucleolar protein 6	0.01	1.00.E-17
Q14202-2	Isoform 2 of Zinc finger MYM-type protein 3	0.01	1.00.E-17
Q9BXS6-5	Isoform 5 of Nucleolar and spindle-associated protein 1	0.01	1.00.E-17
O75717-2	Isoform 2 of WD repeat and HMG-box DNA-binding protein 1	0.06	2.48.E-02
O43818	U3 small nucleolar RNA-interacting protein 2	0.01	1.00.E-17
Q969K3-2	Isoform 2 of E3 ubiquitin-protein ligase RNF34	0.01	1.00.E-17
O00160	Unconventional myosin-If	0.01	1.00.E-17
Q13112	Chromatin assembly factor 1 subunit B	0.01	1.00.E-17
Q9Y5Y6	Suppressor of tumorigenicity 14 protein	0.01	1.00.E-17
Q9BXS9-2	Isoform 2 of Solute carrier family 26 member 6	0.01	1.00.E-17
Q8N4C6-5	Isoform 5 of Ninein	0.01	1.00.E-17
Q9BZA5	Putative gamma-taxilin 2	0.01	1.00.E-17
Q9BXF3-2	Isoform B of Cat eye syndrome critical region protein 2	0.01	1.00.E-17
Q9H361	Polyadenylate-binding protein 3	0.01	1.00.E-17
Q63HN8-4	Isoform 2 of E3 ubiquitin-protein ligase RNF213	0.01	1.00.E-17
P06703	Protein S100-A6	0.037	4.58.E-03
Q96QT6-5	Isoform 5 of PHD finger protein 12	0.01	1.00.E-17
Q9UP95-2	Isoform 2 of Solute carrier family 12 member 4	0.01	1.00.E-17
Q8N4J0	Carnosine N-methyltransferase	0.01	1.00.E-17
Q96T23-3	Isoform 3 of Remodeling and spacing factor 1	0.01	1.00.E-17
Q9P0V3-2	Isoform 2 of SH3 domain-binding protein 4	0.01	1.00.E-17
Q8N4N8	Kinesin-like protein KIF2B	0.01	1.00.E-17
Q9ULU4-13	Isoform 13 of Protein kinase C-binding protein 1	0.01	1.00.E-17
Q9UII4	E3 ISG15--protein ligase HERC5	0.01	1.00.E-17
Q9NWS0-3	Isoform 3 of PIH1 domain-containing protein 1	0.01	1.00.E-17
Q6FIF0	AN1-type zinc finger protein 6	0.01	1.00.E-17
Q9BVJ6-2	Isoform 2 of U3 small nucleolar RNA-associated protein 14 homolog A	0.01	1.00.E-17
Q9GZX6	Interleukin-22	0.01	1.00.E-17
Q9H4L5-6	Isoform 2b of Oxysterol-binding protein-related protein 3	0.01	1.00.E-17
Q12834	Cell division cycle protein 20 homolog	0.01	1.00.E-17
Q96RT1	Erbin	0.01	1.00.E-17
P85298-3	Isoform 3 of Rho GTPase-activating protein 8	0.01	1.00.E-17
P09493-3	Isoform 3 of Tropomyosin alpha-1 chain	0.012	4.24.E-05
Q9BZQ8	Protein Niban 1	0.01	1.00.E-17
Q86V48-2	Isoform 2 of Leucine zipper protein 1	0.01	1.00.E-17
Q8ND30	Liprin-beta-2	0.01	1.00.E-17
Q9H0U6	39S ribosomal protein L18, mitochondrial	0.01	1.00.E-17
Q8NFM7-4	Isoform 4 of Interleukin-17 receptor D	0.01	1.00.E-17
Q6BCY4-2	Isoform 2 of NADH-cytochrome b5 reductase 2	0.047	7.85.E-03
Q9NWU5-2	Isoform 2 of 39S ribosomal protein L22, mitochondrial	0.01	1.00.E-17
Q8NFM7	Interleukin-17 receptor D	0.01	1.00.E-17
Q7Z5L2	Coiled-coil domain-containing protein R3HCC1L	0.01	1.00.E-17
Q8WU90-2	Isoform 2 of Zinc finger CCCH domain-containing protein 15	0.01	1.00.E-17
Q99576	TSC22 domain family protein 3	0.01	1.00.E-17
O43683-3	Isoform 3 of Mitotic checkpoint serine/threonine-protein kinase BUB1	0.046	2.22.E-02
P21802-5	Isoform 5 of Fibroblast growth factor receptor 2	0.01	1.00.E-17
O95379-3	Isoform 3 of Tumor necrosis factor alpha-induced protein 8	0.041	9.58.E-03
P52569	Cationic amino acid transporter 2	0.01	1.00.E-17
O14647-2	Isoform 2 of Chromodomain-helicase-DNA-binding protein 2	0.01	1.00.E-17
Q9BU61-2	Isoform b of NADH dehydrogenase [ubiquinone] 1 alpha subcomplex assembly factor 3	0.01	1.00.E-17

P09493-9	Isoform 9 of Tropomyosin alpha-1 chain	0.012	4.24.E-05
P82094-2	Isoform 2 of TATA element modulatory factor	0.01	1.00.E-17
O60291-2	Isoform 2 of E3 ubiquitin-protein ligase MGRN1	0.01	1.00.E-17
Q8NDV7-2	Isoform 2 of Trinucleotide repeat-containing gene 6A protein	0.01	1.00.E-17
Q9HCN4-3	Isoform 3 of GPN-loop GTPase 1	0.01	1.00.E-17
Q01973	Inactive tyrosine-protein kinase transmembrane receptor ROR1	0.01	1.00.E-17
Q96SI1	BTB/POZ domain-containing protein KCTD15	0.01	1.00.E-17
P09493-7	Isoform 7 of Tropomyosin alpha-1 chain	0.012	4.24.E-05
P85298	Rho GTPase-activating protein 8	0.01	1.00.E-17
Q96G01-2	Isoform 2 of Protein bicaudal D homolog 1	0.01	1.00.E-17
P15884-11	Isoform 11 of Transcription factor 4	0.01	1.00.E-17
Q9NQ55-3	Isoform 3 of Suppressor of SWI4 1 homolog	0.04	1.48.E-02
Q9H6Y2-2	Isoform 2 of WD repeat-containing protein 55	0.01	1.00.E-17
Q96FC9-3	Isoform 3 of ATP-dependent DNA helicase DDX11	0.01	1.00.E-17
Q8NDV7	Trinucleotide repeat-containing gene 6A protein	0.01	1.00.E-17
P32243	Homeobox protein OTX2	0.01	1.00.E-17
Q13472-2	Isoform Short of DNA topoisomerase 3-alpha	0.01	1.00.E-17
O95816	BAG family molecular chaperone regulator 2	0.01	1.00.E-17
Q15746-5	Isoform 4 of Myosin light chain kinase, smooth muscle	0.01	1.00.E-17
P17931	Galectin-3	0.01	1.00.E-17
Q9NVH2-4	Isoform 4 of Integrator complex subunit 7	0.01	1.00.E-17
Q15652	Probable JmjC domain-containing histone demethylation protein 2C	0.01	1.00.E-17
Q9H9Y6-5	Isoform 5 of DNA-directed RNA polymerase I subunit RPA2	0.025	1.89.E-03
Q12816	Trophinin	0.01	1.00.E-17
Q8TE73	Dynein heavy chain 5, axonemal	0.01	1.00.E-17
P50993	Sodium/potassium-transporting ATPase subunit alpha-2	0.01	1.00.E-17
Q96D96	Voltage-gated hydrogen channel 1	0.01	1.00.E-17
Q6VN20-3	Isoform 3 of Ran-binding protein 10	0.01	1.00.E-17
P04233-2	Isoform 2 of HLA class II histocompatibility antigen gamma chain	0.01	1.00.E-17
Q9BWG4	Single-stranded DNA-binding protein 4	0.01	1.00.E-17
P49641-2	Isoform 2 of Alpha-mannosidase 2x	0.01	1.00.E-17
Q5F1R6-2	Isoform 2 of DnaJ homolog subfamily C member 21	0.01	1.00.E-17
Q9H4L5-4	Isoform 1d of Oxysterol-binding protein-related protein 3	0.01	1.00.E-17
Q15742-3	Isoform 3 of NGFI-A-binding protein 2	0.01	1.00.E-17
Q5VZ89-7	Isoform 2 of DENN domain-containing protein 4C	0.01	1.00.E-17
P08123	Collagen alpha-2(I) chain	0.01	1.00.E-17
P84101	Small EDRK-rich factor 2	0.01	1.00.E-17
Q96GK7	Fumarylacetoacetate hydrolase domain-containing protein 2A	0.01	1.00.E-17
Q4LE39-2	Isoform 2 of AT-rich interactive domain-containing protein 4B	0.01	1.00.E-17
Q86UY6	N-alpha-acetyltransferase 40	0.01	1.00.E-17
Q8N1A0	Keratin-like protein KRT222	0.058	3.74.E-02
Q05932	Folylpolyglutamate synthase, mitochondrial	0.037	1.12.E-02
P30307-3	Isoform 4 of M-phase inducer phosphatase 3	0.01	1.00.E-17
Q86V59	Paraneoplastic antigen-like protein 8A	0.01	1.00.E-17
Q9NVX2	Notchless protein homolog 1	0.01	1.00.E-17
Q14244-6	Isoform 6 of Ensconsin	0.01	1.00.E-17
Q9NUQ3-2	Isoform 2 of Gamma-taxilin	0.01	1.00.E-17
Q9BTX1	Nucleoporin NDC1	0.01	1.00.E-17
Q99689	Fasciculation and elongation protein zeta-1	0.01	1.00.E-17
Q96SZ6-5	Isoform 5 of Mitochondrial tRNA methylthiotransferase CDK5RAP1	0.01	1.00.E-17
Q32NC0-2	Isoform 2 of UPF0711 protein C18orf21	0.01	1.00.E-17
P15884-14	Isoform C- of Transcription factor 4	0.01	1.00.E-17
Q9H1X3-3	Isoform 3 of DnaJ homolog subfamily C member 25	0.01	1.00.E-17
Q14CB8-5	Isoform 5 of Rho GTPase-activating protein 19	0.01	1.00.E-17
O43929-2	Isoform 2 of Origin recognition complex subunit 4	0.01	1.00.E-17
Q9UL03	Integrator complex subunit 6	0.01	1.00.E-17
Q01860-2	Isoform B of POU domain, class 5, transcription factor 1	0.01	1.00.E-17
Q14676-3	Isoform 3 of Mediator of DNA damage checkpoint protein 1	0.05	2.46.E-02
Q9Y4A5	Transformation/transcription domain-associated protein	0.01	1.00.E-17
Q14678	KN motif and ankyrin repeat domain-containing protein 1	0.01	1.00.E-17
Q7Z3T8	Zinc finger FYVE domain-containing protein 16	0.01	1.00.E-17
Q96D96-3	Isoform 3 of Voltage-gated hydrogen channel 1	0.01	1.00.E-17
Q9H4L5-8	Isoform 2d of Oxysterol-binding protein-related protein 3	0.01	1.00.E-17
O96017-4	Isoform 4 of Serine/threonine-protein kinase Chk2	0.01	1.00.E-17
Q14244-4	Isoform 4 of Ensconsin	0.01	1.00.E-17
Q13111-3	Isoform 3 of Chromatin assembly factor 1 subunit A	0.01	1.00.E-17

Q15650	Activating signal cointegrator 1	0.01	1.00.E-17
Q9Y2X0	Mediator of RNA polymerase II transcription subunit 16	0.01	1.00.E-17
Q15910-4	Isoform 4 of Histone-lysine N-methyltransferase EZH2	0.01	1.00.E-17
Q96BP3-2	Isoform 2 of Peptidylprolyl isomerase domain and WD repeat-containing protein 1	0.01	1.00.E-17
O95070	Protein YIF1A	0.028	2.66.E-03
Q14451-2	Isoform 2 of Growth factor receptor-bound protein 7	0.01	1.00.E-17
O95926-2	Isoform 2 of Pre-mRNA-splicing factor SYF2	0.01	1.00.E-17
O14578-4	Isoform 4 of Citron Rho-interacting kinase	0.01	1.00.E-17
Q9ULU4-20	Isoform 20 of Protein kinase C-binding protein 1	0.01	1.00.E-17
Q15742-2	Isoform 2 of NGFI-A-binding protein 2	0.01	1.00.E-17
Q8IX90-3	Isoform 3 of Spindle and kinetochore-associated protein 3	0.01	1.00.E-17
Q14244-5	Isoform 5 of Ensconsin	0.01	1.00.E-17
Q6ZT62	Bargin	0.01	1.00.E-17
Q9UKS6	Protein kinase C and casein kinase substrate in neurons protein 3	0.01	1.00.E-17
P12110	Collagen alpha-2(VI) chain	0.01	1.00.E-17
P15884-13	Isoform 13 of Transcription factor 4	0.01	1.00.E-17
Q8N4C6-10	Isoform 3 of Ninein	0.01	1.00.E-17
Q9NVI1-4	Isoform 4 of Fanconi anemia group I protein	0.01	1.00.E-17
Q96QT6-4	Isoform 4 of PHD finger protein 12	0.01	1.00.E-17
Q9ULU4-2	Isoform 2 of Protein kinase C-binding protein 1	0.01	1.00.E-17
Q05932-2	Isoform 2 of Folylpolyglutamate synthase, mitochondrial	0.037	1.12.E-02
P57081	tRNA (guanine-N(7)-)-methyltransferase non-catalytic subunit WDR4	0.01	1.00.E-17
Q9HCN4-4	Isoform 4 of GPN-loop GTPase 1	0.01	1.00.E-17
Q9BVJ6	U3 small nucleolar RNA-associated protein 14 homolog A	0.01	1.00.E-17
Q9BXS6-2	Isoform 2 of Nucleolar and spindle-associated protein 1	0.01	1.00.E-17
Q9BST9-3	Isoform 3 of Rhotekin	0.01	1.00.E-17
Q9Y3Q3	Transmembrane emp24 domain-containing protein 3	0.01	1.00.E-17
Q5T3I0-3	Isoform 3 of G patch domain-containing protein 4	0.01	1.00.E-17
Q12962	Transcription initiation factor TFIID subunit 10	0.01	1.00.E-17
Q86V48-3	Isoform 3 of Leucine zipper protein 1	0.01	1.00.E-17
P29350-3	Isoform 2 of Tyrosine-protein phosphatase non-receptor type 6	0.01	1.00.E-17
Q6P1Q9	tRNA N(3)-methylcytidine methyltransferase METTL2B	0.01	1.00.E-17
Q9H6R4-2	Isoform 2 of Nucleolar protein 6	0.01	1.00.E-17
Q92833-3	Isoform 3 of Protein Jumonji	0.01	1.00.E-17
Q9BTX1-2	Isoform 2 of Nucleoporin NDC1	0.01	1.00.E-17
Q9H6S3	Epidermal growth factor receptor kinase substrate 8-like protein 2	0.01	1.00.E-17
Q13472	DNA topoisomerase 3-alpha	0.01	1.00.E-17
Q9NVM4-2	Isoform 2 of Protein arginine N-methyltransferase 7	0.01	1.00.E-17
Q8TD55-2	Isoform 2 of Pleckstrin homology domain-containing family O member 2	0.01	1.00.E-17
Q9UJ83-4	Isoform 4 of 2-hydroxyacyl-CoA lyase 1	0.01	1.00.E-17
P15884-16	Isoform I- of Transcription factor 4	0.01	1.00.E-17
O95297-5	Isoform 5 of Myelin protein zero-like protein 1	0.01	1.00.E-17
P41214-2	Isoform 2 of Eukaryotic translation initiation factor 2D	0.01	1.00.E-17
Q99576-4	Isoform 3 of TSC22 domain family protein 3	0.01	1.00.E-17
P29350	Tyrosine-protein phosphatase non-receptor type 6	0.01	1.00.E-17
P21802-8	Isoform 7 of Fibroblast growth factor receptor 2	0.01	1.00.E-17
P98173	Protein FAM3A	0.01	1.00.E-17
Q9P0N9-2	Isoform 2 of TBC1 domain family member 7	0.01	1.00.E-17
O95453-4	Isoform 4 of Poly(A)-specific ribonuclease PARN	0.086	4.93.E-02
P07910	Heterogeneous nuclear ribonucleoproteins C1/C2	0.01	1.00.E-17
P61024	Cyclin-dependent kinases regulatory subunit 1	0.076	3.60.E-02
P15884-10	Isoform F- of Transcription factor 4	0.01	1.00.E-17
O15446	DNA-directed RNA polymerase I subunit RPA34	0.01	1.00.E-17
Q8WXC6	COP9 signalosome complex subunit 9	0.01	1.00.E-17
Q9C0B1-3	Isoform 3 of Alpha-ketoglutarate-dependent dioxygenase FTO	0.01	1.00.E-17
Q14244-2	Isoform 2 of Ensconsin	0.01	1.00.E-17
Q9BXS6-3	Isoform 3 of Nucleolar and spindle-associated protein 1	0.01	1.00.E-17
Q8N108-15	Isoform 5 of Mesoderm induction early response protein 1	0.01	1.00.E-17
Q9NP50-2	Isoform 2 of SIN3-HDAC complex-associated factor	0.01	1.00.E-17
Q96MG7	Non-structural maintenance of chromosomes element 3 homolog	0.057	2.94.E-02
Q14191	Werner syndrome ATP-dependent helicase	0.01	1.00.E-17
Q14CB8-2	Isoform 2 of Rho GTPase-activating protein 19	0.01	1.00.E-17
Q05932-3	Isoform 3 of Folylpolyglutamate synthase, mitochondrial	0.037	1.12.E-02
P31947	14-3-3 protein sigma	0.01	1.00.E-17
Q9UBN4	Short transient receptor potential channel 4	0.01	1.00.E-17
O75122	CLIP-associating protein 2	0.01	1.00.E-17

Q9UJX3-2	Isoform 2 of Anaphase-promoting complex subunit 7	0.01	1.00.E-17
Q9UHI5-2	Isoform 2 of Large neutral amino acids transporter small subunit 2	0.01	1.00.E-17
Q8IVW6	AT-rich interactive domain-containing protein 3B	0.01	1.00.E-17
Q86UY6-3	Isoform 2 of N-alpha-acetyltransferase 40	0.01	1.00.E-17
Q96RT1-9	Isoform 9 of Erbin	0.01	1.00.E-17
Q9NW08-2	Isoform 2 of DNA-directed RNA polymerase III subunit RPC2	0.051	1.21.E-02
Q969I6	Sodium-coupled neutral amino acid transporter 4	0.01	1.00.E-17
Q96DR4-2	Isoform 2 of StAR-related lipid transfer protein 4	0.01	1.00.E-17
Q6KC79-3	Isoform 3 of Nipped-B-like protein	0.01	1.00.E-17
P59780	AP-3 complex subunit sigma-2	0.01	1.00.E-17
Q9H813-2	Isoform 2 of Proton-activated chloride channel	0.01	1.00.E-17
P55210	Caspase-7	0.01	1.00.E-17
Q13439-4	Isoform 4 of Golgin subfamily A member 4	0.01	1.00.E-17
Q9H2S1-2	Isoform 2 of Small conductance calcium-activated potassium channel protein 2	0.01	1.00.E-17
P55211-2	Isoform 2 of Caspase-9	0.01	1.00.E-17
O43683	Mitotic checkpoint serine/threonine-protein kinase BUB1	0.046	2.22.E-02
Q8WWK9	Cytoskeleton-associated protein 2	0.01	1.00.E-17
P45983-3	Isoform 3 of Mitogen-activated protein kinase 8	0.01	1.00.E-17
P22607-3	Isoform 3 of Fibroblast growth factor receptor 3	0.01	1.00.E-17
Q9Y3B9	RRP15-like protein	0.01	1.00.E-17
Q86U28-2	Isoform 2 of Iron-sulfur cluster assembly 2 homolog, mitochondrial	0.01	1.00.E-17
Q9Y6X9-2	Isoform 2 of ATPase MORC2	0.01	1.00.E-17
Q9Y3Q8	TSC22 domain family protein 4	0.01	1.00.E-17
Q8IX90	Spindle and kinetochore-associated protein 3	0.01	1.00.E-17
Q16875-3	Isoform 3 of 6-phosphofructo-2-kinase/fructose-2,6-bisphosphatase 3	0.01	1.00.E-17
Q9Y6X9	ATPase MORC2	0.01	1.00.E-17
Q96QT6	PHD finger protein 12	0.01	1.00.E-17
P20839	Inosine-5'-monophosphate dehydrogenase 1	0.01	1.00.E-17
O00570	Transcription factor SOX-1	0.01	1.00.E-17
Q969X6-3	Isoform 3 of U3 small nucleolar RNA-associated protein 4 homolog	0.01	1.00.E-17
Q6NT16	MFS-type transporter SLC18B1	0.01	1.00.E-17
Q9BRF8-2	Isoform 2 of Serine/threonine-protein phosphatase CPPED1	0.01	1.00.E-17
Q05513	Protein kinase C zeta type	0.056	2.69.E-02
Q99590-2	Isoform 2 of Protein SCAF11	0.01	1.00.E-17
Q9UJX4-3	Isoform 3 of Anaphase-promoting complex subunit 5	0.01	1.00.E-17
Q8N490	Probable hydrolase PNKD	0.01	1.00.E-17
Q9BTX1-6	Isoform 6 of Nucleoporin NDC1	0.01	1.00.E-17
Q9H6R3	Acyl-CoA synthetase short-chain family member 3, mitochondrial	0.01	1.00.E-17
O95484	Claudin-9	0.01	1.00.E-17
Q9ULU4-11	Isoform 11 of Protein kinase C-binding protein 1	0.01	1.00.E-17
Q6FIF0-2	Isoform 2 of AN1-type zinc finger protein 6	0.01	1.00.E-17
Q12816-2	Isoform 2 of Trophinin	0.01	1.00.E-17
Q9BY11	Protein kinase C and casein kinase substrate in neurons protein 1	0.01	1.00.E-17
Q06547	GA-binding protein subunit beta-1	0.01	1.00.E-17
Q9UJK0	18S rRNA aminocarboxypropyltransferase	0.01	1.00.E-17
Q8WUP2	Filamin-binding LIM protein 1	0.01	1.00.E-17
Q9BWG4-2	Isoform 2 of Single-stranded DNA-binding protein 4	0.01	1.00.E-17
Q9Y3C1-2	Isoform 2 of Nucleolar protein 16	0.01	1.00.E-17
Q13439-5	Isoform 5 of Golgin subfamily A member 4	0.01	1.00.E-17
Q9Y3B8	Oligoribonuclease, mitochondrial	0.01	1.00.E-17
O60291	E3 ubiquitin-protein ligase MGRN1	0.01	1.00.E-17
Q99576-3	Isoform 2 of TSC22 domain family protein 3	0.01	1.00.E-17
Q9GZU8	PSME3-interacting protein	0.01	1.00.E-17
Q969L2	Protein MAL2	0.01	1.00.E-17
Q9Y6X3-3	Isoform 3 of MAU2 chromatid cohesion factor homolog	0.01	1.00.E-17
Q92540-2	Isoform 2 of Protein SMG7	0.01	1.00.E-17
Q9HCK8-2	Isoform 2 of Chromodomain-helicase-DNA-binding protein 8	0.01	1.00.E-17
Q32NC0	UPF0711 protein C18orf21	0.01	1.00.E-17
P52630-4	Isoform 2 of Signal transducer and activator of transcription 2	0.01	1.00.E-17
Q8N4C6-6	Isoform 6 of Ninein	0.01	1.00.E-17
Q92833	Protein Jumonji	0.01	1.00.E-17
Q5T3I0	G patch domain-containing protein 4	0.01	1.00.E-17
A6ND36	Protein FAM83G	0.01	1.00.E-17
Q9ULU4-23	Isoform 23 of Protein kinase C-binding protein 1	0.01	1.00.E-17
P40967-2	Isoform 2 of Melanocyte protein PMEL	0.01	1.00.E-17
Q6N069-3	Isoform 3 of N-alpha-acetyltransferase 16, NatA auxiliary subunit	0.01	1.00.E-17

Q13574-3	Isoform 3 of Diacylglycerol kinase zeta	0.01	1.00.E-17
P30291	Wee1-like protein kinase	0.01	1.00.E-17
P21802-21	Isoform 15 of Fibroblast growth factor receptor 2	0.01	1.00.E-17
Q9Y6W5-2	Isoform 2 of Wiskott-Aldrich syndrome protein family member 2	0.01	1.00.E-17
Q9BVC3	Sister chromatid cohesion protein DCC1	0.01	1.00.E-17
Q9NVM9	Integrator complex subunit 13	0.01	1.00.E-17
Q9NZJ9-2	Isoform 2 of Diphosphoinositol polyphosphate phosphohydrolase 2	0.01	1.00.E-17
Q8WWK9-5	Isoform 3 of Cytoskeleton-associated protein 2	0.01	1.00.E-17
Q96DZ1-2	Isoform 2 of Endoplasmic reticulum lectin 1	0.01	1.00.E-17
Q15652-2	Isoform 2 of Probable JmjC domain-containing histone demethylation protein 2C	0.01	1.00.E-17
C9JQL5	Putative dispanin subfamily A member 2d	0.01	1.00.E-17
P29084	Transcription initiation factor IIE subunit beta	0.01	1.00.E-17
Q9BT25-2	Isoform 2 of HAUS augmin-like complex subunit 8	0.01	1.00.E-17
Q9NVU0-2	Isoform 2 of DNA-directed RNA polymerase III subunit RPC5	0.043	2.42.E-02
Q6N069-4	Isoform 4 of N-alpha-acetyltransferase 16, NatA auxiliary subunit	0.01	1.00.E-17
Q9UBU8-3	Isoform 3 of Mortality factor 4-like protein 1	0.01	1.00.E-17
P48431	Transcription factor SOX-2	0.01	1.00.E-17
Q96RT1-3	Isoform 3 of Erbin	0.01	1.00.E-17
O75683	Surfeit locus protein 6	0.01	1.00.E-17
Q9UBP6-2	Isoform 2 of tRNA (guanine-N(7)-)-methyltransferase	0.01	1.00.E-17
Q8N108-12	Isoform 2 of Mesoderm induction early response protein 1	0.01	1.00.E-17
Q14244	Ensconsin	0.01	1.00.E-17
P49641	Alpha-mannosidase 2x	0.01	1.00.E-17
P40967	Melanocyte protein PMEL	0.01	1.00.E-17
Q8TE68-2	Isoform 2 of Epidermal growth factor receptor kinase substrate 8-like protein 1	0.01	1.00.E-17
Q9BRZ2	E3 ubiquitin-protein ligase TRIM56	0.01	1.00.E-17
Q9BUL5-4	Isoform 4 of PHD finger protein 23	0.01	1.00.E-17
P20839-4	Isoform 4 of Inosine-5'-monophosphate dehydrogenase 1	0.01	1.00.E-17
Q9Y3C1	Nucleolar protein 16	0.01	1.00.E-17
Q6YHU6-6	Isoform 6 of Thyroid adenoma-associated protein	0.01	1.00.E-17
P21802-15	Isoform 9 of Fibroblast growth factor receptor 2	0.01	1.00.E-17
P41225	Transcription factor SOX-3	0.01	1.00.E-17
Q9BT09	Protein canopy homolog 3	0.01	1.00.E-17
Q8NFM7-2	Isoform 2 of Interleukin-17 receptor D	0.01	1.00.E-17
Q9NXS2-3	Isoform 2 of Glutaminyl-peptide cyclotransferase-like protein	0.01	1.00.E-17
Q9NQW6-2	Isoform 2 of Anillin	0.01	1.00.E-17
Q96RT1-2	Isoform 2 of Erbin	0.01	1.00.E-17
O75448-2	Isoform 2 of Mediator of RNA polymerase II transcription subunit 24	0.01	1.00.E-17
O95273	Cyclin-D1-binding protein 1	0.01	1.00.E-17
O95379-2	Isoform 2 of Tumor necrosis factor alpha-induced protein 8	0.041	9.58.E-03
P33981-2	Isoform 2 of Dual specificity protein kinase TTK	0.01	1.00.E-17
Q9NRX1	RNA-binding protein PNO1	0.06	4.90.E-02
Q9UBP9-3	Isoform 3 of PTB domain-containing engulfment adapter protein 1	0.01	1.00.E-17
Q86TS9-3	Isoform 3 of 39S ribosomal protein L52, mitochondrial	0.01	1.00.E-17
Q96AD5	Patatin-like phospholipase domain-containing protein 2	0.01	1.00.E-17
Q9Y4A5-2	Isoform 2 of Transformation/transcription domain-associated protein	0.01	1.00.E-17
Q9BZD4	Kinetochore protein Nuf2	0.01	1.00.E-17
P50748-2	Isoform 2 of Kinetochore-associated protein 1	0.01	1.00.E-17
P21802-22	Isoform 16 of Fibroblast growth factor receptor 2	0.01	1.00.E-17
Q8WWK9-4	Isoform 2 of Cytoskeleton-associated protein 2	0.01	1.00.E-17
Q9NUQ3	Gamma-taxilin	0.01	1.00.E-17
Q14781	Chromobox protein homolog 2	0.052	2.28.E-02
Q14451	Growth factor receptor-bound protein 7	0.01	1.00.E-17
O94806	Serine/threonine-protein kinase D3	0.01	1.00.E-17
Q9Y3C1-3	Isoform 3 of Nucleolar protein 16	0.01	1.00.E-17
Q9NVM9-2	Isoform 2 of Integrator complex subunit 13	0.01	1.00.E-17
Q9NSI2-2	Isoform B of Protein FAM207A	0.01	1.00.E-17
Q92540-5	Isoform 5 of Protein SMG7	0.01	1.00.E-17
Q5M9Q1	NKAP-like protein	0.01	1.00.E-17
P80365	Corticosteroid 11-beta-dehydrogenase isozyme 2	0.01	1.00.E-17
Q9UHR5-2	Isoform 2 of SAP30-binding protein	0.01	1.00.E-17
Q8WVC0	RNA polymerase-associated protein LEO1	0.01	1.00.E-17
Q9UHP3-3	Isoform USP25m of Ubiquitin carboxyl-terminal hydrolase 25	0.01	1.00.E-17
Q9BY89	Uncharacterized protein KIAA1671	0.01	1.00.E-17
P04818	Thymidylate synthase	0.03	7.87.E-03
Q2VPK5-5	Isoform 3 of Cytoplasmic tRNA 2-thiolation protein 2	0.01	1.00.E-17

Q96DZ1-3	Isoform 3 of Endoplasmic reticulum lectin 1	0.01	1.00.E-17
O95379	Tumor necrosis factor alpha-induced protein 8	0.041	9.58.E-03
P21709-3	Isoform 3 of Ephrin type-A receptor 1	0.01	1.00.E-17
Q8IVW6-3	Isoform 3 of AT-rich interactive domain-containing protein 3B	0.01	1.00.E-17
Q86Y37	CDK2-associated and cullin domain-containing protein 1	0.01	1.00.E-17
Q13277-2	Isoform B of Syntaxin-3	0.01	1.00.E-17
Q9ULU4-7	Isoform 7 of Protein kinase C-binding protein 1	0.01	1.00.E-17
Q8TAG9	Exocyst complex component 6	0.01	1.00.E-17
Q5VZK9	F-actin-uncapping protein LRRC16A	0.01	1.00.E-17
P41440-3	Isoform 3 of Reduced folate transporter	0.01	1.00.E-17
Q9UHR5	SAP30-binding protein	0.01	1.00.E-17
Q96RT1-7	Isoform 7 of Erbin	0.01	1.00.E-17
Q9BU61	NADH dehydrogenase [ubiquinone] 1 alpha subcomplex assembly factor 3	0.01	1.00.E-17
P29508-2	Isoform 2 of Serpin B3	0.01	1.00.E-17
O95453-3	Isoform 3 of Poly(A)-specific ribonuclease PARN	0.086	4.93.E-02
Q16875	6-phosphofructo-2-kinase/fructose-2,6-bisphosphatase 3	0.01	1.00.E-17
Q9H467	CUE domain-containing protein 2	0.01	1.00.E-17
Q9UBN4-5	Isoform Epsilon of Short transient receptor potential channel 4	0.01	1.00.E-17
O60291-4	Isoform 4 of E3 ubiquitin-protein ligase MGRN1	0.01	1.00.E-17
Q9BXS9-5	Isoform 5 of Solute carrier family 26 member 6	0.01	1.00.E-17
Q6PGN9-3	Isoform B of Proline/serine-rich coiled-coil protein 1	0.01	1.00.E-17
P29508	Serpin B3	0.01	1.00.E-17
Q8N108-18	Isoform 8 of Mesoderm induction early response protein 1	0.01	1.00.E-17
O60343-5	Isoform 5 of TBC1 domain family member 4	0.01	1.00.E-17
Q6YHU6-2	Isoform 2 of Thyroid adenoma-associated protein	0.01	1.00.E-17
P49641-1	Isoform 1 of Alpha-mannosidase 2x	0.01	1.00.E-17
Q3L8U1-3	Isoform 3 of Chromodomain-helicase-DNA-binding protein 9	0.01	1.00.E-17
P84101-4	Isoform 4 of Small EDRK-rich factor 2	0.01	1.00.E-17
P15884	Transcription factor 4	0.01	1.00.E-17
Q8TE77-3	Isoform 3 of Protein phosphatase Slingshot homolog 3	0.01	1.00.E-17
Q12774-2	Isoform 2 of Rho guanine nucleotide exchange factor 5	0.01	1.00.E-17
Q8WUB8-3	Isoform 3 of PHD finger protein 10	0.053	1.38.E-02
Q8N108-13	Isoform 3 of Mesoderm induction early response protein 1	0.01	1.00.E-17
Q6KC79	Nipped-B-like protein	0.01	1.00.E-17
Q9H9Y6	DNA-directed RNA polymerase I subunit RPA2	0.025	1.89.E-03
O43929-3	Isoform 3 of Origin recognition complex subunit 4	0.01	1.00.E-17
P15884-15	Isoform C-delta of Transcription factor 4	0.01	1.00.E-17
Q9UJX4-2	Isoform 2 of Anaphase-promoting complex subunit 5	0.01	1.00.E-17
Q9H9A7	RecQ-mediated genome instability protein 1	0.01	1.00.E-17
P05161	Ubiquitin-like protein ISG15	0.01	1.00.E-17
Q86UZ6	Zinc finger and BTB domain-containing protein 46	0.01	1.00.E-17
Q10589	Bone marrow stromal antigen 2	0.01	1.00.E-17
Q9UHI5-3	Isoform 3 of Large neutral amino acids transporter small subunit 2	0.01	1.00.E-17
P10620-2	Isoform 2 of Microsomal glutathione S-transferase 1	0.041	8.39.E-03
Q8N1A0-2	Isoform 2 of Keratin-like protein KRT222	0.058	3.74.E-02
P56270-2	Isoform 2 of Myc-associated zinc finger protein	0.01	1.00.E-17
Q9ULU4-5	Isoform 5 of Protein kinase C-binding protein 1	0.01	1.00.E-17
Q99959	Plakophilin-2	0.01	1.00.E-17
Q9H4L5-5	Isoform 2a of Oxysterol-binding protein-related protein 3	0.01	1.00.E-17
P40967-4	Isoform 4 of Melanocyte protein PMEL	0.01	1.00.E-17
Q9Y6X3-2	Isoform 2 of MAU2 chromatid cohesion factor homolog	0.01	1.00.E-17
Q5VZ89	DENN domain-containing protein 4C	0.01	1.00.E-17
Q9NVM4	Protein arginine N-methyltransferase 7	0.01	1.00.E-17
Q9NQ84-2	Isoform 2 of G-protein coupled receptor family C group 5 member C	0.01	1.00.E-17
Q9H469	F-box/LRR-repeat protein 15	0.01	1.00.E-17
Q8TE77-2	Isoform 2 of Protein phosphatase Slingshot homolog 3	0.01	1.00.E-17
Q9Y5K6	CD2-associated protein	0.01	1.00.E-17
Q8TD26-3	Isoform 3 of Chromodomain-helicase-DNA-binding protein 6	0.01	1.00.E-17
Q9UP95-4	Isoform 4 of Solute carrier family 12 member 4	0.01	1.00.E-17
Q9NZN4-2	Isoform 2 of EH domain-containing protein 2	0.01	1.00.E-17
P84101-3	Isoform 3 of Small EDRK-rich factor 2	0.01	1.00.E-17
Q6KC79-2	Isoform 2 of Nipped-B-like protein	0.01	1.00.E-17
P55210-4	Isoform 4 of Caspase-7	0.01	1.00.E-17
Q9BRJ2	39S ribosomal protein L45, mitochondrial	0.01	1.00.E-17
Q9NQ84	G-protein coupled receptor family C group 5 member C	0.01	1.00.E-17
P41134-2	Isoform ID-B of DNA-binding protein inhibitor ID-1	0.01	1.00.E-17

P15884-12	Isoform E- of Transcription factor 4	0.01	1.00.E-17
P09493-8	Isoform 8 of Tropomyosin alpha-1 chain	0.012	4.24.E-05
Q9H6Y2	WD repeat-containing protein 55	0.01	1.00.E-17
Q8WUP2-2	Isoform 2 of Filamin-binding LIM protein 1	0.01	1.00.E-17
Q9Y219-2	Isoform Short of Protein jagged-2	0.01	1.00.E-17
Q15910	Histone-lysine N-methyltransferase EZH2	0.01	1.00.E-17
Q14451-3	Isoform 3 of Growth factor receptor-bound protein 7	0.01	1.00.E-17
Q9H9Y6-4	Isoform 4 of DNA-directed RNA polymerase I subunit RPA2	0.025	1.89.E-03
Q96D96-4	Isoform 4 of Voltage-gated hydrogen channel 1	0.01	1.00.E-17
Q9H4L5-2	Isoform 1b of Oxysterol-binding protein-related protein 3	0.01	1.00.E-17
Q96II8	DISP complex protein LRCH3	0.01	1.00.E-17
Q9NWT1	p21-activated protein kinase-interacting protein 1	0.01	1.00.E-17
Q3L8U1	Chromodomain-helicase-DNA-binding protein 9	0.01	1.00.E-17
Q9NVM4-3	Isoform 3 of Protein arginine N-methyltransferase 7	0.01	1.00.E-17
Q96FC9	ATP-dependent DNA helicase DDX11	0.01	1.00.E-17
P50579-2	Isoform 2 of Methionine aminopeptidase 2	0.01	1.00.E-17
Q13472-3	Isoform 3 of DNA topoisomerase 3-alpha	0.01	1.00.E-17
P51810	G-protein coupled receptor 143	0.01	1.00.E-17
Q8NI60-3	Isoform 3 of Atypical kinase COQ8A, mitochondrial	0.01	1.00.E-17
Q9Y248	DNA replication complex GINS protein PSF2	0.01	1.00.E-17
Q9BU76-2	Isoform 2 of Multiple myeloma tumor-associated protein 2	0.01	1.00.E-17
Q96KG7	Multiple epidermal growth factor-like domains protein 10	0.01	1.00.E-17
Q9NWS0	PIH1 domain-containing protein 1	0.01	1.00.E-17
Q8TDP1	Ribonuclease H2 subunit C	0.01	1.00.E-17
Q53GG5-3	Isoform 3 of PDZ and LIM domain protein 3	0.01	1.00.E-17
P09327-2	Isoform 2 of Villin-1	0.01	1.00.E-17
Q13439-3	Isoform 3 of Golgin subfamily A member 4	0.01	1.00.E-17
Q9NSA3	Beta-catenin-interacting protein 1	0.01	1.00.E-17
Q9H7L9	Sin3 histone deacetylase corepressor complex component SDS3	0.01	1.00.E-17
O14732	Inositol monophosphatase 2	0.01	1.00.E-17
Q15054-2	Isoform 2 of DNA polymerase delta subunit 3	0.01	1.00.E-17
Q8N442	Translation factor GUF1, mitochondrial	0.01	1.00.E-17
Q5T0Z8	Uncharacterized protein C6orf132	0.01	1.00.E-17
Q5JTV8-2	Isoform 2 of Torsin-1A-interacting protein 1	0.01	1.00.E-17
Q9H9P8-2	Isoform 2 of L-2-hydroxyglutarate dehydrogenase, mitochondrial	0.01	1.00.E-17
Q9BRP4-3	Isoform 3 of Proteasomal ATPase-associated factor 1	0.01	1.00.E-17
Q9H8H0-2	Isoform 2 of Nucleolar protein 11	0.01	1.00.E-17
Q86X29-2	Isoform 2 of Lipolysis-stimulated lipoprotein receptor	0.01	1.00.E-17
O43278	Kunitz-type protease inhibitor 1	0.01	1.00.E-17
P20839-6	Isoform 6 of Inosine-5'-monophosphate dehydrogenase 1	0.01	1.00.E-17
Q14653-5	Isoform 5 of Interferon regulatory factor 3	0.01	1.00.E-17
P40967-5	Isoform 5 of Melanocyte protein PMEL	0.01	1.00.E-17
Q13574-5	Isoform 5 of Diacylglycerol kinase zeta	0.01	1.00.E-17
Q9UMX1-3	Isoform 3 of Suppressor of fused homolog	0.01	1.00.E-17
Q01973-2	Isoform Short of Inactive tyrosine-protein kinase transmembrane receptor ROR1	0.01	1.00.E-17
O75970-3	Isoform 3 of Multiple PDZ domain protein	0.01	1.00.E-17
P20839-7	Isoform 7 of Inosine-5'-monophosphate dehydrogenase 1	0.01	1.00.E-17
Q96D15	Reticulocalbin-3	0.01	1.00.E-17
Q6YHU6	Thyroid adenoma-associated protein	0.01	1.00.E-17
Q15746-4	Isoform 3B of Myosin light chain kinase, smooth muscle	0.01	1.00.E-17
P58340-4	Isoform 4 of Myeloid leukemia factor 1	0.01	1.00.E-17
P21802-20	Isoform 14 of Fibroblast growth factor receptor 2	0.01	1.00.E-17
Q6NW34-2	Isoform 2 of Nucleolus and neural progenitor protein	0.01	1.00.E-17
Q5VZL5-4	Isoform 4 of Zinc finger MYM-type protein 4	0.01	1.00.E-17
Q14CZ7	FAST kinase domain-containing protein 3, mitochondrial	0.01	1.00.E-17
Q15054-3	Isoform 3 of DNA polymerase delta subunit 3	0.01	1.00.E-17
Q5SVZ6	Zinc finger MYM-type protein 1	0.01	1.00.E-17
P11117-2	Isoform 2 of Lysosomal acid phosphatase	0.01	1.00.E-17
P52569-3	Isoform 3 of Cationic amino acid transporter 2	0.01	1.00.E-17
P41440-2	Isoform 2 of Reduced folate transporter	0.01	1.00.E-17
Q9NRG1	Phosphoribosyltransferase domain-containing protein 1	0.01	1.00.E-17
Q96T23	Remodeling and spacing factor 1	0.01	1.00.E-17
Q8N108-14	Isoform 4 of Mesoderm induction early response protein 1	0.01	1.00.E-17
Q9Y3L3-2	Isoform 2 of SH3 domain-binding protein 1	0.01	1.00.E-17
Q9Y6W5	Wiskott-Aldrich syndrome protein family member 2	0.01	1.00.E-17
Q96RL1	BRCA1-A complex subunit RAP80	0.01	1.00.E-17

Q14676	Mediator of DNA damage checkpoint protein 1	0.05	2.46.E-02
Q9BUL5	PHD finger protein 23	0.01	1.00.E-17
Q14202-3	Isoform 3 of Zinc finger MYM-type protein 3	0.01	1.00.E-17
Q01973-3	Isoform 3 of Inactive tyrosine-protein kinase transmembrane receptor ROR1	0.01	1.00.E-17
Q9H4H8	Protein FAM83D	0.01	1.00.E-17
O75843	AP-1 complex subunit gamma-like 2	0.01	1.00.E-17
O96017-13	Isoform 13 of Serine/threonine-protein kinase Chk2	0.01	1.00.E-17
O43683-2	Isoform 2 of Mitotic checkpoint serine/threonine-protein kinase BUB1	0.046	2.22.E-02
Q9BRF8-3	Isoform 3 of Serine/threonine-protein phosphatase CPPED1	0.01	1.00.E-17
Q6IQ49-2	Isoform 2 of Replication stress response regulator SDE2	0.01	1.00.E-17
Q12774	Rho guanine nucleotide exchange factor 5	0.01	1.00.E-17
P58340-5	Isoform 5 of Myeloid leukemia factor 1	0.01	1.00.E-17
P41214	Eukaryotic translation initiation factor 2D	0.01	1.00.E-17
Q9H4H8-2	Isoform 2 of Protein FAM83D	0.01	1.00.E-17
P50151	Guanine nucleotide-binding protein G(I)/G(S)/G(O) subunit gamma-10	0.01	1.00.E-17
Q7Z5L2-2	Isoform 2 of Coiled-coil domain-containing protein R3HCC1L	0.01	1.00.E-17
P58340-3	Isoform 3 of Myeloid leukemia factor 1	0.01	1.00.E-17
O43913	Origin recognition complex subunit 5	0.01	1.00.E-17
P21802-4	Isoform 4 of Fibroblast growth factor receptor 2	0.01	1.00.E-17
Q92925-2	Isoform 2 of SWI/SNF-related matrix-associated actin-dependent regulator of chromatin subfamily D member 2	0.01	1.00.E-17
Q9Y3A4	Ribosomal RNA-processing protein 7 homolog A	0.01	1.00.E-17
P55210-3	Isoform Alpha' of Caspase-7	0.01	1.00.E-17
Q96BP3	Peptidylprolyl isomerase domain and WD repeat-containing protein 1	0.01	1.00.E-17
Q9H501	ESF1 homolog	0.01	1.00.E-17
Q9H1D9	DNA-directed RNA polymerase III subunit RPC6	0.01	1.00.E-17
Q96T88-2	Isoform 2 of E3 ubiquitin-protein ligase UHRF1	0.01	1.00.E-17
Q8IW92	Beta-galactosidase-1-like protein 2	0.01	1.00.E-17
Q10589-2	Isoform 2 of Bone marrow stromal antigen 2	0.01	1.00.E-17
O43741	5'-AMP-activated protein kinase subunit beta-2	0.01	1.00.E-17
Q9HCN4-5	Isoform 5 of GPN-loop GTPase 1	0.01	1.00.E-17
P30414	NK-tumor recognition protein	0.01	1.00.E-17
Q6BCY4	NADH-cytochrome b5 reductase 2	0.047	7.85.E-03
Q9Y3L3	SH3 domain-binding protein 1	0.01	1.00.E-17
P15884-3	Isoform SEF2-1D of Transcription factor 4	0.01	1.00.E-17
P41440	Reduced folate transporter	0.01	1.00.E-17
Q9NRZ9-8	Isoform 8 of Lymphoid-specific helicase	0.01	1.00.E-17
Q9NZN4	EH domain-containing protein 2	0.01	1.00.E-17
Q9UP95-6	Isoform 6 of Solute carrier family 12 member 4	0.01	1.00.E-17
Q96T88	E3 ubiquitin-protein ligase UHRF1	0.01	1.00.E-17

Supplementary Table S2. (a) Significantly enriched terms in the GO enrichment analysis with proteins with increased level in NSCs.

Term (Top10)	Count	-log10(p-value)	Gene Name
glutathione derivative biosynthetic process	8	6.1	ESD/GSTA4/GSTM1/GSTM2/GSTM3/GSTM4/GSTM5/GSTP1
brain development	18	5.2	ATP2B1/H2AFY2/NDRG4/ACAT1/ABR/ADGRG1/CASP2/CADM1/GABRA5/ HPCA/HNMT/IFT172/MEN1/NES/PITPNM1/PLXNB2/SRR/SYNJ1
semaphorin-plexin signaling pathway involved in axon guidance	6	5.2	NRP1/PLXNA2/PLXNA4/PLXNB2/PLXNC1/PLXND1
glutathione metabolic process	10	5	ALDH5A1/GDAP1/GSTA4/GSTM1/GSTM2/GSTM3/GSTM4/GSTM5/GSTP1/SOD2
branchiomotor neuron axon guidance	5	4.4	NRP1/PLXNA2/PLXNA4/PLXNC1/PLXND1
central nervous system development	13	4.3	SRC/ALDH3A2/ALDH5A1/DNER/DCX/DRP2/GSTP1/HPCAL4/NES/ PTPRZ1/RPS6KA3/SERPINI1/TAGLN3
nitrobenzene metabolic process	4	4.2	GSTM1/GSTM2/GSTM3/GSTM4
axon development	5	4.1	DDR1/GAP43/NEFH/NEFL/NEFM
regulation of cardiac muscle contraction by regulation of the release of sequestered calcium ion	6	4.1	ATP1A2/CAMK2D/DMD/GSTM2/PRKACA/SLC8A1
nervous system development	20	4	GPSM1/GFRA1/NAB2/APBA2/APP/CBLN1/CNTFR/CRMP1/CNTN3/DPYSL4/ DPYSL5/DCX/FABP7/KALRN/LSAMP/LY6H/MAP1B/MSI1/NLGN1/SLC4A10

Supplementary Table S2. (b) Significantly enriched terms in the GO enrichment analysis with proteins with increased level in NPCs.

Term (Top10)	Count	-log10(p-value)	Gene Name
cell adhesion	27	4.3	ADAM9/ATP1B2/CD47/PPFIBP1/ADGRG1/APP/CDH2/CDH4/CDH6/CTNNA2/ CNTN1/CNTN3/EFNB1/GNE/ITGA3/ITGA5/ITGA7/ITGAV/LOXL2/NCAM1/NEDD9/ NLGN1/NRP2/RHOB/SORBS2/THBS1/TPBG
mesenchyme migration	4	3.9	ACTA1/ACTA2/ACTC1/ACTG2
protein localization to synapse	5	3.7	CDK5/KLC1/NRXN1/NLGN1/NLGN2
dendrite morphogenesis	7	3.7	ELAVL4/FARP1/CTNNA2/CDK5/ITGB1/MAP2/SDC2
muscle contraction	11	3.6	ACTA1/ACTA2/ACTG2/CALD1/CALM1/CRYAB/DES/GAMT/ITGA1/MYOF/SLC8A1
fatty acid beta-oxidation	7	3.2	DECR1/ABCD1/ACADVL/CROT/HADH/HADHA/HADHB
integrin-mediated signaling pathway	10	3.2	ADAM9/CD47/ITGA1/ITGA3/ITGA5/ITGA7/ITGAV/ITGB1/MYH9/NEDD9
synapse assembly	8	3.2	FARP1/WNT5A/ADGRL3/CDK5/NRXN1/NRXN3/NLGN1/NLGN2
aging	13	3.2	ABAT/ATP2B1/LRP1/RELA/ADA/CANX/CRYAB/ENO3/KRT83/LOXL2/PDE4D/ PPP1R9B/SRR
positive regulation of establishment of protein localization to plasma membrane	6	3.2	EPHA2/ITGA3/ITGB1/KIF5B/NRXN1/PPP1R9B

Supplementary Table S2. (c) Significantly enriched terms in the GO enrichment analysis with proteins with increased level in DAs.

Term (Top10)	Count	-log10(p-value)	Gene Name
neurotransmitter secretion	18	14.2	DNAJC5/PPFIA2/RAB3A/APBA1/LIN7B/NRXN1/NRXN2/NRXN3/SLC32A1/SYN1/SYN3/SYT1/SYT2/STX1A/STXBP1/UNC13A/UNC13B/VAMP2
nervous system development	29	8.6	ELAVL3/EVL/GNG8/GPSM1/GFRA1/L1CAM/SRGAP2B/APBA1/APBA2/APP/CAMK2G/CBLN1/CNTFR/CRMP1/DPYSL2/DPYSL4/DPYSL5/DLG2/DCLK1/DCX/DCTN1/GPM6B/KALRN/LSAMP/LY6H/MAP1B/NLGN1/SCN3B/SLC4A10
synaptic vesicle exocytosis	9	7.3	DNAJC5/RAB3A/ADGRL1/CADPS2/CADPS/UNC13A/UNC13B/UNC13C/VAMP213C/VAMP2
axon guidance	19	6.7	EVL/L1CAM/CHL1/CRMP1/DPYSL2/DPYSL5/EFNB1/EFNB2/KIF5C/MAPK8IP3/NEO1/NCAM1/NRXN1/NRXN3/NRP1/NRP2/SLIT1/SLIT2/TUBB3
chemical synaptic transmission	23	6.4	NOVA1/AMPH/APBA1/APBA2/CBLN1/DLG2/DTNA/GABRA5/MYO5A/NRXN1/NRXN2/PRKCG/PTPRD/RTN3/SLC1A2/SLC6A1/SYN1/SNAP91/SYT1/SYT5/UNC13A/UNC13B/UNC13C
glutamate secretion	9	6.4	PPFIA2/RAB3A/APBA1/SLC1A2/SYT1/STX1A/STXBP1/UNC13B/VAMP2
regulation of cardiac conduction	11	5.8	ATP1A3/ATP1B1/ATP1B2/ATP2B1/ATP2B2/EHD3/FXYD6/PRKACA/SLC8A1/SLC8A2/TMEM65
learning	11	5.7	ATP8A1/ELAVL4/NRXN1/NRXN3/PRKAR2B/PPP1R9B/SLC6A1/SLC8A2/SORCS3/SYNJ1/TH
ion transmembrane transport	20	5.6	ATP6V0A1/ATP6V1G2/ATP1A3/ATP1B1/ATP1B2/ATP8A1/ATP2B1/ATP2B2/FXYD6/ANO8/GABRA1/GABRA2/GABRA3/GABRA5/GABRB3/NEDD4L/NCALD/SFXN1/SFXN3/TTYH1
synapse assembly	11	5.4	ADGRL3/CLSTN3/DNER/DNM3/GPM6A/NRXN1/NRXN2/NRXN3/NLGN1/NLGN2/NRCAM

Supplementary Table S3. (a) N-glycopeptides with increased numbers in the NSCs (>2 fold)

Gene Name	Protein name	Sequence	Glycan Composition	Abundance Ratio: (NSC) / (iPSC)	Abundance Ratio p-value: (NSC) / (iPSC)
A0FGR8	Isoform 2 of Extended synaptotagmin-2	ENLSPK	HexNAc(4)Hex(6)	100	1.00.E-17
A0MZ66	Isoform 3 of Shootin-1	LNKENK	HexNAc(3)Hex(6)Fuc(1)	100	1.00.E-17
A1L157	Tetraspanin-11	QHLNR	HexNAc(5)Hex(5)Fuc(1)NeuAc(1)	100	1.00.E-17
A1L157	Tetraspanin-11	QHLNR	HexNAc(5)Hex(4)Fuc(2)	100	1.00.E-17
A1L157	Tetraspanin-11	QHLNR	HexNAc(5)Hex(4)	100	1.00.E-17
A1L157	Tetraspanin-11	QHLNR	HexNAc(4)Hex(5)NeuAc(1)	100	1.00.E-17
A1L157	Tetraspanin-11	QHLNR	HexNAc(4)Hex(5)Fuc(2)NeuAc(1)	100	1.00.E-17
A4D0S4	Laminin subunit beta-4	RQNDSLDK	HexNAc(5)Hex(4)Fuc(2)	100	1.00.E-17
A4D1P6	WD repeat-containing protein 91	NASLSQSPR	HexNAc(6)Hex(4)	100	1.00.E-17
A6NE02	BTB/POZ domain-containing protein 17	QGNASDVVLR	HexNAc(2)Hex(9)	14.293	1.39.E-04
A6NE02	BTB/POZ domain-containing protein 17	QGNASDVVLR	HexNAc(2)Hex(8)	100	1.00.E-17
A6NE02	BTB/POZ domain-containing protein 17	FFDVNGSAFLPR	HexNAc(2)Hex(9)	8.067	7.42.E-03
A6NGN9	IgLON family member 5	VAWLNR	HexNAc(2)Hex(8)	8.77	1.94.E-04
A6NGN9	IgLON family member 5	HYGNYTCR	HexNAc(4)Hex(5)Fuc(3)	5.729	4.01.E-02
A6NGN9	IgLON family member 5	VAWLNR	HexNAc(2)Hex(7)	3.527	8.59.E-04
A8K979	ERI1 exoribonuclease 2	NLSISTK	HexNAc(5)Hex(5)Fuc(1)	100	1.00.E-17
A8K979	ERI1 exoribonuclease 2	NLSISTK	HexNAc(4)Hex(5)	100	1.00.E-17
A8K979	ERI1 exoribonuclease 2	NLSISTK	HexNAc(5)Hex(5)Fuc(2)	4.452	4.66.E-02
A8K979	ERI1 exoribonuclease 2	NLSISTK	HexNAc(2)Hex(6)	100	1.00.E-17
A8MVW0	Protein FAM171A2	ASVDVFGNR	HexNAc(2)Hex(7)	100	1.00.E-17
A8MVW0	Protein FAM171A2	NGTGVIR	HexNAc(2)Hex(7)	35.687	2.38.E-06
A8MVW0	Protein FAM171A2	ASVDVFGNR	HexNAc(2)Hex(5)	11.26	2.63.E-05
A8MVW0	Protein FAM171A2	NGTGVIR	HexNAc(2)Hex(6)	10.205	7.34.E-04
A8MVW0	Protein FAM171A2	ASVDVFGNR	HexNAc(3)Hex(6)Fuc(1)	5.971	1.83.E-02
A8MVW0	Protein FAM171A2	NGTGVIRK	HexNAc(2)Hex(7)	100	1.00.E-17
A8MVW0	Protein FAM171A2	NGTGVIR	HexNAc(2)Hex(8)	5.989	3.65.E-02
A8MVW0	Protein FAM171A2	ASVDVFGNR	HexNAc(4)Hex(5)Fuc(1)	3.649	4.98.E-02
O00469	Procollagen-lysine,2-oxoglutarate 5-dioxygenase 2	YFNYTVK	HexNAc(2)Hex(8)	100	1.00.E-17
O14525	Astrotactin-1	AAPIYELVTNNQTQR	HexNAc(2)Hex(8)	100	1.00.E-17
O14672	Disintegrin and metalloproteinase domain-containing protein 10	NISFMVK	HexNAc(2)Hex(7)	100	1.00.E-17
O14786	Neuropilin-1	RGPECSQNYTTPSGVIK	HexNAc(4)Hex(5)NeuAc(1)	100	1.00.E-17
O14786	Neuropilin-1	RGPECSQNYTTPSGVIK	HexNAc(4)Hex(5)Fuc(1)NeuAc(1)	100	1.00.E-17
O14786	Neuropilin-1	RGPECSQNYTTPSGVIK	HexNAc(3)Hex(6)Fuc(1)NeuAc(1)	100	1.00.E-17
O14786	Neuropilin-1	RGPECSQNYTTPSGVIK	HexNAc(3)Hex(5)NeuAc(1)	100	1.00.E-17
O14786	Neuropilin-1	RGPECSQNYTTPSGVIK	HexNAc(3)Hex(4)NeuAc(1)	100	1.00.E-17
O14786	Neuropilin-1	RGPECSQNYTTPSGVIK	HexNAc(3)Hex(6)NeuAc(1)	32.776	1.30.E-08
O14786	Neuropilin-1	IGYSNNGSDWK	HexNAc(4)Hex(5)Fuc(2)NeuAc(1)	10.284	1.80.E-03
O15031	Plexin-B2	SCVAVTSAQPQNMSR	HexNAc(5)Hex(5)Fuc(1)	100	1.00.E-17
O15031	Plexin-B2	SCVAVTSAQPQNMSR	HexNAc(5)Hex(5)	100	1.00.E-17
O15031	Plexin-B2	TEAGAFEYVPDPTFENFTGGVK	HexNAc(3)Hex(6)Fuc(1)	100	1.00.E-17
O15031	Plexin-B2	ALSNISLR	HexNAc(5)Hex(4)Fuc(2)	54.184	1.96.E-08
O15031	Plexin-B2	EAESLQPMTVVGTDYVFHNDTK	HexNAc(2)Hex(7)	18.857	1.86.E-05
O15031	Plexin-B2	TEAGAFEYVPDPTFENFTGGVK	HexNAc(3)Hex(5)NeuAc(1)	18.61	6.70.E-05
O15031	Plexin-B2	LHVTLYNCSFGR	HexNAc(2)Hex(7)	16.762	7.60.E-05
O15031	Plexin-B2	ALSNISLR	HexNAc(5)Hex(5)Fuc(3)	16.106	8.04.E-07
O15031	Plexin-B2	SCVAVTSAQPQNMSR	HexNAc(5)Hex(5)Fuc(2)	14.86	7.92.E-05
O15031	Plexin-B2	LHVTLYNCSFGR	HexNAc(2)Hex(6)	12.125	2.97.E-05
O15031	Plexin-B2	EAESLQPMTVVGTDYVFHNDTK	HexNAc(2)Hex(5)	10.619	5.62.E-05
O15031	Plexin-B2	SCVAVTSAQPQNMSR	HexNAc(2)Hex(6)	10.299	4.72.E-04
O15031	Plexin-B2	EAESLQPMTVVGTDYVFHNDTK	HexNAc(2)Hex(6)	6.595	6.36.E-03
O15031	Plexin-B2	LHVTLYNCSFGR	HexNAc(2)Hex(5)	6.408	4.92.E-03
O15031	Plexin-B2	EAESLQPMTVVGTDYVFHNDTK	HexNAc(2)Hex(9)	4.425	1.92.E-02
O15031	Plexin-B2	SINVTGQGFSLIQR	HexNAc(2)Hex(9)	3.87	9.27.E-04
O15031	Plexin-B2	SINVTGQGFSLIQR	HexNAc(2)Hex(8)	2.666	2.33.E-02
O15031	Plexin-B2	LSHDANETLPLHLYVK	HexNAc(4)Hex(5)Fuc(2)NeuAc(1)	2.383	2.68.E-02
O15031	Plexin-B2	SCVAVTSAQPQNMSR	HexNAc(5)Hex(4)Fuc(1)	100	1.00.E-17
O15031	Plexin-B2	NCSFQPER	HexNAc(4)Hex(5)Fuc(1)NeuAc(2)	100	1.00.E-17
O15031	Plexin-B2	ALSNISLR	HexNAc(2)Hex(7)	100	1.00.E-17
O15031	Plexin-B2	EAESLQPMTVVGTDYVFHNDTK	HexNAc(2)Hex(5)	100	1.00.E-17
O15031	Plexin-B2	ALSNISLR	HexNAc(5)Hex(4)Fuc(1)NeuAc(1)	100	1.00.E-17
O15031	Plexin-B2	ALSNISLR	HexNAc(5)Hex(3)Fuc(1)	100	1.00.E-17
O15031	Plexin-B2	SCVAVTSAQPQNMSR	HexNAc(4)Hex(5)NeuAc(2)	100	1.00.E-17
O15031	Plexin-B2	LSHDANETLPLHLYVK	HexNAc(4)Hex(5)Fuc(2)	100	1.00.E-17
O15031	Plexin-B2	EAESLQPMTVVGTDYVFHNDTK	HexNAc(2)Hex(8)	100	1.00.E-17
O15372	Eukaryotic translation initiation factor 3 subunit H	NTSK	HexNAc(4)Hex(5)NeuAc(1)	100	1.00.E-17
O15394	Neural cell adhesion molecule 2	YNCTATNHIGTR	HexNAc(2)Hex(6)	4.225	2.07.E-02
O15394	Neural cell adhesion molecule 2	NTTNLK	HexNAc(6)Hex(3)Fuc(1)	100	1.00.E-17
O43157	Plexin-B1	LTLNGSK	HexNAc(5)Hex(5)Fuc(3)	100	1.00.E-17
O43157	Plexin-B1	LTLNGSK	HexNAc(5)Hex(4)Fuc(2)	100	1.00.E-17
O43157	Plexin-B1	LTLNGSK	HexNAc(4)Hex(5)Fuc(3)	100	1.00.E-17
O43157	Plexin-B1	YTLDPNITSAGPTK	HexNAc(5)Hex(4)Fuc(2)	7.675	5.51.E-03
O43303	Centriolar coiled-coil protein of 110 kDa	NTSEVK	HexNAc(5)Hex(4)Fuc(2)	14.061	7.34.E-10
O43490	Prominin-1	EALENMNSTLK	HexNAc(5)Hex(5)Fuc(2)	100	1.00.E-17
O43529	Carbohydrate sulfotransferase 10	NLSHTPVSK	HexNAc(2)Hex(9)	12.201	2.48.E-03
O43657	Tetraspanin-6	QYNSTGDYR	HexNAc(5)Hex(5)Fuc(1)NeuAc(1)	100	1.00.E-17
O43657	Tetraspanin-6	QYNSTGDYR	HexNAc(5)Hex(4)Fuc(1)	100	1.00.E-17
O43657	Tetraspanin-6	QYNSTGDYR	HexNAc(4)Hex(4)Fuc(1)NeuAc(1)	100	1.00.E-17
O43657	Tetraspanin-6	QYNSTGDYR	HexNAc(4)Hex(3)Fuc(1)	100	1.00.E-17
O43657	Tetraspanin-6	QYNSTGDYR	HexNAc(3)Hex(4)Fuc(1)NeuAc(1)	17.09	1.77.E-04
O43657	Tetraspanin-6	QYNSTGDYR	HexNAc(4)Hex(5)Fuc(2)NeuAc(1)	5.641	1.48.E-03
O43657	Tetraspanin-6	QYNSTGDYR	HexNAc(5)Hex(4)Fuc(1)NeuAc(1)	100	1.00.E-17
O43657	Tetraspanin-6	QYNSTGDYR	HexNAc(5)Hex(5)Fuc(3)	100	1.00.E-17
O43852	Isoform 3 of Calumenin	NATYGYVLDDPDPDDGFNYK	HexNAc(5)Hex(4)	100	1.00.E-17
O60242	Adhesion G protein-coupled receptor B3	NVTDTFK	HexNAc(2)Hex(5)	100	1.00.E-17
O60245	Isoform B of Protocadherin-7	NISYTLLPPSSNVR	HexNAc(2)Hex(5)	100	1.00.E-17
O60486	Plexin-C1	TASTIANSSK	HexNAc(2)Hex(8)	100	1.00.E-17
O60486	Plexin-C1	TNVTVK	HexNAc(2)Hex(7)	100	1.00.E-17
O60486	Plexin-C1	TNVTVK	HexNAc(2)Hex(6)	100	1.00.E-17
O60568	Procollagen-lysine,2-oxoglutarate 5-dioxygenase 3	SAEFFNYTVR	HexNAc(2)Hex(6)	6.167	5.50.E-03
O60568	Procollagen-lysine,2-oxoglutarate 5-dioxygenase 3	SAEFFNYTVR	HexNAc(2)Hex(8)	5.757	5.92.E-03
O60568	Procollagen-lysine,2-oxoglutarate 5-dioxygenase 3	SAEFFNYTVR	HexNAc(2)Hex(7)	5.133	2.54.E-02
O60568	Procollagen-lysine,2-oxoglutarate 5-dioxygenase 3	EQYIHENYSR	HexNAc(4)Hex(3)Fuc(1)	100	1.00.E-17
O60637	Tetraspanin-3	NQSVPLSCCR	HexNAc(3)Hex(6)	100	1.00.E-17
O60637	Tetraspanin-3	NQSVPLSCCR	HexNAc(2)Hex(4)	100	1.00.E-17
O60637	Tetraspanin-3	NQSVPLSCCR	HexNAc(3)Hex(6)Fuc(1)	32.012	3.69.E-08
O60637	Tetraspanin-3	NQSVPLSCCR	HexNAc(2)Hex(5)	13.478	4.09.E-08
O60637	Tetraspanin-3	NQSVPLSCCR	HexNAc(3)Hex(6)NeuAc(1)	3.351	4.02.E-02
O60637	Tetraspanin-3	NQSVPLSCCR	HexNAc(3)Hex(5)Fuc(1)	2.725	3.47.E-02
O60637	Tetraspanin-3	NQSVPLSCCR	HexNAc(3)Hex(6)Fuc(1)NeuAc(1)	100	1.00.E-17
O60716	Catenin delta-1	NISFGR	HexNAc(2)Hex(7)	7.086	1.16.E-02
O75051	Plexin-A2	EHYLSSVNK	HexNAc(2)Hex(8)	100	1.00.E-17

O75051	Plexin-A2	EHYLSSVNK	HexNAc(2)Hex(7)	100	1.00.E-17
O75051	Plexin-A2	EHYLSSVNK	HexNAc(2)Hex(6)	8.183	6.10.E-03
O75054	Isoform 2 of Immunoglobulin superfamily member 3	VQGNSTLLHITDLQAR	HexNAc(5)Hex(3)Fuc(1)	100	1.00.E-17
O75054	Isoform 2 of Immunoglobulin superfamily member 3	VQGNSTLLHITDLQAR	HexNAc(2)Hex(5)	100	1.00.E-17
O75054	Isoform 2 of Immunoglobulin superfamily member 3	NYNNTWTR	HexNAc(2)Hex(5)	8.606	1.91.E-03
O75054	Isoform 2 of Immunoglobulin superfamily member 3	NYNNTWTR	HexNAc(2)Hex(7)	100	1.00.E-17
O75054	Isoform 2 of Immunoglobulin superfamily member 3	TLTLVENKPIQLNCSVK	HexNAc(2)Hex(7)	20.026	5.12.E-07
O75054	Isoform 2 of Immunoglobulin superfamily member 3	TLTLVENKPIQLNCSVK	HexNAc(2)Hex(8)	8.711	1.36.E-03
O75054	Isoform 2 of Immunoglobulin superfamily member 3	LSQAQGNLSVLETR	HexNAc(6)Hex(3)Fuc(1)	100	1.00.E-17
O75054	Isoform 2 of Immunoglobulin superfamily member 3	NYNNTWTR	HexNAc(2)Hex(6)	100	1.00.E-17
O75093	Slit homolog 1 protein	GLEVLTLNNNNITTIPVSSFNHMPK	HexNAc(2)Hex(5)	100	1.00.E-17
O75116	Rho-associated protein kinase 2	NLTIK	HexNAc(4)Hex(7)	100	1.00.E-17
O75443	Alpha-tectorin	NASYK	HexNAc(7)Hex(3)Fuc(1)	8.833	4.90.E-03
O75503	Ceroid-lipofuscinosis neuronal protein 5	NIETNYTR	HexNAc(2)Hex(5)	14.965	1.15.E-03
O75503	Ceroid-lipofuscinosis neuronal protein 5	QDNETGIYYETWNVK	HexNAc(2)Hex(9)	2.683	2.35.E-02
O75533	Splicing factor 3B subunit 1	NGTPPMR	HexNAc(4)Hex(3)Fuc(1)	2.654	1.05.E-02
O75718	Cartilage-associated protein	NCSAAPQPEPAAGLASYPELR	HexNAc(2)Hex(9)	4.614	8.66.E-04
O75882	Attractin	NHSCSEGQISIFR	HexNAc(5)Hex(5)Fuc(3)	100	1.00.E-17
O75882	Attractin	IDSTGNVTNELR	HexNAc(2)Hex(8)	4.044	2.23.E-02
O75882	Attractin	NHSCSEGQISIFR	HexNAc(2)Hex(8)	4.543	3.85.E-02
O75970	Multiple PDZ domain protein	NGTLK	HexNAc(3)Hex(3)Fuc(2)	100	1.00.E-17
O75976	Carboxypeptidase D	NNSNNFDLNR	HexNAc(4)Hex(6)Fuc(2)	100	1.00.E-17
O75976	Carboxypeptidase D	NVTVK	HexNAc(4)Hex(4)Fuc(2)	100	1.00.E-17
O75976	Carboxypeptidase D	NNSNNFDLNR	HexNAc(2)Hex(5)	100	1.00.E-17
O75976	Carboxypeptidase D	NNSNNFDLNR	HexNAc(3)Hex(6)Fuc(1)	20.307	2.68.E-05
O75976	Carboxypeptidase D	NVTVK	HexNAc(5)Hex(5)Fuc(3)	7.741	5.07.E-03
O75976	Carboxypeptidase D	NVTVK	HexNAc(5)Hex(4)Fuc(2)	4.508	2.57.E-02
O75976	Carboxypeptidase D	NVTVK	HexNAc(5)Hex(3)Fuc(1)	100	1.00.E-17
O75976	Carboxypeptidase D	HIWSLEISNKPNVSEPEEPK	HexNAc(5)Hex(5)Fuc(3)	17.421	2.29.E-04
O75976	Carboxypeptidase D	NVTVK	HexNAc(4)Hex(5)Fuc(3)	5.978	1.54.E-02
O75976	Carboxypeptidase D	NVTVK	HexNAc(4)Hex(3)Fuc(1)	100	1.00.E-17
O94779	Contactin-5	ILNASK	HexNAc(2)Hex(5)	100	1.00.E-17
O94806	Serine/threonine-protein kinase D3	IPNNCSGVR	HexNAc(4)Hex(5)Fuc(2)	100	1.00.E-17
O94813	Slit homolog 2 protein	ITCVGNDSFIGLSSVR	HexNAc(5)Hex(4)Fuc(1)NeuAc(1)	100	1.00.E-17
O94813	Slit homolog 2 protein	LDLNGNNITR	HexNAc(2)Hex(8)	100	1.00.E-17
O94856	Neurofascin	VILYNR	HexNAc(2)Hex(8)	100	1.00.E-17
O94856	Neurofascin	YVAFNGTK	HexNAc(2)Hex(5)	9.306	3.99.E-03
O94856	Neurofascin	VILYNR	HexNAc(2)Hex(7)	8.52	5.00.E-03
O94874	E3 UFM1-protein ligase 1	PINK	HexNAc(4)Hex(5)Fuc(2)	100	1.00.E-17
O95297	Myelin protein zero-like protein 1	DASINIENMQFIHNGTYICDVK	HexNAc(3)Hex(5)NeuAc(1)	100	1.00.E-17
O95297	Myelin protein zero-like protein 1	EIFVANGTQGK	HexNAc(4)Hex(5)Fuc(2)NeuAc(1)	3.928	2.29.E-02
O95490	Isoform 5 of Adhesion G protein-coupled receptor L2	LVDTNK	HexNAc(2)Hex(7)	4.809	3.61.E-02
O95490	Isoform 5 of Adhesion G protein-coupled receptor L2	LVDTNK	HexNAc(2)Hex(8)	5.054	3.56.E-02
O95674	Phosphatidate cytidylyltransferase 2	CFVCPVEYNNDTNSFTVDCEPSDLFR	HexNAc(2)Hex(6)	5.122	2.81.E-02
O95754	Semaphorin-4F	TEVTQVNTTNCGR	HexNAc(2)Hex(6)	100	1.00.E-17
O95970	Isoform 2 of Leucine-rich glioma-inactivated protein 1	NYDNITVLR	HexNAc(2)Hex(9)	100	1.00.E-17
P02458	Collagen alpha-1(II) chain	LLSTEGSQNITYHCK	HexNAc(2)Hex(9)	21.594	5.95.E-05
P02458	Collagen alpha-1(II) chain	LLSTEGSQNITYHCK	HexNAc(2)Hex(8)	11.039	2.80.E-04
P02786	Transferrin receptor protein 1	QNNGAFNETLFR	HexNAc(2)Hex(7)	100	1.00.E-17
P04216	Thy-1 membrane glycoprotein	TNFTSK	HexNAc(5)Hex(5)Fuc(3)	9.191	3.52.E-03
P04216	Thy-1 membrane glycoprotein	TNFTSK	HexNAc(3)Hex(6)Fuc(1)	4.772	3.06.E-02
P04216	Thy-1 membrane glycoprotein	TNFTSK	HexNAc(5)Hex(5)Fuc(1)	100	1.00.E-17
P04844	Dolichyl-diphosphooligosaccharide--protein glycosyltransferase subunit 2	SNLDPSNVDSLFYAAQASQALSGCEISISNETK	HexNAc(2)Hex(6)	2.093	4.69.E-02
P05026	Sodium/potassium-transporting ATPase subunit beta-1	LEWLGNCSGLNDETYGYK	HexNAc(5)Hex(5)Fuc(1)NeuAc(2)	100	1.00.E-17
P05026	Sodium/potassium-transporting ATPase subunit beta-1	NESLETYPVMK	HexNAc(6)Hex(7)NeuAc(2)	100	1.00.E-17
P05026	Sodium/potassium-transporting ATPase subunit beta-1	NESLETYPVMK	HexNAc(6)Hex(7)Fuc(4)	100	1.00.E-17
P05026	Sodium/potassium-transporting ATPase subunit beta-1	NESLETYPVMK	HexNAc(2)Hex(5)	100	1.00.E-17
P05155	Isoform 3 of Plasma protease C1 inhibitor	MLFVEPILEVSSLPTTNSTTNSATK	HexNAc(2)Hex(8)	5.799	2.71.E-02
P05155	Isoform 3 of Plasma protease C1 inhibitor	VLSNNSDANLELINTWVAK	HexNAc(2)Hex(8)	3.502	3.23.E-02
P05156	Complement factor I	LISNCSK	HexNAc(2)Hex(9)	100	1.00.E-17
P05156	Complement factor I	LISNCSK	HexNAc(2)Hex(8)	100	1.00.E-17
P05186	Alkaline phosphatase, tissue-nonspecific isozyme	CNTTQGNEVTSILR	HexNAc(5)Hex(5)Fuc(3)	100	1.00.E-17
P05556	Isoform 5 of Integrin beta-1	NPCTSEQNCTSPFSYK	HexNAc(5)Hex(5)Fuc(3)	100	1.00.E-17
P05556	Isoform 5 of Integrin beta-1	LRNPCTSEQNCTSPFSYK	HexNAc(5)Hex(5)Fuc(3)	100	1.00.E-17
P05556	Isoform 5 of Integrin beta-1	DTCTQECSYFNITK	HexNAc(5)Hex(4)Fuc(2)	100	1.00.E-17
P05556	Isoform 5 of Integrin beta-1	KDTCTQECSYFNITK	HexNAc(5)Hex(4)Fuc(2)	100	1.00.E-17
P05556	Isoform 5 of Integrin beta-1	DTCTQECSYFNITK	HexNAc(4)Hex(5)Fuc(2)NeuAc(1)	4.197	8.49.E-03
P05556	Isoform 5 of Integrin beta-1	LRNPCTSEQNCTSPFSYK	HexNAc(6)Hex(7)Fuc(1)NeuAc(1)	100	1.00.E-17
P05556	Isoform 5 of Integrin beta-1	LRNPCTSEQNCTSPFSYK	HexNAc(5)Hex(6)Fuc(1)	100	1.00.E-17
P05556	Isoform 5 of Integrin beta-1	NVTR	HexNAc(5)Hex(5)Fuc(3)	100	1.00.E-17
P05556	Isoform 5 of Integrin beta-1	NVTNR	HexNAc(5)Hex(5)Fuc(3)	100	1.00.E-17
P05556	Isoform 5 of Integrin beta-1	NPCTSEQNCTSPFSYK	HexNAc(5)Hex(5)Fuc(1)NeuAc(1)	100	1.00.E-17
P05556	Isoform 5 of Integrin beta-1	LRNPCTSEQNCTSPFSYK	HexNAc(4)Hex(5)Fuc(1)	100	1.00.E-17
P05556	Isoform 5 of Integrin beta-1	LRNPCTSEQNCTSPFSYK	HexNAc(7)Hex(7)Fuc(1)	100	1.00.E-17
P05556	Isoform 5 of Integrin beta-1	NPCTSEQNCTSPFSYK	HexNAc(5)Hex(6)Fuc(2)	100	1.00.E-17
P05556	Isoform 5 of Integrin beta-1	NVTR	HexNAc(5)Hex(4)Fuc(1)	100	1.00.E-17
P06756	Integrin alpha-V	ENQNHSYSLK	HexNAc(5)Hex(6)Fuc(1)	100	1.00.E-17
P06756	Integrin alpha-V	ENQNHSYSLK	HexNAc(4)Hex(5)Fuc(2)	100	1.00.E-17
P06756	Integrin alpha-V	ENQNHSYSLK	HexNAc(3)Hex(6)Fuc(1)	100	1.00.E-17
P06756	Integrin alpha-V	ANTTQPGIVEGGQVLK	HexNAc(2)Hex(7)	100	1.00.E-17
P06756	Integrin alpha-V	NMTISR	HexNAc(5)Hex(5)Fuc(3)	18.364	8.91.E-07
P06756	Integrin alpha-V	ANTTQPGIVEGGQVLK	HexNAc(3)Hex(6)Fuc(1)	13.665	1.23.E-03
P06756	Integrin alpha-V	ANTTQPGIVEGGQVLK	HexNAc(2)Hex(6)	9.653	1.56.E-03
P06756	Integrin alpha-V	ANTTQPGIVEGGQVLK	HexNAc(2)Hex(5)	7.491	4.86.E-04
P06756	Integrin alpha-V	ENQNHSYSLK	HexNAc(2)Hex(5)	5.435	1.41.E-02
P06756	Integrin alpha-V	ANTTQPGIVEGGQVLK	HexNAc(4)Hex(5)Fuc(1)	4.168	1.85.E-02
P06756	Integrin alpha-V	NMTISR	HexNAc(5)Hex(5)Fuc(1)	100	1.00.E-17
P06756	Integrin alpha-V	ENQNHSYSLK	HexNAc(4)Hex(5)Fuc(3)	100	1.00.E-17
P06756	Integrin alpha-V	ANTTQPGIVEGGQVLK	HexNAc(2)Hex(8)	5.067	9.89.E-03
P06756	Integrin alpha-V	ANTTQPGIVEGGQVLK	HexNAc(3)Hex(6)	100	1.00.E-17
P06756	Integrin alpha-V	ENQNHSYSLK	HexNAc(3)Hex(4)Fuc(1)	100	1.00.E-17
P07195	L-lactate dehydrogenase B chain	NISR	HexNAc(7)Hex(3)	55.647	3.31.E-07
P07602	Isoform Sap-mu-9 of Prosaposin	TCDWLPKPNMSASCK	HexNAc(2)Hex(3)	3.437	3.32.E-02
P07602	Isoform Sap-mu-9 of Prosaposin	TCDWLPKPNMSASCK	HexNAc(4)Hex(3)Fuc(1)	6.805	2.23.E-02
P07602	Isoform Sap-mu-9 of Prosaposin	TCDWLPKPNMSASCK	HexNAc(2)Hex(5)	100	1.00.E-17
P08069	Insulin-like growth factor 1 receptor	DVMQVANTTMSSR	HexNAc(2)Hex(8)	100	1.00.E-17
P08069	Insulin-like growth factor 1 receptor	WNPPSLPNGNLSYYIVR	HexNAc(2)Hex(5)	5.436	4.43.E-02
P08069	Insulin-like growth factor 1 receptor	LNPGNYTAR	HexNAc(2)Hex(5)	3.609	3.62.E-02
P08648	Integrin alpha-5	VTGLNCTTNHPINPK	HexNAc(5)Hex(4)Fuc(2)	100	1.00.E-17
P08648	Integrin alpha-5	VTGLNCTTNHPINPK	HexNAc(5)Hex(5)Fuc(3)	68.151	3.52.E-11
P08648	Integrin alpha-5	VTGLNCTTNHPINPK	HexNAc(5)Hex(5)NeuAc(2)	9.081	8.93.E-04

P08648	Integrin alpha-5	VTGLNCTTNHPINPK	HexNAc(4)Hex(5)Fuc(2)NeuAc(1)	4.801	1.88.E-02
P08648	Integrin alpha-5	VTGLNCTTNHPINPK	HexNAc(4)Hex(5)Fuc(1)NeuAc(2)	100	1.00.E-17
P08648	Integrin alpha-5	TEKEPLSDPVGTCYLSTDNFTR	HexNAc(5)Hex(4)Fuc(2)	5.386	1.88.E-02
P08962	CD63 antigen	NNHTASILDR	HexNAc(6)Hex(6)Fuc(1)NeuAc(2)	100	1.00.E-17
P08962	CD63 antigen	NNHTASILDR	HexNAc(5)Hex(5)Fuc(3)	100	1.00.E-17
P08962	CD63 antigen	NNHTASILDR	HexNAc(5)Hex(5)Fuc(2)	100	1.00.E-17
P08962	CD63 antigen	NNHTASILDR	HexNAc(5)Hex(4)Fuc(2)	100	1.00.E-17
P08962	CD63 antigen	NNHTASILDR	HexNAc(5)Hex(6)NeuAc(3)	10.698	3.16.E-05
P08962	CD63 antigen	NNHTASILDR	HexNAc(3)Hex(6)Fuc(1)NeuAc(1)	10.546	5.98.E-04
P08962	CD63 antigen	NNHTASILDR	HexNAc(2)Hex(5)	4.859	1.16.E-02
P08962	CD63 antigen	NNHTASILDR	HexNAc(4)Hex(5)Fuc(2)	3.178	4.64.E-02
P08962	CD63 antigen	NNHTASILDR	HexNAc(4)Hex(5)Fuc(2)NeuAc(1)	2.588	4.20.E-02
P08962	CD63 antigen	NNHTASILDR	HexNAc(3)Hex(6)Fuc(1)	100	1.00.E-17
P09960	Isoform 4 of Leukotriene A-4 hydrolase	NLSKR	HexNAc(3)Hex(6)NeuAc(1)	100	1.00.E-17
P0C7U0	Protein ELFN1	MYTLEHFNNSK	HexNAc(2)Hex(7)	7.121	1.37.E-02
P0C7U0	Protein ELFN1	MYTLEHFNNSK	HexNAc(2)Hex(6)	100	1.00.E-17
P0C7U0	Protein ELFN1	MYTLEHFNNSK	HexNAc(2)Hex(5)	100	1.00.E-17
P10253	Lysosomal alpha-glucosidase	QVVENMTR	HexNAc(2)Hex(6)	100	1.00.E-17
P10909	Isoform 2 of Clusterin	HNSTGCLR	HexNAc(2)Hex(8)	4.199	4.51.E-02
P11117	Lysosomal acid phosphatase	NLTLMATTSQLPK	HexNAc(2)Hex(9)	3.549	1.40.E-02
P11117	Lysosomal acid phosphatase	NESDKAPWPLSLPGCPHR	HexNAc(2)Hex(7)	9.723	6.20.E-03
P11117	Lysosomal acid phosphatase	YHGFLNTSYHR	HexNAc(6)Hex(6)Fuc(1)NeuAc(2)	100	1.00.E-17
P11142	Heat shock cognate 71 kDa protein	NTTIPTK	HexNAc(4)Hex(6)	100	1.00.E-17
P11279	Lysosome-associated membrane glycoprotein 1	KDNTTVTR	HexNAc(5)Hex(5)Fuc(3)	15.303	1.82.E-04
P11279	Lysosome-associated membrane glycoprotein 1	LLNINPNK	HexNAc(3)Hex(6)Fuc(1)	11.066	4.60.E-05
P11279	Lysosome-associated membrane glycoprotein 1	SSCGKENTSDPSLVIAFGR	HexNAc(5)Hex(4)Fuc(1)	7.127	5.95.E-03
P11279	Lysosome-associated membrane glycoprotein 1	LLNINPNK	HexNAc(5)Hex(4)Fuc(2)	4.104	3.05.E-02
P11279	Lysosome-associated membrane glycoprotein 1	LLNINPNK	HexNAc(4)Hex(5)Fuc(1)	3.575	3.90.E-02
P11279	Lysosome-associated membrane glycoprotein 1	LLNINPNK	HexNAc(2)Hex(5)	2.05	1.79.E-02
P11279	Lysosome-associated membrane glycoprotein 1	LLNINPNK	HexNAc(3)Hex(5)Fuc(1)	3.407	4.31.E-02
P11279	Lysosome-associated membrane glycoprotein 1	SSCGKENTSDPSLVIAFGR	HexNAc(4)Hex(5)Fuc(1)	100	1.00.E-17
P11279	Lysosome-associated membrane glycoprotein 1	LLNINPNK	HexNAc(4)Hex(5)	100	1.00.E-17
P11532	Isoform 2 of Dystrophin	AQNVTR	HexNAc(2)Hex(5)	10.348	2.14.E-03
P11717	Cation-independent mannose-6-phosphate receptor	GYPCGGNK	HexNAc(5)Hex(5)Fuc(3)	100	1.00.E-17
P11717	Cation-independent mannose-6-phosphate receptor	NGSSIVDLSPLIHR	HexNAc(3)Hex(6)Fuc(1)	100	1.00.E-17
P11717	Cation-independent mannose-6-phosphate receptor	SLLEFNTTVSCDQQGTNHR	HexNAc(5)Hex(5)Fuc(3)	100	1.00.E-17
P11717	Cation-independent mannose-6-phosphate receptor	SLLEFNTTVSCDQQGTNHR	HexNAc(5)Hex(4)Fuc(2)	5.215	2.38.E-02
P11717	Cation-independent mannose-6-phosphate receptor	SLLEFNTTVSCDQQGTNHR	HexNAc(4)Hex(5)Fuc(2)	4.672	2.79.E-02
P12107	Isoform B of Collagen alpha-1(XI) chain	VYCNFTSGGETCIYPDKK	HexNAc(2)Hex(9)	100	1.00.E-17
P12107	Isoform B of Collagen alpha-1(XI) chain	VYCNFTSGGETCIYPDK	HexNAc(2)Hex(6)	100	1.00.E-17
P12107	Isoform B of Collagen alpha-1(XI) chain	VYCNFTSGGETCIYPDKK	HexNAc(2)Hex(8)	8.156	7.65.E-03
P12109	Collagen alpha-1(VI) chain	GAPGINGTK	HexNAc(2)Hex(8)	100	1.00.E-17
P12109	Collagen alpha-1(VI) chain	GAPGINGTK	HexNAc(2)Hex(9)	100	1.00.E-17
P12109	Collagen alpha-1(VI) chain	GAPGINGTK	HexNAc(2)Hex(6)	100	1.00.E-17
P13473	Isoform LAMP-2C of Lysosome-associated membrane glycoprotein 2	LNSSTIK	HexNAc(6)Hex(6)	100	1.00.E-17
P13473	Isoform LAMP-2C of Lysosome-associated membrane glycoprotein 2	LNSSTIK	HexNAc(5)Hex(6)Fuc(2)NeuAc(1)	100	1.00.E-17
P13473	Isoform LAMP-2C of Lysosome-associated membrane glycoprotein 2	LNSSTIK	HexNAc(5)Hex(4)Fuc(1)	100	1.00.E-17
P13473	Isoform LAMP-2C of Lysosome-associated membrane glycoprotein 2	LNSSTIK	HexNAc(5)Hex(4)	100	1.00.E-17
P13473	Isoform LAMP-2C of Lysosome-associated membrane glycoprotein 2	VASVININPNTTHSTGSCR	HexNAc(4)Hex(5)Fuc(3)	100	1.00.E-17
P13473	Isoform LAMP-2C of Lysosome-associated membrane glycoprotein 2	VQPFNVTQGK	HexNAc(3)Hex(6)Fuc(1)	100	1.00.E-17
P13473	Isoform LAMP-2C of Lysosome-associated membrane glycoprotein 2	LNSSTIK	HexNAc(5)Hex(5)Fuc(1)NeuAc(1)	7.553	1.13.E-02
P13473	Isoform LAMP-2C of Lysosome-associated membrane glycoprotein 2	VASVININPNTTHSTGSCR	HexNAc(3)Hex(6)Fuc(1)NeuAc(1)	4.381	3.17.E-02
P13473	Isoform LAMP-2C of Lysosome-associated membrane glycoprotein 2	LNSSTIK	HexNAc(4)Hex(5)Fuc(1)NeuAc(1)	3.866	4.96.E-02
P13473	Isoform LAMP-2C of Lysosome-associated membrane glycoprotein 2	VQPFNVTQGK	HexNAc(2)Hex(5)	3.273	3.22.E-03
P13473	Isoform LAMP-2C of Lysosome-associated membrane glycoprotein 2	VQPFNVTQGK	HexNAc(2)Hex(7)	2.147	4.74.E-02
P13473	Isoform LAMP-2C of Lysosome-associated membrane glycoprotein 2	LNSSTIK	HexNAc(6)Hex(6)Fuc(1)	100	1.00.E-17
P13473	Isoform LAMP-2C of Lysosome-associated membrane glycoprotein 2	LNSSTIK	HexNAc(5)Hex(5)Fuc(1)	100	1.00.E-17
P13473	Isoform LAMP-2C of Lysosome-associated membrane glycoprotein 2	VASVININPNTTHSTGSCR	HexNAc(5)Hex(6)Fuc(2)NeuAc(1)	100	1.00.E-17
P13674	Prolyl 4-hydroxylase subunit alpha-1	DMSDGFISNLTIQR	HexNAc(2)Hex(9)	4.543	1.65.E-02
P13674	Prolyl 4-hydroxylase subunit alpha-1	DMSDGFISNLTIQR	HexNAc(2)Hex(9)	2.43	1.48.E-02
P13987	CD59 glycoprotein	TAVNCSSDFDACLITK	HexNAc(5)Hex(5)Fuc(3)	100	1.00.E-17
P13987	CD59 glycoprotein	TAVNCSSDFDACLITK	HexNAc(5)Hex(5)Fuc(1)NeuAc(1)	100	1.00.E-17
P13987	CD59 glycoprotein	TAVNCSSDFDACLITK	HexNAc(5)Hex(5)NeuAc(2)	10.324	9.04.E-04
P13987	CD59 glycoprotein	TAVNCSSDFDACLITK	HexNAc(5)Hex(4)Fuc(1)	100	1.00.E-17
P14384	Carboxypeptidase M	TVAQNYSSVTHLHSIGK	HexNAc(4)Hex(5)Fuc(1)NeuAc(1)	100	1.00.E-17
P14415	Sodium/potassium-transporting ATPase subunit beta-2	FHVNYTQPLVAVK	HexNAc(2)Hex(8)	17.478	2.68.E-05
P14415	Sodium/potassium-transporting ATPase subunit beta-2	FLEPYNDSIQAQK	HexNAc(2)Hex(7)	10.146	4.50.E-04
P14415	Sodium/potassium-transporting ATPase subunit beta-2	FHVNYTQPLVAVK	HexNAc(2)Hex(7)	7.574	2.77.E-04
P14415	Sodium/potassium-transporting ATPase subunit beta-2	TQLGNCSGIGDSTHYGYSTGQPCVFIK	HexNAc(2)Hex(5)	5.237	1.29.E-02
P14415	Sodium/potassium-transporting ATPase subunit beta-2	FLEPYNDSIQAQK	HexNAc(2)Hex(5)	4.074	2.46.E-02
P14415	Sodium/potassium-transporting ATPase subunit beta-2	TQLGNCSGIGDSTHYGYSTGQPCVFIK	HexNAc(2)Hex(6)	4.175	4.08.E-02
P14415	Sodium/potassium-transporting ATPase subunit beta-2	FLEPYNDSIQAQK	HexNAc(2)Hex(6)	3.13	4.53.E-02
P14625	Endoplasmin	TDDEVVQREEEAIQLDGLNASQIR	HexNAc(2)Hex(6)	5.447	1.68.E-03
P14625	Endoplasmin	TDDEVVQREEEAIQLDGLNASQIR	HexNAc(2)Hex(7)	4.92	1.10.E-02
P14625	Endoplasmin	GVVDSDDLPLNVSR	HexNAc(2)Hex(6)	7.287	2.29.E-02
P15586	N-acetylglucosamine-6-sulfatase	GPGIKPNQTSK	HexNAc(5)Hex(3)	100	1.00.E-17
P16278	Beta-galactosidase	NNVITLNITGK	HexNAc(2)Hex(8)	100	1.00.E-17
P16870	Carboxypeptidase E	GNETIVNLIHSTR	HexNAc(2)Hex(6)	4.101	3.30.E-02
P16870	Carboxypeptidase E	GNETIVNLIHSTR	HexNAc(2)Hex(7)	100	1.00.E-17
P16870	Carboxypeptidase E	GNETIVNLIHSTR	HexNAc(2)Hex(5)	4.541	3.83.E-02
P17050	Alpha-N-acetylgalactosaminidase	MAAALNATGR	HexNAc(2)Hex(5)	100	1.00.E-17
P17301	Integrin alpha-2	TNMSLGLILTR	HexNAc(2)Hex(9)	6.588	4.73.E-03
P17301	Integrin alpha-2	GEYFVNVTTR	HexNAc(2)Hex(7)	5.283	2.28.E-03
P17301	Integrin alpha-2	GEYFVNVTTR	HexNAc(2)Hex(8)	3.779	3.78.E-02
P17301	Integrin alpha-2	LNLQTSTSIPNVTEMK	HexNAc(5)Hex(5)Fuc(3)	11.014	3.81.E-04
P17301	Integrin alpha-2	TASCSNVTCWLK	HexNAc(5)Hex(5)Fuc(3)	100	1.00.E-17
P18084	Integrin beta-5	SNLTVLR	HexNAc(3)Hex(6)NeuAc(1)	100	1.00.E-17
P18084	Integrin beta-5	SNLTVLR	HexNAc(3)Hex(6)Fuc(1)	100	1.00.E-17

P18084	Integrin beta-5	NFTALIPGTTVEILDGDSK	HexNAc(2)Hex(7)	8.963	2.66.E-03
P18084	Integrin beta-5	CHAGYIGDNCNCSTDISTCR	HexNAc(2)Hex(6)	3.696	3.42.E-02
P18084	Integrin beta-5	SNLTVLR	HexNAc(2)Hex(4)	100	1.00.E-17
P18507	Isoform 3 of Gamma-aminobutyric acid receptor subunit gamma-2	FNSTIK	HexNAc(6)Hex(4)Fuc(2)	100	1.00.E-17
P19022	Cadherin-2	SNISILR	HexNAc(2)Hex(7)	100	1.00.E-17
P19022	Cadherin-2	SNISILR	HexNAc(2)Hex(4)	100	1.00.E-17
P19022	Cadherin-2	SNISILR	HexNAc(3)Hex(6)Fuc(1)	27.851	5.48.E-06
P19022	Cadherin-2	NLSLR	HexNAc(6)Hex(4)Fuc(2)	9.021	8.31.E-05
P19022	Cadherin-2	SNISILR	HexNAc(2)Hex(6)	8.772	1.70.E-07
P19022	Cadherin-2	SNISILR	HexNAc(2)Hex(5)	7.756	6.12.E-07
P19022	Cadherin-2	SNISILR	HexNAc(2)Hex(9)	7.276	9.85.E-03
P19022	Cadherin-2	SNISILR	HexNAc(2)Hex(8)	6.739	3.38.E-03
P19022	Cadherin-2	NWTITR	HexNAc(2)Hex(8)	6.36	1.44.E-05
P19022	Cadherin-2	NWTITR	HexNAc(2)Hex(7)	3.743	3.91.E-02
P19022	Cadherin-2	NLSLR	HexNAc(4)Hex(4)Fuc(1)	100	1.00.E-17
P19022	Cadherin-2	NWTITR	HexNAc(2)Hex(6)	100	1.00.E-17
P19022	Cadherin-2	NWTITR	HexNAc(2)Hex(9)	6.357	1.01.E-02
P19022	Cadherin-2	NLSLR	HexNAc(2)Hex(8)	5.088	2.98.E-02
P19022	Cadherin-2	SNISILR	HexNAc(5)Hex(4)Fuc(1)	100	1.00.E-17
P19022	Cadherin-2	RNWTITR	HexNAc(2)Hex(8)	9.94	1.68.E-03
P19022	Cadherin-2	VDIIVANLTVTDK	HexNAc(5)Hex(5)Fuc(3)	7.528	4.11.E-03
P20645	Cation-dependent mannose-6-phosphate receptor	EAGNHTSGAGLVQINK	HexNAc(3)Hex(6)Fuc(1)NeuAc(1)	100	1.00.E-17
P20645	Cation-dependent mannose-6-phosphate receptor	EAGNHTSGAGLVQINK	HexNAc(5)Hex(5)Fuc(3)	98.769	1.46.E-09
P20645	Cation-dependent mannose-6-phosphate receptor	EAGNHTSGAGLVQINK	HexNAc(4)Hex(5)Fuc(3)	3.719	6.21.E-03
P20645	Cation-dependent mannose-6-phosphate receptor	EAGNHTSGAGLVQINK	HexNAc(8)Hex(8)	100	1.00.E-17
P20645	Cation-dependent mannose-6-phosphate receptor	EAGNHTSGAGLVQINK	HexNAc(3)Hex(4)Fuc(2)	100	1.00.E-17
P22681	E3 ubiquitin-protein ligase CBL	NLTK	HexNAc(4)Hex(5)Fuc(4)	100	1.00.E-17
P23142	Fibulin-1	CATPHGDNASLEATFVK	HexNAc(2)Hex(9)	3.701	3.17.E-02
P23142	Fibulin-1	CATPHGDNASLEATFVK	HexNAc(2)Hex(8)	2.978	2.49.E-02
P23229	Integrin alpha-6	YQTLNCSVNVNCVNIR	HexNAc(3)Hex(6)Fuc(1)	100	1.00.E-17
P23246	Splicing factor, proline- and glutamine-rich	ANLSLLR	HexNAc(3)Hex(6)Fuc(1)	11.406	2.20.E-03
P23246	Splicing factor, proline- and glutamine-rich	ANLSLLR	HexNAc(3)Hex(6)NeuAc(1)	100	1.00.E-17
P25391	Laminin subunit alpha-1	DVAGLSQELLNTSASLSR	HexNAc(2)Hex(6)	4.746	3.09.E-02
P25391	Laminin subunit alpha-1	HQVSINNTAVMQR	HexNAc(2)Hex(8)	100	1.00.E-17
P26006	Isoform 2 of Integrin alpha-3	NITIVTGAPR	HexNAc(5)Hex(5)Fuc(2)	100	1.00.E-17
P26006	Isoform 2 of Integrin alpha-3	NITIVTGAPR	HexNAc(2)Hex(6)	9.55	2.34.E-03
P26006	Isoform 2 of Integrin alpha-3	TSIPTINMENK	HexNAc(2)Hex(5)	7.074	1.26.E-02
P26006	Isoform 2 of Integrin alpha-3	SLDAYPILNQAQALENHTEVQFQK	HexNAc(2)Hex(8)	3.632	3.31.E-02
P26006	Isoform 2 of Integrin alpha-3	ELAVPDGYTNR	HexNAc(4)Hex(5)Fuc(1)NeuAc(1)	100	1.00.E-17
P26012	Integrin beta-8	NYAIIKPIGFNETAK	HexNAc(2)Hex(8)	8.764	1.73.E-03
P28907	ADP-ribosyl cyclase/cyclic ADP-ribose hydrolase 1	NSTFGSVEVHNLQPEK	HexNAc(2)Hex(6)	100	1.00.E-17
P29317	Ephrin type-A receptor 2	TASVSINQTEPPK	HexNAc(4)Hex(3)Fuc(1)	7.169	2.89.E-03
P29590	Protein PML	NQSVR	HexNAc(6)Hex(3)Fuc(1)	13.234	3.41.E-04
P30291	Wee1-like protein kinase	KMNR	HexNAc(2)Hex(5)	8.36	9.34.E-03
P32004	Neural cell adhesion molecule L1	VTYQNHNK	HexNAc(5)Hex(3)	100	1.00.E-17
P32004	Neural cell adhesion molecule L1	VPGNQTSTTLK	HexNAc(4)Hex(3)Fuc(1)	4.065	2.68.E-02
P32004	Neural cell adhesion molecule L1	VPGNQTSTTLK	HexNAc(5)Hex(4)Fuc(2)	100	1.00.E-17
P32004	Neural cell adhesion molecule L1	VPGNQTSTTLK	HexNAc(5)Hex(3)Fuc(1)	100	1.00.E-17
P32004	Neural cell adhesion molecule L1	VTYQNHNK	HexNAc(2)Hex(5)	5.2	3.63.E-02
P32004	Neural cell adhesion molecule L1	VTYQNHNK	HexNAc(3)Hex(6)NeuAc(1)	100	1.00.E-17
P32004	Neural cell adhesion molecule L1	GEGNETTNMVITWKPLR	HexNAc(2)Hex(5)	100	1.00.E-17
P32004	Neural cell adhesion molecule L1	THNLTDLSPHLR	HexNAc(5)Hex(3)Fuc(1)	4.392	3.40.E-02
P33981	Dual specificity protein kinase TTK	QTNK	HexNAc(8)Hex(3)	100	1.00.E-17
P35052	Glypican-1	ICPQGYTCCTSEMEENLANR	HexNAc(5)Hex(4)NeuAc(1)	100	1.00.E-17
P35052	Glypican-1	SFDDHFQHLLNDSER	HexNAc(2)Hex(5)	16.97	3.84.E-06
P35052	Glypican-1	ICPQGYTCCTSEMEENLANR	HexNAc(5)Hex(5)Fuc(3)	100	1.00.E-17
P35556	Fibrillin-2	AFNTTK	HexNAc(2)Hex(6)	100	1.00.E-17
P35556	Fibrillin-2	FNLSHLGSK	HexNAc(2)Hex(8)	6.29	5.08.E-03
P35556	Fibrillin-2	ASQDQTMCMDVDECERHPCGNGTCK	HexNAc(2)Hex(8)	100	1.00.E-17
P35580	Isoform 4 of Myosin-10	LNLSSR	HexNAc(4)Hex(3)NeuAc(1)	100	1.00.E-17
P35613	Basigin	ILLTCSLNDSATEVTGHR	HexNAc(5)Hex(5)Fuc(3)	100	1.00.E-17
P35613	Basigin	ILLTCSLNDSATEVTGHR	HexNAc(2)Hex(5)	2.693	3.67.E-03
P35613	Basigin	ILLTCSLNDSATEVTGHR	HexNAc(4)Hex(5)Fuc(3)NeuAc(2)	6.178	1.69.E-02
P35968	Vascular endothelial growth factor receptor 2	NSTFVR	HexNAc(2)Hex(6)	4.903	2.32.E-02
P41217	Isoform 3 of OX-2 membrane glycoprotein	FSEDHLNITCSATAR	HexNAc(4)Hex(6)	100	1.00.E-17
P41217	Isoform 3 of OX-2 membrane glycoprotein	FSEDHLNITCSATAR	HexNAc(4)Hex(5)Fuc(1)	100	1.00.E-17
P41217	Isoform 3 of OX-2 membrane glycoprotein	FSEDHLNITCSATAR	HexNAc(3)Hex(6)NeuAc(1)	9.23	1.05.E-03
P41217	Isoform 3 of OX-2 membrane glycoprotein	FSEDHLNITCSATAR	HexNAc(3)Hex(5)NeuAc(1)	6.297	5.15.E-03
P41217	Isoform 3 of OX-2 membrane glycoprotein	FSEDHLNITCSATAR	HexNAc(3)Hex(6)Fuc(1)	5.686	8.68.E-03
P41217	Isoform 3 of OX-2 membrane glycoprotein	FSEDHLNITCSATAR	HexNAc(4)Hex(5)	5.194	2.01.E-02
P41217	Isoform 3 of OX-2 membrane glycoprotein	FSEDHLNITCSATAR	HexNAc(5)Hex(4)Fuc(1)	4.694	1.64.E-02
P41217	Isoform 3 of OX-2 membrane glycoprotein	FSEDHLNITCSATAR	HexNAc(2)Hex(8)	100	1.00.E-17
P41217	Isoform 3 of OX-2 membrane glycoprotein	FSEDHLNITCSATAR	HexNAc(3)Hex(5)Fuc(1)	8.583	6.63.E-03
P41217	Isoform 3 of OX-2 membrane glycoprotein	FSEDHLNITCSATAR	HexNAc(4)Hex(6)Fuc(1)NeuAc(1)	100	1.00.E-17
P41217	Isoform 3 of OX-2 membrane glycoprotein	FSEDHLNITCSATAR	HexNAc(4)Hex(6)Fuc(1)	100	1.00.E-17
P41217	Isoform 3 of OX-2 membrane glycoprotein	FSEDHLNITCSATAR	HexNAc(2)Hex(5)	100	1.00.E-17
P41217	Isoform 3 of OX-2 membrane glycoprotein	FSEDHLNITCSATAR	HexNAc(4)Hex(6)Fuc(2)	6.683	1.37.E-02
P41218	Myeloid cell nuclear differentiation antigen	IIEIANK	HexNAc(2)Hex(9)	100	1.00.E-17
P41594	Metabotropic glutamate receptor 5	ENPNQTAVIKPFPK	HexNAc(3)Hex(4)	100	1.00.E-17
P42261	Isoform 5 of Glutamate receptor 1	TNYTLHVIEMK	HexNAc(2)Hex(9)	100	1.00.E-17
P42696	RNA-binding protein 34	LKNVSKPK	HexNAc(4)Hex(4)Fuc(2)	13.025	1.20.E-03
P42696	RNA-binding protein 34	LKNVSKPK	HexNAc(4)Hex(4)Fuc(2)NeuAc(1)	100	1.00.E-17
P43121	Cell surface glycoprotein MUC18	CVASVPSIPGLNR	HexNAc(5)Hex(4)	100	1.00.E-17
P43146	Netrin receptor DCC	QQLSNGSLLIQNILHSR	HexNAc(2)Hex(5)	5.48	1.59.E-02
P46940	Ras GTPase-activating-like protein IQGAP1	MTNAKNR	HexNAc(2)Hex(8)	100	1.00.E-17
P48723	Heat shock 70 kDa protein 13	NSTIEAANLAGLK	HexNAc(2)Hex(7)	7.552	8.15.E-03
P48723	Heat shock 70 kDa protein 13	QRNSTIEAANLAGLK	HexNAc(2)Hex(8)	2.837	3.15.E-02
P48723	Heat shock 70 kDa protein 13	QRNSTIEAANLAGLK	HexNAc(2)Hex(7)	100	1.00.E-17
P49746	Thrombospondin-3	CNDTVPEDFEPFRR	HexNAc(2)Hex(9)	100	1.00.E-17
P50454	Serpin H1	SLSNSTAR	HexNAc(2)Hex(4)	5.138	1.12.E-02
P50454	Serpin H1	NVTWK	HexNAc(3)Hex(3)Fuc(1)	100	1.00.E-17
P50895	Basal cell adhesion molecule	TQNFTLLVQGSPELK	HexNAc(2)Hex(9)	100	1.00.E-17
P50897	Palmitoyl-protein thioesterase 1	NHSIFLADINQER	HexNAc(4)Hex(3)Fuc(1)	100	1.00.E-17
P51689	Arylsulfatase D	ALQWNAGSGGLPENETTFAR	HexNAc(2)Hex(9)	100	1.00.E-17
P51805	Plexin-A3	SLNR	HexNAc(5)Hex(3)Fuc(1)	100	1.00.E-17
P51805	Plexin-A3	GIETTNTCQVINDTAMLCK	HexNAc(2)Hex(5)	4.838	3.68.E-02
P52803	Ephrin-A5	YAVYWNSSNPR	HexNAc(2)Hex(8)	6.698	1.27.E-02
P52803	Ephrin-A5	YAVYWNSSNPR	HexNAc(2)Hex(7)	4.744	1.82.E-03
P53634	Dipeptidyl peptidase 1	DVNCSVMGPQEK	HexNAc(3)Hex(4)Fuc(1)NeuAc(1)	100	1.00.E-17
P53634	Dipeptidyl peptidase 1	VTTYCNETMTGWVHDVLGR	HexNAc(2)Hex(5)	6.575	4.99.E-03
P54709	Sodium/potassium-transporting ATPase subunit beta-3	NLTVCPDGALFEQK	HexNAc(3)Hex(6)Fuc(1)NeuAc(1)	100	1.00.E-17
P54709	Sodium/potassium-transporting ATPase subunit beta-3	NLTVCPDGALFEQK	HexNAc(5)Hex(4)Fuc(2)	11.734	7.20.E-04
P54709	Sodium/potassium-transporting ATPase subunit beta-3	NLTVCPDGALFEQK	HexNAc(5)Hex(5)Fuc(3)	6.324	5.18.E-03

P54709	Sodium/potassium-transporting ATPase subunit beta-3	NLTVCPDGALFEQK	HexNAc(4)Hex(5)Fuc(2)NeuAc(1)	2.821	9.58.E-03
P54753	Ephrin type-B receptor 3	YAAVNITTNQAAPSEVPTLR	HexNAc(2)Hex(5)	4.917	9.10.E-03
P55268	Laminin subunit beta-2	VNLTR	HexNAc(2)Hex(5)	4.016	3.56.E-02
P56706	Protein Wnt-7b	WNCSALGEK	HexNAc(2)Hex(9)	100	1.00.E-17
P57087	Isoform 3 of Junctional adhesion molecule B	LGSQSTNSSYTMNTK	HexNAc(4)Hex(5)Fuc(2)NeuAc(1)	100	1.00.E-17
P61812	Isoform B of Transforming growth factor beta-2	NASNLVK	HexNAc(2)Hex(6)	100	1.00.E-17
P67809	Nuclease-sensitive element-binding protein 1	NDTK	HexNAc(6)Hex(3)Fuc(3)	100	1.00.E-17
P78310	Coxsackievirus and adenovirus receptor	NASSEYSGTYSCTVR	HexNAc(4)Hex(5)Fuc(1)	100	1.00.E-17
P78310	Coxsackievirus and adenovirus receptor	NASSEYSGTYSCTVR	HexNAc(3)Hex(6)Fuc(1)	100	1.00.E-17
P78310	Coxsackievirus and adenovirus receptor	SGDASINVTNLQLSDIGTYQCK	HexNAc(5)Hex(6)Fuc(1)	13.263	1.65.E-04
P78310	Coxsackievirus and adenovirus receptor	NASSEYSGTYSCTVR	HexNAc(3)Hex(6)Fuc(1)NeuAc(1)	6.37	5.54.E-03
P78310	Coxsackievirus and adenovirus receptor	SGDASINVTNLQLSDIGTYQCK	HexNAc(5)Hex(5)Fuc(2)	5.447	4.01.E-05
P78310	Coxsackievirus and adenovirus receptor	NASSEYSGTYSCTVR	HexNAc(2)Hex(5)	4.387	2.47.E-02
P78310	Coxsackievirus and adenovirus receptor	SGDASINVTNLQLSDIGTYQCK	HexNAc(5)Hex(5)NeuAc(1)	3.131	1.32.E-02
P78310	Coxsackievirus and adenovirus receptor	SGDASINVTNLQLSDIGTYQCK	HexNAc(5)Hex(3)	100	1.00.E-17
P78310	Coxsackievirus and adenovirus receptor	NASSEYSGTYSCTVR	HexNAc(4)Hex(6)Fuc(2)	100	1.00.E-17
P78324	Isoform 2 of Tyrosine-protein phosphatase non-receptor type substrate 1	AENQVNVTCQVR	HexNAc(2)Hex(7)	4.236	2.08.E-02
P78324	Isoform 2 of Tyrosine-protein phosphatase non-receptor type substrate 1	AENQVNVTCQVR	HexNAc(2)Hex(6)	6.03	2.56.E-02
P78344	Eukaryotic translation initiation factor 4 gamma 2	DNISPK	HexNAc(7)Hex(3)	100	1.00.E-17
P78504	Protein jagged-1	DDFFGHYACDQNGNK	HexNAc(5)Hex(5)Fuc(3)	100	1.00.E-17
P98164	Low-density lipoprotein receptor-related protein 2	SFLDCTNR	HexNAc(5)Hex(5)Fuc(3)	100	1.00.E-17
P98164	Low-density lipoprotein receptor-related protein 2	INTTYR	HexNAc(2)Hex(8)	9.137	1.11.E-03
P98164	Low-density lipoprotein receptor-related protein 2	VGMDGTNK	HexNAc(2)Hex(8)	6.072	1.74.E-02
P98164	Low-density lipoprotein receptor-related protein 2	VGMDGTNK	HexNAc(2)Hex(7)	5.463	2.92.E-02
P98164	Low-density lipoprotein receptor-related protein 2	MFLNK	HexNAc(2)Hex(7)	3.588	1.20.E-02
P98172	Ephrin-B1	HHDYYITSTSNGSLEGLENR	HexNAc(5)Hex(5)Fuc(3)	100	1.00.E-17
Q01638	Interleukin-1 receptor-like 1	SPTFNR	HexNAc(4)Hex(5)Fuc(3)	5.015	3.46.E-02
Q01973	Inactive tyrosine-protein kinase transmembrane receptor ROR1	FIGNR	HexNAc(3)Hex(6)Fuc(1)	100	1.00.E-17
Q02246	Contactin-2	MNGTEMK	HexNAc(2)Hex(7)	100	1.00.E-17
Q02246	Contactin-2	GTEILVNSSR	HexNAc(2)Hex(6)	100	1.00.E-17
Q02246	Contactin-2	MNGTEMK	HexNAc(2)Hex(5)	100	1.00.E-17
Q02246	Contactin-2	GTEILVNSSR	HexNAc(2)Hex(5)	100	1.00.E-17
Q02246	Contactin-2	GTEILVNSSR	HexNAc(2)Hex(7)	6.92	9.09.E-03
Q02246	Contactin-2	WDPVVPFRNESAVTGYK	HexNAc(2)Hex(5)	6.003	1.76.E-02
Q02246	Contactin-2	MNGTEMK	HexNAc(2)Hex(6)	100	1.00.E-17
Q02246	Contactin-2	ANSTGILSVR	HexNAc(2)Hex(5)	100	1.00.E-17
Q02487	Desmocollin-2	ANYTILK	HexNAc(5)Hex(5)Fuc(3)	9.164	4.16.E-03
Q02809	Isoform 2 of Procollagen-lysine,2-oxoglutarate 5-dioxygenase 1	EQINITLDHR	HexNAc(2)Hex(7)	100	1.00.E-17
Q02880	Isoform Beta-1 of DNA topoisomerase 2-beta	ITIENR	HexNAc(4)Hex(5)Fuc(1)	100	1.00.E-17
Q03701	CCAAT/enhancer-binding protein zeta	DNASLKQLR	HexNAc(5)Hex(4)	6.777	6.97.E-04
Q05586	Isoform 5 of Glutamate receptor ionotropic, NMDA 1	FANYSIMNLQNR	HexNAc(2)Hex(8)	100	1.00.E-17
Q07954	Prolow-density lipoprotein receptor-related protein 1	CNASSQFLCSSGR	HexNAc(5)Hex(5)Fuc(3)	100	1.00.E-17
Q07954	Prolow-density lipoprotein receptor-related protein 1	IETILLNGTDRK	HexNAc(5)Hex(5)Fuc(3)	100	1.00.E-17
Q07954	Prolow-density lipoprotein receptor-related protein 1	WTGHNVTVVQR	HexNAc(5)Hex(4)Fuc(2)	100	1.00.E-17
Q07954	Prolow-density lipoprotein receptor-related protein 1	DNATDSVPLR	HexNAc(5)Hex(4)Fuc(2)	100	1.00.E-17
Q07954	Prolow-density lipoprotein receptor-related protein 1	CNASSQFLCSSGR	HexNAc(5)Hex(4)Fuc(2)	100	1.00.E-17
Q07954	Prolow-density lipoprotein receptor-related protein 1	IETILLNGTDRK	HexNAc(5)Hex(4)Fuc(2)	100	1.00.E-17
Q07954	Prolow-density lipoprotein receptor-related protein 1	DNATDSVPLR	HexNAc(5)Hex(3)Fuc(1)	100	1.00.E-17
Q07954	Prolow-density lipoprotein receptor-related protein 1	AVNSSCR	HexNAc(5)Hex(3)Fuc(1)	100	1.00.E-17
Q07954	Prolow-density lipoprotein receptor-related protein 1	TCVSNCTASQFVCK	HexNAc(3)Hex(6)NeuAc(1)	100	1.00.E-17
Q07954	Prolow-density lipoprotein receptor-related protein 1	WTGHNVTVVQR	HexNAc(3)Hex(6)Fuc(1)NeuAc(1)	100	1.00.E-17
Q07954	Prolow-density lipoprotein receptor-related protein 1	WTGHNVTVVQR	HexNAc(3)Hex(6)Fuc(1)	100	1.00.E-17
Q07954	Prolow-density lipoprotein receptor-related protein 1	WTGHNVTVVQR	HexNAc(2)Hex(6)	100	1.00.E-17
Q07954	Prolow-density lipoprotein receptor-related protein 1	INNGGCQDLCLLTHQGHVNCSCR	HexNAc(2)Hex(5)	100	1.00.E-17
Q07954	Prolow-density lipoprotein receptor-related protein 1	LTSCATNASICGDEAR	HexNAc(5)Hex(5)Fuc(3)	16.403	2.28.E-04
Q07954	Prolow-density lipoprotein receptor-related protein 1	FNSTEYQVVTR	HexNAc(2)Hex(5)	10.322	6.50.E-04
Q07954	Prolow-density lipoprotein receptor-related protein 1	WTGHNVTVVQR	HexNAc(2)Hex(5)	9.53	1.78.E-03
Q07954	Prolow-density lipoprotein receptor-related protein 1	LNGTDPIVAADSK	HexNAc(2)Hex(7)	7.17	7.26.E-03
Q07954	Prolow-density lipoprotein receptor-related protein 1	FGTCSQLCNNTK	HexNAc(2)Hex(7)	6.524	6.02.E-03
Q07954	Prolow-density lipoprotein receptor-related protein 1	DNTTCYEFK	HexNAc(2)Hex(7)	6.372	5.42.E-03
Q07954	Prolow-density lipoprotein receptor-related protein 1	DNATDSVPLR	HexNAc(5)Hex(5)Fuc(3)	6.228	1.87.E-02
Q07954	Prolow-density lipoprotein receptor-related protein 1	ELQGNCSR	HexNAc(5)Hex(4)Fuc(2)	5.267	2.80.E-02
Q07954	Prolow-density lipoprotein receptor-related protein 1	GVTHLNISGLK	HexNAc(5)Hex(5)Fuc(3)	4.411	2.37.E-02
Q07954	Prolow-density lipoprotein receptor-related protein 1	GVTHLNISGLK	HexNAc(5)Hex(4)Fuc(2)	3.857	4.45.E-02
Q07954	Prolow-density lipoprotein receptor-related protein 1	DNTTCYEFKK	HexNAc(2)Hex(7)	3.762	3.14.E-02
Q07954	Prolow-density lipoprotein receptor-related protein 1	FGTCSQLCNNTK	HexNAc(2)Hex(6)	3.492	3.50.E-02
Q07954	Prolow-density lipoprotein receptor-related protein 1	FGTCSQLCNNTK	HexNAc(2)Hex(5)	3.223	1.67.E-02
Q07954	Prolow-density lipoprotein receptor-related protein 1	TCVSNCTASQFVCK	HexNAc(2)Hex(5)	2.966	2.60.E-02
Q07954	Prolow-density lipoprotein receptor-related protein 1	QPMAPNPCEANGGQGPCSHLCLINYNR	HexNAc(2)Hex(5)	2.963	2.87.E-02
Q07954	Prolow-density lipoprotein receptor-related protein 1	MHLNGSNVQVLHR	HexNAc(5)Hex(4)Fuc(2)NeuAc(1)	100	1.00.E-17
Q07954	Prolow-density lipoprotein receptor-related protein 1	FNSTEYQVVTR	HexNAc(2)Hex(4)	100	1.00.E-17
Q07954	Prolow-density lipoprotein receptor-related protein 1	WTGHNVTVVQR	HexNAc(3)Hex(4)Fuc(2)	10.489	5.03.E-03
Q07954	Prolow-density lipoprotein receptor-related protein 1	AVNSSCR	HexNAc(5)Hex(4)Fuc(2)	8.465	2.24.E-03
Q07954	Prolow-density lipoprotein receptor-related protein 1	THANGSIK	HexNAc(5)Hex(4)Fuc(2)	7.534	8.29.E-03
Q07954	Prolow-density lipoprotein receptor-related protein 1	WTGHNVTVVQR	HexNAc(3)Hex(5)Fuc(1)	4.254	3.36.E-02
Q07954	Prolow-density lipoprotein receptor-related protein 1	CNASSQFLCSSGR	HexNAc(5)Hex(5)Fuc(2)	100	1.00.E-17
Q07954	Prolow-density lipoprotein receptor-related protein 1	DNATDSVPLR	HexNAc(5)Hex(4)Fuc(1)	100	1.00.E-17
Q07954	Prolow-density lipoprotein receptor-related protein 1	TCPLDEFQCNNTLCK	HexNAc(4)Hex(5)Fuc(1)	100	1.00.E-17
Q07954	Prolow-density lipoprotein receptor-related protein 1	GVTHLNISGLK	HexNAc(4)Hex(5)	100	1.00.E-17
Q07954	Prolow-density lipoprotein receptor-related protein 1	DNATDSVPLR	HexNAc(4)Hex(4)Fuc(1)	100	1.00.E-17
Q07954	Prolow-density lipoprotein receptor-related protein 1	TCVSNCTASQFVCK	HexNAc(3)Hex(6)Fuc(1)	100	1.00.E-17
Q07954	Prolow-density lipoprotein receptor-related protein 1	WTGHNVTVVQR	HexNAc(3)Hex(4)Fuc(1)	100	1.00.E-17
Q07954	Prolow-density lipoprotein receptor-related protein 1	FNSTEYQVVTR	HexNAc(2)Hex(6)	100	1.00.E-17
Q07954	Prolow-density lipoprotein receptor-related protein 1	VNRFNSTEYQVVTR	HexNAc(2)Hex(5)	100	1.00.E-17
Q07954	Prolow-density lipoprotein receptor-related protein 1	LTSCATNASICGDEAR	HexNAc(2)Hex(5)	100	1.00.E-17
Q07954	Prolow-density lipoprotein receptor-related protein 1	FGTCSQLCNNTK	HexNAc(3)Hex(6)Fuc(1)	6.811	1.07.E-02
Q08722	Leukocyte surface antigen CD47	SDAVSHTGNYTCEVTELTR	HexNAc(4)Hex(5)Fuc(1)	19.094	4.14.E-05
Q08722	Leukocyte surface antigen CD47	DIYTFDGALNK	HexNAc(3)Hex(6)Fuc(1)	9.291	1.42.E-03
Q08722	Leukocyte surface antigen CD47	DIYTFDGALNK	HexNAc(2)Hex(5)	3.685	3.65.E-02
Q08722	Leukocyte surface antigen CD47	GRDIYTFDGALNK	HexNAc(2)Hex(5)	3.205	3.78.E-02
Q08722	Leukocyte surface antigen CD47	DIYTFDGALNK	HexNAc(2)Hex(7)	2.157	2.41.E-02
Q08722	Leukocyte surface antigen CD47	SDAVSHTGNYTCEVTELTR	HexNAc(4)Hex(5)Fuc(2)	100	1.00.E-17
Q08722	Leukocyte surface antigen CD47	SDAVSHTGNYTCEVTELTR	HexNAc(3)Hex(6)Fuc(1)	5.58	2.38.E-02
Q08722	Leukocyte surface antigen CD47	SDAVSHTGNYTCEVTELTR	HexNAc(4)Hex(6)NeuAc(1)	100	1.00.E-17
Q08722	Leukocyte surface antigen CD47	GRDIYTFDGALNK	HexNAc(3)Hex(5)	100	1.00.E-17
Q08AD1	Calmodulin-regulated spectrin-associated protein 2	LNHTDGK	HexNAc(3)Hex(3)	100	1.00.E-17
Q0D2J5	Zinc finger protein 763	NLTSIGK	HexNAc(5)Hex(5)Fuc(2)	7.07	7.27.E-03
Q0D2J5	Zinc finger protein 763	NLTSIGK	HexNAc(5)Hex(4)Fuc(1)NeuAc(1)	100	1.00.E-17
Q12797	Isoform 7 of Aspartyl/asparaginyl beta-hydroxylase	YNLSEVLQGK	HexNAc(2)Hex(6)	4.131	4.72.E-02
Q12860	Contactin-1	GNYSCFVSSPSITK	HexNAc(5)Hex(3)	100	1.00.E-17

Q12913	Receptor-type tyrosine-protein phosphatase eta	YEIDVGNESTTLGYYNGK	HexNAc(2)Hex(7)	100	1.00.E-17
Q13201	Multimerin-1	LNQSNFQK	HexNAc(2)Hex(9)	100	1.00.E-17
Q13201	Multimerin-1	LNDSIQTLVNDNQR	HexNAc(2)Hex(9)	100	1.00.E-17
Q13201	Multimerin-1	IDNISLTVNDVR	HexNAc(2)Hex(9)	100	1.00.E-17
Q13201	Multimerin-1	HPFTGDNCTIK	HexNAc(2)Hex(9)	100	1.00.E-17
Q13201	Multimerin-1	YQQNMSHLEEK	HexNAc(2)Hex(8)	100	1.00.E-17
Q13201	Multimerin-1	HPFTGDNCTIK	HexNAc(2)Hex(8)	4.968	3.93.E-02
Q13201	Multimerin-1	MYQMFNETTSQVR	HexNAc(2)Hex(9)	100	1.00.E-17
Q13201	Multimerin-1	YQQNMSHLEEK	HexNAc(2)Hex(9)	5.734	1.70.E-02
Q13308	Isoform 6 of Inactive tyrosine-protein kinase 7	DGTPLSDGQSNHTVSSK	HexNAc(5)Hex(5)Fuc(3)	100	1.00.E-17
Q13308	Isoform 6 of Inactive tyrosine-protein kinase 7	NLTLR	HexNAc(3)Hex(6)Fuc(1)	100	1.00.E-17
Q13308	Isoform 6 of Inactive tyrosine-protein kinase 7	SANASFNIK	HexNAc(5)Hex(5)Fuc(3)	6.451	2.81.E-03
Q13308	Isoform 6 of Inactive tyrosine-protein kinase 7	SANASFNIK	HexNAc(5)Hex(6)Fuc(3)	3.165	4.09.E-02
Q13308	Isoform 6 of Inactive tyrosine-protein kinase 7	SANASFNIK	HexNAc(5)Hex(4)Fuc(2)	100	1.00.E-17
Q13308	Isoform 6 of Inactive tyrosine-protein kinase 7	DGTPLSDGQSNHTVSSK	HexNAc(4)Hex(5)Fuc(3)NeuAc(1)	100	1.00.E-17
Q13308	Isoform 6 of Inactive tyrosine-protein kinase 7	DGTPLSDGQSNHTVSSK	HexNAc(4)Hex(5)Fuc(1)NeuAc(2)	100	1.00.E-17
Q13308	Isoform 6 of Inactive tyrosine-protein kinase 7	NLTLR	HexNAc(3)Hex(5)NeuAc(1)	100	1.00.E-17
Q13349	Integrin alpha-D	VNNLSQR	HexNAc(2)Hex(8)	4.991	4.02.E-02
Q13433	Zinc transporter ZIP6	YGENNSLSVEGFR	HexNAc(5)Hex(5)Fuc(3)	100	1.00.E-17
Q13433	Zinc transporter ZIP6	NTNENPQECFNASK	HexNAc(5)Hex(4)Fuc(2)	100	1.00.E-17
Q13433	Zinc transporter ZIP6	YGENNSLSVEGFRK	HexNAc(5)Hex(5)Fuc(3)	62.578	3.96.E-08
Q13433	Zinc transporter ZIP6	NTNENPQECFNASK	HexNAc(5)Hex(5)Fuc(2)	100	1.00.E-17
Q13433	Zinc transporter ZIP6	YGENNSLSVEGFRK	HexNAc(2)Hex(8)	100	1.00.E-17
Q13433	Zinc transporter ZIP6	NTNENPQECFNASK	HexNAc(2)Hex(5)	100	1.00.E-17
Q13439	Isoform 5 of Golgin subfamily A member 4	NQSK	HexNAc(4)Hex(5)Fuc(1)NeuAc(2)	10.177	4.19.E-03
Q13586	Stromal interaction molecule 1	LAVTNTTMTGTVLK	HexNAc(2)Hex(8)	3.003	4.49.E-02
Q13641	Trophoblast glycoprotein	RPPLAELAALNLSGSR	HexNAc(2)Hex(8)	4.616	7.30.E-05
Q13641	Trophoblast glycoprotein	VLHNGTLAELQGLPHIR	HexNAc(4)Hex(5)Fuc(2)NeuAc(1)	3.539	3.81.E-02
Q13641	Trophoblast glycoprotein	RPPLAELAALNLSGSR	HexNAc(2)Hex(7)	2.37	2.57.E-02
Q13683	Integrin alpha-7	ANITVK	HexNAc(2)Hex(8)	22.11	2.11.E-05
Q13683	Integrin alpha-7	NITLDCAR	HexNAc(2)Hex(8)	16.411	4.15.E-05
Q13683	Integrin alpha-7	ANITVK	HexNAc(2)Hex(7)	13.215	9.91.E-06
Q13683	Integrin alpha-7	LWNSTFLEEYSAVK	HexNAc(2)Hex(9)	7.371	2.75.E-04
Q13683	Integrin alpha-7	ANITVK	HexNAc(2)Hex(9)	4.005	2.64.E-02
Q13683	Integrin alpha-7	NITLDCAR	HexNAc(2)Hex(7)	3.098	2.21.E-02
Q13683	Integrin alpha-7	ANITVK	HexNAc(2)Hex(4)	100	1.00.E-17
Q13683	Integrin alpha-7	KNITLDCAR	HexNAc(2)Hex(8)	9.639	1.30.E-03
Q13740	CD166 antigen	NATVVWMK	HexNAc(5)Hex(5)Fuc(2)	100	1.00.E-17
Q14108	Lysosome membrane protein 2	CNMINGTDGDSFHPLITK	HexNAc(2)Hex(9)	5.241	1.14.E-04
Q14108	Lysosome membrane protein 2	CNMINGTDGDSFHPLITK	HexNAc(2)Hex(8)	2.231	4.22.E-02
Q14108	Lysosome membrane protein 2	CNMINGTDGDSFHPLITK	HexNAc(2)Hex(8)	2.108	1.54.E-02
Q14108	Lysosome membrane protein 2	CNMINGTDGDSFHPLITK	HexNAc(2)Hex(9)	100	1.00.E-17
Q14126	Desmoglein-2	YVQNGTYTVK	HexNAc(5)Hex(5)Fuc(3)	5.502	1.71.E-02
Q14157	Isoform 5 of Ubiquitin-associated protein 2-like	PQTNK	HexNAc(4)Hex(7)Fuc(1)	100	1.00.E-17
Q14517	Protocadherin Fat 1	TGALTVQNTTQLR	HexNAc(2)Hex(6)	6.054	2.32.E-02
Q14517	Protocadherin Fat 1	QVTQEMLNHTIAIR	HexNAc(2)Hex(9)	100	1.00.E-17
Q14517	Protocadherin Fat 1	TGALTVQNTTQLR	HexNAc(2)Hex(7)	100	1.00.E-17
Q14686	Nuclear receptor coactivator 6	QNNTNANK	HexNAc(6)Hex(5)	16.166	7.17.E-06
Q14832	Metabotropic glutamate receptor 3	INFTAPFNPNK	HexNAc(5)Hex(5)Fuc(3)	100	1.00.E-17
Q14982	Isoform 4 of Opioid-binding protein/cell adhesion molecule	DYGNYTCVATNK	HexNAc(5)Hex(3)Fuc(1)	100	1.00.E-17
Q15149	Plectin	NLVDNITGQR	HexNAc(5)Hex(3)	100	1.00.E-17
Q15165	Serum paraoxonase/arylesterase 2	IQNILCEKPTVTTVYANNGSVLQGSSVASVYDGK	HexNAc(2)Hex(6)	100	1.00.E-17
Q15165	Serum paraoxonase/arylesterase 2	HTNMNLTQLK	HexNAc(2)Hex(6)	100	1.00.E-17
Q15165	Serum paraoxonase/arylesterase 2	HTNMNLTQLK	HexNAc(2)Hex(8)	4.622	2.19.E-03
Q15165	Serum paraoxonase/arylesterase 2	HTNMNLTQLK	HexNAc(2)Hex(9)	3.289	4.13.E-02
Q15165	Serum paraoxonase/arylesterase 2	HTNMNLTQLK	HexNAc(2)Hex(8)	3.191	1.20.E-03
Q15223	Nectin-1	NPNGTVTVISR	HexNAc(4)Hex(5)	100	1.00.E-17
Q15293	Reticulocalbin-1	VVRPDSELGERPPEDNQSFQYDHEAFLGK	HexNAc(2)Hex(5)	2.716	3.48.E-03
Q15293	Reticulocalbin-1	VVRPDSELGERPPEDNQSFQYDHEAFLGK	HexNAc(4)Hex(3)Fuc(1)	2.267	3.25.E-02
Q15293	Reticulocalbin-1	VVRPDSELGERPPEDNQSFQYDHEAFLGK	HexNAc(2)Hex(7)	7.796	1.35.E-04
Q15293	Reticulocalbin-1	VVRPDSELGERPPEDNQSFQYDHEAFLGK	HexNAc(3)Hex(3)Fuc(1)	4.77	5.56.E-05
Q15652	Probable JmjC domain-containing histone demethylation protein 2C	PNNNLSK	HexNAc(5)Hex(5)Fuc(2)	100	1.00.E-17
Q15910	Isoform 2 of Histone-lysine N-methyltransferase EZH2	NVSCK	HexNAc(4)Hex(7)	14.324	1.92.E-04
Q16288	NT-3 growth factor receptor	NPLGTANQTINGHFLK	HexNAc(2)Hex(5)	100	1.00.E-17
Q16527	Cysteine and glycine-rich protein 2	PTTNPNTSKFAQK	HexNAc(2)Hex(4)	100	1.00.E-17
Q16620	Isoform 4 of BDNF/NT-3 growth factors receptor	NSNLQHINFTR	HexNAc(2)Hex(8)	100	1.00.E-17
Q16851	UTP--glucose-1-phosphate uridylyltransferase	NVSLK	HexNAc(5)Hex(5)Fuc(3)	100	1.00.E-17
Q16851	UTP--glucose-1-phosphate uridylyltransferase	NVSLK	HexNAc(5)Hex(4)Fuc(2)	100	1.00.E-17
Q2KHM9	Protein moonraker	NISEQK	HexNAc(6)Hex(3)Fuc(1)NeuAc(1)	100	1.00.E-17
Q2LD37	Uncharacterized protein KIAA1109	GLQTNVSIPK	HexNAc(4)Hex(5)	100	1.00.E-17
Q32P28	Prolyl 3-hydroxylase 1	LLNGSQR	HexNAc(2)Hex(9)	2.651	3.18.E-02
Q32P28	Prolyl 3-hydroxylase 1	VPLQSAHLYYNVTEK	HexNAc(2)Hex(9)	2.641	4.14.E-03
Q32P28	Prolyl 3-hydroxylase 1	LLNGSQR	HexNAc(2)Hex(5)	100	1.00.E-17
Q49AM3	Tetratricopeptide repeat protein 31	LFGNR	HexNAc(5)Hex(3)Fuc(1)	19.458	2.25.E-04
Q4KMQ2	Isoform 2 of Anoctamin-6	LNITCESSK	HexNAc(6)Hex(4)	100	1.00.E-17
Q58EX2	Isoform 4 of Protein sidekick-2	FWLVEGNSSR	HexNAc(2)Hex(6)	100	1.00.E-17
Q58EX2	Isoform 4 of Protein sidekick-2	VISAGGNDSR	HexNAc(2)Hex(5)	100	1.00.E-17
Q58EX2	Isoform 4 of Protein sidekick-2	TNQSIMIQWQPPPESHQNGILK	HexNAc(2)Hex(5)	8.231	8.35.E-03
Q58EX2	Isoform 4 of Protein sidekick-2	AINLTWTKPFDGNSPLIR	HexNAc(2)Hex(5)	5.629	1.83.E-02
Q5IJ48	Protein crumbs homolog 2	EGPPAAFSGHNASSGR	HexNAc(5)Hex(5)Fuc(3)	100	1.00.E-17
Q5IJ48	Protein crumbs homolog 2	ILLAENFTGCLGR	HexNAc(5)Hex(4)Fuc(2)	7.66	6.99.E-03
Q5IJ48	Protein crumbs homolog 2	LDGCHLPFFPLPLDNSSQPSELGGR	HexNAc(2)Hex(8)	7.094	3.79.E-03
Q5IJ48	Protein crumbs homolog 2	NGSLAGGVR	HexNAc(2)Hex(9)	6.697	3.82.E-03
Q5IJ48	Protein crumbs homolog 2	ILLAENFTGCLGR	HexNAc(2)Hex(9)	5.623	2.01.E-02
Q5IJ48	Protein crumbs homolog 2	NGSLAGGVR	HexNAc(2)Hex(8)	4.95	2.51.E-02
Q5IJ48	Protein crumbs homolog 2	EGPPAAFSGHNASSGR	HexNAc(2)Hex(9)	4.611	1.51.E-02
Q5IJ48	Protein crumbs homolog 2	EGPPAAFSGHNASSGR	HexNAc(2)Hex(6)	4.522	3.80.E-02
Q5IJ48	Protein crumbs homolog 2	EGPPAAFSGHNASSGR	HexNAc(2)Hex(8)	4.465	1.29.E-02
Q5IJ48	Protein crumbs homolog 2	EGPPAAFSGHNASSGR	HexNAc(2)Hex(7)	4.407	4.24.E-02
Q5IJ48	Protein crumbs homolog 2	EVLECASAPCEHNASCLEGLGSFR	HexNAc(4)Hex(4)Fuc(2)NeuAc(1)	100	1.00.E-17
Q5IJ48	Protein crumbs homolog 2	NGSLAGGVR	HexNAc(2)Hex(6)	100	1.00.E-17
Q5JU69	Torsin-2A	SWVQGNLTACGR	HexNAc(2)Hex(9)	4.447	1.96.E-02
Q5T1H1	Protein eyes shut homolog	LNVTVK	HexNAc(2)Hex(9)	100	1.00.E-17
Q5T4D3	Isoform 3 of Transmembrane and TPR repeat-containing protein 4	GNQTAAIR	HexNAc(2)Hex(8)	100	1.00.E-17

Q5T4D3	Isoform 3 of Transmembrane and TPR repeat-containing protein 4	NLADKGNQTAAIR	HexNAc(2)Hex(6)	100	1.00.E-17
Q5T4D3	Isoform 3 of Transmembrane and TPR repeat-containing protein 4	GNQTAAIR	HexNAc(2)Hex(6)	7.668	1.65.E-02
Q5T4D3	Isoform 3 of Transmembrane and TPR repeat-containing protein 4	GNQTAAIR	HexNAc(2)Hex(5)	100	1.00.E-17
Q5T5Y3	Isoform 3 of Calmodulin-regulated spectrin-associated protein 1	LTGTGPKNITK	HexNAc(3)Hex(3)Fuc(2)	100	1.00.E-17
Q5TCQ9	Membrane-associated guanylate kinase, WW and PDZ domain-containing protein 3	VVSNK	HexNAc(4)Hex(7)Fuc(1)	100	1.00.E-17
Q5UIP0	Telomere-associated protein RIF1	TVNGIENK	HexNAc(3)Hex(5)NeuAc(1)	100	1.00.E-17
Q5VU97	VWFA and cache domain-containing protein 1	FNTSLAGDLTNLVHGSHCSK	HexNAc(2)Hex(7)	7.438	8.17.E-03
Q5VU97	VWFA and cache domain-containing protein 1	EAYNVSYAWK	HexNAc(2)Hex(5)	100	1.00.E-17
Q5VYK3	Proteasome-associated protein ECM29 homolog	SLMNNSK	HexNAc(5)Hex(5)NeuAc(1)	17.923	2.49.E-06
Q5ZPR3	CD276 antigen	VVLGANGTYSCLVR	HexNAc(5)Hex(6)NeuAc(3)	100	1.00.E-17
Q5ZPR3	CD276 antigen	QLVHSFAEGQDQGSAYANR	HexNAc(5)Hex(6)Fuc(3)	100	1.00.E-17
Q5ZPR3	CD276 antigen	QLVHSFAEGQDQGSAYANR	HexNAc(5)Hex(5)Fuc(3)	100	1.00.E-17
Q5ZPR3	CD276 antigen	VVLGANGTYSCLVR	HexNAc(5)Hex(4)Fuc(2)	100	1.00.E-17
Q5ZPR3	CD276 antigen	VVLGANGTYSCLVR	HexNAc(5)Hex(5)Fuc(3)	67.349	1.82.E-12
Q5ZPR3	CD276 antigen	TALFPDLLAQGNASLR	HexNAc(3)Hex(6)Fuc(1)	13.452	1.24.E-05
Q5ZPR3	CD276 antigen	TALFPDLLAQGNASLR	HexNAc(2)Hex(6)	10.424	6.07.E-05
Q5ZPR3	CD276 antigen	QLVHSFAEGQDQGSAYANR	HexNAc(5)Hex(5)Fuc(1)NeuAc(1)	10.192	6.03.E-04
Q5ZPR3	CD276 antigen	TALFPDLLAQGNASLR	HexNAc(2)Hex(7)	7.485	6.35.E-03
Q5ZPR3	CD276 antigen	TALFPDLLAQGNASLR	HexNAc(3)Hex(6)NeuAc(1)	4.009	2.59.E-02
Q5ZPR3	CD276 antigen	TALFPDLLAQGNASLR	HexNAc(3)Hex(5)Fuc(1)	3.794	7.75.E-03
Q5ZPR3	CD276 antigen	VVLGANGTYSCLVR	HexNAc(4)Hex(5)Fuc(2)NeuAc(1)	2.484	4.12.E-02
Q5ZPR3	CD276 antigen	TALFPDLLAQGNASLR	HexNAc(3)Hex(6)Fuc(1)NeuAc(1)	100	1.00.E-17
Q5ZPR3	CD276 antigen	QLVHSFAEGQDQGSAYANR	HexNAc(5)Hex(5)Fuc(1)NeuAc(2)	10.816	9.82.E-04
Q5ZPR3	CD276 antigen	TALFPDLLAQGNASLR	HexNAc(3)Hex(5)NeuAc(1)	3.133	1.96.E-02
Q5ZPR3	CD276 antigen	VVLGANGTYSCLVR	HexNAc(5)Hex(6)Fuc(1)NeuAc(2)	100	1.00.E-17
Q6AWC8	Putative uncharacterized protein LOC100129027	NTSLSK	HexNAc(5)Hex(5)Fuc(3)	10.606	1.44.E-03
Q6IQ23	Isoform 2 of Pleckstrin homology domain-containing family A member 7	NSSHVDRR	HexNAc(2)Hex(5)	100	1.00.E-17
Q6N022	Teneurin-4	RNVTLPIDNGLNLVEWR	HexNAc(2)Hex(8)	100	1.00.E-17
Q6N022	Teneurin-4	FNVSLGK	HexNAc(2)Hex(8)	100	1.00.E-17
Q6N022	Teneurin-4	FNVSLGK	HexNAc(2)Hex(7)	100	1.00.E-17
Q6N022	Teneurin-4	FNVSLGK	HexNAc(2)Hex(6)	100	1.00.E-17
Q6N022	Teneurin-4	FNVSLGK	HexNAc(2)Hex(5)	100	1.00.E-17
Q6N022	Teneurin-4	LTNVTFPTGQVSSFR	HexNAc(2)Hex(9)	5.289	8.62.E-03
Q6N022	Teneurin-4	NVTLPIDNGLNLVEWR	HexNAc(2)Hex(8)	100	1.00.E-17
Q6PIU2	Isoform 2 of Neutral cholesterol ester hydrolase 1	LNWTSLLPASFTK	HexNAc(2)Hex(5)	100	1.00.E-17
Q6UVY6	DBH-like monooxygenase protein 1	INLTR	HexNAc(3)Hex(5)Fuc(1)	5.319	3.07.E-02
Q6UVY6	DBH-like monooxygenase protein 1	DAQQDYHLEYAMENSTHTIIEFTR	HexNAc(2)Hex(7)	52.512	3.81.E-06
Q6UX52	Uncharacterized protein C17orf99	ANFTLQDR	HexNAc(4)Hex(5)	100	1.00.E-17
Q6UXK2	Immunoglobulin superfamily containing leucine-rich repeat protein 2	NLSALQLLK	HexNAc(4)Hex(3)NeuAc(1)	100	1.00.E-17
Q6UXK2	Immunoglobulin superfamily containing leucine-rich repeat protein 2	AHNELGANSTSIR	HexNAc(2)Hex(9)	100	1.00.E-17
Q6UXK2	Immunoglobulin superfamily containing leucine-rich repeat protein 2	AHNELGANSTSIR	HexNAc(2)Hex(8)	100	1.00.E-17
Q6UXK2	Immunoglobulin superfamily containing leucine-rich repeat protein 2	AHNELGANSTSIR	HexNAc(5)Hex(3)Fuc(1)	100	1.00.E-17
Q6UXK2	Immunoglobulin superfamily containing leucine-rich repeat protein 2	FLALANGSLLVPLLSAK	HexNAc(2)Hex(9)	100	1.00.E-17
Q6UXK2	Immunoglobulin superfamily containing leucine-rich repeat protein 2	AHNELGANSTSIR	HexNAc(2)Hex(5)	100	1.00.E-17
Q6UXK5	Leucine-rich repeat neuronal protein 1	SCVNVTTK	HexNAc(5)Hex(4)Fuc(2)	6.877	1.18.E-02
Q6UXK5	Leucine-rich repeat neuronal protein 1	SCVNVTTK	HexNAc(5)Hex(5)Fuc(3)	5.566	3.66.E-02
Q6V0I7	Protocadherin Fat 4	INITVSDVNDHTPK	HexNAc(2)Hex(7)	100	1.00.E-17
Q6V0I7	Protocadherin Fat 4	LNITAK	HexNAc(2)Hex(7)	6.642	1.94.E-02
Q6V0I7	Protocadherin Fat 4	NGTATVLSVDR	HexNAc(5)Hex(3)Fuc(1)	100	1.00.E-17
Q6V0I7	Protocadherin Fat 4	LNITAK	HexNAc(2)Hex(9)	100	1.00.E-17
Q6V0I7	Protocadherin Fat 4	LNITAK	HexNAc(2)Hex(6)	100	1.00.E-17
Q6ZPD9	Probable C-mannosyltransferase DPY19L3	QMLQAPTLVQGFHGLIYDNK	HexNAc(2)Hex(9)	100	1.00.E-17
Q6ZSG1	E3 ubiquitin-protein ligase RNF165	LGNVTR	HexNAc(2)Hex(6)	100	1.00.E-17
Q70UQ0	Isoform 4 of Inhibitor of nuclear factor kappa-B kinase-interacting protein	FQNITDFWK	HexNAc(2)Hex(6)	100	1.00.E-17
Q70UQ0	Inhibitor of nuclear factor kappa-B kinase-interacting protein	ISNLTIVQAEIK	HexNAc(2)Hex(6)	4.699	1.60.E-02
Q71F56	Mediator of RNA polymerase II transcription subunit 13-like	DEKPVNK	HexNAc(5)Hex(5)Fuc(1)	100	1.00.E-17
Q75T13	GPI inositol-deacylase	HINLTTLSVAGGFR	HexNAc(2)Hex(6)	4.41	3.89.E-02
Q75T13	GPI inositol-deacylase	LHIAQPENNTHVALFK	HexNAc(2)Hex(8)	3.461	4.43.E-02
Q75V66	Anoctamin-5	LNSTCLASK	HexNAc(2)Hex(6)	100	1.00.E-17
Q7LGA3	Heparan sulfate 2-O-sulfotransferase 1	NITSWK	HexNAc(4)Hex(4)NeuAc(1)	100	1.00.E-17
Q7LGA3	Heparan sulfate 2-O-sulfotransferase 1	NITSWK	HexNAc(4)Hex(5)NeuAc(1)	8.916	2.38.E-03
Q7LGA3	Heparan sulfate 2-O-sulfotransferase 1	YHVLHINTTK	HexNAc(4)Hex(7)Fuc(2)	100	1.00.E-17
Q7LGA3	Heparan sulfate 2-O-sulfotransferase 1	NITSWK	HexNAc(4)Hex(5)Fuc(1)	100	1.00.E-17
Q7LGA3	Heparan sulfate 2-O-sulfotransferase 1	NITSWK	HexNAc(2)Hex(6)	100	1.00.E-17
Q7LGA3	Heparan sulfate 2-O-sulfotransferase 1	YHVLHINTTK	HexNAc(4)Hex(5)NeuAc(1)	100	1.00.E-17
Q7Z388	Probable C-mannosyltransferase DPY19L4	FGLNMTK	HexNAc(2)Hex(6)	100	1.00.E-17
Q7Z3B1	Neuronal growth regulator 1	GAWLNR	HexNAc(2)Hex(7)	5.868	2.18.E-02
Q7Z3B1	Neuronal growth regulator 1	GAWLNR	HexNAc(2)Hex(8)	100	1.00.E-17
Q7Z3B1	Neuronal growth regulator 1	LFNGQQGIIIQNFSTR	HexNAc(2)Hex(5)	100	1.00.E-17
Q7Z4H8	KDEL motif-containing protein 2	VNGTPSPIPIISWCGSLDSR	HexNAc(2)Hex(6)	6.311	1.53.E-02
Q7Z4H8	KDEL motif-containing protein 2	YFYLQAVNSEGQNLTR	HexNAc(2)Hex(9)	2.394	1.00.E-02
Q7Z4K8	Tripartite motif-containing protein 46	NLTLER	HexNAc(4)Hex(5)Fuc(3)	5.828	1.05.E-02
Q7Z4K8	Tripartite motif-containing protein 46	NLTLER	HexNAc(2)Hex(6)	100	1.00.E-17
Q7Z5N4	Protein sidekick-1	TVNSSSTSTMCELTHLK	HexNAc(5)Hex(5)Fuc(3)	100	1.00.E-17
Q7Z5N4	Protein sidekick-1	NLTSHTK	HexNAc(5)Hex(4)Fuc(2)	100	1.00.E-17
Q7Z5N4	Protein sidekick-1	NLTSHTK	HexNAc(6)Hex(6)Fuc(3)	100	1.00.E-17
Q8IV08	Phospholipase D3	DNHTHSDIQVK	HexNAc(5)Hex(5)Fuc(1)NeuAc(2)	100	1.00.E-17
Q8IV08	Phospholipase D3	DNHTHSDIQVK	HexNAc(3)Hex(4)Fuc(1)NeuAc(1)	19.86	8.90.E-06
Q8IV08	Phospholipase D3	DNHTHSDIQVK	HexNAc(5)Hex(4)Fuc(1)NeuAc(1)	17.822	3.88.E-04
Q8IV08	Phospholipase D3	DNHTHSDIQVK	HexNAc(4)Hex(3)Fuc(1)	6.159	2.78.E-02
Q8IV08	Phospholipase D3	ELGVVMYNCSCLAR	HexNAc(2)Hex(5)	5.972	1.43.E-03
Q8IV08	Phospholipase D3	ELGVVMYNCSCLAR	HexNAc(2)Hex(4)	3.98	2.54.E-02
Q8IV08	Phospholipase D3	ELGVVMYNCSCLAR	HexNAc(2)Hex(9)	100	1.00.E-17
Q8IV08	Phospholipase D3	ELGVVMYNCSCLAR	HexNAc(2)Hex(5)	100	1.00.E-17
Q8IV08	Phospholipase D3	DNHTHSDIQVK	HexNAc(6)Hex(5)Fuc(1)NeuAc(1)	100	1.00.E-17
Q8IV08	Phospholipase D3	ELGVVMYNCSCLAR	HexNAc(2)Hex(8)	100	1.00.E-17
Q8IWA5	Choline transporter-like protein 2	NITDLVEGAK	HexNAc(4)Hex(5)Fuc(3)	100	1.00.E-17
Q8IWU5	Extracellular sulfatase Sulf-2	YLNEYNGSYVPPGWK	HexNAc(2)Hex(9)	100	1.00.E-17
Q8IWU6	Extracellular sulfatase Sulf-1	CFILPNDSIHCER	HexNAc(2)Hex(8)	100	1.00.E-17

Q8IWY4	Signal peptide, CUB and EGF-like domain-containing protein 1	RNGTSSGLGPSCSDAPTTPIK	HexNAc(5)Hex(4)Fuc(2)NeuAc(1)	100	1.00.E-17
Q8IYK4	Procollagen galactosyltransferase 2	MAIWAATDHNVDNTTEIFR	HexNAc(2)Hex(9)	22.727	1.96.E-07
Q8IYK4	Procollagen galactosyltransferase 2	ALNTSQLK	HexNAc(2)Hex(9)	17.924	2.65.E-04
Q8IYK4	Procollagen galactosyltransferase 2	ALNTSQLK	HexNAc(2)Hex(8)	10.653	1.64.E-03
Q8IYK4	Procollagen galactosyltransferase 2	ALNTSQLK	HexNAc(2)Hex(7)	6.651	8.06.E-03
Q8IYK4	Procollagen galactosyltransferase 2	ALNTSQLK	HexNAc(2)Hex(6)	4.707	3.56.E-02
Q8IYK4	Procollagen galactosyltransferase 2	ALNTSQLK	HexNAc(2)Hex(4)	100	1.00.E-17
Q8IYR6	Tomoregulin-1	SINCSELNVR	HexNAc(5)Hex(5)Fuc(3)	6.098	1.45.E-02
Q8N0W4	Isoform 2 of Neuroligin-4, X-linked	NTTQFAAVCPQHLDER	HexNAc(5)Hex(5)Fuc(3)	10.108	1.14.E-04
Q8N0W4	Isoform 2 of Neuroligin-4, X-linked	NTTQFAAVCPQHLDER	HexNAc(5)Hex(4)Fuc(1)	100	1.00.E-17
Q8N6C5	Isoform 4 of Immunoglobulin superfamily member 1	NLTLWCR	HexNAc(2)Hex(7)	100	1.00.E-17
Q8N6C5	Isoform 4 of Immunoglobulin superfamily member 1	NLTLWCR	HexNAc(2)Hex(6)	5.445	1.90.E-02
Q8N6C5	Isoform 4 of Immunoglobulin superfamily member 1	NLTLWCR	HexNAc(3)Hex(6)Fuc(1)	100	1.00.E-17
Q8N7P1	Inactive phospholipase D5	LFCPKNR	HexNAc(5)Hex(4)Fuc(2)	100	1.00.E-17
Q8N7P1	Inactive phospholipase D5	LFCPKNR	HexNAc(5)Hex(3)Fuc(1)	100	1.00.E-17
Q8N7P1	Inactive phospholipase D5	LFCPKNR	HexNAc(4)Hex(3)Fuc(1)	100	1.00.E-17
Q8N7P1	Inactive phospholipase D5	LFCPKNR	HexNAc(5)Hex(4)Fuc(1)	5.491	2.35.E-02
Q8N7P1	Inactive phospholipase D5	LFCPKNR	HexNAc(5)Hex(5)Fuc(3)	100	1.00.E-17
Q8N7P1	Inactive phospholipase D5	LFCPKNR	HexNAc(4)Hex(4)Fuc(2)	100	1.00.E-17
Q8NBJ4	Golgi membrane protein 1	AVLVNNITTGER	HexNAc(5)Hex(5)Fuc(3)	20.362	2.15.E-05
Q8NBJ5	Procollagen galactosyltransferase 1	TALWVATDHNMDNTSTVLR	HexNAc(2)Hex(8)	100	1.00.E-17
Q8NBJ5	Procollagen galactosyltransferase 1	TALWVATDHNMDNTSTVLR	HexNAc(2)Hex(7)	4.241	1.40.E-02
Q8NBJ5	Procollagen galactosyltransferase 1	TALWVATDHNMDNTSTVLR	HexNAc(2)Hex(8)	2.39	7.57.E-03
Q8NBJ5	Procollagen galactosyltransferase 1	TALWVATDHNMDNTSTVLR	HexNAc(2)Hex(9)	2.085	2.90.E-02
Q8NBN3	Transmembrane protein 87A	NTTIFLK	HexNAc(3)Hex(6)Fuc(1)	8.256	1.72.E-04
Q8NC26	Zinc finger protein 114	NHSK	HexNAc(4)Hex(6)Fuc(2)	100	1.00.E-17
Q8NFC6	Biorientation of chromosomes in cell division protein 1-like 1	NYTK	HexNAc(8)Hex(4)	100	1.00.E-17
Q8NFC6	Biorientation of chromosomes in cell division protein 1-like 1	NKSTDK	HexNAc(4)Hex(4)	100	1.00.E-17
Q8NFM7	Interleukin-17 receptor D	QLNSSFK	HexNAc(2)Hex(9)	100	1.00.E-17
Q8NFM7	Interleukin-17 receptor D	NSGLYNITFK	HexNAc(2)Hex(8)	100	1.00.E-17
Q8NFM7	Interleukin-17 receptor D	NESNYHPFFFR	HexNAc(2)Hex(7)	100	1.00.E-17
Q8NFM7	Interleukin-17 receptor D	YDNCTTYLNPVGK	HexNAc(5)Hex(5)Fuc(3)	25.092	4.35.E-06
Q8NFM7	Interleukin-17 receptor D	QLNSSFK	HexNAc(2)Hex(8)	5.437	1.82.E-02
Q8NFM7	Interleukin-17 receptor D	NESNYHPFFFR	HexNAc(2)Hex(8)	4.491	1.55.E-02
Q8NFM7	Interleukin-17 receptor D	YDNCTTYLNPVGK	HexNAc(2)Hex(8)	3.502	4.45.E-02
Q8NFM7	Interleukin-17 receptor D	NESNYHPFFFR	HexNAc(2)Hex(9)	100	1.00.E-17
Q8NFP4	Isoform 2 of MAM domain-containing glycosylphosphatidylinositol anchor protein 1	NATFQITPDVIK	HexNAc(2)Hex(9)	100	1.00.E-17
Q8NFP4	Isoform 2 of MAM domain-containing glycosylphosphatidylinositol anchor protein 1	DSGYYNCTATNNVGNPAK	HexNAc(2)Hex(8)	7.533	1.08.E-02
Q8NFQ8	Torsin-1A-interacting protein 2	HLNASNPTEPATIIFTAAR	HexNAc(2)Hex(6)	100	1.00.E-17
Q8NFT8	Delta and Notch-like epidermal growth factor-related receptor	KPCQNNASCIDANEK	HexNAc(5)Hex(3)Fuc(1)	100	1.00.E-17
Q8NFZ8	Cell adhesion molecule 4	QTLFFNGTR	HexNAc(4)Hex(6)Fuc(1)	100	1.00.E-17
Q8NHM5	Isoform 3 of Lysine-specific demethylase 2B	GLNGTPR	HexNAc(2)Hex(7)	6.887	2.13.E-02
Q8NI08	Nuclear receptor coactivator 7	FNITPNK	HexNAc(5)Hex(6)Fuc(1)	100	1.00.E-17
Q8NI27	THO complex subunit 2	NETITK	HexNAc(5)Hex(5)Fuc(1)	100	1.00.E-17
Q8TBZ0	Coiled-coil domain-containing protein 110	NITDLK	HexNAc(5)Hex(5)Fuc(1)	100	1.00.E-17
Q8TDW7	Protocadherin Fat 3	ANYSLK	HexNAc(2)Hex(6)	6.103	5.23.E-03
Q8TEU7	Zinc finger protein 638	NISEK	HexNAc(6)Hex(4)NeuAc(1)	9.476	5.76.E-04
Q8WX77	Insulin-like growth factor-binding protein-like 1	SVHNVTGAQVGLSCEVR	HexNAc(2)Hex(5)	100	1.00.E-17
Q8WZ42	Titin	PEVINITR	HexNAc(4)Hex(4)Fuc(2)	4.699	2.87.E-02
Q92542	Nicastrin	IYIPLNK	HexNAc(2)Hex(6)	3.775	3.44.E-02
Q92608	Dedicator of cytokinesis protein 2	TVNR	HexNAc(5)Hex(4)Fuc(2)	100	1.00.E-17
Q92608	Dedicator of cytokinesis protein 2	TVNR	HexNAc(5)Hex(4)Fuc(1)	6.256	2.79.E-02
Q92608	Dedicator of cytokinesis protein 2	TVNR	HexNAc(5)Hex(5)Fuc(3)	100	1.00.E-17
Q92733	Proline-rich protein PRCC	NLTVK	HexNAc(3)Hex(3)Fuc(1)	100	1.00.E-17
Q92791	Endoplasmic reticulum protein SC65	EEAMLYHNQTAELR	HexNAc(2)Hex(6)	7.169	2.89.E-03
Q92791	Endoplasmic reticulum protein SC65	EEAMLYHNQTAELR	HexNAc(2)Hex(7)	3.668	3.24.E-02
Q92791	Endoplasmic reticulum protein SC65	DSEAFCHANCSGPAPAAKPDPDGGR	HexNAc(2)Hex(8)	2.382	3.58.E-02
Q92791	Endoplasmic reticulum protein SC65	DSEAFCHANCSGPAPAAKPDPDGGR	HexNAc(2)Hex(9)	17.466	1.92.E-04
Q92791	Endoplasmic reticulum protein SC65	EEAMLYHNQTAELR	HexNAc(2)Hex(6)	100	1.00.E-17
Q92823	Neuronal cell adhesion molecule	IPANK	HexNAc(5)Hex(5)Fuc(3)	100	1.00.E-17
Q92823	Neuronal cell adhesion molecule	FNHTQTIQQK	HexNAc(2)Hex(5)	9.741	3.91.E-03
Q92823	Neuronal cell adhesion molecule	FNHTQTIQQK	HexNAc(2)Hex(8)	8.417	6.54.E-03
Q92823	Neuronal cell adhesion molecule	IPANK	HexNAc(5)Hex(4)Fuc(2)	8.098	4.61.E-03
Q92823	Neuronal cell adhesion molecule	FNHTQTIQQK	HexNAc(2)Hex(6)	7.668	1.52.E-03
Q92823	Neuronal cell adhesion molecule	FNHTQTIQQK	HexNAc(2)Hex(7)	6.707	5.38.E-03
Q92823	Neuronal cell adhesion molecule	GSALHEDIYVLHENGTLEIPVAQK	HexNAc(2)Hex(5)	100	1.00.E-17
Q92854	Semaphorin-4D	AANYTSSLNLPDK	HexNAc(5)Hex(5)Fuc(3)	100	1.00.E-17
Q92854	Semaphorin-4D	NSSHSPLR	HexNAc(2)Hex(8)	3.801	4.19.E-02
Q92879	Isoform 4 of CUGBP Elav-like family member 1	NDSK	HexNAc(6)Hex(6)NeuAc(1)	100	1.00.E-17
Q92896	Isoform 2 of Golgi apparatus protein 1	LNLTTDPK	HexNAc(5)Hex(5)Fuc(2)	100	1.00.E-17
Q92896	Isoform 2 of Golgi apparatus protein 1	GNITEYQCHQYITK	HexNAc(4)Hex(5)Fuc(1)NeuAc(1)	100	1.00.E-17
Q92896	Isoform 2 of Golgi apparatus protein 1	GNITEYQCHQYITK	HexNAc(4)Hex(4)Fuc(1)NeuAc(1)	40.129	2.91.E-09
Q92896	Isoform 2 of Golgi apparatus protein 1	LNLTTDPK	HexNAc(4)Hex(6)Fuc(2)	8.713	3.07.E-03
Q92896	Isoform 2 of Golgi apparatus protein 1	LNLTTDPK	HexNAc(4)Hex(7)Fuc(2)	5.971	1.28.E-02
Q92896	Isoform 2 of Golgi apparatus protein 1	LNLTTDPK	HexNAc(5)Hex(6)Fuc(3)	5.044	2.60.E-02
Q92896	Isoform 2 of Golgi apparatus protein 1	LNLTTDPK	HexNAc(4)Hex(6)Fuc(1)	100	1.00.E-17
Q92896	Isoform 2 of Golgi apparatus protein 1	LNLTTDPK	HexNAc(2)Hex(5)	100	1.00.E-17
Q92896	Isoform 2 of Golgi apparatus protein 1	LNLTTDPK	HexNAc(3)Hex(6)Fuc(1)	100	1.00.E-17
Q96AY3	Peptidyl-prolyl cis-trans isomerase FKBP10	TLSRPSETCNETTK	HexNAc(2)Hex(6)	8.655	1.27.E-03
Q96AY3	Peptidyl-prolyl cis-trans isomerase FKBP10	YHYNGSLMDGTLFDSSYSR	HexNAc(2)Hex(9)	3.458	3.90.E-02
Q96AY3	Peptidyl-prolyl cis-trans isomerase FKBP10	YHYNGSLMDGTLFDSSYSR	HexNAc(2)Hex(9)	2.849	2.56.E-03
Q96AY4	Tetratricopeptide repeat protein 28	NNLFNR	HexNAc(2)Hex(6)	100	1.00.E-17
Q96GX1	Tectonic-2	FITNTETPLNNGSTPR	HexNAc(2)Hex(8)	100	1.00.E-17
Q96GX1	Tectonic-2	WNNNTISEINVK	HexNAc(2)Hex(9)	6.582	4.56.E-03
Q96GX1	Tectonic-2	MNNVTTLHLWQSAGR	HexNAc(2)Hex(9)	100	1.00.E-17
Q96HD1	Isoform 2 of Cysteine-rich with EGF-like domain protein 1	NASHLVCSACFGPCAR	HexNAc(2)Hex(5)	3.331	4.26.E-02
Q96HE7	ERO1-like protein alpha	WGHNITEFQQR	HexNAc(2)Hex(9)	2.067	2.13.E-02
Q96J84	Isoform 2 of Kin of IRRE-like protein 1	IDGGPVILLQAGTPHNLTCR	HexNAc(5)Hex(5)Fuc(3)	4.323	3.71.E-02
Q96JE9	Microtubule-associated protein 6	NESPVISAPVK	HexNAc(4)Hex(5)	100	1.00.E-17
Q96JN0	Isoform 3 of Ligand-dependent corepressor	PNLSSSPR	HexNAc(3)Hex(6)Fuc(1)	35.817	1.17.E-06
Q96KM6	Zinc finger protein 512B	LNYTR	HexNAc(3)Hex(6)NeuAc(1)	100	1.00.E-17
Q96M96	FYVE, RhoGEF and PH domain-containing protein 4	NMTER	HexNAc(2)Hex(8)	100	1.00.E-17
Q96MM7	Isoform 4 of Heparan-sulfate 6-O-sulfotransferase 2	YNFTR	HexNAc(5)Hex(5)Fuc(3)	9.374	8.69.E-04
Q96MM7	Isoform 4 of Heparan-sulfate 6-O-sulfotransferase 2	YNFTR	HexNAc(2)Hex(8)	8.343	2.32.E-03
Q96MM7	Isoform 4 of Heparan-sulfate 6-O-sulfotransferase 2	YNFTR	HexNAc(6)Hex(3)Fuc(1)NeuAc(1)	100	1.00.E-17
Q96MM7	Isoform 4 of Heparan-sulfate 6-O-sulfotransferase 2	YNFTR	HexNAc(5)Hex(4)Fuc(2)NeuAc(1)	100	1.00.E-17
Q96MM7	Isoform 4 of Heparan-sulfate 6-O-sulfotransferase 2	YNFTR	HexNAc(5)Hex(4)Fuc(2)	100	1.00.E-17

Q96N03	V-set and transmembrane domain-containing protein 2-like protein	VVGSNISHK	HexNAc(2)Hex(8)	100	1.00.E-17
Q96PQ0	VPS10 domain-containing receptor SorCS2	YVTCAIHNCSEK	HexNAc(4)Hex(5)Fuc(2)NeuAc(1)	100	1.00.E-17
Q96PQ0	VPS10 domain-containing receptor SorCS2	YVTCAIHNCSEK	HexNAc(2)Hex(8)	100	1.00.E-17
Q96PY6	Isoform 3 of Serine/threonine-protein kinase Nek1	LGPNGSPR	HexNAc(4)Hex(4)Fuc(1)	7.349	3.70.E-03
Q96QF7	Acidic repeat-containing protein	IYDLFNR	HexNAc(3)Hex(6)Fuc(1)NeuAc(1)	4.939	2.34.E-02
Q96QF7	Acidic repeat-containing protein	IYDLFNR	HexNAc(5)Hex(4)Fuc(2)	100	1.00.E-17
Q96QF7	Acidic repeat-containing protein	IYDLFNR	HexNAc(3)Hex(6)Fuc(1)	100	1.00.E-17
Q96SM3	Probable carboxypeptidase X1	VPGSTPALHSSPAQPPAETANGTSEQHVR	HexNAc(2)Hex(8)	100	1.00.E-17
Q99435	Isoform 3 of Protein kinase C-binding protein NELL2	QVPGLHNGTK	HexNAc(2)Hex(9)	4.901	1.12.E-02
Q99523	Sortilin	DITDLINNTFIR	HexNAc(4)Hex(5)Fuc(1)NeuAc(1)	100	1.00.E-17
Q99523	Sortilin	DITDLINNTFIR	HexNAc(5)Hex(5)Fuc(3)	16.89	4.02.E-05
Q99523	Sortilin	DITDLINNTFIR	HexNAc(5)Hex(4)Fuc(2)	9.401	3.68.E-03
Q99523	Sortilin	LANNTHQHVFDDLR	HexNAc(4)Hex(5)Fuc(1)NeuAc(2)	5.357	1.72.E-02
Q99985	Semaphorin-3C	VIQTFNR	HexNAc(2)Hex(7)	100	1.00.E-17
Q9BU23	Lipase maturation factor 2	SANSTLAQALHWTR	HexNAc(2)Hex(6)	5.278	2.47.E-03
Q9BX67	Junctional adhesion molecule C	IWNVTR	HexNAc(5)Hex(5)Fuc(3)	100	1.00.E-17
Q9BX67	Junctional adhesion molecule C	IWNVTR	HexNAc(4)Hex(5)Fuc(3)	100	1.00.E-17
Q9BX67	Junctional adhesion molecule C	NSSFHLNSETGTLVFTAVHK	HexNAc(2)Hex(5)	100	1.00.E-17
Q9BX67	Junctional adhesion molecule C	NSSFHLNSETGTLVFTAVHK	HexNAc(2)Hex(7)	4.062	4.16.E-02
Q9BX67	Junctional adhesion molecule C	IWNVTR	HexNAc(4)Hex(5)Fuc(2)NeuAc(1)	3.801	1.97.E-03
Q9BX67	Junctional adhesion molecule C	NSSFHLNSETGTLVFTAVHK	HexNAc(3)Hex(6)Fuc(1)	11.83	6.36.E-04
Q9BX67	Junctional adhesion molecule C	NSSFHLNSETGTLVFTAVHK	HexNAc(3)Hex(6)Fuc(1)NeuAc(1)	5.804	6.29.E-03
Q9BY67	Isoform 5 of Cell adhesion molecule 1	DVTVIEGEVATISCQVNK	HexNAc(6)Hex(4)Fuc(2)	100	1.00.E-17
Q9BYH1	Seizure 6-like protein	MTVHSGQTNK	HexNAc(2)Hex(9)	100	1.00.E-17
Q9BZC7	Isoform 3 of ATP-binding cassette sub-family A member 2	GNPAAYGITVTNHPMNK	HexNAc(2)Hex(9)	100	1.00.E-17
Q9C0A6	SET domain-containing protein 5	PQENISSR	HexNAc(7)Hex(3)	10.933	7.26.E-05
Q9C0A6	SET domain-containing protein 5	PQENISSR	HexNAc(3)Hex(4)Fuc(2)	100	1.00.E-17
Q9C0H2	Isoform 4 of Protein tweety homolog 3	VWDTAVGLNHTAEPSLQTLER	HexNAc(4)Hex(3)Fuc(1)	100	1.00.E-17
Q9C0H2	Isoform 4 of Protein tweety homolog 3	VWDTAVGLNHTAEPSLQTLER	HexNAc(4)Hex(5)Fuc(3)NeuAc(2)	9.665	5.67.E-03
Q9C0H2	Isoform 4 of Protein tweety homolog 3	VWDTAVGLNHTAEPSLQTLER	HexNAc(3)Hex(4)Fuc(1)NeuAc(1)	7.656	1.20.E-02
Q9H1J7	Protein Wnt-5b	WNCSTADNASVFGR	HexNAc(2)Hex(8)	100	1.00.E-17
Q9H1J7	Protein Wnt-5b	WNCSTADNASVFGR	HexNAc(2)Hex(9)	100	1.00.E-17
Q9H2D6	TRIO and F-actin-binding protein	NSSPHR	HexNAc(3)Hex(6)Fuc(1)NeuAc(1)	10.636	1.33.E-03
Q9H2D6	TRIO and F-actin-binding protein	ASSPNRTIQQENLR	HexNAc(2)Hex(5)	100	1.00.E-17
Q9H2E6	Isoform 2 of Semaphorin-6A	NTTQRHR	HexNAc(5)Hex(3)Fuc(1)	9.378	3.66.E-03
Q9H330	Transmembrane protein 245	VNNTAVIEK	HexNAc(2)Hex(9)	4.815	1.21.E-02
Q9H488	GDP-fucose protein O-fucosyltransferase 1	LLNR	HexNAc(2)Hex(6)	3.641	3.84.E-02
Q9H488	GDP-fucose protein O-fucosyltransferase 1	LLNR	HexNAc(2)Hex(9)	2.418	2.48.E-02
Q9H6L2	Isoform 2 of Transmembrane protein 231	NVTTVLNDPNPIWLVGR	HexNAc(2)Hex(9)	6.838	4.79.E-03
Q9H7E2	Isoform 3 of Tudor domain-containing protein 3	NDTR	HexNAc(4)Hex(7)Fuc(1)	5.256	2.75.E-02
Q9H7E2	Isoform 3 of Tudor domain-containing protein 3	NDTR	HexNAc(4)Hex(5)NeuAc(2)	100	1.00.E-17
Q9HAR2	Isoform 4 of Adhesion G protein-coupled receptor L3	NLTPGGK	HexNAc(5)Hex(4)Fuc(2)	100	1.00.E-17
Q9HAR2	Isoform 4 of Adhesion G protein-coupled receptor L3	QSEENFNPNCSFWSYSK	HexNAc(2)Hex(8)	100	1.00.E-17
Q9HAR2	Isoform 4 of Adhesion G protein-coupled receptor L3	LLTTNK	HexNAc(2)Hex(9)	14.24	7.53.E-05
Q9HAR2	Isoform 4 of Adhesion G protein-coupled receptor L3	LLTTNK	HexNAc(2)Hex(8)	12.843	3.82.E-04
Q9HAR2	Isoform 4 of Adhesion G protein-coupled receptor L3	NLTPGGK	HexNAc(5)Hex(5)Fuc(3)	12.027	2.14.E-03
Q9HAR2	Isoform 4 of Adhesion G protein-coupled receptor L3	LLTTNK	HexNAc(2)Hex(7)	9.3	1.10.E-02
Q9HAR2	Isoform 4 of Adhesion G protein-coupled receptor L3	LLTTNK	HexNAc(2)Hex(6)	100	1.00.E-17
Q9HAR2	Isoform 4 of Adhesion G protein-coupled receptor L3	NLTPGGK	HexNAc(2)Hex(5)	100	1.00.E-17
Q9HCM1	Uncharacterized protein KIAA1551	QTPTVVESAETNK	HexNAc(3)Hex(4)	21.747	2.48.E-07
Q9HCU4	Cadherin EGF LAG seven-pass G-type receptor 2	LQRNESGLDSGR	HexNAc(5)Hex(4)Fuc(2)	100	1.00.E-17
Q9HCU4	Cadherin EGF LAG seven-pass G-type receptor 2	NESGLDSGR	HexNAc(4)Hex(3)	100	1.00.E-17
Q9HCU4	Cadherin EGF LAG seven-pass G-type receptor 2	NATQHTAGYFGSDVK	HexNAc(2)Hex(8)	11.944	4.00.E-04
Q9HCU4	Cadherin EGF LAG seven-pass G-type receptor 2	NATQHTAGYFGSDVK	HexNAc(2)Hex(7)	7.999	1.27.E-04
Q9HCU4	Cadherin EGF LAG seven-pass G-type receptor 2	LYLNR	HexNAc(2)Hex(8)	4.776	1.28.E-02
Q9HCU4	Cadherin EGF LAG seven-pass G-type receptor 2	LYLNR	HexNAc(2)Hex(9)	4.466	4.98.E-02
Q9HCU4	Cadherin EGF LAG seven-pass G-type receptor 2	LYLNR	HexNAc(2)Hex(5)	4.054	4.58.E-02
Q9HCU4	Cadherin EGF LAG seven-pass G-type receptor 2	HIDMADFIANNGTVPGCPAK	HexNAc(2)Hex(6)	3.528	3.97.E-02
Q9HCU4	Cadherin EGF LAG seven-pass G-type receptor 2	LQRNESGLDSGR	HexNAc(5)Hex(5)Fuc(3)	100	1.00.E-17
Q9HCU4	Cadherin EGF LAG seven-pass G-type receptor 2	LQRNESGLDSGR	HexNAc(4)Hex(5)Fuc(2)NeuAc(1)	8.756	3.47.E-03
Q9HD45	Transmembrane 9 superfamily member 3	IVDVNLTSEGK	HexNAc(5)Hex(4)NeuAc(1)	100	1.00.E-17
Q9HD45	Transmembrane 9 superfamily member 3	IVDVNLTSEGK	HexNAc(5)Hex(4)	100	1.00.E-17
Q9HD45	Transmembrane 9 superfamily member 3	IVDVNLTSEGK	HexNAc(5)Hex(3)	100	1.00.E-17
Q9HD45	Transmembrane 9 superfamily member 3	IVDVNLTSEGK	HexNAc(4)Hex(6)Fuc(1)NeuAc(1)	3.854	3.80.E-02
Q9HD45	Transmembrane 9 superfamily member 3	IVDVNLTSEGK	HexNAc(3)Hex(5)NeuAc(1)	3.022	4.45.E-02
Q9HD45	Transmembrane 9 superfamily member 3	IVDVNLTSEGK	HexNAc(3)Hex(6)Fuc(1)	6.277	2.60.E-02
Q9HDC9	Adipocyte plasma membrane-associated protein	NMSFVNDLTVTQDGR	HexNAc(2)Hex(6)	4.921	9.14.E-03
Q9HDC9	Adipocyte plasma membrane-associated protein	NMSFVNDLTVTQDGR	HexNAc(2)Hex(6)	4.017	6.23.E-03
Q9HDC9	Adipocyte plasma membrane-associated protein	NMSFVNDLTVTQDGRK	HexNAc(2)Hex(6)	3.591	9.93.E-03
Q9HDC9	Adipocyte plasma membrane-associated protein	AGPNGTLFVADAYK	HexNAc(2)Hex(6)	2.218	1.16.E-02
Q9HDC9	Adipocyte plasma membrane-associated protein	NMSFVNDLTVTQDGRK	HexNAc(2)Hex(5)	100	1.00.E-17
Q9HDC9	Adipocyte plasma membrane-associated protein	NMSFVNDLTVTQDGR	HexNAc(2)Hex(5)	100	1.00.E-17
Q9HDC9	Adipocyte plasma membrane-associated protein	NMSFVNDLTVTQDGRK	HexNAc(2)Hex(6)	8.656	5.02.E-03
Q9NPR2	Semaphorin-4B	FEAEHISNYTALLLSR	HexNAc(2)Hex(8)	7.293	1.27.E-02
Q9NQZ3	Isoform 2 of Deleted in azoospermia protein 1	IITNR	HexNAc(3)Hex(5)NeuAc(1)	10.741	7.65.E-04
Q9NT68	Teneurin-2	LGPYANTTK	HexNAc(5)Hex(4)Fuc(2)	100	1.00.E-17
Q9NT68	Teneurin-2	DSTHIIPGENPFNSSLVSLIR	HexNAc(2)Hex(7)	100	1.00.E-17
Q9NT99	Leucine-rich repeat-containing protein 4B	TGTSMTSVNWLTPNGTLMTHGSYR	HexNAc(2)Hex(5)	100	1.00.E-17
Q9NUN5	Probable lysosomal cobalamin transporter	NQNGTFK	HexNAc(2)Hex(5)	7.202	2.23.E-02
Q9NX58	Cell growth-regulating nucleolar protein	PNVSPK	HexNAc(4)Hex(4)NeuAc(1)	100	1.00.E-17
Q9NXF1	Testis-expressed protein 10	NITTLK	HexNAc(5)Hex(6)Fuc(1)	100	1.00.E-17
Q9NXF1	Testis-expressed protein 10	NITTLK	HexNAc(2)Hex(9)	100	1.00.E-17
Q9NYQ7	Isoform 2 of Cadherin EGF LAG seven-pass G-type receptor 3	GFDPNCNK	HexNAc(2)Hex(5)	100	1.00.E-17
Q9NZ53	Podocalyxin-like protein 2	SLEEIGIQNYSTTSSCQAR	HexNAc(2)Hex(5)	5.168	2.48.E-02
Q9NZN1	Interleukin-1 receptor accessory protein-like 1	EVREDDIGNYTCELK	HexNAc(2)Hex(7)	100	1.00.E-17
Q9P0K1	Isoform 3 of Disintegrin and metalloproteinase domain-containing protein 22	TLNCSGGHVK	HexNAc(2)Hex(5)	6.71	2.49.E-02
Q9P0K1	Isoform 3 of Disintegrin and metalloproteinase domain-containing protein 22	ETCSGNSSQCAPNIHK	HexNAc(2)Hex(5)	4.101	4.73.E-02
Q9P244	Leucine-rich repeat and fibronectin type III domain-containing protein 1	LLGNSSR	HexNAc(2)Hex(7)	8.979	6.84.E-03
Q9P273	Teneurin-3	IGPFANTTK	HexNAc(5)Hex(5)Fuc(3)	100	1.00.E-17
Q9P273	Teneurin-3	FNISLQK	HexNAc(5)Hex(5)Fuc(2)	11.211	8.03.E-04
Q9P273	Teneurin-3	FNISLQK	HexNAc(3)Hex(6)Fuc(1)	100	1.00.E-17
Q9P273	Teneurin-3	FNISLQK	HexNAc(2)Hex(5)	100	1.00.E-17
Q9P273	Teneurin-3	LTNVTFPTGVVTNLHGDMDK	HexNAc(2)Hex(9)	3.738	3.51.E-02
Q9P2B2	Prostaglandin F2 receptor negative regulator	ELDLTCNITTDR	HexNAc(2)Hex(5)	100	1.00.E-17
Q9P2B2	Prostaglandin F2 receptor negative regulator	NVSVAEGK	HexNAc(5)Hex(5)Fuc(1)	100	1.00.E-17
Q9P2K2	Thioredoxin domain-containing protein 16	HWNR	HexNAc(3)Hex(6)	100	1.00.E-17
Q9UBG0	C-type mannose receptor 2	TSNISKPGTLER	HexNAc(5)Hex(5)Fuc(3)	100	1.00.E-17

Q9UBG0	C-type mannose receptor 2	VTPACNTSLPAQR	HexNAc(5)Hex(5)Fuc(3)	100	1.00.E-17
Q9UBG0	C-type mannose receptor 2	VTPACNTSLPAQR	HexNAc(5)Hex(5)Fuc(2)	100	1.00.E-17
Q9UBG0	C-type mannose receptor 2	WNDSPCNQSLPSICK	HexNAc(5)Hex(5)Fuc(1)NeuAc(1)	100	1.00.E-17
Q9UBG0	C-type mannose receptor 2	KKPNATAEPTPPDR	HexNAc(4)Hex(5)Fuc(3)NeuAc(2)	100	1.00.E-17
Q9UBG0	C-type mannose receptor 2	NCTSFR	HexNAc(5)Hex(4)Fuc(2)	10.082	4.49.E-03
Q9UBG0	C-type mannose receptor 2	NCTSFR	HexNAc(2)Hex(7)	8.39	4.41.E-03
Q9UBG0	C-type mannose receptor 2	WNDSPCNQSLPSICK	HexNAc(5)Hex(5)Fuc(3)	6.56	2.76.E-02
Q9UBG0	C-type mannose receptor 2	NCTSFR	HexNAc(2)Hex(5)	6.361	9.71.E-04
Q9UBG0	C-type mannose receptor 2	KKPNATAEPTPPDR	HexNAc(4)Hex(5)Fuc(2)NeuAc(1)	5.61	1.97.E-02
Q9UBG0	C-type mannose receptor 2	KKPNATAEPTPPDR	HexNAc(4)Hex(5)Fuc(1)NeuAc(2)	4.851	3.73.E-02
Q9UBG0	C-type mannose receptor 2	NCTSFR	HexNAc(2)Hex(6)	4.276	1.46.E-02
Q9UBG0	C-type mannose receptor 2	NCTSFR	HexNAc(4)Hex(5)Fuc(3)	100	1.00.E-17
Q9UBG0	C-type mannose receptor 2	VTPACNTSLPAQR	HexNAc(4)Hex(5)Fuc(3)	100	1.00.E-17
Q9UBG0	C-type mannose receptor 2	VTPACNTSLPAQR	HexNAc(3)Hex(5)NeuAc(1)	9.466	3.81.E-03
Q9UBG0	C-type mannose receptor 2	NCTSFR	HexNAc(2)Hex(8)	5.492	2.16.E-02
Q9UBG0	C-type mannose receptor 2	TSNISKPGTLER	HexNAc(4)Hex(5)Fuc(1)NeuAc(2)	100	1.00.E-17
Q9UBG0	C-type mannose receptor 2	VTPACNTSLPAQR	HexNAc(3)Hex(6)Fuc(1)NeuAc(1)	100	1.00.E-17
Q9UBG0	C-type mannose receptor 2	GTDPSLSPSPAALPPAPGTELSYL NGTFR	HexNAc(3)Hex(5)	100	1.00.E-17
Q9UBG0	C-type mannose receptor 2	GTDPSLSPSPAALPPAPGTELSYL NGTFR	HexNAc(2)Hex(7)	100	1.00.E-17
Q9UBG0	C-type mannose receptor 2	WNDSPCNQSLPSICK	HexNAc(4)Hex(5)Fuc(2)NeuAc(1)	6.942	1.08.E-02
Q9UBS5	Isoform 1C of Gamma-aminobutyric acid type B receptor subunit 1	SISNMTSQEFVEK	HexNAc(5)Hex(3)Fuc(1)	100	1.00.E-17
Q9UBV2	Protein sel-1 homolog 1	EASIVGENETYPR	HexNAc(2)Hex(7)	100	1.00.E-17
Q9UH99	Isoform 2 of SUN domain-containing protein 2	ALSPNSTISSAPK	HexNAc(2)Hex(5)	100	1.00.E-17
Q9UH99	Isoform 2 of SUN domain-containing protein 2	ALSPNSTISSAPK	HexNAc(2)Hex(6)	5.083	1.28.E-02
Q9UHG0	Doublecortin domain-containing protein 2	INVSAR	HexNAc(5)Hex(5)Fuc(2)	100	1.00.E-17
Q9UHG0	Doublecortin domain-containing protein 2	INVSAR	HexNAc(5)Hex(4)Fuc(2)	10.138	4.01.E-03
Q9UHG0	Doublecortin domain-containing protein 2	INVSAR	HexNAc(5)Hex(5)Fuc(3)	100	1.00.E-17
Q9UHG3	Prenylcysteine oxidase 1	LLHALGGDDFLGMLNR	HexNAc(2)Hex(9)	16.01	1.92.E-03
Q9UHG3	Prenylcysteine oxidase 1	GELNTSIFSSR	HexNAc(2)Hex(9)	9.204	1.76.E-03
Q9UHG3	Prenylcysteine oxidase 1	GELNTSIFSSRPIDK	HexNAc(2)Hex(8)	4.477	9.39.E-05
Q9UHG3	Prenylcysteine oxidase 1	GELNTSIFSSRPIDK	HexNAc(2)Hex(9)	4.066	2.02.E-04
Q9UHG3	Prenylcysteine oxidase 1	LLHALGGDDFLGMLNR	HexNAc(2)Hex(9)	100	1.00.E-17
Q9UHG3	Prenylcysteine oxidase 1	LLHALGGDDFLGMLNR	HexNAc(2)Hex(7)	100	1.00.E-17
Q9UHG3	Prenylcysteine oxidase 1	GELNTSIFSSRPIDK	HexNAc(2)Hex(5)	100	1.00.E-17
Q9UHN6	Cell surface hyaluronidase	QNGSLSK	HexNAc(5)Hex(4)Fuc(2)	100	1.00.E-17
Q9UHN6	Cell surface hyaluronidase	HPSCVNVSK	HexNAc(2)Hex(7)	9.206	3.15.E-03
Q9UHN6	Cell surface hyaluronidase	HPSCVNVSK	HexNAc(2)Hex(6)	8.181	5.92.E-03
Q9UIQ6	Leucyl-cystinyl aminopeptidase	NQSIGLIQPFATNGK	HexNAc(2)Hex(8)	3.309	4.39.E-02
Q9UIW2	Plexin-A1	YNYTEDPTILR	HexNAc(5)Hex(4)Fuc(2)	18.16	1.39.E-04
Q9UIW2	Plexin-A1	ENGCLVYNDTTMVCR	HexNAc(2)Hex(5)	100	1.00.E-17
Q9UIW2	Plexin-A1	VNVSEDCPQILPSTQIYVPVGVVKP ITLAAR	HexNAc(2)Hex(5)	4.719	2.03.E-02
Q9UKY4	Protein O-mannosyl-transferase 2	MGSYYINR	HexNAc(2)Hex(9)	8.002	2.29.E-03
Q9UL58	Zinc finger protein 215	TNLTK	HexNAc(5)Hex(4)Fuc(2)NeuAc(1)	12.115	4.86.E-04
Q9UL58	Zinc finger protein 215	TNLTK	HexNAc(5)Hex(5)Fuc(1)NeuAc(1)	5.977	1.79.E-02
Q9ULF5	Zinc transporter ZIP10	DLNEDDHHHECLNVTQLLK	HexNAc(2)Hex(5)	11.843	5.12.E-04
Q9ULJ8	Isoform 3 of Neurabin-1	LENWTPK	HexNAc(6)Hex(4)Fuc(1)	100	1.00.E-17
Q9ULK0	Glutamate receptor ionotropic, delta-1	GLNGSLQER	HexNAc(4)Hex(5)NeuAc(1)	100	1.00.E-17
Q9ULK0	Glutamate receptor ionotropic, delta-1	GLNGSLQER	HexNAc(2)Hex(5)	100	1.00.E-17
Q9UN79	Transcription factor SOX-13	NSSHIK	HexNAc(6)Hex(3)Fuc(2)	100	1.00.E-17
Q9UPW6	DNA-binding protein SATB2	VAFNR	HexNAc(5)Hex(5)Fuc(3)	9.68	5.82.E-03
Q9UPW6	DNA-binding protein SATB2	VAFNR	HexNAc(5)Hex(3)Fuc(1)	3.958	3.76.E-02
Q9UQ35	Serine/arginine repetitive matrix protein 2	TPPVALNSSR	HexNAc(3)Hex(3)Fuc(1)	10.271	6.02.E-04
Q9Y4A5	Transformation/transcription domain-associated protein	NYSIAQK	HexNAc(5)Hex(4)Fuc(2)	100	1.00.E-17
Q9Y4C0	Neurexin-3	INGSVR	HexNAc(2)Hex(5)	100	1.00.E-17
Q9Y597	BTB/POZ domain-containing protein KCTD3	KINNTVR	HexNAc(2)Hex(6)	42.008	2.68.E-05
Q9Y5F9	Protocadherin gamma-B6	DHGSPTLSANVSLR	HexNAc(5)Hex(4)Fuc(2)	4.611	4.81.E-02
Q9Y639	Neuroplastin	ANATIEVK	HexNAc(5)Hex(4)Fuc(1)	100	1.00.E-17
Q9Y639	Neuroplastin	NASNMEYR	HexNAc(3)Hex(6)Fuc(1)NeuAc(1)	100	1.00.E-17
Q9Y666	Solute carrier family 12 member 7	SAFDPPDIPVCLLGNR	HexNAc(2)Hex(8)	100	1.00.E-17
Q9Y666	Solute carrier family 12 member 7	SAFDPPDIPVCLLGNR	HexNAc(2)Hex(9)	3.54	1.19.E-02
Q9Y6M1	Insulin-like growth factor 2 mRNA-binding protein 2	NLTK	HexNAc(5)Hex(6)Fuc(2)	100	1.00.E-17
Q9Y6M7	Isoform 7 of Sodium bicarbonate cotransporter 3	NLTVSECK	HexNAc(3)Hex(6)Fuc(1)NeuAc(1)	100	1.00.E-17
Q9Y6N7	Isoform 2 of Roundabout homolog 1	YHINK	HexNAc(5)Hex(5)Fuc(3)	5.737	3.42.E-02
Q9Y6X0	SET-binding protein	KMCNYTK	HexNAc(2)Hex(7)	100	1.00.E-17
Q9Y6X5	Bis(5'-adenosyl)-triphosphatase ENPP4	ENMSR	HexNAc(7)Hex(3)	100	1.00.E-17

Supplementary Table S3. (b) N-glycopeptides with increased numbers in the NPCs (>2 fold)

Gene Name	Protein name	Sequence	Glycan Composition	Abundance Ratio: (NPC) / (iPSC)	Abundance Ratio p-value: (NPC) / (iPSC)
P67809	Nuclease-sensitive element-binding protein 1	NDTK	HexNAc(9)Hex(3)	100.000	1.00.E-17
P05026	Sodium/potassium-transporting ATPase subunit beta-1	NESLETYPVMK	HexNAc(8)Hex(9)Fuc(1)	100.000	1.00.E-17
Q92896	Isoform 2 of Golgi apparatus protein 1	GNITEYQCHQYITK	HexNAc(8)Hex(5)Fuc(1)	100.000	1.00.E-17
Q8NFC6	Biorientation of chromosomes in cell division protein 1-like 1	NYTK	HexNAc(8)Hex(4)	100.000	1.00.E-17
P33981	Dual specificity protein kinase TTK	QTNK	HexNAc(8)Hex(3)	100.000	1.00.E-17
P05556	Isoform 5 of Integrin beta-1	LRNPCTSEQNCTSPFSYK	HexNAc(7)Hex(7)Fuc(1)	100.000	1.00.E-17
O00533	Neural cell adhesion molecule L1-like protein	QNRSVR	HexNAc(7)Hex(4)Fuc(1)	100.000	1.00.E-17
P07195	L-lactate dehydrogenase B chain	NISR	HexNAc(7)Hex(3)	23.226	1.28.E-04
Q9C0A6	SET domain-containing protein 5	PQENISSR	HexNAc(7)Hex(3)	7.106	5.12.E-03
P05026	Sodium/potassium-transporting ATPase subunit beta-1	NESLETYPVMK	HexNAc(6)Hex(7)NeuAc(4)	100.000	1.00.E-17
P11166	Solute carrier family 2, facilitated glucose transporter member 1	VIEEFYNQTWVHR	HexNAc(6)Hex(7)NeuAc(3)	4.082	1.29.E-02
P05556	Isoform 5 of Integrin beta-1	CHEGNGTFECGACR	HexNAc(6)Hex(7)NeuAc(2)	100.000	1.00.E-17
P08195	4F2 cell-surface antigen heavy chain	DASSFLAEWQNITK	HexNAc(6)Hex(7)Fuc(1)NeuAc(3)	100.000	1.00.E-17
P05556	Isoform 5 of Integrin beta-1	KDTCTQECSYFNITK	HexNAc(6)Hex(7)Fuc(1)NeuAc(2)	100.000	1.00.E-17
P08195	4F2 cell-surface antigen heavy chain	DASSFLAEWQNITK	HexNAc(6)Hex(7)Fuc(1)NeuAc(2)	7.736	5.27.E-03
P08962	CD63 antigen	NNHTASILDR	HexNAc(6)Hex(7)Fuc(1)NeuAc(2)	100.000	1.00.E-17
P54709	Sodium/potassium-transporting ATPase subunit beta-3	NLTVCPDGALFEQK	HexNAc(6)Hex(7)Fuc(1)NeuAc(2)	100.000	1.00.E-17
P05556	Isoform 5 of Integrin beta-1	LRNPCTSEQNCTSPFSYK	HexNAc(6)Hex(7)Fuc(1)NeuAc(1)	100.000	1.00.E-17
P08962	CD63 antigen	NNHTASILDR	HexNAc(6)Hex(7)Fuc(1)NeuAc(1)	7.689	2.09.E-03
P13473	Isoform LAMP-2C of Lysosome-associated membrane glycoprotein 2	VASVININPNTTHSTGSCR	HexNAc(6)Hex(7)Fuc(1)	100.000	1.00.E-17
Q9NQC3	Reticulon-4	NTSTK	HexNAc(6)Hex(7)	100.000	1.00.E-17
Q92879	Isoform 4 of CUGBP Elav-like family member 1	NDSK	HexNAc(6)Hex(6)NeuAc(1)	100.000	1.00.E-17
P08962	CD63 antigen	NNHTASILDR	HexNAc(6)Hex(6)Fuc(1)NeuAc(2)	100.000	1.00.E-17
P06756	Integrin alpha-V	NMTISR	HexNAc(6)Hex(6)Fuc(1)NeuAc(1)	100.000	1.00.E-17
P08962	CD63 antigen	NNHTASILDR	HexNAc(6)Hex(6)Fuc(1)NeuAc(1)	100.000	1.00.E-17
P26006	Isoform 2 of Integrin alpha-3	MNITVK	HexNAc(6)Hex(6)Fuc(1)NeuAc(1)	8.269	3.76.E-03
Q8IV08	Phospholipase D3	DNHTHSDIQVK	HexNAc(6)Hex(6)Fuc(1)NeuAc(1)	100.000	1.00.E-17
P04216	Thy-1 membrane glycoprotein	TNFTSK	HexNAc(6)Hex(6)Fuc(1)	100.000	1.00.E-17
P05556	Isoform 5 of Integrin beta-1	NPCTSEQNCTSPFSYK	HexNAc(6)Hex(6)Fuc(1)	100.000	1.00.E-17
P05556	Isoform 5 of Integrin beta-1	LRNPCTSEQNCTSPFSYK	HexNAc(6)Hex(6)Fuc(1)	100.000	1.00.E-17
P08962	CD63 antigen	NNHTASILDR	HexNAc(6)Hex(6)Fuc(1)	100.000	1.00.E-17
P13473	Isoform LAMP-2C of Lysosome-associated membrane glycoprotein 2	LNSSTIK	HexNAc(6)Hex(6)Fuc(1)	100.000	1.00.E-17
P42892	Endothelin-converting enzyme 1	DYYLNK	HexNAc(6)Hex(6)Fuc(1)	100.000	1.00.E-17
Q16851	UTP--glucose-1-phosphate uridylyltransferase	NVSLK	HexNAc(6)Hex(6)Fuc(1)	100.000	1.00.E-17
P13473	Isoform LAMP-2C of Lysosome-associated membrane glycoprotein 2	LNSSTIK	HexNAc(6)Hex(6)	100.000	1.00.E-17
P08962	CD63 antigen	NNHTASILDR	HexNAc(6)Hex(5)Fuc(1)NeuAc(2)	100.000	1.00.E-17
P08069	Insulin-like growth factor 1 receptor	NITR	HexNAc(6)Hex(5)Fuc(1)NeuAc(1)	100.000	1.00.E-17
P08962	CD63 antigen	NNHTASILDR	HexNAc(6)Hex(5)Fuc(1)NeuAc(1)	100.000	1.00.E-17
Q8IV08	Phospholipase D3	DNHTHSDIQVK	HexNAc(6)Hex(5)Fuc(1)NeuAc(1)	100.000	1.00.E-17
Q9UPZ6	Thrombospondin type-1 domain-containing protein 7A	YNAQPVGNWSDCILPEGK	HexNAc(6)Hex(5)Fuc(1)NeuAc(1)	100.000	1.00.E-17
P05556	Isoform 5 of Integrin beta-1	NPCTSEQNCTSPFSYK	HexNAc(6)Hex(5)Fuc(1)	6.396	5.93.E-03
Q01638	Interleukin-1 receptor-like 1	SPTFNR	HexNAc(6)Hex(5)Fuc(1)	100.000	1.00.E-17
Q13740	CD166 antigen	EGDNITLK	HexNAc(6)Hex(5)Fuc(1)	100.000	1.00.E-17
Q15758	Neutral amino acid transporter B(0)	NITGTR	HexNAc(6)Hex(5)Fuc(1)	4.017	1.28.E-02
Q9UBG0	C-type mannose receptor 2	NCTSFR	HexNAc(6)Hex(5)Fuc(1)	4.134	1.97.E-02
O94763	Unconventional prefoldin RPB5 interactor 1	NTTLK	HexNAc(6)Hex(5)	100.000	1.00.E-17
P13473	Isoform LAMP-2C of Lysosome-associated membrane glycoprotein 2	LNSSTIK	HexNAc(6)Hex(5)	100.000	1.00.E-17
P23193	Isoform 2 of Transcription elongation factor A protein 1	NASTR	HexNAc(6)Hex(5)	3.734	2.29.E-02
Q13112	Chromatin assembly factor 1 subunit B	INLTPLK	HexNAc(6)Hex(4)NeuAc(1)	100.000	1.00.E-17
Q8TEU7	Zinc finger protein 638	NISEK	HexNAc(6)Hex(4)NeuAc(1)	6.075	5.28.E-03
O75976	Carboxypeptidase D	NVTVK	HexNAc(6)Hex(4)Fuc(2)	100.000	1.00.E-17
P04216	Thy-1 membrane glycoprotein	TNFTSK	HexNAc(6)Hex(4)Fuc(2)	100.000	1.00.E-17
P11279	Lysosome-associated membrane glycoprotein 1	NATR	HexNAc(6)Hex(4)Fuc(2)	100.000	1.00.E-17
P19022	Cadherin-2	NLSLR	HexNAc(6)Hex(4)Fuc(2)	3.050	2.00.E-02
P29323	Ephrin type-B receptor 2	AGFEAVENGTVCR	HexNAc(6)Hex(4)Fuc(2)	100.000	1.00.E-17
Q9ULJ8	Isoform 3 of Neurabin-1	LENWTPK	HexNAc(6)Hex(4)Fuc(1)	100.000	1.00.E-17
Q9Y639	Neuroplastin	ANATIEVK	HexNAc(6)Hex(4)Fuc(1)	100.000	1.00.E-17
A4D1P6	WD repeat-containing protein 91	NASLSQSPR	HexNAc(6)Hex(4)	100.000	1.00.E-17
P67809	Nuclease-sensitive element-binding protein 1	NDTK	HexNAc(6)Hex(3)Fuc(3)	100.000	1.00.E-17
Q15075	Early endosome antigen 1	NQSESHK	HexNAc(6)Hex(3)Fuc(3)	100.000	1.00.E-17
Q86U86	Protein polybromo-1	NYTDK	HexNAc(6)Hex(3)Fuc(2)NeuAc(1)	2.459	4.50.E-02
O95084	Serine protease 23	GANDSTSAMPEQMK	HexNAc(6)Hex(3)Fuc(1)NeuAc(1)	100.000	1.00.E-17
P43121	Cell surface glycoprotein MUC18	CGLSQSQGNLSHVDWFSVHK	HexNAc(6)Hex(3)Fuc(1)NeuAc(1)	12.567	8.48.E-03
Q2KHM9	Protein moonraker	NISEQK	HexNAc(6)Hex(3)Fuc(1)NeuAc(1)	100.000	1.00.E-17
Q96MM7	Isoform 4 of Heparan-sulfate 6-O-sulfotransferase 2	YNFTR	HexNAc(6)Hex(3)Fuc(1)NeuAc(1)	100.000	1.00.E-17
O15394	Neural cell adhesion molecule 2	NTTNLK	HexNAc(6)Hex(3)Fuc(1)	100.000	1.00.E-17
O43852	Isoform 3 of Calumenin	NATYGYVLDDPDPDDGFNYK	HexNAc(6)Hex(3)Fuc(1)	2.385	2.01.E-02
O60637	Tetraspanin-3	TYNGTNPDAASR	HexNAc(6)Hex(3)Fuc(1)	100.000	1.00.E-17
O75054	Isoform 2 of Immunoglobulin superfamily member 3	LSQAQGNLSVLETR	HexNAc(6)Hex(3)Fuc(1)	100.000	1.00.E-17
O75821	Eukaryotic translation initiation factor 3 subunit G	ADDNATIR	HexNAc(6)Hex(3)Fuc(1)	100.000	1.00.E-17
O95864	Fatty acid desaturase 2	KVYNITK	HexNAc(6)Hex(3)Fuc(1)	4.955	8.99.E-03
P29590	Protein PML	NQSVR	HexNAc(6)Hex(3)Fuc(1)	3.057	2.97.E-02
P33981	Dual specificity protein kinase TTK	NSLRQTNK	HexNAc(6)Hex(3)Fuc(1)	100.000	1.00.E-17
P07996	Thrombospondin-1	VSCPIMPCSNATVPDGECCPR	HexNAc(6)Hex(3)	2.437	3.40.E-02
Q9UHF7	Isoform 3 of Zinc finger transcription factor Trps1	NVTWR	HexNAc(5)Hex(7)Fuc(1)	100.000	1.00.E-17
Q9Y6M7	Isoform 7 of Sodium bicarbonate cotransporter 3	NLTVSECK	HexNAc(5)Hex(7)Fuc(1)	100.000	1.00.E-17

P05556	Isoform 5 of Integrin beta-1	NPCTSEQNCTSPFSYK	HexNAc(5)Hex(7)	100.000	1.00.E-17
P08962	CD63 antigen	NNHTASILDR	HexNAc(5)Hex(6)NeuAc(3)	4.166	1.49.E-02
Q9H8H0	Nucleolar protein 11	NQSLVK	HexNAc(5)Hex(6)NeuAc(1)	9.740	1.75.E-03
P05556	Isoform 5 of Integrin beta-1	NPCTSEQNCTSPFSYK	HexNAc(5)Hex(6)Fuc(3)	100.000	1.00.E-17
P05556	Isoform 5 of Integrin beta-1	LRNPCTSEQNCTSPFSYK	HexNAc(5)Hex(6)Fuc(3)	4.186	1.68.E-02
Q08722	Leukocyte surface antigen CD47	SDAVSHTGNYTCEVTELTR	HexNAc(5)Hex(6)Fuc(3)	100.000	1.00.E-17
Q5ZPR3	CD276 antigen	QLVHSFAEGQDQGSAYANR	HexNAc(5)Hex(6)Fuc(3)	100.000	1.00.E-17
Q8IWB1	Inositol 1,4,5-trisphosphate receptor-interacting protein	ENATVPENEEEIIRK	HexNAc(5)Hex(6)Fuc(3)	100.000	1.00.E-17
P05026	Sodium/potassium-transporting ATPase subunit beta-1	LEWLGNCSGLNDETYGYK	HexNAc(5)Hex(6)Fuc(2)NeuAc(1)	100.000	1.00.E-17
O75976	Carboxypeptidase D	LLNTTDVYLLPSLNPDGFER	HexNAc(5)Hex(6)Fuc(2)	5.625	7.34.E-03
P05556	Isoform 5 of Integrin beta-1	NPCTSEQNCTSPFSYK	HexNAc(5)Hex(6)Fuc(2)	100.000	1.00.E-17
Q07954	Prolow-density lipoprotein receptor-related protein 1	LTSCATNASICGDEAR	HexNAc(5)Hex(6)Fuc(2)	100.000	1.00.E-17
Q9UBG0	C-type mannose receptor 2	VTPACNTSLPAQR	HexNAc(5)Hex(6)Fuc(2)	100.000	1.00.E-17
Q9Y6M1	Insulin-like growth factor 2 mRNA-binding protein 2	NLTK	HexNAc(5)Hex(6)Fuc(2)	100.000	1.00.E-17
P08962	CD63 antigen	NNHTASILDR	HexNAc(5)Hex(6)Fuc(1)NeuAc(3)	2.885	2.04.E-02
P35613	Basigin	ILLTCSLNDSATEVTGHR	HexNAc(5)Hex(6)Fuc(1)NeuAc(3)	100.000	1.00.E-17
Q13641	Trophoblast glycoprotein	VLHNGTLAELQGLPHIR	HexNAc(5)Hex(6)Fuc(1)NeuAc(3)	5.142	7.76.E-03
Q8IV08	Phospholipase D3	DNHTHSDIQVK	HexNAc(5)Hex(6)Fuc(1)NeuAc(3)	100.000	1.00.E-17
Q9UBG0	C-type mannose receptor 2	KKPNATAEPTPPDR	HexNAc(5)Hex(6)Fuc(1)NeuAc(3)	100.000	1.00.E-17
P02786	Transferrin receptor protein 1	DFEDLYTPVNGSIVIVR	HexNAc(5)Hex(6)Fuc(1)NeuAc(2)	39.327	2.31.E-05
P05026	Sodium/potassium-transporting ATPase subunit beta-1	LEWLGNCSGLNDETYGYK	HexNAc(5)Hex(6)Fuc(1)NeuAc(2)	100.000	1.00.E-17
P05556	Isoform 5 of Integrin beta-1	KDTCTQECSYFNITK	HexNAc(5)Hex(6)Fuc(1)NeuAc(2)	6.273	9.28.E-03
P08962	CD63 antigen	NNHTASILDR	HexNAc(5)Hex(6)Fuc(1)NeuAc(2)	8.028	7.80.E-04
P13473	Isoform LAMP-2C of Lysosome-associated membrane glycoprotein 2	VASVININPNTTHSTGSCR	HexNAc(5)Hex(6)Fuc(1)NeuAc(2)	100.000	1.00.E-17
P26006	Isoform 2 of Integrin alpha-3	MNITVK	HexNAc(5)Hex(6)Fuc(1)NeuAc(2)	100.000	1.00.E-17
P54709	Sodium/potassium-transporting ATPase subunit beta-3	NLTVCPDGALFEQK	HexNAc(5)Hex(6)Fuc(1)NeuAc(2)	100.000	1.00.E-17
Q07954	Prolow-density lipoprotein receptor-related protein 1	LNLDGSNYTLLK	HexNAc(5)Hex(6)Fuc(1)NeuAc(2)	100.000	1.00.E-17
Q07954	Prolow-density lipoprotein receptor-related protein 1	MHLNGSNVQVLHR	HexNAc(5)Hex(6)Fuc(1)NeuAc(2)	100.000	1.00.E-17
Q13641	Trophoblast glycoprotein	VLHNGTLAELQGLPHIR	HexNAc(5)Hex(6)Fuc(1)NeuAc(2)	100.000	1.00.E-17
Q5ZPR3	CD276 antigen	VVLGANGTYSCLVR	HexNAc(5)Hex(6)Fuc(1)NeuAc(2)	100.000	1.00.E-17
Q9BX67	Junctional adhesion molecule C	IWNVTR	HexNAc(5)Hex(6)Fuc(1)NeuAc(2)	100.000	1.00.E-17
Q9UBG0	C-type mannose receptor 2	KKPNATAEPTPPDR	HexNAc(5)Hex(6)Fuc(1)NeuAc(2)	100.000	1.00.E-17
P05026	Sodium/potassium-transporting ATPase subunit beta-1	LEWLGNCSGLNDETYGYK	HexNAc(5)Hex(6)Fuc(1)NeuAc(1)	100.000	1.00.E-17
P05556	Isoform 5 of Integrin beta-1	NPCTSEQNCTSPFSYK	HexNAc(5)Hex(6)Fuc(1)NeuAc(1)	100.000	1.00.E-17
P05556	Isoform 5 of Integrin beta-1	DTCTQECSYFNITK	HexNAc(5)Hex(6)Fuc(1)NeuAc(1)	100.000	1.00.E-17
P05556	Isoform 5 of Integrin beta-1	KDTCTQECSYFNITK	HexNAc(5)Hex(6)Fuc(1)NeuAc(1)	100.000	1.00.E-17
P05556	Isoform 5 of Integrin beta-1	LRNPCTSEQNCTSPFSYK	HexNAc(5)Hex(6)Fuc(1)NeuAc(1)	4.186	1.68.E-02
P08962	CD63 antigen	NNHTASILDR	HexNAc(5)Hex(6)Fuc(1)NeuAc(1)	4.104	1.93.E-02
P13473	Isoform LAMP-2C of Lysosome-associated membrane glycoprotein 2	VASVININPNTTHSTGSCR	HexNAc(5)Hex(6)Fuc(1)NeuAc(1)	100.000	1.00.E-17
Q07954	Prolow-density lipoprotein receptor-related protein 1	LNLDGSNYTLLK	HexNAc(5)Hex(6)Fuc(1)NeuAc(1)	100.000	1.00.E-17
Q08722	Leukocyte surface antigen CD47	SDAVSHTGNYTCEVTELTR	HexNAc(5)Hex(6)Fuc(1)NeuAc(1)	100.000	1.00.E-17
Q5ZPR3	CD276 antigen	VVLGANGTYSCLVR	HexNAc(5)Hex(6)Fuc(1)NeuAc(1)	3.156	3.19.E-02
Q9UBG0	C-type mannose receptor 2	KKPNATAEPTPPDR	HexNAc(5)Hex(6)Fuc(1)NeuAc(1)	8.708	1.60.E-03
Q9UBG0	C-type mannose receptor 2	VTPACNTSLPAQR	HexNAc(5)Hex(6)Fuc(1)NeuAc(1)	4.402	7.32.E-03
P05556	Isoform 5 of Integrin beta-1	NPCTSEQNCTSPFSYK	HexNAc(5)Hex(6)Fuc(1)	100.000	1.00.E-17
P05556	Isoform 5 of Integrin beta-1	LRNPCTSEQNCTSPFSYK	HexNAc(5)Hex(6)Fuc(1)	100.000	1.00.E-17
P06756	Integrin alpha-V	NMTISR	HexNAc(5)Hex(6)Fuc(1)	100.000	1.00.E-17
P06756	Integrin alpha-V	ENQNHSYSLK	HexNAc(5)Hex(6)Fuc(1)	100.000	1.00.E-17
P13473	Isoform LAMP-2C of Lysosome-associated membrane glycoprotein 2	VASVININPNTTHSTGSCR	HexNAc(5)Hex(6)Fuc(1)	100.000	1.00.E-17
Q01638	Interleukin-1 receptor-like 1	SPTFNR	HexNAc(5)Hex(6)Fuc(1)	100.000	1.00.E-17
Q07954	Prolow-density lipoprotein receptor-related protein 1	LNLDGSNYTLLK	HexNAc(5)Hex(6)Fuc(1)	100.000	1.00.E-17
Q2KJY2	Kinesin-like protein KIF26B	PPNSTGVR	HexNAc(5)Hex(6)Fuc(1)	10.757	1.96.E-03
Q5IJ48	Protein crumbs homolog 2	NGSLAGGVR	HexNAc(5)Hex(6)Fuc(1)	100.000	1.00.E-17
Q9UBG0	C-type mannose receptor 2	VTPACNTSLPAQR	HexNAc(5)Hex(6)Fuc(1)	100.000	1.00.E-17
P26006	Isoform 2 of Integrin alpha-3	AHCVWLECPIPDAPVVTNVTVK	HexNAc(5)Hex(6)	6.890	1.33.E-02
Q8N3C0	Activating signal cointegrator 1 complex subunit 3	NATVR	HexNAc(5)Hex(6)	8.983	4.40.E-03
P08648	Integrin alpha-5	VTGLNCTTNHPINPK	HexNAc(5)Hex(5)NeuAc(2)	4.963	1.00.E-02
Q07954	Prolow-density lipoprotein receptor-related protein 1	FNSTEYQVVTR	HexNAc(5)Hex(5)NeuAc(1)	100.000	1.00.E-17
Q5VYK3	Proteasome-associated protein ECM29 homolog	SLMNNSK	HexNAc(5)Hex(5)NeuAc(1)	5.630	9.45.E-03
O43157	Plexin-B1	LTLNGSK	HexNAc(5)Hex(5)Fuc(3)	100.000	1.00.E-17
O43657	Tetraspanin-6	QYNSTGDYR	HexNAc(5)Hex(5)Fuc(3)	100.000	1.00.E-17
O75976	Carboxypeptidase D	DLDTDFTNNASQPETK	HexNAc(5)Hex(5)Fuc(3)	3.415	1.67.E-02
P05556	Isoform 5 of Integrin beta-1	NVTNR	HexNAc(5)Hex(5)Fuc(3)	100.000	1.00.E-17
P05556	Isoform 5 of Integrin beta-1	NPCTSEQNCTSPFSYK	HexNAc(5)Hex(5)Fuc(3)	100.000	1.00.E-17
P06756	Integrin alpha-V	NMTISR	HexNAc(5)Hex(5)Fuc(3)	3.154	2.73.E-02
P08648	Integrin alpha-5	VTGLNCTTNHPINPK	HexNAc(5)Hex(5)Fuc(3)	14.962	2.15.E-04
P08962	CD63 antigen	NNHTASILDR	HexNAc(5)Hex(5)Fuc(3)	100.000	1.00.E-17
P11717	Cation-independent mannose-6-phosphate receptor	GYPCGGNK	HexNAc(5)Hex(5)Fuc(3)	100.000	1.00.E-17
P13987	CD59 glycoprotein	TAVNCSSDFDACLITK	HexNAc(5)Hex(5)Fuc(3)	100.000	1.00.E-17
P19022	Cadherin-2	SNISILR	HexNAc(5)Hex(5)Fuc(3)	2.831	3.24.E-02
P20645	Cation-dependent mannose-6-phosphate receptor	EAGNHTSGAGLVQINK	HexNAc(5)Hex(5)Fuc(3)	40.639	1.01.E-05
P35613	Basigin	ILLTCSLNDSATEVTGHR	HexNAc(5)Hex(5)Fuc(3)	100.000	1.00.E-17
P98164	Low-density lipoprotein receptor-related protein 2	SFLDCTNR	HexNAc(5)Hex(5)Fuc(3)	100.000	1.00.E-17
P98172	Ephrin-B1	HHDYYITSTSNGSLEGLENR	HexNAc(5)Hex(5)Fuc(3)	100.000	1.00.E-17
Q07954	Prolow-density lipoprotein receptor-related protein 1	CNASSQFLCSSGR	HexNAc(5)Hex(5)Fuc(3)	100.000	1.00.E-17
Q13308	Isoform 6 of Inactive tyrosine-protein kinase 7	DGTPLSDGQSNHTVSSK	HexNAc(5)Hex(5)Fuc(3)	100.000	1.00.E-17
Q13433	Zinc transporter ZIP6	YGENNSLSVEGFR	HexNAc(5)Hex(5)Fuc(3)	100.000	1.00.E-17
Q13433	Zinc transporter ZIP6	YGENNSLSVEGFRK	HexNAc(5)Hex(5)Fuc(3)	5.460	1.46.E-02
Q5IJ48	Protein crumbs homolog 2	EGPPAAFSGHNASSGR	HexNAc(5)Hex(5)Fuc(3)	100.000	1.00.E-17
Q5ZPR3	CD276 antigen	QLVHSFAEGQDQGSAYANR	HexNAc(5)Hex(5)Fuc(3)	100.000	1.00.E-17

Q5ZPR3	CD276 antigen	VVLGANGTYSCLVR	HexNAc(5)Hex(5)Fuc(3)	7.063	5.47.E-03
Q92608	Dedicator of cytokinesis protein 2	TVNR	HexNAc(5)Hex(5)Fuc(3)	100.000	1.00.E-17
Q92823	Neuronal cell adhesion molecule	IPANK	HexNAc(5)Hex(5)Fuc(3)	100.000	1.00.E-17
Q9P273	Teneurin-3	IGPFANTTK	HexNAc(5)Hex(5)Fuc(3)	100.000	1.00.E-17
Q9UBG0	C-type mannose receptor 2	TSNISKPGTLER	HexNAc(5)Hex(5)Fuc(3)	100.000	1.00.E-17
Q9UBG0	C-type mannose receptor 2	VTPACNTSLPAQR	HexNAc(5)Hex(5)Fuc(3)	100.000	1.00.E-17
Q9UBG0	C-type mannose receptor 2	WNDSPCNQSLPSICK	HexNAc(5)Hex(5)Fuc(3)	8.554	3.99.E-03
Q9UHG0	Doublecortin domain-containing protein 2	INVSAR	HexNAc(5)Hex(5)Fuc(3)	100.000	1.00.E-17
P06756	Integrin alpha-V	NMTISR	HexNAc(5)Hex(5)Fuc(2)	3.679	3.54.E-02
P08648	Integrin alpha-5	VTGLNCTTNHPINPK	HexNAc(5)Hex(5)Fuc(2)	100.000	1.00.E-17
P08962	CD63 antigen	NNHTASILDR	HexNAc(5)Hex(5)Fuc(2)	100.000	1.00.E-17
P26006	Isoform 2 of Integrin alpha-3	NITIVTGAPR	HexNAc(5)Hex(5)Fuc(2)	100.000	1.00.E-17
Q07954	Prolow-density lipoprotein receptor-related protein 1	CNASSQFLCSSGR	HexNAc(5)Hex(5)Fuc(2)	100.000	1.00.E-17
Q0D2J5	Zinc finger protein 763	NLTSIGK	HexNAc(5)Hex(5)Fuc(2)	3.278	3.90.E-02
Q13433	Zinc transporter ZIP6	NTNENPQECFNASK	HexNAc(5)Hex(5)Fuc(2)	100.000	1.00.E-17
Q13740	CD166 antigen	NATVVWMK	HexNAc(5)Hex(5)Fuc(2)	100.000	1.00.E-17
Q9UBG0	C-type mannose receptor 2	VTPACNTSLPAQR	HexNAc(5)Hex(5)Fuc(2)	100.000	1.00.E-17
P05026	Sodium/potassium-transporting ATPase subunit beta-1	LEWLGNCSGLNDETYGYK	HexNAc(5)Hex(5)Fuc(1)NeuAc(2)	100.000	1.00.E-17
P08962	CD63 antigen	NNHTASILDR	HexNAc(5)Hex(5)Fuc(1)NeuAc(2)	6.682	5.48.E-03
Q8IV08	Phospholipase D3	DNHTHSDIQVK	HexNAc(5)Hex(5)Fuc(1)NeuAc(2)	100.000	1.00.E-17
O15031	Plexin-B2	NCSFQPER	HexNAc(5)Hex(5)Fuc(1)NeuAc(1)	100.000	1.00.E-17
O43657	Tetraspanin-6	QYNSTGDYR	HexNAc(5)Hex(5)Fuc(1)NeuAc(1)	100.000	1.00.E-17
O75976	Carboxypeptidase D	LLNTTDVYLLPSLNPDGFER	HexNAc(5)Hex(5)Fuc(1)NeuAc(1)	2.067	4.84.E-02
P05556	Isoform 5 of Integrin beta-1	NPCTSEQNCTSPFSYK	HexNAc(5)Hex(5)Fuc(1)NeuAc(1)	100.000	1.00.E-17
P06756	Integrin alpha-V	NMTISR	HexNAc(5)Hex(5)Fuc(1)NeuAc(1)	2.179	1.71.E-02
P08648	Integrin alpha-5	VTGLNCTTNHPINPK	HexNAc(5)Hex(5)Fuc(1)NeuAc(1)	100.000	1.00.E-17
P08962	CD63 antigen	NNHTASILDR	HexNAc(5)Hex(5)Fuc(1)NeuAc(1)	15.182	5.31.E-04
P13987	CD59 glycoprotein	TAVNCSSDFDACLITK	HexNAc(5)Hex(5)Fuc(1)NeuAc(1)	100.000	1.00.E-17
P14384	Carboxypeptidase M	TVAQNYSSVTHLHSIGK	HexNAc(5)Hex(5)Fuc(1)NeuAc(1)	100.000	1.00.E-17
P29317	Ephrin type-A receptor 2	TASVSINQTEPPK	HexNAc(5)Hex(5)Fuc(1)NeuAc(1)	100.000	1.00.E-17
P41217	Isoform 3 of OX-2 membrane glycoprotein	FSEDHLNITCSATAR	HexNAc(5)Hex(5)Fuc(1)NeuAc(1)	100.000	1.00.E-17
P43121	Cell surface glycoprotein MUC18	CGLSQSQGNLSHVDWFSVHK	HexNAc(5)Hex(5)Fuc(1)NeuAc(1)	5.224	1.31.E-02
P46100	Transcriptional regulator ATRX	RNLSSK	HexNAc(5)Hex(5)Fuc(1)NeuAc(1)	100.000	1.00.E-17
Q07954	Prolow-density lipoprotein receptor-related protein 1	MHLNGSNVQVLHR	HexNAc(5)Hex(5)Fuc(1)NeuAc(1)	2.949	3.46.E-02
Q13308	Isoform 6 of Inactive tyrosine-protein kinase 7	SANASFNIK	HexNAc(5)Hex(5)Fuc(1)NeuAc(1)	2.969	3.11.E-02
Q13433	Zinc transporter ZIP6	YGENNSLSVEGFRK	HexNAc(5)Hex(5)Fuc(1)NeuAc(1)	100.000	1.00.E-17
Q5ZPR3	CD276 antigen	QLVHSFAEGQDQGSAYANR	HexNAc(5)Hex(5)Fuc(1)NeuAc(1)	8.817	1.25.E-03
Q5ZPR3	CD276 antigen	VVLGANGTYSCLVR	HexNAc(5)Hex(5)Fuc(1)NeuAc(1)	3.378	4.21.E-02
Q9BX67	Junctional adhesion molecule C	IWNVTR	HexNAc(5)Hex(5)Fuc(1)NeuAc(1)	11.390	9.10.E-04
Q9UBG0	C-type mannose receptor 2	WNDSPCNQSLPSICK	HexNAc(5)Hex(5)Fuc(1)NeuAc(1)	100.000	1.00.E-17
Q9UHN6	Cell surface hyaluronidase	FDTHEYRNESR	HexNAc(5)Hex(5)Fuc(1)NeuAc(1)	100.000	1.00.E-17
O15031	Plexin-B2	SCVAVTSAQPQNMSR	HexNAc(5)Hex(5)Fuc(1)	100.000	1.00.E-17
O75882	Attractin	CINQSICEK	HexNAc(5)Hex(5)Fuc(1)	100.000	1.00.E-17
O75976	Carboxypeptidase D	DLDTDFTNNASQPETK	HexNAc(5)Hex(5)Fuc(1)	13.354	1.66.E-03
O75976	Carboxypeptidase D	NVTVK	HexNAc(5)Hex(5)Fuc(1)	5.405	7.42.E-03
P01892	HLA class I histocompatibility antigen, A-2 alpha chain	GYYNQSEAGSHTVQR	HexNAc(5)Hex(5)Fuc(1)	4.146	1.90.E-02
P04216	Thy-1 membrane glycoprotein	TNFTSK	HexNAc(5)Hex(5)Fuc(1)	100.000	1.00.E-17
P05556	Isoform 5 of Integrin beta-1	NPCTSEQNCTSPFSYK	HexNAc(5)Hex(5)Fuc(1)	100.000	1.00.E-17
P05556	Isoform 5 of Integrin beta-1	LRNPCTSEQNCTSPFSYK	HexNAc(5)Hex(5)Fuc(1)	23.313	1.63.E-04
P05556	Isoform 5 of Integrin beta-1	NVTNR	HexNAc(5)Hex(5)Fuc(1)	9.617	4.00.E-03
P06756	Integrin alpha-V	NMTISR	HexNAc(5)Hex(5)Fuc(1)	100.000	1.00.E-17
P08648	Integrin alpha-5	VTGLNCTTNHPINPK	HexNAc(5)Hex(5)Fuc(1)	100.000	1.00.E-17
P08962	CD63 antigen	NNHTASILDR	HexNAc(5)Hex(5)Fuc(1)	23.488	3.42.E-05
P13473	Isoform LAMP-2C of Lysosome-associated membrane glycoprotein 2	LNSSTIK	HexNAc(5)Hex(5)Fuc(1)	100.000	1.00.E-17
P14384	Carboxypeptidase M	SQNFSALK	HexNAc(5)Hex(5)Fuc(1)	4.630	3.36.E-02
P19022	Cadherin-2	SNISILR	HexNAc(5)Hex(5)Fuc(1)	19.457	1.36.E-04
P46100	Transcriptional regulator ATRX	RNLSSK	HexNAc(5)Hex(5)Fuc(1)	63.781	2.24.E-07
P51654	Isoform 3 of Glypican-3	NYTNAMFK	HexNAc(5)Hex(5)Fuc(1)	100.000	1.00.E-17
P54709	Sodium/potassium-transporting ATPase subunit beta-3	NLTVCPDGALFEQK	HexNAc(5)Hex(5)Fuc(1)	3.683	3.18.E-02
P55285	Cadherin-6	EDAQINTTIGSVTAQDPDAAR	HexNAc(5)Hex(5)Fuc(1)	100.000	1.00.E-17
P55285	Cadherin-6	IFNIDSGNGSIFTSK	HexNAc(5)Hex(5)Fuc(1)	4.681	2.13.E-02
P56199	Integrin alpha-1	QTQVGIVQYGENVTHEFNLNK	HexNAc(5)Hex(5)Fuc(1)	100.000	1.00.E-17
P78504	Protein jagged-1	DDFFGHYACDQNGNK	HexNAc(5)Hex(5)Fuc(1)	100.000	1.00.E-17
P78504	Protein jagged-1	NCSHLK	HexNAc(5)Hex(5)Fuc(1)	4.768	1.77.E-02
Q01638	Interleukin-1 receptor-like 1	SPTFNR	HexNAc(5)Hex(5)Fuc(1)	65.411	5.43.E-08
Q02487	Desmocollin-2	ANYTILK	HexNAc(5)Hex(5)Fuc(1)	6.459	5.46.E-03
Q02880	Isoform Beta-1 of DNA topoisomerase 2-beta	ITIENR	HexNAc(5)Hex(5)Fuc(1)	100.000	1.00.E-17
Q07954	Prolow-density lipoprotein receptor-related protein 1	IETILLNGTDRK	HexNAc(5)Hex(5)Fuc(1)	100.000	1.00.E-17
Q07954	Prolow-density lipoprotein receptor-related protein 1	CNASSQFLCSSGR	HexNAc(5)Hex(5)Fuc(1)	13.865	1.56.E-03
Q07954	Prolow-density lipoprotein receptor-related protein 1	LTSCATNASICGDEAR	HexNAc(5)Hex(5)Fuc(1)	9.124	1.99.E-03
Q07954	Prolow-density lipoprotein receptor-related protein 1	TCVSNCTASQFVCK	HexNAc(5)Hex(5)Fuc(1)	6.417	1.57.E-02
Q07954	Prolow-density lipoprotein receptor-related protein 1	GVTHLNISGLK	HexNAc(5)Hex(5)Fuc(1)	6.241	1.07.E-02
Q07954	Prolow-density lipoprotein receptor-related protein 1	AVNSSCR	HexNAc(5)Hex(5)Fuc(1)	4.961	5.17.E-03
Q07954	Prolow-density lipoprotein receptor-related protein 1	DNATDSVPLR	HexNAc(5)Hex(5)Fuc(1)	4.043	1.27.E-02
Q07954	Prolow-density lipoprotein receptor-related protein 1	THANGSIK	HexNAc(5)Hex(5)Fuc(1)	3.346	4.23.E-02
Q07954	Prolow-density lipoprotein receptor-related protein 1	FGTCSQLCNNTK	HexNAc(5)Hex(5)Fuc(1)	3.082	1.83.E-02
Q07954	Prolow-density lipoprotein receptor-related protein 1	VDIPQQPMGIIAVANDTNSCELSPCR	HexNAc(5)Hex(5)Fuc(1)	2.391	4.69.E-02
Q0D2J5	Zinc finger protein 763	NLTSIGK	HexNAc(5)Hex(5)Fuc(1)	6.786	3.68.E-03
Q13308	Isoform 6 of Inactive tyrosine-protein kinase 7	RQDVNITVATVPSWLK	HexNAc(5)Hex(5)Fuc(1)	100.000	1.00.E-17
Q13308	Isoform 6 of Inactive tyrosine-protein kinase 7	DDAGNYTCIASNGPQGQIR	HexNAc(5)Hex(5)Fuc(1)	100.000	1.00.E-17
Q13308	Isoform 6 of Inactive tyrosine-protein kinase 7	DGTPLSDGQSNHTVSSK	HexNAc(5)Hex(5)Fuc(1)	25.807	7.54.E-04

Q13308	Isoform 6 of Inactive tyrosine-protein kinase 7	SANASFNIK	HexNAc(5)Hex(5)Fuc(1)	8.883	2.61.E-03
Q13433	Zinc transporter ZIP6	NTNENPQECFNASK	HexNAc(5)Hex(5)Fuc(1)	10.005	1.20.E-02
Q13740	CD166 antigen	NAIKEGDNITLK	HexNAc(5)Hex(5)Fuc(1)	100.000	1.00.E-17
Q13740	CD166 antigen	NATVVWMK	HexNAc(5)Hex(5)Fuc(1)	5.754	3.31.E-03
Q13740	CD166 antigen	RNATGDYK	HexNAc(5)Hex(5)Fuc(1)	3.072	4.98.E-02
Q13740	CD166 antigen	LGDCISEDSYPDGNITWYR	HexNAc(5)Hex(5)Fuc(1)	2.580	4.90.E-02
Q16851	UTP--glucose-1-phosphate uridylyltransferase	NVSLK	HexNAc(5)Hex(5)Fuc(1)	100.000	1.00.E-17
Q5IJ48	Protein crumbs homolog 2	NGSLAGGVR	HexNAc(5)Hex(5)Fuc(1)	100.000	1.00.E-17
Q5IJ48	Protein crumbs homolog 2	EGPPAAFSGHNASSGR	HexNAc(5)Hex(5)Fuc(1)	3.604	2.31.E-02
Q5ZPR3	CD276 antigen	VVLGANGTYSCLVR	HexNAc(5)Hex(5)Fuc(1)	6.727	2.48.E-02
Q6AWC8	Putative uncharacterized protein LOC100129027	NTSLSK	HexNAc(5)Hex(5)Fuc(1)	100.000	1.00.E-17
Q7Z417	Nuclear fragile X mental retardation-interacting protein 2	SGENQSVDK	HexNAc(5)Hex(5)Fuc(1)	5.951	3.26.E-03
Q8TEY7	Ubiquitin carboxyl-terminal hydrolase 33	IPSNTTLK	HexNAc(5)Hex(5)Fuc(1)	6.550	1.29.E-02
Q92608	Dedicator of cytokinesis protein 2	TVNR	HexNAc(5)Hex(5)Fuc(1)	12.661	7.95.E-04
Q92673	Sortilin-related receptor	AASNFTEIK	HexNAc(5)Hex(5)Fuc(1)	100.000	1.00.E-17
Q96AC1	Isoform 3 of Fermitin family homolog 2	VNFSDR	HexNAc(5)Hex(5)Fuc(1)	100.000	1.00.E-17
Q99523	Sortilin	DITDLINNTFIR	HexNAc(5)Hex(5)Fuc(1)	3.364	4.25.E-02
Q9HAR2	Isoform 4 of Adhesion G protein-coupled receptor L3	NLTPGGK	HexNAc(5)Hex(5)Fuc(1)	100.000	1.00.E-17
Q9HCU4	Cadherin EGF LAG seven-pass G-type receptor 2	LQRNESGLDSGR	HexNAc(5)Hex(5)Fuc(1)	100.000	1.00.E-17
Q9HD45	Transmembrane 9 superfamily member 3	IVDVNLTSEGK	HexNAc(5)Hex(5)Fuc(1)	100.000	1.00.E-17
Q9P273	Teneurin-3	IGPFANTTK	HexNAc(5)Hex(5)Fuc(1)	100.000	1.00.E-17
Q9P2B2	Prostaglandin F2 receptor negative regulator	NVSVAEGK	HexNAc(5)Hex(5)Fuc(1)	100.000	1.00.E-17
Q9UBG0	C-type mannose receptor 2	NCTSFR	HexNAc(5)Hex(5)Fuc(1)	100.000	1.00.E-17
Q9UBG0	C-type mannose receptor 2	TSNISKPGTLER	HexNAc(5)Hex(5)Fuc(1)	100.000	1.00.E-17
Q9UBG0	C-type mannose receptor 2	VTPACNTSLPAQR	HexNAc(5)Hex(5)Fuc(1)	5.032	8.15.E-03
Q9UHN6	Cell surface hyaluronidase	QNGSLSK	HexNAc(5)Hex(5)Fuc(1)	3.806	1.88.E-02
A4D0S4	Laminin subunit beta-4	RQNDSLDK	HexNAc(5)Hex(5)	4.565	1.69.E-02
O15031	Plexin-B2	SCVAVTSAQPQNMSR	HexNAc(5)Hex(5)	100.000	1.00.E-17
P05556	Isoform 5 of Integrin beta-1	NVTR	HexNAc(5)Hex(5)	10.441	1.73.E-03
P26006	Isoform 2 of Integrin alpha-3	NITIVTGAPR	HexNAc(5)Hex(5)	100.000	1.00.E-17
P30419	Glycylpeptide N-tetradecanoyltransferase 1	NMTMQR	HexNAc(5)Hex(5)	100.000	1.00.E-17
P42892	Endothelin-converting enzyme 1	ACMNETR	HexNAc(5)Hex(5)	2.629	3.46.E-02
P51805	Plexin-A3	SLNR	HexNAc(5)Hex(5)	6.704	2.47.E-03
P78310	Coxsackievirus and adenovirus receptor	SGDASINVTNLQLSDIGTYQCK	HexNAc(5)Hex(5)	4.520	6.91.E-03
P78504	Protein jagged-1	NCSHLK	HexNAc(5)Hex(5)	3.487	4.54.E-02
Q05209	Tyrosine-protein phosphatase non-receptor type 12	NYSK	HexNAc(5)Hex(5)	4.642	2.30.E-02
Q07954	Prolow-density lipoprotein receptor-related protein 1	VDIPQQPMGIIAVANDTNSCELSPCR	HexNAc(5)Hex(5)	28.610	7.48.E-06
Q9P273	Teneurin-3	FNISLQK	HexNAc(5)Hex(5)	100.000	1.00.E-17
Q9UBV2	Protein sel-1 homolog 1	ILNGSNK	HexNAc(5)Hex(5)	100.000	1.00.E-17
O94874	E3 UFM1-protein ligase 1	PINK	HexNAc(5)Hex(4)NeuAc(2)	100.000	1.00.E-17
Q12797	Aspartyl/asparaginyl beta-hydroxylase	CANETK	HexNAc(5)Hex(4)NeuAc(2)	100.000	1.00.E-17
P05026	Sodium/potassium-transporting ATPase subunit beta-1	LEWLGNCSGLNDETYGYK	HexNAc(5)Hex(4)NeuAc(1)	3.603	4.10.E-02
Q6ZWB6	BTB/POZ domain-containing protein KCTD8	ETNLSK	HexNAc(5)Hex(4)NeuAc(1)	2.279	3.30.E-02
Q9HD45	Transmembrane 9 superfamily member 3	IVDVNLTSEGK	HexNAc(5)Hex(4)NeuAc(1)	100.000	1.00.E-17
Q9Y6N7	Isoform 2 of Roundabout homolog 1	YHINK	HexNAc(5)Hex(4)NeuAc(1)	6.631	1.22.E-02
Q96MM7	Isoform 4 of Heparan-sulfate 6-O-sulfotransferase 2	YNFTR	HexNAc(5)Hex(4)Fuc(2)NeuAc(1)	100.000	1.00.E-17
A1L157	Tetraspanin-11	QHLNR	HexNAc(5)Hex(4)Fuc(2)	100.000	1.00.E-17
A4D0S4	Laminin subunit beta-4	RQNDSLDK	HexNAc(5)Hex(4)Fuc(2)	100.000	1.00.E-17
O14786	Neuropilin-1	IGYSNNGSDWK	HexNAc(5)Hex(4)Fuc(2)	100.000	1.00.E-17
O15031	Plexin-B2	ALSNISLR	HexNAc(5)Hex(4)Fuc(2)	14.918	1.51.E-03
O43157	Plexin-B1	LTLNGSK	HexNAc(5)Hex(4)Fuc(2)	100.000	1.00.E-17
O43157	Plexin-B1	YTLDPNITSAGPTK	HexNAc(5)Hex(4)Fuc(2)	2.975	2.97.E-02
O43303	Centriolar coiled-coil protein of 110 kDa	NTSEVK	HexNAc(5)Hex(4)Fuc(2)	3.314	1.66.E-02
O75882	Attractin	NHSCSEGQISIFR	HexNAc(5)Hex(4)Fuc(2)	100.000	1.00.E-17
O75976	Carboxypeptidase D	DLDTDFTNNASQPETK	HexNAc(5)Hex(4)Fuc(2)	3.501	1.28.E-02
P05026	Sodium/potassium-transporting ATPase subunit beta-1	LEWLGNCSGLNDETYGYK	HexNAc(5)Hex(4)Fuc(2)	3.603	4.10.E-02
P05556	Isoform 5 of Integrin beta-1	KDTCTQECSYFNITK	HexNAc(5)Hex(4)Fuc(2)	100.000	1.00.E-17
P08648	Integrin alpha-5	VTGLNCTTNHPINPK	HexNAc(5)Hex(4)Fuc(2)	100.000	1.00.E-17
P08962	CD63 antigen	NNHTASILDR	HexNAc(5)Hex(4)Fuc(2)	100.000	1.00.E-17
P11717	Cation-independent mannose-6-phosphate receptor	GYPCGGNK	HexNAc(5)Hex(4)Fuc(2)	100.000	1.00.E-17
P19022	Cadherin-2	SNISILR	HexNAc(5)Hex(4)Fuc(2)	6.514	4.37.E-03
P32004	Neural cell adhesion molecule L1	VPGNQTSTTLK	HexNAc(5)Hex(4)Fuc(2)	100.000	1.00.E-17
P51654	Isoform 3 of Glypican-3	NYTNAMFK	HexNAc(5)Hex(4)Fuc(2)	4.610	4.25.E-03
P52797	Ephrin-A3	TCNASQGFK	HexNAc(5)Hex(4)Fuc(2)	100.000	1.00.E-17
P54709	Sodium/potassium-transporting ATPase subunit beta-3	NLTVCPDGALFEQK	HexNAc(5)Hex(4)Fuc(2)	5.817	1.17.E-02
Q07954	Prolow-density lipoprotein receptor-related protein 1	WTGHNVTVVQR	HexNAc(5)Hex(4)Fuc(2)	100.000	1.00.E-17
Q07954	Prolow-density lipoprotein receptor-related protein 1	DNATDSVPLR	HexNAc(5)Hex(4)Fuc(2)	100.000	1.00.E-17
Q07954	Prolow-density lipoprotein receptor-related protein 1	CNASSQFLCSSGR	HexNAc(5)Hex(4)Fuc(2)	100.000	1.00.E-17
Q07954	Prolow-density lipoprotein receptor-related protein 1	IETILLNGTDRK	HexNAc(5)Hex(4)Fuc(2)	100.000	1.00.E-17
Q07954	Prolow-density lipoprotein receptor-related protein 1	THANGSIK	HexNAc(5)Hex(4)Fuc(2)	3.506	4.55.E-02
Q13308	Isoform 6 of Inactive tyrosine-protein kinase 7	SANASFNIK	HexNAc(5)Hex(4)Fuc(2)	100.000	1.00.E-17
Q13433	Zinc transporter ZIP6	NTNENPQECFNASK	HexNAc(5)Hex(4)Fuc(2)	100.000	1.00.E-17
Q16851	UTP--glucose-1-phosphate uridylyltransferase	NVSLK	HexNAc(5)Hex(4)Fuc(2)	100.000	1.00.E-17
Q5ZPR3	CD276 antigen	VVLGANGTYSCLVR	HexNAc(5)Hex(4)Fuc(2)	100.000	1.00.E-17
Q96MM7	Isoform 4 of Heparan-sulfate 6-O-sulfotransferase 2	YNFTR	HexNAc(5)Hex(4)Fuc(2)	100.000	1.00.E-17
Q99523	Sortilin	DITDLINNTFIR	HexNAc(5)Hex(4)Fuc(2)	2.328	4.72.E-02
Q9BX67	Junctional adhesion molecule C	IWNVTR	HexNAc(5)Hex(4)Fuc(2)	100.000	1.00.E-17
Q9HCK4	Isoform 3 of Roundabout homolog 2	FHINK	HexNAc(5)Hex(4)Fuc(2)	100.000	1.00.E-17
Q9HCU4	Cadherin EGF LAG seven-pass G-type receptor 2	LQRNESGLDSGR	HexNAc(5)Hex(4)Fuc(2)	100.000	1.00.E-17
Q9NT68	Teneurin-2	LGPYANTTK	HexNAc(5)Hex(4)Fuc(2)	100.000	1.00.E-17
Q9NT99	Leucine-rich repeat-containing protein 4B	ETVPSNTTCCAR	HexNAc(5)Hex(4)Fuc(2)	100.000	1.00.E-17

Q9UBG0	C-type mannose receptor 2	NCTSFR	HexNAc(5)Hex(4)Fuc(2)	4.819	1.72.E-02
Q9UHN6	Cell surface hyaluronidase	QNGSLSK	HexNAc(5)Hex(4)Fuc(2)	100.000	1.00.E-17
Q9UPZ6	Thrombospondin type-1 domain-containing protein 7A	EVMCINK	HexNAc(5)Hex(4)Fuc(2)	100.000	1.00.E-17
Q9Y639	Neuroplastin	NASNMEYR	HexNAc(5)Hex(4)Fuc(2)	100.000	1.00.E-17
P32780	General transcription factor IIH subunit 1	NNSVK	HexNAc(5)Hex(4)Fuc(1)NeuAc(2)	13.489	4.50.E-04
Q8NBJ4	Golgi membrane protein 1	AVLVNNITTGER	HexNAc(5)Hex(4)Fuc(1)NeuAc(2)	100.000	1.00.E-17
O43852	Isoform 3 of Calumenin	NATYGYVLDDPDPDDGFNYK	HexNAc(5)Hex(4)Fuc(1)NeuAc(1)	3.888	1.03.E-02
O94813	Slit homolog 2 protein	ITCVGNDSFIGLSSVR	HexNAc(5)Hex(4)Fuc(1)NeuAc(1)	100.000	1.00.E-17
P51654	Isoform 3 of Glypican-3	NYTNAMFK	HexNAc(5)Hex(4)Fuc(1)NeuAc(1)	3.145	1.81.E-02
P78504	Protein jagged-1	NCSHLK	HexNAc(5)Hex(4)Fuc(1)NeuAc(1)	100.000	1.00.E-17
Q0D2J5	Zinc finger protein 763	NLTSIGK	HexNAc(5)Hex(4)Fuc(1)NeuAc(1)	100.000	1.00.E-17
Q5IJ48	Protein crumbs homolog 2	NGSLAGGVR	HexNAc(5)Hex(4)Fuc(1)NeuAc(1)	100.000	1.00.E-17
Q8IV08	Phospholipase D3	DNHTHSDIQVK	HexNAc(5)Hex(4)Fuc(1)NeuAc(1)	10.870	2.14.E-03
Q8NFC6	Biorientation of chromosomes in cell division protein 1-like 1	NESK	HexNAc(5)Hex(4)Fuc(1)NeuAc(1)	100.000	1.00.E-17
Q9UBG0	C-type mannose receptor 2	WNDSPCNQSLPSICK	HexNAc(5)Hex(4)Fuc(1)NeuAc(1)	100.000	1.00.E-17
A4D0S4	Laminin subunit beta-4	RQNDSLDK	HexNAc(5)Hex(4)Fuc(1)	100.000	1.00.E-17
A8K979	ERI1 exoribonuclease 2	NLSISTK	HexNAc(5)Hex(4)Fuc(1)	100.000	1.00.E-17
O15031	Plexin-B2	SCVAVTSAQPQNMSR	HexNAc(5)Hex(4)Fuc(1)	100.000	1.00.E-17
O15031	Plexin-B2	EAESLQPMTVVGTDYVFHNDTK	HexNAc(5)Hex(4)Fuc(1)	100.000	1.00.E-17
O15031	Plexin-B2	ALSNISLR	HexNAc(5)Hex(4)Fuc(1)	7.341	3.75.E-03
O43157	Plexin-B1	LTLNGSK	HexNAc(5)Hex(4)Fuc(1)	100.000	1.00.E-17
O43657	Tetraspanin-6	QYNSTGDYR	HexNAc(5)Hex(4)Fuc(1)	100.000	1.00.E-17
O75976	Carboxypeptidase D	DLDTDFTNNASQPETK	HexNAc(5)Hex(4)Fuc(1)	9.137	1.48.E-03
O75976	Carboxypeptidase D	NVTVK	HexNAc(5)Hex(4)Fuc(1)	4.384	8.03.E-03
P05556	Isoform 5 of Integrin beta-1	NPCTSEQNCTSPFSYK	HexNAc(5)Hex(4)Fuc(1)	100.000	1.00.E-17
P05556	Isoform 5 of Integrin beta-1	LRNPCTSEQNCTSPFSYK	HexNAc(5)Hex(4)Fuc(1)	4.251	2.47.E-02
P06756	Integrin alpha-V	NMTISR	HexNAc(5)Hex(4)Fuc(1)	40.028	3.96.E-06
P06756	Integrin alpha-V	ENQNHSYSLK	HexNAc(5)Hex(4)Fuc(1)	5.303	1.21.E-02
P06756	Integrin alpha-V	ANTTQPGIVEGGQVLK	HexNAc(5)Hex(4)Fuc(1)	5.025	1.90.E-03
P08648	Integrin alpha-5	VTGLNCTTNHPINPK	HexNAc(5)Hex(4)Fuc(1)	9.944	1.25.E-03
P08962	CD63 antigen	NNHTASILDR	HexNAc(5)Hex(4)Fuc(1)	5.394	1.06.E-02
P11279	Lysosome-associated membrane glycoprotein 1	SSCGKENTSDPSLVIAFGR	HexNAc(5)Hex(4)Fuc(1)	6.660	4.59.E-03
P13987	CD59 glycoprotein	TAVNCSSDFDACLITK	HexNAc(5)Hex(4)Fuc(1)	100.000	1.00.E-17
P14384	Carboxypeptidase M	SQNFSALK	HexNAc(5)Hex(4)Fuc(1)	100.000	1.00.E-17
P19022	Cadherin-2	SNISILR	HexNAc(5)Hex(4)Fuc(1)	100.000	1.00.E-17
P46100	Transcriptional regulator ATRX	RNLSSK	HexNAc(5)Hex(4)Fuc(1)	100.000	1.00.E-17
P51654	Isoform 3 of Glypican-3	NYTNAMFK	HexNAc(5)Hex(4)Fuc(1)	57.608	1.83.E-06
P51805	Plexin-A3	SLNR	HexNAc(5)Hex(4)Fuc(1)	100.000	1.00.E-17
P55285	Cadherin-6	IFNIDSGNGSIFTSK	HexNAc(5)Hex(4)Fuc(1)	4.956	3.75.E-03
P55285	Cadherin-6	EDAQINTTIGSVTAQDPDAAR	HexNAc(5)Hex(4)Fuc(1)	2.173	3.58.E-02
P56199	Integrin alpha-1	VYVYALNQTR	HexNAc(5)Hex(4)Fuc(1)	100.000	1.00.E-17
P78310	Coxsackievirus and adenovirus receptor	NASSEYSGTYSCTVR	HexNAc(5)Hex(4)Fuc(1)	4.781	1.22.E-02
P78504	Protein jagged-1	DDFFGHYACDQNGNK	HexNAc(5)Hex(4)Fuc(1)	100.000	1.00.E-17
P78504	Protein jagged-1	NCSHLK	HexNAc(5)Hex(4)Fuc(1)	19.703	1.22.E-04
P98164	Low-density lipoprotein receptor-related protein 2	VGMDGTNK	HexNAc(5)Hex(4)Fuc(1)	100.000	1.00.E-17
P98164	Low-density lipoprotein receptor-related protein 2	EFICNGVDNCHDNNTSDEK	HexNAc(5)Hex(4)Fuc(1)	100.000	1.00.E-17
P98164	Low-density lipoprotein receptor-related protein 2	NLTNPR	HexNAc(5)Hex(4)Fuc(1)	11.693	5.94.E-04
Q01638	Interleukin-1 receptor-like 1	SPTFNR	HexNAc(5)Hex(4)Fuc(1)	100.000	1.00.E-17
Q02487	Desmocollin-2	ANYTILK	HexNAc(5)Hex(4)Fuc(1)	7.316	2.15.E-03
Q07954	Prolow-density lipoprotein receptor-related protein 1	DNATDSVPLR	HexNAc(5)Hex(4)Fuc(1)	100.000	1.00.E-17
Q07954	Prolow-density lipoprotein receptor-related protein 1	LTSCATNASICGDEAR	HexNAc(5)Hex(4)Fuc(1)	11.170	5.20.E-04
Q07954	Prolow-density lipoprotein receptor-related protein 1	ELQGNCSR	HexNAc(5)Hex(4)Fuc(1)	10.744	1.51.E-03
Q07954	Prolow-density lipoprotein receptor-related protein 1	AVNSSCR	HexNAc(5)Hex(4)Fuc(1)	10.154	1.21.E-02
Q07954	Prolow-density lipoprotein receptor-related protein 1	CNASSQFLCSSGR	HexNAc(5)Hex(4)Fuc(1)	8.970	6.87.E-03
Q07954	Prolow-density lipoprotein receptor-related protein 1	VNRFNSTEYQVVTR	HexNAc(5)Hex(4)Fuc(1)	8.350	3.84.E-03
Q07954	Prolow-density lipoprotein receptor-related protein 1	GVTHLNISGLK	HexNAc(5)Hex(4)Fuc(1)	7.547	9.07.E-04
Q07954	Prolow-density lipoprotein receptor-related protein 1	TCVSNCTASQFVCK	HexNAc(5)Hex(4)Fuc(1)	5.948	6.06.E-03
Q07954	Prolow-density lipoprotein receptor-related protein 1	FGTCSQLCNNTK	HexNAc(5)Hex(4)Fuc(1)	3.751	1.67.E-02
Q07954	Prolow-density lipoprotein receptor-related protein 1	IETILLNGTDRK	HexNAc(5)Hex(4)Fuc(1)	2.721	2.69.E-02
Q08722	Leukocyte surface antigen CD47	SDAVSHTGNYTCEVTELTR	HexNAc(5)Hex(4)Fuc(1)	11.727	1.62.E-03
Q12860	Contactin-1	ANSTGTLVITDPTR	HexNAc(5)Hex(4)Fuc(1)	100.000	1.00.E-17
Q13308	Isoform 6 of Inactive tyrosine-protein kinase 7	SANASFNIK	HexNAc(5)Hex(4)Fuc(1)	100.000	1.00.E-17
Q13308	Isoform 6 of Inactive tyrosine-protein kinase 7	DGTPLSDGQSNHTVSSK	HexNAc(5)Hex(4)Fuc(1)	100.000	1.00.E-17
Q13349	Integrin alpha-D	VNNLSQR	HexNAc(5)Hex(4)Fuc(1)	100.000	1.00.E-17
Q13740	CD166 antigen	NATVVWMK	HexNAc(5)Hex(4)Fuc(1)	100.000	1.00.E-17
Q13740	CD166 antigen	LGDCISEDSYPDGNITWYR	HexNAc(5)Hex(4)Fuc(1)	4.432	1.81.E-02
Q16851	UTP--glucose-1-phosphate uridylyltransferase	NVSLK	HexNAc(5)Hex(4)Fuc(1)	6.456	8.24.E-03
Q2KJY2	Kinesin-like protein KIF26B	PPNSTGVR	HexNAc(5)Hex(4)Fuc(1)	7.230	6.58.E-03
Q5IJ48	Protein crumbs homolog 2	NGSLAGGVR	HexNAc(5)Hex(4)Fuc(1)	100.000	1.00.E-17
Q5ZPR3	CD276 antigen	QLVHSFAEGQDQGSAYANR	HexNAc(5)Hex(4)Fuc(1)	100.000	1.00.E-17
Q6EMK4	Vasorin	LHEITNETFR	HexNAc(5)Hex(4)Fuc(1)	3.817	2.65.E-02
Q6UXK5	Leucine-rich repeat neuronal protein 1	SCVNVTTK	HexNAc(5)Hex(4)Fuc(1)	3.462	3.90.E-02
Q7Z388	Probable C-mannosyltransferase DPY19L4	VYWNR	HexNAc(5)Hex(4)Fuc(1)	100.000	1.00.E-17
Q8N0W4	Isoform 2 of Neuroligin-4, X-linked	NTTQFAAVCPQHLDER	HexNAc(5)Hex(4)Fuc(1)	100.000	1.00.E-17
Q8N7P1	Inactive phospholipase D5	LFCPKNR	HexNAc(5)Hex(4)Fuc(1)	6.361	5.62.E-03
Q92608	Dedicator of cytokinesis protein 2	TVNR	HexNAc(5)Hex(4)Fuc(1)	10.566	1.25.E-03
Q92673	Sortilin-related receptor	AASNFTEIK	HexNAc(5)Hex(4)Fuc(1)	100.000	1.00.E-17
Q99523	Sortilin	DITDLINNTFIR	HexNAc(5)Hex(4)Fuc(1)	100.000	1.00.E-17
Q9C019	Tripartite motif-containing protein 15	VNQSR	HexNAc(5)Hex(4)Fuc(1)	9.416	2.31.E-03
Q9HAR2	Isoform 4 of Adhesion G protein-coupled receptor L3	NLTPGGK	HexNAc(5)Hex(4)Fuc(1)	100.000	1.00.E-17
Q9HCU4	Cadherin EGF LAG seven-pass G-type receptor 2	LQRNESGLDSGR	HexNAc(5)Hex(4)Fuc(1)	5.996	6.33.E-03

Q9P2B2	Prostaglandin F2 receptor negative regulator	NVSVAEGK	HexNAc(5)Hex(4)Fuc(1)	100.000	1.00.E-17
Q9UBG0	C-type mannose receptor 2	NCTSFR	HexNAc(5)Hex(4)Fuc(1)	100.000	1.00.E-17
Q9UBG0	C-type mannose receptor 2	TSNISKPGTLER	HexNAc(5)Hex(4)Fuc(1)	100.000	1.00.E-17
Q9UBG0	C-type mannose receptor 2	WNDSPCNQSLPSICK	HexNAc(5)Hex(4)Fuc(1)	5.157	1.26.E-02
Q9UHN6	Cell surface hyaluronidase	QNGSLSK	HexNAc(5)Hex(4)Fuc(1)	100.000	1.00.E-17
Q9UHN6	Cell surface hyaluronidase	HPSCVNVSK	HexNAc(5)Hex(4)Fuc(1)	100.000	1.00.E-17
A1L157	Tetraspanin-11	QHLNR	HexNAc(5)Hex(4)	100.000	1.00.E-17
A4D0S4	Laminin subunit beta-4	RQNDSLDK	HexNAc(5)Hex(4)	100.000	1.00.E-17
O15031	Plexin-B2	SCVAVTSAQPQNMSR	HexNAc(5)Hex(4)	6.806	8.87.E-03
O75503	Ceroid-lipofuscinosis neuronal protein 5	NIETNYTR	HexNAc(5)Hex(4)	100.000	1.00.E-17
P07996	Thrombospondin-1	VVNSTTGPGEHLR	HexNAc(5)Hex(4)	100.000	1.00.E-17
P13473	Isoform LAMP-2C of Lysosome-associated membrane glycoprotein 2	LNSSTIK	HexNAc(5)Hex(4)	100.000	1.00.E-17
P15151	Poliovirus receptor	NVTCK	HexNAc(5)Hex(4)	6.341	3.51.E-03
P49750	Isoform 4 of YLP motif-containing protein 1	ALNR	HexNAc(5)Hex(4)	3.722	2.70.E-02
P51805	Plexin-A3	SLNR	HexNAc(5)Hex(4)	10.719	1.51.E-03
P78310	Coxsackievirus and adenovirus receptor	SGDASINVTNLQLSDIGTYQCK	HexNAc(5)Hex(4)	8.319	1.53.E-03
P78504	Protein jagged-1	NCSHLK	HexNAc(5)Hex(4)	6.568	7.44.E-03
Q07954	Prolow-density lipoprotein receptor-related protein 1	INNGGCQDLCLLTHQGHVNCSCR	HexNAc(5)Hex(4)	100.000	1.00.E-17
Q07954	Prolow-density lipoprotein receptor-related protein 1	FNSTEYQVVTR	HexNAc(5)Hex(4)	4.225	2.86.E-03
Q9H0X4	Protein FAM234A	YKPDTLAVAVENGTGTDR	HexNAc(5)Hex(4)	3.293	2.31.E-02
Q9HCK4	Isoform 3 of Roundabout homolog 2	FHINK	HexNAc(5)Hex(4)	100.000	1.00.E-17
Q9HD45	Transmembrane 9 superfamily member 3	IVDVNLTSEGK	HexNAc(5)Hex(4)	100.000	1.00.E-17
O75496	Geminin	NSSVPR	HexNAc(5)Hex(3)Fuc(1)NeuAc(1)	3.243	3.45.E-02
A4D0S4	Laminin subunit beta-4	RQNDSLDK	HexNAc(5)Hex(3)Fuc(1)	100.000	1.00.E-17
O14917	Protocadherin-17	DSGAPAHLESNATVR	HexNAc(5)Hex(3)Fuc(1)	100.000	1.00.E-17
O15031	Plexin-B2	ALSNISLR	HexNAc(5)Hex(3)Fuc(1)	100.000	1.00.E-17
O15031	Plexin-B2	TEAGAFEYVPDPTFENFTGGVK	HexNAc(5)Hex(3)Fuc(1)	2.666	3.08.E-02
O43157	Plexin-B1	LTLNGSK	HexNAc(5)Hex(3)Fuc(1)	100.000	1.00.E-17
O43657	Tetraspanin-6	QYNSTGDYR	HexNAc(5)Hex(3)Fuc(1)	2.918	4.00.E-02
O43852	Isoform 4 of Calumenin	NVTYGTYLDDPDPDDGFNYK	HexNAc(5)Hex(3)Fuc(1)	5.334	1.84.E-03
O43852	Isoform 3 of Calumenin	NATYGYVLDDPDPDDGFNYK	HexNAc(5)Hex(3)Fuc(1)	2.202	1.97.E-02
O60637	Tetraspanin-3	NQSVPLSCCR	HexNAc(5)Hex(3)Fuc(1)	9.283	3.07.E-03
O75054	Isoform 2 of Immunoglobulin superfamily member 3	VQGNSTLLHITDLQAR	HexNAc(5)Hex(3)Fuc(1)	100.000	1.00.E-17
O75976	Carboxypeptidase D	NVTVK	HexNAc(5)Hex(3)Fuc(1)	100.000	1.00.E-17
P04216	Thy-1 membrane glycoprotein	TNFTSK	HexNAc(5)Hex(3)Fuc(1)	100.000	1.00.E-17
P05556	Isoform 5 of Integrin beta-1	LRNPCTSEQNCTSPFSYK	HexNAc(5)Hex(3)Fuc(1)	100.000	1.00.E-17
P05556	Isoform 5 of Integrin beta-1	NVTNR	HexNAc(5)Hex(3)Fuc(1)	4.631	2.81.E-02
P06756	Integrin alpha-V	NMTISR	HexNAc(5)Hex(3)Fuc(1)	100.000	1.00.E-17
P06756	Integrin alpha-V	ANTTQPGIVEGGQVLK	HexNAc(5)Hex(3)Fuc(1)	100.000	1.00.E-17
P08962	CD63 antigen	NNHTASILDR	HexNAc(5)Hex(3)Fuc(1)	100.000	1.00.E-17
P10586	Receptor-type tyrosine-protein phosphatase F	VEVEPLNSTAVHVYWK	HexNAc(5)Hex(3)Fuc(1)	15.923	4.52.E-04
P23435	Cerebellin-2	STNHEPSEMSNR	HexNAc(5)Hex(3)Fuc(1)	100.000	1.00.E-17
P30533	Alpha-2-macroglobulin receptor-associated protein	VIDLWDLAQSANLTDK	HexNAc(5)Hex(3)Fuc(1)	100.000	1.00.E-17
P32004	Neural cell adhesion molecule L1	VPGNQTSTTLK	HexNAc(5)Hex(3)Fuc(1)	100.000	1.00.E-17
P32004	Neural cell adhesion molecule L1	VTYQNHNK	HexNAc(5)Hex(3)Fuc(1)	100.000	1.00.E-17
P51654	Isoform 3 of Glypican-3	NYTNAMFK	HexNAc(5)Hex(3)Fuc(1)	100.000	1.00.E-17
P78310	Coxsackievirus and adenovirus receptor	NASSEYSGTYSCTVR	HexNAc(5)Hex(3)Fuc(1)	100.000	1.00.E-17
Q01638	Interleukin-1 receptor-like 1	SPTFNR	HexNAc(5)Hex(3)Fuc(1)	100.000	1.00.E-17
Q07954	Prolow-density lipoprotein receptor-related protein 1	QSGDVTCNCTDGR	HexNAc(5)Hex(3)Fuc(1)	100.000	1.00.E-17
Q07954	Prolow-density lipoprotein receptor-related protein 1	TCPLDEFQCNNTLCKPLAWK	HexNAc(5)Hex(3)Fuc(1)	100.000	1.00.E-17
Q07954	Prolow-density lipoprotein receptor-related protein 1	DNATDSVPLR	HexNAc(5)Hex(3)Fuc(1)	100.000	1.00.E-17
Q07954	Prolow-density lipoprotein receptor-related protein 1	AVNSSCR	HexNAc(5)Hex(3)Fuc(1)	100.000	1.00.E-17
Q07954	Prolow-density lipoprotein receptor-related protein 1	CTQQVCAGYCANNSTCTVNQGNQPQCR	HexNAc(5)Hex(3)Fuc(1)	100.000	1.00.E-17
Q07954	Prolow-density lipoprotein receptor-related protein 1	CNASSQFLCSSGR	HexNAc(5)Hex(3)Fuc(1)	100.000	1.00.E-17
Q07954	Prolow-density lipoprotein receptor-related protein 1	MHLNGSNVQVLHR	HexNAc(5)Hex(3)Fuc(1)	100.000	1.00.E-17
Q07954	Prolow-density lipoprotein receptor-related protein 1	LTSCATNASICGDEAR	HexNAc(5)Hex(3)Fuc(1)	100.000	1.00.E-17
Q07954	Prolow-density lipoprotein receptor-related protein 1	WTGHNVTVVQR	HexNAc(5)Hex(3)Fuc(1)	15.634	8.37.E-04
Q07954	Prolow-density lipoprotein receptor-related protein 1	GVTHLNISGLK	HexNAc(5)Hex(3)Fuc(1)	4.125	2.02.E-02
Q13308	Isoform 6 of Inactive tyrosine-protein kinase 7	DDAGNYTCIASNGPQGQIR	HexNAc(5)Hex(3)Fuc(1)	100.000	1.00.E-17
Q13433	Zinc transporter ZIP6	YGENNSLSVEGFRK	HexNAc(5)Hex(3)Fuc(1)	100.000	1.00.E-17
Q14126	Desmoglein-2	INATDADEPNTLNSK	HexNAc(5)Hex(3)Fuc(1)	100.000	1.00.E-17
Q16851	UTP--glucose-1-phosphate uridylyltransferase	NVSLK	HexNAc(5)Hex(3)Fuc(1)	100.000	1.00.E-17
Q5ZPR3	CD276 antigen	DQGSAYANR	HexNAc(5)Hex(3)Fuc(1)	100.000	1.00.E-17
Q6UXK5	Leucine-rich repeat neuronal protein 1	SCVNVTTK	HexNAc(5)Hex(3)Fuc(1)	2.320	4.24.E-02
Q8IV08	Phospholipase D3	DNHTHSDIQVK	HexNAc(5)Hex(3)Fuc(1)	6.416	9.29.E-03
Q8N7P1	Inactive phospholipase D5	LFCPKNR	HexNAc(5)Hex(3)Fuc(1)	100.000	1.00.E-17
Q8NFT8	Delta and Notch-like epidermal growth factor-related receptor	KPCQNNASCIDANEK	HexNAc(5)Hex(3)Fuc(1)	100.000	1.00.E-17
Q8TAB3	Protocadherin-19	DGGLPSLQSNATVR	HexNAc(5)Hex(3)Fuc(1)	3.613	3.29.E-02
Q92608	Dedicator of cytokinesis protein 2	TVNR	HexNAc(5)Hex(3)Fuc(1)	6.361	6.46.E-03
Q92673	Sortilin-related receptor	NNTCVK	HexNAc(5)Hex(3)Fuc(1)	100.000	1.00.E-17
Q92854	Semaphorin-4D	AANYTSSLNLPDK	HexNAc(5)Hex(3)Fuc(1)	100.000	1.00.E-17
Q92859	Neogenin	TPASDPHGDNLTYSVFYTK	HexNAc(5)Hex(3)Fuc(1)	100.000	1.00.E-17
Q96MM7	Isoform 4 of Heparan-sulfate 6-O-sulfotransferase 2	YNFTR	HexNAc(5)Hex(3)Fuc(1)	100.000	1.00.E-17
Q9C019	Tripartite motif-containing protein 15	VNQSR	HexNAc(5)Hex(3)Fuc(1)	100.000	1.00.E-17
Q9H8M5	Metal transporter CNNM2	VYGQNINNETWSR	HexNAc(5)Hex(3)Fuc(1)	100.000	1.00.E-17
Q9HCU4	Cadherin EGF LAG seven-pass G-type receptor 2	LQRNESGLDSGR	HexNAc(5)Hex(3)Fuc(1)	100.000	1.00.E-17
Q9HD45	Transmembrane 9 superfamily member 3	IVDVNLTSEGK	HexNAc(5)Hex(3)Fuc(1)	100.000	1.00.E-17
Q9P2B2	Prostaglandin F2 receptor negative regulator	NVSVAEGK	HexNAc(5)Hex(3)Fuc(1)	100.000	1.00.E-17
Q9UHN6	Cell surface hyaluronidase	TTNSSAADPR	HexNAc(5)Hex(3)Fuc(1)	100.000	1.00.E-17
Q9UHN6	Cell surface hyaluronidase	HPSCVNVSK	HexNAc(5)Hex(3)Fuc(1)	100.000	1.00.E-17

O15031	Plexin-B2	SCVAVTSAQPQNMSR	HexNAc(5)Hex(3)	100.000	1.00.E-17
O43490	Prominin-1	EALENMNSTLK	HexNAc(5)Hex(3)	100.000	1.00.E-17
P07996	Thrombospondin-1	VVNSTTGPGEHLR	HexNAc(5)Hex(3)	16.199	1.57.E-04
P15586	N-acetylglucosamine-6-sulfatase	GPGIKPNQTSK	HexNAc(5)Hex(3)	100.000	1.00.E-17
P32004	Neural cell adhesion molecule L1	VTYQNHNK	HexNAc(5)Hex(3)	100.000	1.00.E-17
P41217	Isoform 3 of OX-2 membrane glycoprotein	FSEDHLNITCSATAR	HexNAc(5)Hex(3)	100.000	1.00.E-17
P42658	Dipeptidyl aminopeptidase-like protein 6	LAYAAINDSR	HexNAc(5)Hex(3)	100.000	1.00.E-17
P49750	Isoform 4 of YLP motif-containing protein 1	ALNR	HexNAc(5)Hex(3)	100.000	1.00.E-17
P78310	Coxsackievirus and adenovirus receptor	SGDASINVTNLQLSDIGTYQCK	HexNAc(5)Hex(3)	100.000	1.00.E-17
Q07954	Prolow-density lipoprotein receptor-related protein 1	FNSTEYQVVTR	HexNAc(5)Hex(3)	100.000	1.00.E-17
Q12860	Contactin-1	GNYSCFVSSPSITK	HexNAc(5)Hex(3)	100.000	1.00.E-17
Q15149	Plectin	NLVDNITGQR	HexNAc(5)Hex(3)	100.000	1.00.E-17
Q58EX2	Isoform 4 of Protein sidekick-2	VISAGGNDSR	HexNAc(5)Hex(3)	3.661	4.14.E-02
Q5NDL2	EGF domain-specific O-linked N-acetylglucosamine transferase	LNITQEGPK	HexNAc(5)Hex(3)	100.000	1.00.E-17
Q96PY6	Isoform 3 of Serine/threonine-protein kinase Nek1	LGPNGSPR	HexNAc(5)Hex(3)	100.000	1.00.E-17
Q9H0X4	Protein FAM234A	YKPDTLAVAVENGTGTDR	HexNAc(5)Hex(3)	100.000	1.00.E-17
Q9HCK4	Isoform 3 of Roundabout homolog 2	FHINK	HexNAc(5)Hex(3)	100.000	1.00.E-17
Q9HD45	Transmembrane 9 superfamily member 3	IVDVNLTSEGK	HexNAc(5)Hex(3)	100.000	1.00.E-17
P78310	Coxsackievirus and adenovirus receptor	NASSEYSGTYSCTVR	HexNAc(4)Hex(7)Fuc(2)	100.000	1.00.E-17
P61812	Isoform B of Transforming growth factor beta-2	NASNLVK	HexNAc(4)Hex(7)Fuc(1)	100.000	1.00.E-17
Q14157	Isoform 5 of Ubiquitin-associated protein 2-like	PQTNK	HexNAc(4)Hex(7)Fuc(1)	100.000	1.00.E-17
Q9H7E2	Isoform 3 of Tudor domain-containing protein 3	NDTR	HexNAc(4)Hex(7)Fuc(1)	2.545	3.28.E-02
Q9NX58	Cell growth-regulating nucleolar protein	PNVSPK	HexNAc(4)Hex(7)Fuc(1)	4.762	1.01.E-02
P35269	General transcription factor IIF subunit 1	NTTKK	HexNAc(4)Hex(7)	100.000	1.00.E-17
Q15910	Isoform 2 of Histone-lysine N-methyltransferase EZH2	NVSCK	HexNAc(4)Hex(7)	9.276	5.25.E-03
O15031	Plexin-B2	EAESLQPMTVVGTDYVFHNDTK	HexNAc(4)Hex(6)Fuc(2)	7.657	5.42.E-03
O75976	Carboxypeptidase D	LLNTTDVYLLPSLNPDGFER	HexNAc(4)Hex(6)Fuc(2)	100.000	1.00.E-17
P05556	Isoform 5 of Integrin beta-1	DTCTQECSYFNITK	HexNAc(4)Hex(6)Fuc(2)	7.355	2.29.E-03
Q06210	Glutamine--fructose-6-phosphate aminotransferase [isomerizing] 1	GSCNLSR	HexNAc(4)Hex(6)Fuc(2)	100.000	1.00.E-17
Q07954	Prolow-density lipoprotein receptor-related protein 1	LTSCATNASICGDEAR	HexNAc(4)Hex(6)Fuc(2)	3.472	1.58.E-02
Q13740	CD166 antigen	IIISPEENVTLTCTAENQLER	HexNAc(4)Hex(6)Fuc(2)	3.261	1.38.E-02
Q58EX2	Isoform 4 of Protein sidekick-2	IQTLQAPPDMAPANVSLR	HexNAc(4)Hex(6)Fuc(2)	4.035	2.67.E-02
Q5ZPR3	CD276 antigen	VVLGANGTYSCLVR	HexNAc(4)Hex(6)Fuc(2)	9.870	8.22.E-04
Q8NC26	Zinc finger protein 114	NHSK	HexNAc(4)Hex(6)Fuc(2)	100.000	1.00.E-17
Q9UBG0	C-type mannose receptor 2	WNDSPCNQSLPSICK	HexNAc(4)Hex(6)Fuc(2)	100.000	1.00.E-17
Q9UBG0	C-type mannose receptor 2	VTPACNTSLPAQR	HexNAc(4)Hex(6)Fuc(2)	4.288	1.37.E-02
P41217	Isoform 3 of OX-2 membrane glycoprotein	FSEDHLNITCSATAR	HexNAc(4)Hex(6)Fuc(1)NeuAc(1)	100.000	1.00.E-17
Q13905	Isoform Short of Rap guanine nucleotide exchange factor 1	NITRR	HexNAc(4)Hex(6)Fuc(1)NeuAc(1)	100.000	1.00.E-17
Q92733	Proline-rich protein PRCC	NLTVK	HexNAc(4)Hex(6)Fuc(1)NeuAc(1)	100.000	1.00.E-17
Q9HD45	Transmembrane 9 superfamily member 3	IVDVNLTSEGK	HexNAc(4)Hex(6)Fuc(1)NeuAc(1)	4.735	8.69.E-03
O94806	Serine/threonine-protein kinase D3	IPNNCSGVR	HexNAc(4)Hex(6)Fuc(1)	24.135	6.70.E-05
P0DP91	Chimeric ERCC6-PGBD3 protein	NTTFR	HexNAc(4)Hex(6)Fuc(1)	100.000	1.00.E-17
Q07954	Prolow-density lipoprotein receptor-related protein 1	VDIPQQPMGIIAVANDTNSCELSPCR	HexNAc(4)Hex(6)Fuc(1)	3.605	2.24.E-02
Q8NFZ8	Cell adhesion molecule 4	QTLFFNGTR	HexNAc(4)Hex(6)Fuc(1)	100.000	1.00.E-17
A0FGR8	Isoform 2 of Extended synaptotagmin-2	ENLSPK	HexNAc(4)Hex(6)	100.000	1.00.E-17
P11142	Heat shock cognate 71 kDa protein	NTTIPTK	HexNAc(4)Hex(6)	100.000	1.00.E-17
Q9UBS9	Isoform 2 of SUN domain-containing ossification factor	SISENATATAAPK	HexNAc(4)Hex(6)	100.000	1.00.E-17
O15031	Plexin-B2	SCVAVTSAQPQNMSR	HexNAc(4)Hex(5)NeuAc(2)	100.000	1.00.E-17
P02787	Serotransferrin	QQQHLFGSNVTDCSGNFCLFR	HexNAc(4)Hex(5)NeuAc(2)	6.208	1.92.E-03
P02787	Serotransferrin	CGLVPVLAENYNK	HexNAc(4)Hex(5)NeuAc(2)	5.718	3.42.E-03
P11717	Cation-independent mannose-6-phosphate receptor	TGPVVEDSGSLLLEYVNGSACTTSDGR	HexNAc(4)Hex(5)NeuAc(2)	2.805	2.70.E-02
P17301	Integrin alpha-2	YFFNVSDEAALLEK	HexNAc(4)Hex(5)NeuAc(2)	100.000	1.00.E-17
Q9H7E2	Isoform 3 of Tudor domain-containing protein 3	NDTR	HexNAc(4)Hex(5)NeuAc(2)	100.000	1.00.E-17
A1L157	Tetraspanin-11	QHLNR	HexNAc(4)Hex(5)NeuAc(1)	100.000	1.00.E-17
O14786	Neuropilin-1	RGPECSQNYTTPSGVIK	HexNAc(4)Hex(5)NeuAc(1)	100.000	1.00.E-17
O15372	Eukaryotic translation initiation factor 3 subunit H	NTSK	HexNAc(4)Hex(5)NeuAc(1)	100.000	1.00.E-17
O94874	E3 UFM1-protein ligase 1	PINK	HexNAc(4)Hex(5)NeuAc(1)	100.000	1.00.E-17
P02787	Serotransferrin	CGLVPVLAENYNK	HexNAc(4)Hex(5)NeuAc(1)	3.755	1.38.E-02
P05556	Isoform 5 of Integrin beta-1	CHEGNGTFECGACR	HexNAc(4)Hex(5)NeuAc(1)	100.000	1.00.E-17
P08069	Insulin-like growth factor 1 receptor	IQATSLSGNGSWTDPVFFYVQAK	HexNAc(4)Hex(5)NeuAc(1)	12.547	3.76.E-03
P17301	Integrin alpha-2	YFFNVSDEAALLEK	HexNAc(4)Hex(5)NeuAc(1)	3.784	2.04.E-02
P41217	Isoform 3 of OX-2 membrane glycoprotein	FSEDHLNITCSATAR	HexNAc(4)Hex(5)NeuAc(1)	3.621	1.37.E-02
P51805	Plexin-A3	SLNR	HexNAc(4)Hex(5)NeuAc(1)	100.000	1.00.E-17
Q07954	Prolow-density lipoprotein receptor-related protein 1	VDIPQQPMGIIAVANDTNSCELSPCR	HexNAc(4)Hex(5)NeuAc(1)	3.142	2.99.E-02
Q13308	Isoform 6 of Inactive tyrosine-protein kinase 7	NLTLR	HexNAc(4)Hex(5)NeuAc(1)	100.000	1.00.E-17
Q7LGA3	Heparan sulfate 2-O-sulfotransferase 1	YHVLHINTTK	HexNAc(4)Hex(5)NeuAc(1)	100.000	1.00.E-17
Q7LGA3	Heparan sulfate 2-O-sulfotransferase 1	NITSWK	HexNAc(4)Hex(5)NeuAc(1)	4.351	1.80.E-02
Q9GZZ0	Homeobox protein Hox-D1	TNFSTK	HexNAc(4)Hex(5)NeuAc(1)	15.198	1.81.E-04
P02786	Transferrin receptor protein 1	KDFEDLYTPVNGSIVIVR	HexNAc(4)Hex(5)Fuc(4)	100.000	1.00.E-17
P22681	E3 ubiquitin-protein ligase CBL	NLTK	HexNAc(4)Hex(5)Fuc(4)	100.000	1.00.E-17
Q5ZPR3	CD276 antigen	QLVHSFAEGQDQGSAYANR	HexNAc(4)Hex(5)Fuc(4)	100.000	1.00.E-17
Q9Y3Z3	Deoxynucleoside triphosphate triphosphohydrolase SAMHD1	PENK	HexNAc(4)Hex(5)Fuc(4)	100.000	1.00.E-17
Q9UBG0	C-type mannose receptor 2	KKPNATAEPTPPDR	HexNAc(4)Hex(5)Fuc(3)NeuAc(2)	100.000	1.00.E-17
P02786	Transferrin receptor protein 1	DFEDLYTPVNGSIVIVR	HexNAc(4)Hex(5)Fuc(3)NeuAc(1)	3.163	1.86.E-02
Q12907	Vesicular integral-membrane protein VIP36	VFPYISVMVNNGSLSYDHSK	HexNAc(4)Hex(5)Fuc(3)NeuAc(1)	2.587	3.75.E-02
Q13308	Isoform 6 of Inactive tyrosine-protein kinase 7	DGTPLSDGQSNHTVSSK	HexNAc(4)Hex(5)Fuc(3)NeuAc(1)	100.000	1.00.E-17
Q13683	Integrin alpha-7	PQSWLLVGAPQALALPGQQANR	HexNAc(4)Hex(5)Fuc(3)NeuAc(1)	14.063	6.68.E-04
Q92823	Neuronal cell adhesion molecule	VNVVNSTLAEVHWDPVPLK	HexNAc(4)Hex(5)Fuc(3)NeuAc(1)	3.142	4.65.E-02

Q9C0H2	Isoform 4 of Protein tweety homolog 3	VQEVLNGTEVNLQHLTALVDCR	HexNAc(4)Hex(5)Fuc(3)NeuAc(1)	4.934	1.87.E-02
O15031	Plexin-B2	EAESLQPMTVVGTDYVFHNDTK	HexNAc(4)Hex(5)Fuc(3)	6.185	3.92.E-03
O43157	Plexin-B1	LTLNGSK	HexNAc(4)Hex(5)Fuc(3)	100.000	1.00.E-17
O60293	Zinc finger C3H1 domain-containing protein	NFSR	HexNAc(4)Hex(5)Fuc(3)	100.000	1.00.E-17
O75976	Carboxypeptidase D	NVTVK	HexNAc(4)Hex(5)Fuc(3)	3.400	2.63.E-02
P05556	Isoform 5 of Integrin beta-1	LRNPCTSEQNCTSPFSYK	HexNAc(4)Hex(5)Fuc(3)	5.976	9.83.E-03
P06756	Integrin alpha-V	ENQNHSYSLK	HexNAc(4)Hex(5)Fuc(3)	100.000	1.00.E-17
P08648	Integrin alpha-5	TEKEPLSDPVGTCYLSTDNFTR	HexNAc(4)Hex(5)Fuc(3)	4.723	8.13.E-03
P15151	Poliovirus receptor	VEDEGNYTCLFVTFPQGSR	HexNAc(4)Hex(5)Fuc(3)	8.988	2.22.E-03
P19022	Cadherin-2	SNISILR	HexNAc(4)Hex(5)Fuc(3)	100.000	1.00.E-17
Q01638	Interleukin-1 receptor-like 1	SPTFNR	HexNAc(4)Hex(5)Fuc(3)	3.795	2.20.E-02
Q07954	Prolow-density lipoprotein receptor-related protein 1	CNASSQFLCSSGR	HexNAc(4)Hex(5)Fuc(3)	100.000	1.00.E-17
Q07954	Prolow-density lipoprotein receptor-related protein 1	TCPLDEFQCNNTLCKPLAWK	HexNAc(4)Hex(5)Fuc(3)	9.579	4.20.E-04
Q08722	Leukocyte surface antigen CD47	SDAVSHTGNYTCEVTELTR	HexNAc(4)Hex(5)Fuc(3)	100.000	1.00.E-17
Q13683	Integrin alpha-7	PQSWLLVGAPQALALPGQQANR	HexNAc(4)Hex(5)Fuc(3)	17.443	2.36.E-04
Q13740	CD166 antigen	LGDCISEDSYPDGNITWYR	HexNAc(4)Hex(5)Fuc(3)	100.000	1.00.E-17
Q7Z4K8	Tripartite motif-containing protein 46	NLTLER	HexNAc(4)Hex(5)Fuc(3)	3.662	2.59.E-02
Q9UBG0	C-type mannose receptor 2	NCTSFR	HexNAc(4)Hex(5)Fuc(3)	100.000	1.00.E-17
Q9UBG0	C-type mannose receptor 2	VTPACNTSLPAQR	HexNAc(4)Hex(5)Fuc(3)	100.000	1.00.E-17
O75449	Katanin p60 ATPase-containing subunit A1	EEKNK	HexNAc(4)Hex(5)Fuc(2)NeuAc(1)	100.000	1.00.E-17
P05556	Isoform 5 of Integrin beta-1	DTCTQECSYFNITK	HexNAc(4)Hex(5)Fuc(2)NeuAc(1)	2.270	3.92.E-02
P08069	Insulin-like growth factor 1 receptor	NITR	HexNAc(4)Hex(5)Fuc(2)NeuAc(1)	100.000	1.00.E-17
P08648	Integrin alpha-5	VTGLNCTTNHPINPK	HexNAc(4)Hex(5)Fuc(2)NeuAc(1)	7.150	5.61.E-03
P17301	Integrin alpha-2	TASCSNVTCWLK	HexNAc(4)Hex(5)Fuc(2)NeuAc(1)	100.000	1.00.E-17
P19022	Cadherin-2	SNISILR	HexNAc(4)Hex(5)Fuc(2)NeuAc(1)	2.351	3.46.E-02
P57087	Isoform 3 of Junctional adhesion molecule B	LGSQSTNSSYTMNTK	HexNAc(4)Hex(5)Fuc(2)NeuAc(1)	100.000	1.00.E-17
Q96AC1	Isoform 3 of Fermitin family homolog 2	VNFSDR	HexNAc(4)Hex(5)Fuc(2)NeuAc(1)	3.665	4.76.E-02
Q9UBG0	C-type mannose receptor 2	WNDSPCNQSLPSICK	HexNAc(4)Hex(5)Fuc(2)NeuAc(1)	3.776	1.62.E-02
O15031	Plexin-B2	SCVAVTSAQPQNMSR	HexNAc(4)Hex(5)Fuc(2)	100.000	1.00.E-17
O15394	Neural cell adhesion molecule 2	NTTNLK	HexNAc(4)Hex(5)Fuc(2)	100.000	1.00.E-17
O75976	Carboxypeptidase D	NVTVK	HexNAc(4)Hex(5)Fuc(2)	3.180	2.07.E-02
O94806	Serine/threonine-protein kinase D3	IPNNCSGVR	HexNAc(4)Hex(5)Fuc(2)	100.000	1.00.E-17
P06756	Integrin alpha-V	ENQNHSYSLK	HexNAc(4)Hex(5)Fuc(2)	100.000	1.00.E-17
P06756	Integrin alpha-V	NMTISR	HexNAc(4)Hex(5)Fuc(2)	2.306	4.37.E-02
Q07954	Prolow-density lipoprotein receptor-related protein 1	CNASSQFLCSSGR	HexNAc(4)Hex(5)Fuc(2)	100.000	1.00.E-17
Q07954	Prolow-density lipoprotein receptor-related protein 1	VDIPQQPMGIIAVANDTNSCELSPCR	HexNAc(4)Hex(5)Fuc(2)	5.259	7.53.E-03
Q08722	Leukocyte surface antigen CD47	SDAVSHTGNYTCEVTELTR	HexNAc(4)Hex(5)Fuc(2)	100.000	1.00.E-17
Q9UBG0	C-type mannose receptor 2	NCTSFR	HexNAc(4)Hex(5)Fuc(2)	100.000	1.00.E-17
Q9UBG0	C-type mannose receptor 2	VTPACNTSLPAQR	HexNAc(4)Hex(5)Fuc(2)	3.878	2.52.E-02
O15031	Plexin-B2	NCSFQPER	HexNAc(4)Hex(5)Fuc(1)NeuAc(2)	100.000	1.00.E-17
O75976	Carboxypeptidase D	RFANEYPNITR	HexNAc(4)Hex(5)Fuc(1)NeuAc(2)	7.476	1.00.E-02
O75976	Carboxypeptidase D	LLNTTDVYLLPSLNPDGFER	HexNAc(4)Hex(5)Fuc(1)NeuAc(2)	5.519	4.99.E-03
O75976	Carboxypeptidase D	FANEYPNITR	HexNAc(4)Hex(5)Fuc(1)NeuAc(2)	3.896	1.32.E-02
P02786	Transferrin receptor protein 1	DFEDLYTPVNGSIVIVR	HexNAc(4)Hex(5)Fuc(1)NeuAc(2)	3.163	1.68.E-02
P08648	Integrin alpha-5	VTGLNCTTNHPINPK	HexNAc(4)Hex(5)Fuc(1)NeuAc(2)	100.000	1.00.E-17
P57087	Isoform 3 of Junctional adhesion molecule B	LGSQSTNSSYTMNTK	HexNAc(4)Hex(5)Fuc(1)NeuAc(2)	100.000	1.00.E-17
Q07954	Prolow-density lipoprotein receptor-related protein 1	TCVSNCTASQFVCK	HexNAc(4)Hex(5)Fuc(1)NeuAc(2)	100.000	1.00.E-17
Q13308	Isoform 6 of Inactive tyrosine-protein kinase 7	DGTPLSDGQSNHTVSSK	HexNAc(4)Hex(5)Fuc(1)NeuAc(2)	100.000	1.00.E-17
Q13308	Isoform 6 of Inactive tyrosine-protein kinase 7	SANASFNIK	HexNAc(4)Hex(5)Fuc(1)NeuAc(2)	5.510	1.43.E-02
Q13683	Integrin alpha-7	PQSWLLVGAPQALALPGQQANR	HexNAc(4)Hex(5)Fuc(1)NeuAc(2)	14.063	6.68.E-04
Q5IJ48	Protein crumbs homolog 2	EGPPAAFSGHNASSGR	HexNAc(4)Hex(5)Fuc(1)NeuAc(2)	100.000	1.00.E-17
Q5ZPR3	CD276 antigen	QLVHSFAEGQDQGSAYANR	HexNAc(4)Hex(5)Fuc(1)NeuAc(2)	2.387	4.51.E-02
Q8IV08	Phospholipase D3	DNHTHSDIQVK	HexNAc(4)Hex(5)Fuc(1)NeuAc(2)	7.832	6.97.E-03
Q9C0H2	Isoform 4 of Protein tweety homolog 3	VQEVLNGTEVNLQHLTALVDCR	HexNAc(4)Hex(5)Fuc(1)NeuAc(2)	15.162	7.47.E-04
Q9UBG0	C-type mannose receptor 2	TSNISKPGTLER	HexNAc(4)Hex(5)Fuc(1)NeuAc(2)	100.000	1.00.E-17
Q9UBG0	C-type mannose receptor 2	KKPNATAEPTPPDR	HexNAc(4)Hex(5)Fuc(1)NeuAc(2)	17.830	5.33.E-04
Q9UBG0	C-type mannose receptor 2	VTPACNTSLPAQR	HexNAc(4)Hex(5)Fuc(1)NeuAc(2)	6.553	8.07.E-03
Q9UBG0	C-type mannose receptor 2	WNDSPCNQSLPSICK	HexNAc(4)Hex(5)Fuc(1)NeuAc(2)	6.545	3.35.E-03
O14672	Disintegrin and metalloproteinase domain-containing protein 10	EGICNGFTALCPASDPKPNFTDCNR	HexNAc(4)Hex(5)Fuc(1)NeuAc(1)	100.000	1.00.E-17
O14786	Neuropilin-1	RGPECSQNYTTPSGVIK	HexNAc(4)Hex(5)Fuc(1)NeuAc(1)	100.000	1.00.E-17
O15031	Plexin-B2	EAESLQPMTVVGTDYVFHNDTK	HexNAc(4)Hex(5)Fuc(1)NeuAc(1)	6.185	3.92.E-03
O15031	Plexin-B2	ALSNISLR	HexNAc(4)Hex(5)Fuc(1)NeuAc(1)	3.546	1.88.E-02
O15031	Plexin-B2	NCSFQPER	HexNAc(4)Hex(5)Fuc(1)NeuAc(1)	3.375	2.06.E-02
O43157	Plexin-B1	YTLDPNITSAGPTK	HexNAc(4)Hex(5)Fuc(1)NeuAc(1)	100.000	1.00.E-17
O75976	Carboxypeptidase D	DLDTDFTNNASQPETK	HexNAc(4)Hex(5)Fuc(1)NeuAc(1)	100.000	1.00.E-17
P02786	Transferrin receptor protein 1	DFEDLYTPVNGSIVIVR	HexNAc(4)Hex(5)Fuc(1)NeuAc(1)	7.465	4.94.E-03
P02786	Transferrin receptor protein 1	KDFEDLYTPVNGSIVIVR	HexNAc(4)Hex(5)Fuc(1)NeuAc(1)	6.301	3.21.E-03
P05556	Isoform 5 of Integrin beta-1	NPCTSEQNCTSPFSYK	HexNAc(4)Hex(5)Fuc(1)NeuAc(1)	100.000	1.00.E-17
P05556	Isoform 5 of Integrin beta-1	DTCTQECSYFNITK	HexNAc(4)Hex(5)Fuc(1)NeuAc(1)	9.478	7.73.E-04
P05556	Isoform 5 of Integrin beta-1	KDTCTQECSYFNITK	HexNAc(4)Hex(5)Fuc(1)NeuAc(1)	9.215	2.66.E-04
P05556	Isoform 5 of Integrin beta-1	LRNPCTSEQNCTSPFSYK	HexNAc(4)Hex(5)Fuc(1)NeuAc(1)	5.529	6.56.E-03
P05556	Isoform 5 of Integrin beta-1	NGVNGTGENGRK	HexNAc(4)Hex(5)Fuc(1)NeuAc(1)	2.715	4.37.E-02
P06756	Integrin alpha-V	ENQNHSYSLK	HexNAc(4)Hex(5)Fuc(1)NeuAc(1)	22.690	1.38.E-04
P06756	Integrin alpha-V	NMTISR	HexNAc(4)Hex(5)Fuc(1)NeuAc(1)	8.856	6.60.E-04
P08648	Integrin alpha-5	VTGLNCTTNHPINPK	HexNAc(4)Hex(5)Fuc(1)NeuAc(1)	100.000	1.00.E-17
P08648	Integrin alpha-5	TEKEPLSDPVGTCYLSTDNFTR	HexNAc(4)Hex(5)Fuc(1)NeuAc(1)	4.723	8.13.E-03
P14384	Carboxypeptidase M	TVAQNYSSVTHLHSIGK	HexNAc(4)Hex(5)Fuc(1)NeuAc(1)	100.000	1.00.E-17
P15151	Poliovirus receptor	VEDEGNYTCLFVTFPQGSR	HexNAc(4)Hex(5)Fuc(1)NeuAc(1)	100.000	1.00.E-17
P17301	Integrin alpha-2	LNLQTSTSIPNVTEMK	HexNAc(4)Hex(5)Fuc(1)NeuAc(1)	100.000	1.00.E-17
P17301	Integrin alpha-2	TASCSNVTCWLK	HexNAc(4)Hex(5)Fuc(1)NeuAc(1)	2.616	2.31.E-02
P19022	Cadherin-2	SNISILR	HexNAc(4)Hex(5)Fuc(1)NeuAc(1)	9.958	3.45.E-04

P26006	Isoform 2 of Integrin alpha-3	ELAVPDGYTNR	HexNAc(4)Hex(5)Fuc(1)NeuAc(1)	100.000	1.00.E-17
P46100	Transcriptional regulator ATRX	RNLSSK	HexNAc(4)Hex(5)Fuc(1)NeuAc(1)	29.159	1.68.E-05
P54709	Sodium/potassium-transporting ATPase subunit beta-3	NLTVCPDGALFEQK	HexNAc(4)Hex(5)Fuc(1)NeuAc(1)	3.090	1.14.E-02
P57087	Isoform 3 of Junctional adhesion molecule B	IKNVTR	HexNAc(4)Hex(5)Fuc(1)NeuAc(1)	6.819	9.61.E-03
P78504	Protein jagged-1	CPEDYEGKNCSHLK	HexNAc(4)Hex(5)Fuc(1)NeuAc(1)	100.000	1.00.E-17
P78504	Protein jagged-1	NCSHLK	HexNAc(4)Hex(5)Fuc(1)NeuAc(1)	6.532	4.65.E-03
P98164	Low-density lipoprotein receptor-related protein 2	TWSAFQNGTDRR	HexNAc(4)Hex(5)Fuc(1)NeuAc(1)	100.000	1.00.E-17
P98164	Low-density lipoprotein receptor-related protein 2	NLTNPR	HexNAc(4)Hex(5)Fuc(1)NeuAc(1)	5.182	1.46.E-02
Q06210	Glutamine--fructose-6-phosphate aminotransferase [isomerizing] 1	GSCNLSR	HexNAc(4)Hex(5)Fuc(1)NeuAc(1)	100.000	1.00.E-17
Q07954	Prolow-density lipoprotein receptor-related protein 1	TCPLDEFQCNNTLCK	HexNAc(4)Hex(5)Fuc(1)NeuAc(1)	100.000	1.00.E-17
Q07954	Prolow-density lipoprotein receptor-related protein 1	DNATDSVPLR	HexNAc(4)Hex(5)Fuc(1)NeuAc(1)	100.000	1.00.E-17
Q07954	Prolow-density lipoprotein receptor-related protein 1	THANGSIK	HexNAc(4)Hex(5)Fuc(1)NeuAc(1)	13.863	8.34.E-04
Q07954	Prolow-density lipoprotein receptor-related protein 1	LTSCATNASICGDEAR	HexNAc(4)Hex(5)Fuc(1)NeuAc(1)	4.403	7.85.E-03
Q07954	Prolow-density lipoprotein receptor-related protein 1	IETILLNGTDRK	HexNAc(4)Hex(5)Fuc(1)NeuAc(1)	3.944	4.87.E-03
Q07954	Prolow-density lipoprotein receptor-related protein 1	IETILLNGTDR	HexNAc(4)Hex(5)Fuc(1)NeuAc(1)	3.146	4.15.E-02
Q07954	Prolow-density lipoprotein receptor-related protein 1	TCPLDEFQCNNTLCKPLAWK	HexNAc(4)Hex(5)Fuc(1)NeuAc(1)	2.468	4.43.E-02
Q07954	Prolow-density lipoprotein receptor-related protein 1	GVTHLNISGLK	HexNAc(4)Hex(5)Fuc(1)NeuAc(1)	2.394	3.46.E-02
Q08722	Leukocyte surface antigen CD47	SDAVSHTGNYTCEVTELTR	HexNAc(4)Hex(5)Fuc(1)NeuAc(1)	100.000	1.00.E-17
Q13308	Isoform 6 of Inactive tyrosine-protein kinase 7	DDAGNYTCIASNGPQGQIR	HexNAc(4)Hex(5)Fuc(1)NeuAc(1)	2.259	3.49.E-02
Q13433	Zinc transporter ZIP6	NTNENPQECFNASK	HexNAc(4)Hex(5)Fuc(1)NeuAc(1)	5.397	3.94.E-03
Q13641	Trophoblast glycoprotein	NLTEVPTDLPAYVR	HexNAc(4)Hex(5)Fuc(1)NeuAc(1)	100.000	1.00.E-17
Q13740	CD166 antigen	NATVVWMK	HexNAc(4)Hex(5)Fuc(1)NeuAc(1)	4.856	5.92.E-03
Q58EX2	Isoform 4 of Protein sidekick-2	IQTLQAPPDMAPANVSLR	HexNAc(4)Hex(5)Fuc(1)NeuAc(1)	8.986	3.82.E-03
Q58EX2	Isoform 4 of Protein sidekick-2	LNTTTANTATVEVLAPSAR	HexNAc(4)Hex(5)Fuc(1)NeuAc(1)	4.831	4.81.E-03
Q5ZPR3	CD276 antigen	DQGSAYANR	HexNAc(4)Hex(5)Fuc(1)NeuAc(1)	7.477	4.28.E-03
Q5ZPR3	CD276 antigen	VVLGANGTYSCLVR	HexNAc(4)Hex(5)Fuc(1)NeuAc(1)	5.073	6.12.E-03
Q5ZPR3	CD276 antigen	QLVHSFAEGQDQGSAYANR	HexNAc(4)Hex(5)Fuc(1)NeuAc(1)	4.994	2.15.E-02
Q6UVY6	DBH-like monooxygenase protein 1	NLSFMDAMNK	HexNAc(4)Hex(5)Fuc(1)NeuAc(1)	100.000	1.00.E-17
Q8NHM5	Isoform 3 of Lysine-specific demethylase 2B	GLNGTPR	HexNAc(4)Hex(5)Fuc(1)NeuAc(1)	100.000	1.00.E-17
Q8WXH0	Isoform 2 of Nesprin-2	KLNENK	HexNAc(4)Hex(5)Fuc(1)NeuAc(1)	100.000	1.00.E-17
Q92608	Dedicator of cytokinesis protein 2	TVNR	HexNAc(4)Hex(5)Fuc(1)NeuAc(1)	6.501	6.99.E-03
Q92896	Isoform 2 of Golgi apparatus protein 1	GNITEYQCHQYITK	HexNAc(4)Hex(5)Fuc(1)NeuAc(1)	100.000	1.00.E-17
Q96AC1	Isoform 3 of Fermitin family homolog 2	VNFSDR	HexNAc(4)Hex(5)Fuc(1)NeuAc(1)	14.729	8.25.E-04
Q99523	Sortilin	DITDLINNTFIR	HexNAc(4)Hex(5)Fuc(1)NeuAc(1)	100.000	1.00.E-17
Q9BX67	Junctional adhesion molecule C	IWNVTR	HexNAc(4)Hex(5)Fuc(1)NeuAc(1)	6.880	1.74.E-03
Q9HCU4	Cadherin EGF LAG seven-pass G-type receptor 2	GNELSLVLLNASTGELK	HexNAc(4)Hex(5)Fuc(1)NeuAc(1)	4.779	1.51.E-02
Q9HCU4	Cadherin EGF LAG seven-pass G-type receptor 2	LQRNESGLDSGR	HexNAc(4)Hex(5)Fuc(1)NeuAc(1)	2.759	4.32.E-02
Q9NYI0	PH and SEC7 domain-containing protein 3	TQRVNK	HexNAc(4)Hex(5)Fuc(1)NeuAc(1)	4.295	6.97.E-03
Q9UBG0	C-type mannose receptor 2	KPNATAEPTPPDR	HexNAc(4)Hex(5)Fuc(1)NeuAc(1)	100.000	1.00.E-17
Q9UBG0	C-type mannose receptor 2	WNDSPCNQSLPSICK	HexNAc(4)Hex(5)Fuc(1)NeuAc(1)	46.644	1.72.E-06
Q9UBG0	C-type mannose receptor 2	KKPNATAEPTPPDR	HexNAc(4)Hex(5)Fuc(1)NeuAc(1)	16.012	1.40.E-03
Q9UBG0	C-type mannose receptor 2	TSNISKPGTLER	HexNAc(4)Hex(5)Fuc(1)NeuAc(1)	11.383	3.29.E-03
Q9UBG0	C-type mannose receptor 2	NCTSFR	HexNAc(4)Hex(5)Fuc(1)NeuAc(1)	8.846	1.03.E-03
Q9UBG0	C-type mannose receptor 2	VTPACNTSLPAQR	HexNAc(4)Hex(5)Fuc(1)NeuAc(1)	5.473	1.37.E-03
Q9UHN6	Cell surface hyaluronidase	NFTSVHLSYVELK	HexNAc(4)Hex(5)Fuc(1)NeuAc(1)	4.463	5.91.E-03
Q9UHN6	Cell surface hyaluronidase	QLNISHLLVPLGLAK	HexNAc(4)Hex(5)Fuc(1)NeuAc(1)	2.173	4.03.E-02
O15031	Plexin-B2	TEAGAFEYVPDPTFENFTGGVK	HexNAc(4)Hex(5)Fuc(1)	3.756	1.76.E-02
O75976	Carboxypeptidase D	NVTVK	HexNAc(4)Hex(5)Fuc(1)	7.937	5.14.E-03
P05556	Isoform 5 of Integrin beta-1	NVTNR	HexNAc(4)Hex(5)Fuc(1)	100.000	1.00.E-17
P05556	Isoform 5 of Integrin beta-1	NPCTSEQNCTSPFSYK	HexNAc(4)Hex(5)Fuc(1)	100.000	1.00.E-17
P05556	Isoform 5 of Integrin beta-1	LRNPCTSEQNCTSPFSYK	HexNAc(4)Hex(5)Fuc(1)	100.000	1.00.E-17
P06756	Integrin alpha-V	NMTISR	HexNAc(4)Hex(5)Fuc(1)	23.994	2.98.E-04
P06756	Integrin alpha-V	ANTTQPGIVEGGQVLK	HexNAc(4)Hex(5)Fuc(1)	14.993	1.01.E-04
P08648	Integrin alpha-5	VTGLNCTTNHPINPK	HexNAc(4)Hex(5)Fuc(1)	100.000	1.00.E-17
P11279	Lysosome-associated membrane glycoprotein 1	SSCGKENTSDPSLVIAFGR	HexNAc(4)Hex(5)Fuc(1)	100.000	1.00.E-17
P13473	Isoform LAMP-2C of Lysosome-associated membrane glycoprotein 2	VQPFNVTQGK	HexNAc(4)Hex(5)Fuc(1)	14.527	1.27.E-03
P19022	Cadherin-2	SNISILR	HexNAc(4)Hex(5)Fuc(1)	17.814	1.19.E-04
P32004	Neural cell adhesion molecule L1	VPGNQTSTTLK	HexNAc(4)Hex(5)Fuc(1)	100.000	1.00.E-17
P41217	Isoform 3 of OX-2 membrane glycoprotein	FSEDHLNITCSATAR	HexNAc(4)Hex(5)Fuc(1)	100.000	1.00.E-17
P42892	Endothelin-converting enzyme 1	HLLENSTASVSEAER	HexNAc(4)Hex(5)Fuc(1)	100.000	1.00.E-17
P42892	Endothelin-converting enzyme 1	HLLENSTASVSEAERK	HexNAc(4)Hex(5)Fuc(1)	100.000	1.00.E-17
P51654	Isoform 3 of Glypican-3	NYTNAMFK	HexNAc(4)Hex(5)Fuc(1)	3.611	3.05.E-02
P56199	Integrin alpha-1	VYVYALNQTR	HexNAc(4)Hex(5)Fuc(1)	100.000	1.00.E-17
P78310	Coxsackievirus and adenovirus receptor	NASSEYSGTYSCTVR	HexNAc(4)Hex(5)Fuc(1)	100.000	1.00.E-17
P98164	Low-density lipoprotein receptor-related protein 2	NLTNPR	HexNAc(4)Hex(5)Fuc(1)	3.445	3.29.E-02
Q06210	Glutamine--fructose-6-phosphate aminotransferase [isomerizing] 1	GSCNLSR	HexNAc(4)Hex(5)Fuc(1)	5.356	1.89.E-02
Q07954	Prolow-density lipoprotein receptor-related protein 1	TCPLDEFQCNNTLCK	HexNAc(4)Hex(5)Fuc(1)	100.000	1.00.E-17
Q07954	Prolow-density lipoprotein receptor-related protein 1	AVNSSCR	HexNAc(4)Hex(5)Fuc(1)	16.134	4.02.E-04
Q07954	Prolow-density lipoprotein receptor-related protein 1	CNASSQFLCSSGR	HexNAc(4)Hex(5)Fuc(1)	15.110	9.43.E-05
Q07954	Prolow-density lipoprotein receptor-related protein 1	FGTCSQLCNNTK	HexNAc(4)Hex(5)Fuc(1)	7.862	3.87.E-03
Q07954	Prolow-density lipoprotein receptor-related protein 1	THANGSIK	HexNAc(4)Hex(5)Fuc(1)	7.077	4.04.E-03
Q07954	Prolow-density lipoprotein receptor-related protein 1	WTGHNVTVVQR	HexNAc(4)Hex(5)Fuc(1)	3.771	1.05.E-02
Q07954	Prolow-density lipoprotein receptor-related protein 1	DNATDSVPLR	HexNAc(4)Hex(5)Fuc(1)	3.296	2.71.E-02
Q07954	Prolow-density lipoprotein receptor-related protein 1	TCVSNCTASQFVCK	HexNAc(4)Hex(5)Fuc(1)	2.927	2.80.E-02
Q07954	Prolow-density lipoprotein receptor-related protein 1	TCPLDEFQCNNTLCKPLAWK	HexNAc(4)Hex(5)Fuc(1)	2.450	4.37.E-02
Q08722	Leukocyte surface antigen CD47	SDAVSHTGNYTCEVTELTR	HexNAc(4)Hex(5)Fuc(1)	4.940	1.34.E-02
Q13308	Isoform 6 of Inactive tyrosine-protein kinase 7	DGTPLSDGQSNHTVSSK	HexNAc(4)Hex(5)Fuc(1)	2.977	2.89.E-02
Q7LGA3	Heparan sulfate 2-O-sulfotransferase 1	NITSWK	HexNAc(4)Hex(5)Fuc(1)	100.000	1.00.E-17
Q8NHM5	Isoform 3 of Lysine-specific demethylase 2B	GLNGTPR	HexNAc(4)Hex(5)Fuc(1)	100.000	1.00.E-17
Q96AC1	Isoform 3 of Fermitin family homolog 2	VNFSDR	HexNAc(4)Hex(5)Fuc(1)	100.000	1.00.E-17

Q9BX67	Junctional adhesion molecule C	IWNVTR	HexNAc(4)Hex(5)Fuc(1)	100.000	1.00.E-17
Q9HAR2	Isoform 4 of Adhesion G protein-coupled receptor L3	NLTPGGK	HexNAc(4)Hex(5)Fuc(1)	100.000	1.00.E-17
Q9UBG0	C-type mannose receptor 2	NCTSFR	HexNAc(4)Hex(5)Fuc(1)	100.000	1.00.E-17
Q9UBG0	C-type mannose receptor 2	WNDSPCNQSLPSICK	HexNAc(4)Hex(5)Fuc(1)	100.000	1.00.E-17
Q9UBG0	C-type mannose receptor 2	VTPACNTSLPAQR	HexNAc(4)Hex(5)Fuc(1)	12.871	3.99.E-04
Q9UHN6	Cell surface hyaluronidase	TTNSSAADPR	HexNAc(4)Hex(5)Fuc(1)	5.065	1.84.E-02
A8K979	ERI1 exoribonuclease 2	NLSISTK	HexNAc(4)Hex(5)	100.000	1.00.E-17
O15031	Plexin-B2	SCVAVTSAQPQNMSR	HexNAc(4)Hex(5)	3.471	2.55.E-02
P06756	Integrin alpha-V	ENQNHSYSLK	HexNAc(4)Hex(5)	3.154	3.83.E-02
P14415	Sodium/potassium-transporting ATPase subunit beta-2	ACQFNR	HexNAc(4)Hex(5)	100.000	1.00.E-17
P26006	Isoform 2 of Integrin alpha-3	AHCVWLECPIPDAPVVTNVTVK	HexNAc(4)Hex(5)	5.877	9.09.E-03
P42892	Endothelin-converting enzyme 1	ACMNETR	HexNAc(4)Hex(5)	100.000	1.00.E-17
P61201	Isoform 2 of COP9 signalosome complex subunit 2	NYSEK	HexNAc(4)Hex(5)	100.000	1.00.E-17
Q07954	Prolow-density lipoprotein receptor-related protein 1	FNSTEYQVVTR	HexNAc(4)Hex(5)	2.792	4.69.E-02
Q15910	Isoform 2 of Histone-lysine N-methyltransferase EZH2	NCSIQR	HexNAc(4)Hex(5)	100.000	1.00.E-17
Q6UVY6	DBH-like monooxygenase protein 1	INLTR	HexNAc(4)Hex(5)	100.000	1.00.E-17
Q6UX52	Uncharacterized protein C17orf99	ANFTLQDR	HexNAc(4)Hex(5)	100.000	1.00.E-17
Q96JE9	Microtubule-associated protein 6	NESPVISAPVK	HexNAc(4)Hex(5)	100.000	1.00.E-17
Q9Y639	Neuroplastin	NASNMEYR	HexNAc(4)Hex(5)	100.000	1.00.E-17
Q9Y639	Neuroplastin	ANATIEVK	HexNAc(4)Hex(5)	100.000	1.00.E-17
P13473	Isoform LAMP-2C of Lysosome-associated membrane glycoprotein 2	LNSSTIK	HexNAc(4)Hex(4)NeuAc(1)	100.000	1.00.E-17
P42696	RNA-binding protein 34	LKNVSKPK	HexNAc(4)Hex(4)NeuAc(1)	100.000	1.00.E-17
Q7LGA3	Heparan sulfate 2-O-sulfotransferase 1	NITSWK	HexNAc(4)Hex(4)NeuAc(1)	100.000	1.00.E-17
Q9NX58	Cell growth-regulating nucleolar protein	PNVSPK	HexNAc(4)Hex(4)NeuAc(1)	100.000	1.00.E-17
P42696	RNA-binding protein 34	LKNVSKPK	HexNAc(4)Hex(4)Fuc(2)NeuAc(1)	100.000	1.00.E-17
Q5IJ48	Protein crumbs homolog 2	EVLECASAPCEHNASCLEGLGSFR	HexNAc(4)Hex(4)Fuc(2)NeuAc(1)	100.000	1.00.E-17
Q9UHN6	Cell surface hyaluronidase	TLNSSGLPFGSYTFEK	HexNAc(4)Hex(4)Fuc(2)NeuAc(1)	2.837	3.54.E-02
O43657	Tetraspanin-6	QYNSTGDYR	HexNAc(4)Hex(4)Fuc(1)NeuAc(1)	100.000	1.00.E-17
P06756	Integrin alpha-V	NMTISR	HexNAc(4)Hex(4)Fuc(1)NeuAc(1)	100.000	1.00.E-17
P06756	Integrin alpha-V	TAADTTGLQPILNQFTPANISR	HexNAc(4)Hex(4)Fuc(1)NeuAc(1)	10.471	1.80.E-03
P08648	Integrin alpha-5	TEKEPLSDPVGTCYLSTDNFTR	HexNAc(4)Hex(4)Fuc(1)NeuAc(1)	100.000	1.00.E-17
P08962	CD63 antigen	NNHTASILDR	HexNAc(4)Hex(4)Fuc(1)NeuAc(1)	11.489	1.43.E-03
P11717	Cation-independent mannose-6-phosphate receptor	GYPCGGNK	HexNAc(4)Hex(4)Fuc(1)NeuAc(1)	3.718	3.73.E-02
P57087	Isoform 3 of Junctional adhesion molecule B	IKNVTR	HexNAc(4)Hex(4)Fuc(1)NeuAc(1)	100.000	1.00.E-17
P78504	Protein jagged-1	NCSHLK	HexNAc(4)Hex(4)Fuc(1)NeuAc(1)	100.000	1.00.E-17
Q07954	Prolow-density lipoprotein receptor-related protein 1	LTSCATNASICGDEAR	HexNAc(4)Hex(4)Fuc(1)NeuAc(1)	100.000	1.00.E-17
Q07954	Prolow-density lipoprotein receptor-related protein 1	IETILLNGTDRK	HexNAc(4)Hex(4)Fuc(1)NeuAc(1)	10.819	1.21.E-02
Q07954	Prolow-density lipoprotein receptor-related protein 1	MHLNGSNVQVLHR	HexNAc(4)Hex(4)Fuc(1)NeuAc(1)	4.332	1.68.E-02
Q4KMQ2	Isoform 2 of Anoctamin-6	NKSK	HexNAc(4)Hex(4)Fuc(1)NeuAc(1)	2.817	3.00.E-02
Q5ZPR3	CD276 antigen	QLVHSFAEGQDQGSAYANR	HexNAc(4)Hex(4)Fuc(1)NeuAc(1)	100.000	1.00.E-17
Q8IV08	Phospholipase D3	DNHTHSDIQVK	HexNAc(4)Hex(4)Fuc(1)NeuAc(1)	4.195	9.70.E-03
Q92896	Isoform 2 of Golgi apparatus protein 1	GNITEYQCHQYITK	HexNAc(4)Hex(4)Fuc(1)NeuAc(1)	4.919	1.09.E-02
Q99523	Sortilin	DITDLINNTFIR	HexNAc(4)Hex(4)Fuc(1)NeuAc(1)	3.292	3.70.E-02
O15031	Plexin-B2	TEAGAFEYVPDPTFENFTGGVK	HexNAc(4)Hex(4)Fuc(1)	100.000	1.00.E-17
O60637	Tetraspanin-3	NQSVPLSCCR	HexNAc(4)Hex(4)Fuc(1)	4.597	1.20.E-02
P06756	Integrin alpha-V	ANTTQPGIVEGGQVLK	HexNAc(4)Hex(4)Fuc(1)	7.202	4.64.E-03
P08962	CD63 antigen	NNHTASILDR	HexNAc(4)Hex(4)Fuc(1)	5.242	1.80.E-02
P13473	Isoform LAMP-2C of Lysosome-associated membrane glycoprotein 2	VASVININPNTTHSTGSCR	HexNAc(4)Hex(4)Fuc(1)	100.000	1.00.E-17
P19022	Cadherin-2	NLSLR	HexNAc(4)Hex(4)Fuc(1)	100.000	1.00.E-17
P19022	Cadherin-2	SNISILR	HexNAc(4)Hex(4)Fuc(1)	100.000	1.00.E-17
P41217	Isoform 3 of OX-2 membrane glycoprotein	FSEDHLNITCSATAR	HexNAc(4)Hex(4)Fuc(1)	3.563	2.71.E-02
P51654	Isoform 3 of Glypican-3	NYTNAMFK	HexNAc(4)Hex(4)Fuc(1)	100.000	1.00.E-17
P78310	Coxsackievirus and adenovirus receptor	NASSEYSGTYSCTVR	HexNAc(4)Hex(4)Fuc(1)	100.000	1.00.E-17
P98164	Low-density lipoprotein receptor-related protein 2	NLTNPR	HexNAc(4)Hex(4)Fuc(1)	100.000	1.00.E-17
Q01638	Interleukin-1 receptor-like 1	SPTFNR	HexNAc(4)Hex(4)Fuc(1)	7.263	3.02.E-03
Q07954	Prolow-density lipoprotein receptor-related protein 1	TCVSNCTASQFVCK	HexNAc(4)Hex(4)Fuc(1)	100.000	1.00.E-17
Q07954	Prolow-density lipoprotein receptor-related protein 1	DNATDSVPLR	HexNAc(4)Hex(4)Fuc(1)	100.000	1.00.E-17
Q07954	Prolow-density lipoprotein receptor-related protein 1	CNASSQFLCSSGR	HexNAc(4)Hex(4)Fuc(1)	100.000	1.00.E-17
Q07954	Prolow-density lipoprotein receptor-related protein 1	WTGHNVTVVQR	HexNAc(4)Hex(4)Fuc(1)	13.262	9.56.E-04
Q07954	Prolow-density lipoprotein receptor-related protein 1	AVNSSCR	HexNAc(4)Hex(4)Fuc(1)	10.210	2.24.E-03
Q07954	Prolow-density lipoprotein receptor-related protein 1	FGTCSQLCNNTK	HexNAc(4)Hex(4)Fuc(1)	7.176	5.14.E-03
Q07954	Prolow-density lipoprotein receptor-related protein 1	THANGSIK	HexNAc(4)Hex(4)Fuc(1)	5.549	9.70.E-03
Q08722	Leukocyte surface antigen CD47	SDAVSHTGNYTCEVTELTR	HexNAc(4)Hex(4)Fuc(1)	100.000	1.00.E-17
Q15223	Nectin-1	NPNGTVTVISR	HexNAc(4)Hex(4)Fuc(1)	100.000	1.00.E-17
Q7LGA3	Heparan sulfate 2-O-sulfotransferase 1	NITSWK	HexNAc(4)Hex(4)Fuc(1)	3.937	2.44.E-02
Q9NR09	Baculoviral IAP repeat-containing protein 6	IANATR	HexNAc(4)Hex(4)Fuc(1)	100.000	1.00.E-17
Q9UHN6	Cell surface hyaluronidase	TTNSSAADPR	HexNAc(4)Hex(4)Fuc(1)	100.000	1.00.E-17
P07996	Thrombospondin-1	VVNSTTGPGEHLR	HexNAc(4)Hex(4)	100.000	1.00.E-17
P49454	Centromere protein F	NLTVELEQK	HexNAc(4)Hex(4)	2.729	2.24.E-02
P51805	Plexin-A3	SLNR	HexNAc(4)Hex(4)	5.494	1.46.E-02
Q07954	Prolow-density lipoprotein receptor-related protein 1	FNSTEYQVVTR	HexNAc(4)Hex(4)	100.000	1.00.E-17
Q8NFC6	Biorientation of chromosomes in cell division protein 1-like 1	NKSTDK	HexNAc(4)Hex(4)	100.000	1.00.E-17
Q9NQZ3	Isoform 2 of Deleted in azoospermia protein 1	IITNR	HexNAc(4)Hex(4)	100.000	1.00.E-17
O43639	Cytoplasmic protein NCK2	NAANR	HexNAc(4)Hex(3)NeuAc(1)	100.000	1.00.E-17
P35580	Isoform 4 of Myosin-10	LNLSSR	HexNAc(4)Hex(3)NeuAc(1)	100.000	1.00.E-17
Q6UXK2	Immunoglobulin superfamily containing leucine-rich repeat protein 2	NLSALQLLK	HexNAc(4)Hex(3)NeuAc(1)	100.000	1.00.E-17
A8MVW0	Protein FAM171A2	NGTGVIR	HexNAc(4)Hex(3)Fuc(2)	100.000	1.00.E-17
O43852	Isoform 4 of Calumenin	NVTYGTYLDDPDPDDGFNYK	HexNAc(4)Hex(3)Fuc(1)	2.869	1.28.E-02
O43852	Isoform 3 of Calumenin	NATYGYVLDDPDPDDGFNYK	HexNAc(4)Hex(3)Fuc(1)	2.227	1.92.E-02

O60568	Procollagen-lysine,2-oxoglutarate 5-dioxygenase 3	EQYIHENYSR	HexNAc(4)Hex(3)Fuc(1)	100.000	1.00.E-17
O60637	Tetraspanin-3	NQSVPLSCCR	HexNAc(4)Hex(3)Fuc(1)	100.000	1.00.E-17
O75976	Carboxypeptidase D	NVTVK	HexNAc(4)Hex(3)Fuc(1)	100.000	1.00.E-17
O95714	E3 ubiquitin-protein ligase HERC2	LNLASNR	HexNAc(4)Hex(3)Fuc(1)	3.672	2.65.E-02
P08962	CD63 antigen	NNHTASILDR	HexNAc(4)Hex(3)Fuc(1)	5.053	6.80.E-03
P11717	Cation-independent mannose-6-phosphate receptor	SLLEFNTTVSCDQQGTNHR	HexNAc(4)Hex(3)Fuc(1)	8.567	2.44.E-03
P18077	60S ribosomal protein L35a	NNTVTPGGK	HexNAc(4)Hex(3)Fuc(1)	100.000	1.00.E-17
P32004	Neural cell adhesion molecule L1	VPGNQTSTTLK	HexNAc(4)Hex(3)Fuc(1)	9.077	1.14.E-03
Q06210	Glutamine--fructose-6-phosphate aminotransferase [isomerizing] 1	GSCNLSR	HexNAc(4)Hex(3)Fuc(1)	100.000	1.00.E-17
Q07954	Prolow-density lipoprotein receptor-related protein 1	FGTCSQLCNNTK	HexNAc(4)Hex(3)Fuc(1)	100.000	1.00.E-17
Q07954	Prolow-density lipoprotein receptor-related protein 1	WTGHNVTVVQR	HexNAc(4)Hex(3)Fuc(1)	3.198	3.81.E-02
Q15293	Reticulocalbin-1	VVRPDSELGERPPEDNQSFQYDHEAFLGK	HexNAc(4)Hex(3)Fuc(1)	2.594	1.39.E-02
Q15904	V-type proton ATPase subunit S1	LNASLPALLLIR	HexNAc(4)Hex(3)Fuc(1)	4.014	2.24.E-02
Q6ZXV5	Transmembrane and TPR repeat-containing protein 3	ANESRLEEADQLYR	HexNAc(4)Hex(3)Fuc(1)	3.851	2.27.E-02
Q70UQ0	Isoform 4 of Inhibitor of nuclear factor kappa-B kinase-interacting protein	FQNITDFWK	HexNAc(4)Hex(3)Fuc(1)	100.000	1.00.E-17
Q8N7P1	Inactive phospholipase D5	LFCPKNR	HexNAc(4)Hex(3)Fuc(1)	100.000	1.00.E-17
Q8NC56	LEM domain-containing protein 2	CIPVMEAQEYIANVTSSSSAK	HexNAc(4)Hex(3)Fuc(1)	6.689	6.84.E-03
Q96KA5	Cleft lip and palate transmembrane protein 1-like protein	TVNVSVPK	HexNAc(4)Hex(3)Fuc(1)	10.116	1.46.E-03
Q9C0H2	Isoform 4 of Protein tweety homolog 3	VWDTAVGLNHTAEPSLQTLER	HexNAc(4)Hex(3)Fuc(1)	100.000	1.00.E-17
Q9HDC9	Adipocyte plasma membrane-associated protein	AGPNGTLFVADAYK	HexNAc(4)Hex(3)Fuc(1)	3.982	3.32.E-03
Q9NYU2	UDP-glucose:glycoprotein glucosyltransferase 1	GTEVNTTVIGENDPIDEVQGFLFGK	HexNAc(4)Hex(3)Fuc(1)	2.816	2.83.E-02
P07996	Thrombospondin-1	VVNSTTGPGEHLR	HexNAc(4)Hex(3)	100.000	1.00.E-17
Q9HCU4	Cadherin EGF LAG seven-pass G-type receptor 2	NESGLDSGR	HexNAc(4)Hex(3)	100.000	1.00.E-17
Q9HD45	Transmembrane 9 superfamily member 3	IVDVNLTSEGK	HexNAc(4)Hex(3)	3.440	3.45.E-02
P09960	Isoform 4 of Leukotriene A-4 hydrolase	NLSKR	HexNAc(3)Hex(6)NeuAc(1)	100.000	1.00.E-17
P18084	Integrin beta-5	SNLTVLR	HexNAc(3)Hex(6)NeuAc(1)	100.000	1.00.E-17
P19022	Cadherin-2	SNISILR	HexNAc(3)Hex(6)NeuAc(1)	100.000	1.00.E-17
P23246	Splicing factor, proline- and glutamine-rich	ANLSLLR	HexNAc(3)Hex(6)NeuAc(1)	100.000	1.00.E-17
P53801	Pituitary tumor-transforming gene 1 protein-interacting protein	NVSCLWCNTNK	HexNAc(3)Hex(6)NeuAc(1)	100.000	1.00.E-17
P98164	Low-density lipoprotein receptor-related protein 2	MFLNK	HexNAc(3)Hex(6)NeuAc(1)	100.000	1.00.E-17
Q07954	Prolow-density lipoprotein receptor-related protein 1	TCVSNCTASQFVCK	HexNAc(3)Hex(6)NeuAc(1)	100.000	1.00.E-17
Q07954	Prolow-density lipoprotein receptor-related protein 1	DNTTCYEFK	HexNAc(3)Hex(6)NeuAc(1)	100.000	1.00.E-17
Q13308	Isoform 6 of Inactive tyrosine-protein kinase 7	NLTLR	HexNAc(3)Hex(6)NeuAc(1)	2.419	4.40.E-02
Q8N8S7	Protein enabled homolog	NQIVFDNR	HexNAc(3)Hex(6)NeuAc(1)	100.000	1.00.E-17
Q96K49	Transmembrane protein 87B	TMFNSTDIK	HexNAc(3)Hex(6)NeuAc(1)	4.316	1.81.E-02
Q96KM6	Zinc finger protein 512B	LNYTR	HexNAc(3)Hex(6)NeuAc(1)	100.000	1.00.E-17
O15031	Plexin-B2	TEAGAFEYVPDPTFENFTGGVK	HexNAc(3)Hex(6)Fuc(1)NeuAc(1)	100.000	1.00.E-17
O60486	Plexin-C1	TNVTVK	HexNAc(3)Hex(6)Fuc(1)NeuAc(1)	100.000	1.00.E-17
P04216	Thy-1 membrane glycoprotein	TNFTSK	HexNAc(3)Hex(6)Fuc(1)NeuAc(1)	100.000	1.00.E-17
P06756	Integrin alpha-V	ENQNHSYSLK	HexNAc(3)Hex(6)Fuc(1)NeuAc(1)	100.000	1.00.E-17
P19022	Cadherin-2	SNISILR	HexNAc(3)Hex(6)Fuc(1)NeuAc(1)	4.399	1.23.E-02
P20645	Cation-dependent mannose-6-phosphate receptor	EAGNHTSGAGLVQINK	HexNAc(3)Hex(6)Fuc(1)NeuAc(1)	100.000	1.00.E-17
P54709	Sodium/potassium-transporting ATPase subunit beta-3	NLTVCPDGALFEQK	HexNAc(3)Hex(6)Fuc(1)NeuAc(1)	100.000	1.00.E-17
Q07954	Prolow-density lipoprotein receptor-related protein 1	LHKDNTTCYEFK	HexNAc(3)Hex(6)Fuc(1)NeuAc(1)	100.000	1.00.E-17
Q5ZPR3	CD276 antigen	TALFPDLLAQGNASLR	HexNAc(3)Hex(6)Fuc(1)NeuAc(1)	100.000	1.00.E-17
Q8TCT7	Signal peptide peptidase-like 2B	LVPPGGNK	HexNAc(3)Hex(6)Fuc(1)NeuAc(1)	100.000	1.00.E-17
Q9UBG0	C-type mannose receptor 2	VTPACNTSLPAQR	HexNAc(3)Hex(6)Fuc(1)NeuAc(1)	100.000	1.00.E-17
A0MZ66	Isoform 3 of Shootin-1	LNKENK	HexNAc(3)Hex(6)Fuc(1)	100.000	1.00.E-17
A6NGN9	IgLON family member 5	HYGNYTCR	HexNAc(3)Hex(6)Fuc(1)	100.000	1.00.E-17
O15031	Plexin-B2	TEAGAFEYVPDPTFENFTGGVK	HexNAc(3)Hex(6)Fuc(1)	100.000	1.00.E-17
O60637	Tetraspanin-3	NQSVPLSCCR	HexNAc(3)Hex(6)Fuc(1)	6.081	3.47.E-03
O95297	Myelin protein zero-like protein 1	EIFVANGTQGK	HexNAc(3)Hex(6)Fuc(1)	100.000	1.00.E-17
P06756	Integrin alpha-V	ENQNHSYSLK	HexNAc(3)Hex(6)Fuc(1)	100.000	1.00.E-17
P06756	Integrin alpha-V	ANTTQPGIVEGGQVLK	HexNAc(3)Hex(6)Fuc(1)	24.169	4.03.E-05
P08962	CD63 antigen	NNHTASILDR	HexNAc(3)Hex(6)Fuc(1)	100.000	1.00.E-17
P11717	Cation-independent mannose-6-phosphate receptor	NGSSIVDLSPLIHR	HexNAc(3)Hex(6)Fuc(1)	100.000	1.00.E-17
P13473	Isoform LAMP-2C of Lysosome-associated membrane glycoprotein 2	VQPFNVTQGK	HexNAc(3)Hex(6)Fuc(1)	100.000	1.00.E-17
P18084	Integrin beta-5	SNLTVLR	HexNAc(3)Hex(6)Fuc(1)	100.000	1.00.E-17
P19022	Cadherin-2	SNISILR	HexNAc(3)Hex(6)Fuc(1)	10.539	6.34.E-04
P23229	Integrin alpha-6	YQTLNCSVNVNCVNIR	HexNAc(3)Hex(6)Fuc(1)	100.000	1.00.E-17
P23246	Splicing factor, proline- and glutamine-rich	ANLSLLR	HexNAc(3)Hex(6)Fuc(1)	8.429	2.73.E-03
P78310	Coxsackievirus and adenovirus receptor	NASSEYSGTYSCTVR	HexNAc(3)Hex(6)Fuc(1)	100.000	1.00.E-17
Q01973	Inactive tyrosine-protein kinase transmembrane receptor ROR1	FIGNR	HexNAc(3)Hex(6)Fuc(1)	100.000	1.00.E-17
Q07954	Prolow-density lipoprotein receptor-related protein 1	WTGHNVTVVQR	HexNAc(3)Hex(6)Fuc(1)	100.000	1.00.E-17
Q07954	Prolow-density lipoprotein receptor-related protein 1	TCVSNCTASQFVCK	HexNAc(3)Hex(6)Fuc(1)	100.000	1.00.E-17
Q07954	Prolow-density lipoprotein receptor-related protein 1	FGTCSQLCNNTK	HexNAc(3)Hex(6)Fuc(1)	4.884	1.07.E-02
Q08722	Leukocyte surface antigen CD47	DIYTFDGALNK	HexNAc(3)Hex(6)Fuc(1)	3.174	3.18.E-02
Q13308	Isoform 6 of Inactive tyrosine-protein kinase 7	NLTLR	HexNAc(3)Hex(6)Fuc(1)	100.000	1.00.E-17
Q96JN0	Isoform 3 of Ligand-dependent corepressor	PNLSSSPR	HexNAc(3)Hex(6)Fuc(1)	12.442	1.29.E-03
Q96QF7	Acidic repeat-containing protein	IYDLFNR	HexNAc(3)Hex(6)Fuc(1)	100.000	1.00.E-17
Q9HD45	Transmembrane 9 superfamily member 3	IVDVNLTSEGK	HexNAc(3)Hex(6)Fuc(1)	4.252	8.72.E-03
Q9P273	Teneurin-3	FNISLQK	HexNAc(3)Hex(6)Fuc(1)	100.000	1.00.E-17
O60637	Tetraspanin-3	NQSVPLSCCR	HexNAc(3)Hex(6)	100.000	1.00.E-17
P01130	Low-density lipoprotein receptor	ENGSKPR	HexNAc(3)Hex(6)	100.000	1.00.E-17
P06756	Integrin alpha-V	ANTTQPGIVEGGQVLK	HexNAc(3)Hex(6)	100.000	1.00.E-17
P06756	Integrin alpha-V	ENQNHSYSLK	HexNAc(3)Hex(6)	7.805	4.65.E-03
P08069	Insulin-like growth factor 1 receptor	LNPGNYTAR	HexNAc(3)Hex(6)	100.000	1.00.E-17
P11279	Lysosome-associated membrane glycoprotein 1	LLNINPNK	HexNAc(3)Hex(6)	2.893	4.59.E-02

P19022	Cadherin-2	SNISILR	HexNAc(3)Hex(6)	100.000	1.00.E-17
P23229	Integrin alpha-6	YQTLNCSVNVNCVNIR	HexNAc(3)Hex(6)	4.789	2.26.E-02
P23229	Integrin alpha-6	ANHSGAVVLLK	HexNAc(3)Hex(6)	4.232	1.84.E-02
Q07954	Prolow-density lipoprotein receptor-related protein 1	LNGTDPIVAADSK	HexNAc(3)Hex(6)	10.046	2.32.E-03
Q08722	Leukocyte surface antigen CD47	DIYTFDGALNK	HexNAc(3)Hex(6)	2.308	4.46.E-02
Q13308	Isoform 6 of Inactive tyrosine-protein kinase 7	NLTLR	HexNAc(3)Hex(6)	9.675	1.07.E-03
Q8N8S7	Protein enabled homolog	NQIVFDNR	HexNAc(3)Hex(6)	100.000	1.00.E-17
Q9NUN5	Probable lysosomal cobalamin transporter	NQNGTFK	HexNAc(3)Hex(6)	2.718	3.45.E-02
Q9P2K2	Thioredoxin domain-containing protein 16	HWNR	HexNAc(3)Hex(6)	100.000	1.00.E-17
Q9UBS9	Isoform 2 of SUN domain-containing ossification factor	SISENATATAAPK	HexNAc(3)Hex(6)	3.441	2.77.E-02
O14786	Neuropilin-1	RGPECSQNYTTPSGVIK	HexNAc(3)Hex(5)NeuAc(1)	100.000	1.00.E-17
O95297	Myelin protein zero-like protein 1	DASINIENMQFIHNGTYICDVK	HexNAc(3)Hex(5)NeuAc(1)	100.000	1.00.E-17
P06756	Integrin alpha-V	ENQNHSYSLK	HexNAc(3)Hex(5)NeuAc(1)	100.000	1.00.E-17
Q5UIP0	Telomere-associated protein RIF1	TVNGIENK	HexNAc(3)Hex(5)NeuAc(1)	100.000	1.00.E-17
Q8N6R0	Methyltransferase-like protein 13	ECNATR	HexNAc(3)Hex(5)NeuAc(1)	100.000	1.00.E-17
Q9NQZ3	Isoform 2 of Deleted in azoospermia protein 1	IITNR	HexNAc(3)Hex(5)NeuAc(1)	2.682	3.29.E-02
O60637	Tetraspanin-3	NQSVPLSCCR	HexNAc(3)Hex(5)Fuc(1)	3.127	1.54.E-02
P06756	Integrin alpha-V	ENQNHSYSLK	HexNAc(3)Hex(5)Fuc(1)	100.000	1.00.E-17
P06756	Integrin alpha-V	ANTTQPGIVEGGQVLK	HexNAc(3)Hex(5)Fuc(1)	60.561	3.96.E-08
P08069	Insulin-like growth factor 1 receptor	LNPGNYTAR	HexNAc(3)Hex(5)Fuc(1)	100.000	1.00.E-17
Q07954	Prolow-density lipoprotein receptor-related protein 1	WTGHNVTVVQR	HexNAc(3)Hex(5)Fuc(1)	6.920	8.97.E-03
Q9NQZ3	Isoform 2 of Deleted in azoospermia protein 1	IITNR	HexNAc(3)Hex(5)Fuc(1)	100.000	1.00.E-17
Q9Y4L1	Hypoxia up-regulated protein 1	AEPPLNASASDQGEK	HexNAc(3)Hex(5)Fuc(1)	100.000	1.00.E-17
O95819	Isoform 6 of Mitogen-activated protein kinase kinase kinase kinase 4	NSTSSIEPR	HexNAc(3)Hex(5)	100.000	1.00.E-17
P06756	Integrin alpha-V	ANTTQPGIVEGGQVLK	HexNAc(3)Hex(5)	17.367	1.23.E-04
P06756	Integrin alpha-V	ENQNHSYSLK	HexNAc(3)Hex(5)	7.660	3.64.E-03
P19022	Cadherin-2	SNISILR	HexNAc(3)Hex(5)	6.061	8.05.E-03
P23229	Integrin alpha-6	YQTLNCSVNVNCVNIR	HexNAc(3)Hex(5)	100.000	1.00.E-17
Q07954	Prolow-density lipoprotein receptor-related protein 1	WTGHNVTVVQR	HexNAc(3)Hex(5)	100.000	1.00.E-17
Q07954	Prolow-density lipoprotein receptor-related protein 1	LNGTDPIVAADSK	HexNAc(3)Hex(5)	6.704	3.59.E-03
Q08722	Leukocyte surface antigen CD47	GRDIYTFDGALNK	HexNAc(3)Hex(5)	100.000	1.00.E-17
Q13308	Isoform 6 of Inactive tyrosine-protein kinase 7	NLTLR	HexNAc(3)Hex(5)	100.000	1.00.E-17
Q8WY21	Isoform 2 of VPS10 domain-containing receptor SorCS1	VVSNNCTDGVR	HexNAc(3)Hex(5)	100.000	1.00.E-17
Q9HD45	Transmembrane 9 superfamily member 3	IVDVNLTSEGK	HexNAc(3)Hex(5)	100.000	1.00.E-17
Q9NQZ3	Isoform 2 of Deleted in azoospermia protein 1	IITNR	HexNAc(3)Hex(5)	7.100	4.63.E-03
Q9UBG0	C-type mannose receptor 2	GTDPSLSPSPAALPPAPGTELSYLNGTFR	HexNAc(3)Hex(5)	100.000	1.00.E-17
O14786	Neuropilin-1	RGPECSQNYTTPSGVIK	HexNAc(3)Hex(4)NeuAc(1)	100.000	1.00.E-17
Q03164	Isoform 3 of Histone-lysine N-methyltransferase 2A	NGKENGTENLK	HexNAc(3)Hex(4)NeuAc(1)	2.406	4.00.E-02
P19022	Cadherin-2	SNISILR	HexNAc(3)Hex(4)Fuc(2)	100.000	1.00.E-17
P20645	Cation-dependent mannose-6-phosphate receptor	EAGNHTSGAGLVQINK	HexNAc(3)Hex(4)Fuc(2)	100.000	1.00.E-17
O14976	Cyclin-G-associated kinase	NTTPMYR	HexNAc(3)Hex(4)Fuc(1)NeuAc(1)	4.069	1.97.E-02
O15031	Plexin-B2	TEAGAFEYVPDPTFENFTGGVK	HexNAc(3)Hex(4)Fuc(1)NeuAc(1)	100.000	1.00.E-17
P11279	Lysosome-associated membrane glycoprotein 1	SSCGKENTSDPSLVIAFGR	HexNAc(3)Hex(4)Fuc(1)NeuAc(1)	2.322	4.70.E-02
P11717	Cation-independent mannose-6-phosphate receptor	GYPCGGNK	HexNAc(3)Hex(4)Fuc(1)NeuAc(1)	100.000	1.00.E-17
P11717	Cation-independent mannose-6-phosphate receptor	DAGVGFPEYQEEDNSTYNFR	HexNAc(3)Hex(4)Fuc(1)NeuAc(1)	2.444	2.99.E-02
P49790	Isoform 3 of Nuclear pore complex protein Nup153	PVSIATNR	HexNAc(3)Hex(4)Fuc(1)NeuAc(1)	100.000	1.00.E-17
P53634	Dipeptidyl peptidase 1	DVNCSVMGPQEK	HexNAc(3)Hex(4)Fuc(1)NeuAc(1)	100.000	1.00.E-17
Q07954	Prolow-density lipoprotein receptor-related protein 1	TCVSNCTASQFVCK	HexNAc(3)Hex(4)Fuc(1)NeuAc(1)	100.000	1.00.E-17
Q8IV08	Phospholipase D3	DNHTHSDIQVK	HexNAc(3)Hex(4)Fuc(1)NeuAc(1)	42.746	2.65.E-06
Q8IV08	Phospholipase D3	ELGVVMYNCSCLAR	HexNAc(3)Hex(4)Fuc(1)NeuAc(1)	3.800	1.72.E-02
Q8N8S7	Protein enabled homolog	NQIVFDNR	HexNAc(3)Hex(4)Fuc(1)NeuAc(1)	100.000	1.00.E-17
Q92673	Sortilin-related receptor	CPNGTCIPSSK	HexNAc(3)Hex(4)Fuc(1)NeuAc(1)	100.000	1.00.E-17
Q9UBG0	C-type mannose receptor 2	VTPACNTSLPAQR	HexNAc(3)Hex(4)Fuc(1)NeuAc(1)	100.000	1.00.E-17
Q9Y6M7	Isoform 7 of Sodium bicarbonate cotransporter 3	NLTVSECK	HexNAc(3)Hex(4)Fuc(1)NeuAc(1)	100.000	1.00.E-17
O60637	Tetraspanin-3	NQSVPLSCCR	HexNAc(3)Hex(4)Fuc(1)	2.333	2.90.E-02
P05556	Isoform 5 of Integrin beta-1	LRNPCTSEQNCTSPFSYK	HexNAc(3)Hex(4)Fuc(1)	100.000	1.00.E-17
P06756	Integrin alpha-V	ENQNHSYSLK	HexNAc(3)Hex(4)Fuc(1)	100.000	1.00.E-17
P06756	Integrin alpha-V	ANTTQPGIVEGGQVLK	HexNAc(3)Hex(4)Fuc(1)	27.399	2.04.E-05
P19022	Cadherin-2	SNISILR	HexNAc(3)Hex(4)Fuc(1)	100.000	1.00.E-17
Q07954	Prolow-density lipoprotein receptor-related protein 1	WTGHNVTVVQR	HexNAc(3)Hex(4)Fuc(1)	100.000	1.00.E-17
Q07954	Prolow-density lipoprotein receptor-related protein 1	FGTCSQLCNNTK	HexNAc(3)Hex(4)Fuc(1)	7.914	3.13.E-03
Q08722	Leukocyte surface antigen CD47	SDAVSHTGNYTCEVTELTR	HexNAc(3)Hex(4)Fuc(1)	4.471	1.59.E-02
Q8N8S7	Protein enabled homolog	NQIVFDNR	HexNAc(3)Hex(4)Fuc(1)	4.110	1.08.E-02
Q96KA5	Cleft lip and palate transmembrane protein 1-like protein	TVNVSVPK	HexNAc(3)Hex(4)Fuc(1)	100.000	1.00.E-17
Q9UBG0	C-type mannose receptor 2	VTPACNTSLPAQR	HexNAc(3)Hex(4)Fuc(1)	100.000	1.00.E-17
P06756	Integrin alpha-V	ENQNHSYSLK	HexNAc(3)Hex(4)	100.000	1.00.E-17
P50851	Lipopolysaccharide-responsive and beige-like anchor protein	ATNLTR	HexNAc(3)Hex(4)	100.000	1.00.E-17
Q07954	Prolow-density lipoprotein receptor-related protein 1	LNGTDPIVAADSK	HexNAc(3)Hex(4)	3.191	1.67.E-02
O75970	Multiple PDZ domain protein	NGTLK	HexNAc(3)Hex(3)Fuc(2)	100.000	1.00.E-17
Q5T5Y3	Isoform 3 of Calmodulin-regulated spectrin-associated protein 1	LTGTGPKNITK	HexNAc(3)Hex(3)Fuc(2)	100.000	1.00.E-17
O43852	Isoform 3 of Calumenin	NATYGYVLDDPDPDDGFNYK	HexNAc(3)Hex(3)Fuc(1)	2.842	1.00.E-02
O60637	Tetraspanin-3	NQSVPLSCCR	HexNAc(3)Hex(3)Fuc(1)	5.252	1.48.E-03
P08962	CD63 antigen	NNHTASILDR	HexNAc(3)Hex(3)Fuc(1)	100.000	1.00.E-17
P14625	Endoplasmin	TDDEVVQREEEAIQLDGLNASQIR	HexNAc(3)Hex(3)Fuc(1)	100.000	1.00.E-17
P50454	Serpin H1	NVTWK	HexNAc(3)Hex(3)Fuc(1)	100.000	1.00.E-17
Q06210	Glutamine--fructose-6-phosphate aminotransferase [isomerizing] 1	GSCNLSR	HexNAc(3)Hex(3)Fuc(1)	100.000	1.00.E-17
Q12797	Aspartyl/asparaginyl beta-hydroxylase	LVQLFPNDTSLK	HexNAc(3)Hex(3)Fuc(1)	2.831	4.94.E-02
Q15155	Nodal modulator 1	LENITTGTYTIHAQK	HexNAc(3)Hex(3)Fuc(1)	2.904	3.10.E-02

Q15293	Reticulocalbin-1	VVRPDSELGERPPEDNQSFQYDHEAFLGK	HexNAc(3)Hex(3)Fuc(1)	2.680	3.66.E-02
Q70UQ0	Inhibitor of nuclear factor kappa-B kinase-interacting protein	QDILNNSLTTLSQDITK	HexNAc(3)Hex(3)Fuc(1)	100.000	1.00.E-17
Q70UQ0	Isoform 4 of Inhibitor of nuclear factor kappa-B kinase-interacting protein	FQNITDFWK	HexNAc(3)Hex(3)Fuc(1)	5.538	4.44.E-03
Q92733	Proline-rich protein PRCC	NLTVK	HexNAc(3)Hex(3)Fuc(1)	100.000	1.00.E-17
Q96AQ6	Pre-B-cell leukemia transcription factor-interacting protein 1	LQGLENWGQDPGVSANASK	HexNAc(3)Hex(3)Fuc(1)	6.302	3.13.E-02
Q96AY3	Peptidyl-prolyl cis-trans isomerase FKBP10	YHYNGTFEDGK	HexNAc(3)Hex(3)Fuc(1)	3.157	3.65.E-02
Q96KA5	Cleft lip and palate transmembrane protein 1-like protein	TVNVSVPK	HexNAc(3)Hex(3)Fuc(1)	3.555	3.94.E-02
Q96S52	GPI transamidase component PIG-S	TYNASVLPVR	HexNAc(3)Hex(3)Fuc(1)	10.951	1.00.E-02
Q9C0A0	Contactin-associated protein-like 4	NGSLQIR	HexNAc(3)Hex(3)Fuc(1)	3.662	3.13.E-02
Q9HDC9	Adipocyte plasma membrane-associated protein	AGPNGTLFVADAYK	HexNAc(3)Hex(3)Fuc(1)	3.259	6.82.E-03
Q9NYU2	UDP-glucose:glycoprotein glucosyltransferase 1	GTEVNTTVIGENDPIDEVQGFLFGK	HexNAc(3)Hex(3)Fuc(1)	100.000	1.00.E-17
Q9UPQ9	Trinucleotide repeat-containing gene 6B protein	SPAWNETGR	HexNAc(3)Hex(3)Fuc(1)	100.000	1.00.E-17
Q9UQ35	Serine/arginine repetitive matrix protein 2	TPPVALNSSR	HexNAc(3)Hex(3)Fuc(1)	3.935	2.36.E-02
Q9Y4L1	Hypoxia up-regulated protein 1	LGNTISSLFGGGTTPDAKENGTDTVQEEEESPAEGSK	HexNAc(3)Hex(3)Fuc(1)	4.166	8.22.E-03
O60568	Procollagen-lysine,2-oxoglutarate 5-dioxygenase 3	EQYIHENYSR	HexNAc(3)Hex(3)	100.000	1.00.E-17
Q08AD1	Calmodulin-regulated spectrin-associated protein 2	LNHTDGK	HexNAc(3)Hex(3)	100.000	1.00.E-17
Q16288	NT-3 growth factor receptor	NITSIHIENWR	HexNAc(3)Hex(3)	2.903	4.07.E-02
Q9BWH6	RNA polymerase II-associated protein 1	PGGPSANVTK	HexNAc(3)Hex(3)	100.000	1.00.E-17
O00468	Agrin	NELMLNSSLMR	HexNAc(2)Hex(9)	3.655	2.14.E-02
O15031	Plexin-B2	EAESLQPMTVVGTDYVFHNDTK	HexNAc(2)Hex(9)	2.040	4.24.E-02
O15230	Laminin subunit alpha-5	LVGGPVAGGDPNQTIR	HexNAc(2)Hex(9)	3.094	2.92.E-02
O60486	Plexin-C1	NGTEVVSCHPQGSTAGVVYR	HexNAc(2)Hex(9)	100.000	1.00.E-17
O75718	Cartilage-associated protein	NCSAAPQPEPAAGLASYPELR	HexNAc(2)Hex(9)	3.134	1.01.E-02
O75976	Carboxypeptidase D	NVTVK	HexNAc(2)Hex(9)	100.000	1.00.E-17
O95970	Isoform 2 of Leucine-rich glioma-inactivated protein 1	NYDNITVLR	HexNAc(2)Hex(9)	100.000	1.00.E-17
P02458	Collagen alpha-1(II) chain	LLSTEGSQNITYHCK	HexNAc(2)Hex(9)	21.177	1.25.E-04
P05156	Complement factor I	FLNNGTCTAEGK	HexNAc(2)Hex(9)	100.000	1.00.E-17
P05156	Complement factor I	LISNCSK	HexNAc(2)Hex(9)	100.000	1.00.E-17
P05997	Collagen alpha-2(V) chain	EASQNITYICK	HexNAc(2)Hex(9)	9.063	1.39.E-03
P06756	Integrin alpha-V	TAADTTGLQPILNQFTPANISR	HexNAc(2)Hex(9)	4.407	2.76.E-03
P06756	Integrin alpha-V	ENQNHSYSLK	HexNAc(2)Hex(9)	3.076	2.12.E-02
P07942	Laminin subunit beta-1	CVCNYLGTVQEHCNGSDCQCDK	HexNAc(2)Hex(9)	100.000	1.00.E-17
P07996	Thrombospondin-1	VSCPIMPCSNATVPDGECCPR	HexNAc(2)Hex(9)	100.000	1.00.E-17
P07996	Thrombospondin-1	VVNSTTGPGEHLR	HexNAc(2)Hex(9)	3.493	1.84.E-02
P10586	Receptor-type tyrosine-protein phosphatase F	VEVEPLNSTAVHVYWK	HexNAc(2)Hex(9)	4.315	7.85.E-03
P10909	Isoform 2 of Clusterin	HNSTGCLR	HexNAc(2)Hex(9)	9.401	2.86.E-03
P11047	Laminin subunit gamma-1	KIPAINQTITEANEK	HexNAc(2)Hex(9)	3.208	4.61.E-02
P11047	Laminin subunit gamma-1	LLNNLTSIK	HexNAc(2)Hex(9)	2.635	2.25.E-02
P11532	Isoform 2 of Dystrophin	AQNVTR	HexNAc(2)Hex(9)	2.822	3.99.E-02
P11717	Cation-independent mannose-6-phosphate receptor	NGSSIVDLSPLIHR	HexNAc(2)Hex(9)	100.000	1.00.E-17
P12107	Isoform B of Collagen alpha-1(XI) chain	VYCNFTSGGETCIYPDKK	HexNAc(2)Hex(9)	100.000	1.00.E-17
P12109	Collagen alpha-1(VI) chain	GAPGINGTK	HexNAc(2)Hex(9)	100.000	1.00.E-17
P13674	Prolyl 4-hydroxylase subunit alpha-1	DMSDGFISNLTIQR	HexNAc(2)Hex(9)	4.170	7.11.E-03
P16870	Carboxypeptidase E	GNETIVNLIHSTR	HexNAc(2)Hex(9)	4.394	1.51.E-02
P19022	Cadherin-2	SNISILR	HexNAc(2)Hex(9)	7.362	2.80.E-03
P19022	Cadherin-2	NWTITR	HexNAc(2)Hex(9)	4.793	9.64.E-03
P20645	Cation-dependent mannose-6-phosphate receptor	EAGNHTSGAGLVQINK	HexNAc(2)Hex(9)	100.000	1.00.E-17
P20908	Collagen alpha-1(V) chain	VYCNFTAGGSTCVFPDKK	HexNAc(2)Hex(9)	100.000	1.00.E-17
P25391	Laminin subunit alpha-1	YNNGTWYK	HexNAc(2)Hex(9)	100.000	1.00.E-17
P25391	Laminin subunit alpha-1	HQVSINNTAVMQR	HexNAc(2)Hex(9)	4.290	1.72.E-02
P25391	Laminin subunit alpha-1	DFTQLHQNATLELK	HexNAc(2)Hex(9)	2.782	3.84.E-02
P35556	Fibrillin-2	FNLSHLGSK	HexNAc(2)Hex(9)	3.621	1.84.E-02
P41218	Myeloid cell nuclear differentiation antigen	IIEIANK	HexNAc(2)Hex(9)	100.000	1.00.E-17
P42261	Isoform 5 of Glutamate receptor 1	TNYTLHVIEMK	HexNAc(2)Hex(9)	100.000	1.00.E-17
P49746	Thrombospondin-3	CNDTVPEDFEPFRR	HexNAc(2)Hex(9)	100.000	1.00.E-17
P51689	Arylsulfatase D	ALQWNAGSGGLPENETTFAR	HexNAc(2)Hex(9)	100.000	1.00.E-17
P56199	Integrin alpha-1	DSCESNHNITCK	HexNAc(2)Hex(9)	3.795	4.14.E-02
P56199	Integrin alpha-1	SENASLVLSSSNQK	HexNAc(2)Hex(9)	3.098	1.96.E-02
P56199	Integrin alpha-1	YNHTGQVIIYR	HexNAc(2)Hex(9)	2.306	4.27.E-02
P56706	Protein Wnt-7b	WNCSALGEK	HexNAc(2)Hex(9)	100.000	1.00.E-17
P61812	Isoform B of Transforming growth factor beta-2	NASNLVK	HexNAc(2)Hex(9)	100.000	1.00.E-17
P78357	Contactin-associated protein 1	VDGQLVNLTLVEGR	HexNAc(2)Hex(9)	2.114	3.71.E-02
P98164	Low-density lipoprotein receptor-related protein 2	MFLNK	HexNAc(2)Hex(9)	6.661	5.18.E-03
P98164	Low-density lipoprotein receptor-related protein 2	NLTNPR	HexNAc(2)Hex(9)	4.918	1.23.E-02
P98164	Low-density lipoprotein receptor-related protein 2	INTTYR	HexNAc(2)Hex(9)	3.287	3.71.E-02
P98164	Low-density lipoprotein receptor-related protein 2	VGMDGTNK	HexNAc(2)Hex(9)	3.052	2.83.E-02
Q02487	Desmocollin-2	NVTLHVPSK	HexNAc(2)Hex(9)	100.000	1.00.E-17
Q13201	Multimerin-1	MYQMFNETTSQVR	HexNAc(2)Hex(9)	100.000	1.00.E-17
Q13201	Multimerin-1	LNQSNFQK	HexNAc(2)Hex(9)	100.000	1.00.E-17
Q13201	Multimerin-1	LNDSIQTLVNDNQR	HexNAc(2)Hex(9)	100.000	1.00.E-17
Q13201	Multimerin-1	IDNISLTVNDVR	HexNAc(2)Hex(9)	100.000	1.00.E-17
Q13201	Multimerin-1	HPFTGDNCTIK	HexNAc(2)Hex(9)	100.000	1.00.E-17
Q13201	Multimerin-1	YQQNMSHLEEK	HexNAc(2)Hex(9)	3.002	2.80.E-02
Q13349	Integrin alpha-D	VNNLSQR	HexNAc(2)Hex(9)	4.581	1.94.E-02
Q13683	Integrin alpha-7	ANITVK	HexNAc(2)Hex(9)	3.380	2.51.E-02
Q13683	Integrin alpha-7	KNITLDCAR	HexNAc(2)Hex(9)	3.333	3.05.E-02
Q13683	Integrin alpha-7	LWNSTFLEEYSAVK	HexNAc(2)Hex(9)	2.953	2.75.E-02
Q13683	Integrin alpha-7	NITLDCAR	HexNAc(2)Hex(9)	2.397	3.53.E-02
Q14108	Lysosome membrane protein 2	CNMINGTDGDSFHPLITK	HexNAc(2)Hex(9)	100.000	1.00.E-17

Q14108	Lysosome membrane protein 2	TMVFPVMYLNESVHIDK	HexNAc(2)Hex(9)	4.308	1.58.E-02
Q14517	Protocadherin Fat 1	QVTQEMLNHTIAIR	HexNAc(2)Hex(9)	100.000	1.00.E-17
Q14832	Metabotropic glutamate receptor 3	TLCPNTTK	HexNAc(2)Hex(9)	100.000	1.00.E-17
Q15165	Serum paraoxonase/arylesterase 2	HTNMNLTQLK	HexNAc(2)Hex(9)	2.362	2.27.E-02
Q5IJ48	Protein crumbs homolog 2	NGSLAGGVR	HexNAc(2)Hex(9)	5.067	5.35.E-03
Q5IJ48	Protein crumbs homolog 2	EGPPAAFSGHNASSGR	HexNAc(2)Hex(9)	4.902	6.00.E-03
Q5IJ48	Protein crumbs homolog 2	LDGCHLPFFPLPLDNSSQPSELGGR	HexNAc(2)Hex(9)	4.039	8.01.E-03
Q5T1H1	Protein eyes shut homolog	LNVTVK	HexNAc(2)Hex(9)	100.000	1.00.E-17
Q68DQ2	Very large A-kinase anchor protein	FELNR	HexNAc(2)Hex(9)	100.000	1.00.E-17
Q6UW63	KDEL motif-containing protein 1	SNLSDLLEK	HexNAc(2)Hex(9)	100.000	1.00.E-17
Q6V0I7	Protocadherin Fat 4	LNITAK	HexNAc(2)Hex(9)	100.000	1.00.E-17
Q8IV08	Phospholipase D3	ELGVVMYNCSCLAR	HexNAc(2)Hex(9)	100.000	1.00.E-17
Q8IWU5	Extracellular sulfatase Sulf-2	YLNEYNGSYVPPGWK	HexNAc(2)Hex(9)	100.000	1.00.E-17
Q8IWU5	Extracellular sulfatase Sulf-2	FYNYTLCR	HexNAc(2)Hex(9)	100.000	1.00.E-17
Q8IWU6	Extracellular sulfatase Sulf-1	FYNYTVCR	HexNAc(2)Hex(9)	100.000	1.00.E-17
Q8IWU6	Extracellular sulfatase Sulf-1	YLNEYNGSYIPPGWR	HexNAc(2)Hex(9)	8.265	1.41.E-03
Q8IX30	Isoform 2 of Signal peptide, CUB and EGF-like domain-containing protein 3	CPGNTSTDFDGSTSVAQCK	HexNAc(2)Hex(9)	100.000	1.00.E-17
Q8IYK4	Procollagen galactosyltransferase 2	ALNTSQLK	HexNAc(2)Hex(9)	25.849	4.42.E-05
Q8IYK4	Procollagen galactosyltransferase 2	MAIWAATDHNVDNTTEIFR	HexNAc(2)Hex(9)	11.553	4.45.E-04
Q8NFM7	Interleukin-17 receptor D	QLNSSFK	HexNAc(2)Hex(9)	100.000	1.00.E-17
Q92791	Endoplasmic reticulum protein SC65	DSEAFCHANCSGPAPAAKPDPDGGR	HexNAc(2)Hex(9)	21.500	2.87.E-04
Q92820	Gamma-glutamyl hydrolase	NFTMNEK	HexNAc(2)Hex(9)	4.615	4.31.E-02
Q96GX1	Tectonic-2	WNNNTISEINVK	HexNAc(2)Hex(9)	2.055	4.79.E-02
Q96MM7	Isoform 4 of Heparan-sulfate 6-O-sulfotransferase 2	YNFTR	HexNAc(2)Hex(9)	100.000	1.00.E-17
Q96PQ0	VPS10 domain-containing receptor SorCS2	YVTCAIHNCSEK	HexNAc(2)Hex(9)	100.000	1.00.E-17
Q99985	Semaphorin-3C	VIQTFNR	HexNAc(2)Hex(9)	100.000	1.00.E-17
Q9BXX0	EMILIN-2	SLNDTMHR	HexNAc(2)Hex(9)	3.122	4.54.E-02
Q9BZC7	Isoform 3 of ATP-binding cassette sub-family A member 2	IRQNSSFTEK	HexNAc(2)Hex(9)	100.000	1.00.E-17
Q9BZC7	Isoform 3 of ATP-binding cassette sub-family A member 2	GNPAAYGITVTNHPMNK	HexNAc(2)Hex(9)	100.000	1.00.E-17
Q9H1J7	Protein Wnt-5b	WNCSTADNASVFGR	HexNAc(2)Hex(9)	100.000	1.00.E-17
Q9H6L2	Isoform 2 of Transmembrane protein 231	NVTTVLNDPNPIWLVGR	HexNAc(2)Hex(9)	3.369	2.01.E-02
Q9HAR2	Isoform 4 of Adhesion G protein-coupled receptor L3	LLTTNK	HexNAc(2)Hex(9)	10.067	6.34.E-04
Q9HCU4	Cadherin EGF LAG seven-pass G-type receptor 2	LYLNR	HexNAc(2)Hex(9)	3.032	3.92.E-02
Q9NXF1	Testis-expressed protein 10	NITTLK	HexNAc(2)Hex(9)	100.000	1.00.E-17
Q9NYU1	UDP-glucose:glycoprotein glucosyltransferase 2	DNLTAFQK	HexNAc(2)Hex(9)	2.814	3.88.E-02
Q9P273	Teneurin-3	LTNVTFPTGVVTNLHGDMDK	HexNAc(2)Hex(9)	3.538	1.34.E-02
Q9P2C4	Transmembrane protein 181	GMNFTWK	HexNAc(2)Hex(9)	100.000	1.00.E-17
Q9UBG0	C-type mannose receptor 2	VTPACNTSLPAQR	HexNAc(2)Hex(9)	2.678	3.46.E-02
Q9UHG3	Prenylcysteine oxidase 1	LLHALGGDDFLGMLNR	HexNAc(2)Hex(9)	100.000	1.00.E-17
Q9UHG3	Prenylcysteine oxidase 1	GELNTSIFSSR	HexNAc(2)Hex(9)	3.809	1.20.E-02
Q9UHN6	Cell surface hyaluronidase	TLNSSGLPFGSYTFEK	HexNAc(2)Hex(9)	2.070	4.58.E-02
Q9Y6M7	Isoform 7 of Sodium bicarbonate cotransporter 3	NLTVSECK	HexNAc(2)Hex(9)	2.320	3.63.E-02
A6NE02	BTB/POZ domain-containing protein 17	QGNASDVVLR	HexNAc(2)Hex(8)	100.000	1.00.E-17
O00469	Procollagen-lysine,2-oxoglutarate 5-dioxygenase 2	YFNYTVK	HexNAc(2)Hex(8)	100.000	1.00.E-17
O00469	Procollagen-lysine,2-oxoglutarate 5-dioxygenase 2	YNCSIESPR	HexNAc(2)Hex(8)	3.192	2.34.E-02
O14525	Astrotactin-1	AAPIYELVTNNQTQR	HexNAc(2)Hex(8)	100.000	1.00.E-17
O15031	Plexin-B2	EAESLQPMTVVGTDYVFHNDTK	HexNAc(2)Hex(8)	100.000	1.00.E-17
O15230	Laminin subunit alpha-5	LNASIADLQSQLR	HexNAc(2)Hex(8)	2.598	2.63.E-02
O60486	Plexin-C1	TASTIANSSK	HexNAc(2)Hex(8)	100.000	1.00.E-17
O60568	Procollagen-lysine,2-oxoglutarate 5-dioxygenase 3	SAEFFNYTVR	HexNAc(2)Hex(8)	2.764	4.22.E-02
O75051	Plexin-A2	EHYLSSVNK	HexNAc(2)Hex(8)	100.000	1.00.E-17
O75054	Isoform 2 of Immunoglobulin superfamily member 3	TLTLVENKPIQLNCSVK	HexNAc(2)Hex(8)	2.428	3.07.E-02
O94813	Slit homolog 2 protein	LDLNGNNITR	HexNAc(2)Hex(8)	100.000	1.00.E-17
O94856	Neurofascin	VILYNR	HexNAc(2)Hex(8)	100.000	1.00.E-17
O95970	Isoform 2 of Leucine-rich glioma-inactivated protein 1	NYDNITVLR	HexNAc(2)Hex(8)	100.000	1.00.E-17
P00450	Ceruloplasmin	EHEGAIYPDNTTDFQR	HexNAc(2)Hex(8)	100.000	1.00.E-17
P01137	Transforming growth factor beta-1	YSNNSWR	HexNAc(2)Hex(8)	9.864	2.24.E-03
P01137	Transforming growth factor beta-1	LASPPSQGEVPPGPLPEAVLALYNSTR	HexNAc(2)Hex(8)	6.573	9.38.E-03
P02458	Collagen alpha-1(II) chain	LLSTEGSQNITYHCK	HexNAc(2)Hex(8)	8.865	1.46.E-03
P05156	Complement factor I	LISNCSK	HexNAc(2)Hex(8)	100.000	1.00.E-17
P06756	Integrin alpha-V	NDTVAGQGERDHLITK	HexNAc(2)Hex(8)	100.000	1.00.E-17
P06756	Integrin alpha-V	ANTTQPGIVEGGQVLK	HexNAc(2)Hex(8)	11.863	2.57.E-04
P06756	Integrin alpha-V	TAADTTGLQPILNQFTPANISR	HexNAc(2)Hex(8)	4.966	4.02.E-03
P06756	Integrin alpha-V	NMTISR	HexNAc(2)Hex(8)	4.905	1.07.E-02
P07996	Thrombospondin-1	VSCPIMPCSNATVPDGECCPR	HexNAc(2)Hex(8)	100.000	1.00.E-17
P07996	Thrombospondin-1	VVNSTTGPGEHLR	HexNAc(2)Hex(8)	10.154	9.32.E-04
P08069	Insulin-like growth factor 1 receptor	LNPGNYTAR	HexNAc(2)Hex(8)	2.807	4.04.E-02
P10909	Isoform 2 of Clusterin	HNSTGCLR	HexNAc(2)Hex(8)	18.992	2.10.E-04
P11047	Laminin subunit gamma-1	CDQCEENYFYNR	HexNAc(2)Hex(8)	6.610	7.08.E-03
P12107	Isoform B of Collagen alpha-1(XI) chain	VYCNFTSGGETCIYPDKK	HexNAc(2)Hex(8)	21.586	1.16.E-03
P12109	Collagen alpha-1(VI) chain	GAPGINGTK	HexNAc(2)Hex(8)	100.000	1.00.E-17
P12109	Collagen alpha-1(VI) chain	NFTAADWGQSR	HexNAc(2)Hex(8)	3.028	1.20.E-02
P13674	Prolyl 4-hydroxylase subunit alpha-1	DMSDGFISNLTIQR	HexNAc(2)Hex(8)	3.540	6.63.E-03
P16278	Beta-galactosidase	NNVITLNITGK	HexNAc(2)Hex(8)	100.000	1.00.E-17
P17301	Integrin alpha-2	GEYFVNVTTR	HexNAc(2)Hex(8)	3.242	2.32.E-02
P18084	Integrin beta-5	CHAGYIGDNCNCSTDISTCR	HexNAc(2)Hex(8)	3.380	2.07.E-02
P19022	Cadherin-2	SNISILR	HexNAc(2)Hex(8)	7.992	8.55.E-04
P19022	Cadherin-2	NWTITR	HexNAc(2)Hex(8)	3.646	5.05.E-03
P19022	Cadherin-2	NLSLR	HexNAc(2)Hex(8)	3.428	3.26.E-02
P23246	Splicing factor, proline- and glutamine-rich	ANLSLLR	HexNAc(2)Hex(8)	100.000	1.00.E-17

P25391	Laminin subunit alpha-1	HQVSINNTAVMQR	HexNAc(2)Hex(8)	100.000	1.00.E-17
P25391	Laminin subunit alpha-1	IGNITHSIPACIGDVTVNSK	HexNAc(2)Hex(8)	6.165	1.13.E-02
P26006	Isoform 2 of Integrin alpha-3	SLDAYPILNQAQALENHTEVQFQK	HexNAc(2)Hex(8)	2.609	4.56.E-02
P26012	Integrin beta-8	NYAIIKPIGFNETAK	HexNAc(2)Hex(8)	8.201	5.56.E-04
P35556	Fibrillin-2	ASQDQTMCMDVDECERHPCGNGTCK	HexNAc(2)Hex(8)	100.000	1.00.E-17
P35556	Fibrillin-2	FNLSHLGSK	HexNAc(2)Hex(8)	5.094	6.46.E-03
P48307	Tissue factor pathway inhibitor 2	DEGLCSANVTR	HexNAc(2)Hex(8)	100.000	1.00.E-17
P56199	Integrin alpha-1	NTTFNVESTK	HexNAc(2)Hex(8)	8.064	1.18.E-02
P56199	Integrin alpha-1	NTTFNVESTKK	HexNAc(2)Hex(8)	4.953	6.90.E-03
P56199	Integrin alpha-1	SENASLVLSSSNQK	HexNAc(2)Hex(8)	4.667	8.09.E-03
P56199	Integrin alpha-1	YNHTGQVIIYR	HexNAc(2)Hex(8)	3.850	3.48.E-03
P61812	Isoform B of Transforming growth factor beta-2	NASNLVK	HexNAc(2)Hex(8)	22.676	4.59.E-04
P61812	Isoform B of Transforming growth factor beta-2	ISLHCPCCTFVPSNNYIIPNK	HexNAc(2)Hex(8)	4.860	4.97.E-03
P98164	Low-density lipoprotein receptor-related protein 2	NCNSTETCQPSQFNCPNHR	HexNAc(2)Hex(8)	100.000	1.00.E-17
P98164	Low-density lipoprotein receptor-related protein 2	SFLDCTNR	HexNAc(2)Hex(8)	100.000	1.00.E-17
P98164	Low-density lipoprotein receptor-related protein 2	VCDGILDCPGREDENNTSTGK	HexNAc(2)Hex(8)	13.539	2.28.E-03
P98164	Low-density lipoprotein receptor-related protein 2	INTTYR	HexNAc(2)Hex(8)	5.761	4.61.E-03
P98164	Low-density lipoprotein receptor-related protein 2	NLTNPR	HexNAc(2)Hex(8)	5.581	1.67.E-02
Q02809	Isoform 2 of Procollagen-lysine,2-oxoglutarate 5-dioxygenase 1	YNCSIR	HexNAc(2)Hex(8)	5.140	3.48.E-03
Q07954	Prolow-density lipoprotein receptor-related protein 1	IETILLNGTDRK	HexNAc(2)Hex(8)	100.000	1.00.E-17
Q07954	Prolow-density lipoprotein receptor-related protein 1	LYWISSGNHTINR	HexNAc(2)Hex(8)	100.000	1.00.E-17
Q07954	Prolow-density lipoprotein receptor-related protein 1	NSTTLVMHMK	HexNAc(2)Hex(8)	2.725	4.76.E-02
Q13201	Multimerin-1	YQQNMSHLEEK	HexNAc(2)Hex(8)	100.000	1.00.E-17
Q13201	Multimerin-1	LNQSNFQK	HexNAc(2)Hex(8)	100.000	1.00.E-17
Q13201	Multimerin-1	HPFTGDNCTIK	HexNAc(2)Hex(8)	4.301	1.74.E-02
Q13349	Integrin alpha-D	VNNLSQR	HexNAc(2)Hex(8)	9.050	2.79.E-03
Q13443	Disintegrin and metalloproteinase domain-containing protein 9	NFSSCSAEDFEK	HexNAc(2)Hex(8)	6.303	6.75.E-03
Q13641	Trophoblast glycoprotein	RPPLAELAALNLSGSR	HexNAc(2)Hex(8)	2.247	1.87.E-02
Q13683	Integrin alpha-7	ANITVK	HexNAc(2)Hex(8)	11.160	2.30.E-03
Q13683	Integrin alpha-7	NITLDCAR	HexNAc(2)Hex(8)	5.927	1.23.E-02
Q13683	Integrin alpha-7	KNITLDCAR	HexNAc(2)Hex(8)	5.352	5.97.E-03
Q14108	Lysosome membrane protein 2	CNMINGTDGDSFHPLITK	HexNAc(2)Hex(8)	4.155	9.16.E-03
Q14517	Protocadherin Fat 1	QVTQEMLNHTIAIR	HexNAc(2)Hex(8)	4.236	2.60.E-02
Q15165	Serum paraoxonase/arylesterase 2	HTNMNLTQLK	HexNAc(2)Hex(8)	2.475	1.84.E-02
Q15303	Receptor tyrosine-protein kinase erbB-4	NLTEILNGGVYVDQNK	HexNAc(2)Hex(8)	100.000	1.00.E-17
Q15334	Lethal(2) giant larvae protein homolog 1	QELFAFNK	HexNAc(2)Hex(8)	100.000	1.00.E-17
Q16851	UTP--glucose-1-phosphate uridylyltransferase	NVSLK	HexNAc(2)Hex(8)	4.703	1.90.E-02
Q29983	MHC class I polypeptide-related sequence A	TVPPMVNVTR	HexNAc(2)Hex(8)	2.557	4.17.E-02
Q5IJ48	Protein crumbs homolog 2	NGSLAGGVR	HexNAc(2)Hex(8)	7.007	5.01.E-03
Q5IJ48	Protein crumbs homolog 2	EGPPAAFSGHNASSGR	HexNAc(2)Hex(8)	4.755	7.08.E-03
Q5IJ48	Protein crumbs homolog 2	LDGCHLPFFPLPLDNSSQPSELGGR	HexNAc(2)Hex(8)	4.652	1.35.E-02
Q5T4D3	Isoform 3 of Transmembrane and TPR repeat-containing protein 4	GNQTAAIR	HexNAc(2)Hex(8)	100.000	1.00.E-17
Q6EMK4	Vasorin	NLSGPDK	HexNAc(2)Hex(8)	3.826	4.16.E-02
Q6N022	Teneurin-4	FNVSLGK	HexNAc(2)Hex(8)	100.000	1.00.E-17
Q6UXK2	Immunoglobulin superfamily containing leucine-rich repeat protein 2	AHNELGANSTSIR	HexNAc(2)Hex(8)	100.000	1.00.E-17
Q7Z553	Isoform 3 of MAM domain-containing glycosylphosphatidylinositol anchor protein 2	IVNVSR	HexNAc(2)Hex(8)	100.000	1.00.E-17
Q8IV08	Phospholipase D3	ELGVVMYNCSCLAR	HexNAc(2)Hex(8)	100.000	1.00.E-17
Q8IWU6	Extracellular sulfatase Sulf-1	FYNYTVCR	HexNAc(2)Hex(8)	100.000	1.00.E-17
Q8IWU6	Extracellular sulfatase Sulf-1	CFILPNDSIHCER	HexNAc(2)Hex(8)	100.000	1.00.E-17
Q8IYK4	Procollagen galactosyltransferase 2	ALNTSQLK	HexNAc(2)Hex(8)	21.718	5.70.E-05
Q8NBJ4	Golgi membrane protein 1	AVLVNNITTGER	HexNAc(2)Hex(8)	100.000	1.00.E-17
Q8NBJ5	Procollagen galactosyltransferase 1	TALWVATDHNMDNTSTVLR	HexNAc(2)Hex(8)	100.000	1.00.E-17
Q92820	Gamma-glutamyl hydrolase	NFTMNEK	HexNAc(2)Hex(8)	100.000	1.00.E-17
Q92823	Neuronal cell adhesion molecule	FNHTQTIQQK	HexNAc(2)Hex(8)	5.633	3.10.E-02
Q96AC1	Isoform 3 of Fermitin family homolog 2	VNFSDR	HexNAc(2)Hex(8)	100.000	1.00.E-17
Q96GX1	Tectonic-2	FITNTETPLNNGSTPR	HexNAc(2)Hex(8)	100.000	1.00.E-17
Q96M96	FYVE, RhoGEF and PH domain-containing protein 4	NMTER	HexNAc(2)Hex(8)	100.000	1.00.E-17
Q96MM7	Isoform 4 of Heparan-sulfate 6-O-sulfotransferase 2	YNFTR	HexNAc(2)Hex(8)	9.247	8.24.E-04
Q96N03	V-set and transmembrane domain-containing protein 2-like protein	VVGSNISHK	HexNAc(2)Hex(8)	100.000	1.00.E-17
Q96NI6	Leucine-rich repeat and fibronectin type-III domain-containing protein 5	LISNATR	HexNAc(2)Hex(8)	100.000	1.00.E-17
Q96PQ0	VPS10 domain-containing receptor SorCS2	YVTCAIHNCSEK	HexNAc(2)Hex(8)	100.000	1.00.E-17
Q96SM3	Probable carboxypeptidase X1	VPGSTPALHSSPAQPPAETANGTSEQHVR	HexNAc(2)Hex(8)	100.000	1.00.E-17
Q99435	Isoform 3 of Protein kinase C-binding protein NELL2	QVPGLHNGTK	HexNAc(2)Hex(8)	2.600	3.19.E-02
Q99571	Isoform 2 of P2X purinoceptor 4	FNFSK	HexNAc(2)Hex(8)	2.323	3.99.E-02
Q9BXX0	EMILIN-2	SLNDTMHR	HexNAc(2)Hex(8)	100.000	1.00.E-17
Q9H1J7	Protein Wnt-5b	WNCSTADNASVFGR	HexNAc(2)Hex(8)	100.000	1.00.E-17
Q9H330	Transmembrane protein 245	VNNTAVIEK	HexNAc(2)Hex(8)	2.105	4.12.E-02
Q9HAR2	Isoform 4 of Adhesion G protein-coupled receptor L3	QSEENFNPNCSFWSYSK	HexNAc(2)Hex(8)	100.000	1.00.E-17
Q9HAR2	Isoform 4 of Adhesion G protein-coupled receptor L3	LLTTNK	HexNAc(2)Hex(8)	10.980	2.02.E-02
Q9HCU4	Cadherin EGF LAG seven-pass G-type receptor 2	NATQHTAGYFGSDVK	HexNAc(2)Hex(8)	3.930	2.23.E-02
Q9HD45	Transmembrane 9 superfamily member 3	IVDVNLTSEGK	HexNAc(2)Hex(8)	100.000	1.00.E-17
Q9UBG0	C-type mannose receptor 2	VTPACNTSLPAQR	HexNAc(2)Hex(8)	100.000	1.00.E-17
Q9UBG0	C-type mannose receptor 2	NCTSFR	HexNAc(2)Hex(8)	9.897	1.96.E-03
Q9UBS9	Isoform 2 of SUN domain-containing ossification factor	ALEVNMSLSGR	HexNAc(2)Hex(8)	6.529	5.09.E-03
Q9UBS9	Isoform 2 of SUN domain-containing ossification factor	SALNASDNLK	HexNAc(2)Hex(8)	6.296	5.47.E-03
Q9UBS9	Isoform 2 of SUN domain-containing ossification factor	SISENATATAAPK	HexNAc(2)Hex(8)	4.098	9.31.E-03

Q9UBV2	Protein sel-1 homolog 1	EASIVGENETYPR	HexNAc(2)Hex(8)	2.173	4.25.E-02
Q9UH99	Isoform 2 of SUN domain-containing protein 2	ALSPNSTISSAPK	HexNAc(2)Hex(8)	100.000	1.00.E-17
Q9UHG3	Prenylcysteine oxidase 1	GELNTSIFSSRPIDK	HexNAc(2)Hex(8)	2.296	1.77.E-02
Q9UHN6	Cell surface hyaluronidase	HPSCVNVSK	HexNAc(2)Hex(8)	2.259	4.36.E-02
Q9UPZ6	Thrombospondin type-1 domain-containing protein 7A	TRNISCVVSDGSADDFSK	HexNAc(2)Hex(8)	100.000	1.00.E-17
Q9Y4K0	Lysyl oxidase homolog 2	NVTCENGLPAVVSCVPGQVFSPDGPS R	HexNAc(2)Hex(8)	100.000	1.00.E-17
Q9Y666	Solute carrier family 12 member 7	SAFDPPDIPVCLLGNR	HexNAc(2)Hex(8)	100.000	1.00.E-17
A8MVW0	Protein FAM171A2	ASVDVFGNR	HexNAc(2)Hex(7)	100.000	1.00.E-17
A8MVW0	Protein FAM171A2	NGTGVIR	HexNAc(2)Hex(7)	3.355	1.88.E-02
O00469	Procollagen-lysine,2-oxoglutarate 5-dioxygenase 2	YNCSIESPR	HexNAc(2)Hex(7)	4.010	3.04.E-02
O14672	Disintegrin and metalloproteinase domain-containing protein 10	NISFMVK	HexNAc(2)Hex(7)	100.000	1.00.E-17
O14672	Disintegrin and metalloproteinase domain-containing protein 10	INTTADEKDPTNPFR	HexNAc(2)Hex(7)	100.000	1.00.E-17
O15031	Plexin-B2	ALSNISLR	HexNAc(2)Hex(7)	100.000	1.00.E-17
O43852	Isoform 3 of Calumenin	NATYGYVLDDPDPDDGFNYK	HexNAc(2)Hex(7)	2.057	3.34.E-02
O60486	Plexin-C1	TNVTVK	HexNAc(2)Hex(7)	100.000	1.00.E-17
O60568	Procollagen-lysine,2-oxoglutarate 5-dioxygenase 3	SAEFFNYTVR	HexNAc(2)Hex(7)	8.204	3.31.E-02
O75051	Plexin-A2	EHYLSSVNK	HexNAc(2)Hex(7)	100.000	1.00.E-17
O75054	Isoform 2 of Immunoglobulin superfamily member 3	TLTLVENKPIQLNCSVK	HexNAc(2)Hex(7)	7.065	4.02.E-03
O75718	Cartilage-associated protein	NCSAAPQPEPAAGLASYPELR	HexNAc(2)Hex(7)	3.154	1.16.E-02
O75882	Attractin	GICNSSDVR	HexNAc(2)Hex(7)	100.000	1.00.E-17
O95490	Isoform 5 of Adhesion G protein-coupled receptor L2	LVDTNK	HexNAc(2)Hex(7)	5.611	1.28.E-02
P02458	Collagen alpha-1(II) chain	LLSTEGSQNITYHCK	HexNAc(2)Hex(7)	100.000	1.00.E-17
P02786	Transferrin receptor protein 1	QNNGAFNETLFR	HexNAc(2)Hex(7)	100.000	1.00.E-17
P06756	Integrin alpha-V	ANTTQPGIVEGGQVLK	HexNAc(2)Hex(7)	100.000	1.00.E-17
P06756	Integrin alpha-V	TAADTTGLQPILNQFTPANISR	HexNAc(2)Hex(7)	4.853	7.85.E-03
P07942	Laminin subunit beta-1	LSDTTSQSNSTAK	HexNAc(2)Hex(7)	100.000	1.00.E-17
P07996	Thrombospondin-1	VVNSTTGPGEHLR	HexNAc(2)Hex(7)	4.220	2.13.E-02
P11047	Laminin subunit gamma-1	CDQCEENYFYNR	HexNAc(2)Hex(7)	100.000	1.00.E-17
P11047	Laminin subunit gamma-1	VNNTLSSQISR	HexNAc(2)Hex(7)	3.142	2.56.E-02
P11717	Cation-independent mannose-6-phosphate receptor	MNFTGGDTCHK	HexNAc(2)Hex(7)	2.014	4.87.E-02
P12109	Collagen alpha-1(VI) chain	NVTAQICIDK	HexNAc(2)Hex(7)	3.789	2.63.E-02
P13674	Prolyl 4-hydroxylase subunit alpha-1	DMSDGFISNLTIQR	HexNAc(2)Hex(7)	4.654	1.06.E-02
P14415	Sodium/potassium-transporting ATPase subunit beta-2	FHVNYTQPLVAVK	HexNAc(2)Hex(7)	3.143	2.34.E-02
P14625	Endoplasmin	TDDEVVQREEEAIQLDGLNASQIR	HexNAc(2)Hex(7)	2.302	4.59.E-02
P17301	Integrin alpha-2	GEYFVNVTTR	HexNAc(2)Hex(7)	3.940	3.54.E-03
P18084	Integrin beta-5	NFTALIPGTTVEILDGDSK	HexNAc(2)Hex(7)	3.599	1.83.E-02
P19022	Cadherin-2	SNISILR	HexNAc(2)Hex(7)	100.000	1.00.E-17
P19022	Cadherin-2	NWTITR	HexNAc(2)Hex(7)	2.772	4.00.E-02
P26012	Integrin beta-8	NYAIIKPIGFNETAK	HexNAc(2)Hex(7)	10.199	1.68.E-03
P27448	Isoform 6 of MAP/microtubule affinity-regulating kinase 3	RNMSFR	HexNAc(2)Hex(7)	100.000	1.00.E-17
P28907	ADP-ribosyl cyclase/cyclic ADP-ribose hydrolase 1	NSTFGSVEVHNLQPEK	HexNAc(2)Hex(7)	2.921	4.26.E-02
P31644	Gamma-aminobutyric acid receptor subunit alpha-5	SIAHNMTTPNK	HexNAc(2)Hex(7)	100.000	1.00.E-17
P35613	Basigin	ALMNGSESR	HexNAc(2)Hex(7)	100.000	1.00.E-17
P43307	Translocon-associated protein subunit alpha	YPQDYQFYIQNFTALPLNTVVPPQR	HexNAc(2)Hex(7)	3.692	1.35.E-02
P48723	Heat shock 70 kDa protein 13	NSTIEAANLAGLK	HexNAc(2)Hex(7)	12.041	5.61.E-04
P56199	Integrin alpha-1	DSCESNHNITCK	HexNAc(2)Hex(7)	5.287	1.29.E-02
P56199	Integrin alpha-1	SENASLVLSSSNQK	HexNAc(2)Hex(7)	5.239	7.95.E-03
P56199	Integrin alpha-1	NTTFNVESTK	HexNAc(2)Hex(7)	3.055	1.62.E-02
P56199	Integrin alpha-1	NTTFNVESTKK	HexNAc(2)Hex(7)	2.748	1.38.E-02
P58215	Lysyl oxidase homolog 3	NITAEDCSHSQDAGVR	HexNAc(2)Hex(7)	100.000	1.00.E-17
P61812	Isoform B of Transforming growth factor beta-2	NASNLVK	HexNAc(2)Hex(7)	4.613	1.01.E-02
P98160	Basement membrane-specific heparan sulfate proteoglycan core protein	ALVNFTR	HexNAc(2)Hex(7)	100.000	1.00.E-17
P98164	Low-density lipoprotein receptor-related protein 2	VCDGILDCPGREDENNTSTGK	HexNAc(2)Hex(7)	100.000	1.00.E-17
P98164	Low-density lipoprotein receptor-related protein 2	MFLNK	HexNAc(2)Hex(7)	3.892	2.19.E-02
P98164	Low-density lipoprotein receptor-related protein 2	INTTYR	HexNAc(2)Hex(7)	3.548	3.42.E-02
P98164	Low-density lipoprotein receptor-related protein 2	YNLSSER	HexNAc(2)Hex(7)	3.308	1.94.E-02
P98164	Low-density lipoprotein receptor-related protein 2	VGMDGTNK	HexNAc(2)Hex(7)	2.464	4.60.E-02
Q02809	Isoform 2 of Procollagen-lysine,2-oxoglutarate 5-dioxygenase 1	EQINITLDHR	HexNAc(2)Hex(7)	100.000	1.00.E-17
Q07954	Prolow-density lipoprotein receptor-related protein 1	NSTTLVMHMK	HexNAc(2)Hex(7)	3.077	2.66.E-02
Q07954	Prolow-density lipoprotein receptor-related protein 1	LNGSFR	HexNAc(2)Hex(7)	2.745	3.06.E-02
Q12913	Receptor-type tyrosine-protein phosphatase eta	YEIDVGNESTTLGYYNGK	HexNAc(2)Hex(7)	100.000	1.00.E-17
Q13349	Integrin alpha-D	VNNLSQR	HexNAc(2)Hex(7)	5.009	1.09.E-02
Q13683	Integrin alpha-7	ANITVK	HexNAc(2)Hex(7)	9.092	7.33.E-04
Q14108	Lysosome membrane protein 2	CNMINGTDGDSFHPLITK	HexNAc(2)Hex(7)	100.000	1.00.E-17
Q14517	Protocadherin Fat 1	TGALTVQNTTQLR	HexNAc(2)Hex(7)	100.000	1.00.E-17
Q14517	Protocadherin Fat 1	QVTQEMLNHTIAIR	HexNAc(2)Hex(7)	5.786	3.87.E-03
Q15303	Receptor tyrosine-protein kinase erbB-4	NLTEILNGGVYVDQNK	HexNAc(2)Hex(7)	100.000	1.00.E-17
Q16620	Isoform 4 of BDNF/NT-3 growth factors receptor	NLTIVDSGLK	HexNAc(2)Hex(7)	100.000	1.00.E-17
Q29983	MHC class I polypeptide-related sequence A	TVPPMVNVTR	HexNAc(2)Hex(7)	3.937	2.15.E-02
Q5IJ48	Protein crumbs homolog 2	EGPPAAFSGHNASSGR	HexNAc(2)Hex(7)	6.055	6.39.E-03
Q6N022	Teneurin-4	FNVSLGK	HexNAc(2)Hex(7)	100.000	1.00.E-17
Q6V0I7	Protocadherin Fat 4	INITVSDVNDHTPK	HexNAc(2)Hex(7)	100.000	1.00.E-17
Q8IWU6	Extracellular sulfatase Sulf-1	FYNYTVCR	HexNAc(2)Hex(7)	100.000	1.00.E-17
Q8IYK4	Procollagen galactosyltransferase 2	ALNTSQLK	HexNAc(2)Hex(7)	28.646	3.52.E-04
Q8NBJ5	Procollagen galactosyltransferase 1	TALWVATDHNMDNTSTVLR	HexNAc(2)Hex(7)	6.135	9.38.E-03
Q8NHM5	Isoform 3 of Lysine-specific demethylase 2B	GLNGTPR	HexNAc(2)Hex(7)	4.321	1.43.E-02
Q92791	Endoplasmic reticulum protein SC65	EEAMLYHNQTAELR	HexNAc(2)Hex(7)	3.528	1.93.E-02
Q92791	Endoplasmic reticulum protein SC65	DSEAFCHANCSGPAPAAKPDPDGGR	HexNAc(2)Hex(7)	2.118	3.81.E-02
Q96KA5	Cleft lip and palate transmembrane protein 1-like protein	DLMVINR	HexNAc(2)Hex(7)	3.122	2.30.E-02

Q99985	Semaphorin-3C	VIQTFNR	HexNAc(2)Hex(7)	100.000	1.00.E-17
Q9H330	Transmembrane protein 245	VNNTAVIEK	HexNAc(2)Hex(7)	4.079	1.41.E-02
Q9H330	Transmembrane protein 245	ILGDKVNNTAVIEK	HexNAc(2)Hex(7)	2.489	2.76.E-02
Q9H488	GDP-fucose protein O-fucosyltransferase 1	LLNR	HexNAc(2)Hex(7)	2.120	4.08.E-02
Q9HAR2	Isoform 4 of Adhesion G protein-coupled receptor L3	QSEENFNPNCSFWSYSK	HexNAc(2)Hex(7)	100.000	1.00.E-17
Q9HAR2	Isoform 4 of Adhesion G protein-coupled receptor L3	LLTTNK	HexNAc(2)Hex(7)	12.252	4.78.E-04
Q9HCK4	Isoform 3 of Roundabout homolog 2	IWCLGNETR	HexNAc(2)Hex(7)	100.000	1.00.E-17
Q9HCU4	Cadherin EGF LAG seven-pass G-type receptor 2	NATQHTAGYFGSDVK	HexNAc(2)Hex(7)	2.658	2.71.E-02
Q9HDC9	Adipocyte plasma membrane-associated protein	NMSFVNDLTVTQDGR	HexNAc(2)Hex(7)	4.243	1.25.E-02
Q9NZN1	Interleukin-1 receptor accessory protein-like 1	EVREDDIGNYTCELK	HexNAc(2)Hex(7)	100.000	1.00.E-17
Q9UBG0	C-type mannose receptor 2	GTDPSLSPSPAALPPAPGTELSYLNGTFR	HexNAc(2)Hex(7)	100.000	1.00.E-17
Q9UBG0	C-type mannose receptor 2	NCTSFR	HexNAc(2)Hex(7)	10.169	3.40.E-03
Q9UBS9	Isoform 2 of SUN domain-containing ossification factor	SALNASDNLK	HexNAc(2)Hex(7)	100.000	1.00.E-17
Q9UBS9	Isoform 2 of SUN domain-containing ossification factor	ALEVNMSLSGR	HexNAc(2)Hex(7)	100.000	1.00.E-17
Q9UBS9	Isoform 2 of SUN domain-containing ossification factor	TATDFYAELQNSTDLGYANGNLVHGSNQK	HexNAc(2)Hex(7)	9.509	2.73.E-03
Q9UBV2	Protein sel-1 homolog 1	EASIVGENETYPR	HexNAc(2)Hex(7)	100.000	1.00.E-17
Q9UH99	Isoform 2 of SUN domain-containing protein 2	ALSPNSTISSAPK	HexNAc(2)Hex(7)	100.000	1.00.E-17
Q9UHC6	Contactin-associated protein-like 2	SINLTLDR	HexNAc(2)Hex(7)	100.000	1.00.E-17
Q9UHG3	Prenylcysteine oxidase 1	LLHALGGDDFLGMLNR	HexNAc(2)Hex(7)	100.000	1.00.E-17
Q9UHN6	Cell surface hyaluronidase	NNISLVK	HexNAc(2)Hex(7)	4.200	1.02.E-02
Q9UIQ6	Leucyl-cystinyl aminopeptidase	NQSIGLIQPFATNGK	HexNAc(2)Hex(7)	2.564	3.64.E-02
O00469	Procollagen-lysine,2-oxoglutarate 5-dioxygenase 2	YNCSIESPRK	HexNAc(2)Hex(6)	100.000	1.00.E-17
O15031	Plexin-B2	LHVTLYNCSFGR	HexNAc(2)Hex(6)	5.484	6.58.E-03
O15031	Plexin-B2	SCVAVTSAQPQNMSR	HexNAc(2)Hex(6)	4.043	2.50.E-02
O15230	Laminin subunit alpha-5	DNATLQATLHAAR	HexNAc(2)Hex(6)	2.806	3.36.E-02
O15460	Prolyl 4-hydroxylase subunit alpha-2	TLTNQTEAELATPEGIYERPVDYLPER	HexNAc(2)Hex(6)	3.941	9.07.E-03
O60568	Procollagen-lysine,2-oxoglutarate 5-dioxygenase 3	SAEFFNYTVR	HexNAc(2)Hex(6)	8.628	1.31.E-03
O75179	Ankyrin repeat domain-containing protein 17	PSNVSQDR	HexNAc(2)Hex(6)	2.426	4.20.E-02
O94813	Slit homolog 2 protein	IITNLSK	HexNAc(2)Hex(6)	100.000	1.00.E-17
O95674	Phosphatidate cytidylyltransferase 2	CFVCPVEYNNDTNSFTVDCEPSDLFR	HexNAc(2)Hex(6)	14.019	2.03.E-04
O95754	Semaphorin-4F	TEVTQVNTTNCGR	HexNAc(2)Hex(6)	100.000	1.00.E-17
P00450	Ceruloplasmin	EHEGAIYPDNTTDFQR	HexNAc(2)Hex(6)	100.000	1.00.E-17
P01137	Transforming growth factor beta-1	YSNNSWR	HexNAc(2)Hex(6)	100.000	1.00.E-17
P05997	Collagen alpha-2(V) chain	EASQNITYICK	HexNAc(2)Hex(6)	14.155	1.04.E-03
P06756	Integrin alpha-V	ANTTQPGIVEGGQVLK	HexNAc(2)Hex(6)	9.853	1.22.E-03
P08842	Steryl-sulfatase	NYEIIQQPMSYDNLTQR	HexNAc(2)Hex(6)	2.679	2.39.E-02
P10909	Isoform 2 of Clusterin	LANLTQGEDQYYLR	HexNAc(2)Hex(6)	100.000	1.00.E-17
P11047	Laminin subunit gamma-1	TANDTSTEAYNLLLR	HexNAc(2)Hex(6)	3.278	1.62.E-02
P11047	Laminin subunit gamma-1	VNNTLSSQISR	HexNAc(2)Hex(6)	3.019	2.35.E-02
P11047	Laminin subunit gamma-1	KIPAINQTITEANEK	HexNAc(2)Hex(6)	2.124	3.56.E-02
P12107	Isoform B of Collagen alpha-1(XI) chain	VYCNFTSGGETCIYPDK	HexNAc(2)Hex(6)	100.000	1.00.E-17
P12107	Isoform B of Collagen alpha-1(XI) chain	VYCNFTSGGETCIYPDKK	HexNAc(2)Hex(6)	7.527	4.49.E-03
P12109	Collagen alpha-1(VI) chain	NVTAQICIDK	HexNAc(2)Hex(6)	3.249	1.58.E-02
P13674	Prolyl 4-hydroxylase subunit alpha-1	DMSDGFISNLTIQR	HexNAc(2)Hex(6)	4.743	9.87.E-03
P14314	Glucosidase 2 subunit beta	DGSDEPGTAACPNGSFHCTNTGYK	HexNAc(2)Hex(6)	100.000	1.00.E-17
P14625	Endoplasmin	GVVDSDDLPLNVSR	HexNAc(2)Hex(6)	6.593	6.35.E-03
P14625	Endoplasmin	TDDEVVQREEEAIQLDGLNASQIR	HexNAc(2)Hex(6)	2.584	1.62.E-02
P19022	Cadherin-2	NWTITR	HexNAc(2)Hex(6)	100.000	1.00.E-17
P20908	Collagen alpha-1(V) chain	VYCNFTAGGSTCVFPDKK	HexNAc(2)Hex(6)	4.027	2.38.E-02
P25391	Laminin subunit alpha-1	DVAGLSQELLNTSASLSR	HexNAc(2)Hex(6)	8.020	1.78.E-03
P26006	Isoform 2 of Integrin alpha-3	NITIVTGAPR	HexNAc(2)Hex(6)	4.889	1.27.E-02
P28907	ADP-ribosyl cyclase/cyclic ADP-ribose hydrolase 1	NSTFGSVEVHNLQPEK	HexNAc(2)Hex(6)	100.000	1.00.E-17
P35556	Fibrillin-2	AFNTTK	HexNAc(2)Hex(6)	100.000	1.00.E-17
P43146	Netrin receptor DCC	QQLSNGSLLIQNILHSR	HexNAc(2)Hex(6)	100.000	1.00.E-17
P48723	Heat shock 70 kDa protein 13	NSTIEAANLAGLK	HexNAc(2)Hex(6)	100.000	1.00.E-17
P49746	Thrombospondin-3	LGFLGNQSQGCLPAR	HexNAc(2)Hex(6)	7.158	3.76.E-03
P51654	Isoform 3 of Glypican-3	NYTNAMFK	HexNAc(2)Hex(6)	2.859	4.42.E-02
P56199	Integrin alpha-1	VYVYALNQTR	HexNAc(2)Hex(6)	100.000	1.00.E-17
P56199	Integrin alpha-1	NTTFNVESTK	HexNAc(2)Hex(6)	4.523	1.27.E-02
P56199	Integrin alpha-1	NTTFNVESTKK	HexNAc(2)Hex(6)	3.816	1.72.E-02
P61812	Isoform B of Transforming growth factor beta-2	NASNLVK	HexNAc(2)Hex(6)	100.000	1.00.E-17
P98164	Low-density lipoprotein receptor-related protein 2	YNLSSER	HexNAc(2)Hex(6)	7.502	4.14.E-03
P98164	Low-density lipoprotein receptor-related protein 2	INTTYR	HexNAc(2)Hex(6)	2.915	2.95.E-02
P98164	Low-density lipoprotein receptor-related protein 2	NLTNPR	HexNAc(2)Hex(6)	2.874	4.66.E-02
Q07954	Prolow-density lipoprotein receptor-related protein 1	WTGHNVTVVQR	HexNAc(2)Hex(6)	100.000	1.00.E-17
Q07954	Prolow-density lipoprotein receptor-related protein 1	NSTTLVMHMK	HexNAc(2)Hex(6)	6.469	4.86.E-03
Q07954	Prolow-density lipoprotein receptor-related protein 1	LNGSFR	HexNAc(2)Hex(6)	3.095	1.64.E-02
Q08722	Leukocyte surface antigen CD47	DIYTFDGALNK	HexNAc(2)Hex(6)	2.175	2.53.E-02
Q12797	Isoform 7 of Aspartyl/asparaginyl beta-hydroxylase	YNLSEVLQGK	HexNAc(2)Hex(6)	3.622	2.28.E-02
Q13443	Disintegrin and metalloproteinase domain-containing protein 9	NFSSCSAEDFEK	HexNAc(2)Hex(6)	100.000	1.00.E-17
Q13683	Integrin alpha-7	KNITLDCAR	HexNAc(2)Hex(6)	6.173	8.59.E-03
Q13683	Integrin alpha-7	NITLDCAR	HexNAc(2)Hex(6)	3.210	1.93.E-02
Q13683	Integrin alpha-7	ANITVK	HexNAc(2)Hex(6)	2.441	2.07.E-02
Q14517	Protocadherin Fat 1	QVTQEMLNHTIAIR	HexNAc(2)Hex(6)	100.000	1.00.E-17
Q14517	Protocadherin Fat 1	TGALTVQNTTQLR	HexNAc(2)Hex(6)	17.218	1.97.E-04
Q15165	Serum paraoxonase/arylesterase 2	IQNILCEKPTVTTVYANNGSVLQGSSVASVYDGK	HexNAc(2)Hex(6)	100.000	1.00.E-17
Q15165	Serum paraoxonase/arylesterase 2	HTNMNLTQLK	HexNAc(2)Hex(6)	100.000	1.00.E-17
Q15904	V-type proton ATPase subunit S1	QPVSPVIHPPVSYNDTAPR	HexNAc(2)Hex(6)	3.469	1.22.E-02

Q16620	Isoform 4 of BDNF/NT-3 growth factors receptor	NSNLQHINFTR	HexNAc(2)Hex(6)	100.000	1.00.E-17
Q16620	Isoform 4 of BDNF/NT-3 growth factors receptor	NLTIVDSGLK	HexNAc(2)Hex(6)	100.000	1.00.E-17
Q4KMQ2	Isoform 2 of Anoctamin-6	LNITCESSK	HexNAc(2)Hex(6)	2.692	4.78.E-02
Q58EX2	Isoform 4 of Protein sidekick-2	FWLVEGNSSR	HexNAc(2)Hex(6)	100.000	1.00.E-17
Q5IJ48	Protein crumbs homolog 2	NGSLAGGVR	HexNAc(2)Hex(6)	100.000	1.00.E-17
Q5IJ48	Protein crumbs homolog 2	EGPPAAFSGHNASSGR	HexNAc(2)Hex(6)	3.377	2.75.E-02
Q5T4D3	Isoform 3 of Transmembrane and TPR repeat-containing protein 4	NLADKGNQTAAIR	HexNAc(2)Hex(6)	100.000	1.00.E-17
Q5T4D3	Isoform 3 of Transmembrane and TPR repeat-containing protein 4	GNQTAAIR	HexNAc(2)Hex(6)	12.927	1.53.E-03
Q6N022	Teneurin-4	FNVSLGK	HexNAc(2)Hex(6)	100.000	1.00.E-17
Q70UQ0	Isoform 4 of Inhibitor of nuclear factor kappa-B kinase-interacting protein	FQNITDFWK	HexNAc(2)Hex(6)	100.000	1.00.E-17
Q70UQ0	Inhibitor of nuclear factor kappa-B kinase-interacting protein	ISNLTIVQAEIK	HexNAc(2)Hex(6)	6.556	5.21.E-03
Q7Z388	Probable C-mannosyltransferase DPY19L4	FGLNMTK	HexNAc(2)Hex(6)	100.000	1.00.E-17
Q7Z4H8	KDEL motif-containing protein 2	VNGTPSPIPIISWCGSLDSR	HexNAc(2)Hex(6)	3.969	2.25.E-02
Q7Z4K8	Tripartite motif-containing protein 46	NLTLER	HexNAc(2)Hex(6)	100.000	1.00.E-17
Q7Z5J1	Isoform 2 of Hydroxysteroid 11-beta-dehydrogenase 1-like protein	QELNVTAAAA	HexNAc(2)Hex(6)	100.000	1.00.E-17
Q8IWU6	Extracellular sulfatase Sulf-1	FYNYTVCR	HexNAc(2)Hex(6)	100.000	1.00.E-17
Q8IWU6	Extracellular sulfatase Sulf-1	CFILPNDSIHCER	HexNAc(2)Hex(6)	100.000	1.00.E-17
Q8IYK4	Procollagen galactosyltransferase 2	ALNTSQLK	HexNAc(2)Hex(6)	16.715	2.04.E-04
Q8IYK4	Procollagen galactosyltransferase 2	MAIWAATDHNVDNTTEIFR	HexNAc(2)Hex(6)	12.315	1.16.E-04
Q8NBJ5	Procollagen galactosyltransferase 1	AMNTSQVEALGIQMLPGYR	HexNAc(2)Hex(6)	4.869	1.10.E-02
Q8TDW7	Protocadherin Fat 3	ANYSLK	HexNAc(2)Hex(6)	3.608	1.69.E-02
Q92791	Endoplasmic reticulum protein SC65	EEAMLYHNQTAELR	HexNAc(2)Hex(6)	100.000	1.00.E-17
Q92823	Neuronal cell adhesion molecule	FNHTQTIQQK	HexNAc(2)Hex(6)	2.369	3.62.E-02
Q96AY4	Tetratricopeptide repeat protein 28	NNLFNR	HexNAc(2)Hex(6)	100.000	1.00.E-17
Q96G97	Isoform 3 of Seipin	TDCDSSTTSLCSFPVANVSLTK	HexNAc(2)Hex(6)	2.216	4.11.E-02
Q96JJ7	Protein disulfide-isomerase TMX3	LVALAVIDEKNTSVEHTR	HexNAc(2)Hex(6)	100.000	1.00.E-17
Q96KA5	Cleft lip and palate transmembrane protein 1-like protein	DLMVINR	HexNAc(2)Hex(6)	100.000	1.00.E-17
Q96MM7	Isoform 4 of Heparan-sulfate 6-O-sulfotransferase 2	YNFTR	HexNAc(2)Hex(6)	100.000	1.00.E-17
Q99435	Isoform 3 of Protein kinase C-binding protein NELL2	QVPGLHNGTK	HexNAc(2)Hex(6)	6.532	1.15.E-02
Q99574	Neuroserpin	DANLTGLSDNK	HexNAc(2)Hex(6)	100.000	1.00.E-17
Q9BU23	Lipase maturation factor 2	SANSTLAQALHWTR	HexNAc(2)Hex(6)	5.537	4.44.E-03
Q9C0A0	Contactin-associated protein-like 4	NGSLQIR	HexNAc(2)Hex(6)	100.000	1.00.E-17
Q9H330	Transmembrane protein 245	ILGDKVNNTAVIEK	HexNAc(2)Hex(6)	7.563	3.79.E-03
Q9H330	Transmembrane protein 245	VNNTAVIEK	HexNAc(2)Hex(6)	4.408	6.90.E-03
Q9H488	GDP-fucose protein O-fucosyltransferase 1	LLNR	HexNAc(2)Hex(6)	3.518	2.33.E-02
Q9HAR2	Isoform 4 of Adhesion G protein-coupled receptor L3	NLTPGGK	HexNAc(2)Hex(6)	100.000	1.00.E-17
Q9HAR2	Isoform 4 of Adhesion G protein-coupled receptor L3	LLTTNK	HexNAc(2)Hex(6)	100.000	1.00.E-17
Q9HCK4	Isoform 3 of Roundabout homolog 2	IWCLGNETR	HexNAc(2)Hex(6)	100.000	1.00.E-17
Q9HDC9	Adipocyte plasma membrane-associated protein	NMSFVNDLTVTQDGRK	HexNAc(2)Hex(6)	18.106	2.86.E-04
Q9HDC9	Adipocyte plasma membrane-associated protein	NMSFVNDLTVTQDGR	HexNAc(2)Hex(6)	5.180	6.17.E-03
Q9NXG6	Isoform 3 of Transmembrane prolyl 4-hydroxylase	LVANESVPFETSCR	HexNAc(2)Hex(6)	3.514	9.04.E-03
Q9NY27	Serine/threonine-protein phosphatase 4 regulatory subunit 2	NNSNSLNR	HexNAc(2)Hex(6)	2.668	4.71.E-02
Q9UBG0	C-type mannose receptor 2	NCTSFR	HexNAc(2)Hex(6)	4.906	6.68.E-03
Q9UBS5	Isoform 1C of Gamma-aminobutyric acid type B receptor subunit 1	SISNMTSQEFVEK	HexNAc(2)Hex(6)	100.000	1.00.E-17
Q9UBS9	Isoform 2 of SUN domain-containing ossification factor	SALNASDNLK	HexNAc(2)Hex(6)	9.634	3.12.E-03
Q9UBS9	Isoform 2 of SUN domain-containing ossification factor	SISENATATAAPK	HexNAc(2)Hex(6)	3.537	1.43.E-02
Q9UBS9	Isoform 2 of SUN domain-containing ossification factor	TATDFYAELQNSTDLGYANGNLVHGSNQK	HexNAc(2)Hex(6)	3.211	2.66.E-02
Q9UBV2	Protein sel-1 homolog 1	MYSEGSDIVPQSNETALHYFK	HexNAc(2)Hex(6)	100.000	1.00.E-17
Q9UH99	Isoform 2 of SUN domain-containing protein 2	ALSPNSTISSAPK	HexNAc(2)Hex(6)	5.772	1.02.E-02
Q9UIQ6	Leucyl-cystinyl aminopeptidase	NQSIGLIQPFATNGK	HexNAc(2)Hex(6)	100.000	1.00.E-17
Q9UJ14	Glutathione hydrolase 7	NLSDSLAR	HexNAc(2)Hex(6)	100.000	1.00.E-17
Q9Y4D7	Plexin-D1	ANFTIYDCSR	HexNAc(2)Hex(6)	7.571	1.82.E-03
O00469	Procollagen-lysine,2-oxoglutarate 5-dioxygenase 2	YNCSIESPR	HexNAc(2)Hex(5)	100.000	1.00.E-17
O15031	Plexin-B2	SCVAVTSAQPQNMSR	HexNAc(2)Hex(5)	100.000	1.00.E-17
O15031	Plexin-B2	LHVTLYNCSFGR	HexNAc(2)Hex(5)	3.258	1.96.E-02
O15394	Neural cell adhesion molecule 2	YNCTATNHIGTR	HexNAc(2)Hex(5)	4.582	4.21.E-02
O60242	Adhesion G protein-coupled receptor B3	NVTDTFK	HexNAc(2)Hex(5)	100.000	1.00.E-17
O60245	Isoform B of Protocadherin-7	NISYTLLPPSSNVR	HexNAc(2)Hex(5)	100.000	1.00.E-17
O60486	Plexin-C1	NGTEVVSCHPQGSTAGVVYR	HexNAc(2)Hex(5)	100.000	1.00.E-17
O60568	Procollagen-lysine,2-oxoglutarate 5-dioxygenase 3	EQYIHENYSR	HexNAc(2)Hex(5)	3.507	1.24.E-02
O60637	Tetraspanin-3	NQSVPLSCCR	HexNAc(2)Hex(5)	3.403	7.68.E-03
O75882	Attractin	CINQSICEK	HexNAc(2)Hex(5)	100.000	1.00.E-17
O75976	Carboxypeptidase D	NNSNNFDLNR	HexNAc(2)Hex(5)	100.000	1.00.E-17
O76024	Wolframin	CLYGEAYPACSPGNTSTAEEELCR	HexNAc(2)Hex(5)	100.000	1.00.E-17
O94779	Contactin-5	ILNASK	HexNAc(2)Hex(5)	100.000	1.00.E-17
O94856	Neurofascin	VILYNR	HexNAc(2)Hex(5)	100.000	1.00.E-17
O94905	Erlin-1	NYTADYDK	HexNAc(2)Hex(5)	100.000	1.00.E-17
P01137	Transforming growth factor beta-1	YSNNSWR	HexNAc(2)Hex(5)	100.000	1.00.E-17
P05556	Isoform 5 of Integrin beta-1	NGVNGTGENGRK	HexNAc(2)Hex(5)	3.930	2.72.E-02
P06756	Integrin alpha-V	NMTISR	HexNAc(2)Hex(5)	100.000	1.00.E-17
P06756	Integrin alpha-V	ANTTQPGIVEGGQVLK	HexNAc(2)Hex(5)	11.347	5.28.E-04
P06756	Integrin alpha-V	ENQNHSYSLK	HexNAc(2)Hex(5)	9.765	8.34.E-04
P07996	Thrombospondin-1	VSCPIMPCSNATVPDGECCPR	HexNAc(2)Hex(5)	100.000	1.00.E-17
P07996	Thrombospondin-1	VVNSTTGPGEHLR	HexNAc(2)Hex(5)	5.418	9.89.E-03
P08069	Insulin-like growth factor 1 receptor	LNPGNYTAR	HexNAc(2)Hex(5)	7.230	4.48.E-03
P08581	Isoform 2 of Hepatocyte growth factor receptor	NFTVACQHR	HexNAc(2)Hex(5)	100.000	1.00.E-17
P0C7U0	Protein ELFN1	MYTLEHFNNSK	HexNAc(2)Hex(5)	100.000	1.00.E-17

P11047	Laminin subunit gamma-1	TLAGENQTAFEIEELNRK	HexNAc(2)Hex(5)	100.000	1.00.E-17
P11047	Laminin subunit gamma-1	KIPAINQTITEANEK	HexNAc(2)Hex(5)	100.000	1.00.E-17
P11047	Laminin subunit gamma-1	VNNTLSSQISR	HexNAc(2)Hex(5)	5.373	1.21.E-02
P11532	Isoform 2 of Dystrophin	AQNVTR	HexNAc(2)Hex(5)	12.491	1.16.E-03
P13473	Isoform LAMP-2C of Lysosome-associated membrane glycoprotein 2	LNSSTIK	HexNAc(2)Hex(5)	100.000	1.00.E-17
P13591	Neural cell adhesion molecule 1	DGQLLPSSNYSNIK	HexNAc(2)Hex(5)	100.000	1.00.E-17
P13674	Prolyl 4-hydroxylase subunit alpha-1	DMSDGFISNLTIQR	HexNAc(2)Hex(5)	3.487	2.33.E-02
P14415	Sodium/potassium-transporting ATPase subunit beta-2	FLEPYNDSIQAQK	HexNAc(2)Hex(5)	3.005	3.12.E-02
P14625	Endoplasmin	LGVIEDHSNR	HexNAc(2)Hex(5)	100.000	1.00.E-17
P14625	Endoplasmin	TDDEVVQREEEAIQLDGLNASQIR	HexNAc(2)Hex(5)	5.242	7.76.E-03
P17050	Alpha-N-acetylgalactosaminidase	MAAALNATGR	HexNAc(2)Hex(5)	100.000	1.00.E-17
P19022	Cadherin-2	SNISILR	HexNAc(2)Hex(5)	3.054	8.21.E-03
P26006	Isoform 2 of Integrin alpha-3	TSIPTINMENK	HexNAc(2)Hex(5)	6.108	4.61.E-03
P38435	Vitamin K-dependent gamma-carboxylase	VENGSETGPLPPELQPLLEGEVK	HexNAc(2)Hex(5)	4.202	1.47.E-02
P42892	Endothelin-converting enzyme 1	HLLENSTASVSEAERK	HexNAc(2)Hex(5)	100.000	1.00.E-17
P52799	Ephrin-B2	SIVLEPIYWNSSNSK	HexNAc(2)Hex(5)	3.937	2.85.E-02
P54802	Alpha-N-acetylglucosaminidase	SVYNCSGEACR	HexNAc(2)Hex(5)	100.000	1.00.E-17
P61812	Isoform B of Transforming growth factor beta-2	NASNLVK	HexNAc(2)Hex(5)	7.165	5.63.E-03
P62910	60S ribosomal protein L32	ELEVLLMCNK	HexNAc(2)Hex(5)	100.000	1.00.E-17
P98164	Low-density lipoprotein receptor-related protein 2	MFLNK	HexNAc(2)Hex(5)	100.000	1.00.E-17
P98164	Low-density lipoprotein receptor-related protein 2	MLAQHCVDANNTFCFDNPR	HexNAc(2)Hex(5)	3.415	3.15.E-02
P98164	Low-density lipoprotein receptor-related protein 2	NLTNPR	HexNAc(2)Hex(5)	2.622	4.13.E-02
P98164	Low-density lipoprotein receptor-related protein 2	VGMDGTNK	HexNAc(2)Hex(5)	2.077	4.19.E-02
Q02246	Contactin-2	GTEILVNSSR	HexNAc(2)Hex(5)	100.000	1.00.E-17
Q07954	Prolow-density lipoprotein receptor-related protein 1	QTTAMDFSYANETVCWVHVGDSAAQTQLK	HexNAc(2)Hex(5)	100.000	1.00.E-17
Q07954	Prolow-density lipoprotein receptor-related protein 1	VNRFNSTEYQVVTR	HexNAc(2)Hex(5)	100.000	1.00.E-17
Q07954	Prolow-density lipoprotein receptor-related protein 1	INNGGCQDLCLLTHQGHVNCSCR	HexNAc(2)Hex(5)	100.000	1.00.E-17
Q07954	Prolow-density lipoprotein receptor-related protein 1	LYWISSGNHTINR	HexNAc(2)Hex(5)	100.000	1.00.E-17
Q07954	Prolow-density lipoprotein receptor-related protein 1	LTSCATNASICGDEAR	HexNAc(2)Hex(5)	100.000	1.00.E-17
Q07954	Prolow-density lipoprotein receptor-related protein 1	NSTTLVMHMK	HexNAc(2)Hex(5)	7.699	2.42.E-03
Q07954	Prolow-density lipoprotein receptor-related protein 1	WLCDGDNDCGNSEDESNATCSAR	HexNAc(2)Hex(5)	3.695	2.63.E-02
Q07954	Prolow-density lipoprotein receptor-related protein 1	FNSTEYQVVTR	HexNAc(2)Hex(5)	3.448	3.22.E-02
Q07954	Prolow-density lipoprotein receptor-related protein 1	TCVSNCTASQFVCK	HexNAc(2)Hex(5)	2.991	1.92.E-02
Q07954	Prolow-density lipoprotein receptor-related protein 1	LNGTDPIVAADSK	HexNAc(2)Hex(5)	2.717	1.73.E-02
Q07954	Prolow-density lipoprotein receptor-related protein 1	FGTCSQLCNNTK	HexNAc(2)Hex(5)	2.179	3.44.E-02
Q07954	Prolow-density lipoprotein receptor-related protein 1	DNTTCYEFKK	HexNAc(2)Hex(5)	2.013	2.41.E-02
Q08722	Leukocyte surface antigen CD47	DIYTFDGALNK	HexNAc(2)Hex(5)	5.876	1.24.E-02
Q12866	Tyrosine-protein kinase Mer	NCSIQVK	HexNAc(2)Hex(5)	3.309	3.57.E-02
Q13201	Multimerin-1	YQQNMSHLEEK	HexNAc(2)Hex(5)	100.000	1.00.E-17
Q13308	Isoform 6 of Inactive tyrosine-protein kinase 7	NLTLR	HexNAc(2)Hex(5)	2.625	3.47.E-02
Q13433	Zinc transporter ZIP6	NTNENPQECFNASK	HexNAc(2)Hex(5)	100.000	1.00.E-17
Q13510	Isoform 2 of Acid ceramidase	MCLNR	HexNAc(2)Hex(5)	100.000	1.00.E-17
Q13683	Integrin alpha-7	KNITLDCAR	HexNAc(2)Hex(5)	100.000	1.00.E-17
Q13683	Integrin alpha-7	NITLDCAR	HexNAc(2)Hex(5)	7.490	2.10.E-03
Q13683	Integrin alpha-7	ANITVK	HexNAc(2)Hex(5)	3.010	3.90.E-02
Q14517	Protocadherin Fat 1	TGALTVQNTTQLR	HexNAc(2)Hex(5)	100.000	1.00.E-17
Q14517	Protocadherin Fat 1	ENQPVGSSVIFMNSTDLDTGFNGK	HexNAc(2)Hex(5)	6.221	3.35.E-03
Q15223	Nectin-1	NPNGTVTVISR	HexNAc(2)Hex(5)	100.000	1.00.E-17
Q15303	Receptor tyrosine-protein kinase erbB-4	ACDGIGTGSLMSAQTVDSSNIDKFINCTK	HexNAc(2)Hex(5)	100.000	1.00.E-17
Q16288	NT-3 growth factor receptor	NPLGTANQTINGHFLK	HexNAc(2)Hex(5)	100.000	1.00.E-17
Q16827	Receptor-type tyrosine-protein phosphatase O	GSNTSMLR	HexNAc(2)Hex(5)	100.000	1.00.E-17
Q32P28	Prolyl 3-hydroxylase 1	LLNGSQR	HexNAc(2)Hex(5)	100.000	1.00.E-17
Q4KMQ2	Isoform 2 of Anoctamin-6	NINGTDPIQK	HexNAc(2)Hex(5)	100.000	1.00.E-17
Q58EX2	Isoform 4 of Protein sidekick-2	VISAGGNDSR	HexNAc(2)Hex(5)	100.000	1.00.E-17
Q5T4D3	Isoform 3 of Transmembrane and TPR repeat-containing protein 4	GNQTAAIR	HexNAc(2)Hex(5)	100.000	1.00.E-17
Q6IQ23	Isoform 2 of Pleckstrin homology domain-containing family A member 7	NSSHVDRR	HexNAc(2)Hex(5)	100.000	1.00.E-17
Q6N022	Teneurin-4	VGPYANTTR	HexNAc(2)Hex(5)	100.000	1.00.E-17
Q86YD3	Transmembrane protein 25	AQHELNCSLQDPR	HexNAc(2)Hex(5)	100.000	1.00.E-17
Q8IWU5	Extracellular sulfatase Sulf-2	FYNYTLCR	HexNAc(2)Hex(5)	100.000	1.00.E-17
Q8IYK4	Procollagen galactosyltransferase 2	ALNTSQLK	HexNAc(2)Hex(5)	100.000	1.00.E-17
Q8IZA0	Dyslexia-associated protein KIAA0319-like protein	ALEVNTVTCQLNCSDHGHCDSFTK	HexNAc(2)Hex(5)	100.000	1.00.E-17
Q8NBJ5	Procollagen galactosyltransferase 1	AMNTSQVEALGIQMLPGYR	HexNAc(2)Hex(5)	2.998	4.52.E-02
Q8TDW7	Protocadherin Fat 3	ANYSLK	HexNAc(2)Hex(5)	3.648	1.53.E-02
Q8WX77	Insulin-like growth factor-binding protein-like 1	SVHNVTGAQVGLSCEVR	HexNAc(2)Hex(5)	100.000	1.00.E-17
Q92820	Gamma-glutamyl hydrolase	NFTMNEK	HexNAc(2)Hex(5)	3.455	1.81.E-02
Q92823	Neuronal cell adhesion molecule	FNHTQTIQQK	HexNAc(2)Hex(5)	6.734	5.68.E-03
Q92896	Isoform 2 of Golgi apparatus protein 1	LNLTTDPK	HexNAc(2)Hex(5)	100.000	1.00.E-17
Q96AQ6	Pre-B-cell leukemia transcription factor-interacting protein 1	LQGLENWGQDPGVSANASK	HexNAc(2)Hex(5)	5.469	8.55.E-03
Q96G97	Isoform 3 of Seipin	TDCDSSTTSLCSFPVANVSLTK	HexNAc(2)Hex(5)	2.371	2.71.E-02
Q96HD1	Isoform 2 of Cysteine-rich with EGF-like domain protein 1	NASHLVCSACFGPCAR	HexNAc(2)Hex(5)	2.251	4.84.E-02
Q99985	Semaphorin-3C	VIQTFNR	HexNAc(2)Hex(5)	100.000	1.00.E-17
Q9H2D6	TRIO and F-actin-binding protein	ASSPNRTIQQENLR	HexNAc(2)Hex(5)	100.000	1.00.E-17
Q9HCK4	Isoform 3 of Roundabout homolog 2	IWCLGNETR	HexNAc(2)Hex(5)	100.000	1.00.E-17
Q9HCU4	Cadherin EGF LAG seven-pass G-type receptor 2	LYLNR	HexNAc(2)Hex(5)	3.495	1.89.E-02
Q9HCU4	Cadherin EGF LAG seven-pass G-type receptor 2	NATQHTAGYFGSDVK	HexNAc(2)Hex(5)	2.497	4.69.E-02
Q9HDC9	Adipocyte plasma membrane-associated protein	NMSFVNDLTVTQDGRK	HexNAc(2)Hex(5)	100.000	1.00.E-17
Q9HDC9	Adipocyte plasma membrane-associated protein	NMSFVNDLTVTQDGR	HexNAc(2)Hex(5)	100.000	1.00.E-17

Q9NT99	Leucine-rich repeat-containing protein 4B	ETVPSNTTCCAR	HexNAc(2)Hex(5)	100.000	1.00.E-17
Q9NUM4	Transmembrane protein 106B	LNNITIIGPLDMK	HexNAc(2)Hex(5)	3.444	1.72.E-02
Q9NUN5	Probable lysosomal cobalamin transporter	NQNGTFK	HexNAc(2)Hex(5)	3.473	3.70.E-02
Q9NY47	Voltage-dependent calcium channel subunit alpha-2/delta-2	DLNASDNNTEFLK	HexNAc(2)Hex(5)	100.000	1.00.E-17
Q9NZ53	Podocalyxin-like protein 2	SLEEIGIQNYSTTSSCQAR	HexNAc(2)Hex(5)	2.717	4.75.E-02
Q9P273	Teneurin-3	FNISLQK	HexNAc(2)Hex(5)	100.000	1.00.E-17
Q9P2B2	Prostaglandin F2 receptor negative regulator	ELDLTCNITTDR	HexNAc(2)Hex(5)	100.000	1.00.E-17
Q9UBG0	C-type mannose receptor 2	VTPACNTSLPAQR	HexNAc(2)Hex(5)	9.809	1.85.E-03
Q9UBG0	C-type mannose receptor 2	NCTSFR	HexNAc(2)Hex(5)	8.864	6.24.E-04
Q9UBS9	Isoform 2 of SUN domain-containing ossification factor	SALNASDNLK	HexNAc(2)Hex(5)	4.435	1.43.E-02
Q9UH99	Isoform 2 of SUN domain-containing protein 2	ALSPNSTISSAPK	HexNAc(2)Hex(5)	100.000	1.00.E-17
Q9UHG3	Prenylcysteine oxidase 1	GELNTSIFSSRPIDK	HexNAc(2)Hex(5)	100.000	1.00.E-17
Q9UHN6	Cell surface hyaluronidase	QAWALVGVIDGGSTSCNESVR	HexNAc(2)Hex(5)	3.661	3.16.E-02
Q9UHN6	Cell surface hyaluronidase	HPSCVNVSK	HexNAc(2)Hex(5)	2.097	4.49.E-02
Q9UIQ6	Leucyl-cystinyl aminopeptidase	SALLEFACTHNLGNCSTTAMK	HexNAc(2)Hex(5)	100.000	1.00.E-17
Q9UIQ6	Leucyl-cystinyl aminopeptidase	NQSIGLIQPFATNGK	HexNAc(2)Hex(5)	100.000	1.00.E-17
Q9UIW2	Plexin-A1	ENGCLVYNDTTMVCR	HexNAc(2)Hex(5)	100.000	1.00.E-17
Q9ULB1	Isoform 3a of Neurexin-1	NTTLFIDQVEAK	HexNAc(2)Hex(5)	100.000	1.00.E-17
Q9ULK0	Glutamate receptor ionotropic, delta-1	GLNGSLQER	HexNAc(2)Hex(5)	100.000	1.00.E-17
Q9ULK0	Glutamate receptor ionotropic, delta-1	GLNGSLQERPMGSR	HexNAc(2)Hex(5)	100.000	1.00.E-17
Q9UPU3	VPS10 domain-containing receptor SorCS3	IVSNNCTDGLR	HexNAc(2)Hex(5)	100.000	1.00.E-17
Q9Y4D7	Plexin-D1	ANFTIYDCSR	HexNAc(2)Hex(5)	100.000	1.00.E-17
Q9Y6M7	Isoform 7 of Sodium bicarbonate cotransporter 3	NLTVSECKK	HexNAc(2)Hex(5)	100.000	1.00.E-17
O60637	Tetraspanin-3	NQSVPLSCCR	HexNAc(2)Hex(4)	100.000	1.00.E-17
P07602	Isoform Sap-mu-9 of Prosaposin	TCDWLPKPNMSASCK	HexNAc(2)Hex(4)	2.631	3.93.E-02
P18084	Integrin beta-5	SNLTVLR	HexNAc(2)Hex(4)	100.000	1.00.E-17
P54802	Alpha-N-acetylglucosaminidase	SVYNCSGEACR	HexNAc(2)Hex(4)	100.000	1.00.E-17
Q13683	Integrin alpha-7	ANITVK	HexNAc(2)Hex(4)	100.000	1.00.E-17
Q16527	Cysteine and glycine-rich protein 2	PTTNPNTSKFAQK	HexNAc(2)Hex(4)	100.000	1.00.E-17
Q8IYK4	Procollagen galactosyltransferase 2	ALNTSQLK	HexNAc(2)Hex(4)	100.000	1.00.E-17
Q8N8S7	Protein enabled homolog	NQIVFDNR	HexNAc(2)Hex(4)	100.000	1.00.E-17
O75503	Ceroid-lipofuscinosis neuronal protein 5	NIETNYTR	HexNAc(2)Hex(3)	100.000	1.00.E-17
P07602	Isoform Sap-mu-9 of Prosaposin	TCDWLPKPNMSASCK	HexNAc(2)Hex(3)	100.000	1.00.E-17
P50454	Serpin H1	SLSNSTAR	HexNAc(2)Hex(3)	3.867	1.43.E-02
P54802	Alpha-N-acetylglucosaminidase	SVYNCSGEACR	HexNAc(2)Hex(3)	2.962	1.99.E-02
Q13459	Unconventional myosin-IXb	SGAANR	HexNAc(2)Hex(3)	100.000	1.00.E-17
Q9BTY2	Plasma alpha-L-fucosidase	SQNDTVTPDVWYTSKPK	HexNAc(2)Hex(3)	100.000	1.00.E-17

Supplementary Table S3. (c) N-glycopeptides with increased numbers in the DAs (>2 fold)

Gene Name	Protein name	Sequence	Glycan Composition	Abundance Ratio: (DA) / (iPSC)	Abundance Ratio p-value: (DA) / (iPSC)
A0FGR8	Isoform 2 of Extended synaptotagmin-2	ENLSPK	HexNAc(4)Hex(6)	100	1.00E-17
A0MZ66	Isoform 3 of Shootin-1	LNKENK	HexNAc(3)Hex(6)Fuc(1)	100	1.00E-17
A2RU67	Protein FAM234B	MLSAFNATSGK	HexNAc(2)Hex(9)	100	1.00E-17
A2RU67	Protein FAM234B	NGSAVGVSR	HexNAc(2)Hex(5)	100	1.00E-17
A2RU67	Protein FAM234B	MLSAFNATSGK	HexNAc(2)Hex(5)	15.603	2.18E-03
A4D0S4	Laminin subunit beta-4	RQNDSLDK	HexNAc(5)Hex(4)Fuc(2)	100	1.00E-17
A4D0S4	Laminin subunit beta-4	RQNDSLDK	HexNAc(5)Hex(3)Fuc(1)	100	1.00E-17
A4D1P6	WD repeat-containing protein 91	NASLSQSPR	HexNAc(6)Hex(4)	100	1.00E-17
A6NE02	BTB/POZ domain-containing protein 17	QGNASDVVLR	HexNAc(2)Hex(8)	100	1.00E-17
A6NGN9	IgLON family member 5	HYGNYTCR	HexNAc(4)Hex(5)Fuc(1)	12.45	4.19E-03
A6NGN9	IgLON family member 5	HYGNYTCR	HexNAc(4)Hex(4)Fuc(1)	100	1.00E-17
A6NGN9	IgLON family member 5	HYGNYTCR	HexNAc(3)Hex(6)Fuc(1)	100	1.00E-17
A6NGN9	IgLON family member 5	VAWLNR	HexNAc(2)Hex(9)	3.234	4.54E-02
A6NGN9	IgLON family member 5	VAWLNR	HexNAc(2)Hex(8)	24.398	1.41E-05
A6NGN9	IgLON family member 5	VAWLNR	HexNAc(2)Hex(7)	8.276	5.74E-04
A6NGN9	IgLON family member 5	HYGNYTCR	HexNAc(2)Hex(5)	100	1.00E-17
A7MBM2	Protein dispatched homolog 2	SNSSLVRDPAFSASGPEAQR	HexNAc(2)Hex(9)	100	1.00E-17
A7MBM2	Protein dispatched homolog 2	SNSSLVRDPAFSASGPEAQR	HexNAc(2)Hex(8)	5.692	2.19E-02
A8K979	ERI1 exoribonuclease 2	NLSISTK	HexNAc(6)Hex(3)Fuc(1)	100	1.00E-17
A8K979	ERI1 exoribonuclease 2	NLSISTK	HexNAc(5)Hex(4)Fuc(1)	100	1.00E-17
A8K979	ERI1 exoribonuclease 2	NLSISTK	HexNAc(5)Hex(3)Fuc(1)	100	1.00E-17
A8K979	ERI1 exoribonuclease 2	NLSISTK	HexNAc(4)Hex(5)	100	1.00E-17
A8K979	ERI1 exoribonuclease 2	NLSISTK	HexNAc(3)Hex(4)	100	1.00E-17
A8K979	ERI1 exoribonuclease 2	NLSISTK	HexNAc(2)Hex(6)	100	1.00E-17
A8MVW0	Protein FAM171A2	NGTGVIR	HexNAc(4)Hex(3)Fuc(2)	100	1.00E-17
A8MVW0	Protein FAM171A2	NGTGVIR	HexNAc(2)Hex(8)	10.807	1.93E-03
A8MVW0	Protein FAM171A2	ASVDVFGNR	HexNAc(2)Hex(7)	100	1.00E-17
A8MVW0	Protein FAM171A2	NGTGVIR	HexNAc(2)Hex(7)	29.732	3.98E-05
A8MVW0	Protein FAM171A2	NGTGVIR	HexNAc(2)Hex(6)	9.207	1.32E-03
A8MVW0	Protein FAM171A2	ASVDVFGNR	HexNAc(2)Hex(5)	20.871	1.76E-04
A8MVW0	Protein FAM171A2	NGTGVIR	HexNAc(2)Hex(5)	2.821	1.50E-02
A8MVW0	Protein FAM171A2	ASVDVFGNR	HexNAc(2)Hex(4)	100	1.00E-17
A8MWY0	UPF0577 protein KIAA1324-like	MALCTNNITDFTVK	HexNAc(2)Hex(5)	7.094	1.93E-03
O00469	Procollagen-lysine,2-oxoglutarate 5-dioxygenase 2	YNCSIESPR	HexNAc(2)Hex(5)	100	1.00E-17
O00533	Neural cell adhesion molecule L1-like protein	QNRSVR	HexNAc(7)Hex(4)Fuc(1)	100	1.00E-17
O00533	Neural cell adhesion molecule L1-like protein	LTVNSSNSIK	HexNAc(6)Hex(4)Fuc(2)	100	1.00E-17
O14525	Astrotactin-1	AAPIYELVTNNQTQR	HexNAc(2)Hex(8)	100	1.00E-17
O14656	Torsin-1A	GNVSACAR	HexNAc(2)Hex(5)	100	1.00E-17
O14672	Disintegrin and metalloproteinase domain-containing protein 10	INTTADEKDPTNPFR	HexNAc(2)Hex(7)	100	1.00E-17
O14786	Neuropilin-1	IGYSNNGSDWK	HexNAc(5)Hex(4)Fuc(2)	100	1.00E-17
O14786	Neuropilin-1	RGPECSQNYTTPSGVIK	HexNAc(3)Hex(6)NeuAc(1)	12.936	9.17E-05
O14786	Neuropilin-1	RGPECSQNYTTPSGVIK	HexNAc(3)Hex(6)Fuc(1)NeuAc(1)	100	1.00E-17
O14786	Neuropilin-1	RGPECSQNYTTPSGVIK	HexNAc(3)Hex(6)Fuc(1)	100	1.00E-17
O14786	Neuropilin-1	RGPECSQNYTTPSGVIK	HexNAc(3)Hex(6)	100	1.00E-17
O14786	Neuropilin-1	RGPECSQNYTTPSGVIK	HexNAc(3)Hex(5)NeuAc(1)	100	1.00E-17
O14786	Neuropilin-1	RGPECSQNYTTPSGVIK	HexNAc(3)Hex(5)	100	1.00E-17
O14786	Neuropilin-1	RGPECSQNYTTPSGVIK	HexNAc(3)Hex(4)NeuAc(1)	100	1.00E-17
O14786	Neuropilin-1	RGPECSQNYTTPSGVIK	HexNAc(2)Hex(5)	6.667	7.20E-03
O14917	Protocadherin-17	DSGAPAHLESNATVR	HexNAc(5)Hex(4)Fuc(2)	100	1.00E-17
O14917	Protocadherin-17	DSGAPAHLESNATVR	HexNAc(5)Hex(3)Fuc(1)	100	1.00E-17
O14917	Protocadherin-17	DGGSPPLNSTK	HexNAc(2)Hex(6)	100	1.00E-17
O14917	Protocadherin-17	DGGSPPLNSTK	HexNAc(2)Hex(5)	100	1.00E-17
O14920	Isoform 2 of Inhibitor of nuclear factor kappa-B kinase subunit beta	NNSCLSK	HexNAc(6)Hex(3)Fuc(1)	100	1.00E-17
O15031	Plexin-B2	NCSFQPER	HexNAc(5)Hex(4)Fuc(2)	100	1.00E-17
O15031	Plexin-B2	ALSNISLR	HexNAc(5)Hex(4)Fuc(2)	28.097	3.44E-04
O15031	Plexin-B2	SCVAVTSAQPQNMSR	HexNAc(5)Hex(4)Fuc(1)	100	1.00E-17
O15031	Plexin-B2	ALSNISLR*	HexNAc(5)Hex(3)Fuc(1)	100	1.00E-17
O15031	Plexin-B2	SCVAVTSAQPQNMSR	HexNAc(5)Hex(3)	100	1.00E-17
O15031	Plexin-B2	SCVAVTSAQPQNMSR	HexNAc(4)Hex(5)	8.604	1.52E-02
O15031	Plexin-B2	TEAGAFEYVPDPTFENFTGGVK	HexNAc(3)Hex(6)NeuAc(1)	3.21	3.89E-02
O15031	Plexin-B2	TEAGAFEYVPDPTFENFTGGVK	HexNAc(3)Hex(6)Fuc(1)	100	1.00E-17
O15031	Plexin-B2	TEAGAFEYVPDPTFENFTGGVK	HexNAc(3)Hex(5)NeuAc(1)	6.446	1.38E-02
O15031	Plexin-B2	EAESLQPMTVVGTDYVFHNDTK	HexNAc(2)Hex(7)	5.419	1.75E-02
O15031	Plexin-B2	SCVAVTSAQPQNMSR	HexNAc(2)Hex(6)	100	1.00E-17
O15031	Plexin-B2	EAESLQPMTVVGTDYVFHNDTK	HexNAc(2)Hex(6)	4.404	3.46E-02
O15031	Plexin-B2	SCVAVTSAQPQNMSR	HexNAc(2)Hex(5)	100	1.00E-17
O15031	Plexin-B2	EAESLQPMTVVGTDYVFHNDTK	HexNAc(2)Hex(5)	100	1.00E-17
O15031	Plexin-B2	LSHDANETLPLHLYVK	HexNAc(2)Hex(5)	6.892	1.05E-02
O15084	Isoform 3 of Serine/threonine-protein phosphatase 6 regulatory ankyrin repeat subunit A	LPQENK	HexNAc(3)Hex(4)	100	1.00E-17
O15372	Eukaryotic translation initiation factor 3 subunit H	NTSK	HexNAc(4)Hex(5)NeuAc(1)	100	1.00E-17
O15394	Neural cell adhesion molecule 2	NTTNLK	HexNAc(6)Hex(4)Fuc(2)	4.951	2.30E-02
O15394	Neural cell adhesion molecule 2	NTTNLK	HexNAc(6)Hex(3)Fuc(1)	100	1.00E-17

O15394	Neural cell adhesion molecule 2	NTTNLK	HexNAc(5)Hex(5)Fuc(3)	4.754	2.57E-02
O15394	Neural cell adhesion molecule 2	NTTNLK	HexNAc(5)Hex(4)Fuc(2)	40.163	2.20E-04
O15394	Neural cell adhesion molecule 2	SFNATAER	HexNAc(5)Hex(4)Fuc(2)	7.666	8.09E-03
O15394	Neural cell adhesion molecule 2	SFNATAERGEEMTFSCR	HexNAc(5)Hex(4)Fuc(2)	3.074	4.21E-02
O15394	Neural cell adhesion molecule 2	NTTNLK	HexNAc(5)Hex(3)Fuc(1)	100	1.00E-17
O15394	Neural cell adhesion molecule 2	SFNATAER*	HexNAc(5)Hex(3)Fuc(1)	100	1.00E-17
O15394	Neural cell adhesion molecule 2	NTTNLK	HexNAc(4)Hex(5)Fuc(2)	100	1.00E-17
O15394	Neural cell adhesion molecule 2	NTTNLK	HexNAc(4)Hex(5)Fuc(1)	15.38	8.87E-04
O15394	Neural cell adhesion molecule 2	NTTNLK	HexNAc(4)Hex(4)Fuc(1)	100	1.00E-17
O15394	Neural cell adhesion molecule 2	NTTNLK	HexNAc(4)Hex(3)Fuc(1)	100	1.00E-17
O15394	Neural cell adhesion molecule 2	YNCTATNHIGTR	HexNAc(2)Hex(6)	42.743	3.10E-06
O15394	Neural cell adhesion molecule 2	YNCTATNHIGTR	HexNAc(2)Hex(5)	29.273	2.62E-05
O43157	Plexin-B1	LTLNGSK	HexNAc(5)Hex(5)Fuc(3)	100	1.00E-17
O43157	Plexin-B1	LTLNGSK	HexNAc(5)Hex(4)Fuc(2)	100	1.00E-17
O43157	Plexin-B1	YTLDPNITSAGPTK	HexNAc(5)Hex(4)Fuc(2)	9.36	1.97E-03
O43157	Plexin-B1	LTLNGSK	HexNAc(5)Hex(4)Fuc(1)	100	1.00E-17
O43157	Plexin-B1	YTLDPNITSAGPTK	HexNAc(5)Hex(3)Fuc(1)	100	1.00E-17
O43157	Plexin-B1	LTLNGSK	HexNAc(5)Hex(3)Fuc(1)	100	1.00E-17
O43157	Plexin-B1	YTLDPNITSAGPTK	HexNAc(4)Hex(5)Fuc(1)NeuAc(1)	100	1.00E-17
O43157	Plexin-B1	LTLNGSK	HexNAc(4)Hex(5)Fuc(1)	100	1.00E-17
O43157	Plexin-B1	LTLNGSK	HexNAc(2)Hex(5)	9.198	9.81E-03
O43490	Prominin-1	EALENMNSTLK	HexNAc(5)Hex(3)	100	1.00E-17
O43493	Trans-Golgi network integral membrane protein 2	DVPNK	HexNAc(4)Hex(6)NeuAc(1)	11.553	5.61E-03
O43529	Carbohydrate sulfotransferase 10	NLSHTPVSK	HexNAc(2)Hex(9)	5.3	1.82E-02
O43639	Cytoplasmic protein NCK2	NAANR	HexNAc(4)Hex(3)NeuAc(1)	100	1.00E-17
O43657	Tetraspanin-6	QYNSTGDYR	HexNAc(5)Hex(5)Fuc(3)	100	1.00E-17
O43657	Tetraspanin-6	QYNSTGDYR	HexNAc(5)Hex(4)Fuc(1)	100	1.00E-17
O60242	Adhesion G protein-coupled receptor B3	NVTDTFK	HexNAc(2)Hex(5)	100	1.00E-17
O60242	Adhesion G protein-coupled receptor B3	NVTDTFKR	HexNAc(2)Hex(5)	100	1.00E-17
O60242	Adhesion G protein-coupled receptor B3	NYTVINSK	HexNAc(2)Hex(5)	100	1.00E-17
O60245	Isoform B of Protocadherin-7	NISYTLLPPSSNVR	HexNAc(2)Hex(5)	100	1.00E-17
O60271	C-Jun-amino-terminal kinase-interacting protein 4	VTDAPNK	HexNAc(5)Hex(3)Fuc(1)NeuAc(1)	4.238	3.11E-02
O60292	Signal-induced proliferation-associated 1-like protein 3	NVSFSR	HexNAc(6)Hex(3)Fuc(2)	100	1.00E-17
O60293	Zinc finger C3H1 domain-containing protein	NFSR	HexNAc(4)Hex(5)Fuc(3)	100	1.00E-17
O60486	Plexin-C1	TASTIANSSK	HexNAc(2)Hex(9)	100	1.00E-17
O60486	Plexin-C1	DVIQVSHVLNDTHMK	HexNAc(2)Hex(9)	100	1.00E-17
O60486	Plexin-C1	TNVTVK	HexNAc(2)Hex(8)	100	1.00E-17
O60486	Plexin-C1	TASTIANSSK	HexNAc(2)Hex(8)	100	1.00E-17
O60486	Plexin-C1	TNVTVK	HexNAc(2)Hex(7)	100	1.00E-17
O60486	Plexin-C1	ASNITMILK	HexNAc(2)Hex(7)	100	1.00E-17
O60486	Plexin-C1	DQAGNCTEPVSLAPPARPR	HexNAc(2)Hex(7)	6.748	1.09E-02
O60486	Plexin-C1	TNVTVK	HexNAc(2)Hex(6)	100	1.00E-17
O60486	Plexin-C1	NGTEVVSCHPQGSTAGVVYR	HexNAc(2)Hex(5)	100	1.00E-17
O60486	Plexin-C1	SNVIVTGANFTR	HexNAc(2)Hex(5)	100	1.00E-17
O60524	Nuclear export mediator factor NEMF	NETEPPK	HexNAc(3)Hex(3)	6.383	1.12E-02
O60637	Tetraspanin-3	TYNGTNPDAASR	HexNAc(6)Hex(3)Fuc(1)	100	1.00E-17
O60637	Tetraspanin-3	NQSVPLSCCR	HexNAc(4)Hex(3)Fuc(1)	100	1.00E-17
O60637	Tetraspanin-3	NQSVPLSCCR	HexNAc(3)Hex(6)Fuc(1)NeuAc(1)	100	1.00E-17
O60637	Tetraspanin-3	NQSVPLSCCR	HexNAc(3)Hex(6)Fuc(1)	3.027	3.95E-02
O60637	Tetraspanin-3	NQSVPLSCCR	HexNAc(3)Hex(6)	100	1.00E-17
O60637	Tetraspanin-3	NQSVPLSCCR	HexNAc(2)Hex(5)	13.395	1.18E-04
O60637	Tetraspanin-3	NQSVPLSCCR	HexNAc(2)Hex(4)	100	1.00E-17
O60637	Tetraspanin-3	NQSVPLSCCR	HexNAc(2)Hex(3)	4.655	3.32E-02
O75051	Plexin-A2	EHYLSSVNK	HexNAc(2)Hex(8)	100	1.00E-17
O75051	Plexin-A2	EHYLSSVNK	HexNAc(2)Hex(7)	100	1.00E-17
O75051	Plexin-A2	FYNCSAHQLCLSCVNSAFR	HexNAc(2)Hex(7)	4.505	1.66E-02
O75051	Plexin-A2	EHYLSSVNK	HexNAc(2)Hex(6)	41.219	1.17E-05
O75051	Plexin-A2	EHYLSSVNK	HexNAc(2)Hex(5)	100	1.00E-17
O75051	Plexin-A2	LTGNLTIQVAHK	HexNAc(2)Hex(5)	100	1.00E-17
O75054	Isoform 2 of Immunoglobulin superfamily member 3	LSQAQGNLSVLETR	HexNAc(6)Hex(3)Fuc(1)	100	1.00E-17
O75054	Isoform 2 of Immunoglobulin superfamily member 3	QVQLECVVLNR	HexNAc(5)Hex(4)Fuc(2)	100	1.00E-17
O75054	Isoform 2 of Immunoglobulin superfamily member 3	VQGNSTLLHITDLQAR	HexNAc(5)Hex(3)Fuc(1)	100	1.00E-17
O75054	Isoform 2 of Immunoglobulin superfamily member 3	NYNNTWTR	HexNAc(2)Hex(8)	100	1.00E-17
O75054	Isoform 2 of Immunoglobulin superfamily member 3	TLTLVENKPIQLNCSVK	HexNAc(2)Hex(8)	18.806	1.24E-04
O75054	Isoform 2 of Immunoglobulin superfamily member 3	NYNNTWTR	HexNAc(2)Hex(7)	100	1.00E-17
O75054	Isoform 2 of Immunoglobulin superfamily member 3	TLTLVENKPIQLNCSVK	HexNAc(2)Hex(7)	39.765	2.19E-05
O75054	Isoform 2 of Immunoglobulin superfamily member 3	VQGNSTLLHITDLQAR	HexNAc(2)Hex(7)	5.169	1.96E-02
O75054	Isoform 2 of Immunoglobulin superfamily member 3	NYNNTWTR	HexNAc(2)Hex(6)	100	1.00E-17

O75054	Isoform 2 of Immunoglobulin superfamily member 3	TLTLVENKPIQLNCSVK	HexNAc(2)Hex(6)	17.742	2.38E-04
O75054	Isoform 2 of Immunoglobulin superfamily member 3	VQGNSTLLHITDLQAR	HexNAc(2)Hex(5)	100	1.00E-17
O75054	Isoform 2 of Immunoglobulin superfamily member 3	NYNNTWTR	HexNAc(2)Hex(5)	36.763	4.10E-05
O75054	Isoform 2 of Immunoglobulin superfamily member 3	TLTLVENKPIQLNCSVK	HexNAc(2)Hex(5)	14.973	1.69E-04
O75054	Isoform 2 of Immunoglobulin superfamily member 3	QVQLECVVLNR	HexNAc(2)Hex(5)	11.786	6.60E-04
O75054	Isoform 2 of Immunoglobulin superfamily member 3	QVQLECVVLNR	HexNAc(2)Hex(4)	100	1.00E-17
O75093	Slit homolog 1 protein	ISCIHNDSFTGLR	HexNAc(5)Hex(3)Fuc(1)NeuAc(1)	100	1.00E-17
O75093	Slit homolog 1 protein	ISCIHNDSFTGLR	HexNAc(5)Hex(3)Fuc(1)	6.739	1.11E-02
O75093	Slit homolog 1 protein	ISCIHNDSFTGLR	HexNAc(2)Hex(9)	100	1.00E-17
O75116	Rho-associated protein kinase 2	NLTIK	HexNAc(4)Hex(7)	100	1.00E-17
O75116	Rho-associated protein kinase 2	NLTIK	HexNAc(2)Hex(5)	100	1.00E-17
O75179	Ankyrin repeat domain-containing protein 17	IGSSAPTTTAANTSLMGIK	HexNAc(2)Hex(4)	3.153	4.45E-02
O75326	Semaphorin-7A	NASVR	HexNAc(5)Hex(3)Fuc(1)	100	1.00E-17
O75449	Katanin p60 ATPase-containing subunit A1	EEKNK	HexNAc(4)Hex(5)Fuc(2)NeuAc(1)	100	1.00E-17
O75503	Ceroid-lipofuscinosis neuronal protein 5	NIETNYTR	HexNAc(5)Hex(4)	100	1.00E-17
O75503	Ceroid-lipofuscinosis neuronal protein 5	QDNETGIYYETWNVK	HexNAc(2)Hex(9)	3.851	7.38E-03
O75503	Ceroid-lipofuscinosis neuronal protein 5	NIETNYTR	HexNAc(2)Hex(6)	6.587	1.21E-02
O75503	Ceroid-lipofuscinosis neuronal protein 5	NIETNYTR	HexNAc(2)Hex(5)	20.716	1.33E-03
O75503	Ceroid-lipofuscinosis neuronal protein 5	NIETNYTR	HexNAc(2)Hex(3)	100	1.00E-17
O75592	E3 ubiquitin-protein ligase MYCBP2	TLPANR	HexNAc(6)Hex(4)NeuAc(1)	4.335	4.34E-02
O75882	Attractin	NHSCSEGQISIFR	HexNAc(5)Hex(4)Fuc(2)	100	1.00E-17
O75882	Attractin	CINQSICEK	HexNAc(5)Hex(3)Fuc(1)	100	1.00E-17
O75882	Attractin	GICNSSDVR	HexNAc(2)Hex(7)	100	1.00E-17
O75882	Attractin	CENLTTGK	HexNAc(2)Hex(5)	100	1.00E-17
O75882	Attractin	CINQSICEK	HexNAc(2)Hex(5)	100	1.00E-17
O75882	Attractin	MPSQAPTGNFYPQPLLNSSMCLEDSR	HexNAc(2)Hex(5)	100	1.00E-17
O75882	Attractin	GICNSSDVR	HexNAc(2)Hex(5)	8.706	1.46E-02
O75970	Multiple PDZ domain protein	NTSR	HexNAc(5)Hex(3)Fuc(1)NeuAc(1)	100	1.00E-17
O75970	Multiple PDZ domain protein	NGTLK	HexNAc(3)Hex(3)Fuc(2)	100	1.00E-17
O75976	Carboxypeptidase D	NVTVK	HexNAc(6)Hex(4)Fuc(2)	100	1.00E-17
O75976	Carboxypeptidase D	NVTVK	HexNAc(5)Hex(3)Fuc(1)	100	1.00E-17
O75976	Carboxypeptidase D	NVTVK	HexNAc(4)Hex(5)Fuc(1)	5.942	1.50E-02
O75976	Carboxypeptidase D	NVTVK	HexNAc(4)Hex(3)Fuc(1)	100	1.00E-17
O75976	Carboxypeptidase D	NNSNNFDLNR	HexNAc(3)Hex(6)Fuc(1)	4.397	3.65E-02
O75976	Carboxypeptidase D	NNSNNFDLNR	HexNAc(2)Hex(5)	100	1.00E-17
O75976	Carboxypeptidase D	DLDTDFTNNASQPETK	HexNAc(2)Hex(5)	2.843	1.68E-02
O76024	Wolframin	CLYGEAYPACSPGNTSTAEEELCR	HexNAc(2)Hex(5)	100	1.00E-17
O94779	Contactin-5	ILNASK	HexNAc(5)Hex(4)Fuc(2)	100	1.00E-17
O94779	Contactin-5	ILNASK	HexNAc(2)Hex(5)	100	1.00E-17
O94806	Serine/threonine-protein kinase D3	IPNNCSGVR	HexNAc(4)Hex(5)Fuc(2)	100	1.00E-17
O94813	Slit homolog 2 protein	LDLNGNNITR	HexNAc(2)Hex(8)	100	1.00E-17
O94813	Slit homolog 2 protein	IITNLSK	HexNAc(2)Hex(6)	100	1.00E-17
O94856	Neurofascin	EAWNNVTVWGSR	HexNAc(5)Hex(5)Fuc(3)	100	1.00E-17
O94856	Neurofascin	QIVENFSPNQTK	HexNAc(5)Hex(5)Fuc(3)	6.682	1.33E-02
O94856	Neurofascin	QIVENFSPNQTK	HexNAc(5)Hex(4)Fuc(2)	28.886	4.52E-05
O94856	Neurofascin	EAWNNVTVWGSR	HexNAc(5)Hex(4)Fuc(2)	10.872	9.47E-04
O94856	Neurofascin	QIVENFSPNQTK	HexNAc(5)Hex(4)Fuc(1)NeuAc(1)	100	1.00E-17
O94856	Neurofascin	QIVENFSPNQTK	HexNAc(5)Hex(3)Fuc(1)NeuAc(1)	9.264	6.90E-03
O94856	Neurofascin	NNMEITWTPMNATSAFGPNLR	HexNAc(5)Hex(3)Fuc(1)	100	1.00E-17
O94856	Neurofascin	EAWNNVTVWGSR*	HexNAc(5)Hex(3)Fuc(1)	100	1.00E-17
O94856	Neurofascin	QIVENFSPNQTK	HexNAc(5)Hex(3)Fuc(1)	8.54	1.42E-03
O94856	Neurofascin	QIVENFSPNQTK	HexNAc(4)Hex(5)Fuc(2)NeuAc(1)	100	1.00E-17
O94856	Neurofascin	VILYNR	HexNAc(2)Hex(8)	100	1.00E-17
O94856	Neurofascin	WANITWK	HexNAc(2)Hex(7)	100	1.00E-17
O94856	Neurofascin	VILYNR	HexNAc(2)Hex(7)	63.213	1.05E-06
O94856	Neurofascin	YVAFNGTK	HexNAc(2)Hex(7)	22.367	4.49E-05
O94856	Neurofascin	YVAFNGTK	HexNAc(2)Hex(6)	37.227	7.10E-06
O94856	Neurofascin	VILYNR	HexNAc(2)Hex(6)	15.8	1.26E-04
O94856	Neurofascin	WANITWK	HexNAc(2)Hex(6)	9.663	4.90E-03
O94856	Neurofascin	VILYNR	HexNAc(2)Hex(5)	100	1.00E-17
O94856	Neurofascin	WANITWK	HexNAc(2)Hex(5)	100	1.00E-17
O94856	Neurofascin	YVAFNGTK	HexNAc(2)Hex(5)	100	1.00E-17
O94874	E3 UFM1-protein ligase 1	PINK	HexNAc(5)Hex(4)NeuAc(2)	100	1.00E-17
O94874	E3 UFM1-protein ligase 1	PINK	HexNAc(4)Hex(6)Fuc(2)	100	1.00E-17
O94874	E3 UFM1-protein ligase 1	PINK	HexNAc(4)Hex(5)Fuc(2)	100	1.00E-17
O94885	SAM and SH3 domain-containing protein 1	LVNSTR	HexNAc(5)Hex(4)NeuAc(1)	100	1.00E-17
O94905	Erlin-1	NYTADYDK	HexNAc(2)Hex(5)	100	1.00E-17
O95297	Myelin protein zero-like protein 1	EIFVANGTQGK	HexNAc(6)Hex(5)Fuc(1)NeuAc(2)	100	1.00E-17
O95297	Myelin protein zero-like protein 1	DASINIENMQFIHNGTYICDVK	HexNAc(6)Hex(4)NeuAc(1)	3.532	4.65E-02
O95297	Myelin protein zero-like protein 1	EIFVANGTQGK	HexNAc(3)Hex(6)NeuAc(1)	100	1.00E-17
O95297	Myelin protein zero-like protein 1	EIFVANGTQGK	HexNAc(3)Hex(6)Fuc(1)NeuAc(1)	6.232	1.06E-02

O95297	Myelin protein zero-like protein 1	EIFVANGTQGK	HexNAc(3)Hex(6)Fuc(1)	100	1.00E-17
O95297	Myelin protein zero-like protein 1	DASINIENMQFIHNGTYICDVK	HexNAc(3)Hex(5)NeuAc(1)	100	1.00E-17
O95297	Myelin protein zero-like protein 1	EIFVANGTQGK	HexNAc(3)Hex(5)Fuc(1)	7.632	1.95E-02
O95490	Isoform 5 of Adhesion G protein-coupled receptor L2	SLGQFLSTENATIK	HexNAc(2)Hex(7)	2.12	3.49E-02
O95631	Netrin-1	SCHLCNASDPK	HexNAc(2)Hex(5)	100	1.00E-17
O95631	Netrin-1	TCNQTTGQCPCK	HexNAc(2)Hex(5)	100	1.00E-17
O95674	Phosphatidate cytidylyltransferase 2	CFVCPVEYNNDTNSFTVDCEPSDLFR	HexNAc(2)Hex(6)	11.886	2.47E-03
O95674	Phosphatidate cytidylyltransferase 2	CFVCPVEYNNDTNSFTVDCEPSDLFR	HexNAc(2)Hex(5)	5.752	1.87E-02
O95754	Semaphorin-4F	TEVTQVNTTNCGR	HexNAc(2)Hex(6)	100	1.00E-17
O95819	Isoform 6 of Mitogen-activated protein kinase kinase kinase kinase 4	NSTSSIEPR	HexNAc(3)Hex(5)	100	1.00E-17
O95970	Isoform 2 of Leucine-rich glioma-inactivated protein 1	NYDNITVLR	HexNAc(2)Hex(9)	100	1.00E-17
P02461	Collagen alpha-1(III) chain	ASQNITYHCK	HexNAc(2)Hex(9)	12.752	4.21E-03
P02786	Transferrin receptor protein 1	KDFEDLYTPVNGSIVIVR	HexNAc(4)Hex(5)Fuc(4)	100	1.00E-17
P02786	Transferrin receptor protein 1	QNNGAFNETLFR	HexNAc(2)Hex(7)	100	1.00E-17
P02787	Serotransferrin	CGLVPVLAENYNK	HexNAc(4)Hex(6)Fuc(1)NeuAc(1)	4.397	3.18E-02
P02787	Serotransferrin	CGLVPVLAENYNK	HexNAc(4)Hex(5)NeuAc(2)	4.2	5.99E-03
P02787	Serotransferrin	QQQHLFGSNVTDCSGNFCLFR	HexNAc(4)Hex(5)NeuAc(2)	2.942	2.04E-02
P04066	Tissue alpha-L-fucosidase	NTTSVWYTSK	HexNAc(2)Hex(5)	3.237	3.25E-02
P04216	Thy-1 membrane glycoprotein	TNFTSK	HexNAc(6)Hex(4)Fuc(2)	100	1.00E-17
P04216	Thy-1 membrane glycoprotein	TNFTSK	HexNAc(5)Hex(5)Fuc(1)	100	1.00E-17
P04216	Thy-1 membrane glycoprotein	TNFTSK	HexNAc(5)Hex(4)Fuc(2)	10.434	8.35E-03
P04216	Thy-1 membrane glycoprotein	TNFTSK	HexNAc(5)Hex(3)Fuc(1)	100	1.00E-17
P04216	Thy-1 membrane glycoprotein	TNFTSK	HexNAc(4)Hex(6)Fuc(2)	100	1.00E-17
P04216	Thy-1 membrane glycoprotein	HENTSSSPIQYEFSLTR	HexNAc(4)Hex(5)Fuc(1)	3.514	3.36E-02
P04216	Thy-1 membrane glycoprotein	TNFTSK	HexNAc(3)Hex(6)Fuc(1)NeuAc(1)	100	1.00E-17
P04216	Thy-1 membrane glycoprotein	TNFTSK	HexNAc(3)Hex(6)Fuc(1)	5.155	2.59E-02
P04216	Thy-1 membrane glycoprotein	TNFTSK	HexNAc(2)Hex(5)	8.78	8.17E-04
P04626	Receptor tyrosine-protein kinase erbB-2	ENTSPK	HexNAc(7)Hex(3)	100	1.00E-17
P05026	Sodium/potassium-transporting ATPase subunit beta-1	NESLETYPVMK	HexNAc(8)Hex(9)Fuc(1)	100	1.00E-17
P05026	Sodium/potassium-transporting ATPase subunit beta-1	NESLETYPVMK	HexNAc(8)Hex(9)	100	1.00E-17
P05026	Sodium/potassium-transporting ATPase subunit beta-1	LEWLGNCSGLNDETYGYK	HexNAc(7)Hex(8)	100	1.00E-17
P05026	Sodium/potassium-transporting ATPase subunit beta-1	NESLETYPVMK	HexNAc(6)Hex(7)NeuAc(4)	100	1.00E-17
P05026	Sodium/potassium-transporting ATPase subunit beta-1	NESLETYPVMK	HexNAc(6)Hex(7)NeuAc(3)	3.439	4.13E-02
P05026	Sodium/potassium-transporting ATPase subunit beta-1	NESLETYPVMK	HexNAc(6)Hex(7)Fuc(4)NeuAc(1)	4.543	2.75E-02
P05026	Sodium/potassium-transporting ATPase subunit beta-1	NESLETYPVMK	HexNAc(6)Hex(7)Fuc(1)NeuAc(4)	8.94	1.79E-03
P05026	Sodium/potassium-transporting ATPase subunit beta-1	NESLETYPVMK	HexNAc(6)Hex(7)Fuc(1)NeuAc(3)	9.483	7.97E-04
P05026	Sodium/potassium-transporting ATPase subunit beta-1	LEWLGNCSGLNDETYGYK	HexNAc(6)Hex(6)Fuc(3)	5.902	1.27E-02
P05026	Sodium/potassium-transporting ATPase subunit beta-1	LEWLGNCSGLNDETYGYK	HexNAc(6)Hex(6)Fuc(2)	100	1.00E-17
P05026	Sodium/potassium-transporting ATPase subunit beta-1	LEWLGNCSGLNDETYGYK	HexNAc(6)Hex(6)Fuc(1)NeuAc(1)	3.678	4.92E-02
P05026	Sodium/potassium-transporting ATPase subunit beta-1	LEWLGNCSGLNDETYGYK	HexNAc(6)Hex(5)Fuc(2)	4.641	4.21E-02
P05026	Sodium/potassium-transporting ATPase subunit beta-1	LEWLGNCSGLNDETYGYK	HexNAc(6)Hex(5)Fuc(1)NeuAc(2)	6.935	1.10E-02
P05026	Sodium/potassium-transporting ATPase subunit beta-1	LEWLGNCSGLNDETYGYK	HexNAc(6)Hex(5)Fuc(1)NeuAc(1)	3.228	1.55E-02
P05026	Sodium/potassium-transporting ATPase subunit beta-1	LEWLGNCSGLNDETYGYK	HexNAc(6)Hex(5)Fuc(1)	100	1.00E-17
P05026	Sodium/potassium-transporting ATPase subunit beta-1	LEWLGNCSGLNDETYGYK	HexNAc(5)Hex(6)NeuAc(3)	100	1.00E-17
P05026	Sodium/potassium-transporting ATPase subunit beta-1	LEWLGNCSGLNDETYGYK	HexNAc(5)Hex(6)Fuc(2)	100	1.00E-17
P05026	Sodium/potassium-transporting ATPase subunit beta-1	LEWLGNCSGLNDETYGYK	HexNAc(5)Hex(6)Fuc(1)NeuAc(2)	100	1.00E-17
P05026	Sodium/potassium-transporting ATPase subunit beta-1	LEWLGNCSGLNDETYGYK	HexNAc(5)Hex(6)Fuc(1)NeuAc(1)	100	1.00E-17
P05026	Sodium/potassium-transporting ATPase subunit beta-1	LEWLGNCSGLNDETYGYK	HexNAc(5)Hex(5)NeuAc(2)	7.953	1.64E-02
P05026	Sodium/potassium-transporting ATPase subunit beta-1	LEWLGNCSGLNDETYGYK	HexNAc(5)Hex(5)NeuAc(1)	3.021	3.25E-02
P05026	Sodium/potassium-transporting ATPase subunit beta-1	LEWLGNCSGLNDETYGYK	HexNAc(5)Hex(5)Fuc(3)	9.721	4.10E-03
P05026	Sodium/potassium-transporting ATPase subunit beta-1	LEWLGNCSGLNDETYGYK	HexNAc(5)Hex(5)Fuc(1)NeuAc(2)	100	1.00E-17
P05026	Sodium/potassium-transporting ATPase subunit beta-1	LEWLGNCSGLNDETYGYK	HexNAc(5)Hex(5)Fuc(1)NeuAc(1)	7.334	1.73E-02
P05026	Sodium/potassium-transporting ATPase subunit beta-1	LEWLGNCSGLNDETYGYK	HexNAc(5)Hex(4)NeuAc(1)	21.457	1.45E-03
P05026	Sodium/potassium-transporting ATPase subunit beta-1	LEWLGNCSGLNDETYGYK	HexNAc(5)Hex(4)Fuc(2)	21.457	1.45E-03
P05026	Sodium/potassium-transporting ATPase subunit beta-1	LEWLGNCSGLNDETYGYK	HexNAc(5)Hex(3)Fuc(1)	100	1.00E-17
P05026	Sodium/potassium-transporting ATPase subunit beta-1	NESLETYPVMK	HexNAc(2)Hex(5)	100	1.00E-17
P05156	Complement factor I	FLNNGTCTAEGK	HexNAc(2)Hex(9)	100	1.00E-17
P05156	Complement factor I	LISNCSK	HexNAc(2)Hex(9)	100	1.00E-17

P05156	Complement factor I	LISNCSK	HexNAc(2)Hex(8)	100	1.00E-17
P05556	Isoform 5 of Integrin beta-1	LRNPCTSEQNCTSPFSYK	HexNAc(6)Hex(7)Fuc(1)NeuAc(1)	100	1.00E-17
P05556	Isoform 5 of Integrin beta-1	NPCTSEQNCTSPFSYK	HexNAc(6)Hex(6)Fuc(1)	100	1.00E-17
P05556	Isoform 5 of Integrin beta-1	KDTCTQECSYFNITK	HexNAc(5)Hex(4)Fuc(2)	100	1.00E-17
P05556	Isoform 5 of Integrin beta-1	LRNPCTSEQNCTSPFSYK	HexNAc(4)Hex(5)Fuc(1)	100	1.00E-17
P05556	Isoform 5 of Integrin beta-1	NVTR	HexNAc(3)Hex(5)	100	1.00E-17
P05556	Isoform 5 of Integrin beta-1	NVTNR	HexNAc(3)Hex(3)Fuc(1)	100	1.00E-17
P05556	Isoform 5 of Integrin beta-1	NGVNGTGENGRK	HexNAc(2)Hex(5)	6.433	1.13E-02
P05556	Isoform 5 of Integrin beta-1	NPCTSEQNCTSPFSYK	HexNAc(2)Hex(5)	3.904	6.96E-03
P06753	Isoform 5 of Tropomyosin alpha-3 chain	NVTNNLK	HexNAc(2)Hex(6)	100	1.00E-17
P06756	Integrin alpha-V	NMTISR	HexNAc(6)Hex(4)NeuAc(1)	9.449	1.47E-02
P06756	Integrin alpha-V	ENQNHSYSLK	HexNAc(5)Hex(6)Fuc(1)	100	1.00E-17
P06756	Integrin alpha-V	NMTISR	HexNAc(5)Hex(3)Fuc(1)	100	1.00E-17
P06756	Integrin alpha-V	ENQNHSYSLK	HexNAc(4)Hex(5)Fuc(2)	100	1.00E-17
P06756	Integrin alpha-V	ENQNHSYSLK	HexNAc(3)Hex(6)Fuc(1)	100	1.00E-17
P06756	Integrin alpha-V	ANTTQPGIVEGGQVLK	HexNAc(3)Hex(6)	100	1.00E-17
P06756	Integrin alpha-V	ENQNHSYSLK	HexNAc(3)Hex(5)NeuAc(1)	100	1.00E-17
P06756	Integrin alpha-V	ENQNHSYSLK	HexNAc(3)Hex(5)Fuc(1)	100	1.00E-17
P06756	Integrin alpha-V	ENQNHSYSLK	HexNAc(3)Hex(4)Fuc(1)	100	1.00E-17
P06756	Integrin alpha-V	ANTTQPGIVEGGQVLK	HexNAc(2)Hex(7)	100	1.00E-17
P06756	Integrin alpha-V	ANTTQPGIVEGGQVLK	HexNAc(2)Hex(5)	3.46	3.16E-02
P06865	Beta-hexosaminidase subunit alpha	SAEGTFFINK	HexNAc(2)Hex(7)	100	1.00E-17
P07195	L-lactate dehydrogenase B chain	NISR	HexNAc(7)Hex(3)	13.684	2.08E-03
P07602	Isoform Sap-mu-9 of Prosaposin	TCDWLPKPNMSASCK	HexNAc(2)Hex(5)	100	1.00E-17
P07602	Isoform Sap-mu-9 of Prosaposin	TCDWLPKPNMSASCK	HexNAc(2)Hex(4)	3.805	1.21E-02
P07602	Isoform Sap-mu-9 of Prosaposin	TCDWLPKPNMSASCK	HexNAc(2)Hex(3)	100	1.00E-17
P08069	Insulin-like growth factor 1 receptor	NITR	HexNAc(6)Hex(5)Fuc(1)NeuAc(1)	100	1.00E-17
P08069	Insulin-like growth factor 1 receptor	LNPGNYTAR	HexNAc(2)Hex(5)	6.83	6.89E-04
P08195	4F2 cell-surface antigen heavy chain	DASSFLAEWQNITK	HexNAc(6)Hex(7)Fuc(1)NeuAc(3)	100	1.00E-17
P08581	Isoform 2 of Hepatocyte growth factor receptor	NFTVACQHR	HexNAc(2)Hex(5)	100	1.00E-17
P08648	Integrin alpha-5	VTGLNCTTNHPINPK	HexNAc(5)Hex(5)Fuc(1)	100	1.00E-17
P08648	Integrin alpha-5	VTGLNCTTNHPINPK	HexNAc(5)Hex(4)Fuc(2)	100	1.00E-17
P08648	Integrin alpha-5	TEKEPLSDPVGTCYLSTDNFTR	HexNAc(5)Hex(4)Fuc(1)	100	1.00E-17
P08648	Integrin alpha-5	VTGLNCTTNHPINPK	HexNAc(4)Hex(5)Fuc(1)NeuAc(1)	100	1.00E-17
P08962	CD63 antigen	NNHTASILDR	HexNAc(6)Hex(6)Fuc(1)NeuAc(2)	100	1.00E-17
P08962	CD63 antigen	NNHTASILDR	HexNAc(6)Hex(6)Fuc(1)NeuAc(1)	100	1.00E-17
P08962	CD63 antigen	NNHTASILDR	HexNAc(6)Hex(6)Fuc(1)	100	1.00E-17
P08962	CD63 antigen	NNHTASILDR	HexNAc(6)Hex(5)Fuc(1)NeuAc(2)	100	1.00E-17
P08962	CD63 antigen	NNHTASILDR	HexNAc(6)Hex(5)Fuc(1)NeuAc(1)	100	1.00E-17
P08962	CD63 antigen	NNHTASILDR	HexNAc(5)Hex(6)NeuAc(3)	2.601	3.66E-02
P08962	CD63 antigen	NNHTASILDR	HexNAc(5)Hex(5)Fuc(3)	100	1.00E-17
P08962	CD63 antigen	NNHTASILDR	HexNAc(5)Hex(5)Fuc(2)	100	1.00E-17
P08962	CD63 antigen	NNHTASILDR	HexNAc(5)Hex(4)Fuc(2)	100	1.00E-17
P08962	CD63 antigen	NNHTASILDR	HexNAc(3)Hex(6)Fuc(1)NeuAc(1)	4.288	1.75E-02
P08962	CD63 antigen	NNHTASILDR	HexNAc(3)Hex(6)Fuc(1)	100	1.00E-17
P08962	CD63 antigen	NNHTASILDR	HexNAc(3)Hex(3)Fuc(1)	100	1.00E-17
P08962	CD63 antigen	NNHTASILDR	HexNAc(2)Hex(5)	11.657	2.55E-04
P0C7U0	Protein ELFN1	MYTLEHFNNSK	HexNAc(2)Hex(7)	5.329	1.10E-02
P0C7U0	Protein ELFN1	MYTLEHFNNSK	HexNAc(2)Hex(6)	100	1.00E-17
P0C7U0	Protein ELFN1	MYTLEHFNNSK	HexNAc(2)Hex(5)	100	1.00E-17
P10253	Lysosomal alpha-glucosidase	QVVENMTR	HexNAc(2)Hex(7)	9.75	2.92E-03
P10253	Lysosomal alpha-glucosidase	QVVENMTR	HexNAc(2)Hex(6)	100	1.00E-17
P10253	Lysosomal alpha-glucosidase	QVVENMTR	HexNAc(2)Hex(5)	4.216	1.28E-02
P10586	Receptor-type tyrosine-protein phosphatase F	VEVEPLNSTAVHVYWK	HexNAc(5)Hex(3)Fuc(1)	10.191	6.81E-03
P10586	Receptor-type tyrosine-protein phosphatase F	VEVEPLNSTAVHVYWK	HexNAc(2)Hex(9)	4.281	3.39E-02
P10909	Isoform 2 of Clusterin	HNSTGCLR	HexNAc(2)Hex(5)	7.673	8.71E-03
P11117	Lysosomal acid phosphatase	NLTLMATTSQLPK	HexNAc(2)Hex(9)	2.038	3.83E-02
P11117	Lysosomal acid phosphatase	NESDKAPWPLSLPGCPHR	HexNAc(2)Hex(7)	3.627	4.72E-02
P11117	Lysosomal acid phosphatase	NLTLMATTSQLPK	HexNAc(2)Hex(7)	3.201	2.62E-02
P11142	Heat shock cognate 71 kDa protein	NTTIPTK	HexNAc(4)Hex(6)	100	1.00E-17
P11279	Lysosome-associated membrane glycoprotein 1	NATR	HexNAc(4)Hex(5)Fuc(1)NeuAc(1)	100	1.00E-17
P11279	Lysosome-associated membrane glycoprotein 1	SSCGKENTSDPSLVIAFGR	HexNAc(4)Hex(5)Fuc(1)	100	1.00E-17
P11279	Lysosome-associated membrane glycoprotein 1	LLNINPNK	HexNAc(4)Hex(5)	100	1.00E-17
P11279	Lysosome-associated membrane glycoprotein 1	ENTSDPSLVIAFGR	HexNAc(2)Hex(5)	5.246	8.66E-03
P11717	Cation-independent mannose-6-phosphate receptor	GYPCGGNK	HexNAc(5)Hex(5)Fuc(3)	100	1.00E-17
P11717	Cation-independent mannose-6-phosphate receptor	GYPCGGNK	HexNAc(5)Hex(4)Fuc(2)	100	1.00E-17
P11717	Cation-independent mannose-6-phosphate receptor	GYPCGGNK	HexNAc(5)Hex(3)Fuc(1)	100	1.00E-17
P11717	Cation-independent mannose-6-phosphate receptor	NGSSIVDLSPLIHR	HexNAc(3)Hex(6)Fuc(1)	100	1.00E-17
P11717	Cation-independent mannose-6-phosphate receptor	NGSSIVDLSPLIHR	HexNAc(2)Hex(9)	100	1.00E-17
P11717	Cation-independent mannose-6-phosphate receptor	MNFTGGDTCHK	HexNAc(2)Hex(6)	100	1.00E-17
P11717	Cation-independent mannose-6-phosphate receptor	MNFTGGDTCHK	HexNAc(2)Hex(5)	100	1.00E-17

P11717	Cation-independent mannose-6-phosphate receptor	GYPCGGNK	HexNAc(2)Hex(5)	100	1.00E-17
P11717	Cation-independent mannose-6-phosphate receptor	SLLEFNTTVSCDQQGTNHR	HexNAc(2)Hex(5)	3.685	3.18E-02
P11717	Cation-independent mannose-6-phosphate receptor	TNITLVCKPGDLESAPVLR	HexNAc(2)Hex(5)	2.948	1.22E-02
P12109	Collagen alpha-1(VI) chain	GAPGINGTK	HexNAc(2)Hex(9)	100	1.00E-17
P12109	Collagen alpha-1(VI) chain	GAPGINGTK	HexNAc(2)Hex(8)	100	1.00E-17
P12109	Collagen alpha-1(VI) chain	GAPGINGTK	HexNAc(2)Hex(6)	100	1.00E-17
P12109	Collagen alpha-1(VI) chain	RNFTAADWGQSR	HexNAc(2)Hex(5)	100	1.00E-17
P13473	Isoform LAMP-2C of Lysosome-associated membrane glycoprotein 2	LNSSTIK	HexNAc(6)Hex(6)	100	1.00E-17
P13473	Isoform LAMP-2C of Lysosome-associated membrane glycoprotein 2	LNSSTIK	HexNAc(6)Hex(5)	100	1.00E-17
P13473	Isoform LAMP-2C of Lysosome-associated membrane glycoprotein 2	VASVININPNTTHSTGSCR	HexNAc(5)Hex(6)Fuc(1)NeuAc(2)	100	1.00E-17
P13473	Isoform LAMP-2C of Lysosome-associated membrane glycoprotein 2	LNSSTIK	HexNAc(5)Hex(5)Fuc(1)	100	1.00E-17
P13473	Isoform LAMP-2C of Lysosome-associated membrane glycoprotein 2	LNSSTIK	HexNAc(5)Hex(4)Fuc(1)	100	1.00E-17
P13473	Isoform LAMP-2C of Lysosome-associated membrane glycoprotein 2	LNSSTIK	HexNAc(5)Hex(4)	100	1.00E-17
P13473	Isoform LAMP-2C of Lysosome-associated membrane glycoprotein 2	LNSSTIK	HexNAc(4)Hex(4)NeuAc(1)	100	1.00E-17
P13473	Isoform LAMP-2C of Lysosome-associated membrane glycoprotein 2	LNSSTIK	HexNAc(4)Hex(3)Fuc(1)	100	1.00E-17
P13473	Isoform LAMP-2C of Lysosome-associated membrane glycoprotein 2	LNSSTIK	HexNAc(4)Hex(3)	100	1.00E-17
P13473	Isoform LAMP-2C of Lysosome-associated membrane glycoprotein 2	VQPFNVTQGK	HexNAc(3)Hex(6)Fuc(1)	100	1.00E-17
P13473	Isoform LAMP-2C of Lysosome-associated membrane glycoprotein 2	VASVININPNTTHSTGSCR	HexNAc(2)Hex(5)	100	1.00E-17
P13473	Isoform LAMP-2C of Lysosome-associated membrane glycoprotein 2	LNSSTIK	HexNAc(2)Hex(5)	100	1.00E-17
P13473	Isoform LAMP-2C of Lysosome-associated membrane glycoprotein 2	VQPFNVTQGK	HexNAc(2)Hex(5)	2.084	3.82E-02
P13473	Isoform LAMP-2C of Lysosome-associated membrane glycoprotein 2	LNSSTIK	HexNAc(2)Hex(4)	100	1.00E-17
P13473	Isoform LAMP-2C of Lysosome-associated membrane glycoprotein 2	LNSSTIK	HexNAc(2)Hex(3)	100	1.00E-17
P13591	Neural cell adhesion molecule 1	DGQLLPSSNYSNIK	HexNAc(6)Hex(5)Fuc(2)	100	1.00E-17
P13591	Neural cell adhesion molecule 1	DGQLLPSSNYSNIK	HexNAc(6)Hex(5)Fuc(1)NeuAc(1)	100	1.00E-17
P13591	Neural cell adhesion molecule 1	NISSEEK	HexNAc(6)Hex(4)Fuc(2)	100	1.00E-17
P13591	Neural cell adhesion molecule 1	DGQLLPSSNYSNIK	HexNAc(6)Hex(4)Fuc(2)	100	1.00E-17
P13591	Neural cell adhesion molecule 1	DGQLLPSSNYSNIK	HexNAc(5)Hex(6)Fuc(2)NeuAc(1)	100	1.00E-17
P13591	Neural cell adhesion molecule 1	DGQLLPSSNYSNIK	HexNAc(5)Hex(4)Fuc(2)	7.794	1.02E-02
P13591	Neural cell adhesion molecule 1	DGQLLPSSNYSNIK	HexNAc(5)Hex(4)Fuc(1)	4.25	3.11E-02
P13591	Neural cell adhesion molecule 1	NISSEEK	HexNAc(5)Hex(3)Fuc(1)	100	1.00E-17
P13591	Neural cell adhesion molecule 1	DGQLLPSSNYSNIK	HexNAc(5)Hex(3)Fuc(1)	8.412	8.64E-03
P13591	Neural cell adhesion molecule 1	IYNTPSASYLEVTPDSENDFGNYNCTAVNR	HexNAc(5)Hex(3)	100	1.00E-17
P13591	Neural cell adhesion molecule 1	DGQLLPSSNYSNIK	HexNAc(2)Hex(8)	100	1.00E-17
P13591	Neural cell adhesion molecule 1	DGQLLPSSNYSNIK	HexNAc(2)Hex(7)	100	1.00E-17
P13591	Neural cell adhesion molecule 1	DGQLLPSSNYSNIK	HexNAc(2)Hex(5)	100	1.00E-17
P13591	Neural cell adhesion molecule 1	IYNTPSASYLEVTPDSENDFGNYNCTAVNR	HexNAc(2)Hex(5)	4.029	2.51E-02
P13674	Prolyl 4-hydroxylase subunit alpha-1	DMSDGFISNLTIQR	HexNAc(2)Hex(5)	100	1.00E-17
P13987	CD59 glycoprotein	TAVNCSSDFDACLITK	HexNAc(5)Hex(5)NeuAc(2)	4.916	3.10E-02
P13987	CD59 glycoprotein	TAVNCSSDFDACLITK	HexNAc(5)Hex(5)Fuc(3)	100	1.00E-17
P13987	CD59 glycoprotein	TAVNCSSDFDACLITK	HexNAc(5)Hex(5)Fuc(1)NeuAc(1)	100	1.00E-17
P13987	CD59 glycoprotein	TAVNCSSDFDACLITK	HexNAc(5)Hex(4)Fuc(2)	3.398	1.48E-02
P13987	CD59 glycoprotein	TAVNCSSDFDACLITK	HexNAc(5)Hex(3)Fuc(1)	9.987	6.19E-04
P14314	Glucosidase 2 subunit beta	DGSDEPGTAACPNGSFHCTNTGYK	HexNAc(2)Hex(6)	100	1.00E-17
P14314	Glucosidase 2 subunit beta	DGSDEPGTAACPNGSFHCTNTGYKPLYIPSNR	HexNAc(2)Hex(5)	11.962	2.00E-03
P14314	Glucosidase 2 subunit beta	YEQGTGCWQGPNR	HexNAc(2)Hex(5)	3.488	7.27E-03
P14384	Carboxypeptidase M	TVAQNYSSVTHLHSIGK	HexNAc(4)Hex(5)Fuc(1)NeuAc(1)	100	1.00E-17
P14415	Sodium/potassium-transporting ATPase subunit beta-2	ACQFNR	HexNAc(4)Hex(6)	100	1.00E-17
P14415	Sodium/potassium-transporting ATPase subunit beta-2	ACQFNR	HexNAc(4)Hex(5)	100	1.00E-17
P14415	Sodium/potassium-transporting ATPase subunit beta-2	FHVNYTQPLVAVK	HexNAc(2)Hex(9)	2.642	1.49E-02
P14415	Sodium/potassium-transporting ATPase subunit beta-2	ACQFNR	HexNAc(2)Hex(8)	100	1.00E-17
P14415	Sodium/potassium-transporting ATPase subunit beta-2	FHVNYTQPLVAVK	HexNAc(2)Hex(8)	68.664	1.96E-07
P14415	Sodium/potassium-transporting ATPase subunit beta-2	FLEPYNDSIQAQK	HexNAc(2)Hex(8)	12.166	1.61E-03
P14415	Sodium/potassium-transporting ATPase subunit beta-2	RACQFNR	HexNAc(2)Hex(7)	100	1.00E-17
P14415	Sodium/potassium-transporting ATPase subunit beta-2	FLEPYNDSIQAQK	HexNAc(2)Hex(7)	55.596	4.97E-07
P14415	Sodium/potassium-transporting ATPase subunit beta-2	FHVNYTQPLVAVK	HexNAc(2)Hex(7)	26.547	1.02E-05
P14415	Sodium/potassium-transporting ATPase subunit beta-2	ACQFNR	HexNAc(2)Hex(7)	18.909	1.04E-03
P14415	Sodium/potassium-transporting ATPase subunit beta-2	RACQFNR	HexNAc(2)Hex(6)	100	1.00E-17
P14415	Sodium/potassium-transporting ATPase subunit beta-2	TQLGNCSGIGDSTHYGYSTGQPCVFIK	HexNAc(2)Hex(6)	41.955	4.31E-05

P14415	Sodium/potassium-transporting ATPase subunit beta-2	FLEPYNDSIQAQK	HexNAc(2)Hex(6)	29.542	6.74E-06
P14415	Sodium/potassium-transporting ATPase subunit beta-2	ACQFNR	HexNAc(2)Hex(6)	3.358	4.20E-02
P14415	Sodium/potassium-transporting ATPase subunit beta-2	FHVNYTQPLVAVK	HexNAc(2)Hex(6)	2.849	2.31E-02
P14415	Sodium/potassium-transporting ATPase subunit beta-2	TQLGNCSGIGDSTHYGYSTGQPCVFIK	HexNAc(2)Hex(5)	64.898	2.52E-07
P14415	Sodium/potassium-transporting ATPase subunit beta-2	FLEPYNDSIQAQK	HexNAc(2)Hex(5)	33.212	4.30E-05
P14415	Sodium/potassium-transporting ATPase subunit beta-2	ACQFNR	HexNAc(2)Hex(5)	27.863	3.36E-04
P14415	Sodium/potassium-transporting ATPase subunit beta-2	RACQFNR	HexNAc(2)Hex(5)	6.1	3.90E-02
P14625	Endoplasmin	TDDEVVQREEEAIQLDGLNASQIR	HexNAc(3)Hex(3)Fuc(1)	100	1.00E-17
P14625	Endoplasmin	TDDEVVQREEEAIQLDGLNASQIR	HexNAc(2)Hex(7)	2.489	3.90E-02
P14625	Endoplasmin	TDDEVVQREEEAIQLDGLNASQIR	HexNAc(2)Hex(6)	4.987	5.78E-03
P14625	Endoplasmin	EGSRTDDEVVQREEEAIQLDGLNASQIR	HexNAc(2)Hex(6)	3.034	4.18E-02
P14625	Endoplasmin	EGSRTDDEVVQREEEAIQLDGLNASQIR	HexNAc(2)Hex(5)	100	1.00E-17
P14625	Endoplasmin	LGVIEDHSNR	HexNAc(2)Hex(5)	100	1.00E-17
P14625	Endoplasmin	TDDEVVQREEEAIQLDGLNASQIR	HexNAc(2)Hex(5)	31.303	3.67E-05
P14625	Endoplasmin	EEEAIQLDGLNASQIR	HexNAc(2)Hex(5)	4.914	7.90E-03
P14867	Gamma-aminobutyric acid receptor subunit alpha-1	SVAHNMTMPNK	HexNAc(2)Hex(6)	100	1.00E-17
P15586	N-acetylglucosamine-6-sulfatase	GPGIKPNQTSK	HexNAc(5)Hex(3)	100	1.00E-17
P15586	N-acetylglucosamine-6-sulfatase	TPMTNSSIQFLDNAFR	HexNAc(2)Hex(5)	100	1.00E-17
P15586	N-acetylglucosamine-6-sulfatase	GPGIKPNQTSK	HexNAc(2)Hex(5)	100	1.00E-17
P15924	Desmoplakin	QVQNLVNK	HexNAc(5)Hex(5)	100	1.00E-17
P15924	Desmoplakin	QVQNLVNK	HexNAc(3)Hex(6)	100	1.00E-17
P15924	Desmoplakin	QVQNLVNK	HexNAc(3)Hex(4)	100	1.00E-17
P16870	Carboxypeptidase E	GNETIVNLIHSTR	HexNAc(2)Hex(9)	9.182	4.61E-03
P16870	Carboxypeptidase E	GNETIVNLIHSTR	HexNAc(2)Hex(8)	5.139	1.70E-02
P16870	Carboxypeptidase E	GNETIVNLIHSTR	HexNAc(2)Hex(7)	100	1.00E-17
P16870	Carboxypeptidase E	GNETIVNLIHSTR	HexNAc(2)Hex(6)	31.925	3.64E-05
P16870	Carboxypeptidase E	DLQGNPIANATISVEGIDHDVTSAK	HexNAc(2)Hex(6)	30.257	4.11E-06
P16870	Carboxypeptidase E	DLQGNPIANATISVEGIDHDVTSAK	HexNAc(2)Hex(5)	40.864	4.07E-06
P16870	Carboxypeptidase E	GNETIVNLIHSTR	HexNAc(2)Hex(5)	35.016	2.41E-05
P17050	Alpha-N-acetylgalactosaminidase	MAAALNATGR	HexNAc(2)Hex(5)	100	1.00E-17
P18077	60S ribosomal protein L35a	NNTVTPGGK	HexNAc(4)Hex(3)Fuc(1)	100	1.00E-17
P18084	Integrin beta-5	SNLTVLR	HexNAc(3)Hex(6)NeuAc(1)	100	1.00E-17
P18084	Integrin beta-5	SNLTVLR	HexNAc(3)Hex(6)Fuc(1)	100	1.00E-17
P18084	Integrin beta-5	SNLTVLR	HexNAc(2)Hex(4)	100	1.00E-17
P18507	Isoform 3 of Gamma-aminobutyric acid receptor subunit gamma-2	FNSTIK	HexNAc(6)Hex(4)Fuc(2)	100	1.00E-17
P18507	Isoform 3 of Gamma-aminobutyric acid receptor subunit gamma-2	NTTEVVK	HexNAc(2)Hex(6)	100	1.00E-17
P19022	Cadherin-2	SNISILR	HexNAc(5)Hex(4)Fuc(1)	100	1.00E-17
P19022	Cadherin-2	NLSLR	HexNAc(4)Hex(4)Fuc(1)	100	1.00E-17
P19022	Cadherin-2	SNISILR	HexNAc(3)Hex(6)Fuc(1)	3.614	4.61E-02
P19022	Cadherin-2	NWTITR	HexNAc(2)Hex(9)	7.075	9.15E-04
P19022	Cadherin-2	RNWTITR	HexNAc(2)Hex(8)	6.203	1.88E-02
P19022	Cadherin-2	NWTITR	HexNAc(2)Hex(8)	4.473	4.92E-03
P19022	Cadherin-2	SNISILR	HexNAc(2)Hex(7)	100	1.00E-17
P19022	Cadherin-2	NWTITR	HexNAc(2)Hex(6)	100	1.00E-17
P19022	Cadherin-2	SNISILR	HexNAc(2)Hex(6)	3.75	5.34E-03
P19022	Cadherin-2	SNISILR	HexNAc(2)Hex(5)	3.302	7.36E-03
P19022	Cadherin-2	SNISILR	HexNAc(2)Hex(4)	100	1.00E-17
P20645	Cation-dependent mannose-6-phosphate receptor	EAGNHTSGAGLVQINK	HexNAc(8)Hex(8)	100	1.00E-17
P20645	Cation-dependent mannose-6-phosphate receptor	EAGNHTSGAGLVQINK	HexNAc(5)Hex(5)Fuc(3)	27.331	4.46E-04
P20645	Cation-dependent mannose-6-phosphate receptor	EAGNHTSGAGLVQINK	HexNAc(3)Hex(6)Fuc(1)NeuAc(1)	100	1.00E-17
P20645	Cation-dependent mannose-6-phosphate receptor	EAGNHTSGAGLVQINK	HexNAc(3)Hex(4)Fuc(2)	100	1.00E-17
P20645	Cation-dependent mannose-6-phosphate receptor	EAGNHTSGAGLVQINK	HexNAc(2)Hex(5)	3.489	4.91E-02
P22681	E3 ubiquitin-protein ligase CBL	NLTK	HexNAc(4)Hex(5)Fuc(4)	100	1.00E-17
P23246	Splicing factor, proline- and glutamine-rich	ANLSLLR	HexNAc(2)Hex(8)	100	1.00E-17
P23416	Glycine receptor subunit alpha-2	QQWNDSR	HexNAc(2)Hex(8)	100	1.00E-17
P23416	Glycine receptor subunit alpha-2	QQWNDSR	HexNAc(2)Hex(7)	100	1.00E-17
P23416	Glycine receptor subunit alpha-2	QQWNDSR	HexNAc(2)Hex(6)	9.879	7.29E-03
P23416	Glycine receptor subunit alpha-2	QQWNDSR	HexNAc(2)Hex(5)	20.719	1.30E-03
P23435	Cerebellin-2	STNHEPSEMSNR	HexNAc(5)Hex(3)Fuc(1)	100	1.00E-17
P23435	Cerebellin-2	STNHEPSEMSNR	HexNAc(2)Hex(5)	6.163	1.55E-02
P23471	Receptor-type tyrosine-protein phosphatase zeta	NFTLR	HexNAc(7)Hex(4)	100	1.00E-17
P23921	Ribonucleoside-diphosphate reductase large subunit	VYNNTAR	HexNAc(2)Hex(9)	3.773	4.02E-02
P25054	Adenomatous polyposis coli protein	LPINR	HexNAc(3)Hex(4)Fuc(1)NeuAc(1)	100	1.00E-17
P26006	Isoform 2 of Integrin alpha-3	NITIVTGAPR	HexNAc(5)Hex(5)	100	1.00E-17
P26006	Isoform 2 of Integrin alpha-3	ELAVPDGYTNR	HexNAc(4)Hex(5)Fuc(1)NeuAc(1)	100	1.00E-17
P26640	Valine--tRNA ligase	LWNATK	HexNAc(5)Hex(4)Fuc(2)	100	1.00E-17
P28472	Gamma-aminobutyric acid receptor subunit beta-3	LAYSGIPLNLTLDNR	HexNAc(2)Hex(6)	100	1.00E-17
P29323	Ephrin type-B receptor 2	AGFEAVENGTVCR	HexNAc(6)Hex(4)Fuc(2)	100	1.00E-17
P29590	Protein PML	NQSVR	HexNAc(6)Hex(3)Fuc(1)	8.666	9.35E-03

P30291	Wee1-like protein kinase	KMNR	HexNAc(2)Hex(5)	25.04	4.45E-04
P30533	Alpha-2-macroglobulin receptor-associated protein	VIDLWDLAQSANLTDK	HexNAc(5)Hex(3)Fuc(1)	100	1.00E-17
P31644	Gamma-aminobutyric acid receptor subunit alpha-5	SIAHNMTTPNK	HexNAc(2)Hex(9)	100	1.00E-17
P31644	Gamma-aminobutyric acid receptor subunit alpha-5	SIAHNMTTPNK	HexNAc(2)Hex(8)	100	1.00E-17
P31644	Gamma-aminobutyric acid receptor subunit alpha-5	SIAHNMTTPNK	HexNAc(2)Hex(7)	100	1.00E-17
P31644	Gamma-aminobutyric acid receptor subunit alpha-5	SIAHNMTTPNK	HexNAc(2)Hex(6)	100	1.00E-17
P32004	Neural cell adhesion molecule L1	VPGNQTSTTLK	HexNAc(5)Hex(4)Fuc(2)	100	1.00E-17
P32004	Neural cell adhesion molecule L1	VTYQNHNK	HexNAc(5)Hex(4)Fuc(2)	24.381	3.97E-04
P32004	Neural cell adhesion molecule L1	VTYQNHNK	HexNAc(5)Hex(4)	6.767	1.35E-02
P32004	Neural cell adhesion molecule L1	VPGNQTSTTLK	HexNAc(5)Hex(3)Fuc(1)	100	1.00E-17
P32004	Neural cell adhesion molecule L1	VTYQNHNK	HexNAc(5)Hex(3)Fuc(1)	100	1.00E-17
P32004	Neural cell adhesion molecule L1	VTYQNHNK	HexNAc(5)Hex(3)	100	1.00E-17
P32004	Neural cell adhesion molecule L1	VTYQNHNK	HexNAc(4)Hex(6)	100	1.00E-17
P32004	Neural cell adhesion molecule L1	VTYQNHNK	HexNAc(4)Hex(5)Fuc(2)	100	1.00E-17
P32004	Neural cell adhesion molecule L1	VPGNQTSTTLK	HexNAc(4)Hex(5)Fuc(1)	100	1.00E-17
P32004	Neural cell adhesion molecule L1	VTYQNHNK	HexNAc(4)Hex(5)Fuc(1)	26.626	9.22E-04
P32004	Neural cell adhesion molecule L1	THNLTDLSPHLR	HexNAc(4)Hex(5)Fuc(1)	4.2	2.42E-02
P32004	Neural cell adhesion molecule L1	VPGNQTSTTLK	HexNAc(4)Hex(5)	100	1.00E-17
P32004	Neural cell adhesion molecule L1	GEGNETTNMVITWKPLR	HexNAc(4)Hex(5)	100	1.00E-17
P32004	Neural cell adhesion molecule L1	VTYQNHNK	HexNAc(4)Hex(5)	32.447	7.01E-04
P32004	Neural cell adhesion molecule L1	VPGNQTSTTLK	HexNAc(4)Hex(4)Fuc(1)	100	1.00E-17
P32004	Neural cell adhesion molecule L1	THNLTDLSPHLR	HexNAc(4)Hex(4)Fuc(1)	100	1.00E-17
P32004	Neural cell adhesion molecule L1	VTYQNHNK	HexNAc(4)Hex(4)Fuc(1)	5.983	2.18E-02
P32004	Neural cell adhesion molecule L1	VTYQNHNK	HexNAc(4)Hex(4)	100	1.00E-17
P32004	Neural cell adhesion molecule L1	VTYQNHNK	HexNAc(3)Hex(6)NeuAc(1)	100	1.00E-17
P32004	Neural cell adhesion molecule L1	VTYQNHNK	HexNAc(3)Hex(6)Fuc(1)	100	1.00E-17
P32004	Neural cell adhesion molecule L1	VTYQNHNK	HexNAc(3)Hex(5)NeuAc(1)	100	1.00E-17
P32004	Neural cell adhesion molecule L1	VTYQNHNK	HexNAc(3)Hex(5)Fuc(1)	7.512	1.59E-02
P32004	Neural cell adhesion molecule L1	VPGNQTSTTLK	HexNAc(3)Hex(5)Fuc(1)	3.746	3.90E-02
P32004	Neural cell adhesion molecule L1	VTYQNHNK	HexNAc(3)Hex(5)	100	1.00E-17
P32004	Neural cell adhesion molecule L1	VTYQNHNK	HexNAc(2)Hex(9)	10.23	5.06E-03
P32004	Neural cell adhesion molecule L1	GYNVTYWR	HexNAc(2)Hex(9)	3.957	2.95E-02
P32004	Neural cell adhesion molecule L1	GYNVTYWR	HexNAc(2)Hex(8)	17.352	3.95E-04
P32004	Neural cell adhesion molecule L1	GYNVTYWR	HexNAc(2)Hex(7)	18.133	9.90E-05
P32004	Neural cell adhesion molecule L1	THNLTDLSPHLR	HexNAc(2)Hex(7)	9.628	1.29E-02
P32004	Neural cell adhesion molecule L1	VTYQNHNK	HexNAc(2)Hex(6)	49.637	1.81E-04
P32004	Neural cell adhesion molecule L1	GYNVTYWR	HexNAc(2)Hex(6)	9.734	1.13E-03
P32004	Neural cell adhesion molecule L1	VPGNQTSTTLK	HexNAc(2)Hex(5)	100	1.00E-17
P32004	Neural cell adhesion molecule L1	VTYQNHNK	HexNAc(2)Hex(5)	100	1.00E-17
P32004	Neural cell adhesion molecule L1	GEGNETTNMVITWKPLR	HexNAc(2)Hex(5)	100	1.00E-17
P32004	Neural cell adhesion molecule L1	THNLTDLSPHLR	HexNAc(2)Hex(5)	28.084	1.83E-05
P32004	Neural cell adhesion molecule L1	FFPYANGTLGIR	HexNAc(2)Hex(5)	10.143	1.15E-02
P32004	Neural cell adhesion molecule L1	VPGNQTSTTLK	HexNAc(2)Hex(4)	100	1.00E-17
P32004	Neural cell adhesion molecule L1	VTYQNHNK	HexNAc(2)Hex(4)	37.106	4.46E-04
P32004	Neural cell adhesion molecule L1	VPGNQTSTTLK	HexNAc(2)Hex(3)	100	1.00E-17
P32004	Neural cell adhesion molecule L1	VTYQNHNK	HexNAc(2)Hex(3)	100	1.00E-17
P32004	Neural cell adhesion molecule L1	THNLTDLSPHLR	HexNAc(2)Hex(3)	100	1.00E-17
P33981	Dual specificity protein kinase TTK	QTNK	HexNAc(8)Hex(3)	100	1.00E-17
P33981	Dual specificity protein kinase TTK	NSLRQTNK	HexNAc(4)Hex(5)Fuc(2)	100	1.00E-17
P33981	Dual specificity protein kinase TTK	NSLRQTNK	HexNAc(4)Hex(5)Fuc(1)NeuAc(1)	100	1.00E-17
P34903	Gamma-aminobutyric acid receptor subunit alpha-3	HAPDIPDDSTDNITIFTR	HexNAc(5)Hex(4)Fuc(2)	5.057	2.13E-02
P34903	Gamma-aminobutyric acid receptor subunit alpha-3	SVAHNMTTPNK	HexNAc(2)Hex(9)	7.231	7.85E-03
P34903	Gamma-aminobutyric acid receptor subunit alpha-3	SVAHNMTTPNK	HexNAc(2)Hex(8)	11.467	3.47E-03
P34903	Gamma-aminobutyric acid receptor subunit alpha-3	SVAHNMTTPNK	HexNAc(2)Hex(7)	13.294	3.37E-03
P34903	Gamma-aminobutyric acid receptor subunit alpha-3	SVAHNMTTPNK	HexNAc(2)Hex(6)	100	1.00E-17
P34903	Gamma-aminobutyric acid receptor subunit alpha-3	SVAHNMTTPNK	HexNAc(2)Hex(5)	100	1.00E-17
P35052	Glypican-1	ICPQGYTCCTSEMEENLANR	HexNAc(5)Hex(4)NeuAc(1)	100	1.00E-17
P35221	Isoform 2 of Catenin alpha-1	NTSDVISAAK	HexNAc(4)Hex(3)NeuAc(1)	5.441	4.84E-03
P35269	General transcription factor IIF subunit 1	NTTKK	HexNAc(4)Hex(7)	100	1.00E-17
P35556	Fibrillin-2	AFNTTK	HexNAc(2)Hex(6)	100	1.00E-17
P35580	Isoform 4 of Myosin-10	LNLSSR	HexNAc(4)Hex(3)NeuAc(1)	100	1.00E-17
P35613	Basigin	ILLTCSLNDSATEVTGHR	HexNAc(5)Hex(5)Fuc(3)	100	1.00E-17
P35968	Vascular endothelial growth factor receptor 2	NSTFVR	HexNAc(2)Hex(6)	3.673	4.61E-02
P41217	Isoform 3 of OX-2 membrane glycoprotein	FSEDHLNITCSATAR	HexNAc(6)Hex(5)Fuc(1)NeuAc(1)	100	1.00E-17
P41217	Isoform 3 of OX-2 membrane glycoprotein	FSEDHLNITCSATAR	HexNAc(6)Hex(4)NeuAc(1)	100	1.00E-17
P41217	Isoform 3 of OX-2 membrane glycoprotein	FSEDHLNITCSATAR	HexNAc(5)Hex(6)NeuAc(1)	100	1.00E-17
P41217	Isoform 3 of OX-2 membrane glycoprotein	FSEDHLNITCSATAR	HexNAc(5)Hex(4)Fuc(1)	10.826	6.41E-04
P41217	Isoform 3 of OX-2 membrane glycoprotein	FSEDHLNITCSATAR	HexNAc(5)Hex(3)	100	1.00E-17
P41217	Isoform 3 of OX-2 membrane glycoprotein	FSEDHLNITCSATAR	HexNAc(4)Hex(6)Fuc(2)	5.902	9.66E-03
P41217	Isoform 3 of OX-2 membrane glycoprotein	FSEDHLNITCSATAR	HexNAc(4)Hex(6)Fuc(1)NeuAc(1)	100	1.00E-17

P41217	Isoform 3 of OX-2 membrane glycoprotein	FSEDHLNITCSATAR	HexNAc(4)Hex(6)Fuc(1)	100	1.00E-17
P41217	Isoform 3 of OX-2 membrane glycoprotein	FSEDHLNITCSATAR	HexNAc(4)Hex(6)	100	1.00E-17
P41217	Isoform 3 of OX-2 membrane glycoprotein	FSEDHLNITCSATAR	HexNAc(4)Hex(5)Fuc(2)	3.048	3.89E-02
P41217	Isoform 3 of OX-2 membrane glycoprotein	FSEDHLNITCSATAR	HexNAc(4)Hex(5)Fuc(1)	100	1.00E-17
P41217	Isoform 3 of OX-2 membrane glycoprotein	FSEDHLNITCSATAR	HexNAc(4)Hex(5)	100	1.00E-17
P41217	Isoform 3 of OX-2 membrane glycoprotein	FSEDHLNITCSATAR	HexNAc(4)Hex(4)Fuc(1)	10.086	3.37E-03
P41217	Isoform 3 of OX-2 membrane glycoprotein	FSEDHLNITCSATAR	HexNAc(4)Hex(4)	100	1.00E-17
P41217	Isoform 3 of OX-2 membrane glycoprotein	FSEDHLNITCSATAR	HexNAc(3)Hex(6)NeuAc(1)	28.49	1.73E-05
P41217	Isoform 3 of OX-2 membrane glycoprotein	FSEDHLNITCSATAR	HexNAc(3)Hex(6)Fuc(1)	17.998	1.15E-04
P41217	Isoform 3 of OX-2 membrane glycoprotein	FSEDHLNITCSATAR	HexNAc(3)Hex(6)	100	1.00E-17
P41217	Isoform 3 of OX-2 membrane glycoprotein	FSEDHLNITCSATAR	HexNAc(3)Hex(5)NeuAc(1)	8.384	9.66E-04
P41217	Isoform 3 of OX-2 membrane glycoprotein	FSEDHLNITCSATAR	HexNAc(3)Hex(5)Fuc(1)	29.668	4.19E-05
P41217	Isoform 3 of OX-2 membrane glycoprotein	FSEDHLNITCSATAR	HexNAc(3)Hex(5)	100	1.00E-17
P41217	Isoform 3 of OX-2 membrane glycoprotein	FSEDHLNITCSATAR	HexNAc(3)Hex(4)	100	1.00E-17
P41217	Isoform 3 of OX-2 membrane glycoprotein	FSEDHLNITCSATAR	HexNAc(2)Hex(8)	100	1.00E-17
P41217	Isoform 3 of OX-2 membrane glycoprotein	FSEDHLNITCSATAR	HexNAc(2)Hex(7)	32.125	2.08E-05
P41217	Isoform 3 of OX-2 membrane glycoprotein	FSEDHLNITCSATAR	HexNAc(2)Hex(6)	14.038	1.03E-04
P41217	Isoform 3 of OX-2 membrane glycoprotein	FSEDHLNITCSATAR	HexNAc(2)Hex(5)	100	1.00E-17
P42658	Dipeptidyl aminopeptidase-like protein 6	CEGPGVPMVTVHNTTDK	HexNAc(5)Hex(3)	100	1.00E-17
P42658	Dipeptidyl aminopeptidase-like protein 6	LAYAAINDSR	HexNAc(5)Hex(3)	100	1.00E-17
P42658	Dipeptidyl aminopeptidase-like protein 6	LAYAAINDSR	HexNAc(4)Hex(4)	100	1.00E-17
P42658	Dipeptidyl aminopeptidase-like protein 6	LAYAAINDSR	HexNAc(4)Hex(3)	100	1.00E-17
P42658	Dipeptidyl aminopeptidase-like protein 6	ANYSLQIYPDESHYFTSSSLK	HexNAc(2)Hex(9)	13.514	4.52E-03
P42658	Dipeptidyl aminopeptidase-like protein 6	LWNVETNTSTVLIEGK	HexNAc(2)Hex(8)	4.457	2.53E-02
P42658	Dipeptidyl aminopeptidase-like protein 6	AQNVSILTLCDATTGVCTK	HexNAc(2)Hex(6)	4.972	3.39E-03
P42658	Dipeptidyl aminopeptidase-like protein 6	AQNVSILTLCDATTGVCTK	HexNAc(2)Hex(5)	5.092	3.61E-02
P42785	Isoform 2 of Lysosomal Pro-X carboxypeptidase	NYSVLYFQQK	HexNAc(2)Hex(5)	2.581	1.33E-02
P42785	Isoform 2 of Lysosomal Pro-X carboxypeptidase	NYSVLYFQQK	HexNAc(2)Hex(4)	3.496	4.03E-02
P43121	Cell surface glycoprotein MUC18	CVASVPSIPGLNR	HexNAc(5)Hex(4)	100	1.00E-17
P43146	Netrin receptor DCC	ALNTTQPGSLQLTVGNLKPEAMYTFR	HexNAc(2)Hex(6)	100	1.00E-17
P43146	Netrin receptor DCC	ALNTTQPGSLQLTVGNLKPEAMYTFR	HexNAc(2)Hex(5)	3.916	3.58E-02
P46100	Transcriptional regulator ATRX	RNLSSK	HexNAc(5)Hex(5)Fuc(1)NeuAc(1)	100	1.00E-17
P46100	Transcriptional regulator ATRX	RNLSSK	HexNAc(5)Hex(5)Fuc(1)	4.412	3.65E-02
P46459	Vesicle-fusing ATPase	AENSSLNLIGK	HexNAc(4)Hex(4)Fuc(1)	100	1.00E-17
P46459	Vesicle-fusing ATPase	AENSSLNLIGK	HexNAc(2)Hex(7)	100	1.00E-17
P48723	Heat shock 70 kDa protein 13	QRNSTIEAANLAGLK	HexNAc(2)Hex(7)	100	1.00E-17
P48723	Heat shock 70 kDa protein 13	NSTIEAANLAGLK	HexNAc(2)Hex(7)	17.914	1.05E-03
P48723	Heat shock 70 kDa protein 13	QRNSTIEAANLAGLK	HexNAc(2)Hex(6)	100	1.00E-17
P48723	Heat shock 70 kDa protein 13	NSTIEAANLAGLK	HexNAc(2)Hex(6)	100	1.00E-17
P48723	Heat shock 70 kDa protein 13	QRNSTIEAANLAGLK	HexNAc(2)Hex(5)	100	1.00E-17
P48723	Heat shock 70 kDa protein 13	NSTIEAANLAGLK	HexNAc(2)Hex(5)	100	1.00E-17
P49454	Centromere protein F	NLTVELEQK	HexNAc(4)Hex(4)NeuAc(1)	8.602	6.48E-03
P49588	Isoform 2 of Alanine--tRNA ligase, cytoplasmic	LNASR	HexNAc(4)Hex(3)Fuc(2)	7.893	9.48E-03
P49756	RNA-binding protein 25	NATK	HexNAc(6)Hex(6)	100	1.00E-17
P49790	Isoform 3 of Nuclear pore complex protein Nup153	PVSIATNR	HexNAc(3)Hex(4)Fuc(1)NeuAc(1)	100	1.00E-17
P50454	Serpin H1	NVTWK	HexNAc(3)Hex(3)Fuc(1)	100	1.00E-17
P50851	Lipopolysaccharide-responsive and beige-like anchor protein	ATNLTR	HexNAc(3)Hex(4)	100	1.00E-17
P50895	Basal cell adhesion molecule	TQNFTLLVQGSPELK	HexNAc(2)Hex(9)	100	1.00E-17
P51654	Isoform 3 of Glypican-3	NYTNAMFK	HexNAc(5)Hex(3)Fuc(1)	100	1.00E-17
P51689	Arylsulfatase D	ALQWNAGSGGLPENETTFAR	HexNAc(2)Hex(9)	100	1.00E-17
P51805	Plexin-A3	ANISSPGLIYTYTQDPTVTR	HexNAc(5)Hex(4)NeuAc(1)	3.649	4.43E-02
P51805	Plexin-A3	SLNR*	HexNAc(5)Hex(3)Fuc(1)	100	1.00E-17
P51805	Plexin-A3	LAPNLTELR*	HexNAc(5)Hex(3)Fuc(1)	100	1.00E-17
P51805	Plexin-A3	LAPNLTELR	HexNAc(4)Hex(5)Fuc(1)	3.814	3.70E-02
P51805	Plexin-A3	SLNR	HexNAc(3)Hex(5)	100	1.00E-17
P51805	Plexin-A3	GIETTNTCQVINDTAMLCK	HexNAc(2)Hex(6)	3.296	4.10E-02
P51805	Plexin-A3	LAPNLTELR	HexNAc(2)Hex(5)	100	1.00E-17
P51805	Plexin-A3	GIETTNTCQVINDTAMLCK	HexNAc(2)Hex(5)	13.101	2.93E-04
P52292	Importin subunit alpha-1	EATWTMSNITAGR	HexNAc(3)Hex(4)Fuc(1)	100	1.00E-17
P52797	Ephrin-A3	TCNASQGFK	HexNAc(6)Hex(3)Fuc(2)	100	1.00E-17
P52797	Ephrin-A3	TCNASQGFK	HexNAc(5)Hex(5)Fuc(3)	4.716	5.00E-02
P52797	Ephrin-A3	TCNASQGFK	HexNAc(5)Hex(4)Fuc(2)	100	1.00E-17
P52797	Ephrin-A3	TCNASQGFK	HexNAc(2)Hex(5)	100	1.00E-17
P52799	Ephrin-B2	DYYIISTSNGSLEGLDNQEGGVCQTR	HexNAc(5)Hex(4)Fuc(2)	100	1.00E-17
P52799	Ephrin-B2	SIVLEPIYWNSSNSK	HexNAc(2)Hex(8)	6.69	1.23E-02
P52799	Ephrin-B2	SIVLEPIYWNSSNSK	HexNAc(2)Hex(6)	6.212	3.27E-03
P52799	Ephrin-B2	SIVLEPIYWNSSNSK	HexNAc(2)Hex(5)	5.1	3.38E-02
P53634	Dipeptidyl peptidase 1	DVNCSVMGPQEK	HexNAc(3)Hex(4)Fuc(1)NeuAc(1)	100	1.00E-17
P54289	Voltage-dependent calcium channel subunit alpha-2/delta-1	YIDKGNR	HexNAc(5)Hex(3)	3.53	4.03E-02
P54289	Voltage-dependent calcium channel subunit alpha-2/delta-1	YIDKGNR	HexNAc(4)Hex(5)	5.177	2.24E-02
P54289	Voltage-dependent calcium channel subunit alpha-2/delta-1	YIDKGNR	HexNAc(2)Hex(5)	25.674	8.53E-04
P54709	Sodium/potassium-transporting ATPase subunit beta-3	NLTVCPDGALFEQK	HexNAc(5)Hex(6)NeuAc(3)	3.271	3.79E-02

P54709	Sodium/potassium-transporting ATPase subunit beta-3	NLTVCPDGALFEQK	HexNAc(5)Hex(6)Fuc(1)NeuAc(2)	100	1.00E-17
P54709	Sodium/potassium-transporting ATPase subunit beta-3	NLTVCPDGALFEQK	HexNAc(5)Hex(4)Fuc(2)	13.733	4.78E-04
P54709	Sodium/potassium-transporting ATPase subunit beta-3	NLTVCPDGALFEQK	HexNAc(5)Hex(4)Fuc(1)NeuAc(1)	2.808	1.09E-02
P54709	Sodium/potassium-transporting ATPase subunit beta-3	NLTVCPDGALFEQK	HexNAc(5)Hex(3)Fuc(1)	7.557	2.20E-03
P54709	Sodium/potassium-transporting ATPase subunit beta-3	NLTVCPDGALFEQK	HexNAc(4)Hex(4)Fuc(1)	100	1.00E-17
P54709	Sodium/potassium-transporting ATPase subunit beta-3	NLTVCPDGALFEQK	HexNAc(3)Hex(6)Fuc(1)NeuAc(1)	100	1.00E-17
P54709	Sodium/potassium-transporting ATPase subunit beta-3	NLTVCPDGALFEQK	HexNAc(3)Hex(6)Fuc(1)	4.093	2.17E-02
P54709	Sodium/potassium-transporting ATPase subunit beta-3	NLTVCPDGALFEQK	HexNAc(3)Hex(4)Fuc(1)	100	1.00E-17
P54709	Sodium/potassium-transporting ATPase subunit beta-3	NLTVCPDGALFEQK	HexNAc(2)Hex(5)	13.63	2.46E-04
P54709	Sodium/potassium-transporting ATPase subunit beta-3	NLTVCPDGALFEQK	HexNAc(2)Hex(4)	100	1.00E-17
P54753	Ephrin type-B receptor 3	YAAVNITTNQAAPSEVPTLR	HexNAc(2)Hex(5)	8.89	6.18E-04
P54802	Alpha-N-acetylglucosaminidase	SVYNCSGEACR	HexNAc(2)Hex(6)	100	1.00E-17
P54802	Alpha-N-acetylglucosaminidase	SVYNCSGEACR	HexNAc(2)Hex(5)	100	1.00E-17
P54802	Alpha-N-acetylglucosaminidase	SVYNCSGEACR	HexNAc(2)Hex(4)	100	1.00E-17
P56199	Integrin alpha-1	VYVYALNQTR	HexNAc(4)Hex(5)Fuc(1)	100	1.00E-17
P56373	P2X purinoceptor 3	GNLLPNLTAR	HexNAc(2)Hex(8)	100	1.00E-17
P60842	Isoform 2 of Eukaryotic initiation factor 4A-I	LYPQNR	HexNAc(2)Hex(8)	100	1.00E-17
P61812	Isoform B of Transforming growth factor beta-2	NASNLVK	HexNAc(4)Hex(7)Fuc(1)	100	1.00E-17
P61812	Isoform B of Transforming growth factor beta-2	NASNLVK	HexNAc(2)Hex(9)	100	1.00E-17
P61812	Isoform B of Transforming growth factor beta-2	NASNLVK	HexNAc(2)Hex(6)	100	1.00E-17
P62910	60S ribosomal protein L32	ELEVLLMCNK	HexNAc(2)Hex(5)	100	1.00E-17
P67809	Nuclease-sensitive element-binding protein 1	NDTK	HexNAc(9)Hex(3)	100	1.00E-17
P67809	Nuclease-sensitive element-binding protein 1	NDTK	HexNAc(6)Hex(3)Fuc(3)	100	1.00E-17
P78310	Coxsackievirus and adenovirus receptor	SGDASINVTNLQLSDIGTYQCK	HexNAc(5)Hex(5)	6.1	2.87E-03
P78310	Coxsackievirus and adenovirus receptor	SGDASINVTNLQLSDIGTYQCK	HexNAc(5)Hex(4)NeuAc(1)	5.665	8.92E-03
P78310	Coxsackievirus and adenovirus receptor	SGDASINVTNLQLSDIGTYQCK	HexNAc(5)Hex(4)Fuc(2)	4.907	1.43E-02
P78310	Coxsackievirus and adenovirus receptor	SGDASINVTNLQLSDIGTYQCK	HexNAc(5)Hex(4)Fuc(1)	12.452	2.71E-04
P78310	Coxsackievirus and adenovirus receptor	NASSEYSGTYSCTVR	HexNAc(5)Hex(3)Fuc(1)	100	1.00E-17
P78310	Coxsackievirus and adenovirus receptor	SGDASINVTNLQLSDIGTYQCK	HexNAc(5)Hex(3)Fuc(1)	100	1.00E-17
P78310	Coxsackievirus and adenovirus receptor	SGDASINVTNLQLSDIGTYQCK	HexNAc(5)Hex(3)	100	1.00E-17
P78310	Coxsackievirus and adenovirus receptor	NASSEYSGTYSCTVR	HexNAc(4)Hex(7)Fuc(2)	100	1.00E-17
P78310	Coxsackievirus and adenovirus receptor	NASSEYSGTYSCTVR	HexNAc(4)Hex(6)Fuc(2)	100	1.00E-17
P78310	Coxsackievirus and adenovirus receptor	NASSEYSGTYSCTVR	HexNAc(4)Hex(6)Fuc(1)NeuAc(1)	100	1.00E-17
P78310	Coxsackievirus and adenovirus receptor	NASSEYSGTYSCTVR	HexNAc(4)Hex(6)Fuc(1)	6.893	9.87E-03
P78310	Coxsackievirus and adenovirus receptor	NASSEYSGTYSCTVR	HexNAc(4)Hex(6)	100	1.00E-17
P78310	Coxsackievirus and adenovirus receptor	NASSEYSGTYSCTVR	HexNAc(4)Hex(5)Fuc(1)	100	1.00E-17
P78310	Coxsackievirus and adenovirus receptor	SGDASINVTNLQLSDIGTYQCK	HexNAc(4)Hex(5)Fuc(1)	23.26	5.50E-05
P78310	Coxsackievirus and adenovirus receptor	SGDASINVTNLQLSDIGTYQCK	HexNAc(4)Hex(5)	37.699	1.85E-06
P78310	Coxsackievirus and adenovirus receptor	NASSEYSGTYSCTVR	HexNAc(4)Hex(4)Fuc(1)	100	1.00E-17
P78310	Coxsackievirus and adenovirus receptor	SGDASINVTNLQLSDIGTYQCK	HexNAc(4)Hex(3)	20.991	1.08E-03
P78310	Coxsackievirus and adenovirus receptor	NASSEYSGTYSCTVR	HexNAc(3)Hex(6)Fuc(1)NeuAc(1)	21.821	1.95E-04
P78310	Coxsackievirus and adenovirus receptor	NASSEYSGTYSCTVR	HexNAc(3)Hex(6)Fuc(1)	100	1.00E-17
P78310	Coxsackievirus and adenovirus receptor	SGDASINVTNLQLSDIGTYQCK	HexNAc(3)Hex(6)Fuc(1)	100	1.00E-17
P78310	Coxsackievirus and adenovirus receptor	NASSEYSGTYSCTVR	HexNAc(2)Hex(7)	4.384	4.33E-03
P78310	Coxsackievirus and adenovirus receptor	NASSEYSGTYSCTVR	HexNAc(2)Hex(6)	4.245	2.40E-02
P78310	Coxsackievirus and adenovirus receptor	NASSEYSGTYSCTVR	HexNAc(2)Hex(5)	56.092	4.78E-07
P78310	Coxsackievirus and adenovirus receptor	NASSEYSGTYSCTVR	HexNAc(2)Hex(4)	100	1.00E-17
P78324	Isoform 2 of Tyrosine-protein phosphatase non-receptor type substrate 1	GTANLSETIR*	HexNAc(5)Hex(3)Fuc(1)	100	1.00E-17
P78324	Isoform 2 of Tyrosine-protein phosphatase non-receptor type substrate 1	AENQVNVTCQVR	HexNAc(2)Hex(7)	3.729	2.16E-02
P78344	Eukaryotic translation initiation factor 4 gamma 2	DNISPK	HexNAc(7)Hex(3)	100	1.00E-17
P98160	Basement membrane-specific heparan sulfate proteoglycan core protein	ALVNFTR	HexNAc(2)Hex(7)	100	1.00E-17
P98164	Low-density lipoprotein receptor-related protein 2	INTTYR	HexNAc(4)Hex(6)Fuc(2)	100	1.00E-17
P98164	Low-density lipoprotein receptor-related protein 2	INTTYR	HexNAc(4)Hex(5)Fuc(2)	100	1.00E-17
P98164	Low-density lipoprotein receptor-related protein 2	TDGTNR	HexNAc(3)Hex(6)Fuc(1)	100	1.00E-17
P98172	Ephrin-B1	HHDYYITSTSNGSLEGLENR	HexNAc(5)Hex(5)Fuc(3)	100	1.00E-17
Q01974	Tyrosine-protein kinase transmembrane receptor ROR2	YHQCYNGSGMDYR	HexNAc(2)Hex(5)	100	1.00E-17
Q02246	Contactin-2	ANSTGILSVR*	HexNAc(5)Hex(3)Fuc(1)	100	1.00E-17
Q02246	Contactin-2	GFDNHSPIAK	HexNAc(3)Hex(4)Fuc(1)	100	1.00E-17
Q02246	Contactin-2	MNGTEMK	HexNAc(2)Hex(8)	100	1.00E-17
Q02246	Contactin-2	MNGTEMK	HexNAc(2)Hex(7)	100	1.00E-17
Q02246	Contactin-2	GTEILVNSSR	HexNAc(2)Hex(7)	9.876	4.90E-03
Q02246	Contactin-2	MNGTEMK	HexNAc(2)Hex(6)	100	1.00E-17
Q02246	Contactin-2	GTEILVNSSR	HexNAc(2)Hex(6)	100	1.00E-17
Q02246	Contactin-2	ANSTGILSVR	HexNAc(2)Hex(5)	100	1.00E-17
Q02246	Contactin-2	MNGTEMK	HexNAc(2)Hex(5)	100	1.00E-17
Q02246	Contactin-2	GTEILVNSSR	HexNAc(2)Hex(5)	100	1.00E-17
Q02246	Contactin-2	WDPVVPFRNESAVTGYK	HexNAc(2)Hex(5)	7.067	1.15E-02
Q02487	Desmocollin-2	NVTLHVPSK	HexNAc(2)Hex(9)	100	1.00E-17

Q02880	Isoform Beta-1 of DNA topoisomerase 2-beta	ITIENR	HexNAc(5)Hex(5)Fuc(1)	100	1.00E-17
Q02880	Isoform Beta-1 of DNA topoisomerase 2-beta	ITIENR	HexNAc(4)Hex(5)Fuc(1)	100	1.00E-17
Q03701	CCAAT/enhancer-binding protein zeta	DNASLKQLR	HexNAc(5)Hex(4)	2.052	3.80E-02
Q05586	Isoform 5 of Glutamate receptor ionotropic, NMDA 1	FANYSIMNLQNR	HexNAc(2)Hex(8)	100	1.00E-17
Q06210	Glutamine--fructose-6-phosphate aminotransferase [isomerizing] 1	GSCNLSR	HexNAc(4)Hex(3)Fuc(1)	100	1.00E-17
Q06210	Glutamine--fructose-6-phosphate aminotransferase [isomerizing] 1	GSCNLSR	HexNAc(3)Hex(3)Fuc(1)	100	1.00E-17
Q07954	Prolow-density lipoprotein receptor-related protein 1	FNSTEYQVVTR	HexNAc(5)Hex(5)NeuAc(1)	100	1.00E-17
Q07954	Prolow-density lipoprotein receptor-related protein 1	CNASSQFLCSSGR	HexNAc(5)Hex(5)Fuc(2)	100	1.00E-17
Q07954	Prolow-density lipoprotein receptor-related protein 1	WLCDGDNDCGNSEDESNATCSAR	HexNAc(5)Hex(4)Fuc(2)	100	1.00E-17
Q07954	Prolow-density lipoprotein receptor-related protein 1	DNATDSVPLR	HexNAc(5)Hex(4)Fuc(2)	100	1.00E-17
Q07954	Prolow-density lipoprotein receptor-related protein 1	CNASSQFLCSSGR	HexNAc(5)Hex(4)Fuc(2)	100	1.00E-17
Q07954	Prolow-density lipoprotein receptor-related protein 1	IETILLNGTDRK	HexNAc(5)Hex(4)Fuc(2)	100	1.00E-17
Q07954	Prolow-density lipoprotein receptor-related protein 1	LTSCATNASICGDEAR	HexNAc(5)Hex(4)Fuc(2)	17.695	2.18E-03
Q07954	Prolow-density lipoprotein receptor-related protein 1	ELQGNCSR	HexNAc(5)Hex(4)Fuc(2)	5.445	3.35E-02
Q07954	Prolow-density lipoprotein receptor-related protein 1	DNATDSVPLR	HexNAc(5)Hex(4)Fuc(1)	100	1.00E-17
Q07954	Prolow-density lipoprotein receptor-related protein 1	QSGDVTCNCTDGR	HexNAc(5)Hex(3)Fuc(1)	100	1.00E-17
Q07954	Prolow-density lipoprotein receptor-related protein 1	WLCDGDNDCGNSEDESNATCSAR	HexNAc(5)Hex(3)Fuc(1)	100	1.00E-17
Q07954	Prolow-density lipoprotein receptor-related protein 1	TCPLDEFQCNNTLCKPLAWK	HexNAc(5)Hex(3)Fuc(1)	100	1.00E-17
Q07954	Prolow-density lipoprotein receptor-related protein 1	DNATDSVPLR*	HexNAc(5)Hex(3)Fuc(1)	100	1.00E-17
Q07954	Prolow-density lipoprotein receptor-related protein 1	AVNSSCR*	HexNAc(5)Hex(3)Fuc(1)	100	1.00E-17
Q07954	Prolow-density lipoprotein receptor-related protein 1	CTQQVCAGYCANNSTCTVNQGNQPQCR	HexNAc(5)Hex(3)Fuc(1)	100	1.00E-17
Q07954	Prolow-density lipoprotein receptor-related protein 1	CNASSQFLCSSGR*	HexNAc(5)Hex(3)Fuc(1)	100	1.00E-17
Q07954	Prolow-density lipoprotein receptor-related protein 1	MHLNGSNVQVLHR	HexNAc(5)Hex(3)Fuc(1)	100	1.00E-17
Q07954	Prolow-density lipoprotein receptor-related protein 1	LTSCATNASICGDEAR*	HexNAc(5)Hex(3)Fuc(1)	100	1.00E-17
Q07954	Prolow-density lipoprotein receptor-related protein 1	ELQGNCSR	HexNAc(5)Hex(3)Fuc(1)	7.35	1.04E-02
Q07954	Prolow-density lipoprotein receptor-related protein 1	IETILLNGTDRK*	HexNAc(5)Hex(3)Fuc(1)	5.47	1.37E-02
Q07954	Prolow-density lipoprotein receptor-related protein 1	FNSTEYQVVTR	HexNAc(5)Hex(3)	100	1.00E-17
Q07954	Prolow-density lipoprotein receptor-related protein 1	CNASSQFLCSSGR	HexNAc(4)Hex(5)Fuc(2)	100	1.00E-17
Q07954	Prolow-density lipoprotein receptor-related protein 1	TCPLDEFQCNNTLCK	HexNAc(4)Hex(5)Fuc(1)	100	1.00E-17
Q07954	Prolow-density lipoprotein receptor-related protein 1	AVNSSCR	HexNAc(4)Hex(5)Fuc(1)	18.222	6.62E-04
Q07954	Prolow-density lipoprotein receptor-related protein 1	CNASSQFLCSSGR	HexNAc(4)Hex(5)Fuc(1)	6.988	1.84E-02
Q07954	Prolow-density lipoprotein receptor-related protein 1	LTSCATNASICGDEAR	HexNAc(4)Hex(5)Fuc(1)	4.619	3.10E-02
Q07954	Prolow-density lipoprotein receptor-related protein 1	GVTHLNISGLK	HexNAc(4)Hex(5)	100	1.00E-17
Q07954	Prolow-density lipoprotein receptor-related protein 1	LTSCATNASICGDEAR	HexNAc(4)Hex(4)Fuc(1)NeuAc(1)	100	1.00E-17
Q07954	Prolow-density lipoprotein receptor-related protein 1	DNATDSVPLR	HexNAc(4)Hex(4)Fuc(1)	100	1.00E-17
Q07954	Prolow-density lipoprotein receptor-related protein 1	CNASSQFLCSSGR	HexNAc(4)Hex(4)Fuc(1)	100	1.00E-17
Q07954	Prolow-density lipoprotein receptor-related protein 1	AVNSSCR	HexNAc(4)Hex(4)Fuc(1)	15.435	9.80E-04
Q07954	Prolow-density lipoprotein receptor-related protein 1	LTSCATNASICGDEAR	HexNAc(4)Hex(4)Fuc(1)	7.489	8.40E-03
Q07954	Prolow-density lipoprotein receptor-related protein 1	DNATDSVPLR	HexNAc(4)Hex(3)Fuc(1)	9.805	6.72E-03
Q07954	Prolow-density lipoprotein receptor-related protein 1	WTGHNVTVVQR	HexNAc(3)Hex(6)Fuc(1)	100	1.00E-17
Q07954	Prolow-density lipoprotein receptor-related protein 1	TCVSNCTASQFVCK	HexNAc(3)Hex(6)Fuc(1)	100	1.00E-17
Q07954	Prolow-density lipoprotein receptor-related protein 1	WTGHNVTVVQR	HexNAc(3)Hex(5)	100	1.00E-17
Q07954	Prolow-density lipoprotein receptor-related protein 1	WTGHNVTVVQR	HexNAc(3)Hex(4)Fuc(1)	100	1.00E-17
Q07954	Prolow-density lipoprotein receptor-related protein 1	LYWISSGNHTINR	HexNAc(2)Hex(8)	100	1.00E-17
Q07954	Prolow-density lipoprotein receptor-related protein 1	LNGTDPIVAADSKR	HexNAc(2)Hex(7)	100	1.00E-17
Q07954	Prolow-density lipoprotein receptor-related protein 1	LNGTDPIVAADSK	HexNAc(2)Hex(7)	12.55	1.09E-04
Q07954	Prolow-density lipoprotein receptor-related protein 1	WTGHNVTVVQR	HexNAc(2)Hex(7)	10.756	8.96E-04
Q07954	Prolow-density lipoprotein receptor-related protein 1	FGTCSQLCNNTK	HexNAc(2)Hex(7)	8.916	1.70E-03
Q07954	Prolow-density lipoprotein receptor-related protein 1	TCVSNCTASQFVCK	HexNAc(2)Hex(7)	7.24	1.19E-03
Q07954	Prolow-density lipoprotein receptor-related protein 1	DNTTCYEFK	HexNAc(2)Hex(7)	4.643	1.74E-02
Q07954	Prolow-density lipoprotein receptor-related protein 1	DNTTCYEFKK	HexNAc(2)Hex(7)	3.168	3.40E-02
Q07954	Prolow-density lipoprotein receptor-related protein 1	WTGHNVTVVQR	HexNAc(2)Hex(6)	100	1.00E-17
Q07954	Prolow-density lipoprotein receptor-related protein 1	FNSTEYQVVTR	HexNAc(2)Hex(6)	100	1.00E-17
Q07954	Prolow-density lipoprotein receptor-related protein 1	GVTHLNISGLK	HexNAc(2)Hex(6)	100	1.00E-17

Q07954	Prolow-density lipoprotein receptor-related protein 1	FGTCSQLCNNTK	HexNAc(2)Hex(6)	5.764	1.41E-03
Q07954	Prolow-density lipoprotein receptor-related protein 1	TCVSNCTASQFVCK	HexNAc(2)Hex(6)	5.26	4.51E-03
Q07954	Prolow-density lipoprotein receptor-related protein 1	DNTTCYEFKK	HexNAc(2)Hex(6)	2.722	4.77E-02
Q07954	Prolow-density lipoprotein receptor-related protein 1	QPMAPNPCEANGGQGPCSHLCLINYNR	HexNAc(2)Hex(5)	100	1.00E-17
Q07954	Prolow-density lipoprotein receptor-related protein 1	VNRFNSTEYQVVTR	HexNAc(2)Hex(5)	100	1.00E-17
Q07954	Prolow-density lipoprotein receptor-related protein 1	CNQFVDCEDASDEMNCSATDCSSYFR	HexNAc(2)Hex(5)	100	1.00E-17
Q07954	Prolow-density lipoprotein receptor-related protein 1	CTQQVCAGYCANNSTCTVNQGNQPQCR	HexNAc(2)Hex(5)	100	1.00E-17
Q07954	Prolow-density lipoprotein receptor-related protein 1	INNGGCQDLCLLTHQGHVNCSCR	HexNAc(2)Hex(5)	100	1.00E-17
Q07954	Prolow-density lipoprotein receptor-related protein 1	IETILLNGTDRK	HexNAc(2)Hex(5)	100	1.00E-17
Q07954	Prolow-density lipoprotein receptor-related protein 1	IETILLNGTDR	HexNAc(2)Hex(5)	100	1.00E-17
Q07954	Prolow-density lipoprotein receptor-related protein 1	LYWISSGNHTINR	HexNAc(2)Hex(5)	100	1.00E-17
Q07954	Prolow-density lipoprotein receptor-related protein 1	LYWTDGDNISMANMDGSNR	HexNAc(2)Hex(5)	100	1.00E-17
Q07954	Prolow-density lipoprotein receptor-related protein 1	LTSCATNASICGDEAR	HexNAc(2)Hex(5)	100	1.00E-17
Q07954	Prolow-density lipoprotein receptor-related protein 1	FNSTEYQVVTR	HexNAc(2)Hex(5)	54.843	3.15E-06
Q07954	Prolow-density lipoprotein receptor-related protein 1	WTGHNVTVVQR	HexNAc(2)Hex(5)	43.291	1.44E-06
Q07954	Prolow-density lipoprotein receptor-related protein 1	NSTTLVMHMK	HexNAc(2)Hex(5)	19.117	1.25E-03
Q07954	Prolow-density lipoprotein receptor-related protein 1	VDIPQQPMGIIAVANDTNSCELSPCR	HexNAc(2)Hex(5)	11.761	8.77E-04
Q07954	Prolow-density lipoprotein receptor-related protein 1	TCVSNCTASQFVCK	HexNAc(2)Hex(5)	9.698	1.02E-03
Q07954	Prolow-density lipoprotein receptor-related protein 1	GVTHLNISGLK	HexNAc(2)Hex(5)	6.638	2.98E-03
Q07954	Prolow-density lipoprotein receptor-related protein 1	CNASSQFLCSSGR	HexNAc(2)Hex(5)	6.457	2.72E-03
Q07954	Prolow-density lipoprotein receptor-related protein 1	FGTCSQLCNNTK	HexNAc(2)Hex(5)	5.073	4.86E-03
Q07954	Prolow-density lipoprotein receptor-related protein 1	DNTTCYEFKK	HexNAc(2)Hex(5)	3.792	8.18E-03
Q07954	Prolow-density lipoprotein receptor-related protein 1	DNTTCYEFK	HexNAc(2)Hex(5)	3.086	1.18E-02
Q07954	Prolow-density lipoprotein receptor-related protein 1	CRDGTCIGNSSR	HexNAc(2)Hex(5)	3.049	1.35E-02
Q07954	Prolow-density lipoprotein receptor-related protein 1	TCPLDEFQCNNTLCKPLAWK	HexNAc(2)Hex(5)	3.015	8.64E-03
Q07954	Prolow-density lipoprotein receptor-related protein 1	TCVSNCTASQFVCK	HexNAc(2)Hex(4)	100	1.00E-17
Q07954	Prolow-density lipoprotein receptor-related protein 1	FNSTEYQVVTR	HexNAc(2)Hex(4)	100	1.00E-17
Q08380	Galectin-3-binding protein	AAIPSALDTNSSK	HexNAc(2)Hex(5)	5.319	4.11E-03
Q08380	Galectin-3-binding protein	AAIPSALDTNSSK	HexNAc(2)Hex(4)	100	1.00E-17
Q08722	Leukocyte surface antigen CD47	SDAVSHTGNYTCEVTELTR	HexNAc(4)Hex(6)NeuAc(1)	100	1.00E-17
Q08722	Leukocyte surface antigen CD47	SDAVSHTGNYTCEVTELTR	HexNAc(4)Hex(5)Fuc(2)	100	1.00E-17
Q08722	Leukocyte surface antigen CD47	SDAVSHTGNYTCEVTELTR	HexNAc(4)Hex(5)Fuc(1)NeuAc(1)	100	1.00E-17
Q08722	Leukocyte surface antigen CD47	SDAVSHTGNYTCEVTELTR	HexNAc(4)Hex(5)Fuc(1)	11.505	3.77E-03
Q08722	Leukocyte surface antigen CD47	SDAVSHTGNYTCEVTELTR	HexNAc(4)Hex(4)Fuc(1)	100	1.00E-17
Q08722	Leukocyte surface antigen CD47	GRDIYTFDGALNK	HexNAc(3)Hex(5)	100	1.00E-17
Q08722	Leukocyte surface antigen CD47	DIYTFDGALNK	HexNAc(2)Hex(7)	2.033	3.22E-02
Q08722	Leukocyte surface antigen CD47	SDAVSHTGNYTCEVTELTR	HexNAc(2)Hex(5)	11.779	7.26E-04
Q08722	Leukocyte surface antigen CD47	DIYTFDGALNK	HexNAc(2)Hex(5)	9.455	1.22E-03
Q08722	Leukocyte surface antigen CD47	GRDIYTFDGALNK	HexNAc(2)Hex(5)	3.126	1.19E-02
Q08AD1	Calmodulin-regulated spectrin-associated protein 2	LNHTDGK	HexNAc(3)Hex(3)	100	1.00E-17
Q0D2J5	Zinc finger protein 763	NLTSIGK	HexNAc(5)Hex(4)Fuc(2)	100	1.00E-17
Q0D2J5	Zinc finger protein 763	NLTSIGK	HexNAc(2)Hex(6)	100	1.00E-17
Q12797	Isoform 7 of Aspartyl/asparaginyl beta-hydroxylase	YNLSEVLQGK	HexNAc(2)Hex(6)	21.804	1.01E-05
Q12797	Isoform 7 of Aspartyl/asparaginyl beta-hydroxylase	YNLSEVLQGK	HexNAc(2)Hex(5)	22.766	9.10E-05
Q12860	Contactin-1	ANSTGTLVITDPTR	HexNAc(6)Hex(4)Fuc(2)	100	1.00E-17
Q12860	Contactin-1	ANSTGTLVITDPTR	HexNAc(6)Hex(3)Fuc(1)	3.487	4.61E-02
Q12860	Contactin-1	ANSTGTLVITDPTR	HexNAc(5)Hex(4)Fuc(2)	7.738	1.29E-03
Q12860	Contactin-1	ANSTGTLVITDPTR	HexNAc(5)Hex(4)Fuc(1)	100	1.00E-17
Q12860	Contactin-1	ANSTGTLVITDPTR*	HexNAc(5)Hex(3)Fuc(1)	100	1.00E-17
Q12860	Contactin-1	GNYSCFVSSPSITK	HexNAc(5)Hex(3)	100	1.00E-17
Q12860	Contactin-1	ANSTGTLVITDPTR	HexNAc(4)Hex(5)Fuc(1)	100	1.00E-17
Q12860	Contactin-1	ANSTGTLVITDPTR	HexNAc(4)Hex(4)Fuc(1)	4.255	4.47E-02
Q12860	Contactin-1	ANSTGTLVITDPTR	HexNAc(2)Hex(9)	100	1.00E-17
Q12860	Contactin-1	YTCTAQTIVDNSSASADLVVR	HexNAc(2)Hex(8)	100	1.00E-17
Q12860	Contactin-1	GTEWLVNSSR	HexNAc(2)Hex(8)	100	1.00E-17
Q12860	Contactin-1	GTEWLVNSSR	HexNAc(2)Hex(7)	11.62	6.78E-04
Q12860	Contactin-1	YTCTAQTIVDNSSASADLVVR	HexNAc(2)Hex(7)	3.544	2.73E-02
Q12860	Contactin-1	GTEWLVNSSR	HexNAc(2)Hex(6)	100	1.00E-17
Q12860	Contactin-1	GNYSCFVSSPSITK	HexNAc(2)Hex(5)	100	1.00E-17
Q12860	Contactin-1	GTEWLVNSSR	HexNAc(2)Hex(5)	100	1.00E-17
Q12860	Contactin-1	ANSTGTLVITDPTR	HexNAc(2)Hex(5)	3.607	9.13E-03
Q13002	Isoform 4 of Glutamate receptor ionotropic, kainate 2	ITFNK	HexNAc(2)Hex(7)	100	1.00E-17
Q13003	Glutamate receptor ionotropic, kainate 3	IVFNK	HexNAc(2)Hex(8)	100	1.00E-17

Q13003	Glutamate receptor ionotropic, kainate 3	IVFNK	HexNAc(2)Hex(6)	100	1.00E-17
Q13201	Multimerin-1	LNQSNFQK	HexNAc(2)Hex(9)	100	1.00E-17
Q13201	Multimerin-1	YQQNMSHLEEK	HexNAc(2)Hex(8)	100	1.00E-17
Q13224	Glutamate receptor ionotropic, NMDA 2B	NLSFSEDGYQMHPK	HexNAc(2)Hex(8)	100	1.00E-17
Q13308	Isoform 6 of Inactive tyrosine-protein kinase 7	DGTPLSDGQSNHTVSSK	HexNAc(5)Hex(5)Fuc(3)	100	1.00E-17
Q13308	Isoform 6 of Inactive tyrosine-protein kinase 7	RQDVNITVATVPSWLK	HexNAc(5)Hex(5)Fuc(1)	100	1.00E-17
Q13308	Isoform 6 of Inactive tyrosine-protein kinase 7	NGTLR	HexNAc(5)Hex(5)	100	1.00E-17
Q13308	Isoform 6 of Inactive tyrosine-protein kinase 7	SANASFNIK	HexNAc(5)Hex(4)Fuc(2)	100	1.00E-17
Q13308	Isoform 6 of Inactive tyrosine-protein kinase 7	SANASFNIK	HexNAc(5)Hex(4)Fuc(1)	100	1.00E-17
Q13308	Isoform 6 of Inactive tyrosine-protein kinase 7	DGTPLSDGQSNHTVSSK	HexNAc(5)Hex(3)Fuc(1)	4.487	2.21E-02
Q13308	Isoform 6 of Inactive tyrosine-protein kinase 7	NLTLR	HexNAc(4)Hex(5)NeuAc(1)	100	1.00E-17
Q13308	Isoform 6 of Inactive tyrosine-protein kinase 7	DGTPLSDGQSNHTVSSK	HexNAc(4)Hex(5)Fuc(1)NeuAc(2)	100	1.00E-17
Q13308	Isoform 6 of Inactive tyrosine-protein kinase 7	NLTLR	HexNAc(3)Hex(6)Fuc(1)	100	1.00E-17
Q13308	Isoform 6 of Inactive tyrosine-protein kinase 7	NLTLR	HexNAc(3)Hex(5)NeuAc(1)	100	1.00E-17
Q13308	Isoform 6 of Inactive tyrosine-protein kinase 7	NLTLR	HexNAc(3)Hex(5)Fuc(1)	100	1.00E-17
Q13308	Isoform 6 of Inactive tyrosine-protein kinase 7	NLTLR	HexNAc(3)Hex(5)	100	1.00E-17
Q13332	Receptor-type tyrosine-protein phosphatase S	VEAEALNATAIR	HexNAc(5)Hex(3)Fuc(1)	100	1.00E-17
Q13349	Integrin alpha-D	VNNLSQR	HexNAc(2)Hex(8)	3.522	4.91E-02
Q13433	Zinc transporter ZIP6	YGENNSLSVEGFR	HexNAc(5)Hex(5)Fuc(3)	100	1.00E-17
Q13433	Zinc transporter ZIP6	YGENNSLSVEGFRK	HexNAc(5)Hex(5)Fuc(3)	9.757	3.31E-03
Q13433	Zinc transporter ZIP6	NTNENPQECFNASK	HexNAc(5)Hex(5)Fuc(2)	100	1.00E-17
Q13433	Zinc transporter ZIP6	NTNENPQECFNASK	HexNAc(5)Hex(4)Fuc(2)	100	1.00E-17
Q13433	Zinc transporter ZIP6	YGENNSLSVEGFRK	HexNAc(5)Hex(3)Fuc(1)	100	1.00E-17
Q13433	Zinc transporter ZIP6	YGENNSLSVEGFR	HexNAc(5)Hex(3)Fuc(1)	100	1.00E-17
Q13433	Zinc transporter ZIP6	NTNENPQECFNASK	HexNAc(5)Hex(3)Fuc(1)	3.911	2.84E-02
Q13433	Zinc transporter ZIP6	NTNENPQECFNASK	HexNAc(4)Hex(5)Fuc(2)	100	1.00E-17
Q13433	Zinc transporter ZIP6	NTNENPQECFNASK	HexNAc(4)Hex(5)Fuc(1)	5.954	2.05E-02
Q13433	Zinc transporter ZIP6	YGENNSLSVEGFRK	HexNAc(2)Hex(8)	100	1.00E-17
Q13433	Zinc transporter ZIP6	NTNENPQECFNASK	HexNAc(2)Hex(5)	100	1.00E-17
Q13439	Isoform 5 of Golgin subfamily A member 4	NQSK	HexNAc(4)Hex(7)Fuc(1)	13.729	1.28E-03
Q13443	Disintegrin and metalloproteinase domain-containing protein 9	NQTAVR	HexNAc(2)Hex(5)	100	1.00E-17
Q13459	Unconventional myosin-IXb	SGAANR	HexNAc(2)Hex(3)	100	1.00E-17
Q13510	Isoform 2 of Acid ceramidase	TVLENSTSYEEAK	HexNAc(2)Hex(8)	2.703	2.52E-02
Q13510	Isoform 2 of Acid ceramidase	MCLNR	HexNAc(2)Hex(5)	5.605	2.56E-02
Q13683	Integrin alpha-7	ANITVK	HexNAc(2)Hex(8)	5.937	1.26E-02
Q13683	Integrin alpha-7	NITLDCAR	HexNAc(2)Hex(8)	2.47	4.98E-02
Q13740	CD166 antigen	EGDNITLK	HexNAc(6)Hex(4)Fuc(2)	100	1.00E-17
Q13740	CD166 antigen	NAIKEGDNITLK	HexNAc(6)Hex(4)Fuc(2)	7.438	9.71E-03
Q13740	CD166 antigen	NAIKEGDNITLK	HexNAc(6)Hex(3)Fuc(1)	100	1.00E-17
Q13740	CD166 antigen	EGDNITLK	HexNAc(6)Hex(3)Fuc(1)	100	1.00E-17
Q13740	CD166 antigen	NATVVWMK	HexNAc(5)Hex(4)Fuc(2)	9.162	5.67E-03
Q13740	CD166 antigen	NATVVWMK	HexNAc(5)Hex(4)Fuc(1)	100	1.00E-17
Q13740	CD166 antigen	RNATGDYK	HexNAc(5)Hex(3)Fuc(1)	100	1.00E-17
Q13740	CD166 antigen	NATGDYK	HexNAc(5)Hex(3)Fuc(1)	100	1.00E-17
Q13740	CD166 antigen	NATVVWMK	HexNAc(5)Hex(3)Fuc(1)	8.805	2.22E-02
Q13740	CD166 antigen	LGDCISEDSYPDGNITWYR	HexNAc(2)Hex(5)	100	1.00E-17
Q14008	Isoform 3 of Cytoskeleton-associated protein 5	LNDSNK	HexNAc(6)Hex(4)Fuc(1)	100	1.00E-17
Q14108	Lysosome membrane protein 2	CNMINGTDGDSFHPLITK	HexNAc(2)Hex(9)	100	1.00E-17
Q14108	Lysosome membrane protein 2	TMVFPVMYLNESVHIDK	HexNAc(2)Hex(9)	4.997	9.87E-03
Q14108	Lysosome membrane protein 2	CNMINGTDGDSFHPLITK	HexNAc(2)Hex(8)	5.036	5.29E-03
Q14108	Lysosome membrane protein 2	CNMINGTDGDSFHPLITK	HexNAc(2)Hex(7)	100	1.00E-17
Q14315	Filamin-C	PNITDNK	HexNAc(6)Hex(3)	100	1.00E-17
Q14517	Protocadherin Fat 1	TGALTVQNTTQLR	HexNAc(2)Hex(5)	100	1.00E-17
Q14644	Ras GTPase-activating protein 3	DNGSK	HexNAc(4)Hex(7)Fuc(1)	6.318	1.09E-02
Q14832	Metabotropic glutamate receptor 3	INFTAPFNPNK	HexNAc(5)Hex(4)Fuc(2)	100	1.00E-17
Q14966	Zinc finger protein 638	KENETPR	HexNAc(3)Hex(6)	100	1.00E-17
Q14982	Isoform 4 of Opioid-binding protein/cell adhesion molecule	DYGNYTCVATNK	HexNAc(5)Hex(4)Fuc(2)	6.311	8.23E-03
Q14982	Isoform 4 of Opioid-binding protein/cell adhesion molecule	DYGNYTCVATNK	HexNAc(5)Hex(3)Fuc(1)	100	1.00E-17
Q14982	Isoform 4 of Opioid-binding protein/cell adhesion molecule	DYGNYTCVATNK	HexNAc(2)Hex(5)	100	1.00E-17
Q14BN4	Isoform 7 of Sarcolemmal membrane-associated protein	RRASNQSGR	HexNAc(2)Hex(5)	100	1.00E-17
Q15149	Plectin	NLVDNITGQR	HexNAc(5)Hex(3)	100	1.00E-17
Q15165	Serum paraoxonase/arylesterase 2	IQNILCEKPTVTTVYANNGSVLQGSSVASVYDGK	HexNAc(2)Hex(6)	100	1.00E-17
Q15165	Serum paraoxonase/arylesterase 2	HTNMNLTQLK	HexNAc(2)Hex(6)	100	1.00E-17
Q15223	Nectin-1	NPNGTVTVISR	HexNAc(4)Hex(6)Fuc(2)	100	1.00E-17
Q15223	Nectin-1	NPNGTVTVISR	HexNAc(4)Hex(6)Fuc(1)	100	1.00E-17
Q15223	Nectin-1	NPNGTVTVISR	HexNAc(4)Hex(5)Fuc(1)	100	1.00E-17
Q15223	Nectin-1	NPNGTVTVISR	HexNAc(4)Hex(5)	100	1.00E-17
Q15223	Nectin-1	NPNGTVTVISR	HexNAc(4)Hex(4)Fuc(1)	100	1.00E-17
Q15223	Nectin-1	NPNGTVTVISR	HexNAc(3)Hex(6)NeuAc(1)	100	1.00E-17
Q15223	Nectin-1	NPNGTVTVISR	HexNAc(3)Hex(6)Fuc(1)	100	1.00E-17
Q15223	Nectin-1	NPNGTVTVISR	HexNAc(2)Hex(5)	100	1.00E-17
Q15223	Nectin-1	GEAEYQEIRNPNGTVTVISR	HexNAc(2)Hex(5)	100	1.00E-17
Q15287	RNA-binding protein with serine-rich domain 1	NVTK	HexNAc(6)Hex(5)Fuc(1)NeuAc(1)	100	1.00E-17

Q15303	Receptor tyrosine-protein kinase erbB-4	FINCTK	HexNAc(3)Hex(6)Fuc(1)	100	1.00E-17
Q15303	Receptor tyrosine-protein kinase erbB-4	FINCTK	HexNAc(3)Hex(5)	100	1.00E-17
Q15303	Receptor tyrosine-protein kinase erbB-4	NLTEILNGGVYVDQNK	HexNAc(2)Hex(8)	100	1.00E-17
Q15303	Receptor tyrosine-protein kinase erbB-4	FINCTK	HexNAc(2)Hex(8)	100	1.00E-17
Q15303	Receptor tyrosine-protein kinase erbB-4	NLTEILNGGVYVDQNK	HexNAc(2)Hex(7)	100	1.00E-17
Q15303	Receptor tyrosine-protein kinase erbB-4	EFENGSICVECDPQCEK	HexNAc(2)Hex(7)	100	1.00E-17
Q15303	Receptor tyrosine-protein kinase erbB-4	FINCTK	HexNAc(2)Hex(7)	100	1.00E-17
Q15303	Receptor tyrosine-protein kinase erbB-4	EFENGSICVECDPQCEK	HexNAc(2)Hex(6)	100	1.00E-17
Q15303	Receptor tyrosine-protein kinase erbB-4	FINCTK	HexNAc(2)Hex(6)	100	1.00E-17
Q15303	Receptor tyrosine-protein kinase erbB-4	ACDGIGTGSLMSAQTVDSSNIDKFINCTK	HexNAc(2)Hex(5)	100	1.00E-17
Q15303	Receptor tyrosine-protein kinase erbB-4	FINCTK	HexNAc(2)Hex(5)	19.026	7.10E-04
Q15652	Probable JmjC domain-containing histone demethylation protein 2C	PNNNLSK	HexNAc(5)Hex(5)Fuc(2)	100	1.00E-17
Q15904	V-type proton ATPase subunit S1	QPVSPVIHPPVSYNDTAPR	HexNAc(5)Hex(4)Fuc(2)	100	1.00E-17
Q15904	V-type proton ATPase subunit S1	QPVSPVIHPPVSYNDTAPR	HexNAc(5)Hex(3)Fuc(1)	5.268	5.22E-03
Q15904	V-type proton ATPase subunit S1	QPVSPVIHPPVSYNDTAPR	HexNAc(4)Hex(4)Fuc(2)	100	1.00E-17
Q15910	Isoform 2 of Histone-lysine N-methyltransferase EZH2	NCSIQR	HexNAc(4)Hex(5)	100	1.00E-17
Q16288	NT-3 growth factor receptor	NPLGTANQTINGHFLK	HexNAc(2)Hex(5)	100	1.00E-17
Q16288	NT-3 growth factor receptor	NITSIHIENWR	HexNAc(2)Hex(5)	5.033	1.60E-02
Q16527	Cysteine and glycine-rich protein 2	PTTNPNTSKFAQK	HexNAc(2)Hex(4)	100	1.00E-17
Q16620	Isoform 4 of BDNF/NT-3 growth factors receptor	NSNLQHINFTR	HexNAc(2)Hex(8)	100	1.00E-17
Q16620	Isoform 4 of BDNF/NT-3 growth factors receptor	NLTIVDSGLK	HexNAc(2)Hex(8)	100	1.00E-17
Q16620	Isoform 4 of BDNF/NT-3 growth factors receptor	NLTIVDSGLK	HexNAc(2)Hex(7)	100	1.00E-17
Q16620	Isoform 4 of BDNF/NT-3 growth factors receptor	NSNLQHINFTR	HexNAc(2)Hex(7)	3.328	4.26E-02
Q16620	Isoform 4 of BDNF/NT-3 growth factors receptor	NSNLQHINFTR	HexNAc(2)Hex(6)	100	1.00E-17
Q16620	Isoform 4 of BDNF/NT-3 growth factors receptor	NLTIVDSGLK	HexNAc(2)Hex(6)	100	1.00E-17
Q16827	Receptor-type tyrosine-protein phosphatase O	GSNTSMLR	HexNAc(2)Hex(8)	3.685	4.69E-02
Q16827	Receptor-type tyrosine-protein phosphatase O	GSNTSMLR	HexNAc(2)Hex(7)	100	1.00E-17
Q16827	Receptor-type tyrosine-protein phosphatase O	VNISYWEGK	HexNAc(2)Hex(7)	9.475	5.24E-03
Q16827	Receptor-type tyrosine-protein phosphatase O	GSNTSMLR	HexNAc(2)Hex(6)	23.416	6.42E-04
Q16827	Receptor-type tyrosine-protein phosphatase O	SLFAVNK	HexNAc(2)Hex(5)	100	1.00E-17
Q16827	Receptor-type tyrosine-protein phosphatase O	GSNTSMLR	HexNAc(2)Hex(5)	100	1.00E-17
Q16851	UTP--glucose-1-phosphate uridylyltransferase	NVSLK	HexNAc(5)Hex(5)Fuc(3)	100	1.00E-17
Q16851	UTP--glucose-1-phosphate uridylyltransferase	NVSLK	HexNAc(5)Hex(4)Fuc(2)	100	1.00E-17
Q16851	UTP--glucose-1-phosphate uridylyltransferase	NVSLK	HexNAc(5)Hex(3)Fuc(1)	100	1.00E-17
Q16851	UTP--glucose-1-phosphate uridylyltransferase	NVSLK	HexNAc(4)Hex(5)Fuc(2)	100	1.00E-17
Q24JP5	Isoform 2 of Transmembrane protein 132A	VQQVGHYPPANSSLSSR	HexNAc(2)Hex(5)	100	1.00E-17
Q2LD37	Uncharacterized protein KIAA1109	GLQTNVSIPK	HexNAc(4)Hex(5)	100	1.00E-17
Q49AM3	Tetratricopeptide repeat protein 31	LFGNR*	HexNAc(5)Hex(3)Fuc(1)	4.62	4.79E-02
Q4KMQ2	Isoform 2 of Anoctamin-6	NINGTDPIQK	HexNAc(2)Hex(5)	100	1.00E-17
Q58EX2	Isoform 4 of Protein sidekick-2	FWLVEGNSSR	HexNAc(2)Hex(6)	100	1.00E-17
Q58EX2	Isoform 4 of Protein sidekick-2	VISAGGNDSR	HexNAc(2)Hex(5)	100	1.00E-17
Q5IJ48	Protein crumbs homolog 2	NGSLAGGVR	HexNAc(5)Hex(6)Fuc(1)	100	1.00E-17
Q5IJ48	Protein crumbs homolog 2	NGSLAGGVR	HexNAc(5)Hex(5)Fuc(1)	100	1.00E-17
Q5IJ48	Protein crumbs homolog 2	NGSLAGGVR	HexNAc(5)Hex(4)Fuc(1)NeuAc(1)	100	1.00E-17
Q5NDL2	EGF domain-specific O-linked N-acetylglucosamine transferase	LNITQEGPK	HexNAc(5)Hex(3)	100	1.00E-17
Q5T0Z8	Uncharacterized protein C6orf132	NNYSDLR	HexNAc(2)Hex(6)	100	1.00E-17
Q5T1H1	Protein eyes shut homolog	LNVTVK	HexNAc(2)Hex(9)	100	1.00E-17
Q5T4D3	Isoform 3 of Transmembrane and TPR repeat-containing protein 4	NATVLK	HexNAc(5)Hex(3)Fuc(1)	100	1.00E-17
Q5T4D3	Isoform 3 of Transmembrane and TPR repeat-containing protein 4	GNQTAAIR	HexNAc(2)Hex(8)	100	1.00E-17
Q5T4D3	Isoform 3 of Transmembrane and TPR repeat-containing protein 4	NLADKGNQTAAIR	HexNAc(2)Hex(8)	5.164	2.82E-02
Q5T4D3	Isoform 3 of Transmembrane and TPR repeat-containing protein 4	NLADKGNQTAAIR	HexNAc(2)Hex(7)	4.292	2.10E-02
Q5T4D3	Isoform 3 of Transmembrane and TPR repeat-containing protein 4	NLADKGNQTAAIR	HexNAc(2)Hex(6)	100	1.00E-17
Q5T4D3	Isoform 3 of Transmembrane and TPR repeat-containing protein 4	GNQTAAIR	HexNAc(2)Hex(6)	35.984	5.20E-05
Q5T4D3	Isoform 3 of Transmembrane and TPR repeat-containing protein 4	NLADKGNQTAAIR	HexNAc(2)Hex(5)	100	1.00E-17
Q5T4D3	Isoform 3 of Transmembrane and TPR repeat-containing protein 4	GNQTAAIR	HexNAc(2)Hex(5)	100	1.00E-17
Q5T4S7	Isoform 2 of E3 ubiquitin-protein ligase UBR4	NSSK	HexNAc(4)Hex(7)NeuAc(1)	100	1.00E-17
Q5T5Y3	Isoform 3 of Calmodulin-regulated spectrin-associated protein 1	LTGTGPKNITK	HexNAc(3)Hex(3)Fuc(2)	100	1.00E-17
Q5UIP0	Telomere-associated protein RIF1	TVNGIENK	HexNAc(3)Hex(5)NeuAc(1)	100	1.00E-17
Q5VU97	VWFA and cache domain-containing protein 1	EAYNVSYAWK	HexNAc(5)Hex(3)	100	1.00E-17
Q5VU97	VWFA and cache domain-containing protein 1	EAYNVSYAWK	HexNAc(2)Hex(5)	100	1.00E-17
Q5VV63	Attractin-like protein 1	NLTTGK	HexNAc(6)Hex(3)Fuc(1)	100	1.00E-17
Q5VV63	Attractin-like protein 1	NLTTGK	HexNAc(5)Hex(3)Fuc(1)	100	1.00E-17
Q5VV63	Attractin-like protein 1	NLTTGK	HexNAc(2)Hex(5)	100	1.00E-17
Q5VYK3	Proteasome-associated protein ECM29 homolog	SLMNNSK	HexNAc(5)Hex(5)NeuAc(1)	6.675	4.95E-03
Q5ZPR3	CD276 antigen	QLVHSFAEGQDQGSAYANR	HexNAc(6)Hex(4)NeuAc(1)	14.048	2.62E-03
Q5ZPR3	CD276 antigen	QLVHSFAEGQDQGSAYANR	HexNAc(6)Hex(4)Fuc(2)	7.977	2.58E-03
Q5ZPR3	CD276 antigen	VVLGANGTYSCLVR	HexNAc(6)Hex(4)Fuc(2)	3.602	4.26E-02
Q5ZPR3	CD276 antigen	VVLGANGTYSCLVR	HexNAc(5)Hex(6)NeuAc(3)	100	1.00E-17
Q5ZPR3	CD276 antigen	VVLGANGTYSCLVR	HexNAc(5)Hex(6)Fuc(1)NeuAc(2)	100	1.00E-17

Q5ZPR3	CD276 antigen	QLVHSFAEGQDQGSAYANR	HexNAc(5)Hex(5)Fuc(3)	100	1.00E-17
Q5ZPR3	CD276 antigen	VVLGANGTYSCLVR	HexNAc(5)Hex(5)Fuc(3)	22.122	1.52E-05
Q5ZPR3	CD276 antigen	QLVHSFAEGQDQGSAYANR	HexNAc(5)Hex(5)Fuc(1)NeuAc(1)	3.711	3.62E-02
Q5ZPR3	CD276 antigen	VVLGANGTYSCLVR	HexNAc(5)Hex(4)Fuc(2)	100	1.00E-17
Q5ZPR3	CD276 antigen	QLVHSFAEGQDQGSAYANR	HexNAc(5)Hex(4)Fuc(2)	7.781	1.76E-03
Q5ZPR3	CD276 antigen	DQGSAYANR	HexNAc(5)Hex(4)Fuc(2)	5.13	1.53E-02
Q5ZPR3	CD276 antigen	QLVHSFAEGQDQGSAYANR	HexNAc(5)Hex(4)Fuc(1)	100	1.00E-17
Q5ZPR3	CD276 antigen	VVLGANGTYSCLVR	HexNAc(5)Hex(3)Fuc(1)	100	1.00E-17
Q5ZPR3	CD276 antigen	DQGSAYANR*	HexNAc(5)Hex(3)Fuc(1)	100	1.00E-17
Q5ZPR3	CD276 antigen	QLVHSFAEGQDQGSAYANR	HexNAc(5)Hex(3)Fuc(1)	14.425	1.58E-04
Q5ZPR3	CD276 antigen	QLVHSFAEGQDQGSAYANR	HexNAc(4)Hex(5)Fuc(4)	100	1.00E-17
Q5ZPR3	CD276 antigen	TALFPDLLAQGNASLR	HexNAc(3)Hex(6)NeuAc(1)	3.454	1.94E-02
Q5ZPR3	CD276 antigen	TALFPDLLAQGNASLR	HexNAc(3)Hex(6)Fuc(1)NeuAc(1)	100	1.00E-17
Q5ZPR3	CD276 antigen	VVLGANGTYSCLVR	HexNAc(3)Hex(6)Fuc(1)NeuAc(1)	8.115	4.99E-03
Q5ZPR3	CD276 antigen	TALFPDLLAQGNASLR	HexNAc(3)Hex(6)Fuc(1)	6.163	2.81E-03
Q5ZPR3	CD276 antigen	TALFPDLLAQGNASLR	HexNAc(2)Hex(7)	5.203	3.80E-03
Q5ZPR3	CD276 antigen	TALFPDLLAQGNASLR	HexNAc(2)Hex(6)	4.942	3.21E-03
Q5ZPR3	CD276 antigen	DQGSAYANR	HexNAc(2)Hex(5)	100	1.00E-17
Q5ZPR3	CD276 antigen	VVLGANGTYSCLVR	HexNAc(2)Hex(5)	4.388	3.16E-02
Q68DQ2	Very large A-kinase anchor protein	FELNR	HexNAc(2)Hex(9)	100	1.00E-17
Q6IQ23	Isoform 2 of Pleckstrin homology domain-containing family A member 7	NSSHVDRR	HexNAc(2)Hex(5)	100	1.00E-17
Q6N022	Teneurin-4	LTNVTFPTGQVSSFR	HexNAc(2)Hex(9)	5.466	1.20E-02
Q6N022	Teneurin-4	NVTLPIDNGLNLVEWR	HexNAc(2)Hex(8)	100	1.00E-17
Q6N022	Teneurin-4	FNVSLGK	HexNAc(2)Hex(7)	100	1.00E-17
Q6N022	Teneurin-4	FNVSLGK	HexNAc(2)Hex(6)	100	1.00E-17
Q6N022	Teneurin-4	VGPYANTTR	HexNAc(2)Hex(5)	100	1.00E-17
Q6N022	Teneurin-4	FNVSLGK	HexNAc(2)Hex(5)	100	1.00E-17
Q6P2E9	Enhancer of mRNA-decapping protein 4	NLTDAIAR	HexNAc(3)Hex(4)Fuc(1)NeuAc(1)	100	1.00E-17
Q6UVY6	DBH-like monooxygenase protein 1	INLTR	HexNAc(4)Hex(5)	100	1.00E-17
Q6UX52	Uncharacterized protein C17orf99	ANFTLQDR	HexNAc(4)Hex(5)	100	1.00E-17
Q6UX52	Uncharacterized protein C17orf99	ANFTLQDR	HexNAc(2)Hex(8)	100	1.00E-17
Q6UXK2	Immunoglobulin superfamily containing leucine-rich repeat protein 2	AHNELGANSTSIR	HexNAc(7)Hex(3)Fuc(1)	100	1.00E-17
Q6UXK2	Immunoglobulin superfamily containing leucine-rich repeat protein 2	AHNELGANSTSIR	HexNAc(6)Hex(4)Fuc(2)	100	1.00E-17
Q6UXK2	Immunoglobulin superfamily containing leucine-rich repeat protein 2	AHNELGANSTSIR	HexNAc(6)Hex(3)Fuc(1)	100	1.00E-17
Q6UXK2	Immunoglobulin superfamily containing leucine-rich repeat protein 2	AHNELGANSTSIR	HexNAc(5)Hex(4)Fuc(2)	3.712	4.20E-02
Q6UXK2	Immunoglobulin superfamily containing leucine-rich repeat protein 2	AHNELGANSTSIR*	HexNAc(5)Hex(3)Fuc(1)	100	1.00E-17
Q6UXK2	Immunoglobulin superfamily containing leucine-rich repeat protein 2	NLSALQLLK	HexNAc(4)Hex(3)NeuAc(1)	100	1.00E-17
Q6UXK2	Immunoglobulin superfamily containing leucine-rich repeat protein 2	AHNELGANSTSIR	HexNAc(2)Hex(9)	100	1.00E-17
Q6UXK2	Immunoglobulin superfamily containing leucine-rich repeat protein 2	AHNELGANSTSIR	HexNAc(2)Hex(8)	100	1.00E-17
Q6UXK2	Immunoglobulin superfamily containing leucine-rich repeat protein 2	AHNELGANSTSIR	HexNAc(2)Hex(5)	100	1.00E-17
Q6UXK5	Leucine-rich repeat neuronal protein 1	SCVNVTTK	HexNAc(5)Hex(4)Fuc(2)	3.94	3.63E-02
Q6UXK5	Leucine-rich repeat neuronal protein 1	SCVNVTTK	HexNAc(5)Hex(4)Fuc(1)	4.502	2.11E-02
Q6UXK5	Leucine-rich repeat neuronal protein 1	SCVNVTTK	HexNAc(5)Hex(3)Fuc(1)	10.591	6.67E-03
Q6V0I7	Protocadherin Fat 4	NGTATVLSVDR	HexNAc(5)Hex(3)Fuc(1)	100	1.00E-17
Q6V0I7	Protocadherin Fat 4	LNITAK	HexNAc(2)Hex(9)	100	1.00E-17
Q6V0I7	Protocadherin Fat 4	INITVSDVNDHTPK	HexNAc(2)Hex(7)	100	1.00E-17
Q6V0I7	Protocadherin Fat 4	LNITAK	HexNAc(2)Hex(6)	100	1.00E-17
Q6ZSG1	E3 ubiquitin-protein ligase RNF165	LGNVTR	HexNAc(5)Hex(4)Fuc(2)	100	1.00E-17
Q6ZSG1	E3 ubiquitin-protein ligase RNF165	LGNVTR	HexNAc(4)Hex(5)Fuc(2)	100	1.00E-17
Q6ZSG1	E3 ubiquitin-protein ligase RNF165	LGNVTR	HexNAc(4)Hex(5)Fuc(1)	100	1.00E-17
Q6ZSG1	E3 ubiquitin-protein ligase RNF165	LGNVTR	HexNAc(2)Hex(6)	100	1.00E-17
Q6ZSY5	Protein phosphatase 1 regulatory subunit 3F	NYTVLLR	HexNAc(3)Hex(4)Fuc(1)NeuAc(1)	3.215	8.53E-03
Q6ZUS6	Isoform 4 of Coiled-coil domain-containing protein 149	PAVNSPANQSR	HexNAc(3)Hex(5)Fuc(1)	100	1.00E-17
Q75T13	GPI inositol-deacylase	LHIAQPENNTHVALFK	HexNAc(2)Hex(9)	2.458	2.00E-02
Q75T13	GPI inositol-deacylase	LHIAQPENNTHVALFK	HexNAc(2)Hex(8)	6.348	9.83E-04
Q75T13	GPI inositol-deacylase	LHIAQPENNTHVALFK	HexNAc(2)Hex(7)	8.077	9.38E-03
Q75V66	Anoctamin-5	LNSTCLASK	HexNAc(2)Hex(6)	100	1.00E-17
Q7L0J3	Synaptic vesicle glycoprotein 2A	LINSTFLHNK	HexNAc(2)Hex(6)	100	1.00E-17
Q7LGA3	Heparan sulfate 2-O-sulfotransferase 1	YHVLHINTTK	HexNAc(4)Hex(5)NeuAc(1)	100	1.00E-17
Q7LGA3	Heparan sulfate 2-O-sulfotransferase 1	NITSWK	HexNAc(4)Hex(4)NeuAc(1)	100	1.00E-17
Q7Z3B1	Neuronal growth regulator 1	GAWLNR	HexNAc(2)Hex(8)	100	1.00E-17
Q7Z3B1	Neuronal growth regulator 1	GAWLNR	HexNAc(2)Hex(7)	29.056	3.07E-06
Q7Z3B1	Neuronal growth regulator 1	GAWLNR	HexNAc(2)Hex(6)	6.16	1.65E-02
Q7Z3B1	Neuronal growth regulator 1	LFNGQQGIIIQNFSTR	HexNAc(2)Hex(5)	100	1.00E-17
Q7Z407	Isoform 2 of CUB and sushi domain-containing protein 3	VVNCSDPGIPANSK	HexNAc(2)Hex(8)	100	1.00E-17
Q7Z408	Isoform 4 of CUB and sushi domain-containing protein 2	IINCTDPGHQENSVR	HexNAc(2)Hex(5)	7.858	2.79E-02
Q7Z4K8	Tripartite motif-containing protein 46	NLTLER	HexNAc(4)Hex(5)Fuc(3)	4.939	4.96E-02
Q7Z553	Isoform 3 of MAM domain-containing glycosylphosphatidylinositol anchor protein 2	IVNVSR	HexNAc(2)Hex(8)	100	1.00E-17

Q7Z553	Isoform 3 of MAM domain-containing glycosylphosphatidylinositol anchor protein 2	IVNVSR	HexNAc(2)Hex(5)	100	1.00E-17
Q7Z5J1	Isoform 2 of Hydroxysteroid 11-beta-dehydrogenase 1-like protein	QELNVTAAAA	HexNAc(2)Hex(6)	100	1.00E-17
Q7Z5N4	Protein sidekick-1	NLTSHTK	HexNAc(6)Hex(4)Fuc(2)	100	1.00E-17
Q7Z5N4	Protein sidekick-1	NLTSHTK	HexNAc(5)Hex(4)Fuc(2)	100	1.00E-17
Q7Z5N4	Protein sidekick-1	TVNSSSTSTMCELTHLK	HexNAc(5)Hex(3)Fuc(1)	100	1.00E-17
Q7Z5N4	Protein sidekick-1	NLTSHTK	HexNAc(2)Hex(5)	5.871	1.85E-02
Q86TE4	Leucine zipper protein 2	DLQNENK	HexNAc(4)Hex(5)Fuc(1)	100	1.00E-17
Q86YD3	Transmembrane protein 25	AQHELNCSLQDPR	HexNAc(2)Hex(5)	100	1.00E-17
Q86YS7	Isoform 5 of C2 domain-containing protein 5	ASPVGDGNFRNR	HexNAc(2)Hex(5)	3.43	4.15E-02
Q8IV08	Phospholipase D3	DNHTHSDIQVK	HexNAc(6)Hex(5)Fuc(1)NeuAc(1)	100	1.00E-17
Q8IV08	Phospholipase D3	DNHTHSDIQVK	HexNAc(5)Hex(5)Fuc(1)NeuAc(2)	100	1.00E-17
Q8IV08	Phospholipase D3	DNHTHSDIQVK	HexNAc(3)Hex(4)Fuc(1)NeuAc(1)	7.912	3.58E-03
Q8IV08	Phospholipase D3	ELGVVMYNCSCLAR	HexNAc(2)Hex(5)	100	1.00E-17
Q8IVF5	Isoform 2 of T-lymphoma invasion and metastasis-inducing protein 2	NESIASTPPGEDR	HexNAc(4)Hex(4)	2.435	1.62E-02
Q8IWA5	Choline transporter-like protein 2	NITDLVEGAK	HexNAc(8)Hex(9)Fuc(1)	100	1.00E-17
Q8IWA5	Choline transporter-like protein 2	NITDLVEGAK	HexNAc(6)Hex(7)Fuc(4)NeuAc(1)	100	1.00E-17
Q8IWA5	Choline transporter-like protein 2	NITDLVEGAK	HexNAc(4)Hex(5)Fuc(3)	100	1.00E-17
Q8IWA5	Choline transporter-like protein 2	NITDLVEGAK	HexNAc(4)Hex(5)Fuc(2)	7.213	8.83E-03
Q8IWA5	Choline transporter-like protein 2	NITDLVEGAK	HexNAc(4)Hex(4)Fuc(1)	4.354	4.00E-02
Q8IWA5	Choline transporter-like protein 2	TCNPETFPSSNESR	HexNAc(2)Hex(5)	100	1.00E-17
Q8IWB1	Inositol 1,4,5-trisphosphate receptor-interacting protein	ENATVPENEEEIIRK	HexNAc(5)Hex(6)Fuc(3)	100	1.00E-17
Q8IWU5	Extracellular sulfatase Sulf-2	FYNYTLCR	HexNAc(2)Hex(9)	100	1.00E-17
Q8IWU5	Extracellular sulfatase Sulf-2	FYNYTLCR	HexNAc(2)Hex(8)	100	1.00E-17
Q8IWU5	Extracellular sulfatase Sulf-2	FYNYTLCR	HexNAc(2)Hex(6)	100	1.00E-17
Q8IWU5	Extracellular sulfatase Sulf-2	FYNYTLCR	HexNAc(2)Hex(5)	100	1.00E-17
Q8IWU5	Extracellular sulfatase Sulf-2	LFPNASQHITPSYNYAPNPDK	HexNAc(2)Hex(5)	3.751	3.25E-02
Q8IXT5	RNA-binding protein 12B	NLSLSIDER	HexNAc(3)Hex(4)NeuAc(1)	4.935	6.39E-03
Q8IYK4	Procollagen galactosyltransferase 2	ALNTSQLK	HexNAc(2)Hex(5)	100	1.00E-17
Q8IYR6	Tomoregulin-1	SINCSELNVR	HexNAc(5)Hex(5)Fuc(3)	4.242	3.07E-02
Q8IYR6	Tomoregulin-1	SINCSELNVR*	HexNAc(5)Hex(3)Fuc(1)	5.537	1.03E-02
Q8IZA0	Dyslexia-associated protein KIAA0319-like protein	ALEVNTVTCQLNCSDHGHCDSFTK	HexNAc(2)Hex(5)	100	1.00E-17
Q8N0W4	Isoform 2 of Neuroligin-4, X-linked	NTTQFAAVCPQHLDER	HexNAc(6)Hex(4)NeuAc(1)	100	1.00E-17
Q8N0W4	Isoform 2 of Neuroligin-4, X-linked	NTTQFAAVCPQHLDER	HexNAc(6)Hex(4)Fuc(2)	100	1.00E-17
Q8N0W4	Isoform 2 of Neuroligin-4, X-linked	NTTQFAAVCPQHLDER	HexNAc(6)Hex(3)Fuc(1)	100	1.00E-17
Q8N0W4	Isoform 2 of Neuroligin-4, X-linked	NTTQFAAVCPQHLDER	HexNAc(5)Hex(5)Fuc(3)	3.993	2.56E-02
Q8N0W4	Isoform 2 of Neuroligin-4, X-linked	NTTQFAAVCPQHLDER	HexNAc(5)Hex(4)Fuc(2)	12.094	2.07E-04
Q8N0W4	Isoform 2 of Neuroligin-4, X-linked	NTTQFAAVCPQHLDER	HexNAc(5)Hex(4)Fuc(1)	100	1.00E-17
Q8N0W4	Isoform 2 of Neuroligin-4, X-linked	NTTQFAAVCPQHLDER	HexNAc(5)Hex(3)Fuc(1)	43.892	8.99E-06
Q8N0W4	Isoform 2 of Neuroligin-4, X-linked	NTTQFAAVCPQHLDER	HexNAc(4)Hex(5)Fuc(2)	100	1.00E-17
Q8N126	Isoform 2 of Cell adhesion molecule 3	MTQESALIFPFLNK	HexNAc(8)Hex(8)	6.214	1.17E-02
Q8N126	Isoform 2 of Cell adhesion molecule 3	MTQESALIFPFLNK	HexNAc(6)Hex(6)Fuc(3)	100	1.00E-17
Q8N126	Isoform 2 of Cell adhesion molecule 3	MTQESALIFPFLNK	HexNAc(6)Hex(6)Fuc(1)NeuAc(1)	100	1.00E-17
Q8N126	Isoform 2 of Cell adhesion molecule 3	MTQESALIFPFLNK	HexNAc(4)Hex(6)Fuc(2)	100	1.00E-17
Q8N126	Isoform 2 of Cell adhesion molecule 3	MTQESALIFPFLNK	HexNAc(3)Hex(6)Fuc(1)NeuAc(1)	100	1.00E-17
Q8N3J6	Isoform 3 of Cell adhesion molecule 2	GSQGQFPLTQNVTVVEGGTAILTCR	HexNAc(2)Hex(5)	7.235	9.83E-03
Q8N608	Isoform 3 of Inactive dipeptidyl peptidase 10	WINDTDVVYK	HexNAc(5)Hex(3)Fuc(1)	3.883	3.96E-02
Q8N6C5	Isoform 4 of Immunoglobulin superfamily member 1	NLTLWCR	HexNAc(2)Hex(7)	100	1.00E-17
Q8N7P1	Inactive phospholipase D5	LFCPKNR	HexNAc(5)Hex(4)Fuc(2)	100	1.00E-17
Q8N7P1	Inactive phospholipase D5	LFCPKNR	HexNAc(5)Hex(3)Fuc(1)	100	1.00E-17
Q8N7P1	Inactive phospholipase D5	LFCPKNR	HexNAc(4)Hex(5)Fuc(2)	100	1.00E-17
Q8N7P1	Inactive phospholipase D5	LFCPKNR	HexNAc(4)Hex(4)Fuc(2)	100	1.00E-17
Q8N7P1	Inactive phospholipase D5	LFCPKNR	HexNAc(4)Hex(4)Fuc(1)	6.473	2.28E-02
Q8N7P1	Inactive phospholipase D5	LFCPKNR	HexNAc(4)Hex(3)Fuc(1)	100	1.00E-17
Q8N7P1	Inactive phospholipase D5	LFCPKNR	HexNAc(3)Hex(4)Fuc(2)	100	1.00E-17
Q8NBJ4	Golgi membrane protein 1	AVLVNNITTGER	HexNAc(5)Hex(4)Fuc(1)NeuAc(2)	100	1.00E-17
Q8NCS7	Choline transporter-like protein 5	NGTLTIGSK	HexNAc(2)Hex(6)	100	1.00E-17
Q8NFC6	Biorientation of chromosomes in cell division protein 1-like 1	NESK	HexNAc(5)Hex(4)Fuc(1)NeuAc(1)	100	1.00E-17
Q8NFC6	Biorientation of chromosomes in cell division protein 1-like 1	NESK	HexNAc(3)Hex(3)Fuc(2)	11.302	1.66E-02
Q8NFP4	Isoform 2 of MAM domain-containing glycosylphosphatidylinositol anchor protein 1	LTNTTAPPALK	HexNAc(5)Hex(4)Fuc(2)	100	1.00E-17
Q8NFP4	Isoform 2 of MAM domain-containing glycosylphosphatidylinositol anchor protein 1	LTNTTAPPALK	HexNAc(5)Hex(3)Fuc(1)	100	1.00E-17
Q8NFP4	Isoform 2 of MAM domain-containing glycosylphosphatidylinositol anchor protein 1	NATFQITPDVIK	HexNAc(2)Hex(9)	100	1.00E-17
Q8NFP4	Isoform 2 of MAM domain-containing glycosylphosphatidylinositol anchor protein 1	DSGYYNCTATNNVGNPAK	HexNAc(2)Hex(9)	100	1.00E-17
Q8NFP4	Isoform 2 of MAM domain-containing glycosylphosphatidylinositol anchor protein 1	DSGYYNCTATNNVGNPAK	HexNAc(2)Hex(8)	5.855	2.63E-02
Q8NFP4	Isoform 2 of MAM domain-containing glycosylphosphatidylinositol anchor protein 1	LVSPLYNASAK	HexNAc(2)Hex(5)	100	1.00E-17
Q8NFQ8	Torsin-1A-interacting protein 2	HLNASNPTEPATIIFTAAR	HexNAc(2)Hex(6)	100	1.00E-17
Q8NFT8	Delta and Notch-like epidermal growth factor-related receptor	LVSFEVPQNTSVK	HexNAc(6)Hex(4)Fuc(2)	100	1.00E-17
Q8NFT8	Delta and Notch-like epidermal growth factor-related receptor	KPCQNNASCIDANEK	HexNAc(5)Hex(4)Fuc(2)	100	1.00E-17

Q8NFT8	Delta and Notch-like epidermal growth factor-related receptor	KPCQNNASCIDANEK	HexNAc(5)Hex(3)Fuc(1)	100	1.00E-17
Q8NFT8	Delta and Notch-like epidermal growth factor-related receptor	KPCQNNASCIDANEK	HexNAc(2)Hex(9)	100	1.00E-17
Q8NFT8	Delta and Notch-like epidermal growth factor-related receptor	KPCQNNASCIDANEK	HexNAc(2)Hex(8)	100	1.00E-17
Q8NFZ8	Cell adhesion molecule 4	QTLFFNGTR	HexNAc(4)Hex(6)Fuc(2)	5.818	1.54E-02
Q8NFZ8	Cell adhesion molecule 4	QTLFFNGTR	HexNAc(4)Hex(6)Fuc(1)	100	1.00E-17
Q8NHE4	Isoform 4 of V-type proton ATPase subunit e 2	NETIWYVR	HexNAc(2)Hex(9)	10.936	5.76E-04
Q8NHE4	Isoform 4 of V-type proton ATPase subunit e 2	NETIWYVR	HexNAc(2)Hex(8)	100	1.00E-17
Q8NHE4	Isoform 4 of V-type proton ATPase subunit e 2	NETIWYVR	HexNAc(2)Hex(7)	100	1.00E-17
Q8NHE4	Isoform 4 of V-type proton ATPase subunit e 2	NETIWYVR	HexNAc(2)Hex(6)	4.185	9.09E-03
Q8NI08	Nuclear receptor coactivator 7	FNITPNK	HexNAc(5)Hex(6)Fuc(1)	100	1.00E-17
Q8NI27	THO complex subunit 2	NETITK	HexNAc(5)Hex(5)Fuc(1)	100	1.00E-17
Q8TAB3	Protocadherin-19	EQHDQYNLTIQAR	HexNAc(5)Hex(3)Fuc(1)	100	1.00E-17
Q8TAB3	Protocadherin-19	DGGLPSLQSNATVR	HexNAc(5)Hex(3)Fuc(1)	8.999	7.56E-03
Q8TAB3	Protocadherin-19	DGGLPSLQSNATVR	HexNAc(2)Hex(5)	2.692	2.79E-02
Q8TCT7	Signal peptide peptidase-like 2B	LVPPGGNK	HexNAc(2)Hex(5)	100	1.00E-17
Q8TCT8	Signal peptide peptidase-like 2A	DMNQTLGDNITVK	HexNAc(2)Hex(8)	100	1.00E-17
Q8TDW7	Protocadherin Fat 3	HESEVNVTIR	HexNAc(5)Hex(3)Fuc(1)	100	1.00E-17
Q8TDW7	Protocadherin Fat 3	ANYSLK	HexNAc(2)Hex(8)	100	1.00E-17
Q8TDW7	Protocadherin Fat 3	YVLNVSVSDGR	HexNAc(2)Hex(7)	100	1.00E-17
Q8TDW7	Protocadherin Fat 3	QQFYNLTVR	HexNAc(2)Hex(6)	100	1.00E-17
Q8TDW7	Protocadherin Fat 3	ANYSLK	HexNAc(2)Hex(6)	8.502	3.59E-03
Q8TDW7	Protocadherin Fat 3	QQFYNLTVR	HexNAc(2)Hex(5)	100	1.00E-17
Q8WTV0	Scavenger receptor class B member 1	AFMNR	HexNAc(4)Hex(3)Fuc(1)	100	1.00E-17
Q8WVV9	Heterogeneous nuclear ribonucleoprotein L-like	MNCSR	HexNAc(3)Hex(4)Fuc(1)NeuAc(1)	100	1.00E-17
Q8WX77	Insulin-like growth factor-binding protein-like 1	SVHNVTGAQVGLSCEVR	HexNAc(2)Hex(5)	100	1.00E-17
Q8WY21	Isoform 2 of VPS10 domain-containing receptor SorCS1	VVSNNCTDGVR	HexNAc(2)Hex(7)	100	1.00E-17
Q8WY21	Isoform 2 of VPS10 domain-containing receptor SorCS1	DCSLGQSYLNSTGYRK	HexNAc(2)Hex(5)	4.094	3.87E-02
Q8WYK1	Contactin-associated protein-like 5	QVNFTVDK	HexNAc(2)Hex(5)	100	1.00E-17
Q8WYK1	Contactin-associated protein-like 5	NGSLQVR	HexNAc(2)Hex(5)	100	1.00E-17
Q8WYK1	Contactin-associated protein-like 5	IWTSVQHNNTELTR	HexNAc(2)Hex(5)	100	1.00E-17
Q8WYP5	Isoform 2 of Protein ELYS	PKSSSTALTTNVTEQTEK	HexNAc(2)Hex(3)	3.899	3.42E-02
Q92608	Dedicator of cytokinesis protein 2	TVNR	HexNAc(5)Hex(5)Fuc(3)	100	1.00E-17
Q92608	Dedicator of cytokinesis protein 2	TVNR	HexNAc(5)Hex(4)Fuc(2)	100	1.00E-17
Q92608	Dedicator of cytokinesis protein 2	TVNR	HexNAc(4)Hex(5)Fuc(1)	13.032	1.30E-03
Q92609	Isoform 2 of TBC1 domain family member 5	GADLMNK	HexNAc(4)Hex(5)Fuc(2)	4.372	2.92E-02
Q92673	Sortilin-related receptor	NNTCVK	HexNAc(5)Hex(3)Fuc(1)	100	1.00E-17
Q92820	Gamma-glutamyl hydrolase	NFTMNEK	HexNAc(2)Hex(8)	100	1.00E-17
Q92820	Gamma-glutamyl hydrolase	NFTMNEK	HexNAc(2)Hex(7)	4.675	2.88E-02
Q92820	Gamma-glutamyl hydrolase	NFTMNEK	HexNAc(2)Hex(6)	3.969	1.08E-02
Q92820	Gamma-glutamyl hydrolase	NFTMNEK	HexNAc(2)Hex(5)	8.089	1.15E-02
Q92823	Neuronal cell adhesion molecule	DGDDEWTSVVVANVSK	HexNAc(5)Hex(5)Fuc(3)	100	1.00E-17
Q92823	Neuronal cell adhesion molecule	IPANK	HexNAc(5)Hex(4)Fuc(2)	10.458	8.75E-03
Q92823	Neuronal cell adhesion molecule	IPANK	HexNAc(5)Hex(3)Fuc(1)	66.389	2.49E-05
Q92823	Neuronal cell adhesion molecule	IPANK	HexNAc(4)Hex(5)Fuc(2)	6.456	2.61E-02
Q92823	Neuronal cell adhesion molecule	IPANK	HexNAc(4)Hex(5)Fuc(1)	5.506	2.05E-02
Q92823	Neuronal cell adhesion molecule	IPANK	HexNAc(4)Hex(4)Fuc(1)	100	1.00E-17
Q92823	Neuronal cell adhesion molecule	FNHTQTIQQK	HexNAc(2)Hex(8)	9.457	6.98E-03
Q92823	Neuronal cell adhesion molecule	FNHTQTIQQK	HexNAc(2)Hex(7)	9.554	2.53E-04
Q92823	Neuronal cell adhesion molecule	FNHTQTIQQK	HexNAc(2)Hex(6)	19.83	1.96E-04
Q92823	Neuronal cell adhesion molecule	FNHTQTIQQK	HexNAc(2)Hex(5)	45.652	3.12E-06
Q92823	Neuronal cell adhesion molecule	NLNFSTR	HexNAc(2)Hex(5)	10.414	1.10E-03
Q92823	Neuronal cell adhesion molecule	VNVVNSTLAEVHWDPVPLK	HexNAc(2)Hex(5)	7.523	9.00E-03
Q92854	Semaphorin-4D	AANYTSSLNLPDK	HexNAc(5)Hex(3)Fuc(1)	100	1.00E-17
Q92859	Neogenin	TPASDPHGDNLTYSVFYTK	HexNAc(6)Hex(4)Fuc(2)	100	1.00E-17
Q92859	Neogenin	TPASDPHGDNLTYSVFYTK	HexNAc(5)Hex(4)NeuAc(1)	8.544	1.65E-03
Q92859	Neogenin	TPASDPHGDNLTYSVFYTK	HexNAc(5)Hex(4)Fuc(2)	100	1.00E-17
Q92859	Neogenin	TLSDVPSAAPQNLSLEVR	HexNAc(5)Hex(4)Fuc(2)	100	1.00E-17
Q92859	Neogenin	TPASDPHGDNLTYSVFYTK	HexNAc(5)Hex(3)Fuc(1)	100	1.00E-17
Q92859	Neogenin	TLSDVPSAAPQNLSLEVR	HexNAc(5)Hex(3)Fuc(1)	15.205	3.72E-04
Q92879	Isoform 4 of CUGBP Elav-like family member 1	NDSK	HexNAc(6)Hex(6)NeuAc(1)	100	1.00E-17
Q92896	Isoform 2 of Golgi apparatus protein 1	LNLTTDPK	HexNAc(5)Hex(4)Fuc(1)	100	1.00E-17
Q92896	Isoform 2 of Golgi apparatus protein 1	LNLTTDPK	HexNAc(4)Hex(7)Fuc(2)	5.899	1.64E-02
Q92896	Isoform 2 of Golgi apparatus protein 1	LNLTTDPK	HexNAc(4)Hex(6)Fuc(2)	4.404	2.77E-02
Q92896	Isoform 2 of Golgi apparatus protein 1	LNLTTDPK	HexNAc(4)Hex(6)Fuc(1)	100	1.00E-17
Q92896	Isoform 2 of Golgi apparatus protein 1	LNLTTDPK	HexNAc(4)Hex(5)Fuc(2)	3.965	3.39E-02
Q92896	Isoform 2 of Golgi apparatus protein 1	GNITEYQCHQYITK	HexNAc(4)Hex(5)Fuc(1)NeuAc(1)	100	1.00E-17
Q92896	Isoform 2 of Golgi apparatus protein 1	GNITEYQCHQYITK	HexNAc(4)Hex(4)Fuc(1)NeuAc(1)	16.875	1.60E-03
Q92896	Isoform 2 of Golgi apparatus protein 1	LNLTTDPK	HexNAc(2)Hex(5)	100	1.00E-17
Q969P0	Immunoglobulin superfamily member 8	IGPGEPLELLCNVSGALPPAGR	HexNAc(2)Hex(5)	5.519	2.64E-02
Q96G97	Isoform 3 of Seipin	TDCDSSTTSLCSFPVANVSLTK	HexNAc(2)Hex(8)	2.405	1.95E-02
Q96G97	Isoform 3 of Seipin	TDCDSSTTSLCSFPVANVSLTK	HexNAc(2)Hex(6)	4.325	9.33E-03
Q96G97	Isoform 3 of Seipin	TDCDSSTTSLCSFPVANVSLTK	HexNAc(2)Hex(5)	8.288	1.75E-03
Q96HE7	ERO1-like protein alpha	WGHNITEFQQR	HexNAc(2)Hex(6)	2.427	2.08E-02

Q96HE7	ERO1-like protein alpha	WGHNITEFQQR	HexNAc(2)Hex(5)	5.482	1.63E-02
Q96ID5	Immunoglobulin superfamily member 21	VPAELNGSMYR	HexNAc(2)Hex(6)	100	1.00E-17
Q96ID5	Immunoglobulin superfamily member 21	VPAELNGSMYR	HexNAc(2)Hex(5)	100	1.00E-17
Q96JE9	Microtubule-associated protein 6	NESPVISAPVK	HexNAc(4)Hex(5)	100	1.00E-17
Q96JE9	Microtubule-associated protein 6	NESPVISAPVK	HexNAc(3)Hex(3)Fuc(1)	100	1.00E-17
Q96JJ7	Protein disulfide-isomerase TMX3	LVALAVIDEKNTSVEHTR	HexNAc(2)Hex(6)	100	1.00E-17
Q96KA5	Cleft lip and palate transmembrane protein 1-like protein	DLMVINR	HexNAc(2)Hex(6)	100	1.00E-17
Q96M96	FYVE, RhoGEF and PH domain-containing protein 4	NMTER	HexNAc(2)Hex(8)	100	1.00E-17
Q96MM7	Isoform 4 of Heparan-sulfate 6-O-sulfotransferase 2	YNFTR	HexNAc(5)Hex(4)Fuc(2)	100	1.00E-17
Q96MM7	Isoform 4 of Heparan-sulfate 6-O-sulfotransferase 2	YNFTR*	HexNAc(5)Hex(3)Fuc(1)	100	1.00E-17
Q96MM7	Isoform 4 of Heparan-sulfate 6-O-sulfotransferase 2	YNFTR	HexNAc(3)Hex(3)	2.332	2.91E-02
Q96MM7	Isoform 4 of Heparan-sulfate 6-O-sulfotransferase 2	YNFTR	HexNAc(2)Hex(9)	100	1.00E-17
Q96MM7	Isoform 4 of Heparan-sulfate 6-O-sulfotransferase 2	YNFTR	HexNAc(2)Hex(6)	100	1.00E-17
Q96N03	V-set and transmembrane domain-containing protein 2-like protein	VVGSNISHK	HexNAc(2)Hex(8)	100	1.00E-17
Q96NI6	Leucine-rich repeat and fibronectin type-III domain-containing protein 5	LISNATR	HexNAc(2)Hex(8)	100	1.00E-17
Q96NI6	Leucine-rich repeat and fibronectin type-III domain-containing protein 5	LISNATR	HexNAc(2)Hex(7)	100	1.00E-17
Q96NI6	Leucine-rich repeat and fibronectin type-III domain-containing protein 5	LISNATR	HexNAc(2)Hex(6)	5.094	1.65E-02
Q96NI6	Leucine-rich repeat and fibronectin type-III domain-containing protein 5	LISNATR	HexNAc(2)Hex(5)	100	1.00E-17
Q96PQ0	VPS10 domain-containing receptor SorCS2	YVTCAIHNCSEK	HexNAc(5)Hex(3)Fuc(1)	100	1.00E-17
Q96PQ0	VPS10 domain-containing receptor SorCS2	YVTCAIHNCSEK	HexNAc(2)Hex(8)	100	1.00E-17
Q96PY6	Isoform 3 of Serine/threonine-protein kinase Nek1	LGPNGSPR	HexNAc(4)Hex(4)Fuc(1)	12.185	3.82E-04
Q96QE3	ATPase family AAA domain-containing protein 5	AANLSEK	HexNAc(5)Hex(3)Fuc(1)	100	1.00E-17
Q96QF7	Acidic repeat-containing protein	IYDLFNR	HexNAc(5)Hex(4)Fuc(2)	100	1.00E-17
Q96QF7	Acidic repeat-containing protein	IYDLFNR	HexNAc(3)Hex(6)Fuc(1)NeuAc(1)	5.774	1.97E-02
Q96RE7	Nucleus accumbens-associated protein 1	DLFNNSR	HexNAc(2)Hex(9)	100	1.00E-17
Q96SZ4	Zinc finger and SCAN domain-containing protein 10	NSSNLAR	HexNAc(7)Hex(3)Fuc(1)	100	1.00E-17
Q96SZ4	Zinc finger and SCAN domain-containing protein 10	NSSNLAR	HexNAc(6)Hex(3)Fuc(1)	100	1.00E-17
Q99435	Isoform 3 of Protein kinase C-binding protein NELL2	QVPGLHNGTK	HexNAc(2)Hex(6)	5.912	1.89E-02
Q99435	Isoform 3 of Protein kinase C-binding protein NELL2	QVPGLHNGTK	HexNAc(2)Hex(5)	4.811	2.80E-02
Q99523	Sortilin	DITDLINNTFIR	HexNAc(5)Hex(4)Fuc(2)	5.95	8.91E-03
Q99523	Sortilin	DITDLINNTFIR	HexNAc(2)Hex(5)	100	1.00E-17
Q99574	Neuroserpin	WVENNTNNLVK	HexNAc(6)Hex(3)Fuc(1)	100	1.00E-17
Q99574	Neuroserpin	DANLTGLSDNK	HexNAc(5)Hex(3)Fuc(1)	6.188	1.72E-02
Q99574	Neuroserpin	DANLTGLSDNKEIFLSK	HexNAc(5)Hex(3)Fuc(1)	5.898	8.89E-03
Q99574	Neuroserpin	WVENNTNNLVK	HexNAc(5)Hex(3)Fuc(1)	2.892	4.37E-02
Q99574	Neuroserpin	WVENNTNNLVK	HexNAc(3)Hex(3)Fuc(1)	100	1.00E-17
Q99574	Neuroserpin	WVENNTNNLVK	HexNAc(2)Hex(8)	100	1.00E-17
Q99574	Neuroserpin	WVENNTNNLVK	HexNAc(2)Hex(7)	100	1.00E-17
Q99574	Neuroserpin	DANLTGLSDNK	HexNAc(2)Hex(6)	100	1.00E-17
Q99574	Neuroserpin	WVENNTNNLVK	HexNAc(2)Hex(5)	100	1.00E-17
Q99784	Noelin	VQNMSQSIEVLDRR	HexNAc(2)Hex(8)	9.039	6.71E-03
Q99784	Noelin	VQNMSQSIEVLDRR	HexNAc(2)Hex(7)	100	1.00E-17
Q99784	Noelin	VQNMSQSIEVLDRR	HexNAc(2)Hex(6)	100	1.00E-17
Q99784	Noelin	VQNMSQSIEVLDRR	HexNAc(2)Hex(5)	4.413	2.01E-02
Q99985	Semaphorin-3C	VIQTFNR	HexNAc(2)Hex(7)	100	1.00E-17
Q99985	Semaphorin-3C	VIQTFNR	HexNAc(2)Hex(5)	100	1.00E-17
Q9BTY2	Plasma alpha-L-fucosidase	SQNDTVTPDVWYTSKPK	HexNAc(2)Hex(3)	100	1.00E-17
Q9BX67	Junctional adhesion molecule C	IWNVTR	HexNAc(5)Hex(6)Fuc(1)NeuAc(2)	100	1.00E-17
Q9BX67	Junctional adhesion molecule C	IWNVTR	HexNAc(5)Hex(5)Fuc(3)	100	1.00E-17
Q9BX67	Junctional adhesion molecule C	IWNVTR	HexNAc(5)Hex(4)Fuc(2)	100	1.00E-17
Q9BX67	Junctional adhesion molecule C	IWNVTR	HexNAc(4)Hex(5)Fuc(3)	100	1.00E-17
Q9BXF3	Cat eye syndrome critical region protein 2	NVSSIPGK	HexNAc(2)Hex(7)	100	1.00E-17
Q9BY67	Isoform 5 of Cell adhesion molecule 1	DVTVIEGEVATISCQVNK	HexNAc(6)Hex(4)Fuc(2)	100	1.00E-17
Q9BY67	Isoform 5 of Cell adhesion molecule 1	VSLTNVSISDEGR	HexNAc(2)Hex(9)	100	1.00E-17
Q9BY67	Isoform 5 of Cell adhesion molecule 1	DVTVIEGEVATISCQVNK	HexNAc(2)Hex(9)	100	1.00E-17
Q9BYH1	Seizure 6-like protein	MTVHSGQTNK	HexNAc(2)Hex(9)	100	1.00E-17
Q9BYH1	Seizure 6-like protein	MTVHSGQTNK	HexNAc(2)Hex(5)	100	1.00E-17
Q9BZC7	Isoform 3 of ATP-binding cassette sub-family A member 2	IRQNSSFTEK	HexNAc(2)Hex(9)	100	1.00E-17
Q9BZC7	Isoform 3 of ATP-binding cassette sub-family A member 2	GNPAAYGITVTNHPMNK	HexNAc(2)Hex(9)	100	1.00E-17
Q9BZM5	UL16-binding protein 6	TFLHYDCGNK	HexNAc(2)Hex(5)	100	1.00E-17
Q9C019	Tripartite motif-containing protein 15	VNQSR	HexNAc(5)Hex(3)Fuc(1)	100	1.00E-17
Q9C0A0	Contactin-associated protein-like 4	QVNFTVDEHR	HexNAc(2)Hex(7)	100	1.00E-17
Q9C0A0	Contactin-associated protein-like 4	QVNFTVDEHR	HexNAc(2)Hex(6)	100	1.00E-17
Q9C0B6	BMP/retinoic acid-inducible neural-specific protein 2	TNVDAAAQCQNWTITLGNR	HexNAc(2)Hex(6)	100	1.00E-17
Q9C0C4	Semaphorin-4C	NNQTECFNFIR*	HexNAc(5)Hex(3)Fuc(1)	100	1.00E-17

Q9C0H2	Isoform 4 of Protein tweety homolog 3	VWDTAVGLNHTAEPSLQTLER	HexNAc(4)Hex(3)Fuc(1)	100	1.00E-17
Q9C0H2	Isoform 4 of Protein tweety homolog 3	VWDTAVGLNHTAEPSLQTLER	HexNAc(3)Hex(4)Fuc(1)NeuAc(1)	11.36	6.23E-03
Q9GZZ0	Homeobox protein Hox-D1	TNFSTK	HexNAc(4)Hex(5)NeuAc(1)	94.357	6.24E-05
Q9H0X4	Protein FAM234A	YKPDTLAVAVENGTGTDR	HexNAc(5)Hex(3)	100	1.00E-17
Q9H1J7	Protein Wnt-5b	WNCSTADNASVFGR	HexNAc(2)Hex(9)	100	1.00E-17
Q9H1J7	Protein Wnt-5b	WNCSTADNASVFGR	HexNAc(2)Hex(8)	100	1.00E-17
Q9H2D6	TRIO and F-actin-binding protein	NSSPHR	HexNAc(3)Hex(6)Fuc(1)NeuAc(1)	6.083	1.20E-02
Q9H2E6	Isoform 2 of Semaphorin-6A	NTTQRHR	HexNAc(5)Hex(3)Fuc(1)	100	1.00E-17
Q9H330	Transmembrane protein 245	ILGDKVNNTAVIEK	HexNAc(2)Hex(7)	2.762	2.04E-02
Q9H330	Transmembrane protein 245	ILGDKVNNTAVIEK	HexNAc(2)Hex(6)	10.624	6.95E-04
Q9H330	Transmembrane protein 245	VNNTAVIEK	HexNAc(2)Hex(6)	2.889	4.20E-02
Q9H330	Transmembrane protein 245	VNNTAVIEK	HexNAc(2)Hex(5)	9.409	8.42E-03
Q9H7E2	Isoform 3 of Tudor domain-containing protein 3	NDTR	HexNAc(4)Hex(7)NeuAc(1)	100	1.00E-17
Q9H8M5	Metal transporter CNNM2	VYGQNINNETWSR	HexNAc(5)Hex(3)Fuc(1)	100	1.00E-17
Q9H9Q2	COP9 signalosome complex subunit 7b	ANQYKENHNR	HexNAc(3)Hex(5)	4.982	2.18E-02
Q9HAR2	Isoform 4 of Adhesion G protein-coupled receptor L3	NLTPGGK	HexNAc(5)Hex(5)Fuc(1)	100	1.00E-17
Q9HAR2	Isoform 4 of Adhesion G protein-coupled receptor L3	NLTPGGK	HexNAc(5)Hex(4)Fuc(2)	100	1.00E-17
Q9HAR2	Isoform 4 of Adhesion G protein-coupled receptor L3	NLTPGGK	HexNAc(5)Hex(3)Fuc(1)	12.493	7.80E-03
Q9HAR2	Isoform 4 of Adhesion G protein-coupled receptor L3	NLTPGGK	HexNAc(4)Hex(5)Fuc(1)	100	1.00E-17
Q9HAR2	Isoform 4 of Adhesion G protein-coupled receptor L3	NLTPGGK	HexNAc(4)Hex(5)	100	1.00E-17
Q9HAR2	Isoform 4 of Adhesion G protein-coupled receptor L3	NLTPGGK	HexNAc(4)Hex(4)Fuc(1)	100	1.00E-17
Q9HAR2	Isoform 4 of Adhesion G protein-coupled receptor L3	NLTPGGK	HexNAc(4)Hex(3)Fuc(1)	100	1.00E-17
Q9HAR2	Isoform 4 of Adhesion G protein-coupled receptor L3	LLTTNK	HexNAc(2)Hex(9)	52.984	5.40E-06
Q9HAR2	Isoform 4 of Adhesion G protein-coupled receptor L3	QSEENFNPNCSFWSYSK	HexNAc(2)Hex(8)	100	1.00E-17
Q9HAR2	Isoform 4 of Adhesion G protein-coupled receptor L3	LLTTNK	HexNAc(2)Hex(8)	36.678	4.14E-06
Q9HAR2	Isoform 4 of Adhesion G protein-coupled receptor L3	QSEENFNPNCSFWSYSK	HexNAc(2)Hex(7)	100	1.00E-17
Q9HAR2	Isoform 4 of Adhesion G protein-coupled receptor L3	LLTTNK	HexNAc(2)Hex(7)	31.721	8.01E-04
Q9HAR2	Isoform 4 of Adhesion G protein-coupled receptor L3	LLTTNK	HexNAc(2)Hex(6)	100	1.00E-17
Q9HAR2	Isoform 4 of Adhesion G protein-coupled receptor L3	NLTPGGK	HexNAc(2)Hex(5)	100	1.00E-17
Q9HC56	Protocadherin-9	SNVSFDR	HexNAc(5)Hex(3)Fuc(1)	100	1.00E-17
Q9HC56	Protocadherin-9	IVASDSGKPSLNQTALVR	HexNAc(2)Hex(5)	100	1.00E-17
Q9HCK4	Isoform 3 of Roundabout homolog 2	FHINK	HexNAc(5)Hex(4)Fuc(2)	100	1.00E-17
Q9HCK4	Isoform 3 of Roundabout homolog 2	FHINK	HexNAc(5)Hex(3)	100	1.00E-17
Q9HCK4	Isoform 3 of Roundabout homolog 2	FHINK	HexNAc(4)Hex(5)	100	1.00E-17
Q9HCK4	Isoform 3 of Roundabout homolog 2	FHINK	HexNAc(4)Hex(4)Fuc(1)	100	1.00E-17
Q9HCK4	Isoform 3 of Roundabout homolog 2	FHINK	HexNAc(2)Hex(7)	100	1.00E-17
Q9HCK4	Isoform 3 of Roundabout homolog 2	IWCLGNETR	HexNAc(2)Hex(7)	100	1.00E-17
Q9HCK4	Isoform 3 of Roundabout homolog 2	FHINK	HexNAc(2)Hex(6)	100	1.00E-17
Q9HCK4	Isoform 3 of Roundabout homolog 2	IWCLGNETR	HexNAc(2)Hex(6)	100	1.00E-17
Q9HCK4	Isoform 3 of Roundabout homolog 2	IWCLGNETR	HexNAc(2)Hex(5)	100	1.00E-17
Q9HCK4	Isoform 3 of Roundabout homolog 2	FHINK	HexNAc(2)Hex(5)	12.921	2.60E-03
Q9HCK4	Isoform 3 of Roundabout homolog 2	FHINK	HexNAc(2)Hex(4)	100	1.00E-17
Q9HCK4	Isoform 3 of Roundabout homolog 2	FHINK	HexNAc(2)Hex(3)	100	1.00E-17
Q9HCM1	Uncharacterized protein KIAA1551	QTPTVVESAETNK	HexNAc(3)Hex(4)	3.021	4.19E-02
Q9HCM2	Plexin-A4	NHSLAFVGTK	HexNAc(2)Hex(7)	8.003	1.86E-02
Q9HCM2	Plexin-A4	NHSLAFVGTK	HexNAc(2)Hex(6)	100	1.00E-17
Q9HCM2	Plexin-A4	NHSLAFVGTK	HexNAc(2)Hex(5)	21.235	6.94E-04
Q9HCM3	UPF0606 protein KIAA1549	ATVAAGNSVVQVVNVSR	HexNAc(2)Hex(5)	4.808	2.66E-02
Q9HCU4	Cadherin EGF LAG seven-pass G-type receptor 2	LQRNESGLDSGR	HexNAc(5)Hex(4)Fuc(2)	100	1.00E-17
Q9HCU4	Cadherin EGF LAG seven-pass G-type receptor 2	LQRNESGLDSGR	HexNAc(5)Hex(3)Fuc(1)	100	1.00E-17
Q9HCU4	Cadherin EGF LAG seven-pass G-type receptor 2	NESGLDSGR	HexNAc(4)Hex(3)	100	1.00E-17
Q9HCU4	Cadherin EGF LAG seven-pass G-type receptor 2	NATQHTAGYFGSDVK	HexNAc(2)Hex(8)	3.893	2.69E-02
Q9HD45	Transmembrane 9 superfamily member 3	IVDVNLTSEGK	HexNAc(5)Hex(4)NeuAc(1)	100	1.00E-17
Q9HD45	Transmembrane 9 superfamily member 3	IVDVNLTSEGK	HexNAc(5)Hex(4)	100	1.00E-17
Q9HD45	Transmembrane 9 superfamily member 3	IVDVNLTSEGK	HexNAc(5)Hex(3)Fuc(1)	100	1.00E-17
Q9HD45	Transmembrane 9 superfamily member 3	IVDVNLTSEGK	HexNAc(5)Hex(3)	100	1.00E-17
Q9HD45	Transmembrane 9 superfamily member 3	IVDVNLTSEGK	HexNAc(3)Hex(6)Fuc(1)	6.708	1.96E-02
Q9HD45	Transmembrane 9 superfamily member 3	IVDVNLTSEGK	HexNAc(3)Hex(5)	100	1.00E-17
Q9HDC9	Adipocyte plasma membrane-associated protein	AGPNGTLFVADAYK	HexNAc(5)Hex(3)Fuc(1)	3.689	4.28E-02
Q9HDC9	Adipocyte plasma membrane-associated protein	NMSFVNDLTVTQDGRK	HexNAc(2)Hex(6)	4.396	6.84E-03
Q9HDC9	Adipocyte plasma membrane-associated protein	NMSFVNDLTVTQDGR	HexNAc(2)Hex(6)	4.224	2.41E-02
Q9HDC9	Adipocyte plasma membrane-associated protein	NMSFVNDLTVTQDGRK	HexNAc(2)Hex(5)	100	1.00E-17
Q9HDC9	Adipocyte plasma membrane-associated protein	NMSFVNDLTVTQDGR	HexNAc(2)Hex(5)	100	1.00E-17
Q9NPF0	CD320 antigen	NCSR	HexNAc(5)Hex(4)Fuc(2)NeuAc(1)	100	1.00E-17
Q9NPF0	CD320 antigen	NCSR	HexNAc(2)Hex(6)	100	1.00E-17
Q9NQC3	Reticulon-4	NTSTK	HexNAc(6)Hex(7)	100	1.00E-17
Q9NQZ3	Isoform 2 of Deleted in azoospermia protein 1	IITNR	HexNAc(4)Hex(4)	100	1.00E-17
Q9NR09	Baculoviral IAP repeat-containing protein 6	IANATR	HexNAc(4)Hex(4)Fuc(1)	100	1.00E-17

Q9NR09	Baculoviral IAP repeat-containing protein 6	IANATR	HexNAc(2)Hex(6)	100	1.00E-17
Q9NRN7	L-aminoadipate-semialdehyde dehydrogenase-phosphopantetheinyl transferase	NGTK	HexNAc(4)Hex(5)Fuc(3)NeuAc(1)	100	1.00E-17
Q9NT68	Teneurin-2	LGPYANTTK	HexNAc(5)Hex(4)Fuc(2)	100	1.00E-17
Q9NT68	Teneurin-2	FNISLGK	HexNAc(5)Hex(4)Fuc(1)	100	1.00E-17
Q9NT68	Teneurin-2	NSYQLCNNGTLR	HexNAc(2)Hex(9)	100	1.00E-17
Q9NT68	Teneurin-2	DSTHIIPGENPFNSSLVSLIR	HexNAc(2)Hex(7)	100	1.00E-17
Q9NT99	Leucine-rich repeat-containing protein 4B	ETVPSNTTCCAR	HexNAc(5)Hex(4)Fuc(2)	100	1.00E-17
Q9NT99	Leucine-rich repeat-containing protein 4B	TGTSMTSVNWLTPNGTLMTHGSYR	HexNAc(2)Hex(5)	100	1.00E-17
Q9NT99	Leucine-rich repeat-containing protein 4B	ETVPSNTTCCAR	HexNAc(2)Hex(5)	100	1.00E-17
Q9NTN9	Isoform 2 of Semaphorin-4G	NNQTECFNHVR	HexNAc(5)Hex(4)Fuc(2)	100	1.00E-17
Q9NTN9	Isoform 2 of Semaphorin-4G	NNQTECFNHVR	HexNAc(5)Hex(3)Fuc(1)	100	1.00E-17
Q9NTN9	Isoform 2 of Semaphorin-4G	GQAQNYSTLLLEEASAR	HexNAc(2)Hex(8)	3.179	3.50E-02
Q9NUM4	Transmembrane protein 106B	LNNITIIGPLDMK	HexNAc(2)Hex(5)	4.41	2.93E-02
Q9NUN5	Probable lysosomal cobalamin transporter	NQNGTFK	HexNAc(2)Hex(5)	4.134	3.79E-02
Q9NVI7	Isoform 2 of ATPase family AAA domain-containing protein 3A	NRSLYR	HexNAc(4)Hex(6)Fuc(1)	100	1.00E-17
Q9NX58	Cell growth-regulating nucleolar protein	PNVSPK	HexNAc(4)Hex(4)NeuAc(1)	100	1.00E-17
Q9NXF1	Testis-expressed protein 10	NITTLK	HexNAc(2)Hex(9)	100	1.00E-17
Q9NY47	Voltage-dependent calcium channel subunit alpha-2/delta-2	VLASNR*	HexNAc(5)Hex(3)Fuc(1)	100	1.00E-17
Q9NY47	Voltage-dependent calcium channel subunit alpha-2/delta-2	NYTWVPIR	HexNAc(2)Hex(7)	2.89	2.10E-02
Q9NY47	Voltage-dependent calcium channel subunit alpha-2/delta-2	NYTWVPIR	HexNAc(2)Hex(6)	2.419	3.21E-02
Q9NY47	Voltage-dependent calcium channel subunit alpha-2/delta-2	DLNASDNNTEFLK	HexNAc(2)Hex(5)	100	1.00E-17
Q9NY47	Voltage-dependent calcium channel subunit alpha-2/delta-2	VLASNR	HexNAc(2)Hex(5)	18.937	6.32E-04
Q9NY72	Sodium channel subunit beta-3	LQWNGSK	HexNAc(2)Hex(9)	100	1.00E-17
Q9NY72	Sodium channel subunit beta-3	LQWNGSK	HexNAc(2)Hex(8)	100	1.00E-17
Q9NY72	Sodium channel subunit beta-3	LQWNGSK	HexNAc(2)Hex(7)	100	1.00E-17
Q9NY72	Sodium channel subunit beta-3	LQWNGSK	HexNAc(2)Hex(6)	100	1.00E-17
Q9NY72	Sodium channel subunit beta-3	LQWNGSK	HexNAc(2)Hex(5)	100	1.00E-17
Q9NYQ7	Isoform 2 of Cadherin EGF LAG seven-pass G-type receptor 3	VDMAAFVANNGTMAGCQAK	HexNAc(9)Hex(4)	4.308	2.87E-02
Q9NYQ7	Isoform 2 of Cadherin EGF LAG seven-pass G-type receptor 3	LLQTANR	HexNAc(2)Hex(9)	3.107	4.70E-02
Q9NYQ7	Isoform 2 of Cadherin EGF LAG seven-pass G-type receptor 3	LLQTANR	HexNAc(2)Hex(8)	4.547	1.72E-02
Q9NYQ7	Isoform 2 of Cadherin EGF LAG seven-pass G-type receptor 3	LLQTANR	HexNAc(2)Hex(7)	100	1.00E-17
Q9NYQ7	Isoform 2 of Cadherin EGF LAG seven-pass G-type receptor 3	GFDPNCNK	HexNAc(2)Hex(6)	100	1.00E-17
Q9NYQ7	Isoform 2 of Cadherin EGF LAG seven-pass G-type receptor 3	GFDPNCNK	HexNAc(2)Hex(5)	100	1.00E-17
Q9NYV4	Cyclin-dependent kinase 12	TQEPAGSLEENNSDK	HexNAc(4)Hex(5)Fuc(1)NeuAc(1)	2.52	1.94E-02
Q9NZ53	Podocalyxin-like protein 2	SLEEIGIQNYSTTSSCQAR	HexNAc(5)Hex(4)NeuAc(1)	100	1.00E-17
Q9NZ53	Podocalyxin-like protein 2	SLEEIGIQNYSTTSSCQAR	HexNAc(5)Hex(4)Fuc(2)	100	1.00E-17
Q9NZN1	Interleukin-1 receptor accessory protein-like 1	EVREDDIGNYTCELK	HexNAc(2)Hex(7)	100	1.00E-17
Q9NZN1	Interleukin-1 receptor accessory protein-like 1	LTIQETQLGDSANLTCR	HexNAc(2)Hex(6)	100	1.00E-17
Q9NZN1	Interleukin-1 receptor accessory protein-like 1	LTIQETQLGDSANLTCR	HexNAc(2)Hex(5)	100	1.00E-17
Q9NZN1	Interleukin-1 receptor accessory protein-like 1	NSTYCMK	HexNAc(2)Hex(5)	12.139	1.01E-02
Q9NZR2	Low-density lipoprotein receptor-related protein 1B	NCNNTDCTHFYK	HexNAc(2)Hex(5)	100	1.00E-17
Q9NZR2	Low-density lipoprotein receptor-related protein 1B	THTCACPTNFYLAADNR	HexNAc(2)Hex(5)	100	1.00E-17
Q9P0K1	Isoform 3 of Disintegrin and metalloproteinase domain-containing protein 22	TLNCSGGHVK	HexNAc(2)Hex(5)	25.091	8.98E-04
Q9P0K1	Isoform 3 of Disintegrin and metalloproteinase domain-containing protein 22	ETCSGNSSQCAPNIHK	HexNAc(2)Hex(5)	10.238	3.82E-03
Q9P0L1	Zinc finger protein with KRAB and SCAN domains 7	AFNR	HexNAc(5)Hex(3)Fuc(1)	100	1.00E-17
Q9P244	Leucine-rich repeat and fibronectin type III domain-containing protein 1	LLGNSSR	HexNAc(2)Hex(7)	17.016	1.06E-03
Q9P244	Leucine-rich repeat and fibronectin type III domain-containing protein 1	LLGNSSR	HexNAc(2)Hex(6)	5.367	1.94E-02
Q9P244	Leucine-rich repeat and fibronectin type III domain-containing protein 1	LLGNSSR	HexNAc(2)Hex(5)	6.758	1.13E-02
Q9P273	Teneurin-3	IGPFANTTK	HexNAc(5)Hex(5)Fuc(3)	100	1.00E-17
Q9P273	Teneurin-3	FNISLQK	HexNAc(5)Hex(5)Fuc(2)	7.522	5.63E-03
Q9P273	Teneurin-3	IGPFANTTK	HexNAc(5)Hex(4)Fuc(2)	100	1.00E-17
Q9P273	Teneurin-3	FNISLQK	HexNAc(3)Hex(6)Fuc(1)	100	1.00E-17
Q9P273	Teneurin-3	LTNVTFPTGVVTNLHGDMDK	HexNAc(2)Hex(9)	3.888	2.72E-02
Q9P273	Teneurin-3	FNISLQK	HexNAc(2)Hex(5)	100	1.00E-17
Q9P273	Teneurin-3	IGPFANTTK	HexNAc(2)Hex(5)	2.553	4.39E-02
Q9P2B2	Prostaglandin F2 receptor negative regulator	NVSVAEGK	HexNAc(5)Hex(5)Fuc(1)	100	1.00E-17
Q9P2B2	Prostaglandin F2 receptor negative regulator	NVSVAEGK	HexNAc(5)Hex(4)Fuc(1)	100	1.00E-17
Q9P2B2	Prostaglandin F2 receptor negative regulator	NVSVAEGK	HexNAc(5)Hex(3)Fuc(1)	100	1.00E-17
Q9P2B2	Prostaglandin F2 receptor negative regulator	ELDLTCNITTDR	HexNAc(2)Hex(5)	100	1.00E-17
Q9P2C4	Transmembrane protein 181	GMNFTWK	HexNAc(2)Hex(9)	100	1.00E-17
Q9P2C4	Transmembrane protein 181	GMNFTWK	HexNAc(2)Hex(5)	100	1.00E-17
Q9P2K2	Thioredoxin domain-containing protein 16	HWNR	HexNAc(3)Hex(6)	100	1.00E-17
Q9UBG0	C-type mannose receptor 2	TSNISKPGTLER	HexNAc(5)Hex(5)Fuc(3)	100	1.00E-17
Q9UBG0	C-type mannose receptor 2	VTPACNTSLPAQR	HexNAc(5)Hex(5)Fuc(3)	100	1.00E-17
Q9UBG0	C-type mannose receptor 2	VTPACNTSLPAQR	HexNAc(5)Hex(5)Fuc(2)	100	1.00E-17

Q9UBG0	C-type mannose receptor 2	NCTSFR	HexNAc(5)Hex(4)Fuc(1)	100	1.00E-17
Q9UBG0	C-type mannose receptor 2	KKPNATAEPTPPDR	HexNAc(4)Hex(5)Fuc(3)NeuAc(2)	100	1.00E-17
Q9UBG0	C-type mannose receptor 2	NCTSFR	HexNAc(4)Hex(5)Fuc(3)	100	1.00E-17
Q9UBG0	C-type mannose receptor 2	TSNISKPGTLER	HexNAc(4)Hex(5)Fuc(1)NeuAc(2)	100	1.00E-17
Q9UBG0	C-type mannose receptor 2	NCTSFR	HexNAc(4)Hex(5)Fuc(1)	100	1.00E-17
Q9UBG0	C-type mannose receptor 2	VTPACNTSLPAQR	HexNAc(3)Hex(6)Fuc(1)NeuAc(1)	100	1.00E-17
Q9UBG0	C-type mannose receptor 2	VTPACNTSLPAQR	HexNAc(2)Hex(5)	8.293	3.15E-02
Q9UBS5	Isoform 1C of Gamma-aminobutyric acid type B receptor subunit 1	LEDFNYNNQTITDQIYR	HexNAc(2)Hex(9)	100	1.00E-17
Q9UBS5	Isoform 1C of Gamma-aminobutyric acid type B receptor subunit 1	SISNMTSQEFVEK	HexNAc(2)Hex(9)	7.557	8.92E-03
Q9UBS5	Isoform 1C of Gamma-aminobutyric acid type B receptor subunit 1	CLANGSWTDMDTPSR	HexNAc(2)Hex(8)	100	1.00E-17
Q9UBS5	Isoform 1C of Gamma-aminobutyric acid type B receptor subunit 1	SISNMTSQEFVEK	HexNAc(2)Hex(8)	3.821	2.55E-02
Q9UBS5	Isoform 1C of Gamma-aminobutyric acid type B receptor subunit 1	SISNMTSQEFVEK	HexNAc(2)Hex(7)	100	1.00E-17
Q9UBS5	Isoform 1C of Gamma-aminobutyric acid type B receptor subunit 1	LEDFNYNNQTITDQIYR	HexNAc(2)Hex(7)	4.242	2.44E-02
Q9UBS5	Isoform 1C of Gamma-aminobutyric acid type B receptor subunit 1	SISNMTSQEFVEK	HexNAc(2)Hex(6)	100	1.00E-17
Q9UBS5	Isoform 1C of Gamma-aminobutyric acid type B receptor subunit 1	LEDFNYNNQTITDQIYR	HexNAc(2)Hex(6)	22.032	1.69E-04
Q9UBV2	Protein sel-1 homolog 1	ILNGSNK	HexNAc(5)Hex(5)	100	1.00E-17
Q9UBV2	Protein sel-1 homolog 1	ILNGSNK	HexNAc(5)Hex(4)Fuc(2)	7.056	1.25E-02
Q9UBV2	Protein sel-1 homolog 1	EASIVGENETYPR	HexNAc(2)Hex(7)	100	1.00E-17
Q9UBV2	Protein sel-1 homolog 1	MYSEGSDIVPQSNETALHYFK	HexNAc(2)Hex(6)	100	1.00E-17
Q9UH99	Isoform 2 of SUN domain-containing protein 2	ALSPNSTISSAPK	HexNAc(2)Hex(8)	100	1.00E-17
Q9UH99	Isoform 2 of SUN domain-containing protein 2	ALSPNSTISSAPK	HexNAc(2)Hex(7)	100	1.00E-17
Q9UH99	Isoform 2 of SUN domain-containing protein 2	ALSPNSTISSAPK	HexNAc(2)Hex(6)	5.664	1.83E-02
Q9UH99	Isoform 2 of SUN domain-containing protein 2	ALSPNSTISSAPK	HexNAc(2)Hex(5)	100	1.00E-17
Q9UHC6	Contactin-associated protein-like 2	NMANGQPHSVNITR	HexNAc(2)Hex(8)	100	1.00E-17
Q9UHC6	Contactin-associated protein-like 2	VYCNMTEDK	HexNAc(2)Hex(8)	100	1.00E-17
Q9UHC6	Contactin-associated protein-like 2	SINLTLDR	HexNAc(2)Hex(8)	4.849	2.05E-02
Q9UHC6	Contactin-associated protein-like 2	SINLTLDR	HexNAc(2)Hex(7)	100	1.00E-17
Q9UHF7	Isoform 3 of Zinc finger transcription factor Trps1	NVTWR	HexNAc(5)Hex(7)Fuc(1)	100	1.00E-17
Q9UHG0	Doublecortin domain-containing protein 2	INVSAR	HexNAc(6)Hex(4)Fuc(2)	100	1.00E-17
Q9UHG0	Doublecortin domain-containing protein 2	INVSAR	HexNAc(5)Hex(5)Fuc(3)	100	1.00E-17
Q9UHG0	Doublecortin domain-containing protein 2	INVSAR	HexNAc(5)Hex(5)Fuc(2)	100	1.00E-17
Q9UHG0	Doublecortin domain-containing protein 2	INVSAR	HexNAc(5)Hex(4)Fuc(2)	37.576	2.69E-04
Q9UHG0	Doublecortin domain-containing protein 2	INVSAR	HexNAc(4)Hex(6)Fuc(2)	100	1.00E-17
Q9UHG0	Doublecortin domain-containing protein 2	INVSAR	HexNAc(4)Hex(6)Fuc(1)	100	1.00E-17
Q9UHG0	Doublecortin domain-containing protein 2	INVSAR	HexNAc(4)Hex(5)Fuc(2)	100	1.00E-17
Q9UHG0	Doublecortin domain-containing protein 2	INVSAR	HexNAc(4)Hex(5)Fuc(1)	100	1.00E-17
Q9UHG3	Prenylcysteine oxidase 1	LLHALGGDDFLGMLNR	HexNAc(2)Hex(9)	100	1.00E-17
Q9UHG3	Prenylcysteine oxidase 1	GELNTSIFSSRPIDK	HexNAc(2)Hex(8)	4.332	3.66E-03
Q9UHG3	Prenylcysteine oxidase 1	LLHALGGDDFLGMLNR	HexNAc(2)Hex(7)	100	1.00E-17
Q9UHG3	Prenylcysteine oxidase 1	GELNTSIFSSRPIDK	HexNAc(2)Hex(6)	2.992	1.65E-02
Q9UHG3	Prenylcysteine oxidase 1	GELNTSIFSSRPIDK	HexNAc(2)Hex(5)	100	1.00E-17
Q9UHN6	Cell surface hyaluronidase	TTNSSAADPR	HexNAc(5)Hex(3)Fuc(1)	100	1.00E-17
Q9UHN6	Cell surface hyaluronidase	NESR	HexNAc(4)Hex(6)Fuc(2)	100	1.00E-17
Q9UHN6	Cell surface hyaluronidase	TTNSSAADPR	HexNAc(4)Hex(4)Fuc(1)	100	1.00E-17
Q9UHN6	Cell surface hyaluronidase	HPSCVNVSK	HexNAc(2)Hex(7)	8.054	2.34E-02
Q9UHN6	Cell surface hyaluronidase	HPSCVNVSK	HexNAc(2)Hex(6)	5.57	2.14E-02
Q9UIQ6	Leucyl-cystinyl aminopeptidase	NQSIGLIQPFATNGK	HexNAc(2)Hex(6)	100	1.00E-17
Q9UIQ6	Leucyl-cystinyl aminopeptidase	SALLEFACTHNLGNCSTTAMK	HexNAc(2)Hex(5)	100	1.00E-17
Q9UIQ6	Leucyl-cystinyl aminopeptidase	NQSIGLIQPFATNGK	HexNAc(2)Hex(5)	100	1.00E-17
Q9UIW2	Plexin-A1	YNYTEDPTILR	HexNAc(5)Hex(4)Fuc(2)	14.69	2.10E-03
Q9UIW2	Plexin-A1	YNYTEDPTILR	HexNAc(5)Hex(3)Fuc(1)	100	1.00E-17
Q9UIW2	Plexin-A1	LSGNLTLLR*	HexNAc(5)Hex(3)Fuc(1)	17.044	1.74E-03
Q9UIW2	Plexin-A1	LSGNLTLLR	HexNAc(4)Hex(5)Fuc(2)	100	1.00E-17
Q9UIW2	Plexin-A1	LSGNLTLLR	HexNAc(4)Hex(5)Fuc(1)	3.216	3.06E-02
Q9UIW2	Plexin-A1	YNYTEDPTILR	HexNAc(4)Hex(5)	100	1.00E-17
Q9UIW2	Plexin-A1	LSGNLTLLR	HexNAc(4)Hex(4)Fuc(1)	100	1.00E-17
Q9UIW2	Plexin-A1	YNYTEDPTILR	HexNAc(4)Hex(4)	100	1.00E-17
Q9UIW2	Plexin-A1	ENGCLVYNDTTMVCR	HexNAc(2)Hex(7)	100	1.00E-17
Q9UIW2	Plexin-A1	ENGCLVYNDTTMVCR	HexNAc(2)Hex(6)	100	1.00E-17
Q9UIW2	Plexin-A1	YNYTEDPTILR	HexNAc(2)Hex(5)	100	1.00E-17
Q9UIW2	Plexin-A1	ENGCLVYNDTTMVCR	HexNAc(2)Hex(5)	100	1.00E-17
Q9UIW2	Plexin-A1	VNVSEDCPQILPSTQIYVPVGVVKPITLAAR	HexNAc(2)Hex(5)	12.653	9.59E-04
Q9UJ14	Glutathione hydrolase 7	NLSDSLAR	HexNAc(2)Hex(6)	100	1.00E-17
Q9UJ14	Glutathione hydrolase 7	NLSDSLAR	HexNAc(2)Hex(5)	5.2	1.39E-02
Q9UK53	Isoform 2 of Inhibitor of growth protein 1	NVSLMR	HexNAc(4)Hex(6)	38.755	7.42E-05
Q9UKM9	RNA-binding protein Raly	LQASNVTNKNDPK	HexNAc(3)Hex(3)Fuc(1)	4.149	3.44E-02
Q9UKV5	E3 ubiquitin-protein ligase AMFR	FLNK	HexNAc(4)Hex(5)Fuc(3)	5.351	2.32E-02
Q9UL58	Zinc finger protein 215	TNLTK	HexNAc(5)Hex(5)Fuc(1)NeuAc(1)	10.407	5.73E-03
Q9UL58	Zinc finger protein 215	TNLTK	HexNAc(5)Hex(4)Fuc(2)NeuAc(1)	8.823	6.46E-04
Q9ULB1	Isoform 3a of Neurexin-1	INCNSSK	HexNAc(5)Hex(4)Fuc(2)	100	1.00E-17
Q9ULB1	Isoform 3a of Neurexin-1	INCNSSK	HexNAc(5)Hex(3)Fuc(1)	25.894	1.92E-04

Q9ULB1	Isoform 3a of Neurexin-1	VNSSQVLPVDSGEVK	HexNAc(2)Hex(5)	4.014	3.07E-02
Q9ULJ8	Isoform 3 of Neurabin-1	LENWTPK	HexNAc(6)Hex(4)Fuc(1)	100	1.00E-17
Q9ULK0	Glutamate receptor ionotropic, delta-1	GLNGSLQER	HexNAc(4)Hex(5)NeuAc(1)	100	1.00E-17
Q9ULK0	Glutamate receptor ionotropic, delta-1	GLNGSLQER	HexNAc(2)Hex(5)	100	1.00E-17
Q9ULK0	Glutamate receptor ionotropic, delta-1	GLNGSLQERPMGSR	HexNAc(2)Hex(5)	100	1.00E-17
Q9UPQ9	Trinucleotide repeat-containing gene 6B protein	NTTPLPR	HexNAc(6)Hex(3)Fuc(1)NeuAc(1)	100	1.00E-17
Q9UPQ9	Trinucleotide repeat-containing gene 6B protein	SPAWNETGR	HexNAc(3)Hex(3)Fuc(1)	100	1.00E-17
Q9UPU3	VPS10 domain-containing receptor SorCS3	IVSNNCTDGLR	HexNAc(2)Hex(7)	100	1.00E-17
Q9UPU3	VPS10 domain-containing receptor SorCS3	IVSNNCTDGLR	HexNAc(2)Hex(6)	100	1.00E-17
Q9UPU3	VPS10 domain-containing receptor SorCS3	RIVSNNCTDGLR	HexNAc(2)Hex(5)	100	1.00E-17
Q9UPU3	VPS10 domain-containing receptor SorCS3	IVSNNCTDGLREK	HexNAc(2)Hex(5)	100	1.00E-17
Q9UPU3	VPS10 domain-containing receptor SorCS3	IVSNNCTDGLR	HexNAc(2)Hex(5)	100	1.00E-17
Q9UPZ6	Thrombospondin type-1 domain-containing protein 7A	YNAQPVGNWSDCILPEGK	HexNAc(6)Hex(5)Fuc(1)NeuAc(1)	100	1.00E-17
Q9UPZ6	Thrombospondin type-1 domain-containing protein 7A	YNAQPVGNWSDCILPEGK	HexNAc(5)Hex(5)Fuc(1)	4.202	3.84E-02
Q9UPZ6	Thrombospondin type-1 domain-containing protein 7A	YNAQPVGNWSDCILPEGK	HexNAc(5)Hex(4)NeuAc(1)	100	1.00E-17
Q9UPZ6	Thrombospondin type-1 domain-containing protein 7A	YNAQPVGNWSDCILPEGK	HexNAc(5)Hex(4)Fuc(2)	100	1.00E-17
Q9UPZ6	Thrombospondin type-1 domain-containing protein 7A	EVMCINK	HexNAc(5)Hex(4)Fuc(2)	100	1.00E-17
Q9UPZ6	Thrombospondin type-1 domain-containing protein 7A	CPNSSALQEVR	HexNAc(5)Hex(3)Fuc(1)	100	1.00E-17
Q9UPZ6	Thrombospondin type-1 domain-containing protein 7A	TRNISCVVSDGSADDFSK	HexNAc(2)Hex(8)	100	1.00E-17
Q9UPZ6	Thrombospondin type-1 domain-containing protein 7A	NISCVVSDGSADDFSK	HexNAc(2)Hex(8)	2.929	4.92E-02
Q9UPZ6	Thrombospondin type-1 domain-containing protein 7A	NISCVVSDGSADDFSK	HexNAc(2)Hex(7)	100	1.00E-17
Q9UPZ6	Thrombospondin type-1 domain-containing protein 7A	TRNISCVVSDGSADDFSK	HexNAc(2)Hex(7)	100	1.00E-17
Q9UPZ6	Thrombospondin type-1 domain-containing protein 7A	NISCVVSDGSADDFSK	HexNAc(2)Hex(6)	100	1.00E-17
Q9UPZ6	Thrombospondin type-1 domain-containing protein 7A	GNQTALCGGGIQTR	HexNAc(2)Hex(5)	100	1.00E-17
Q9UPZ6	Thrombospondin type-1 domain-containing protein 7A	SDGINVTGGCLVMSQPDADR	HexNAc(2)Hex(5)	7.696	1.35E-02
Q9UQ35	Serine/arginine repetitive matrix protein 2	TPPVALNSSR	HexNAc(3)Hex(3)Fuc(1)	9.275	3.99E-03
Q9Y2T2	AP-3 complex subunit mu-1	ENSSCGR	HexNAc(2)Hex(5)	100	1.00E-17
Q9Y3Q0	N-acetylated-alpha-linked acidic dipeptidase 2	NFSEAASDFHK	HexNAc(2)Hex(8)	100	1.00E-17
Q9Y4C0	Neurexin-3	INGSVR	HexNAc(5)Hex(4)Fuc(2)	12.85	8.71E-04
Q9Y4C0	Neurexin-3	INGSVR*	HexNAc(5)Hex(3)Fuc(1)	100	1.00E-17
Q9Y4C0	Neurexin-3	INGSVR	HexNAc(4)Hex(5)Fuc(1)	100	1.00E-17
Q9Y4C0	Neurexin-3	INGSVR	HexNAc(2)Hex(5)	100	1.00E-17
Q9Y4K0	Lysyl oxidase homolog 2	NVTCENGLPAVVSCVPGQVFSPDGPSR	HexNAc(2)Hex(8)	100	1.00E-17
Q9Y4L1	Hypoxia up-regulated protein 1	NATLAEQAK	HexNAc(2)Hex(6)	2.198	2.43E-02
Q9Y4L1	Hypoxia up-regulated protein 1	VINETWAWK	HexNAc(2)Hex(5)	8.778	1.41E-03
Q9Y4L1	Hypoxia up-regulated protein 1	VFGSQNLTTVK	HexNAc(2)Hex(5)	4.859	2.88E-03
Q9Y597	BTB/POZ domain-containing protein KCTD3	KINNTVR	HexNAc(2)Hex(7)	15.585	4.64E-03
Q9Y5B9	FACT complex subunit SPT16	SNVSYK	HexNAc(5)Hex(4)Fuc(2)	100	1.00E-17
Q9Y5F9	Protocadherin gamma-B6	DHGSPTLSANVSLR	HexNAc(5)Hex(3)Fuc(1)	4.287	6.51E-03
Q9Y639	Neuroplastin	ANATIEVK	HexNAc(7)Hex(3)	100	1.00E-17
Q9Y639	Neuroplastin	ANATIEVK	HexNAc(6)Hex(5)Fuc(2)	100	1.00E-17
Q9Y639	Neuroplastin	ANATIEVK	HexNAc(6)Hex(5)Fuc(1)NeuAc(1)	100	1.00E-17
Q9Y639	Neuroplastin	ANATIEVK	HexNAc(6)Hex(5)Fuc(1)	13.083	4.21E-03
Q9Y639	Neuroplastin	ANATIEVK	HexNAc(6)Hex(4)Fuc(1)	100	1.00E-17
Q9Y639	Neuroplastin	ANATIEVK	HexNAc(6)Hex(3)	100	1.00E-17
Q9Y639	Neuroplastin	ANATIEVK	HexNAc(5)Hex(6)Fuc(3)	100	1.00E-17
Q9Y639	Neuroplastin	ANATIEVK	HexNAc(5)Hex(5)Fuc(2)	100	1.00E-17
Q9Y639	Neuroplastin	NASNMEYR	HexNAc(5)Hex(4)Fuc(2)	100	1.00E-17
Q9Y639	Neuroplastin	ANATIEVK	HexNAc(5)Hex(4)Fuc(1)	100	1.00E-17
Q9Y639	Neuroplastin	NASNMEYR	HexNAc(5)Hex(3)Fuc(1)	100	1.00E-17
Q9Y639	Neuroplastin	NASNMEYR	HexNAc(4)Hex(5)Fuc(2)	9.026	6.18E-03
Q9Y639	Neuroplastin	NASNMEYR	HexNAc(4)Hex(5)Fuc(1)	13.402	3.93E-03
Q9Y639	Neuroplastin	NASNMEYR	HexNAc(4)Hex(5)	100	1.00E-17
Q9Y639	Neuroplastin	ANATIEVK	HexNAc(4)Hex(5)	100	1.00E-17
Q9Y639	Neuroplastin	NASNMEYR	HexNAc(3)Hex(6)Fuc(1)NeuAc(1)	100	1.00E-17
Q9Y639	Neuroplastin	ANATIEVK	HexNAc(2)Hex(5)	100	1.00E-17
Q9Y639	Neuroplastin	NASNMEYR	HexNAc(2)Hex(5)	12.682	5.16E-03
Q9Y666	Solute carrier family 12 member 7	SAFDPPDIPVCLLGNR	HexNAc(2)Hex(8)	100	1.00E-17
Q9Y6G9	Cytoplasmic dynein 1 light intermediate chain 1	VPGGSPRTPNR	HexNAc(3)Hex(5)	100	1.00E-17
Q9Y6M1	Insulin-like growth factor 2 mRNA-binding protein 2	NLTK	HexNAc(5)Hex(6)Fuc(2)	100	1.00E-17
Q9Y6M7	Isoform 7 of Sodium bicarbonate cotransporter 3	NLTVSECK	HexNAc(5)Hex(7)Fuc(1)	100	1.00E-17
Q9Y6M7	Isoform 7 of Sodium bicarbonate cotransporter 3	NLTVSECK	HexNAc(3)Hex(6)Fuc(1)NeuAc(1)	100	1.00E-17
Q9Y6M7	Isoform 7 of Sodium bicarbonate cotransporter 3	NLTVSECK	HexNAc(3)Hex(4)Fuc(1)NeuAc(1)	100	1.00E-17
Q9Y6M7	Isoform 7 of Sodium bicarbonate cotransporter 3	NLTVSECKK	HexNAc(2)Hex(5)	100	1.00E-17
Q9Y6N7	Isoform 2 of Roundabout homolog 1	YHINK	HexNAc(6)Hex(4)Fuc(2)	100	1.00E-17
Q9Y6N7	Isoform 2 of Roundabout homolog 1	YHINK	HexNAc(6)Hex(3)Fuc(1)	100	1.00E-17
Q9Y6N7	Isoform 2 of Roundabout homolog 1	YHINK	HexNAc(5)Hex(4)NeuAc(1)	6.439	1.80E-02
Q9Y6N7	Isoform 2 of Roundabout homolog 1	YHINK	HexNAc(4)Hex(5)Fuc(2)	4.432	2.77E-02

Q9Y6N7	Isoform 2 of Roundabout homolog 1	YHINK	HexNAc(4)Hex(5)Fuc(1)	8.016	1.21E-02
Q9Y6N7	Isoform 2 of Roundabout homolog 1	YHINK	HexNAc(4)Hex(4)Fuc(1)	100	1.00E-17
Q9Y6N7	Isoform 2 of Roundabout homolog 1	YHINK	HexNAc(2)Hex(5)	100	1.00E-17
Q9Y6N7	Isoform 2 of Roundabout homolog 1	YHINK	HexNAc(2)Hex(3)	100	1.00E-17
Q9Y6Q9	Nuclear receptor coactivator 3	NISAFPMLPK	HexNAc(3)Hex(4)Fuc(2)	100	1.00E-17
Q9Y6X0	SET-binding protein	KMCNYTK	HexNAc(2)Hex(7)	100	1.00E-17

* Fucosylated and bisected biantennary glycan-modified protein, which is deduced from the presence of diagnostic Y ion in the product ion spectrum.

Supplementary Table S4. (a) Significantly enriched terms in GO enrichment analysis of the HN5H3F1-modified proteins.

Term (Top10)	Count	-log10(p-value)	Gene Name (* bisected GlcNAc product spectrum detected)
cell adhesion	19	11.8	ATP1B1/L1CAM/THY1/ALCAM/CNTN1*/CNTN2*/ITGAV/LAMB4/ NEO1/NCAM1/NCAM2*/NLGN4X/OPCML/PTK7/PTPRF/PTPRS/ PCDH17/SEMA4D/SIRPA
homophilic cell adhesion via plasma membrane adhesion molecules	10	7.4	FAT3/FAT4/CELSR2/NPTN/PLXNB2/PCDH17/ PCDH19/PCDH9/PCDHGB6/SDK1
axon guidance	9	6.2	L1CAM/CNTN2*/NEO1/NCAM1/NRXN1/ NRXN3/NFASC*/SEMA6A/SLIT1
semaphorin-plexin signaling pathway	8	10.0	PLXNA3*/PLXNB1/PLXNB2/SEMA4C*/ SEMA4D/SEMA4G/SEMA6A/SEMA7A
negative chemotaxis	8	9.9	ITGAV/PLXNA3*/SEMA4C*/SEMA4D/ SEMA4G/SEMA6A/SEMA7A/SLIT1
negative regulation of axon extension involved in axon guidance	6	7.0	ADGRL3/DNER/NRXN1/NRXN3*/NRCAM/SDK1
synapse assembly	6	5.1	ADGRL3/DNER/NRXN1/NRXN3*/NRCAM/SDK1
neuron migration	6	4.0	MDGA1/ADGRL3/CNTN2/DNER/NRCAM/SEMA6A
leukocyte migration	6	3.6	ATP1B1/ATP1B3/L1CAM/CXADR/ITGAV/SIRPA
neural crest cell migration	5	4.3	SEMA4C*/SEMA4D/SEMA4G/SEMA6A/SEMA7A

Supplementarys Table S4. (b) Significantly enriched terms in GO enrichment analysis of the HN5H3F1-unmodified proteins.

Term (Top10)	Count	-log10(p-value)	Gene Name
cell adhesion	35	10.9	ADAM22/ADAM9/APC/ATP1B2/CD47/FAT1/BCAM/CDH2/CHST10 /CTNNA1/CHL1/COL6A1/CNTN5/CNTNAP4/CNTNAP5/CNTNAP2/ DSC2/EFNB1/EFNB2/LGALS3BP/ITGA3/ITGA5/ITGA7/ITGAD/ITGB5/ LOXL2/MCAM/MMRN1/MPDZ/MYH10/NECTIN1/PLXNC1/ROBO1/ ROBO2/TOR1A
axon guidance	21	10.6	DCC/EPHB2/EPHB3/CHL1/EFNA3/EFNB1/EFNB2/GPC1/LGI1/ MYH10/NECTIN1/NTN1/NRP1/PTPRO/ROBO1/ROBO2/SEMA3C/ SLIT2/SEMA4F/TENM2/TGFB2
homophilic cell adhesion via plasma membrane adhesion molecules	16	6.5	CD200/FAT1/CDH2/CELSR3/CADM1/CADM2/CADM3/CADM4/DSC2/ ITGB1/NECTIN1/PCDH7/ROBO1/ROBO2/SDK2/TENM3
extracellular matrix organization	16	5.3	CD47/BSG/COL3A1/COL6A1/ERO1A/FBN2/HSPG2/ITGA1/ITGA3/ ITGA5/ITGA7/ITGAD/ITGB1/ITGB5/JAM3/KDR
ion transmembrane transport	16	4.9	ATP6V0E2/ATP1B2/ANO5/ANO6/ASPH/GABRA1/GABRA3/GABRA5/ GABRB3/GABRG2/GRIN2B/GRID1/GRIK2/GRIK3/GLRA2/TTYH3
positive regulation of cell proliferation	15	1.2	ADAM10/CD47/TTK/UFL1/BIRC6/EFNB2/ERBB4/IGF1R/ITGB1/KDR/ NTN1/NTRK2/ NTRK3/NACC1/TGFB2
peptidyl-tyrosine phosphorylation	13	4.4	EPHB2/EPHB3/ERLIN2/MET/NEK1/TTK/WEE1/ERBB2/ERBB4/KDR /NTRK2/NTRK3/ROR2
positive regulation of cell migration	13	3.7	ADAM10/APC/SUN2/WNT5B/IGF1R/ITGA5/KDR/MCAM/NTRK3/ ROR2/SEMA3C/SPAG9/SEMA4F
transport	13	1.5	ABCA2/ATP1B2/DYNC1LI1/GABRA1/GABRA3/GABRA5/GABRB3/ GABRG2/GRIN2B/GRIK2/MAP6/P2RX3/SLC12A7
heterophilic cell-cell adhesion via plasma membrane cell adhesion molecules	12	8.7	CD200/CDH2/CADM1/CADM2/CADM3/CADM4/ITGA5/IL1RAPL1/ NECTIN1/TENM2/TENM3/TENM4

a

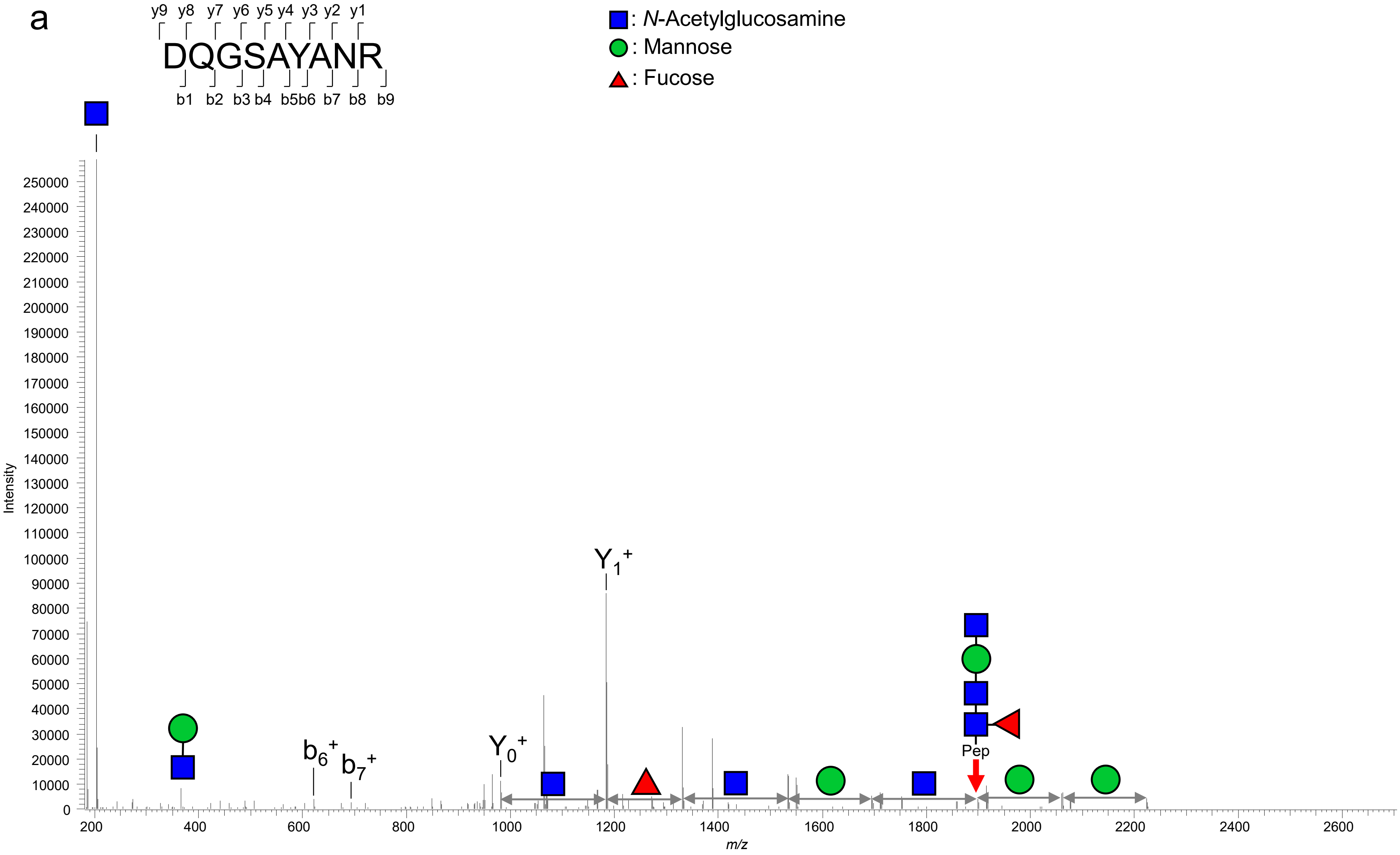

Supplementary Figure S1. (a) Product ion spectrum of CD276 antigen glycopeptide arising from precursor ion at *m/z*877.0223 (charge: 3)

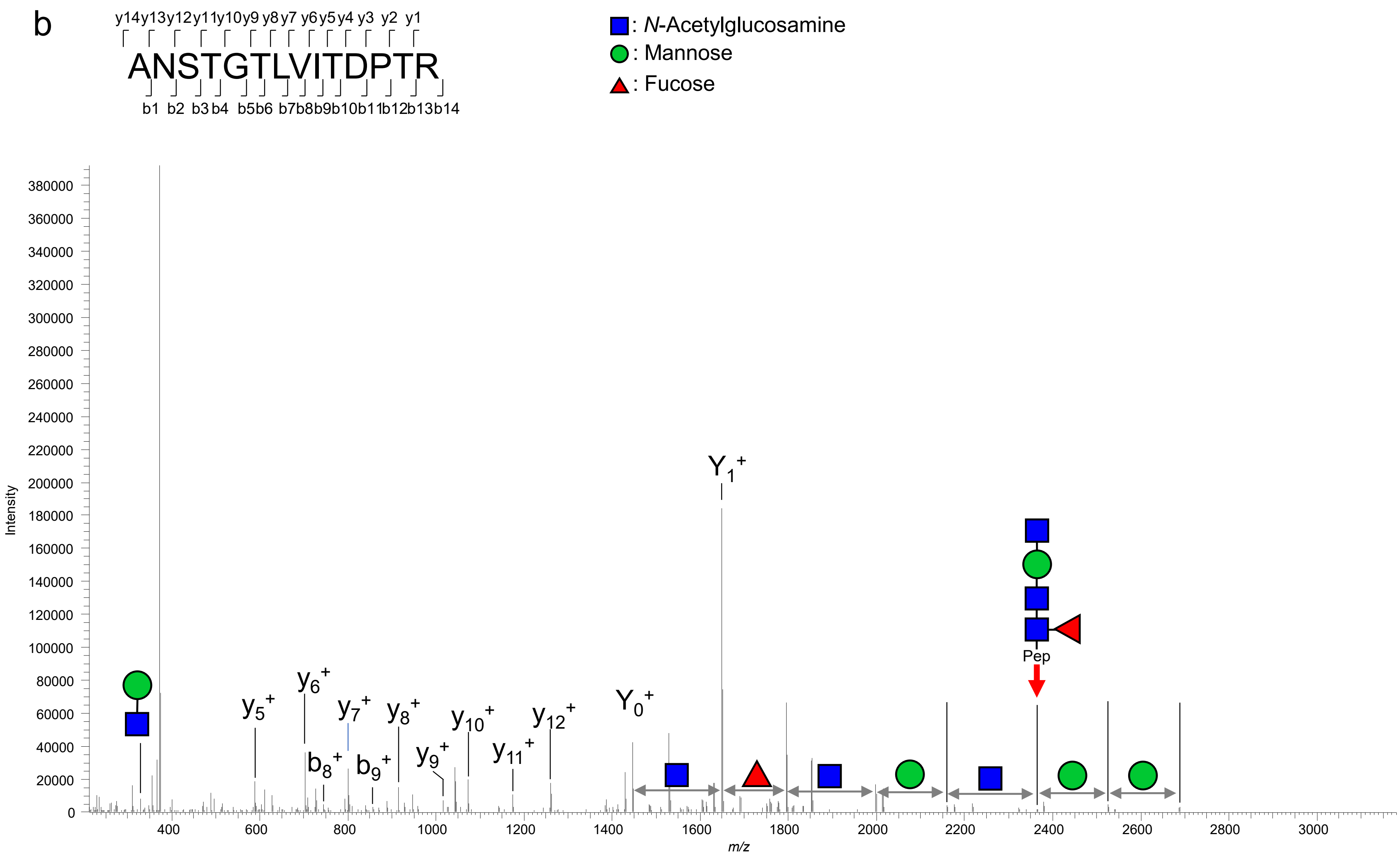

Supplementary Figure S1. (b) Product ion spectrum of contactin 1 glycopeptide arising from precursor ion at *m/z*1031.7959 (charge: 3)

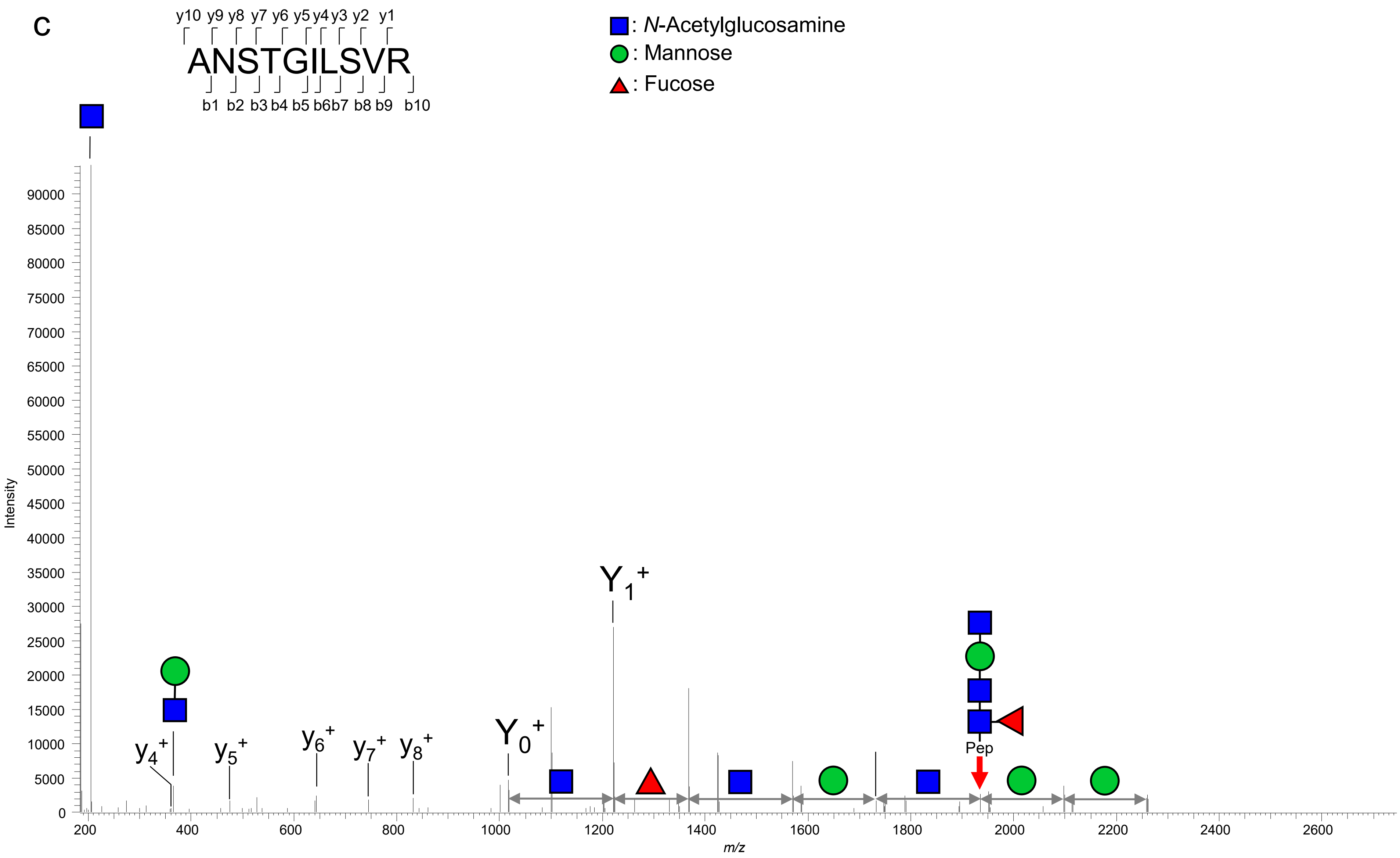

Supplementary Figure S1. (c) Product ion spectrum of contactin 2 glycopeptide arising from precursor ion at *m/z*889.0653 (charge: 3)

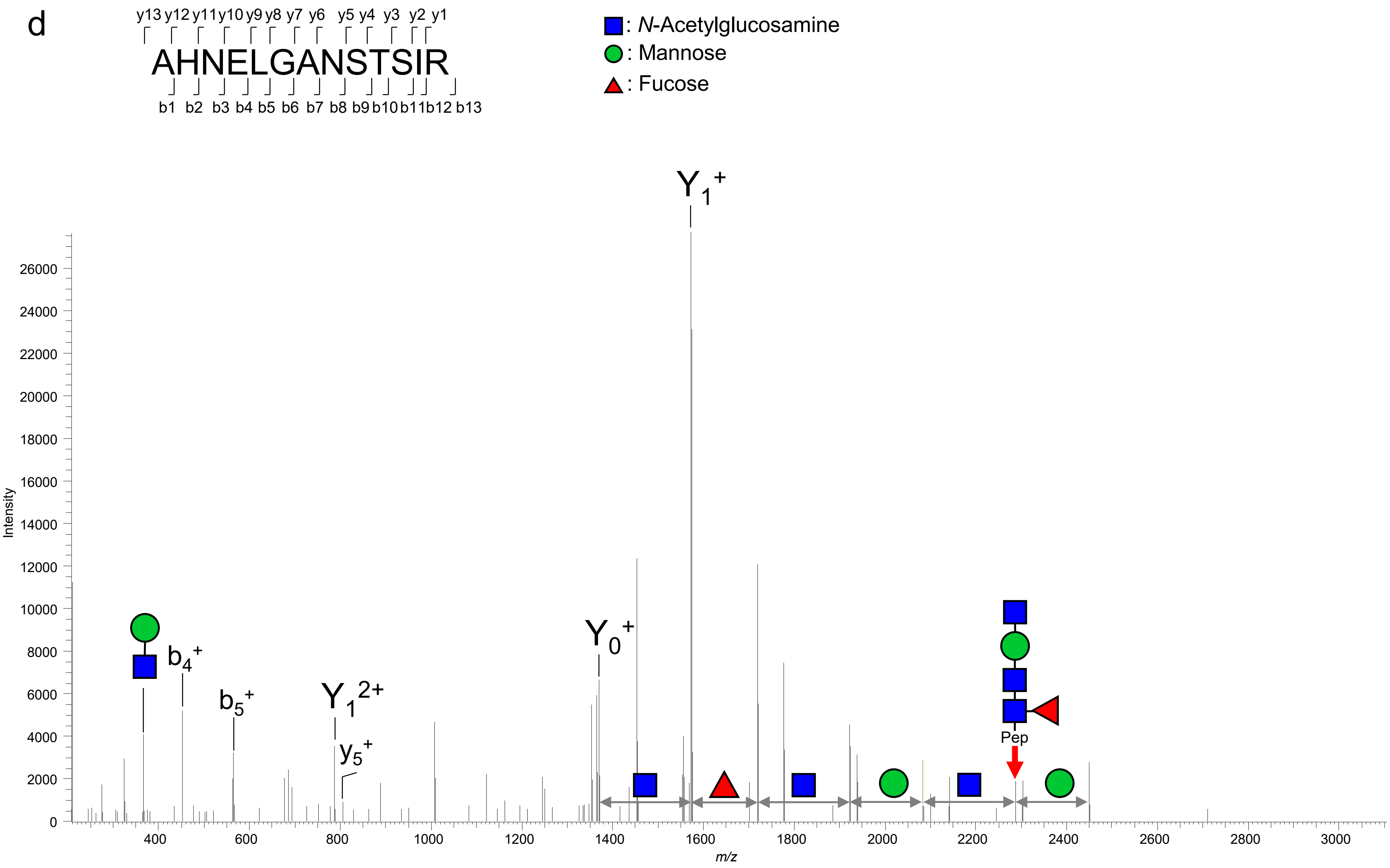

Supplementary Figure S1. (d) Product ion spectrum of Immunoglobulin superfamily containing leucine-rich repeat protein2 glycopeptide arising from precursor ion at *m/z*1006.4357 (charge: 3)

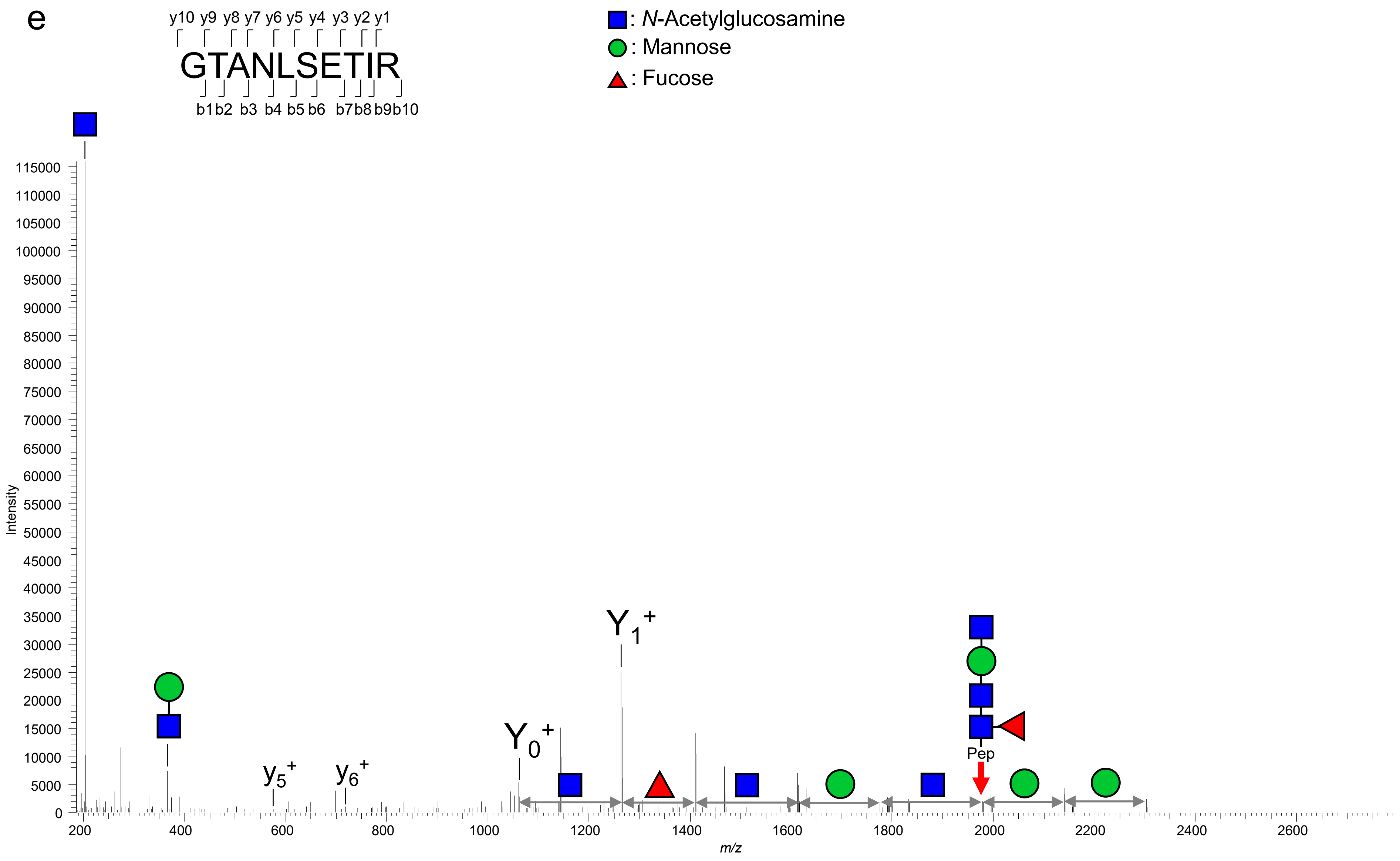

Supplementary Figure S1. (e) Product ion spectrum of tyrosine-protein phosphatase non-receptor type substrate 1 glycopeptide arising from precursor ion at *m/z*903.0242 (charge: 3)

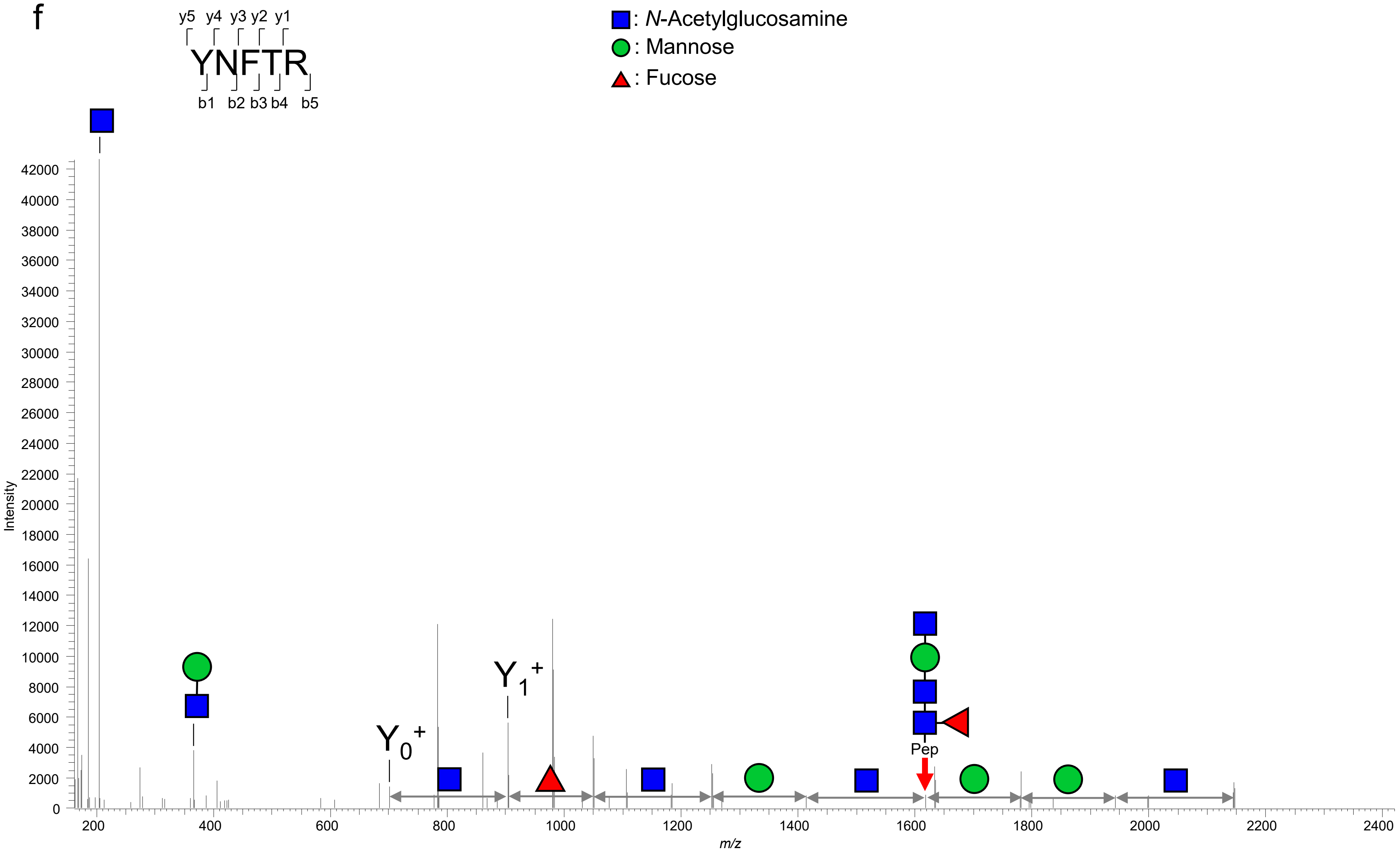

Supplementary Figure S1. (f) Product ion spectrum of heparan-sulfate 6-*o*-sulfotransferase 2 glycopeptide arising from precursor ion at *m/z*1174.4841 (charge: 3)

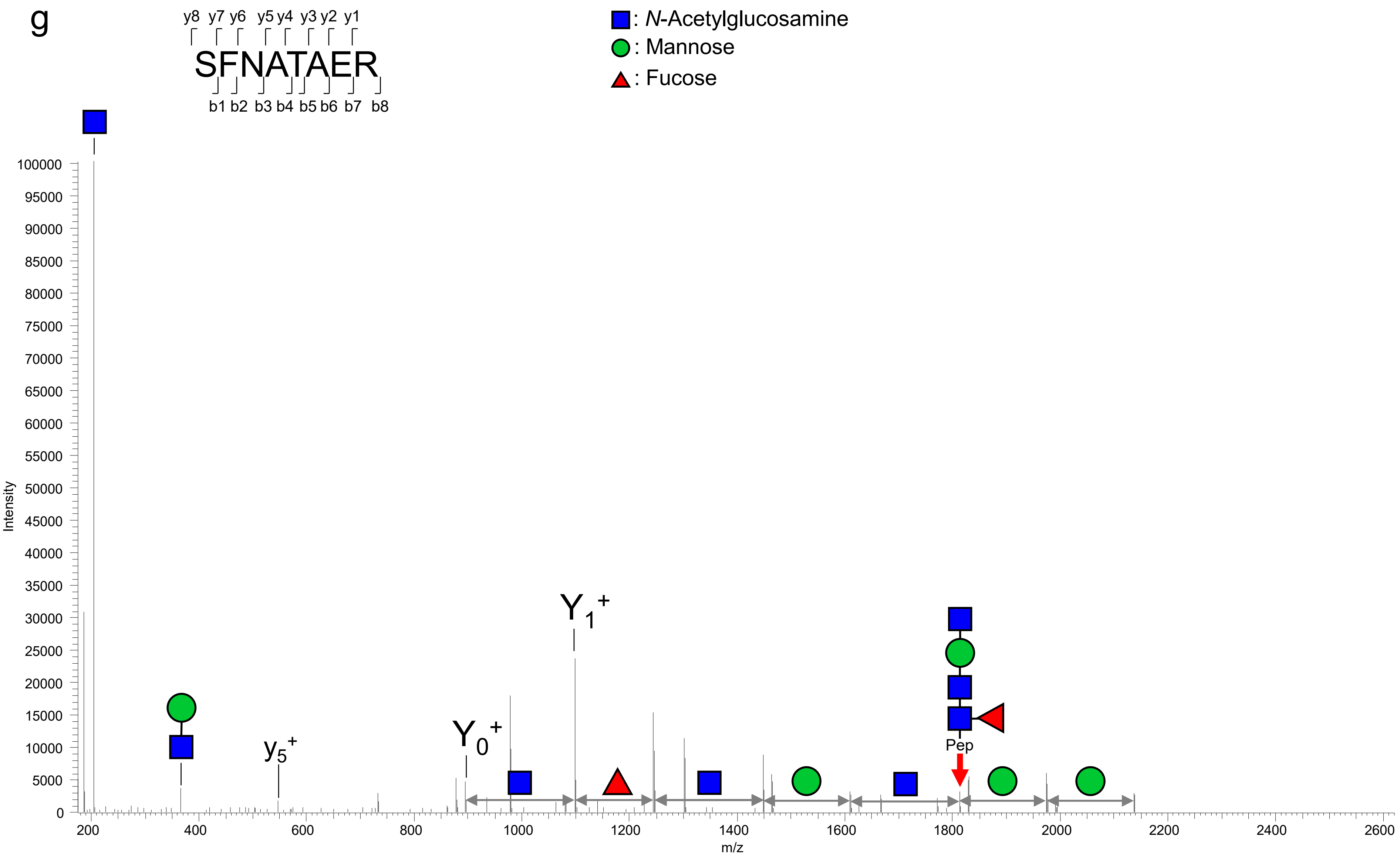

Supplementary Figure S1. (g) Product ion spectrum of neural cell adhesion 2 glycopeptide arising from precursor ion at *m/z*848.3494 (charge: 3)

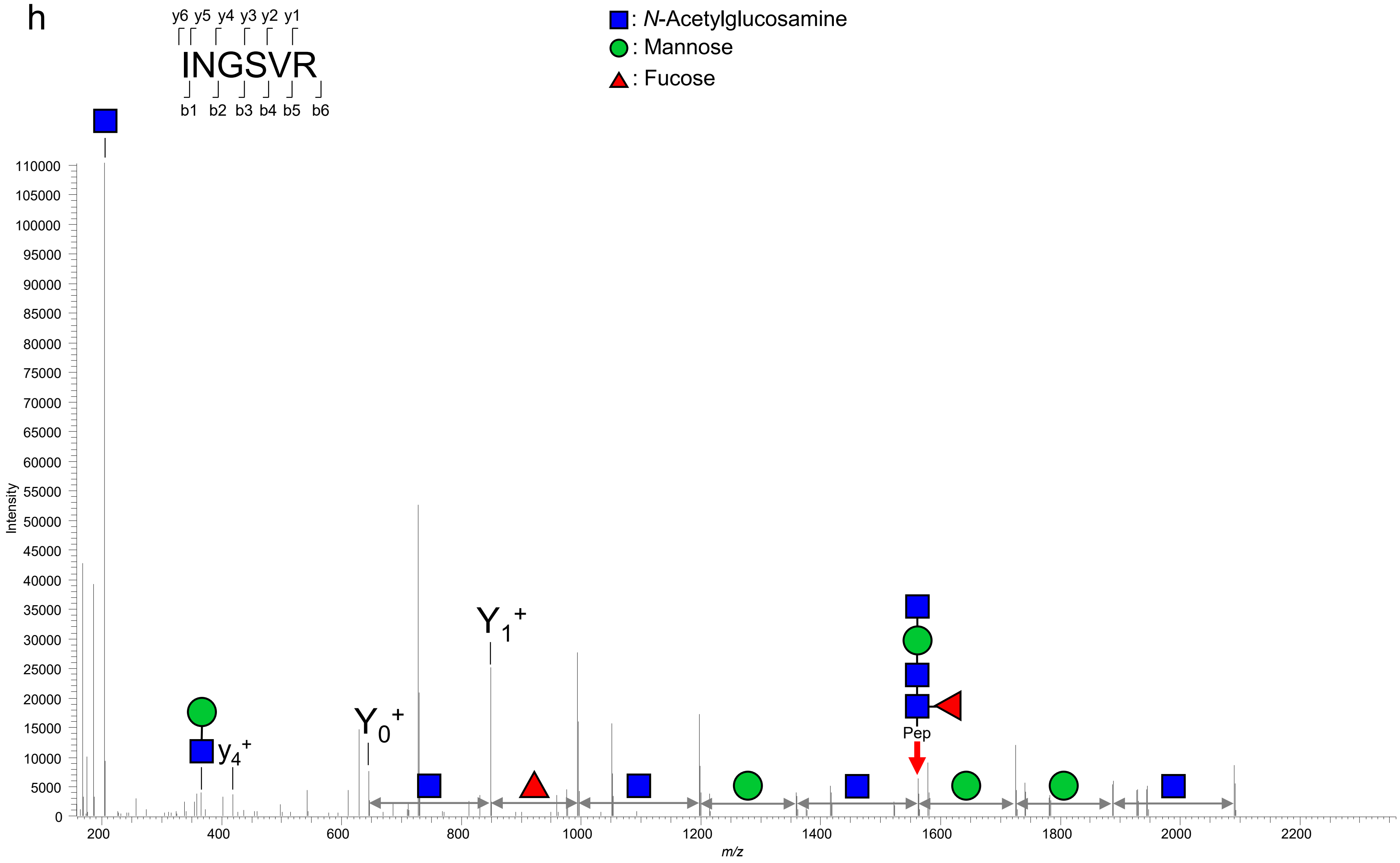

Supplementary Figure S1. (h) Product ion spectrum of neurexin 3 glycopeptide arising from precursor ion at *m/z*1146.9940 (charge: 2)

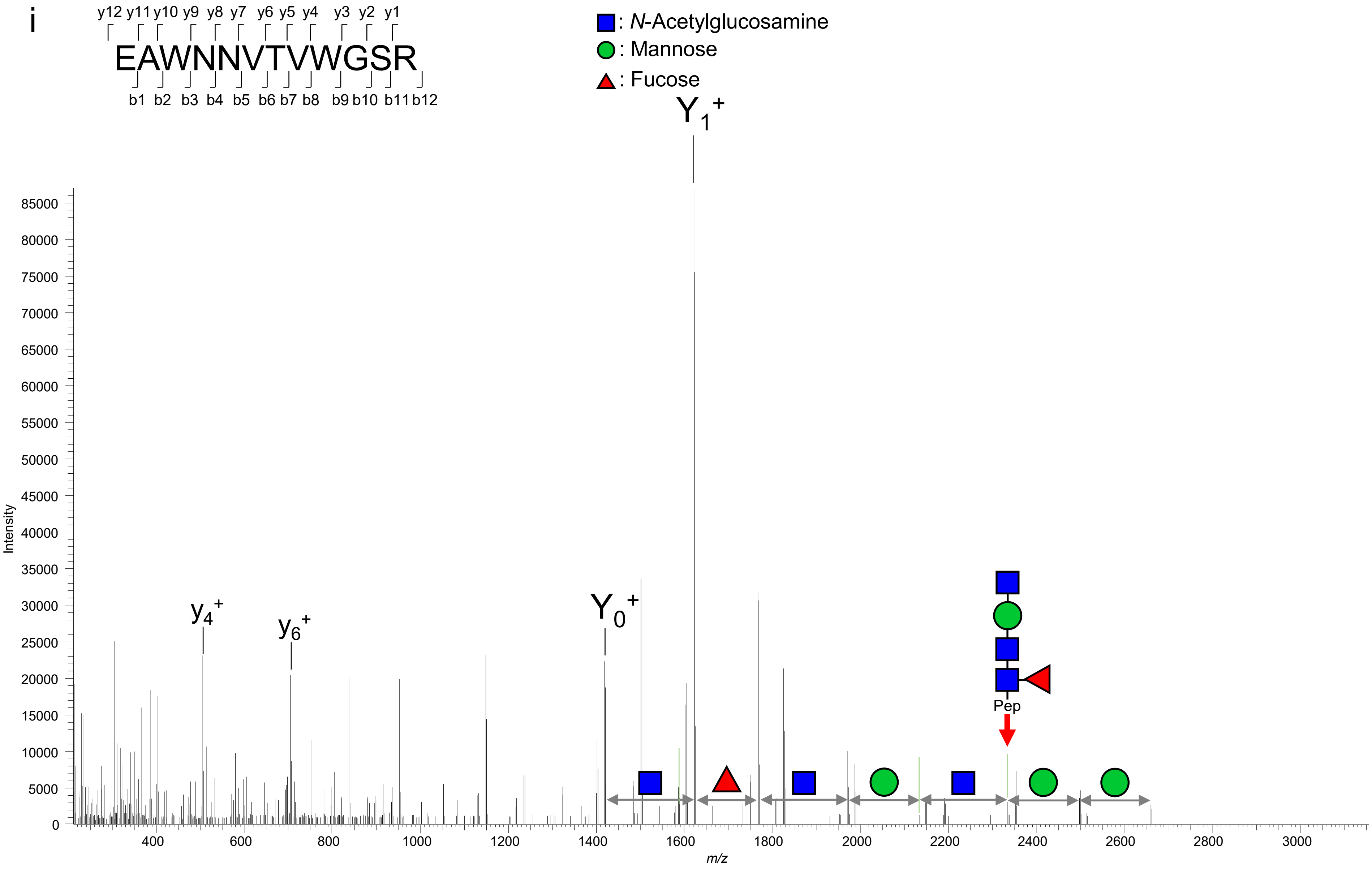

Supplementary Figure S1. (i) Product ion spectrum of neurofascin glycopeptide arising from precursor ion at *m/z*1022.7697 (charge: 2)

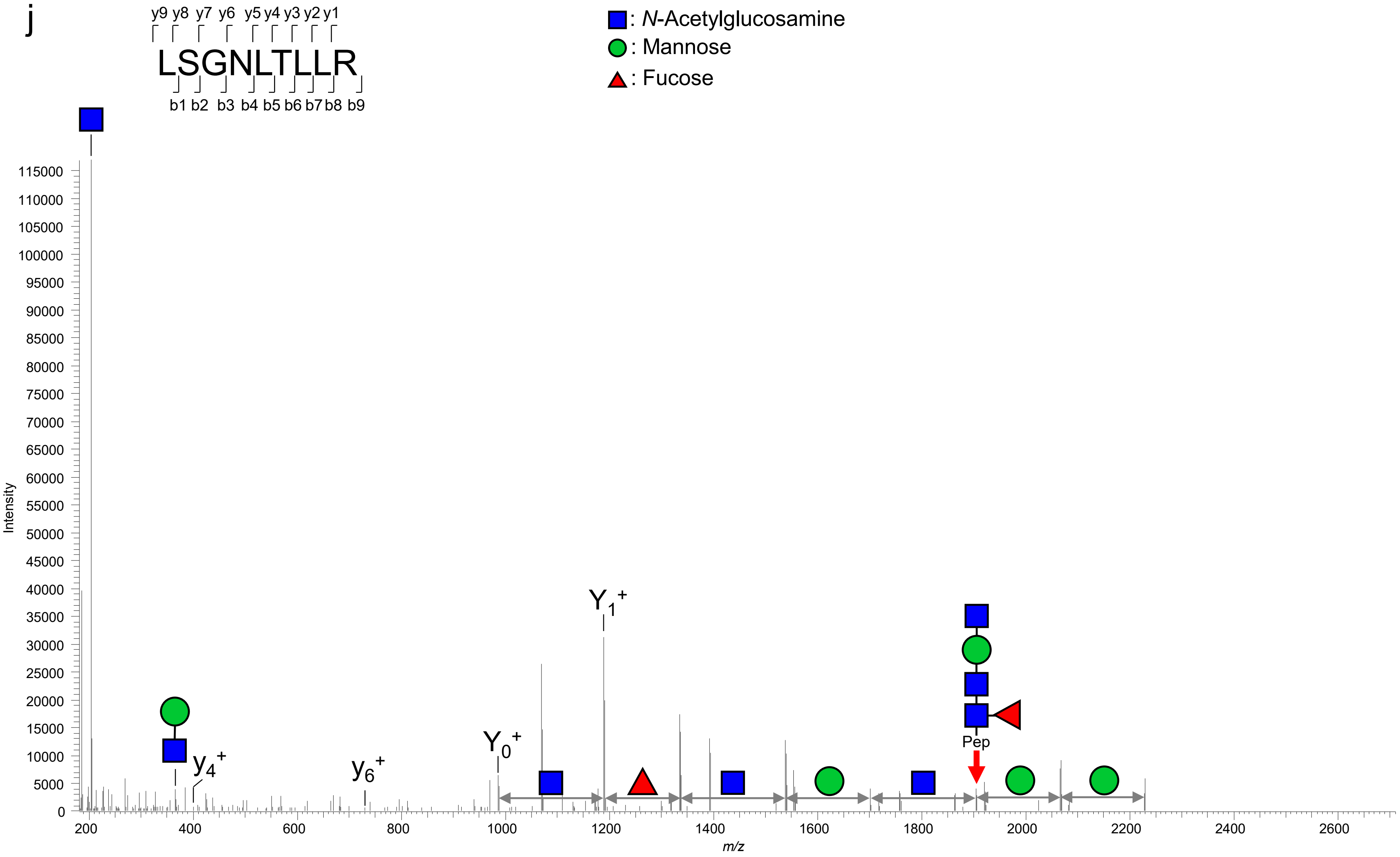

Supplementary Figure S1. (j) Product ion spectrum of plexin A1 glycopeptide arising from precursor ion at *m/z*878.7424 (charge: 3)

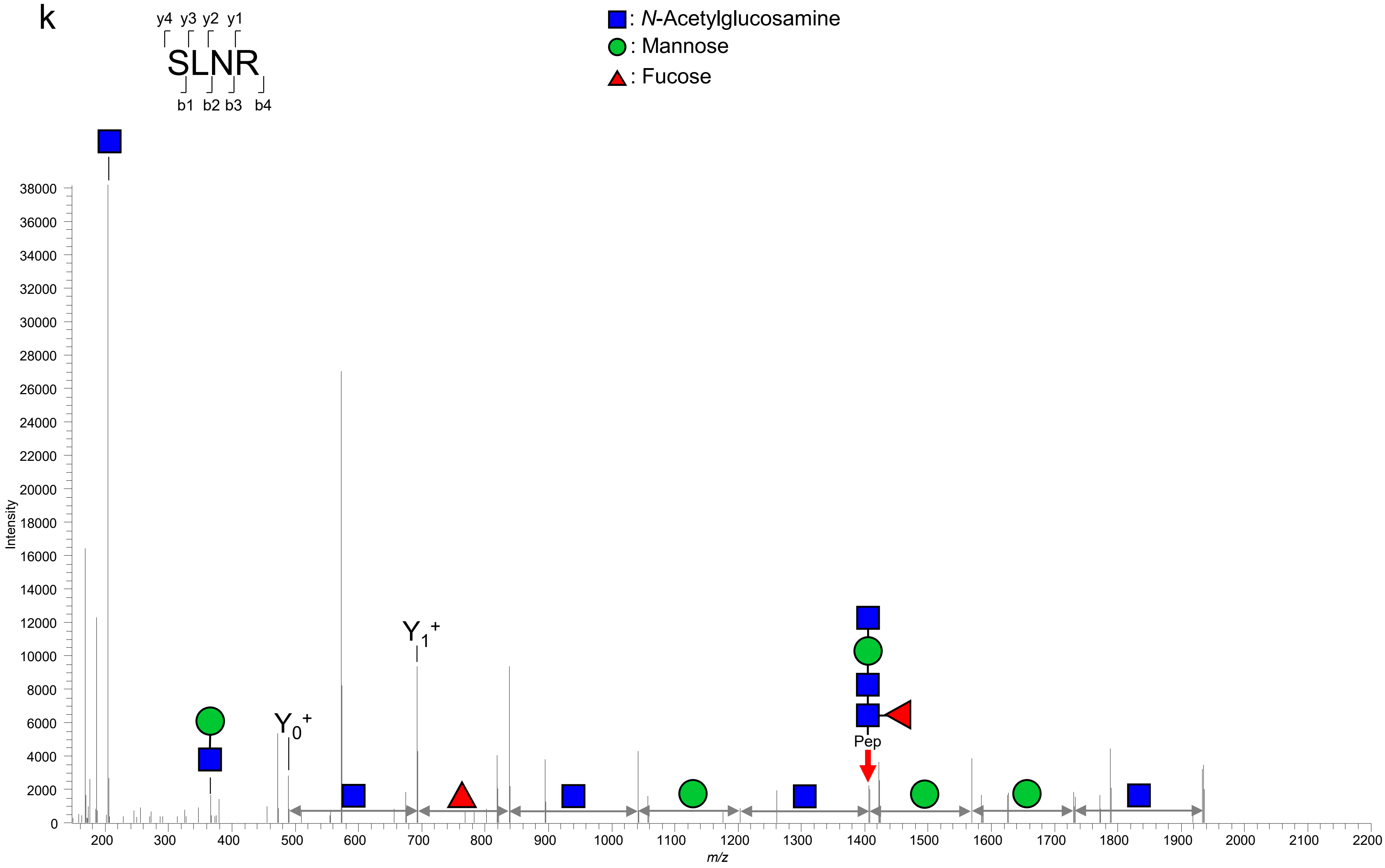

Supplementary Figure S1. (k) Product ion spectrum of plexin A3 glycopeptide arising from precursor ion at *m/z*1068.9490 (charge: 2)

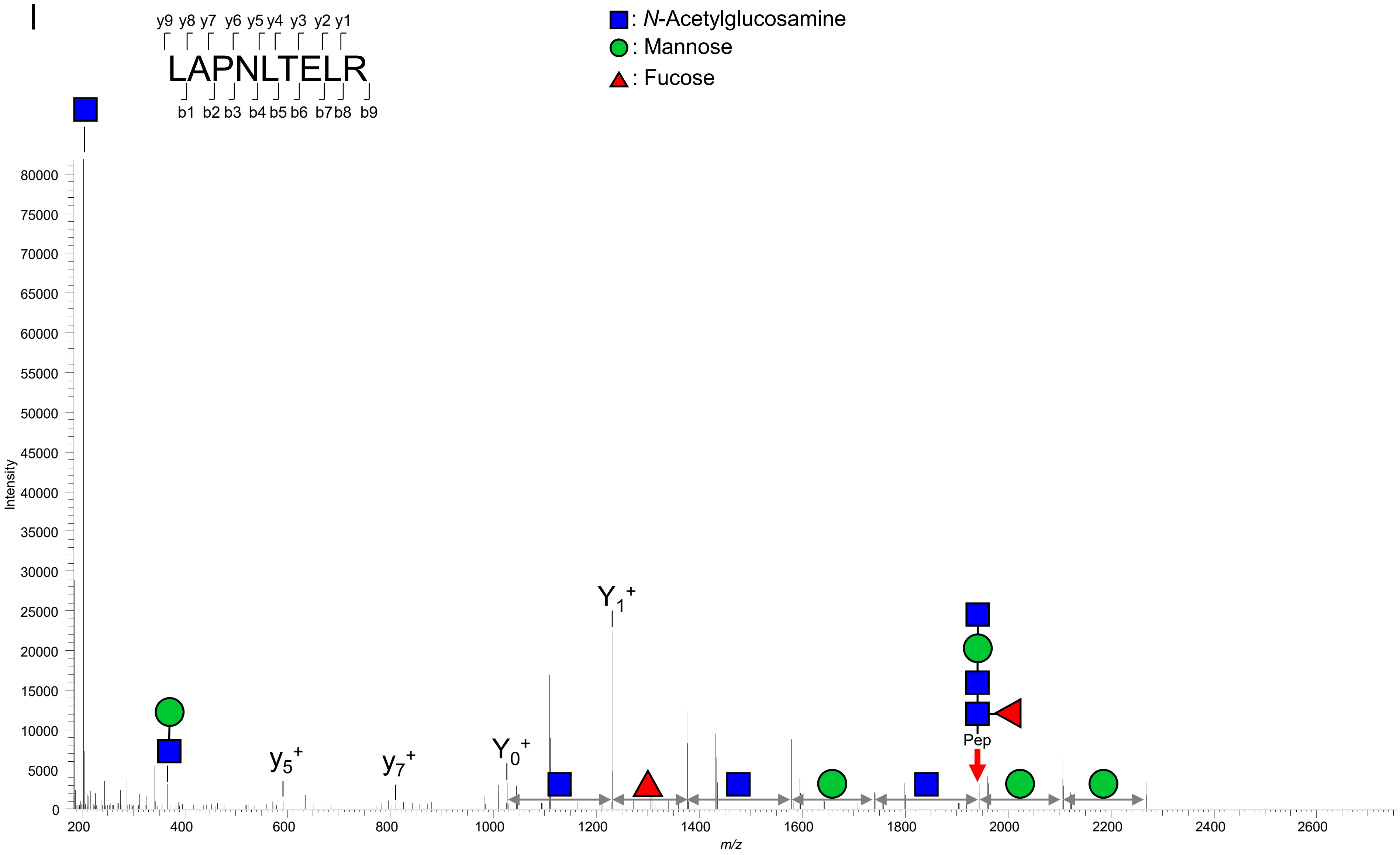

Supplementary Figure S1. (I) Product ion spectrum of plexin A3 glycopeptide arising from precursor ion at *m/z*892.0740 (charge: 2)

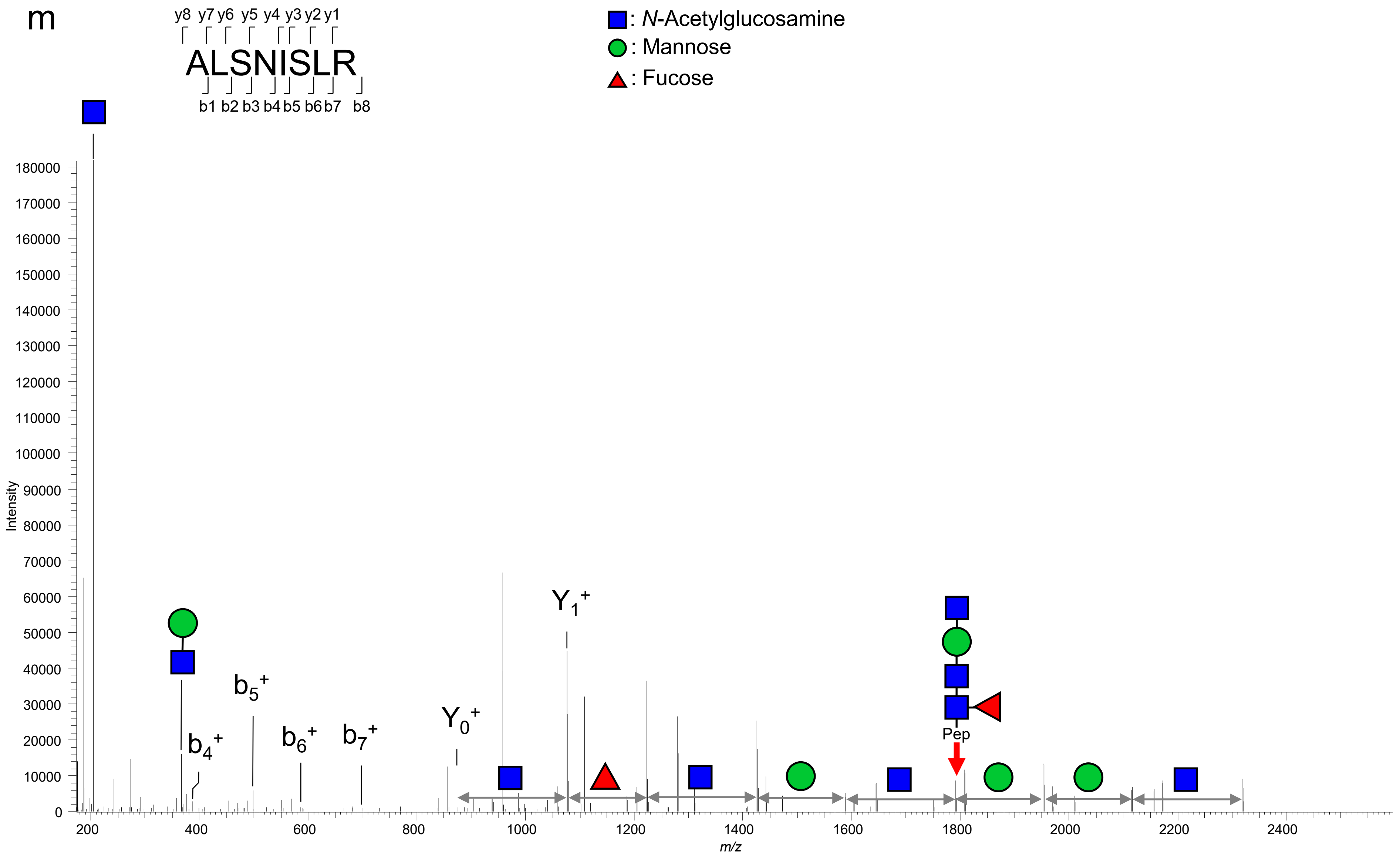

Supplementary Figure S1. (m) Product ion spectrum of plexin B2 glycopeptide arising from precursor ion at *m/z*1261.0690 (charge: 2)

n

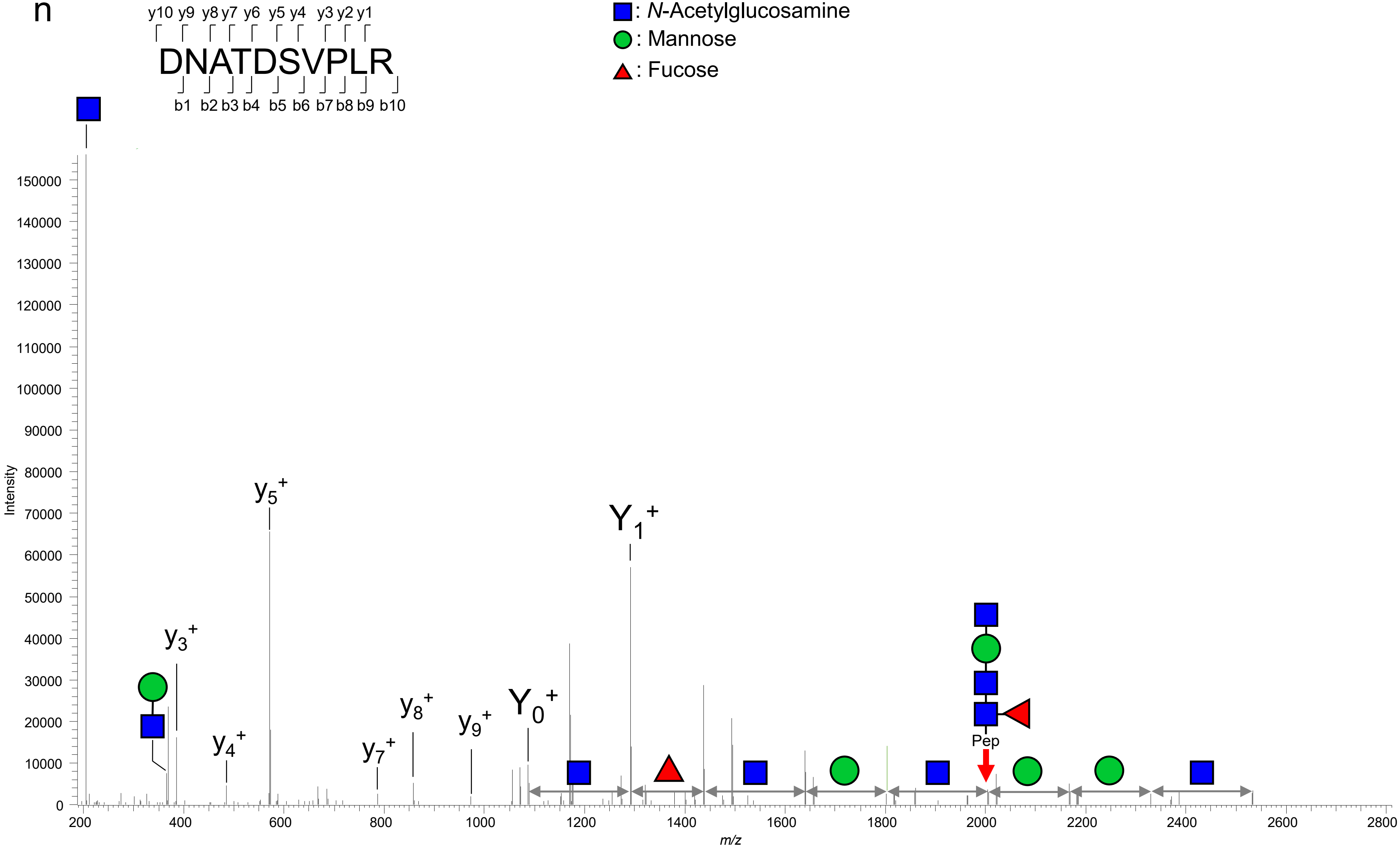

Supplementary Figure S1. (n) Product ion spectrum of prolow-density lipoprotein receptor-related protein 1 glycopeptide arising from precursor ion at *m/z*1368.0802 (charge: 2)

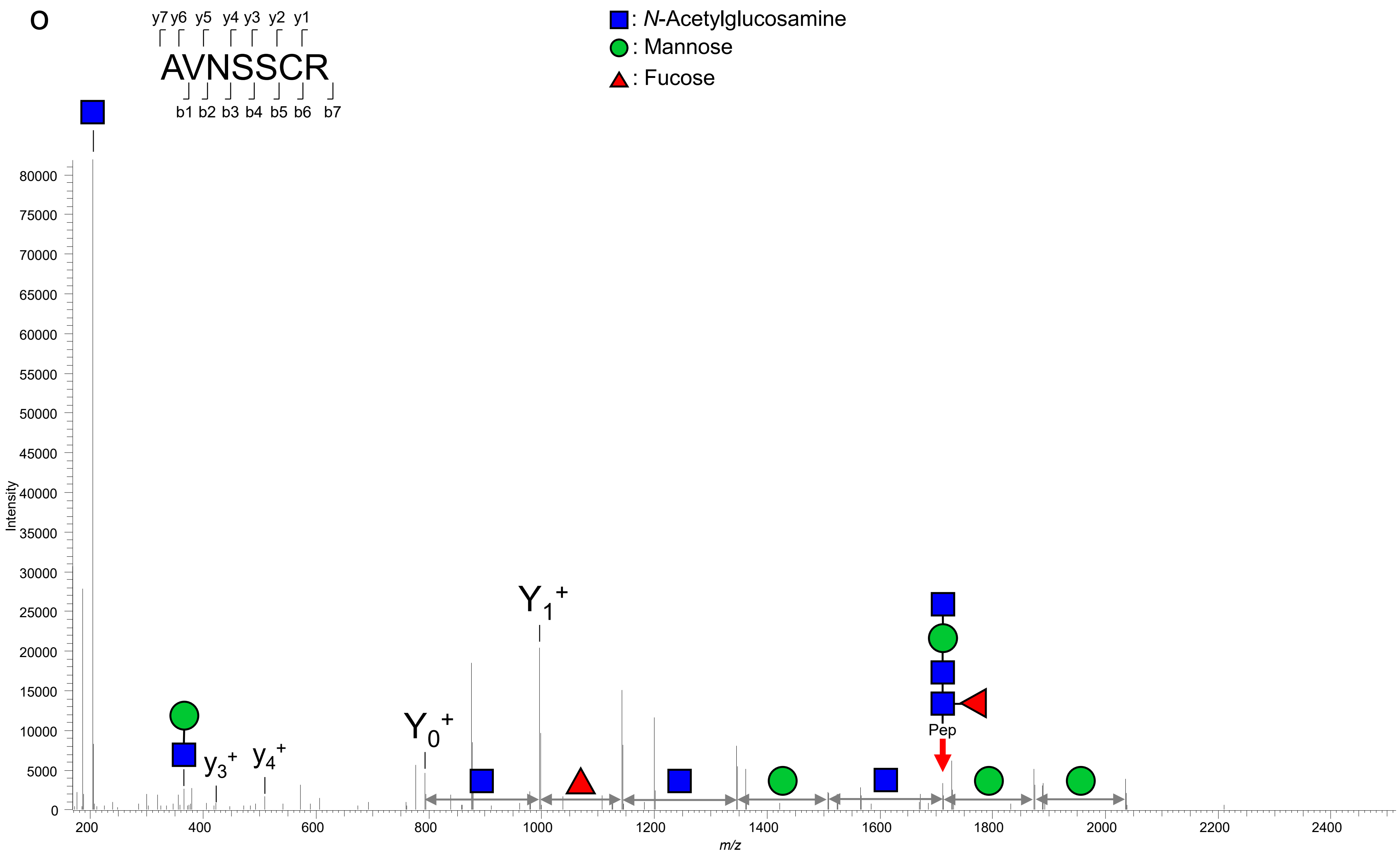

Supplementary Figure S1. (o) Product ion spectrum of prolow-density lipoprotein receptor-related protein 1 glycopeptide arising from precursor ion at *m/z*814.3300 (charge: 3)

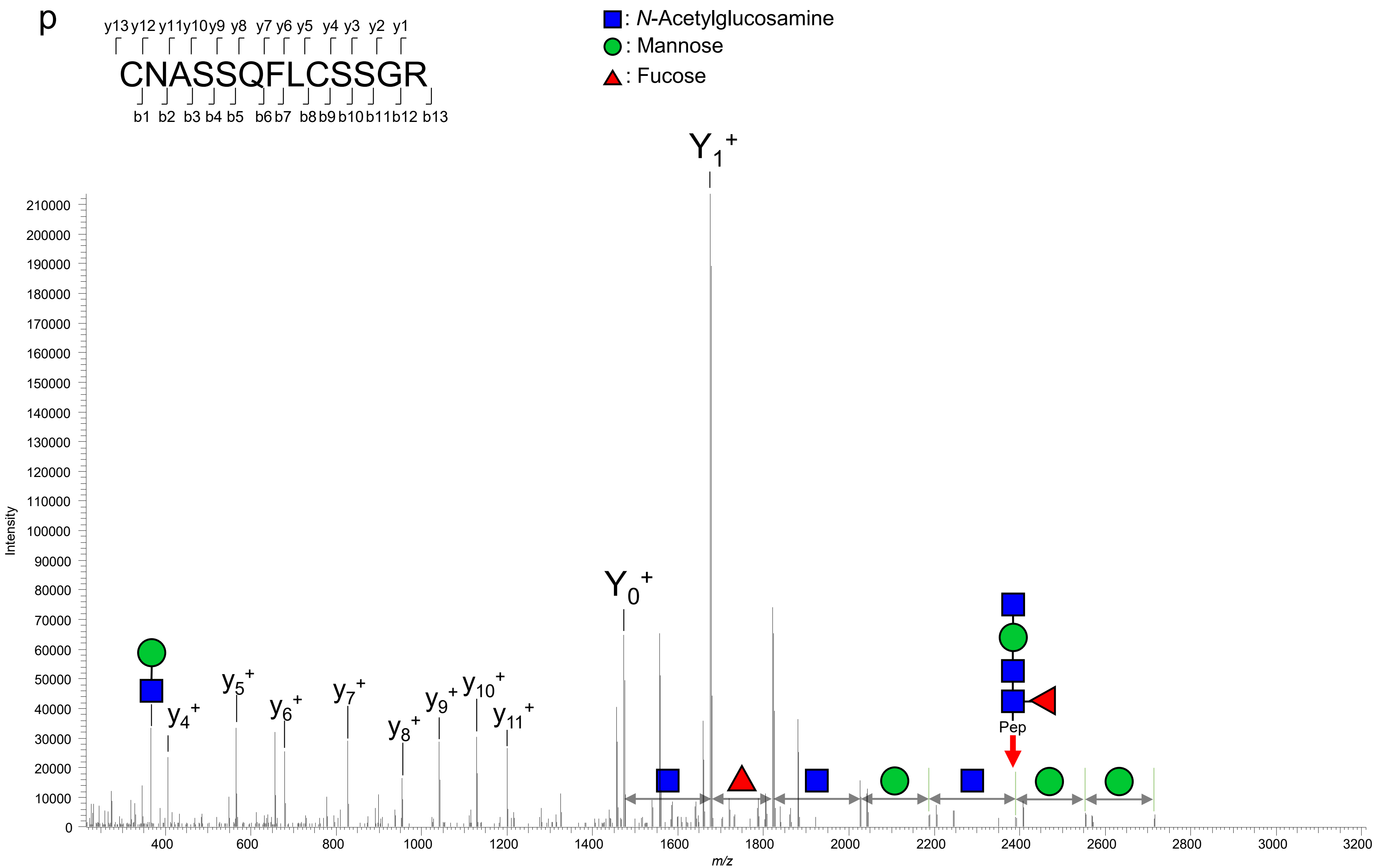

Supplementary Figure S1. (p) Product ion spectrum of prolow-density lipoprotein receptor-related protein 1 glycopeptide arising from precursor ion at *m/z*1041.0829 (charge: 3)

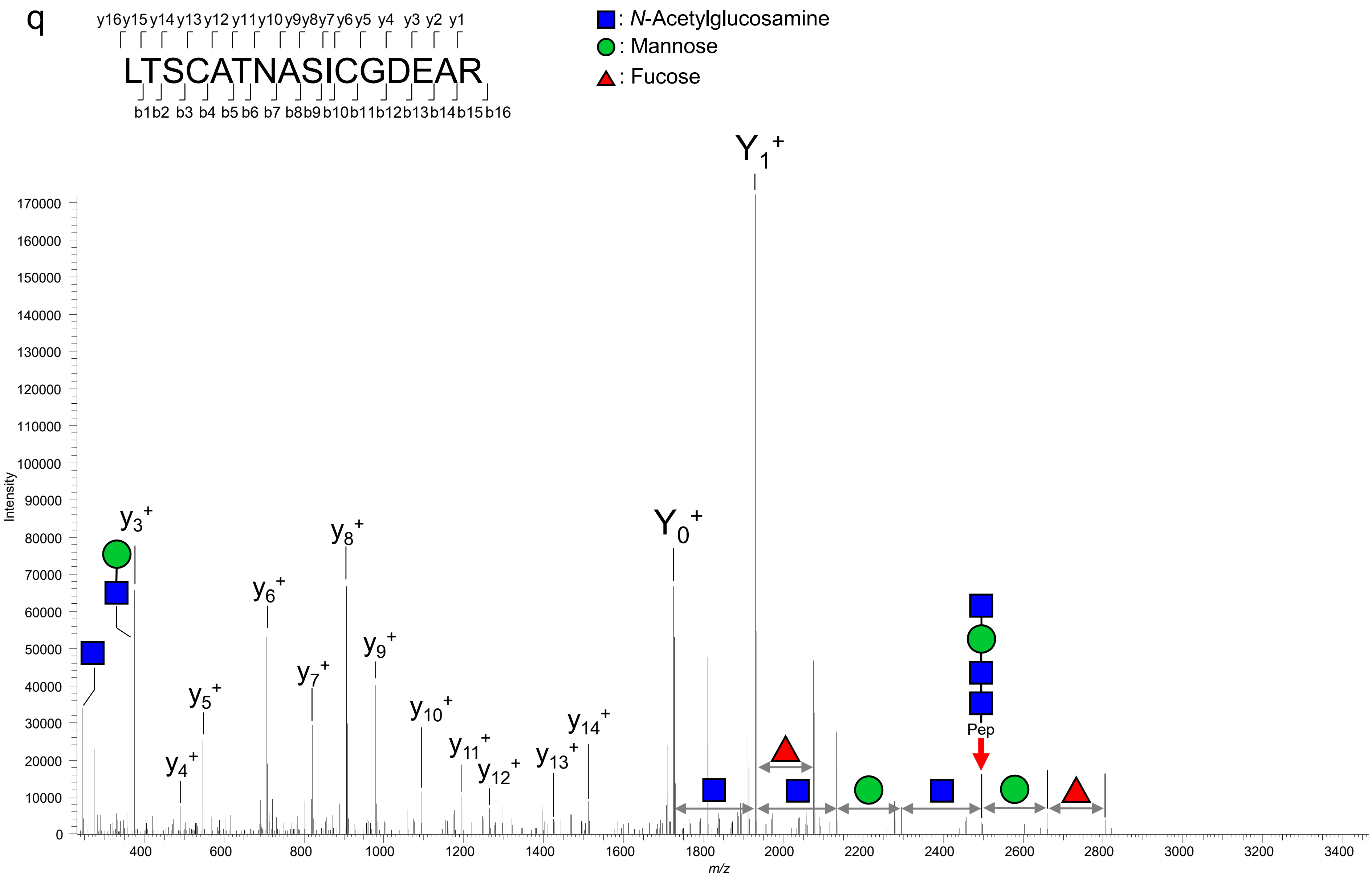

Supplementary Figure S1. (q) Product ion spectrum of prolow-density lipoprotein receptor-related protein 1 glycopeptide arising from precursor ion at *m/z*1125.1271 (charge: 3)

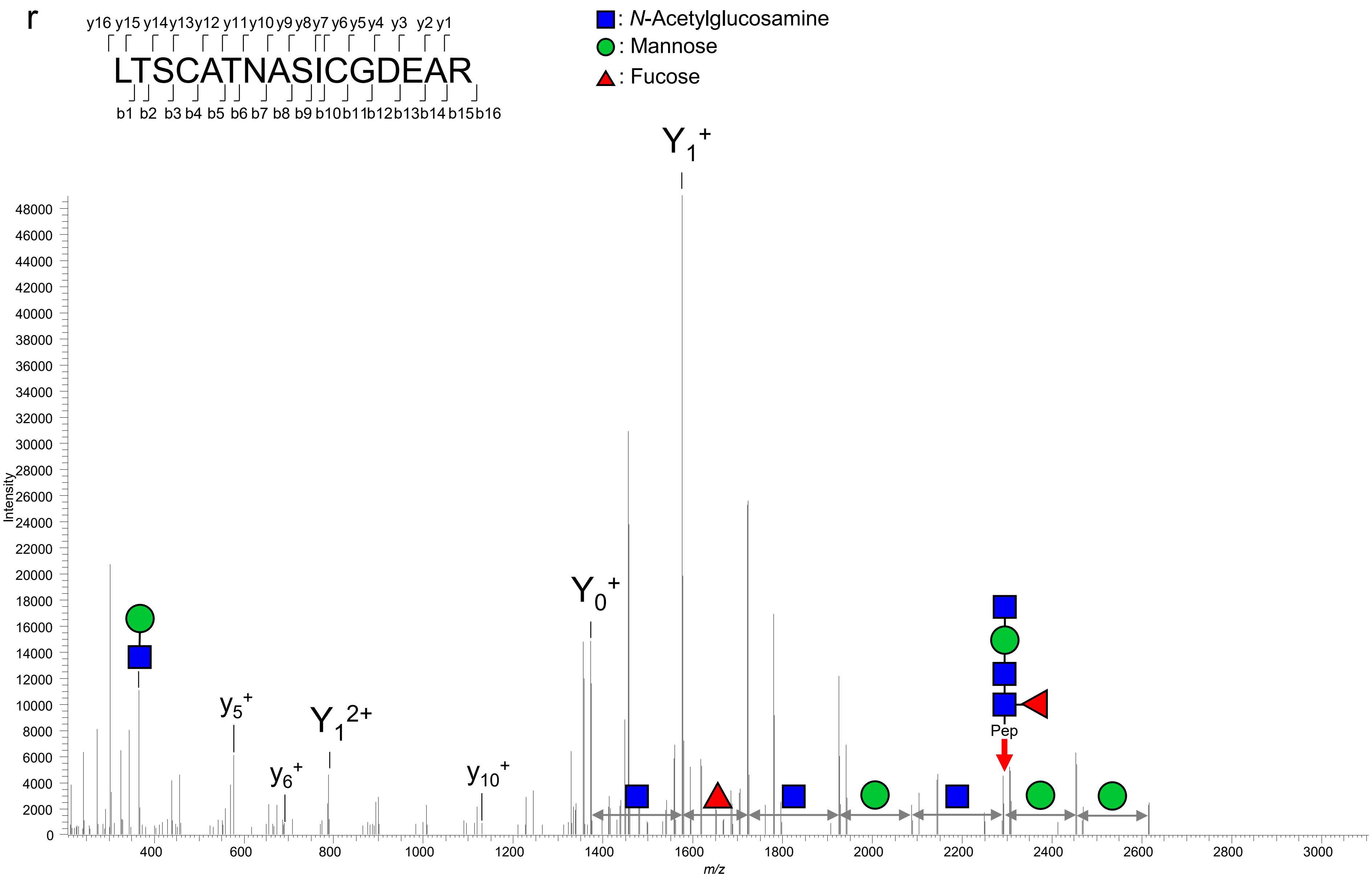

Supplementary Figure S1. (r) Product ion spectrum of prolow-density lipoprotein receptor-related protein 1 glycopeptide arising from precursor ion at *m/z*1007.4698 (charge: 3)

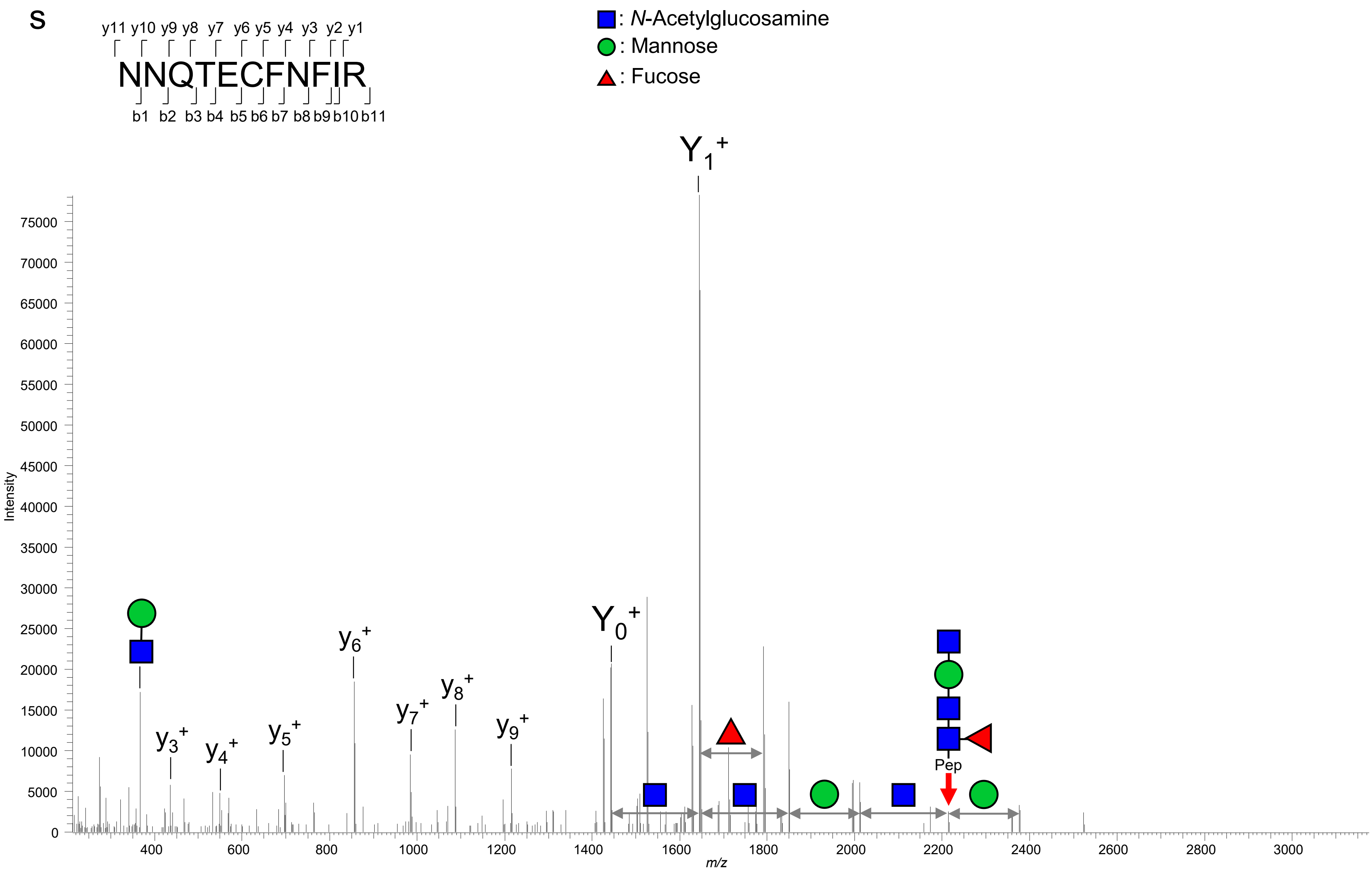

Supplementary Figure S1. (s) Product ion spectrum of semaphorin 4C glycopeptide arising from precursor ion at *m/z*1030.7586 (charge: 3)

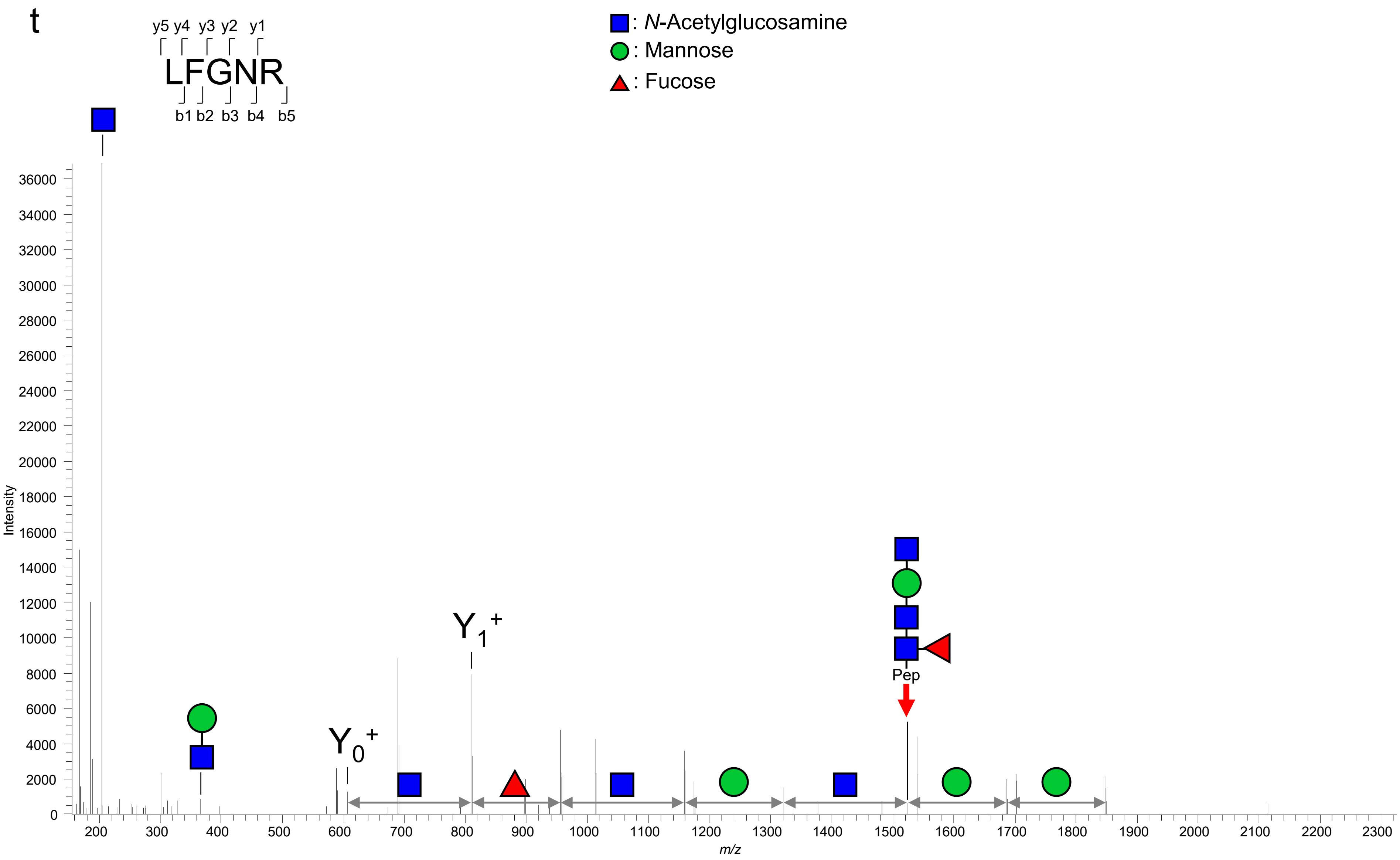

Supplementary Figure S1. (t) Product ion spectrum of tetratricopeptide repeat protein 31 glycopeptide arising from precursor ion at *m/z*751.9879 (charge: 3)

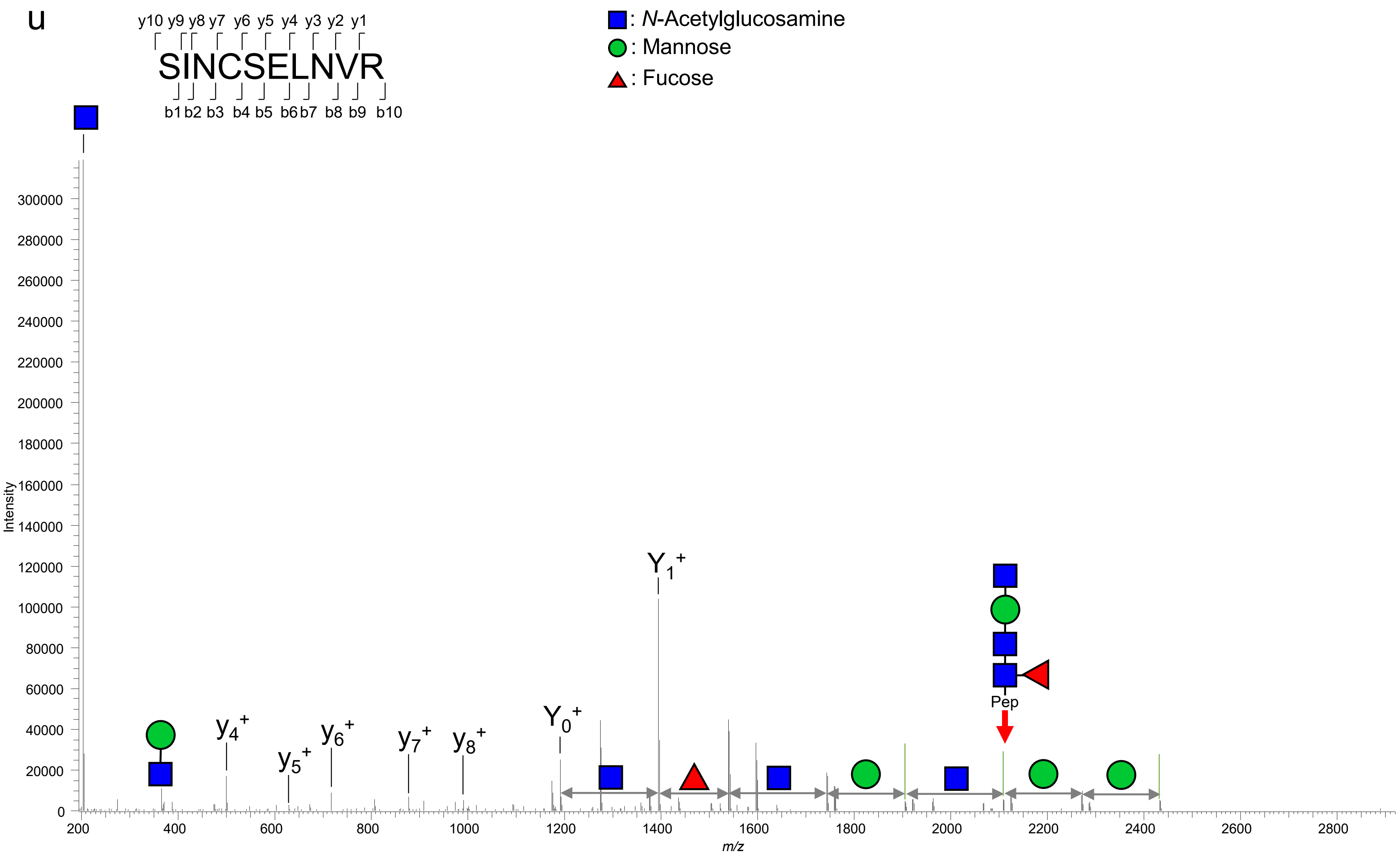

Supplementary Figure S1. (u) Product ion spectrum of tomoregulin-1 glycopeptide arising from precursor ion at *m/z*947.0677 (charge: 3)

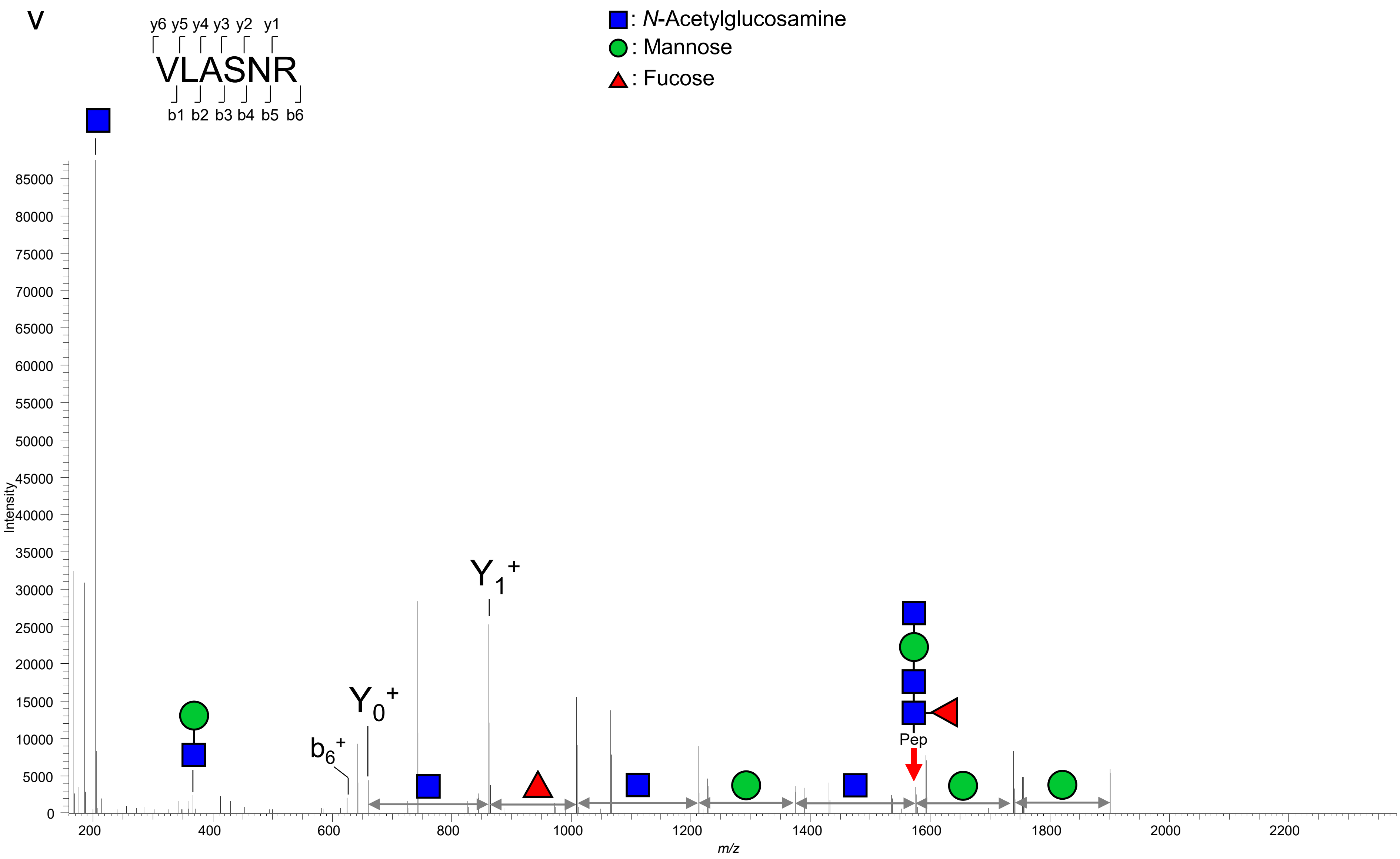

Supplementary Figure S1. (v) Product ion spectrum of voltage-dependent calcium channel subunit alpha-2/delta-2 glycopeptide arising from Precursor ion at *m/z*769.6707 (charge: 3)

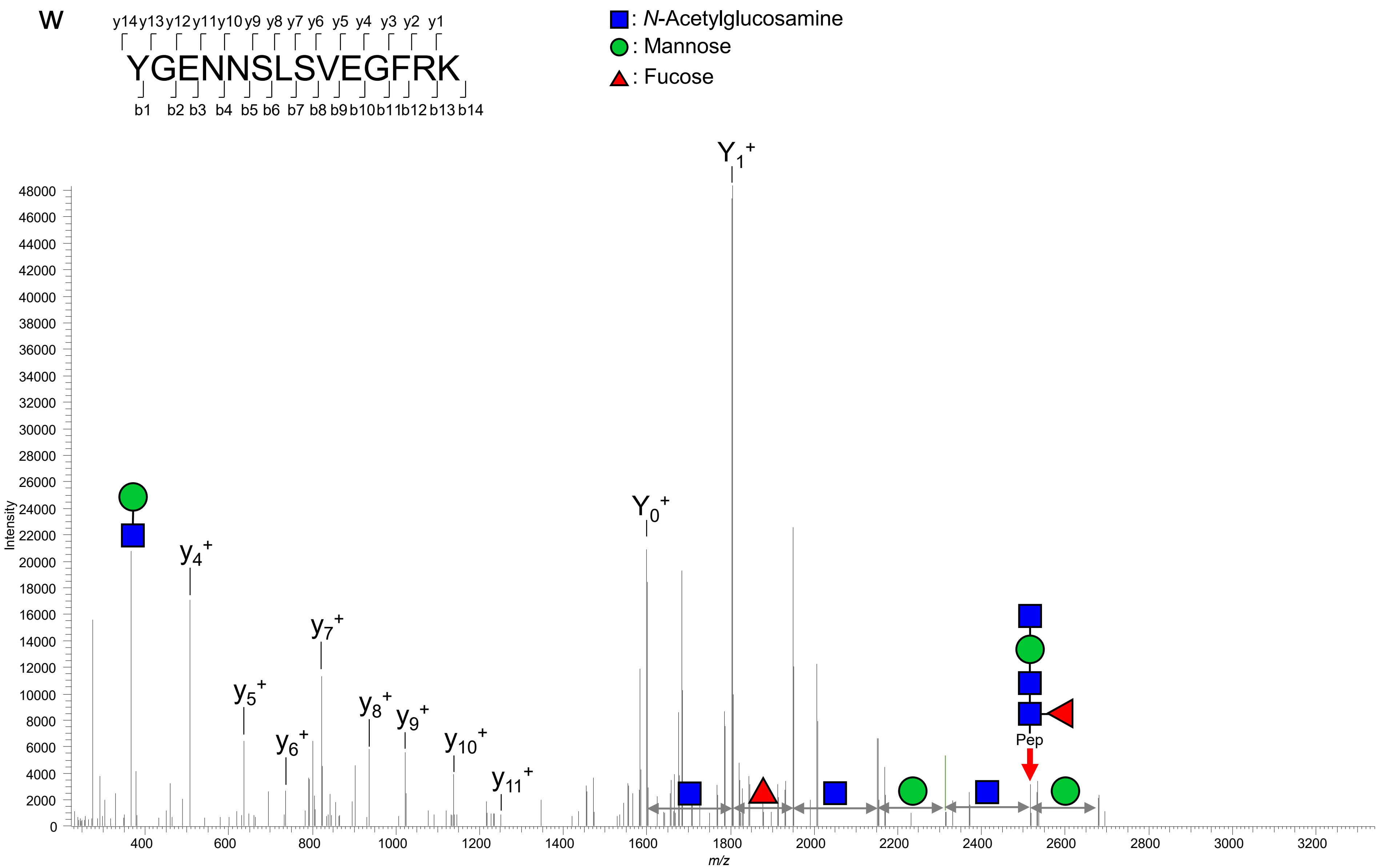

Supplementary Figure S1. (w) Product ion spectrum of zinc transporter zip 6 glycopeptide arising from precursor ion at *m/z*1083.1353 (charge: 3)

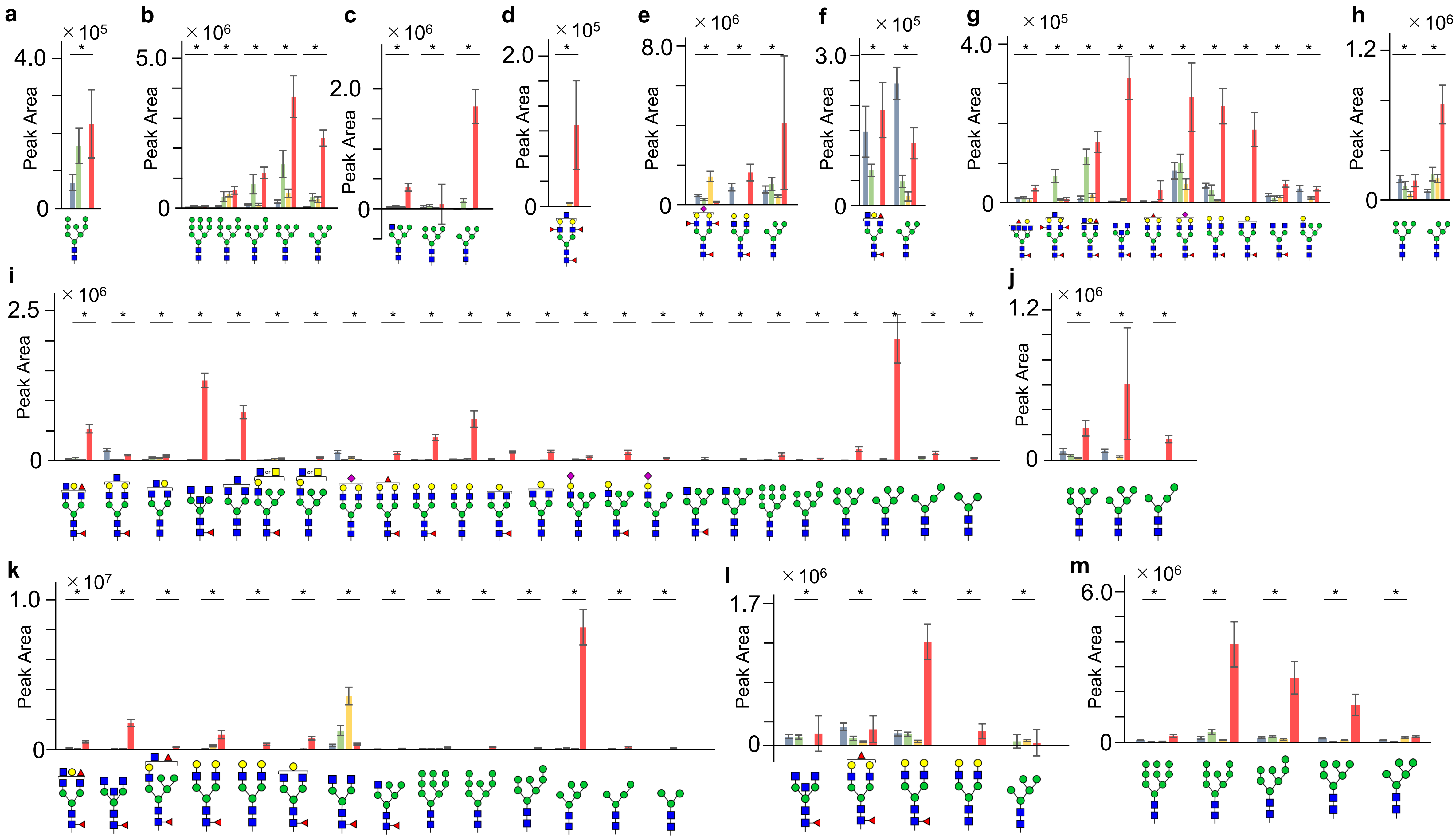
a
b
c
d
e
f
g
h
i
j
k
l
m
Peak Area
× 10^5
× 10^6
× 10^7
4.0
5.0
2.0
8.0
3.0
1.2
2.5
1.0
1.7
6.0
0
*
or

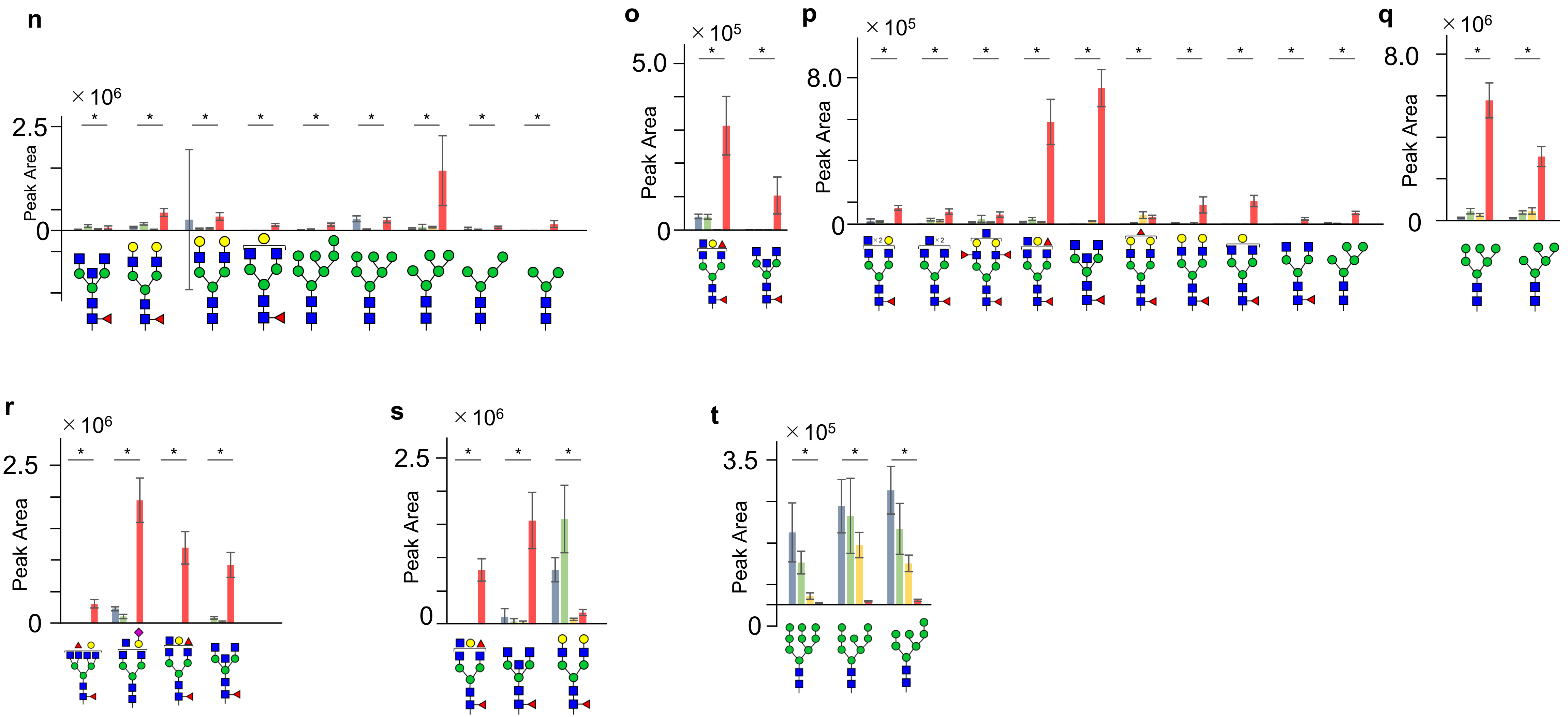

Supplementary Figure S2. Glycoform peak area of neural cell adhesion molecules.

(a) NrCAM N83, (b) NrCAM N222, (c) NrCAM N494, (d) NrCAM N790, (e) NrCAM N854, (f) NrCAM N989, (g) NrCAM N1006, (h) NrCAM 1017, (i) L1CAM N288, (j) L1CAM N474, (k) L1CAM N668, (l) L1CAM N723, (m) L1CAM N847, (n) L1CAM N977, (o) NCAM2 N217, (p) NCAM2 N445, (q) NCAM2 N473, (r) NEO1 N461, (s) NEO1 N628, (t) NEO1 N1297

Blue bar: iPSC, Green bar: NSC, Yellow bar: NPC, Red bar: DA. *: q-value 2D < 0.01.
